# Chelation enables selectivity control in enantioconvergent Suzuki–Miyaura cross-couplings on acyclic allylic systems

**DOI:** 10.1038/s41557-023-01430-8

**Published:** 2024-02-08

**Authors:** Violeta Stojalnikova, Stephen J. Webster, Ke Liu, Stephen P. Fletcher

**Affiliations:** Chemistry Research Laboratory, University of Oxford, Oxford, United Kingdom

**Keywords:** Asymmetric catalysis, Synthetic chemistry methodology, Synthetic chemistry methodology, Asymmetric synthesis

## Abstract

Asymmetric Suzuki–Miyaura cross-couplings with aryl boronic acids and allylic electrophiles are a powerful method to convert racemic mixtures into enantioenriched products. Currently, enantioconvergent allylic arylations are limited to substrates that are symmetrical about the allylic unit, and the absence of strategies to control regio-, *E*/*Z*- and enantioselectivity in acyclic allylic systems is a major restriction. Here, using a system capable of either conjugate addition or allylic arylation, we have discovered the structural features and experimental conditions that allow an acyclic system to undergo chemo- and regioselective, enantioconvergent allylic Suzuki–Miyaura-type arylation. A wide variety of boronic acid coupling partners can be used, and both alkyl and aromatic substituents are tolerated on the allylic unit so that a wide variety of structures can be obtained. Preliminary mechanistic studies reveal that the chelating ability of the ester group is crucial to obtaining high regio- and enantioselectivity. Using this method, we were able to synthesize the natural products (*S*)-curcumene and (*S*)-4,7-dimethyl-1-tetralone and the clinically used antidepressant sertraline (Zoloft).

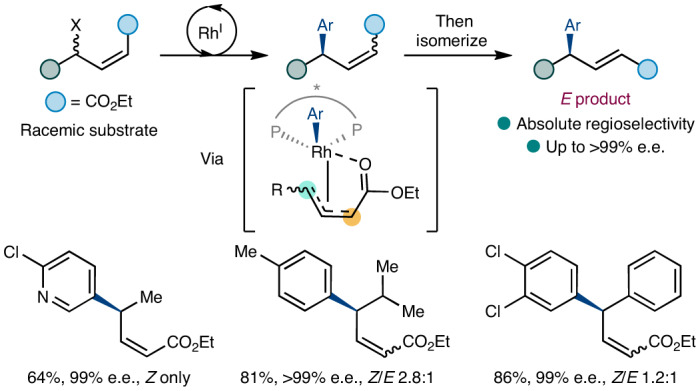

## Abstract (web summary)

Achieving selectivity control in allylic arylations is a long-standing challenge in catalysis. Now a rhodium-catalysed system demonstrates chemo-, regio- and enantioselectivity, enabling Suzuki–Miyaura-type arylation with racemic, non-symmetrical, acyclic allylic systems; chelation is speculated to facilitate oxidative addition and enable both enantiomers of the starting material to converge onto a single product.

## Main

The emergence of transition-metal catalysis has given chemists a diverse array of techniques to construct C–C bonds. Enantioselective methods for forming C–C bonds while simultaneously generating single isomer products have transformed synthetic planning^1^. In particular, transition-metal-catalysed asymmetric allylic addition reactions have emerged as indispensable tools in organic synthesis, owing to their capacity to generate C(*sp*^3^)-rich molecules. Tremendous advances have been made in the development of Pd-, Ir-, Ni- and Cu-catalysed enantioselective substitution reactions of allylic species with a variety of organometallic nucleophiles^2–14^. However, there are relatively few examples of using aryl nucleophiles in these transformations^15,16^. While regio- and enantioselective allylic arylations have been reported^17,18^, these have been restricted to reactions that use prochiral starting materials, and the use of heteroaromatic nucleophiles in these transformations is limited.

**Fig. 1:**
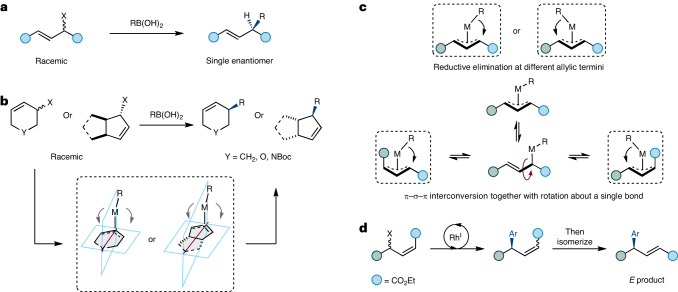
Rh-catalysed enantioconvergent couplings. **a**, Generalized representation of asymmetric Suzuki–Miyaura-type cross-coupling with racemic allylic electrophiles. **b**, Previously reported reactions used starting materials that had a plane of symmetry (purple line) after oxidative addition (ignoring metal/ligand stereochemistry). **c**, When using acyclic, non-symmetrical systems, multiple products could arise via reductive elimination at either termini to give two regioisomers; *Z*/*E* isomers can also be produced by interconversion and bond rotation, and enantiomers can also be produced, so a total of eight stereoisomers can be formed. **d**, In this work, enantioconvergent Suzuki–Miyaura cross-coupling reactions occur using acyclic non-symmetrical allylic systems that are completely regioselective. Different double bond isomeric ratios (from 1:1 to *Z* only) are observed, and double bond mixtures can be converted to the *E* isomer. M, metal-ligand complex.

The Suzuki–Miyaura reaction has seen tremendous advances over the last 40 years, resulting in the development of diverse methods for the formation of C–C bonds^19,20^. Typically, the Suzuki–Miyaura involves C(*sp*^2^)–C(*sp*^2^) formation between (hetero)arenes, and the reaction is so widely used that some drug candidate libraries are skewed towards planar molecules^21–23^. Several enantioselective cross-coupling reactions involving boronic acid derivatives are known^24–26^. Suzuki-type cross-coupling reactions with allylic electrophiles preserve the advantage of using commercially available and experimentally convenient boronic acids while controlling the stereochemistry of a new chiral centre (Fig. 1a). Asymmetric reactions using racemic starting materials are attractive because, in principle, racemates are more readily available than prochiral starting materials—but methods that can effectively use racemic starting materials are currently limited. Asymmetric additions of (hetero)aryl boronic acids to cyclic allylic halides^27–30^ (Fig. 1b) have been reported and applied in the synthesis of complex molecules like Zejula (niraparib)^30^, tafluprost^31^ and YZJ-1139(1) (ref. ^32^). These Rh-catalysed deracemizing transformations are currently limited to electrophiles that, after oxidative addition, are achiral about the allyl unit, which allows ligand-controlled enantioselective C–C bond formation (Fig. 1b)^33^. Another key problem is that only cyclic electrophiles can be used. Asymmetric addition to racemic non-cyclic allylic systems is challenging because the double bond geometry in those electrophiles is not fixed, allowing π–σ–π interconversion together with single bond rotation, which would be expected to give a mixture of products^34–36^. Further, in linear substrates without pseudosymmetry about the allyl unit, reductive elimination can occur at either termini to give regioisomeric products, and so eight products, possibly of similar energies, would be obtained (Fig. 1c).

Learning how to perform asymmetric Suzuki–Miyaura reactions on racemic acyclic substrates, particularly those that are unsymmetrical (inequivalent π-allyl termini) would address a longstanding selectivity challenge in catalysis and provide a useful strategy to synthesize biologically active molecules. Regiospecific allylic arylations of acyclic, non-pseudosymmetric compounds have been performed on prochiral substrates or enantiospecifically, that is, using enantiomerically enriched starting materials^37^. Here we report a rhodium-catalysed system where after oxidative addition to a *Z* double bond containing allylic electrophile, reductive elimination occurs only at one position, resulting in regio- and enantioselective cross-coupling with good to modest *Z*/*E* selectivity. It is remarkable that the substrate, an α,β-unsaturated ester, can undergo 1,4-additon as well as allylic arylation and that we observe absolute chemoselectivity for allylic arylation in our rhodium-catalysed system.

## Results and discussion

Several relatively simple acyclic allylic halides were examined to see if they would be suitable for Rh-catalysed allylic arylations; these tended to undergo a variety of side reactions or were unreactive. Eventually we found that when γ-carbonate enoate (±)-**1** was used, better results could be obtained.

Coupling (±)-**1** with phenylboronic acid **2a** using methods previously reported^27^ for cyclic allylic halides and aryl boronic acids was largely unsuccessful. No desired product was obtained using (2,2′-bis(diphenylphosphino)-1,1′-binaphthyl) (BINAP) **L1** and (4,4'-bis(bis(3,5-dimethylphenyl)phosphino)-2,2',6,6'-tetramethoxy-3,3'-bipyridine) (Xyl-P-Phos) **L2** (Table 1, entries 1 and 2) and conjugate addition (**3a′**) was observed with (5,5′-Bis(diphenylphosphino)-4,4′-bi-1,3-benzodioxole) (SegPhos) **L3** (entry 3). However, **L4** (entry 4) showed a complete reversal in chemoselectivity from **L3**, so that allylic arylation rather than 1,4-addition was observed. The arylation proceeded with complete regioselectivity and a 2.3:1 mixture of *Z*/*E* products was obtained in 73% yield. Both the *Z* and the *E* products had the same absolute stereochemistry at the newly formed stereogenic centre, as determined by hydrogenation of the alkenes using Wilkinson’s catalyst into a common product (Supplementary Information pages 3–7 contain details), which had an e.e. of 92%.

**Table 1:** Reaction optimization


Entry	Ligand	Additive	*T* (°C)	Yield of 3a (%)	e.e. (%)	*Z*/*E* of 3a	Yield of 3a′ (%)
1	**L1**	w/o	60	0	-	-	0
2	**L2**	w/o	60	0	-	-	0
3	**L3**	w/o	60	2	-	-	31
4	**L4**	w/o	60	73	92	2.3	0
5	**L5**	w/o	60	72	90	1.3	0
6	**L6**	w/o	60	55	84	1.2	0
7	**L7**	w/o	60	83	98	3.4	0
8	**L7**	w/o	r.t.	7	98	-	0
9^a^	**L7**	w/o	r.t.	5	97	-	0
10^b^	**L7**	w/o	r.t.	80	90	3.4	0
11	**L7**	Zn(OTf)_2_	r.t.	94	98	4.0	0
12	**L7**	AgOTf	r.t.	36	98	4.1	0
13	**L7**	TfOH	r.t.	73	97	3.1	0

Conditions were as follows: [Rh(cod)OH]_2_ (2.5 mol%), ligand (6.0 mol%), Cs_2_CO_3_ (1.0 equiv.), additive (0.2 equiv.), phenylboronic acid (2.0 equiv.), 16 h. ^a^50 wt% aqueous CsOH used as a base. ^b^Toluene used as a solvent. *T*, temperature; r.t., room temperature; THF, tetrahydrofuran; cod, cyclooctadiene; w/o, without.

Further investigations revealed that **L7** gave the highest enantioselectivity, yield and *Z*/*E* ratio of the ligands we examined. Generally, we observed higher enantio- and regioselectivities, but incomplete conversion, at lower temperatures. We examined an array of bases, solvents and additives (Supplementary Information pages 104–105 contain details) and found that full conversion could be achieved using 20 mol% Zn(OTf)_2_ (Tf, triflate) at room temperature, an additive that had previously been found to improve turnover frequency^38^ and substrate activation^39^ in Rh-catalysed reactions. These conditions conserve the high enantioselectivity (98% e.e.) and result in higher *Z*/*E* ratios than at elevated temperatures.

With optimized conditions in hand, we examined the scope of the reaction (Table 2) with respect to the boronic acid coupling partner. The reported *Z*/*E* ratios are based on ^1^H NMR spectroscopy of the crude reaction mixture. When desired, we found that we could (as in the case of **3a** described previously) separate the *Z* and *E* isomers by careful column chromatography, but for ease of characterization, the yield and e.e. values of compounds reported in Tables 2 and 3 were determined on the product of hydrogenation of the alkenes obtained. Hydrogenation of the alkenes using Wilkinson’s catalyst converted the *Z*/*E* mixture of compounds **3** and **5** obtained in the arylation reaction to a common product.

**Table 2:**
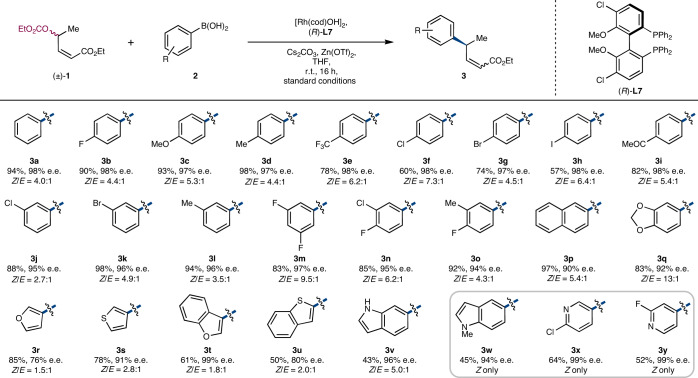
Scope of boronic acids

Reaction conditions were as follows: [Rh(cod)OH]_2_ (2.5 mol%), **L7** (6.0 mol%), (±)-**1** (0.4 mmol, 1.0 equiv.), **2** (2.0 equiv.), Cs_2_CO_3_ (1.0 equiv.), Zn(OTf)_2_ (20 mol%), THF (0.1 M), r.t., 14 h. All experiments were performed on the 0.4 mmol scale. All compounds were isolated as single regioisomers (r.r. >99:1). *Z*/*E* ratios were determined by ^1^H NMR spectroscopy on crude reaction mixtures. Yields were determined by subsequent hydrogenation of the product mixture. The e.e. values were determined by hydrogenation of the product mixture and SFC analysis using a chiral non-racemic stationary phase. Absolute configurations were assigned by analogy to product **3c**, which was converted to (*S*)-curcumene as determined by comparing optical rotation values to those previously reported.

**Table 3:**
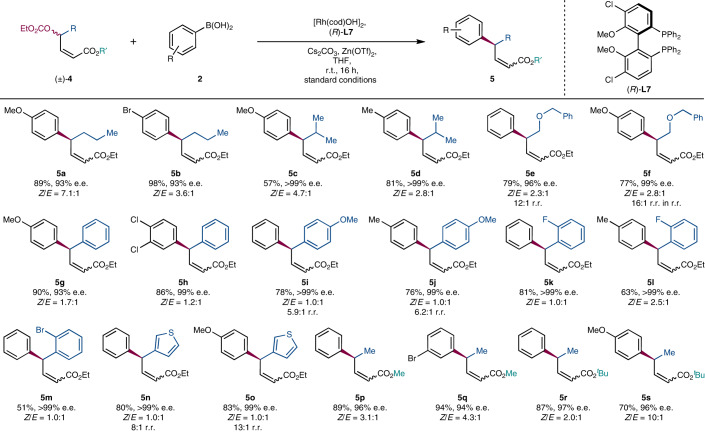
Variation in allylic carbonate

Reaction conditions were as follows: [Rh(cod)OH]_2_ (2.5 mol%), **L7** (6.0 mol%), (±)-**1** (0.4 mmol, 1.0 equiv.), **2** (2.0 equiv.), Cs_2_CO_3_ (1.0 equiv.), Zn(OTf)_2_ (20 mol%), THF (0.1 M), r.t., 14 h, 0.4 mmol. Unless stated otherwise, all compounds were isolated as single regioisomers (r.r. >99:1). *Z*/*E* ratios were determined by ^1^H NMR spectroscopy on crude reaction mixtures. Yields were determined by subsequent hydrogenation of the product mixture. The e.e. values were determined by hydrogenation of the product mixture and SFC analysis using a chiral non-racemic stationary phase.

A broad range of functionality was tolerated, including halogens (**3f**–**3h**, **3j** and **3k**), which can be used in subsequent reactions. Disubstituted boronic acids gave good yields and excellent enantioselectivities (**3m**–**3q**). Electron-poor aryl boronic acids were well tolerated with trifluoromethyl (**3e**) and ketone (**3i**) substituents giving the desired products in good yield and excellent e.e. Interestingly, *ortho*-substituted boronic acids furnished 1,4-addition products under the standard reaction conditions; using 2-methylphenylboronic acid gave the 1,4-addition product in 30% yield, as a 1.4:1 ratio of diastereomers with the major diastereoisomer having 80% e.e., and no allylic arylation product was observed. More challenging heteroaryl boronic acids^40,41^ displaying a variety of functional motifs and features common to pharmaceutical agents^42–44^ were also found to be suitable substrates for this method. For example, indole-derived boronic acids produced arylated unsaturated esters (**3v** and **3w**) in reasonable yields with >94% e.e.

Furan and thiophene-derived coupling partners also performed well (**3r**–**3u**), although a reduction in enantioselectivity was observed when using furan-3-ylboronic acid (76% e.e.), and the *Z*/*E* ratios with the five-membered heterocyclic boronic acids examined were lower (1.5:1–2.8:1). Pyridine-based boronic acids can also be used (**3y** and **3z**): products were obtained in moderate (52–64%) yield, but only the *Z* products were observed and the enantioselectivity in both cases examined was superb (99% e.e.). The 2-halide substituents on the pyridyl boronic acids are necessary for reactivity, but they can be removed or serve as handles for subsequent functionalization. Finally, alkyl and alkenyl boronic acids were found to be unsuccessful using our reaction conditions.

The scope of allylic carbonate coupling partners was also examined (Table 3). When we used another, arbitrarily chosen, primary-alkyl-substituted substrate, the reaction also gave good results; with *n*-propyl allylic carbonate (±)-**4**, product **5a** was formed in 89% yield with a 7:1 *Z*/*E* ratio and 93% e.e., and similar results were obtained with Br-substituted **5b** (98%, 3.6:1, 93% e.e.). When much more sterically demanding isopropyl-substituted (±)-**4** was examined, the products **5c** and **5d** were both obtained in >99% e.e. albeit with slightly lower yields and *Z*/*E* ratios.

The introduction of a benzyl-protected alcohol on the aliphatic chain of an allylic carbonate yielded the desired γ-arylation product, providing **5e** and **5f** in high yield and excellent enantioselectivity. Interestingly, we observed slightly decreased regioselectivity, from typical values of >99:1 regioisomeric ratio (r.r.), to 12:1 r.r. in **5e** and 16:1 r.r. in **5f**. This may be due to competitive coordination of the benzyl ether with rhodium, which could direct arylation in the α-position to the ester.

Remarkably, we found that the reaction even tolerated *sp*^2^-hybridized phenyl substituents without changing the ligand or any other reaction conditions. Using phenyl-substituted (±)-**4**, we obtained γ,γ-diaryl-substituted ester **5g** in 90% yield and 93% e.e., albeit with a lower 1.7:1 *Z*/*E* ratio. The 3,4-chlorodisubstituted product **5h** was furnished at 86% yield, 1.2:1 *Z*/*E* ratio and excellent enantioselectivity (99% e.e.).

Importantly, allylic substrates containing *ortho*-substituted phenyls (**5k**–**5m**) were tolerated in the reaction, all giving products with >99% e.e. As discussed previously, *ortho*-substituted boronic acid nucleophiles were found to undergo unselective 1,4-addition, and incorporating *ortho* groups into the allyl unit provides a means to access products with this substitution pattern. Product **5m** features an *ortho*-Br group, providing opportunities to elaborate the product.

A thiophene-substituted allylic carbonate gave γ-arylation products **5n** and **5o** with >80% isolated yields and excellent enantioselectivities (99% e.e.), but the branched products were not exclusively obtained here (8:1 r.r.). With highly electron-rich substituents on the allyl unit (**5i**, **5j**, **5n** and **5o**), some of the α-product was observed in the arylation, although the isolated yields of the desired chiral products are still good.

The system also performs well when the ester moiety is varied, and excellent yields and enantioselectivities were obtained in products featuring methyl (**5p** and **5q**) and bulky *tert*-butyl (**5r** and **5s**) esters.

**Fig. 2:**
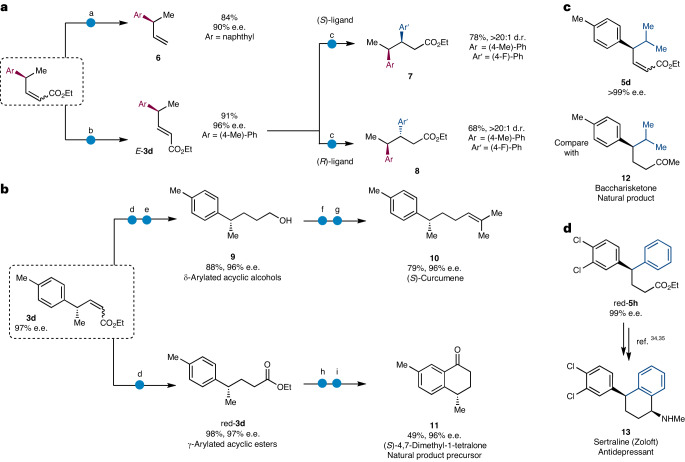
Product derivatization and application to natural product synthesis. **a**, Metathesis with ethylene to furnish terminal alkene **6** (top). Isomerization to the more stable *E* isomer ***E*-3d** and subsequent 1,4-addition to give either diastereoisomer **7** or **8** (bottom). **b**, Synthesis of natural product (*S*)-curcumene and (*S*)-4,7-dimethyl-1-tetralone. **c**, Product **5d** is similar in structure to baccharisketone. **d**, Formal synthesis of antidepressant sertraline from **5h**. ^a^Ethylene, Grubbs II, toluene, 75 °C. ^b^Ph_2_S_2_, 4CzIPN, THF, blue light-emitting diode (LED). ^c^[Rh(C_2_H_4_)_2_Cl]_2_, (*S*)- or (*R*)-BINAP, KOH, (4-fluoro)phenylboronic acid, dioxane/H_2_O, 60 or 80 °C. ^d^H_2_, [Rh(PPh_3_)_3_Cl], THF. ^e^LiAlH_4_, THF. ^f^DMP, THF, MeOH. ^g^Isopropyltriphenylphosphonium iodide, *n*-BuLi, THF, –15 °C. ^h^LiOH, THF, MeOH. ^i^TFA, TFAA, 0 °C.

The major *Z* products obtained in these reactions can be isolated in good yields. The products also feature an array of useful functionalities and can be further derivatized using well-established reactions (Fig. 2a). As described previously, to determine the e.e. values on most of our products, they were first hydrogenated. A metathesis with ethylene can be used to afford naphthyl-substituted **6** while conserving the enantiomeric ratio of the starting material (90% e.e.) in the product (90% e.e.), and these products are suitable for a variety of further transformations including olefin metathesis reactions. We can also convert the mixture of *E* and *Z* products into the thermodynamically stable *E* isomer *E*-**3d** by using a photocatalytic isomerization (91% yield) with high levels of stereoretention (98% enantiospecificity (e.s)). Both the starting materials and products in the Rh-addition reactions are α,β-unsaturated esters, which are well known Michael acceptors, and while we observed complete selectivity in favour of allylic arylation with compounds **1** and **4**, we anticipated that subsequent 1,4-additions would provide the opportunity to form two contiguous stereocentres where the stereochemistry of each centre is determined entirely by ligand control. Both diastereomers (**7** and **8**) could be accessed by 1,4-conjugate addition of an aryl group to *E*-**3d** using appropriate enantiomers of BINAP (>20:1 d.r. in each case).

To demonstrate the value of this method in synthesis, we made (*S*)-curcumene and (*S*)-4,7-dimethyl-1-tetralone (Fig. 2b). Reduction of α,β-unsaturated ester **3d** with Wilkinson’s catalyst gave γ-substituted ester red-**3d** (red, reduced), a key intermediate in the synthesis of both natural products. Treatment with LiAlH_4_ gave remotely substituted alcohol **9**, in good yield with minimal loss of stereochemical integrity. Oxidation with Dess–Martin periodinane and Wittig olefination furnishes (*S*)-curcumene **10**. The γ-arylated acyclic ester red-**3d** can also be hydrolysed and transformed into (*S*)-4,7-dimethyl-1-tetralone **11** by acid-promoted cyclization. Species **11** is a key intermediate in the total synthesis of several natural products, for example (–)-lacvigatin, (*S*)-ar-himachalene and (+)-erogorgiaene^45^. Our method provides a conceptually different approach to these targets and a means to prepare analogues by starting with different boronic acids. It should allow access to a variety of terpene analogues and derivatives by starting with different allyl partners; for example, isopropyl-substituted baccharishketone (**12**)^46^ is similar in structure to **5d** (Fig. 2c), which was obtained in 99% e.e. from **4b**.

Our method provides a straightforward and concise approach to γ,γ-diaryl-substituted carbonyls (Table 3, **5g**–**5m**) and γ,γ-aryl-heteroaryl-substituted carbonyls (**5n** and **5o**). Chiral non-racemic γ,γ-diaryl-substituted carbonyls and their derivatives are present in natural products, pharmaceuticals and bioactive compounds. Enantioenriched γ,γ-diaryl-substituted carbonyl compounds are generally prepared using optically enriched precursors^16,47^, although a few multistep^48^ or limited-in-scope^49^ catalytic asymmetric approaches are known. There is minimal precedent in direct and modular asymmetric formation of γ-arylated carbonyl compounds.

Cyclization and reductive amination of red-**5h** have been used to give sertraline^15^ (Zoloft) **13**, a potent antidepressant (Fig. 2d), which has recently been shown to have antiviral, antifungal and anticancer activity^50^.

**Fig. 3:**
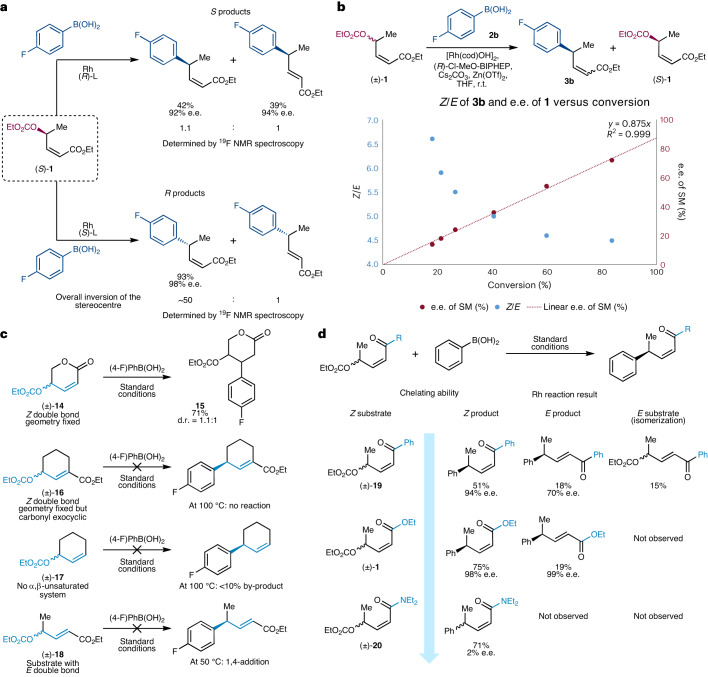
Mechanistic experiments. **a**, Reactions using enantiopure (99% e.e.) (*S*)-**1** starting material and different enantiomers of ligand (L). The *S* ligand gives an excellent *Z*/*E* ratio and 98% e.e., while the *R* ligand gives an ~1:1 mixture of double bond isomers and <95% e.e. **b**, Following a reaction with (±)-**1** over time (bottom plot) shows that when using boronic acid **2b**, as in the schematic (top), the product *Z*/*E* ratio decreases over time, and that one enantiomer of the starting material (SM) preferentially reacts so that the starting material becomes enantiomerically enriched. **c**, Control experiments with alternative allylic carbonates are consistent with the idea that γ-arylation can be achieved on allylic enones where the rhodium can chelate to the carbonyl, as **14**, **16**, **17** and **18** did not undergo allylic arylation. **d**, Control experiments using allylic carbonates with different carbonyl groups suggest that the directing or chelating ability of the carbonyl plays a key role in the selectivity of these reactions. R^2^, coefficient of determination.

Next, we preformed initial studies into the reaction mechanism by varying some key structural features of the substrate. We prepared (*S*)-**1** (99% e.e., as determined by supercritical fluid chromatography (SFC) analysis on a chiral non-racemic stationary phase; Supplementary Information page 254 and Supplementary Figure 11.51) and subjected it to the standard reaction conditions using both enantiomers of the ligand (Fig. 3a). Upon reaction with (*R*)-Cl-MeO-BIPHEP ((+)-2,2′-Bis(diphenylphosphino)-5,5′-dichloro-6,6′-dimethoxy-1,1′-biphenyl), (*S*)-**1** was transformed into an ~1:1 mixture of *E* and *Z* products, and both were obtained with high e.e. values (92% and 94% e.e.), but these values were lower than when using racemic **1**. When using (*S*)-Cl-MeO-BIPHEP, the *Z* product was obtained almost exclusively with 98% e.e. This suggests that on racemic material, one enantiomer forms the *Z* product with very high e.e., while the other enantiomer gives a mixture of *Z* and *E* products with slightly lower enantioselectivity. As seen in Tables 2 and 3, the final *Z*/*E* ratio depends on which boronic acid is used, and a decrease in the *Z*/*E* ratios is also observed while monitoring reactions over time (Fig. 3b). The relationship between structure and *Z*/*E* product ratio is complicated, but the experiment shown in Fig. 3a suggests that the ‘mismatched’ enantiomer of starting material and ligand leads to the formation of a mixture of *Z* and *E* product, and is at least partially responsible for the *Z*/*E* variation. As well as showing that product *Z*/*E* ratios can change during the course of the reaction, Fig. 3b also shows that the e.e. of the starting material increases in time, consistent with one enantiomer of starting material reacting faster than the other.

To establish what features of the starting material are required for these regioselective, enantioconvergent transformations, we prepared a series of model substrates (Fig. 3c). Cyclic γ-carbonate unsaturated ester (±)-**14**, with a double bond fixed in the *Z* configuration, underwent 1,4-addition to **15** instead of allylic arylation. Substrate (±)-**16** with an exocyclic carbonyl, on the other hand, is unreactive, even at 100 °C. This could be due to a number of reasons including having more substitution on the olefin or lack of Rh–carbonyl chelation, and 1,4-additions to such exocyclic electrophiles are rare. The simple allylic carbonate (±)-**17** also gave no arylation under our standard conditions and <10% of an unknown product when forced. In starting materials featuring an *E* double bond ((±)-**18** in Fig. 3c), the allylic system is again unreactive (Fig. 3c) under standard reaction conditions. However, upon heating to 50 °C, 1,4-addition is observed. Overall, these experiments are consistent with γ-arylation being achieved on allylic enones where the rhodium can chelate to the carbonyl.

The reactivity profile observed when using substrates with different carbonyls (Fig. 3d) is consistent with the chelating or directing ability of the carbonyl playing a key role in the reaction outcome. Amides are prominent directed metalation groups^51^, and under the standard reaction conditions, amide (±)-**20** furnishes the *Z* product, albeit as an almost racemic mixture. Ketone (±)-**19**, on the other hand, furnishes *Z* product in 51% yield and 94% e.e., but the *E* product is formed in lower (70%) e.e., and we also recovered some isomerized *E*-ketone while using (±)-**19**. Chelation-promoted reactivity is consistent with substrates (±)-**14**, (±)-**17** and (±)-**18** not undergoing arylation. We speculate that the Rh–carbonyl interaction facilitates oxidative addition and enables a pathway by which both enantiomers of the starting material may converge on a single Rh intermediate before reductive elimination to give a single enantiomer and regioisomer of product.

**Fig. 4:**
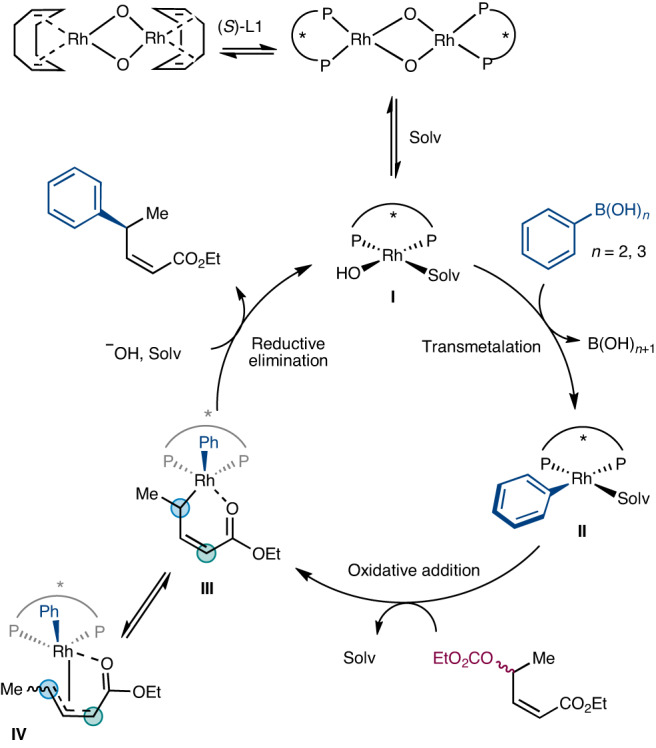
A preliminary mechanistic proposal for these reactions. We speculate that chelation facilitates oxidative addition, and provides pathways where both enantiomers of the starting material can converge onto a single Rh intermediate before reductive elimination can give one regioisomer and enantiomer of product. Solv, solvent molecule.

The necessary components for the allylic arylation appear to be an allylic leaving group featuring a *Z*-olefin, conjugated to an ester. Based on the above experimental observations and previous mechanistic studies^33^, we propose a tentative mechanism for Rh-catalysed allylic arylation of acyclic substrates to form the major *Z* product (Fig. 4). First, complex **I** undergoes rapid transmetalation of aryl boronic acid to give **II**. Complex **II** undergoes oxidative addition with the substrate to form the intermediate **III**, which is likely in equilibrium with **IV**. Enantio-determining irreversible reductive elimination leads to the observed *Z* product. A combination of π–σ–π interconversion and rotation about single bonds provides opportunities for both enantiomers of starting material to converge on a single product.

## Conclusion

We developed an asymmetric Suzuki–Miyaura-type arylation of non-symmetrical, acyclic allylic systems. A combination of chemo-, regio- and enantioselectivity is observed when using γ-carbonate *Z*-enoates. A broad range of electrophiles and nucleophiles can be used. This cross-coupling offers a straightforward and modular asymmetric approach to remotely substituted esters and alcohols—which are widely seen in bioactive molecules. We used the method in the asymmetric syntheses of natural products (*S*)-curcumene and (*S*)-4,7-dimethyl-1-tetralone and an enantioselective and modular route to sertraline (Zoloft). It is anticipated that the development of this reaction and the observation that chelation is key to obtaining selective allylic arylation will facilitate the development of other highly selective asymmetric reactions using complex racemic starting materials.

## Methods

General procedures for the cross-coupling of aryl boronic acids and acyclic allylic electrophiles and the subsequent reduction of the products are presented in the following sections.

### General procedure A (rhodium-catalysed arylation)

All reactions were carried out under an inert argon atmosphere using standard Schlenk techniques with all reagents weighed open to air.

#### Preparation of the catalyst

The [Rh(cod)OH]_2_ (20.5 mg, 0.045 mmol, 2.5 mol%) and (*R*)-Cl-MeO-BIPHEP (70.4 mg, 0.108 mmol, 6.0 mol%) were added to a 25 ml flask containing a stirring bar and dissolved in dry THF (18.0 ml) under an argon atmosphere at ambient temperature (23 °C). This solution was stirred for 5 minutes and used (in the same hour) for four asymmetric reactions on a 0.4 mmol scale.

#### Cross-coupling reaction

Boronic acid (0.80 mmol, 2.0 equiv.), Cs_2_CO_3_ (130.3 mg, 0.40 mmol, 1.0 equiv.) and Zn(OTf)_2_ (29.1 mg, 0.08 mmol, 0.2 equiv.) were added to a 7 ml vial containing a stirring bar. To this vial a stock solution of the rhodium hydroxy complex (4.0 ml) was added via syringe under an argon atmosphere. The allylic carbonate (0.40 mmol, 1.0 equiv.) was added via microsyringe and the reaction mixture was stirred at ambient temperature (23 °C). The mixture was diluted with hexane (4.0 ml) and filtered through a plug of silica. The crude was loaded onto a Chem Tube-Hydromatrix, and flash column chromatography was performed to afford the desired products.

### General procedure B (hydrogenation)

Hydrogen (~1 atm, from a balloon) was bubbled through a solution of [RhCl(PPh_3_)_3_] (37.1 mg, 0.040 mmol, 0.10 equiv.) in THF (0.70 ml) for 5 minutes. A mixture of *Z* and *E* products obtained from the rhodium arylation reaction (general procedure A) dissolved in THF (0.30 ml) was then added via syringe to the catalyst solution. Hydrogen (~1 atm, from a balloon) was bubbled through the reaction mixture for a further 5 minutes. The reaction mixture was equipped with a hydrogen balloon and stirred at ambient temperature (23 °C) for 16 h. Then the mixture was diluted with hexane (4.0 ml) and filtered through a plug of silica. The crude was loaded onto a Chem Tube-Hydromatrix, and flash column chromatography was performed to afford the desired product.

All modifications from these procedures are specified within the Supplementary Information of the Article.

## Online content

Any methods, additional references, Nature Portfolio reporting summaries, source data, extended data, supplementary information, acknowledgements, peer review information; details of author contributions and competing interests; and statements of data and code availability are available at 10.1038/s41557-023-01430-8.

### Supplementary information

Supplementary Information Supplementary figures, general methods, detailed experimental and analytical data, NMR spectra and SFC chromatograms, as well as all other supporting data for the study.

## Data Availability

The online version of this paper provides Supplementary Information, encompassing supplementary figures, general methods, detailed experimental and analytical data, NMR spectra and SFC chromatograms, as well as all other supporting data for the study.
