# Supplementary material for: Chelation enables selectivity control in enantioconvergent Suzuki–Miyaura cross-couplings on acyclic allylic systems
Source: Nat Chem. 2024 Feb 8;16(5):791–9. doi: 10.1038/s41557-023-01430-8 (PMC11087250; doi:10.1038/s41557-023-01430-8)
Supplement: Supplementary file 1 — Supplementary figures, general methods, detailed experimental and analytical data, NMR spectra and SFC chromatograms, as well as all other supporting data for the study. [file 41557_2023_1430_MOESM1_ESM.pdf]

nature chemistry

Article

https://doi.org/10.1038/s41557-023-01430-8

Chelation enables selectivity control in enantioconvergent Suzuki–Miyaura cross-couplings on acyclic allylic systems

In the format provided by the authors and unedited

Table of Contents

1. General Methods 2
2. Procedures for the rhodium-catalyzed reactions 3
2.1. General procedure A (Rhodium-catalyzed arylation) 3
2.2. General procedure B (Hydrogenation) 3
2.3. Rh-catalyzed reactions of (±)-**1** 4
1,4-Addition of *ortho*-boronic acid to (±)-**1** 38
2.4. Rh-catalyzed reactions of (±)-**4a-j** 39
3. Determination of the absolute stereochemistry 65
4. Procedures for the synthesis of starting materials 66
5. Procedures for the product derivatization 90
6. Reaction optimisation 104
6.1. Screening of reaction additives 104
6.2. Variation of additive equivalents 105
6.3. Variation of base 105
7. Optimisation of Rh-catalyzed 1,4-addition to *E*-**3d** 106
8. Procedures for the synthesis of starting materials used in mechanistic studies 107
9. Mechanistic studies 115
9.1. Reactions of enantiopure substrate (*S*)-**1** 115
9.2. (*S*)- and (*R*)-ligands result in opposite absolute stereochemistry 117
9.3. Reaction monitoring 118
10. NMR spectra 120
10.1. Spectra of the starting materials 120
10.2. Spectra of the coupling products 153
10.3. Spectra of the derivatization products 207
10.4. Spectra of the alternative starting materials 219
11. SFC traces 227
12. References 255

1. General Methods

All reactions were carried out in flame-dried glassware, in anhydrous solvents with continuous magnetic stirring under an inert argon atmosphere. Heating was performed using DrySyn heating blocks.

Nuclear magnetic resonance (NMR) spectroscopy measurements were carried out at room temperature. ^{1}H NMR, ^{13}C NMR, ^{19}F NMR, COSY, HSQC, HMBC and NOESY experiments were carried out using Bruker AVN-400 (400/100 MHz), DQX-400 (400/100 MHz) or AVC-500 (500/125 MHz) spectrometers. Chemical shifts (δ) are reported in ppm relative to the residual solvent peak with corresponding coupling constants (*J*) in Hertz (Hz) and multiplicities (s: singlet, d: doublet, t: triplet, q: quartet, m: multiplet and combinations of these). Assignment follows HSQC, COSY, HMBC spectra, chemical shift and coupling constant analysis.

Optical rotations ($[\alpha]_{20}^{D}$) were recorded using a Perkin Elmer-241 Polarimeter. Concentrations (*c*) are reported in g/100 mL.

Infrared (IR, neat, thin film) spectroscopy was carried out on a Bruker Tensor 27 FT–IR spectrometer with in internal calibration range of 4000 – 600 cm^{-1}. Only characteristic bands are

Chiral SFC (supercritical fluid chromatography) separations were conducted on a Waters Acquity UPC2 system using Waters Empower software. Chiralpak® columns (150×3 mm, particle size 3 μm) were used as specified in the text. Solvents used were of HPLC grade (Fisher Scientific, Sigma Aldrich or Rathburn).

Chiral GC (gas chromatography) separations were conducted on an Agilent Technologies 7820A system using Hydrodex β-3P (25 m, 0.25 mm ID) column.

Commercially available reagents were purchased from Sigma Aldrich, Alfa Aesar, Acros Organics, Flurochem and Strem Chemicals and unless otherwise stated were used without further purification. $[Rh(cod)OH]_2$ was bought from Sigma Aldrich and Strem Chemicals.

All boronic acids were used without additional purification. Dry solvents were collected fresh from an mBraun SPS–800 solvent purification system after having passed through anhydrous alumina columns. Deuterated solvents were purchased from Sigma Aldrich.

Medium pressure chromatography was performed on a Combiflash Next Gen 100 system.

2. Procedures for the rhodium-catalyzed reactions

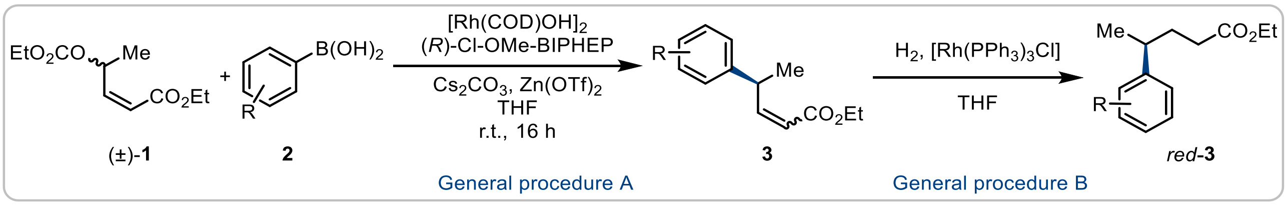

2.1. General procedure A (Rhodium-catalyzed arylation)

All reactions were carried out under an inert argon atmosphere using standard Schlenk techniques with all reagents weighed open to air.

$[Rh(cod)OH]_2$ (20.5 mg, 0.045 mmol, 2.5 mol%) and (*R*)-Cl-MeO-BIPHEP (70.4 mg, 0.108 mmol, 6.0 mol%) were added to 25 mL flask containing a stirring bar, and dissolved in dry THF (18.0 mL) under an argon atmosphere at ambient temperature (23 °C). This solution was stirred for 5 minutes and used for four asymmetric reactions on a 0.4 mmol scale).

Boronic acid (0.80 mmol, 2.0 equiv.), Cs_2CO_3 (130.3 mg, 0.40 mmol, 1.0 equiv.) and $Zn(OTf)_2$ (29.1 mg, 0.08 mmol, 0.2 equiv.) were added to a 7 mL vial containing a stirring bar. To this vial a stock solution of the rhodium hydroxy complex (4.0 mL) was added *via* syringe under an argon atmosphere. The allylic carbonate (0.40 mmol, 1.0 equiv.) was added *via* microsyringe and the reaction mixture was stirred at ambient temperature (23 °C).

The mixture was diluted with hexane (4.0 mL) and filtered through a plug of silica. The crude was loaded onto Chem Tube-Hydromatrix and flash column chromatography was performed to afford the desired products.

2.2. General procedure B (Hydrogenation)

Hydrogen (~1 atm, from a balloon) was bubbled through a solution of $[RhCl(PPh_3)_3]$ (37.1 mg, 0.040 mmol, 0.10 equiv.) in THF (0.70 mL) for 5 minutes. A mixture of Z- and E-products obtained from the rhodium arylation reaction (General procedure A) dissolved in THF (0.30 mL) was then added *via* syringe to the catalyst solution. Hydrogen (~1 atm, from a balloon) was bubbled through the reaction mixture for a further 5 minutes. The reaction mixture was equipped with a hydrogen balloon and stirred at ambient temperature (23 °C) for 16 h.

Then, the mixture was diluted with hexane (4.0 ml) and filtered through a plug of silica. The crude was loaded onto Chem Tube-Hydromatrix and flash column chromatography was performed to afford the desired product.

2.3. Rh-catalyzed reactions of (±)-**1**

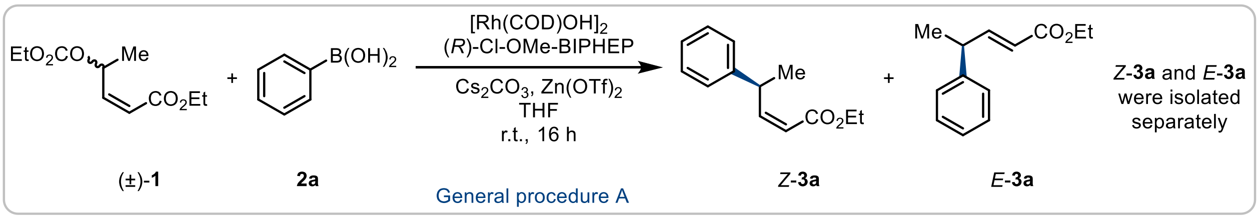

Z-**3a** and *E*-**3a** were prepared using General procedure A with phenylboronic acid. Crude mixture (*Z*:*E*=4.0:1) was purified by automated medium-pressure chromatography (E_2tO/hexane = 0/100 to 15/85) to afford (+)-ethyl (*S*,*Z*)-4-phenylpent-2-enoate *Z*-**3a** (61.3 mg, 0.30 mmol, 75%) as a colourless oil and (–)-ethyl (*S*,*E*)-4-phenylpent-2-enoate *E*-**3a** (15.5 mg, 0.08 mmol, 19%) as a colourless oil. SFC analysis showed an enantiomeric excess of 98% of *Z*-**3a** and 99% of *E*-**3a**.

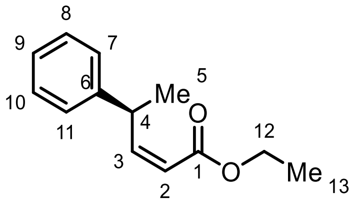

(+)-Ethyl (*S*,*Z*)-4-phenylpent-2-enoate *Z*-3a

^{1}H NMR (400 MHz, $CDCl_3$) δ 7.22 (m, 4H, C(Ar)-H x4), 7.13 (m, 1H, C(9)-H), 6.17 (dd, *J* = 11.4, 10.3 Hz, 1H, C(3)-H), 5.65 (dd, *J* = 11.5, 1.0 Hz, 1H, C(2)-H), 4.83 (dq, *J* = 10.4, 6.9, 1.0 Hz, 1H, C(4)-H), 4.12 (q, *J* = 7.0 Hz, 2H, C(12)-H_2), 1.32 (d, *J* = 6.9 Hz, 3H, C(5)-H_3), 1.22 (t, *J* = 7.1 Hz, 3H, C(13)-H_3).

^{13}C NMR (101 MHz, $CDCl_3$) δ 166.3 (C(1)), 153.7 (C(3)), 144.6 (C(6)), 128.6 (C(Ar) x2), 127.1 (C(Ar) x2), 126.4 (C(2)), 117.9 (C(9)), 60.0 (C(12)), 37.7 (C(4)), 20.9 (C(5)), 14.3 (C(13)).

IR (CH_3Cl film): 3027 (w), 2980 (w), 1716 (m), 1614 (w), 1414 (w), 1184 (s), 1030 (w), 830 (w), 754 (s), 700 (m), 668 (w) cm^{-1}.

HRMS (ESI): m/z calculated for $C_{13}H_{16}O_2Na^+$ $[M+Na]^+$ 227.1043 found 227.1044.

SFC: Chiralpak® IG, 1500 psi, 30 °C; flow: 1.0 mL/min; 1% to 30% MeOH over 5 min, 98.8:1.2 e.r. (minor enantiomer t_R = 1.46 min, major enantiomer t_R = 1.30 min).

$\boldsymbol{\alpha_D^{25}}$ = +326.2 (c = 1.0, $CHCl_3$).

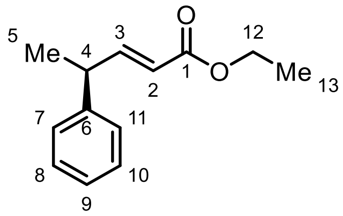

(–)-Ethyl (*S*,*E*)-4-phenylpent-2-enoate *E*-3a

^{1}H NMR (400 MHz, $CDCl_3$) δ 7.32 (m, 2H, C(Ar)-H x2), 7.22 (m, 3H, C(Ar)-H, x3), 7.12 (ddd, *J* = 15.7, 6.7 Hz, 1H, C(3)-H), 5.81 (dd, *J* = 15.7, 1.5Hz, 1H, C(2)-H), 4.18 (q, *J* = 7.1 Hz, 2H, C(12)-H_2), 3.62 (pd, *J* = 7.0, 1.6 Hz, 1H, C(4)-H), 1.44 (d, *J* = 7.0 Hz, 3H, C(5)-H_3), 1.28 (t, *J* = 7.1 Hz, 3H, C(13)-H_3).

^{13}C NMR (101 MHz, $CDCl_3$) δ 166.8 (C(1)), 152.6 (C(3)), 143.4 (C(6)), 128.7 (C(Ar) x2), 127.4 (C(Ar) x2), 126.7 (C(9)), 120.2 (C(2)), 60.3 (C(12)), 42.1 (C(4)), 20.2 (C(5)), 14.3 (C(13)).

IR (CH_3Cl film): 2952 (w), 1722 (m), 1653 (m), 1437 (m), 1281 (m), 1173 (m), 1018 (m), 909 (m), 762 (m), 729 (m), 699 (s) cm^{-1}.

HRMS (ESI): m/z calculated for $C_{13}H_{17}O_2^+$ $[M+H]^+$ 205.1223 found 205.1225.

SFC: Chiralpak® IG, 1500 psi, 30 °C; flow: 1.0 mL/min; 1% to 30% MeOH over 5 min, 99.5:0.5 e.r. (minor enantiomer t_R = 1.85 min, major enantiomer t_R = 1.68 min).

α_D^{25} = –14.8 (c = 1.0, $CHCl_3$).

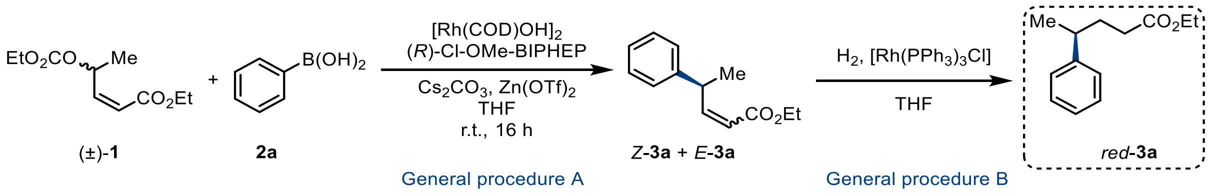

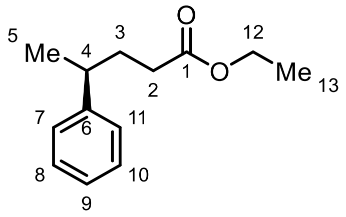

(+)-Ethyl (*S*)-4-phenylpentanoate *red*-3a

(+)-Ethyl (*S*)-4-phenylpentanoate *red*-**3a** was prepared using General procedure A with phenylboronic acid **2a**, followed by reduction of the resulting crude mixture of products (Z:E=4.0:1) using General procedure B. Purification by automated medium-pressure chromatography (E_2tO/hexane = 0/100 to 15/85) afforded (+)-ethyl (*S*)-4-phenylpentanoate *red*-**3a** (77.6 mg, 0.38 mmol, 94%) as a colourless oil. SFC analysis showed an enantiomeric excess of 98%.

^{1}H NMR (400 MHz, $CDCl_3$) δ 7.30 (td, *J* = 7.0, 1.3 Hz, 2H, C(Ar)-H x2), 7.19 (td, *J* = 7.0, 1.5 Hz, 3H, C(Ar)-H x3), 4.09 (qd, *J* = 7.1, 1.0 Hz, 2H, C(12)-H_2), 2.72 (h, *J* = 7.0 Hz, 1H. C(4)-H), 2.18 (m, 2H, C(2)-H_2), 1.92 (m, 2H, C(3)-H_2), 1.28 (d, *J* = 7.0 Hz, 3H, C(5)-H_3), 1.23 (t, *J* = 7.1 Hz, 3H, C(13)-H_3).

^{13}C NMR (101 MHz, $CDCl_3$) δ 173.7 (C(1)), 146.3 (C(6)), 128.5 (C(Ar) x2), 127.0 (C(Ar) x2), 126.2 (C(Ar)), 60.2 (C(12)), 39.5 (C(4)), 33.3 (C(2)), 32.6 (C(3)), 22.2 (C(4)), 14.2 (C(13)).

IR (CH_3Cl film): 2963 (w), 1733 (s), 1453 (w), 1375 (w), 1216 (m), 1163 (m), 1027 (w), 754 (s), 701 (s), 668 (w) cm^{-1}.

HRMS (ESI): m/z calculated for $C_{13}H_{18}O_2Na^+$ $[M+Na]^+$ 229.1199 found 229.1200.

SFC: Chiralpak® IG, 1500 psi, 30 °C; flow: 1.0 mL/min; 1% to 30% MeOH over 5 min, 99:1 e.r. (minor enantiomer t_R = 1.64 min, major enantiomer t_R = 1.52 min

α_D^{25} = +19.4 (c = 1.0, $CHCl_3$).

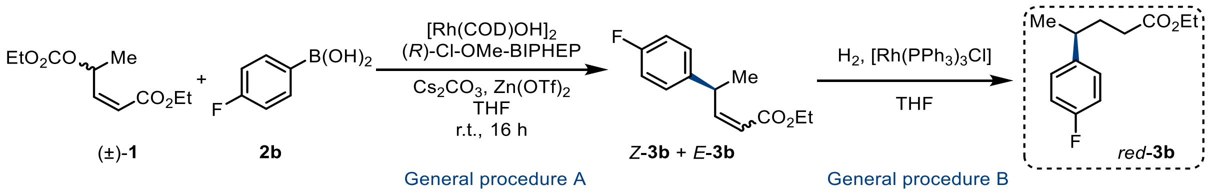

(+)-Ethyl (*S*)-4-(4-fluorophenyl)pentanoate *red*-3b

(+)-Ethyl (*S*)-4-(4-fluorophenyl)pentanoate *red*-**3b** was prepared using General procedure A with 4-fluorophenyl boronic acid **2b**, followed by reduction of the resulting crude mixture of products (Z:E=4.4:1) using General procedure B. Purification by automated medium-pressure chromatography (E_2tO/hexane = 0/100 to 20/80) afforded (+)-ethyl (*S*)-4-(4-fluorophenyl)pentanoate *red*-**3b** (80.7 mg, 0.36 mmol, 90%) as a colourless oil. SFC analysis showed an enantiomeric excess of 98%.

^{1}H NMR (400 MHz, $CDzCl_3$) δ 7.12 (m, 2H, C(Ar)-H x2), 6.97 (m, 2H, C(Ar)-H x2), 4.08 (q, *J* = 7.1 Hz, 2H, C(12)-H_2), 2.71 (dp, *J* = 8.9, 6.8 Hz, 1H, C(4)-H), 2.16 (m, 2H, C(2)-H_2), 1.88 (m, 2H, C(3)-H_2), 1.23 (m, 6H, C(5)-H_3 and C(13)-H_3).

^{13}C NMR (101 MHz, $CDCl_3$) δ 173.5 (C(1)), 161.4 (d, *J* = 243.6 Hz, C(9)), 141.9 (d, *J* = 3.1 Hz, C(6)), 128.3 (d, *J* = 7.8 Hz, C(7) and C(11)), 115.2 (d, *J* = 21.0 Hz, C(8) and C(10)), 60.3 (C(12)), 38.7 (C(4)), 33.3 (C(3)), 32.5 (C(2)), 22.3 (C(5)), 14.2 (C(13)).

^{19}F (13C)NMR (376 MHz, $CDCl_3$) δ −117.3.

IR (CH_3Cl film): 2980 (w), 1733 (m), 1510 (s), 1377 (m), 1223 (s), 1159 (s), 1033 (w), 835 (m), 757 (s), 668 (w) cm^{-1}.

HRMS (ESI): m/z calculated for $C_{13}H_{18}O_2F^+$ $[M+H]^+$ 225.1285 found 225.1286.

SFC: Chiralpak® IG, 1500 psi, 30 °C; flow: 1.0 mL/min; 1% to 30% MeOH over 5 min, 99:1 e.r. (minor enantiomer t_R = 1.54 min, major enantiomer t_R = 1.31 min).

α_D^{25} = +19.9 (c = 1.0, $CHCl_3$).

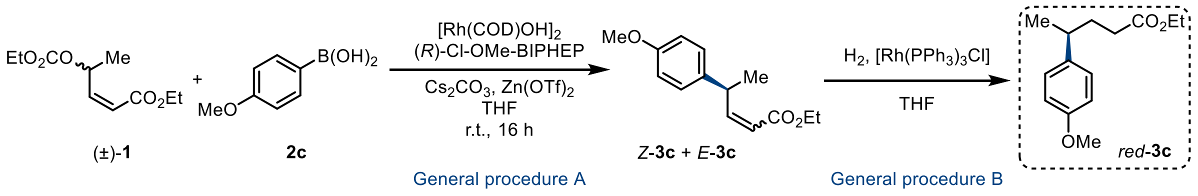

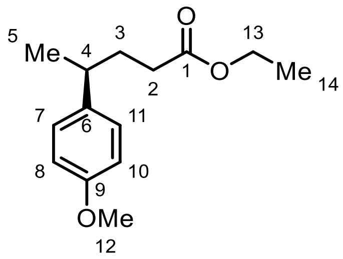

(+)-Ethyl (*S*)-4-(4-methoxyphenyl)pentanoate *red*-3c

(+)-Ethyl (*S*)-4-(4-methoxyphenyl)pentanoate *red*-**3c** was prepared using General procedure A with 4-methoxyphenylboronic acid **2c**, followed by reduction of the resulting crude mixture of products (Z:E=5.3:1) using General procedure B. Purification by automated medium-pressure chromatography (E_2tO/hexane = 0/100 to 15/85) afforded (+)-ethyl (*S*)-4-(4-methoxyphenyl)pentanoate *red*-**3c** (87.9 mg, 0.37 mmol, 93%) as a colourless oil. SFC analysis showed an enantiomeric excess of 97%.

^{1}H NMR (400 MHz, $CDCl_3$) δ 7.09 (d, *J* = 8.6 Hz, 2H, C(Ar)-H x2), 6.84 (d, *J* = 8.6 Hz, 2H, C(Ar)-H x2), 4.08 (q, *J* = 7.1 Hz, 2H, C(13)-H_2), 3.79 (s, 3H, C(12)-H_3), 2.67 (h, *J* = 6.8 Hz, 1H, C(4)-H), 2.18 (m, 2H, C(2)-H_2), 1.87 (m, 2H, C(3)-H_2), 1.23 (m, 6H, C(5)-H_3 and C(14)-H_3).

^{13}C NMR (101 MHz, $CDCl_3$) δ 173.7 (C(1)), 158.0 (C(9)), 138.4 (C(6)), 127.9 (C(7) and C(11)), 113.8 (C(8) and C(10)), 60.2 (C(13)), 55.2 (C(12)), 38.6 (C(4)), 33.4 (C(2)), 32.6 (C(3)), 22.3 (C(5)), 14.2 (C(14)).

IR (CH_3Cl film): 2981 (w), 1733 (s), 1513 (s), 1377 (m), 1247 (s), 1178 (m), 1035 (m), 831 (m), 755 (m), 668 (w) cm^{-1}.

HRMS (ESI): m/z calculated for $C_{14}H_{20}O_3Na^+$ $[M+Na]^+$ 259.1305 found 259.1304.

SFC: Chiralpak® IG, 1500 psi, 30 °C; flow: 1.0 mL/min; 1% to 30% MeOH over 5 min, 98.6:1.4 e.r. (minor enantiomer t_R = 2.42 min, major enantiomer t_R = 2.01 min).

α_D^{25} = +21.6 (c = 1.0, $CHCl_3$).

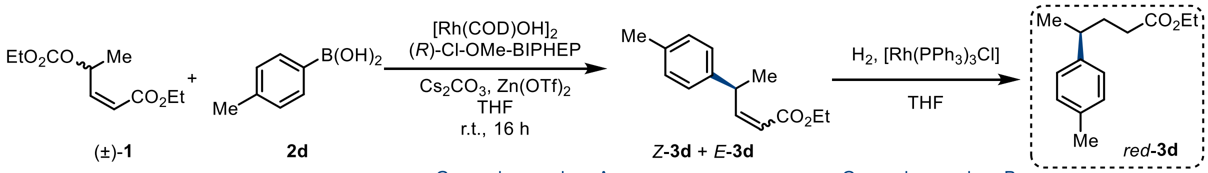

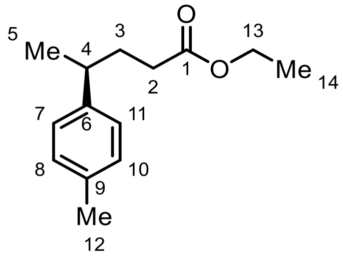

(+)-Ethyl (S)-4-(*p*-tolyl)pentanoate *red*-**3d**

(+)-Ethyl (S)-4-(*p*-tolyl)pentanoate *red*-**3d** was prepared using General procedure A with *p*-tolylboronic acid **2d**, followed by reduction of the resulting crude mixture of products (Z:E=4.4:1) using General procedure B. Purification by automated medium-pressure chromatography (E_2tO/hexane = 0/100 to 15/85) afforded (+)-ethyl (*S*)-4-(*p*-tolyl)pentanoate *red*-**3d** (84.6 mg, 0.38 mmol, 96%) as a colourless oil. SFC analysis showed an enantiomeric excess of 97%. Characterisation data match literature reports.[1]

^{1}H NMR (400 MHz, $CDCl_3$) δ 7.12 (dd, *J* = 8.0, 2.3 Hz, 2H, C(Ar)-H x2), 7.08 (d, *J* = 8.1, 2.3 Hz, 2H, C(Ar)-H x2), 4.10 (q, *J* = 7.1 Hz, 2H, C(13)-H_2), 2.69 (dp, *J* = 8.7, 6.8 Hz, 1H, C(4)-H), 2.33 (s, 3H, C(12)-H_3), 2.19 (m, 2H, C(2)-H_2), 1.90 (m, 2H, C(3)-H_2), 1.27 (d, *J* = 7.0 Hz, 3H, C(5)-H_3), 1.23 (t, *J* = 7.1 Hz, 3H, C(14)-H_3).

^{13}C NMR (101 MHz, $CDCl_3$) δ 173.7 (C(1)), 143.3 (C(6)), 135.6 (C(9)), 129.1 (C(Ar) x2), 126.9 (C(Ar) x2), 60.2 (C(13)), 39.0 (C(4)), 33.3 (C(2)), 32.6 (C(3)), 22.3 (C(12)), 21.0 (C(5)), 14.2 (C(14)).

IR (CH_3Cl film): 3021 (w), 1728 (m), 1515 (w), 1217 (m), 1033 (w), 819 (w), 755 (s), 668 (w) cm^{-1}.

HRMS (ESI): m/z calculated for $C_{14}H_{20}O_2Na^+$ $[M+Na]^+$ 243.1356 found 243.1357.

SFC: Chiralpak® IG, 1500 psi, 30 °C; flow: 1.0 mL/min; 1% to 30% MeOH over 5 min, 98.6:1.4 e.r. (minor enantiomer t_R = 1.83 min, major enantiomer t_R = 1.64 min).

α_D^{25} = +18.4 (c = 1.0, $CHCl_3$) (Lit[1].: α_D^{25} = +13.9 (c = 4.9, $CHCl_3$)).

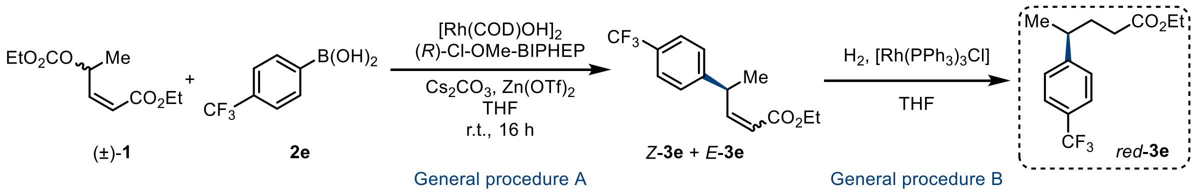

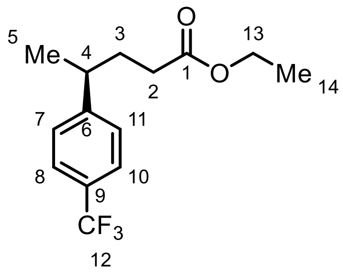

(+)-Ethyl (*S*)-4-(4-(trifluoromethyl)phenyl)pentanoate *red*-**3e**

(+)-Ethyl (*S*)-4-(4-(trifluoromethyl)phenyl)pentanoate *red*-**3e** was prepared using General procedure A with 4-(trifluoromethyl boronic acid **2e**, followed by reduction of the resulting crude mixture of products (Z:E=6.2:1) using General procedure B. Purification by automated medium-pressure chromatography (E_2tO/hexane = 0/100 to 20/80) afforded (+)-ethyl (*S*)-4-(4-(trifluoromethyl)pentanoate *red*-**3e** (85.6 mg, 0.31 mmol, 78%) as a colourless oil. SFC analysis showed an enantiomeric excess of 98%.

^{1}H NMR (400 MHz, $CDCl_3$) δ 7.55 (d, *J* = 8.0 Hz, 2H, C(8)-H and C(10)-H), 7.29 (d, *J* = 8.0 Hz, 2H, C(7)-H and C(11)-H), 4.08 (q, *J* = 7.1 Hz, 2H, C(13)-H_2), 2.80 (dp, *J* = 8.7, 6.9 Hz, 1H, C(4)-H), 2.17 (m, 2H, C(2)-H_2), 1.93 (m, 2H, C(3)-H_2), 1.28 (d, *J* = 7.0 Hz, 3H, C(5)-H_3), 1.22 (t, *J* = 7.1 Hz, 3H, C(14)-H_3).

^{13}C NMR (101 MHz, $CDCl_3$) δ 173.3 (C(1)), 150.5 (C(6)), 128.6 (q, *J* = 32.2 Hz, C(9)), 127.4 (C(7) and C(11)), 125.4 (q, *J* = 3.8 Hz, C(8) and C(10)), 124.3 (q, *J* = 271.8 Hz, C(12)), 60.3 (C(13)), 39.3 (C(4)), 33.0 (C(2)), 32.4 (C(3)), 21.9 (C(5)), 14.2 (C(14)).

^{19}F (13C)NMR (376 MHz, $CDCl_3$) δ −62.4.

IR (CH_3Cl film): 2980 (w), 1735 (s), 1619 (w), 1327 (s), 1164 (s), 1122 (s), 1069 (m), 1017 (w), 841 (m) cm^{-1}.

HRMS (ESI): m/z calculated for $C_{14}H_{18}O_2F_3^+$ $[M+H]^+$ 275.1253 found 275.1254.

SFC: Chiralpak® IG, 1500 psi, 30 °C; flow: 1.0 mL/min; 1% to 30% MeOH over 5 min, 98.8:1.2 e.r. (minor enantiomer t_R = 1.30 min, major enantiomer t_R = 1.08 min).

$\boldsymbol{\alpha_D^{25}}$ = +18.6 (c = 1.0, $CHCl_3$).

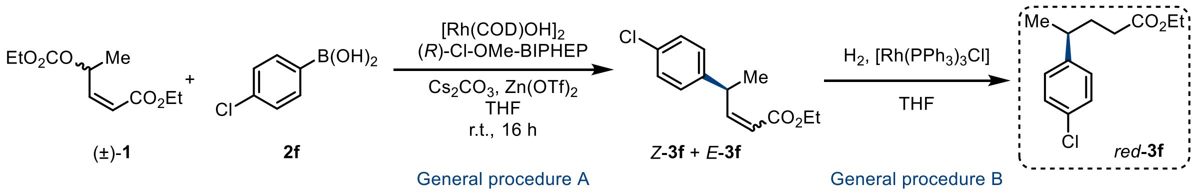

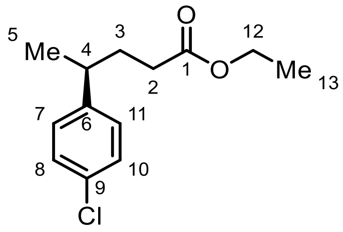

(+)-Ethyl (*S*)-4-(4-chlorophenyl)pentanoate *red*-3f

(+)-Ethyl (*S*)-4-(4-chlorophenyl)pentanoate *red*-**3f** was prepared using General procedure A with (4-chlorophenyl)boronic acid **2f**, followed by reduction of the resulting crude mixture of products (Z:E=7.3:1) using General procedure B. Purification by automated medium-pressure chromatography (E_2tO/hexane = 0/100 to 15/85) afforded (+)-ethyl (*S*)-4-(4-chlorophenyl)pentanoate *red*-**3f** (57.8 mg, 0.24 mmol, 60%) as a colourless oil. SFC analysis showed an enantiomeric excess of 98%.

^{1}H NMR (400 MHz, $CDCl_3$) δ 7.26 (d, *J* = 8.5 Hz, 2H, C(Ar)-H x2), 7.11 (d, *J* = 8.4 Hz, 2H, C(Ar)-H x2), 4.08 (q, *J* = 7.2 Hz, 2H, C(12)-H_2), 2.70 (dp, *J* = 8.9, 6.8 Hz, 1H, C(4)-H), 2.16 (m, 2H, C(2)-H_2), 1.88 (m, 2H, C(3)-H_2), 1.23 (m, 6H, C(5)-H_3 and C(13)-H_3).

^{13}C NMR (101 MHz, $CDCl_3$) δ 173.5 (C(1)), 144.8 (C(6)), 131.8 (C(9)), 128.6 (C(Ar) x2), 128.4 (C(Ar) x2), 60.3 (C(12)), 38.9 (C(4)), 33.1 (C(2)), 32.5 (C(3)), 22.1 (C(5)), 14.2 (C(13)).

IR (CH_3Cl film): 2970 (w), 1733 (s), 1459 (w), 1375 (w), 1215 (m), 1063 (m), 1033 (w), 754 (s), 698 (m), 668 (w) cm^{-1}.

HRMS (ESI): m/z calculated for $C_{13}H_{18}O_2Cl^+$ $[M+H]^+$ 241.0990 found 241.0991.

SFC: Chiralpak® IG, 1500 psi, 30 °C; flow: 1.0 mL/min; 1% to 30% MeOH over 5 min, 98.9:1.1 e.r. (minor enantiomer t_R = 2.17 min, major enantiomer t_R = 1.75 min).

α_D^{25} = +24.1 (c = 1.0, $CHCl_3$).

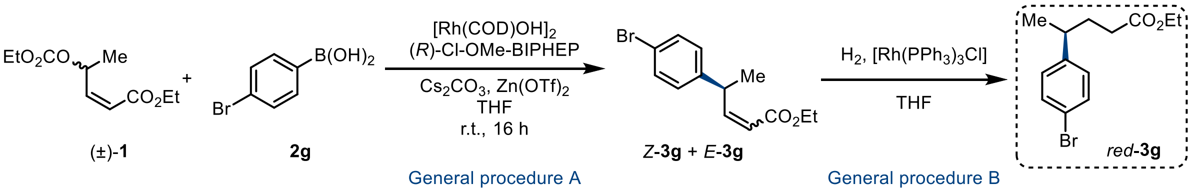

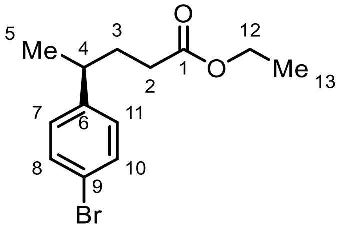

(+)-Ethyl (*S*)-4-(4-bromophenyl)pentanoate *red*-3g

(+)-Ethyl (*S*)-4-(4-bromophenyl)pentanoate *red*-**3g** was prepared using General procedure A with (4-bromophenyl)boronic acid **2g**, followed by reduction of the resulting crude mixture of products (*Z*:*E*=4.5:1) using General procedure B. Purification by automated medium-pressure chromatography (Et_2O/hexane = 0/100 to 15/85) afforded (+)-ethyl (*S*)-4-(4-bromophenyl)pentanoate *red*-**3g** (84.4 mg, 0.30 mmol, 74%) as a colourless oil. SFC analysis showed an enantiomeric excess of 97%.

^{1}H NMR (400 MHz, $CDCl_3$) δ 7.41 (d, *J* = 8.4 Hz, 1H, C(8)-H and C(10)-H), 7.05 (d, *J* = 8.4 Hz, 1H, C(7)-H and C(11)-H), 4.08 (q, *J* = 7.2 Hz, 2H, C(12)-H_2), 2.69 (dp, *J* = 8.9, 6.9 Hz, 1H, C(4)-H), 2.16 (m, 2H, C(2)-H_2), 1.88 (m, 2H, C(3)-H_2), 1.23 (m, 6H, C(5)-H_3 and C(13)-H_3).

^{13}C NMR (101 MHz, $CDCl_3$) δ 173.4 (C(1)), 145.3 (C(6)), 131.5 (C(7) and C(11)), 128.8 (C(8) and C(10)), 119.8 (C(9)), 60.3 (C(12)), 38.9 (C(4)), 33.1 (C(2)), 32.4 (C(3)), 22.0 (C(5)), 14.2 (C(13)).

IR (CH_3Cl film): 2963 (w), 1732 (s), 1490 (w), 1215 (m), 1010 (w), 824 (w), 755 (s), 668 (w) cm^{-1}.

HRMS (ESI): m/z calculated for $C_{13}H_{18}O_2Br^+$ $[M+H]^+$ 285.0485 found 285.0485.

SFC: Chiralpak® IG, 1500 psi, 30 °C; flow: 1.0 mL/min; 1% to 30% MeOH over 5 min, 98.4:1.6 e.r. (minor enantiomer t_R = 2.62 min, major enantiomer t_R = 2.04 min).

α_D^{25} = +23.0 (c = 1.0, $CHCl_3$).

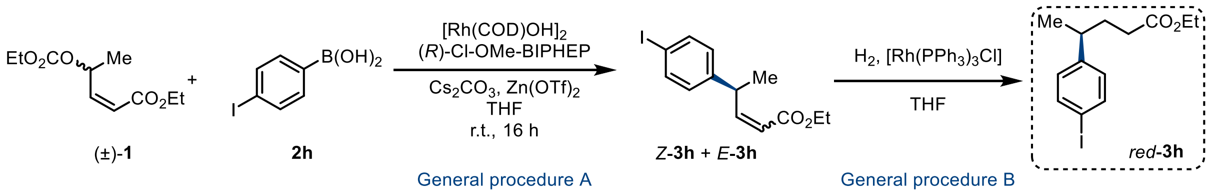

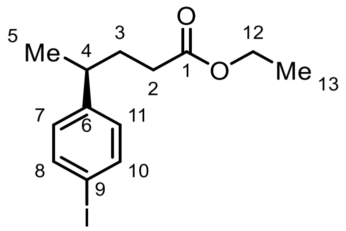

(+)-Ethyl (*S*)-4-(4-iodophenyl)pentanoate *red*-3h

(+)-Ethyl (*S*)-4-(4-iodophenyl)pentanoate ***red*-3h** was prepared using General procedure A with (4-iodophenyl)boronic acid **2h**, followed by reduction of the resulting crude mixture of products (*Z*:*E*=6.4:1) using General procedure B. Purification by automated medium-pressure chromatography (E_2tO/hexane = 0/100 to 15/85) afforded (+)-ethyl (*S*)-4-(4-iodophenyl)pentanoate ***red*-3h** (75.7 mg, 0.23 mmol, 57%) as a colourless oil. SFC analysis showed an enantiomeric excess of 98%.

^{1}H NMR (400 MHz, $CDCl_3$) δ 7.61 (d, *J* = 8.3 Hz, 2H, C(8)-H and C(10)-H), 6.93 (d, *J* = 8.3 Hz, 2H, C(7)-H and C(11)-H), 4.08 (q, *J* = 7.1 Hz, 2H, C(12)-H_2), 2.67 (dp, *J* = 9.0, 6.8 Hz, 1H, C(4)-H), 2.17 (m, 2H, C(2)-H_2), 1.88 (m, 2H, C(3)-H_2), 1.23 (m, 6H, C(5)-H_3 and C(13)-H_3).

^{13}C NMR (101 MHz, $CDCl_3$) δ 173.5 (C(1)), 146.0 (C(6)), 137.5 (C(8) and C(10)), 129.2 (C(7) and C(11)), 91.2 (C(9)), 60.3 (C(12)), 39.0 (C(4)), 33.0 (C(2)), 32.4 (C(3)), 22.0 (C(5)), 14.2 (C(13)).

IR (CH_3Cl film): 2962 (w), 1732 (s), 1486 (w), 1375 (m), 1162 (m), 1031 (m), 1006 (m), 821 (m), 756 (s), 668 (w) cm^{-1}.

HRMS (ESI): m/z calculated for $C_{13}H_{18}O_2I^+$ $[M+H]^+$ 333.0346 found 333.0346.

SFC: Chiralpak® IF, 1500 psi, 30 °C; flow: 1.0 mL/min; 1% to 30% MeOH over 5 min, 98.8:1.2 e.r. (minor enantiomer t_R = 2.66 min, major enantiomer t_R = 2.21 min).

α_D^{25} = +22.8 (c = 1.0, $CHCl_3$).

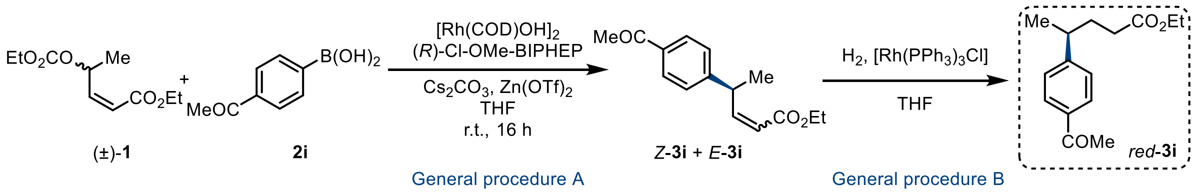

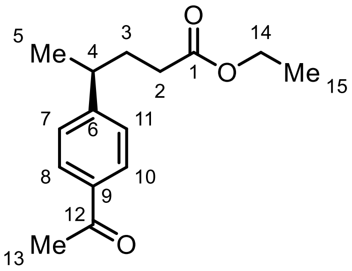

(+)-Ethyl (*S*)-4-(4-acetylphenyl)pentanoate *red*-3i

(+)-Ethyl (*S*)-4-(4-acetylphenyl)pentanoate *red*-**3i** was prepared using General procedure A with (4-acetylphenyl)boronic acid **2i**, followed by reduction of the resulting crude mixture of products (*Z*:*E*=5.4:1) using General procedure B. Purification by automated medium-pressure chromatography (E_2tO/hexane = 0/100 to 40/60) afforded (+)-ethyl (*S*)-4-(4-acetylphenyl)pentanoate *red*-**3i** (81.4 mg, 0.33 mmol, 82%) as a colourless oil. SFC analysis showed an enantiomeric excess of 98%.

^{1}H NMR (400 MHz, $CDCl_3$) δ 7.90 (d, *J* = 8.2 Hz, 1H, C(7)-H and C(11)-H), 7.27 (d, *J* = 8.1 Hz, 1H, 1H, C(8)-H and C(10)-H), 4.09 (q, *J* = 7.2 Hz, 2H, C(14)-H_2), 2.80 (dp, *J* = 8.7, 6.8 Hz, 1H, C(4)-H), 2.58 (s, 3H, C(13)-H_3), 2.17 (m, 2H, C(2)-H_2), 1.94 (m, 2H, C(3)-H_2), 1.29 (d, *J* = 6.9 Hz, 3H, C(5)-H_3), 1.22 (t, *J* = 7.1 Hz, 3H, C(15)-H_3).

^{13}C NMR (101 MHz, $CDCl_3$) δ 197.8 (C(12)), 173.4 (C(1)), 152.1 (C(6)), 135.5 (C(9)), 128.7 (C(8) and C(10)), 127.3 (C(7) and C(11)), 60.3 (C(14)), 39.5 (C(4)), 32.9 (C(2)), 32.4 (C(3)), 26.5 (C(13)), 21.8 (C(5)), 14.2 (C(15)).

IR (CH_3Cl film): 2965 (w), 1733 (s), 1683 (s), 1607 (m), 1458 (w), 1360 (m), 1267 (s), 1183 (m), 1032 (w), 957 (m), 835 (m) cm^{-1}.

HRMS (ESI): m/z calculated for $C_{15}H_{21}O_3^+$ $[M+H]^+$ 249.1485 found 249.1486.

SFC: Chiralpak® IG, 1500 psi, 30 °C; flow: 1.0 mL/min; 1% to 30% MeOH over 5 min, 98.8:1.2 e.r. (minor enantiomer t_R = 3.87 min, major enantiomer t_R = 3.31 min).

α_D^{25} = +27.6 (c = 1.0, $CHCl_3$).

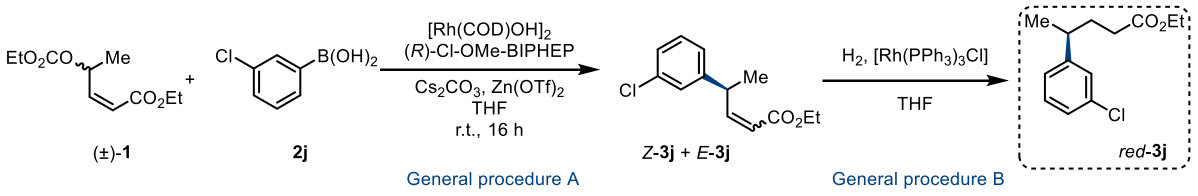

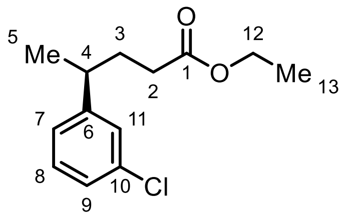

(+)-Ethyl (*S*)-4-(3-chlorophenyl)pentanoate *red*-3j

(+)-Ethyl (*S*)-4-(3-chlorophenyl)pentanoate *red*-**3j** was prepared using General procedure A with 3-chlorophenyl boronic acid **2j**, followed by reduction of the resulting crude mixture of products (*Z*:*E*=2.7:1) using General procedure B. Purification by automated medium-pressure chromatography (E_2tO/hexane = 0/100 to 15/85) afforded (+)-ethyl (*S*)-4-(3-chlorophenyl)pentanoate *red*-**3j** (84.7 mg, 0.35 mmol, 88%) as a colourless oil. SFC analysis showed an enantiomeric excess of 95%.

^{1}H NMR (400 MHz, $CDCl_3$) δ 7.22 (dd, *J* = 8.6, 7.4 Hz, 1H, C(8)-H), 7.17 (m, 2H, C(Ar)-H x2), 7.05 (dt, *J* = 7.5, 1.5 Hz, 1H, C(7)-H), 4.09 (q, *J* = 7.2 Hz, 2H, C(12)-H_2), 2.70 (dp, *J* = 8.8, 6.8 Hz, 1H, C(4)-H), 2.17 (m, 2H, C(2)-H_2), 1.90 (m, 2H, C(3)-H_2), 1.24 (m, 6H, C(5)-H_3 and C(13)-H_3).

^{13}C NMR (101 MHz, $CDCl_3$) δ 173.4 (C(1)), 148.5 (C(6)), 134.3 (C(10)), 129.7 (C(Ar)), 127.2 (C(Ar)), 126.4 (C(Ar)), 125.3 (C(Ar)), 60.3 (C(12)), 39.2 (C(4)), 33.0 (C(2)), 32.4 (C(3)), 21.9 (C(5)), 14.2 (C(13)).

IR (CH_3Cl film): 2970 (w), 1733 (s), 1459 (m), 1375 (m), 1215 (m), 1163 (m), 1033 (w), 754 (s), 698 (m), 668 (w) cm^{-1}.

HRMS (ESI): m/z calculated for $C_{13}H_{18}O_2Cl^+$ $[M+H]^+$ 241.0990 found 241.0991.

SFC: Chiralpak® ID, 1500 psi, 30 °C; flow: 1.0 mL/min; 1% to 30% MeOH over 5 min, 97.6:2.4 e.r. (minor enantiomer t_R = 1.29 min, major enantiomer t_R = 1.36 min).

α_D^{25} = +21.1 (c = 1.0, $CHCl_3$)

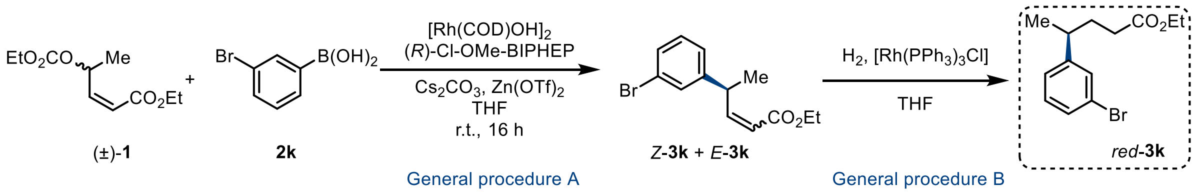

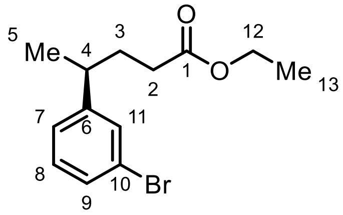

(+)-Ethyl (*S*)-4-(3-bromophenyl)pentanoate *red*-3k

(+)-Ethyl (*S*)-4-(3-bromophenyl)pentanoate *red*-**3k** was prepared using General procedure A with 3-bromophenyl boronic acid **2k**, followed by reduction of the resulting crude mixture of products (Z:E=4.9:1) using General procedure B. Purification by automated medium-pressure chromatography (E_2tO/hexane = 0/100 to 15/85) afforded (+)-ethyl (*S*)-4-(3-bromophenyl)pentanoate *red*-**3k** (111.4 mg, 0.39 mmol, 98%) as a colourless oil. SFC analysis showed an enantiomeric excess of 96%.

^{1}H NMR (400 MHz, $CDCl_3$) δ 7.32 (m, 2H, C(Ar)-H x2), 7.16 (t, *J* = 8.0 Hz, 1H, C(8)-H), 7.10 (dt, *J* = 7.7, 1.5 Hz, 1H, C(7)-H), 4.09 (q, *J* = 7.2 Hz, 2H, C(12)-H_2), 2.69 (dp, *J* = 8.7, 6.8 Hz, 1H, C(4)-H), 2.17 (m, 2H, C(2)-H_2), 1.89 (m, 2H, C(3)-H_2), 1.24 (m, 6H, C(5)-H_3 and C(13)-H_3).

^{13}C NMR (101 MHz, $CDCl_3$) δ 173.4 (C(1)), 148.8 (C(6)), 130.2 (C(Ar)), 130.1 (C(Ar)), 129.3 (C(Ar)), 125.8 (C(Ar)), 122.6 (C(10)), 60.3 (C(12)), 39.2 (C(4)), 33.0 (C(2)), 32.4 (C(3)), 22.0 (C(5)), 14.2 (C(13)).

IR (CH_3Cl film): 2969 (w), 1734 (s), 1491 (w), 1235 (m), 1215 (m), 1017 (w), 823 (w), 756 (s), 668 (w) cm^{-1}.

HRMS (ESI): m/z calculated for $C_{13}H_{18}O_2Br^+$ $[M+H]^+$ 287.0465 found 287.0464.

SFC: Chiralpak® IF, 1500 psi, 30 °C; flow: 1.0 mL/min; 1% to 30% MeOH over 5 min, 98.2:1.8 e.r. (minor enantiomer t_R = 1.64 min, major enantiomer t_R = 1.69 min).

α_D^{25} = +20.8 (c = 1.0, $CHCl_3$)

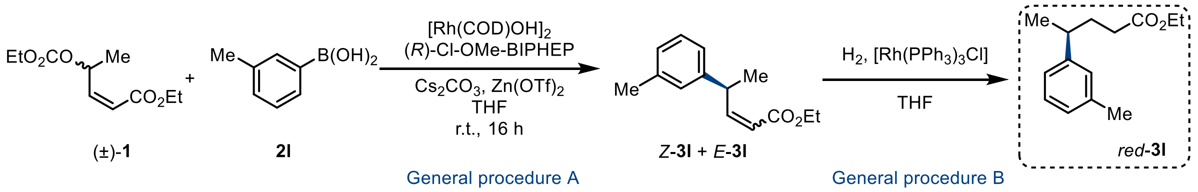

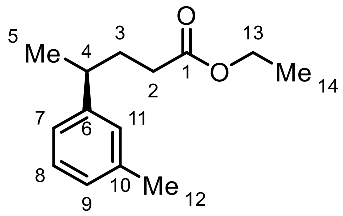

(+)-Ethyl (*S*)-4-(*m*-tolyl)pentanoate *red*-3l

(+)-Ethyl (*S*)-4-(*m*-tolyl)pentanoate *red*-**3l** was prepared using General procedure A with *m*-tolyl boronic acid **2l**, followed by reduction of the resulting crude mixture of products (*Z*:*E*=3.5:1) using General procedure B. Purification by automated medium-pressure chromatography (E_2tO/hexane = 0/100 to 15/85) afforded (+)-ethyl (*S*)-4-(*m*-tolyl)pentanoate *red*-**3l** (82.8 mg, 0.38 mmol, 94%) as a colourless oil. SFC analysis showed an enantiomeric excess of 96%.

^{1}H NMR (400 MHz, $CDCl_3$) δ 7.19 (td, *J* = 7.3, 1.1 Hz, 1H, C(Ar)-H), 7.00 (m, 3H, C(Ar)-H x3), 4.10 (q, *J* = 7.1 Hz, 2H, C(13)-H_2), 2.69 (dt, *J* = 8.5, 6.8 Hz, 1H, C(4)-H), 2.34 (s, 3H, C(12)-H_3), 2.20 (m, 2H, C(2)-H_2), 1.91 (m, 2H, C(3)-H_2), 1.27 (d, *J* = 6.9 Hz, 3H, C(5)-H_3), 1.24 (t, *J* = 7.1 Hz, 3H, C(14)-H_3).

^{13}C NMR (101 MHz, $CDCl_3$) δ 173.7 (C(1)), 146.3 (C(6)), 137.9 (C(10)), 128.3 (C(Ar)), 127.8 (C(Ar)), 126.9 (C(Ar)), 124.0 (C(Ar)), 60.2 (C(13)), 39.4 (C(4)), 33.2 (C(2)), 32.7 (C(3)), 22.2 (C(12)), 21.5 (C(5)), 14.2 (C(14)).

IR (CH_3Cl film): 2960 (w), 2928 (w), 1725 (s), 1458 (m), 1376 (m), 1181 (m), 1035 (w), 751 (s), 668 (m) cm^{-1}.

HRMS (ESI): m/z calculated for $C_{14}H_{21}O_2^+$ $[M+H]^+$ 221.1536 found 221.1538.

SFC: Chiralpak® IG, 1500 psi, 30 °C; flow: 1.0 mL/min; 1% to 30% MeOH over 5 min, 97.8:2.2 e.r. (minor enantiomer t_R = 1.42 min, major enantiomer t_R = 1.35 min).

α_D^{25} = +19.8 (c = 1.0, $CHCl_3$)

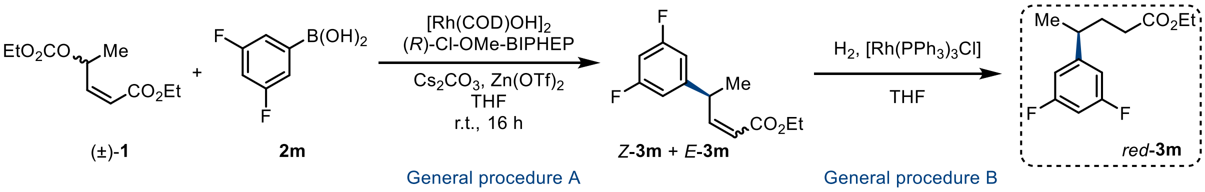

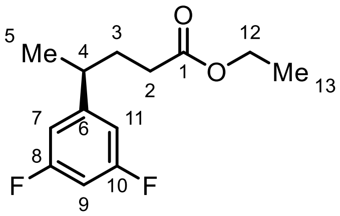

(+)-Ethyl (*S*)-4-(3,5-difluorophenyl)pentanoate *red*-3m

(+)-Ethyl (*S*)-4-(3,5-difluorophenyl)pentanoate ***red*-3m** was prepared using General procedure A with 3,5-difluorophenyl boronic acid **2m**, followed by reduction of the resulting crude mixture of products (*Z*:*E*=9.5:1) using General procedure B. Purification by automated medium-pressure chromatography (Et_2O/hexane = 0/100 to 20/80) afforded (+)-ethyl (*S*)-4-(3,5-difluorophenyl)pentanoate ***red*-3m** (80.4 mg, 0.33 mmol, 83%) as a colourless oil. SFC analysis showed an enantiomeric excess of 97%.

^{1}H NMR (400 MHz, $CDCl_3$) δ 6.69 (m, 2H, C(7)-H and C(11)-H), 6.62 (tt, *J* = 8.9, 2.3 Hz, 1H, C(9)-H), 4.09 (q, *J* = 7.1 Hz, 2H, C(12)-H_2), 2.72 (h, *J* = 8.5, 6.7 Hz, 1H, C(4)-H), 2.19 (m, 2H, C(2)-H_2), 1.88 (m, 2H, C(3)-H_2), 1.23 (m, 6H, C(5)-H_3 and C(13)-H_3).

^{13}C NMR (101 MHz, $CDCl_3$) δ 173.2 (C(1)), 163.13 (dd, *J* = 248.0, 12.9 Hz, C(8) and C(10)), 150.5 (t, *J* = 8.4 Hz, C(6)), 109.8 (d, *J* = 24.6Hz, C(7) and C(11)), 101.6 (t, *J* = 25.3 Hz, C(9)), 60.3 (C(12)), 39.3 (t, *J* = 2.0 Hz, C(4)), 32.8 (C(2)), 32.3 (C(3)), 21.7 (C(5)), 14.2 (C(13)).

^{19}F (13C)NMR (376 MHz, $CDCl_3$) δ −110.2.

IR (CH_3Cl film): 2936 (w), 2360 (w), 1725 (s), 1624 (s), 1597 (s), 1461 (s), 1375 (m), 1307 (w), 1263 (w), 1182 (m), 1138 (s), 1035 (w), 944 (s), 849 (s), 750 (s), 693 (m) cm^{-1}.

HRMS (GC EI MS): m/z calculated for $C_{13}H_{16}F_2O_2^+$ $[M]^+$ 242.11129 found 242.11268.

SFC: Chiralpak® IG, 1500 psi, 30 °C; flow: 1.0 mL/min; isocratic: 100% CO_2 for 3 min then gradient: 0% to 10% MeOH/CO_2 over 5 min, 98.6:1.4 e.r. (minor enantiomer t_R = 3.75 min, major enantiomer t_R = 4.20 min).

α_D^{25} = +25.0 (c = 1.0, $CHCl_3$)

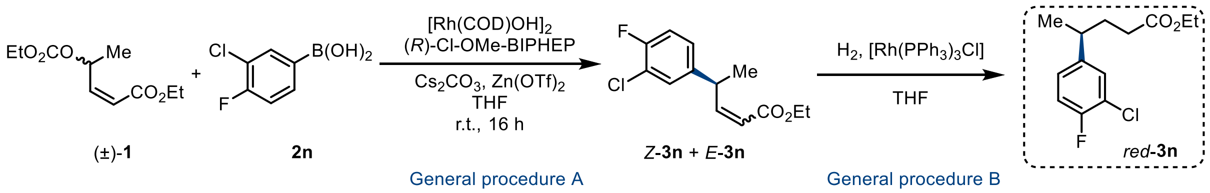

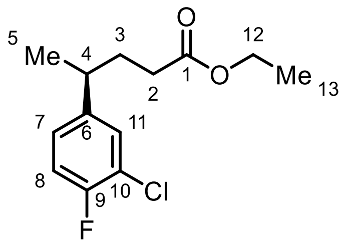

(+)-Ethyl (*S*)-4-(3-chloro-4-fluorophenyl)pentanoate *red*-3n

(+)-Ethyl (*S*)-4-(3-chloro-4-fluorophenyl)pentanoate *red*-**3n** was prepared using General procedure A with 3-chloro-4-fluorophenyl boronic acid **2n**, followed by reduction of the resulting crude mixture of products (Z:E=6.2:1) using General procedure B. Purification by automated medium-pressure chromatography (E_2tO/hexane = 0/100 to 20/80) afforded (+)-ethyl (*S*)-4-(3-chloro-4-fluorophenyl)pentanoate *red*-**3n** (88.0 mg, 0.34 mmol, 85%) as a colourless oil. SFC analysis showed an enantiomeric excess of 95%.

^{1}H NMR (400 MHz, $CDCl_3$) δ 7.20 (dd, *J* = 7.1, 2.0 Hz, 1H, C(8)-H), 7.05 (m, 2H, C(Ar)-H x2), 4.09 (q, *J* = 7.2 Hz, 2H, C(12)-H_2), 2.70 (dp, *J* = 8.8, 6.9 Hz, 1H, C(4)-H), 2.17 (m, 2H, C(2)-H_2), 1.87 (m, 2H, C(3)-H_2), 1.23 (t, *J* = 7.1 Hz, 6H, C(5)-H_3 and C(13)-H_3).

^{13}C NMR (101 MHz, $CDCl_3$) δ 173.3 (C(1)), 156.6 (d, *J* = 246.8 Hz, C(9)), 143.4 (d, *J* = 3.9 Hz, C(6)), 129.0 (C(11)), 126.6 (d, *J* = 6.8 Hz, C(7)), 120.7 (d, *J* = 17.7 Hz, C(10)), 116.4 (d, *J* = 20.7 Hz, C(8)), 60.3 (C(12)), 38.6 (C(4)), 33.1 (C(2)), 32.4 (C(3)), 22.0 (C(5)), 14.2 (C(13)).

^{19}F (13C)NMR (376 MHz, $CDCl_3$) δ −119.4.

IR (CH_3Cl film): 2962 (w), 2361 (w), 1732 (s), 1507 (s), 1460 (w), 1374 (w), 1249 (s), 1205 (m), 1165 (m), 1099 (w), 1061 (m), 1034 (m), 823 (w), 754 (w) cm^{-1}.

HRMS (ESI): m/z calculated for $C_{13}H_{17}ClFO_2^+$ $[M+H]^+$ 259.0896 found 259.0897.

SFC: Chiralpak® IG, 1500 psi, 30 °C; flow: 1.0 mL/min; 1% to 30% MeOH over 5 min, 97.6:2.4 e.r. (minor enantiomer t_R = 1.48 min, major enantiomer t_R = 1.43 min).

α_D^{25} = +24.7 (c = 1.0, $CHCl_3$)

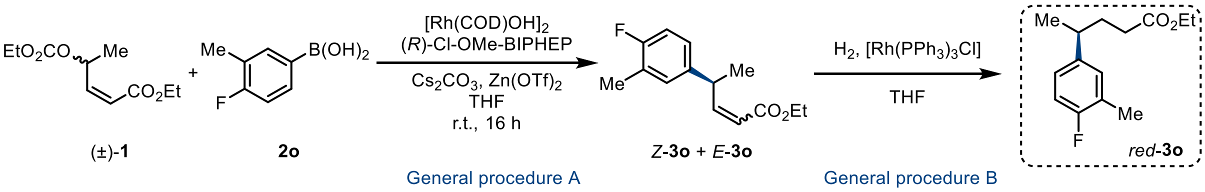

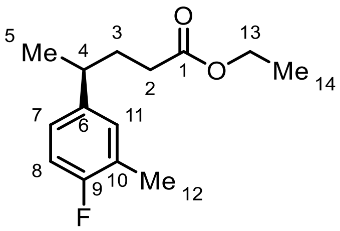

(+)-Ethyl (*S*)-4-(4-fluoro-3-methylphenyl)pentanoate *red*-**3o**

(+)-Ethyl (*S*)-4-(4-fluoro-3-methylphenyl)pentanoate *red*-**3o** was prepared using General procedure A with 4-fluoro-3-methylphenyl boronic acid **2o**, followed by reduction of the resulting crude mixture of products (Z:E=4.3:1) using General procedure B. Purification by automated medium-pressure chromatography (E_2tO/hexane = 0/100 to 20/80) afforded (+)-ethyl (*S*)-4-(4-fluoro-3-methylphenyl)pentanoate *red*-**3o** (87.7mg, 0.37 mmol, 92%) as a colourless oil. SFC analysis showed an enantiomeric excess of 94%.

^{1}H NMR (400 MHz, $CDCl_3$) δ 6.97 (dt, *J* = 8.3, 1.4 Hz, 1H, C(7)-H), 6.92 (m, 2H, C(Ar)-H), 4.09 (q, *J* = 7.1 Hz, 2H, C(13)-H_2), 2.66 (dp, *J* = 9.0, 6.8 Hz, 1H, C(4)-H), 2.25 (d, *J* = 2.0 Hz, 3H, C(12)-H_3), 2.17 (m, 2H, C(2)-H_2), 1.87 (m, 2H, C(3)-H_2), 1.24 (m, 6H, C(5)-H_3 and C(14)-H_3).

^{13}C NMR (101 MHz, $CDCl_3$) δ 173.6 (C(1)), 159.9 (d, *J* = 242.4 Hz, C(9)), 141.7 (d, *J* = 3.6 Hz, C(6)), 129.9 (d, *J* = 5.0 Hz, C(11)), 125.5 (d, *J* = 7.8 Hz, C(7)), 124.5 (d, *J* = 17.1 Hz, C(10)), 114.8 (d, *J* = 22.1 Hz, C(8)), 60.2 (C(13)), 38.7 (C(4)), 33.3 (C(2)), 32.5 (C(3)), 22.3 (C(5)), 14.6 (d, *J* = 3.6 Hz, C(12)), 14.2 (C(14)).

^{19}F (13C)NMR (376 MHz, $CDCl_3$) δ −121.63.

IR (CH_3Cl film): 2961 (w), 2361 (w), 1725 (w), 1504 (s), 1453 (w), 1375 (w), 1247 (m), 1213 (s), 1177 (m), 1121 (m), 1035 (m), 885 (w), 821 (m), 762 (s) cm^{-1}.

HRMS (ESI): m/z calculated for $C_{14}H_{20}O_2F^+$ $[M+H]^+$ 239.1442 found 239.1444.

SFC: Chiralpak® IG, 1500 psi, 30 °C; flow: 1.0 mL/min; isocratic: 100% CO_2 for 3 min then gradient: 0% to 10% MeOH/CO_2 over 5 min, 96.8:3.2 e.r. (minor enantiomer t_R = 4.62 min, major enantiomer t_R = 3.75 min).

$\boldsymbol{\alpha_D^{25}}$ = +22.0 (c = 1.0, $CHCl_3$).

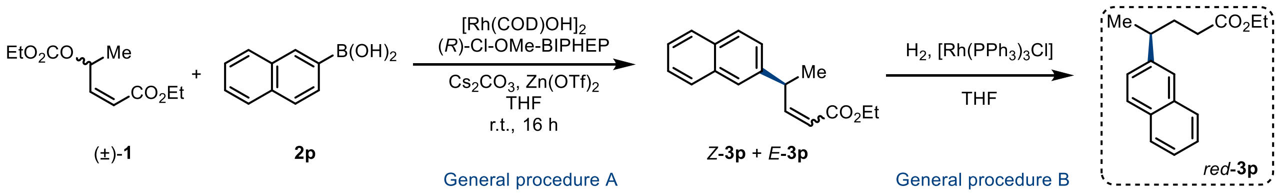

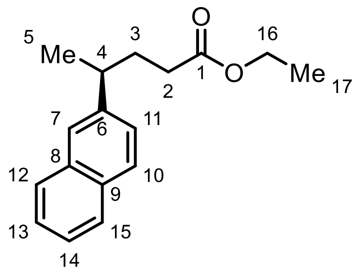

(+)-Ethyl (*S*)-4-(naphthalen-2-yl)pentanoate *red*-3p

(+)-Ethyl (*S*)-4-(naphthalen-2-yl)pentanoate *red*-**3p** was prepared using General procedure A with naphthalen-2-ylboronic acid **2p**, followed by reduction of the resulting crude mixture of products (Z:E=5.4:1) using General procedure B. Purification by automated medium-pressure chromatography (E_2tO/hexane = 0/100 to 15/85) afforded (+)-ethyl (*S*)-4-(naphthalen-2-yl)pentanoate *red*-**3p** (99.5 mg, 0.39 mmol, 97%) as a colourless oil. SFC analysis showed an enantiomeric excess of 90%.

^{1}H NMR (400 MHz, $CDCl_3$) δ 7.81 (m, 3H, C(Ar)-H x3), 7.62 (d, *J* = 2.0 Hz, 1H, C(7)-H), 7.47 (t, *J* = 7.1 Hz, 1H, C(Ar)-H), 7.44 (t, *J* = 6.6 Hz, 1H, C(Ar)-H), 7.36 (dd, *J* = 8.5, 1.8 Hz, 1H, C(Ar)-H), 4.08 (q, *J* = 7.2 Hz, 2H, C(16)-H_2), 2.92 (h, *J* = 7.1 Hz, 1H, C(4)-H), 2.23 (m, 2H, C(2)-H_2), 2.03 (m, 2H, C(3)-H_2), 1.38 (d, *J* = 6.9 Hz, 3H, C(5)-H_3), 1.22 (t, *J* = 7.4 Hz, 3H, C(17)-H_3).

^{13}C NMR (101 MHz, $CDCl_3$) δ 173.7 (C(1)), 143.7 (C(6)), 133.6 (C(8)), 132.3 (C(9)), 128.2 (C(Ar)), 127.62 (C(Ar)), 127.59 (C(Ar)), 126.0 (C(Ar)), 125.6 (C(Ar)), 125.4 (C(Ar)), 125.3 (C(Ar)), 60.2 (C(16)), 39.6 (C(4)), 33.1 (C(2)), 32.6 (C(3)), 22.2 (C(5)), 14.2 (C(17)).

IR (CH_3Cl film): 2965 (w), 1729 (w), 1507 (w), 1457 (w), 1374 (w), 1216 (m), 1032 (m), 885 (w), 856 (w), 820 (w), 753 (s), 668 (w) cm^{-1}.

HRMS (ESI): m/z calculated for $C_{17}H_{20}O_2Na^+$ $[M+Na]^+$ 279.1356 found 279.1355.

SFC: Chiralpak® IG, 1500 psi, 30 °C; flow: 1.0 mL/min; 1% to 30% MeOH over 5 min, 95.1:4.9 e.r. (minor enantiomer t_R = 2.79 min, major enantiomer t_R = 2.51 min).

$\boldsymbol{\alpha_D^{25}}$ = +22.3 (c = 1.0, $CHCl_3$).

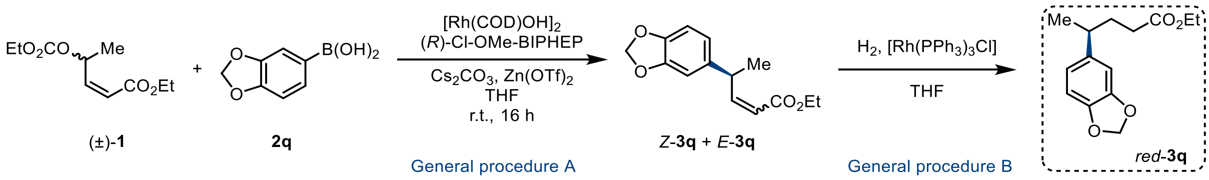

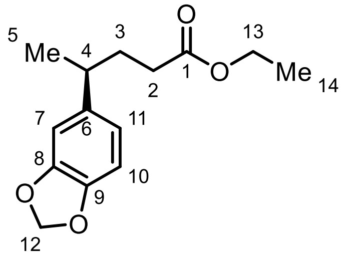

(+)-Ethyl (*S*)-4-(benzo[d][1,3]dioxol-5-yl)pentanoate *red*-3q

(+)-Ethyl (*S*)-4-(benzo[d][1,3]dioxol-5-yl)pentanoate *red*-**3q** was prepared using General procedure A with benzo[d][1,3]dioxol-5-ylboronic acid **2q**, followed by reduction of the resulting crude mixture of products (Z:E=13:1) using General procedure B. Purification by automated medium-pressure chromatography (E_2tO/hexane = 0/100 to 20/80) afforded (+)-ethyl (*S*)-4-(benzo[d][1,3]dioxol-5-yl)pentanoate *red*-**3q** (83.1 mg, 0.33 mmol, 83%) as a colourless oil. SFC analysis showed an enantiomeric excess of 92%.

^{1}H NMR (400 MHz, $CDCl_3$) δ 6.72 (d, *J* = 7.9 Hz, 1H, C(10)-H), 6.67 (d, *J* = 1.7 Hz, 1H, C(7)-H), 6.61 (dd, *J* = 7.9, 1.8 Hz, 1H, C(11)-H), 5.91 (s, 2H, C(12)-H_2), 4.09 (q, *J* = 7.3 Hz, 2H, C(13)-H_2), 2.64 (h, *J* = 6.8 Hz, 1H, C(4)-H), 2.17 (m, 2H, C(2)-H_2), 1.86 (m, 2H, C(3)-H_2), 1.23 (m, 6H, C(5)-H_3 and C(14)-H_3).

^{13}C NMR (101 MHz, $CDCl_3$) δ 173.6 (C(1)), 147.7 (C(6)), 145.8 (C(8)), 140.3 (C(9)), 120.0 (C(7)), 108.1 (C(11)), 107.2 (C(10)), 100.8 (C(12)), 60.2 (C(13)), 39.2 (C(4)), 33.4 (C(2)), 32.5 (C(3)), 22.4 (C(5)), 14.2 (C(14)).

IR (CH_3Cl film): 2962 (m), 1732 (s), 1505 (m), 1487 (s), 1440 (m), 1374 (m), 1214 (s), 1039 (s), 938 (m), 859 (w), 812 (m), 755 (s), 639 (w) cm^{-1}.

HRMS (ESI): m/z calculated for $C_{14}H_{18}O_4Na^+$ $[M+Na]^+$ 273.1097 found 273.1096.

SFC: Chiralpak® IG, 1500 psi, 30 °C; flow: 1.0 mL/min; 1% to 30% MeOH over 5 min, 96.0:4.0 e.r. (minor enantiomer t_R = 2.53 min, major enantiomer t_R = 2.79 min).

α_D^{25} = +26.5 (c = 1.0, $CHCl_3$).

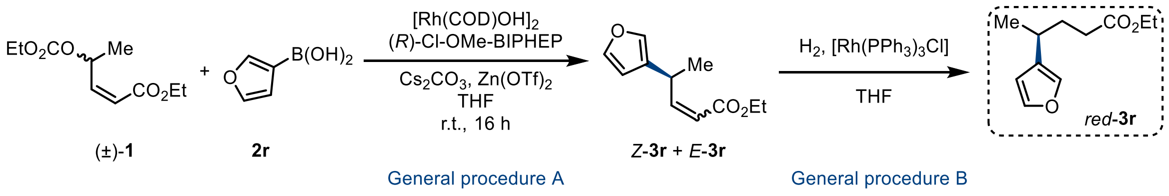

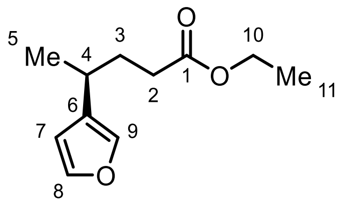

(+)-Ethyl (*S*)-4-(furan-3-yl)pentanoate *red*-**3r**

(+)-Ethyl (*S*)-4-(furan-3-yl)pentanoate *red*-**3r** was prepared using General procedure A with furan-3-ylboronic acid **2r**, followed by reduction of the resulting crude mixture of products (*Z*:*E*=1.5:1) using General procedure B. Purification by automated medium-pressure chromatography (Et_2O/hexane = 0/100 to 35/65) afforded (+)-ethyl (*S*)-4-(furan-3-yl)pentanoate *red*-**3r** (66.7 mg, 0.34 mmol, 85%) as a colourless oil. SFC analysis showed an enantiomeric excess of 76%.

^{1}H NMR (400 MHz, $CDCl_3$) δ 7.35 (t, *J* = 1.7 Hz, 1H, C(Ar)-H), 7.21 (dt, *J* = 1.6, 0.8 Hz, 1H, C(Ar)-H), 6.28 (dd, *J* = 2.0, 0.9 Hz, 1H, C(Ar)-H), 4.11 (q, *J* = 7.2 Hz, 2H, C(10)-H_2), 2.68 (h, *J* = 6.9 Hz, 1H, C(4)-H), 2.25 (t, *J* = 8.0 Hz, 2H, C(2)-H_2), 1.83 (m, 2H, C(3)-H_2), 1.24 (t, *J* = 7.1 Hz, 3H, C(11)-H_3), 1.21 (d, *J* = 6.9 Hz, 3H, C(5)-H_3).

^{13}C NMR (101 MHz, $CDCl_3$) δ 173.7 (C(1)), 142.9 (C(Ar)), 138.3 (C(Ar)), 129.7 (C(6)), 109.3 (C(7)), 60.3 (C(10)), 32.5 (C(4)), 32.2 (C(2)), 29.7 (C(3)), 21.1 (C(5)), 14.2 (C(11)).

IR (CH_3Cl film): 2962 (w), 1733 (s), 1456 (w), 1375 (w), 1164 (s), 1027 (m), 874 (m), 755 (s), 668 (w) cm^{-1}.

HRMS (ESI): m/z calculated for $C_{11}H_{17}O_3^+$ $[M+H]^+$ 197.1172 found 197.1174.

SFC: Chiralpak® IG, 1500 psi, 30 °C; flow: 1.0 mL/min; 1% to 30% MeOH over 5 min, 87.8:12.2 e.r. (minor enantiomer t_R = 1.53 min, major enantiomer t_R = 1.36 min).

α_D^{25} = +12.9 (c = 1.0, $CHCl_3$).

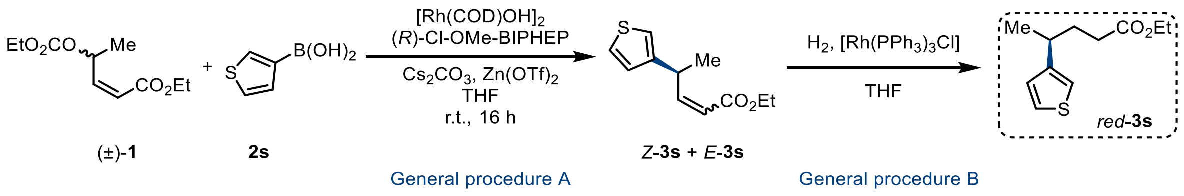

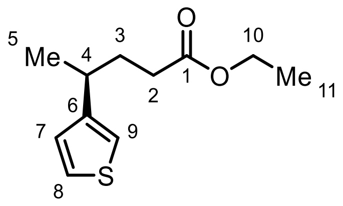

(+)-Ethyl (*S*)-4-(thiophen-3-yl)pentanoate *red*-3s

(+)-Ethyl (*S*)-4-(thiophen-3-yl)pentanoate *red*-**3s** was prepared using General procedure A with thiophen-3-ylboronic acid **2s**, followed by reduction of the resulting crude mixture of products (*Z*:*E*=2.8:1) using General procedure B. Purification by automated medium-pressure chromatography (Et_2O/hexane = 0/100 to 30/70) afforded (+)-ethyl (*S*)-4-(thiophen-3-yl)pentanoate *red*-**3s** (66.2 mg, 0.31 mmol, 78%) as a colourless oil. SFC analysis showed an enantiomeric excess of 91%.

^{1}H NMR (400 MHz, $CDCl_3$) δ 7.17 (dd, J = 4.9, 2.9 Hz, 1H, C(Ar)-H), 6.88 (dd, J = 5.0, 1.3 Hz, 1H, C(Ar)-H), 6.86 (dd, J = 3.0, 1.4 Hz, 1H, C(Ar)-H), 4.02 (q, J = 7.2 Hz, 2H, C(10)-H_2), 2.80 (h, J = 7.0 Hz, 1H, C(4)-H), 2.14 (m, 2H, C(2)-H_2), 1.82 (m, 2H, C(3)-H_2), 1.19 (d, J = 7.0 Hz, 3H, C(5)-H_3), 1.15 (t, J = 7.1 Hz, 3H, C(11)-H_3).

^{13}C NMR (101 MHz, $CDCl_3$) δ 173.7 (C(1)), 147.3 (C(4)), 126.6 (C(Ar)), 125.5 (C(Ar)), 119.4 (C(Ar)), 60.2 (C(10)), 34.7 (C(4)), 33.1 (C(2)), 32.4 (C(3)), 21.7 (C(5)), 14.2 (C(11)).

IR (CH_3Cl film): 2966 (w), 2361 (w), 1727 (w), 1457 (w), 1376 (w), 1322 (w), 1216 (m), 1035 (m), 1033 (w), 931 (w), 852 (w), 753 (s), 668 (w) cm^{-1}.

HRMS (GC EI MS): m/z calculated for $C_{11}H_{16}O_2S^+$ $[M]^+$ 212.08655 found 212.09113.

SFC: Chiralpak® IG, 1500 psi, 30 °C; flow: 1.0 mL/min; 1% to 30% MeOH over 5 min, 95.5:4.5 e.r. (minor enantiomer t_R = 2.03 min, major enantiomer t_R = 1.79 min).

α_D^{25} = +24.8 (c = 1.0, $CHCl_3$).

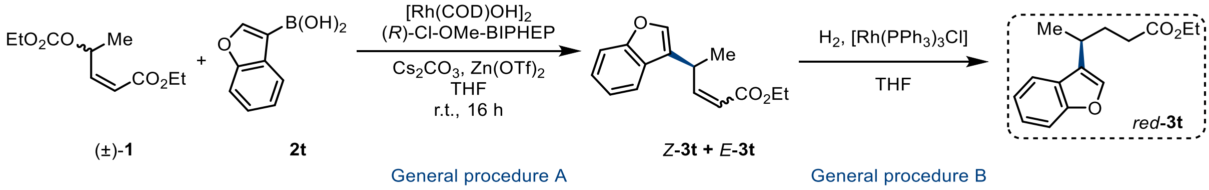

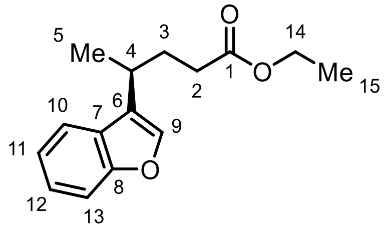

(+)-Ethyl (*S*)-4-(benzofuran-3-yl)pentanoate *red*-**3t**

(+)-Ethyl (*S*)-4-(benzofuran-3-yl)pentanoate *red*-**3t** was prepared using General procedure A with benzofuran-3-ylboronic acid **2t**, followed by reduction of the resulting crude mixture of products (*Z*:*E*=1.8:1) using General procedure B. Purification by automated medium-pressure chromatography (Et_2O/hexane = 0/100 to 25/75) afforded (+)-ethyl (*S*)-4-(benzofuran-3-yl)pentanoate *red*-**3t** (60.1 mg, 0.24 mmol, 61%) as a colourless oil. SFC analysis showed an enantiomeric excess of 99%.

^{1}H NMR (400 MHz, $CDCl_3$) δ 7.61 (ddd, *J* = 7.6, 1.5, 0.8 Hz, 1H, C(Ar)-H), 7.47 (dt, *J* = 8.3, 0.9 Hz, 1H, C(Ar)-H), 7.40 (d, *J* = 0.8 Hz, 1H, C(9)-H), 7.27 (m, 1H, C(Ar)-H), 7.22 (td, *J* = 7.4, 1.1 Hz, 1H, C(Ar)-H), 4.09 (q, *J* = 7.1 Hz, 2H, C(14)-H_2), 3.00 (h, *J* = 7.0 Hz, 1H, C(4)-H), 2.32 (t, *J* = 7.9 Hz, 2H, C(2)-H_2), 2.12 (dq, *J* = 13.6, 7.6 Hz, 1H, C(3)-H), 1.98 (ddt, *J* = 13.8, 8.1, 7.2 Hz, 1H, C(3)-H), 1.38 (d, *J* = 7.0 Hz, 3H, C(5)-H_3), 1.22 (t, *J* = 7.1 Hz, 3H, C(15)-H_3).

^{13}C NMR (101 MHz, $CDCl_3$) δ 173.6 (C(1)), 155.6 (C(8)), 140.6 (hC(9)), 127.3 (C(Ar)), 124.8 (C(Ar)), 124.1 (C(Ar)), 122.2 (C(Ar)), 120.2 (C(Ar)), 111.6 (C(13)), 60.3 (C(14)), 32.3 (C(4)), 31.5 (C(2)), 29.4 (C(3)), 20.3 (C(5)), 14.2 (C(15)).

IR (CH_3Cl film): 2960 (w), 1728 (w), 1512 (m), 1453 (w), 1243 (m), 1211 (s), (m), 1103 (w), 1034 (m), 885 (w), 762 (s), 668 (w) cm^{-1}.

HRMS (ESI): m/z calculated for $C_{15}H_{18}O_3Na^+$ $[M+Na]^+$ 269.1148 found 269.1148.

SFC: Chiralpak® IG, 1500 psi, 30 °C; flow: 1.0 mL/min; 1% to 30% MeOH over 5 min, 99.7:0.3 e.r. (minor enantiomer t_R = 2.67 min, major enantiomer t_R = 2.26 min).

$\boldsymbol{\alpha}_{\mathbf{D}}^{\mathbf{25}}$ = +20.9 (c = 1.0, $CHCl_3$).

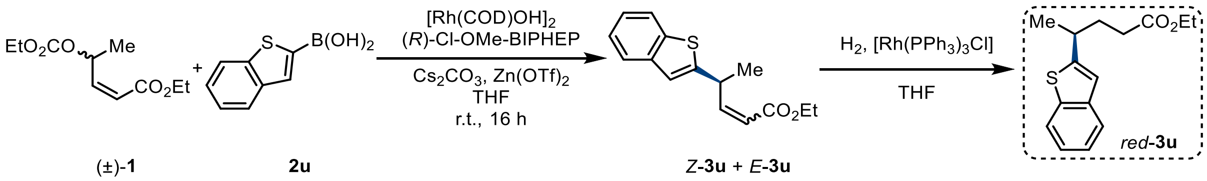

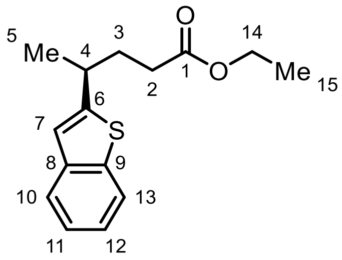

(+)-Ethyl (*S*)-4-(benzo[b]thiophen-2-yl)pentanoate *red*-3u

(+)-Ethyl (*S*)-4-(benzo[b]thiophen-2-yl)pentanoate *red*-**3u** was prepared using General procedure A with benzo[b]thiophen-3-yl boronic acid **2u**, followed by reduction of the resulting crude mixture of products (*Z*:*E*=2.0:1) using General procedure B. Purification by automated medium-pressure chromatography (Et_2O/hexane = 0/100 to 20/80) afforded (+)-ethyl (*S*)-4-(benzo[b]thiophen-3-yl)pentanoate *red*-**3u** (52.5 mg, 0.20 mmol, 50%) as a colourless oil. SFC analysis showed an enantiomeric excess of 80%.

^{1}H NMR (400 MHz, $CDCl_3$) δ 7.78 (ddt, *J* = 7.8, 1.4, 0.8 Hz, 1H, C(Ar)-H), 7.68 (ddd, *J* = 7.8, 1.4, 0.7 Hz, 1H, C(Ar)-H), 7.29 (m, 2H, C(Ar)-H x2), 7.03 (d, *J* = 0.8 Hz, 1H, C(7)-H), 4.11 (q, *J* = 7.2 Hz, 2H, C(14)-H_2), 3.15 (m, 1H, C(4)-H), 2.32 (m, 2H, C(2)-H_2), 2.02 (m, 2H, C(3)-H_2), 1.41 (d, *J* = 6.9 Hz, 3H, C(5)-H_3), 1.24 (t, *J* = 7.1 Hz, 3H, C(15)-H_3).

^{13}C NMR (101 MHz, $CDCl_3$) δ 173.4 (C(1)), 151.3 (C(6)), 139.9 (C(Ar)), 138.9 (C(Ar)), 124.1 (C(Ar)), 123.6 (C(Ar)), 122.9 (C(Ar)), 122.3 (C(Ar)), 119.7 (C(7)), 60.3 (C(14)), 35.6 (C(4)), 33.6 (C(2)), 32.2 (C(3)), 22.7 (C(5)), 14.2 (C(15)).

IR (CH_3Cl film): 2968 (w), 1732 (s), 1457 (w), 1437 (w), 1376 (w), 1316 (m), 1216 (m), 1184 (m), 1097 (w), 1024 (m), 857 (w), 827 (m), 750 (s), 727 (m), 668 (w) cm^{-1}.

HRMS (ESI): m/z calculated for $C_{15}H_{18}O_2NaS^+$ $[M+Na]^+$ 285.0920 found 285.0920.

SFC: Chiralpak® IG, 1500 psi, 30 °C; flow: 1.0 mL/min; 1% to 30% MeOH over 5 min, 89.9:10.1 e.r. (minor enantiomer t_R = 3.40 min, major enantiomer t_R = 2.95 min).

α_D^{25} = +33.2 (c = 1.0, $CHCl_3$).

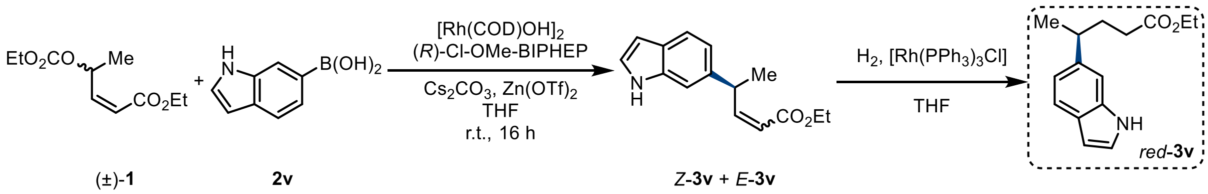

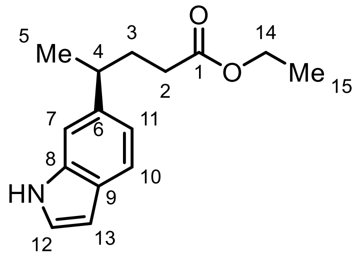

(+)-Ethyl (*S*)-4-(1H-indol-6-yl)pentanoate *red*-3v

(+)-Ethyl (*S*)-4-(1H-indol-6-yl)pentanoate *red*-**3v** was prepared using General procedure A with (1H-indol-6-yl)boronic acid **2v**, followed by reduction of the resulting crude mixture of products (*Z*:*E*=5.0:1) using General procedure B. Purification by automated medium-pressure chromatography (EtOAc/hexane = 0/100 to 30/70) afforded (+)-ethyl (*S*)-4-(1H-indol-6-yl)pentanoate *red*-**3v** (98.1 mg, 0.17 mmol, 43%) as a colourless oil. SFC analysis showed an enantiomeric excess of 96%.

^{1}H NMR (400 MHz, $CDCl_3$) δ 8.10 (s, 1H, -NH), 7.57 (dt, *J* = 8.2, 0.8 Hz, 1H, C(Ar)-H), 7.19 (dt, *J* = 1.4, 0.7 Hz, 1H, C(Ar)-H), 7.16 (dd, *J* = 3.2, 2.4 Hz, 1H, C(Ar)-H), 6.97 (dd, *J* = 8.2, 1.5 Hz, 1H, C(Ar)-H), 6.51 (ddd, *J* = 3.1, 2.0, 1.0 Hz, 1H, C(Ar)-H), 4.08 (q, *J* = 7.2 Hz, 2H, C(14)-H_2), 2.82 (dp, *J* = 8.8, 6.9 Hz, 1H, C(4)-H), 2.21 (m, 2H, C(2)-H_2), 1.97 (m, 2H, C(3)-H_2), 1.33 (d, *J* = 7.0 Hz, 3H, C(5)-H_3), 1.22 (t, *J* = 7.1 Hz, 3H, C(15)-H_3).

^{13}C NMR (101 MHz, $CDCl_3$) δ 174.0 (C(1)), 140.5 (C(6)), 136.2 (C(8)), 126.4 (C(9)), 123.8 (C(Ar)), 120.6 (C(Ar)), 119.5 (C(Ar)), 109.1 (C(Ar)), 102.4 (C(Ar)), 60.2 (C(14)), 39.7 (C(4)), 33.7 (C(2)), 32.8 (C(3)), 22.8 (C(5)), 14.2 (C(15)).

IR (CH_3Cl film): 2962 (w), 1731 (m), 1455 (w), 1376 (w), 1298 (m), 1243 (m), 1150 (m), 1065 (m), 921 (m), 743(s) cm^{-1}.

HRMS (ESI): m/z calculated for $C_{15}H_{19}O_2NNa^+$ $[M+Na]^+$ 268.1308 found 268.1307.

SFC: Chiralpak® IG, 1500 psi, 30 °C; flow: 1.0 mL/min; 1% to 30% MeOH over 5 min, 97.8:2.2 e.r. (minor enantiomer t_R = 3.95 min, major enantiomer t_R = 3.42 min).

$\boldsymbol{\alpha_D^{25}}$ = +34.1 (c = 1.0, $CHCl_3$).

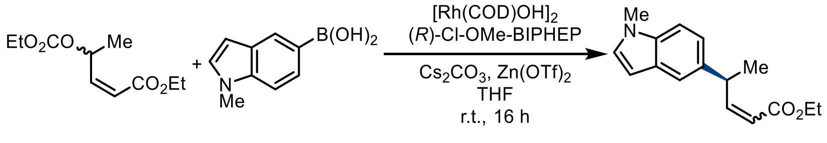

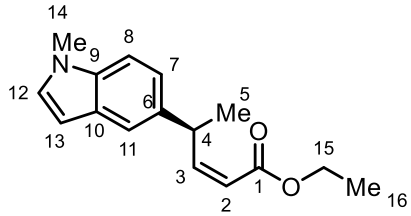

(+)-Ethyl (*S*,*Z*)-4-(1-methyl-1H-indol-5-yl)pent-2-enoate *Z*-3w

(+)-Ethyl (*S*,*Z*)-4-(1-methyl-1H-indol-5-yl)pent-2-enoate *Z*-**3w** was prepared using General procedure A with (1-methyl-1H-indol-5-yl)boronic acid **2w**. Only Z product was observed in the crude mixture. Purification by automated medium-pressure chromatography (Et_2O/hexane = 0/100 to 25/75) afforded (+)-ethyl (*S*,*Z*)-4-(1-methyl-1H-indol-5-yl)pent-2-enoate *Z*-**3w** (46.32 mg, 0.18 mmol, 45%) as a colourless oil. SFC analysis showed an enantiomeric excess of 94%.

^{1}H NMR (400 MHz, $CDCl_3$) δ 7.47 (dd, *J* = 1.8, 0.8 Hz, 1H), 7.18 (dt, *J* = 8.9, 1.0 Hz, 1H), 7.10 (dd, *J* = 8.5, 1.7 Hz, 1H), 6.94 (d, *J* = 3.1 Hz, 1H), 6.36 (dd, *J* = 3.1, 0.8 Hz, 1H), 6.25 (dd, *J* = 11.4, 10.5 Hz, 1H), 5.61 (dd, *J* = 11.4, 1.0 Hz, 1H), 4.92 (dq, *J* = 10.5, 6.9 Hz, 1H), 4.14 (q, *J* = 7.2 Hz, 2H), 3.68 (s, 3H), 1.38 (d, *J* = 7.0 Hz, 3H), 1.24 (t, *J* = 7.1 Hz, 3H).

^{13}C NMR (101 MHz, $CDCl_3$) δ 166.5 (C(1)), 155.0 (C(3)), 135.6 (C(Ar)), 129.1 (C(Ar)), 128.7 (C(Ar)), 121.3 (C(2)), 118.7 (C(Ar)), 116.9 (C(Ar)), 109.3 (C(Ar)), 100.8 (C(Ar)), 59.9 (C(15)), 37.7 (C(6)), 32.8 (C(14)), 21.3 (C(5)), 14.3 (C(16)).

IR (CH_3Cl film): 2961 (w), 1716 (s), 1608 (s), 1504 (s), 1453 (w), 1375 (w), 1169 (m), 1123 (m), 1033 (m), 825 (m), 755 (m), 668 (w) cm^{-1}.

HRMS (ESI): m/z calculated for $C_{16}H_{20}O_2N^+$ $[M+H]^+$ 258.1489 found 258.1489.

SFC: Chiralpak® ID, 1500 psi, 30 °C; flow: 1.0 mL/min; 1% to 30% MeOH over 5 min, 96.8:3.2 e.r. (minor enantiomer t_R = 2.23 min, major enantiomer t_R = 2.15 min).

α_D^{25} = +332.0 (c = 1.0, $CHCl_3$).

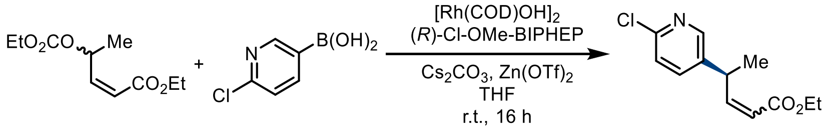

(±)-**1** **2x** *Z*-**3x** (Z only)

General procedure A

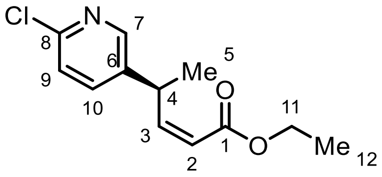

(+)-Ethyl (*S*,*Z*)-4-(6-chloropyridin-3-yl)pent-2-enoate *Z*-3x

(+)-Ethyl (*S*,*Z*)-4-(6-chloropyridin-3-yl)pent-2-enoate *Z*-**3x** was prepared using General procedure A with 6-chloropyridin-3-ylboronic acid **2x**. Only *Z* product was observed in the crude mixture. Purification by automated medium-pressure chromatography (Et_2O/hexane = 0/100 to 45/55) afforded (+)-ethyl (*S*,*Z*)-4-(6-chloropyridin-3-yl)pent-2-enoate *Z*-**3x** (61.4 mg, 0.26 mmol, 64%) as a colourless oil. SFC analysis showed an enantiomeric excess of 99%.

^{1}H NMR (400 MHz, $CDCl_3$) δ 8.31 (dd, *J* = 2.6, 0.7 Hz, 1H, C(7)-H), 7.57 (ddd, *J* = 8.3, 2.6 Hz, 1H, C(10)-H), 7.24 (dd, *J* = 8.2, 0.7 Hz, 1H, C(9)-H), 6.16 (dd, *J* = 11.3, 10.1 Hz, 1H, C(3)-H), 5.78 (dd, *J* = 11.4, 1.0 Hz, 1H, C(2)-H), 4.91 (dq, *J* = 10.2, 7.4 Hz, 1H, C(4)-H), 4.17 (q, *J* = 7.1 Hz, 2H, C(11)-H_2), 1.39 (d, *J* = 7.0 Hz, 3H, C(5)-H_3), 1.28 (t, *J* = 7.1 Hz, 3H, C(12)-H_3).

^{13}C NMR (101 MHz, $CDCl_3$) δ 165.9 (C(1)), 151.4 (C(3)), 149.5 (C(8)), 148.6 (C(7)), 138.9 (C(6)), 137.6 (C(10)), 124.1 (C(2)), 119.3 (C(9)), 60.2 (C(11)), 34.7 (C(4)), 20.6 (C(5)), 14.2 (C(12)).

IR (CH_3Cl film): 1715 (s), 1614 (w), 1457 (s), 1386 (m), 1107 (s), 1111 (m), 1029 (m), 829 (m), 753 (m), 633 (w) cm^{-1}.

HRMS (ESI): m/z calculated for $C_{12}H_{15}O_2NCl^+$ $[M+H]^+$ 240.0786 found 240.0786.

SFC: Chiralpak® IG, 1500 psi, 30 °C; flow: 1.0 mL/min; 1% to 30% MeOH over 5 min, 99.5:0.5 e.r. (minor enantiomer t_R = 2.03 min, major enantiomer t_R = 1.96 min).

α_D^{25} = +321.6 (c = 1.0, $CHCl_3$).

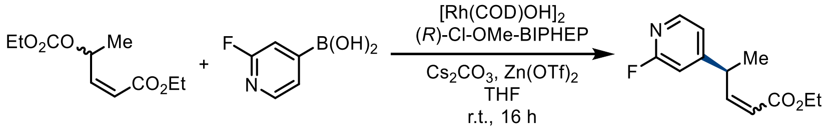

(±)-**1** **2y** *Z*-**3y** (Z only)

General procedure A

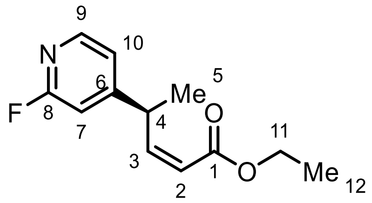

(+)-Ethyl (*S*,*Z*)-4-(2-fluoropyridin-4-yl)pent-2-enoate *Z*-3y

(+)-Ethyl (*S*,*Z*)-4-(2-fluoropyridin-4-yl)pent-2-enoate *Z*-**3y** was prepared using General procedure A with 2-fluoropyridin-4-yl boronic acid **2y**. Only Z product was observed in the crude mixture. Purification by automated medium-pressure chromatography (Et_2O/hexane = 0/100 to 40/60) afforded (+)-ethyl (*S*,*Z*)-4-(2-fluoropyridin-4-yl)pent-2-enoate *Z*-**3y** (46.4 mg, 0.21 mmol, 52%) as a colourless oil. SFC analysis showed an enantiomeric excess of 99%.

^{1}H NMR (400 MHz, $CDCl_3$) δ 8.13 (d, *J* = 2.0 Hz, 1H, C(Ar)-H), 7.71 (tdd, *J* = 7.8, 2.6, 0.5 Hz, 1H, C(Ar)-H), 6.86 (dd, *J* = 8.5, 3.0 Hz, 1H, C(Ar)-H), 6.18 (dd, *J* = 11.3, 10.2 Hz, 1H, C(3)-H), 5.78 (dd, *J* = 11.4, 1.0 Hz, 1H, C(2)-H), 4.94 (dq, *J* = 10.2, 7.0 Hz, 1H, C(4)-H), 4.19 (q, *J* = 7.1 Hz, 2H, C(11)-H_2), 1.41 (d, *J* = 7.0 Hz, 3H, C(5)-H_3), 1.29 (t, *J* = 7.1 Hz, 3H, C(12)-H_3).

^{13}C NMR (101 MHz, $CDCl_3$) δ 166.0 (C(1)), 162.5 (d, *J* = 237.6 Hz, C(8)-H), 151.8 (C(3)), 146.1 (d, *J* = 14.7 Hz, C(Ar)-H), 139.9 (d, *J* = 7.8 Hz, C(Ar)-H), 137.6 (d, *J* = 4.5 Hz, C(Ar)-H), 119.1 (C(2)), 109.3 (d, *J* = 37.4 Hz, C(Ar)-H), 60.2 (C(11)), 34.5 (C(4)), 20.7 (C(5)), 14.2 (C(12)).

^{19}F (13C)NMR (376 MHz, $CDCl_3$) δ −119.4.

IR (CH_3Cl film): 1719 (s), 1621 (w), 1458 (m), 1109(s), 1113 (m), 1033 (m), 829 (m), 755 (s), 638 (w) cm^{-1}.

HRMS (ESI): m/z calculated for $C_{12}H_{15}FNO_2^+$ $[M+H]^+$ 224.1081 found 224.1082.

SFC: Chiralpak® IG, 1500 psi, 30 °C; flow: 1.0 mL/min; 1% to 30% MeOH over 5 min, 99.6:0.4 e.r. (minor enantiomer t_R = 1.94 min, major enantiomer t_R = 1.76 min).

$\boldsymbol{\alpha_D^{25}}$ = +323.8 (c = 1.0, $CHCl_3$).

1,4-Addition of *ortho*-boronic acid to (±)-**1**

The use of *ortho*-boronic acids gave rise to 1,4-addition to (±)-**1** instead of the γ-selective Suzuki-Miyarua reaction. In the case of the reaction between *ortho*-methylphenyl boronic acid and (±)-**1** under the standard reaction conditions, the 1,4-addition product was obtained in 30% yield, as a 1.4:1 ratio of diastereomers with 80% ee in the major diastereomer. Asymmetric 1,4 addition is well described, so we did not pursue these results any further.

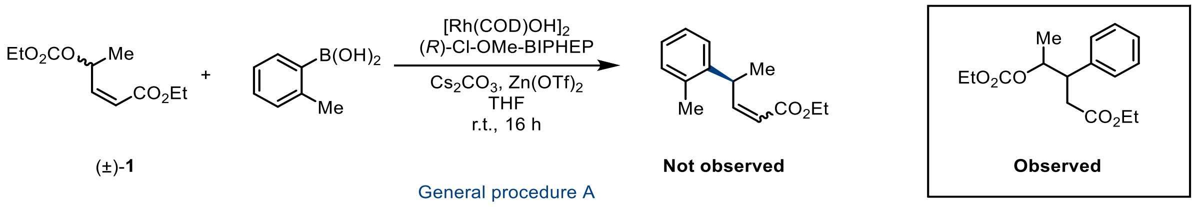

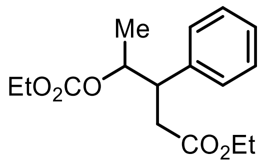

Major diastereomer:

^{1}H NMR (400 MHz, $CDCl_3$) δ 7.23 (dd, J = 7.4, 1.6 Hz, 1H), 7.19 – 7.08 (m, 3H), 4.99 (qd, J = 6.4, 4.9 Hz, 1H), 4.17 (dq, J = 14.2, 7.1 Hz, 2H), 4.09 – 3.92 (m, 2H), 3.82 (ddd, J = 9.2, 6.2, 4.9 Hz, 1H), 2.93 – 2.69 (m, 2H), 2.44 (s, 3H), 1.28 (t, J = 7.1 Hz, 3H), 1.18 (d, J = 6.4 Hz, 3H), 1.10 (t, J = 7.1 Hz, 3H).

^{13}C NMR (101 MHz, $CDCl_3$) δ 172.1, 154.8, 137.7, 137.3, 130.6, 127.2, 126.9, 126.2, 76.2, 64.0, 60.6, 40.9, 35.6, 20.0, 16.4, 14.4, 14.1.

IR (CH_3Cl film): 2987, 2362, 2339, 1744, 1466, 1374, 1270, 1178, 1036 cm^{-1}.

HRMS (ESI): m/z calculated for $C_{17}H_{24}O_5Na^+$ $[M+Na]^+$ 331.1516 found 331.1512.

SFC: Chiralpak® IG, 1500 psi, 30 °C; flow: 1.0 mL/min; 1% to 30% MeOH over 5 min, 89.9:10.1 e.r. (minor enantiomer t_R = 2.40 min, major enantiomer t_R = 2.04 min).

2.4. Rh-catalyzed reactions of (±)-4a-j

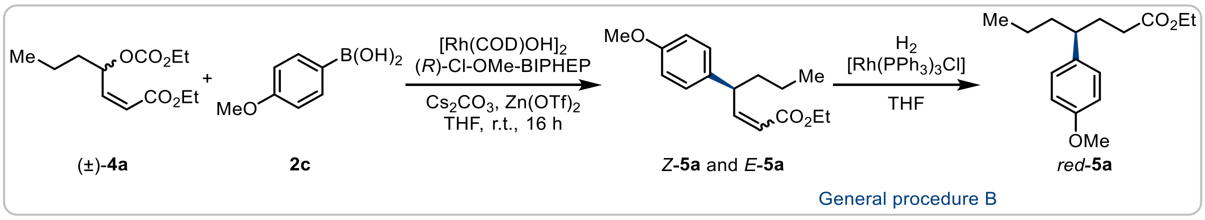

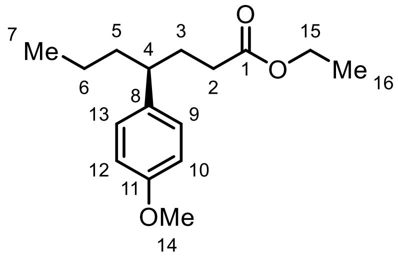

ethyl (*S*)-4-(4-methoxyphenyl)heptanoate *red*-5a

$[Rh(cod)OH]_2$ (4.6 mg, 0.010 mmol, 2.5 mol%) and (*R*)-Cl-MeO-BIPHEP (15.6 mg, 0.024 mmol, 6.0 mol%) were added to 7 mL vial containing a stirring bar and dissolved in dry THF (4.0 mL) under an argon atmosphere at ambient temperature (23 °C). This solution was stirred for 5 minutes.

(4-methoxyphenyl)boronic acid (121.6 mg, 0.80 mmol, 2.0 equiv.), Cs_2CO_3 (130.3 mg, 0.40 mmol, 1.0 equiv.) and $Zn(OTf)_2$ (29.1 mg, 0.08 mmol, 0.2 equiv.) were added to a 7 mL vial containing a stirring bar. To this vial the solution of the rhodium hydroxy complex (4.0 mL) was added *via* syringe under an argon atmosphere. The allylic carbonate (±)-**4a** (97.7 mg, 0.40 mmol, 1.0 equiv.) was added *via* microsyringe and the reaction mixture was stirred at ambient temperature (23 °C) for 16 hours.

The mixture was diluted with hexane (4.0 mL) and filtered through a plug of silica.

The resulting crude mixture of products, *Z*-**5a** and *E*-**5a** (*Z:E* = 7.1:1) was: hydrogen (~1 atm, from a balloon) was bubbled through a solution of $[RhCl(PPh_3)_3]$ (37.1 mg, 0.040 mmol, 0.10 equiv.) in THF (0.70 mL) for 5 minutes. A mixture of *Z*-**5a** and *E*-**5a** was dissolved in THF (0.30 mL) was then added *via* syringe to the Rh-catalyst solution. Hydrogen (~1 atm, from a balloon) was bubbled through the reaction mixture for a further 5 minutes. The reaction mixture was equipped with a hydrogen balloon and stirred at ambient temperature (23 °C) for 16 h. Then, the mixture was diluted with hexane (4.0 ml) and filtered through a plug of silica. The crude was loaded onto Chem Tube-Hydromatrix and automated medium-pressure chromatography (Et_2O/hexane = 0/100 to 15/85) was performed to afford ethyl (*S*)-4-(4-

methoxyphenyl)heptanoate *red*-**5a** (94.1 mg, 0.36 mmol, 89%) as a colourless oil. SFC analysis showed an enantiomeric excess of 93%.

^{1}H NMR (400 MHz, $CDCl_3$) δ 7.04 (d, *J* = 8.7 Hz, 2H, C(9)-H and C(13)-H), 6.83 (d, *J* = 8.6 Hz, 2H, C(10)-H and C(12)-H), 4.07 (q, *J* = 7.2 Hz, 1H, C(15)-H_2), 3.79 (s, 3H, C(14)-H_3), 2.47 (tt, *J* = 9.9, 5.2 Hz, 1H, C(4)-H), 2.11 (m, 2H, C(2)-H_2), 1.98 (m, 1H, C(3)-H), 1.77 (m, 1H, C(3)-H), 1.56 (m, 2H, C(5)-H_2), 1.20 (m, 5H, C(6)-H_2 and C(16)-H_3), 0.83 (t, *J* = 7.3 Hz, 3H, C(7)-H_3).

^{13}C NMR (101 MHz, $CDCl_3$) δ 173.8 (C(1)), 157.9 (C(11)), 136.8 (C(8)), 128.5 (C(9) and C(13)), 113.8 (C(10) and C(12)), 60.2 (C(15)), 55.2 (C(14)), 44.3 (C(4)), 39.2 (C(5)), 32.5 (C(2)), 32.0 (C(3)), 20.6 (C(6)), 14.2 (C(16)), 14.1 (C(7)).

HRMS (ESI): m/z calculated for $C_{16}H_{25}O_3^+$ $[M+H]^+$ 265.1798 found 265.1798.

IR (CH_3Cl film): 2956 (w), 2932 (w), 2871 (w), 1734 (s), 1612 (w), 1584 (w), 1513 (s), 1464 (w), 1375 (w), 1302 (w), 1248 (s), 1178 (m), 1116 (w), 1037 (m), 810 (m), 762 (w) cm^{-1}.

SFC: Chiralpak® IF, 1500 psi, 30 °C; flow: 0.6 mL/min; 1% to 5% MeOH over 11 min, 96.5:3.5 e.r. (minor enantiomer t_R = 6.19 min, major enantiomer t_R = 6.34 min).

α_D^{25} = +12.3 (c = 1.0, $CHCl_3$).

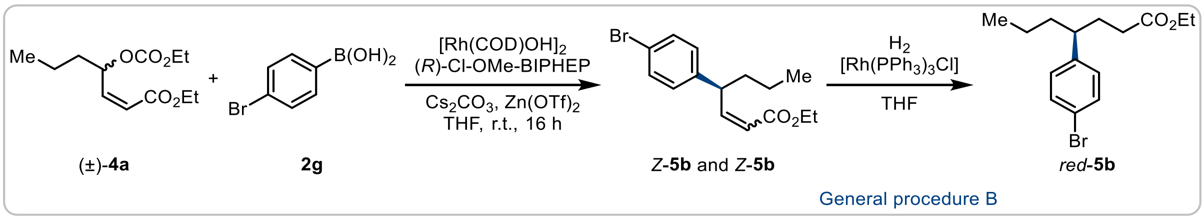

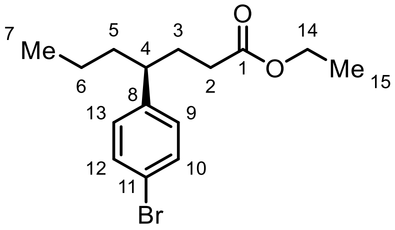

ethyl (*S*)-4-(4-bromophenyl)heptanoate *red*-5b

$[Rh(cod)OH]_2$ (4.6 mg, 0.010 mmol, 2.5 mol%) and (*R*)-Cl-MeO-BIPHEP (15.6 mg, 0.024 mmol, 6.0 mol%) were added to 7 mL vial containing a stirring bar and dissolved in dry THF (4.0 mL) under an argon atmosphere at ambient temperature (23 °C). This solution was stirred for 5 minutes.

(4-bromophenyl)boronic acid (160.7 mg, 0.80 mmol, 2.0 equiv.), Cs_2CO_3 (130.3 mg, 0.40 mmol, 1.0 equiv.) and $Zn(OTf)_2$ (29.1 mg, 0.08 mmol, 0.2 equiv.) were added to a 7 mL vial containing a stirring bar. To this vial a stock solution of the rhodium hydroxy complex (2.0 mL) was added *via* syringe under an argon atmosphere. The allylic carbonate (±)-**4a** (97.7 mg, 0.40 mmol, 1.0 equiv.) was added *via* microsyringe and the reaction mixture was stirred at ambient temperature (23 °C) for 16 hours.

The mixture was diluted with hexane (4.0 mL) and filtered through a plug of silica.

The resulting crude mixture of products, *Z*-**5b** and *E*-**5b** (*Z:E* = 3.6:1) was reduced: hydrogen (~1 atm, from a balloon) was bubbled through a solution of $[RhCl(PPh_3)_3]$ (37.1 mg, 0.040 mmol, 0.10 equiv.) in THF (0.70 mL) for 5 minutes. A mixture of *Z*-**5b** and *E*-**5b** was dissolved in THF (0.30 mL) was then added *via* syringe to the Rh-catalyst solution. Hydrogen (~1 atm, from a balloon) was bubbled through the reaction mixture for a further 5 minutes. The reaction mixture was equipped with a hydrogen balloon and stirred at ambient temperature (23 °C) for 16 h. Then, the mixture was diluted with hexane (4.0 ml) and filtered through a plug of silica. The crude was loaded onto Chem Tube-Hydromatrix and automated medium-pressure chromatography (Et_2O/hexane = 0/100 to 20/80) was performed to afford ethyl (*S*)-4-(4-

bromophenyl)heptanoate *red*-**5b** (122.8 mg, 0.39 mmol, 98%) as a colourless oil. SFC analysis showed an enantiomeric excess of 93%.

^{1}H NMR (400 MHz, $CDCl_3$) δ 7.40 (d, *J* = 8.4 Hz, 2H, C(9)-H and C(13)-H), 7.01 (d, *J* = 8.4 Hz, 2H, C(10)-H and C(12)-H), 4.07 (q, *J* = 7.1 Hz, 2H, C(14)-H_2), 2.50 (tt, *J* = 9.9, 5.1 Hz, 1H, C(4)-H), 2.10 (dd, *J* = 9.1, 7.2 Hz, 2H, C(2)-H_2), 2.00 (m, 1H, C(3)-H), 1.78 (m, 1H, C(3)-H), 1.55 (m, 2H, C(5)-H_2), 1.21 (t, *J* = 7.1 Hz, 3H, C(15)-H_3), 1.15 (m, 2H, C(6)-H_2), 0.83 (t, *J* = 7.3 Hz, 3H, C(7)-H_3).

^{13}C NMR (101 MHz, $CDCl_3$) δ 173.5 (C(1)), 143.8 (C(4)), 131.5 (C(Ar) x2), 129.5 (C(Ar) x2), 119.8 (C(11)), 60.3 (C(14)), 44.7 (C(4)), 38.9 (C(5)), 32.4 (C(3)), 31.7 (C(2)), 20.5 (C(6)), 14.2 (C(15)), 14.0 (C(7)).

HRMS (ESI): m/z calculated for $C_{15}H_{22}O_2Br^+$ $[M+H]^+$ 315.0777 found 315.0777.

IR (CH_3Cl film): 2958 (w), 1732 (m), 1486 (w), 1376 (w), 1217 (w), 1160 (w), 1074 (w), 1035 (w), 1010 (w), 823 (w), 758 (s), 668 (w) cm^{-1}.

SFC: Chiralpak® IG, 1500 psi, 30 °C; flow: 1.0 mL/min; 1% to 30% MeOH over 5 min, 96.5:3.5 e.r. (minor enantiomer t_R = 2.01 min, major enantiomer t_R = 1.90 min).

α_D^{25} = +15.1 (c = 1.0, $CHCl_3$).

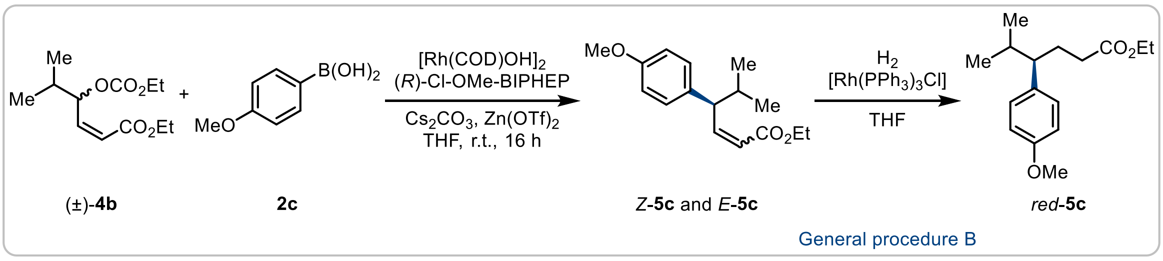

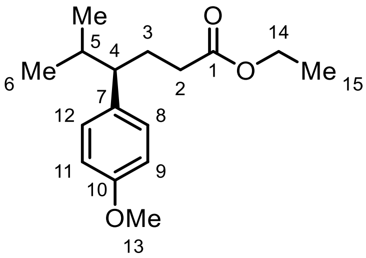

ethyl (*R*)-4-(4-methoxyphenyl)-5-methylhexanoate *red*-5c

[Rh(cod)OH]$_2$ (4.6 mg, 0.010 mmol, 2.5 mol%) and (*R*)-Cl-MeO-BIPHEP (15.6 mg, 0.024 mmol, 6.0 mol%) were added to 7 mL vial containing a stirring bar and dissolved in dry THF (4.0 mL) under an argon atmosphere at ambient temperature (23 °C). This solution was stirred for 5 minutes.

(4-methoxyphenyl)boronic acid (121.6 mg, 0.80 mmol, 2.0 equiv.), Cs_2CO_3 (130.3 mg, 0.40 mmol, 1.0 equiv.) and Zn(OTf)$_2$ (29.1 mg, 0.08 mmol, 0.2 equiv.) were added to a 7 mL vial containing a stirring bar. To this vial a stock solution of the rhodium hydroxy complex (2.0 mL) was added *via* syringe under an argon atmosphere. The allylic carbonate (±)-**4b** (97.7 mg, 0.40 mmol, 1.0 equiv.) was added *via* microsyringe and the reaction mixture was stirred at ambient temperature (23 °C) for 16 hours.

The mixture was diluted with hexane (4.0 mL) and filtered through a plug of silica.

The resulting crude mixture of products, *Z*-**5c** and *E*-**5c** (*Z:E* = 4.7:1) was reduced: hydrogen (~1 atm, from a balloon) was bubbled through a solution of [RhCl(PPh$_3$)$_3$] (37.1 mg, 0.040 mmol, 0.10 equiv.) in THF (0.70 mL) for 5 minutes. A mixture of *Z*-**5c** and *E*-**5c** was dissolved in THF (0.30 mL) was then added *via* syringe to the Rh-catalyst solution. Hydrogen (~1 atm, from a balloon) was bubbled through the reaction mixture for a further 5 minutes. The reaction mixture was equipped with a hydrogen balloon and stirred at ambient temperature (23 °C) for 16 h. Then, the mixture was diluted with hexane (4.0 ml) and filtered through a plug of silica. The crude was loaded onto Chem Tube-Hydromatrix and automated medium-pressure chromatography (E$_2$tO/hexane = 0/100 to 15/85) was performed to afford ethyl (*R*)-4-(4-

methoxyphenyl)-5-methylhexanoate *red*-**5c** (60.3 mg, 0.23 mmol, 57%) as a colourless oil. SFC analysis showed an enantiomeric excess of >99%.

^{1}H NMR (400 MHz, $CDCl_3$) δ 7.01 (d, *J* = 8.7 Hz, 2H, C(8)-H and C(12)-H), 6.82 (d, *J* = 8.7 Hz, 1H, C(9)-H and C(11)-H), 4.06 (q, *J* = 7.2 Hz, 2H, C(14)-H_2), 3.79 (s, 3H, C(13)-H_3), 2.16 (m, 1H, C(4)-H and C(5)-H), 2.04 (m, 1H, C(2)-H_2), 1.80 (m, 1H, C(3)-H_2), 1.21 (t, *J* = 7.1 Hz, 3H, C(15)-H_3), 0.96 (d, *J* = 6.6 Hz, 3H, C(6)-H_3), 0.71 (d, *J* = 6.7 Hz, 3H, C(6)-H_3).

^{13}C NMR (101 MHz, $CDCl_3$) δ 173.9 (C(1)), 157.9 (C(11)), 135.4 (C(7)), 129.3 (C(8) and C(12)), 113.5 (C(9) and C(11)), 60.1 (C(14)), 55.2 (C(13)), 51.8 (C(4)), 33.6 (C(2)), 32.9 (C(3)), 28.3 (C(5)), 20.9 (C(6)), 20.7 (C(6)), 14.2 (C(15)).

HRMS (ESI): m/z calculated for $C_{16}H_{25}O_3^+$ $[M+H]^+$ 265.1798 found 265.1798.

IR (CH_3Cl film): 2958 (w), 2874 (w), 1733 (s), 1611 (w), 1512 (s), 1466 (w), 1369 (w), 1303 (w), 1248 (s), 1216 (m), 1179 (m), 1038 (m), 828 (w), 757 (s), 668 (w) cm^{-1}.

SFC: Chiralpak® IG, 1500 psi, 30 °C; flow: 1.0 mL/min; 1% to 30% MeOH over 5 min, 99.8:0.2 e.r. (minor enantiomer t_R = 1.97 min, major enantiomer t_R = 1.82 min).

α_D^{25} = +9.0 (c = 1.0, $CHCl_3$).

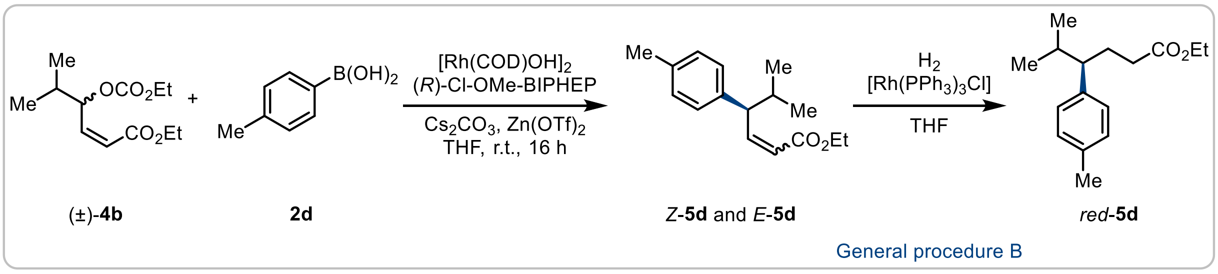

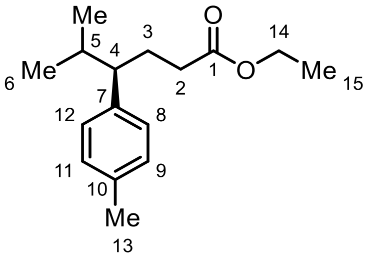

ethyl (*R*)-5-methyl-4-(*p*-tolyl)hexanoate *red*-5d

$[Rh(cod)OH]_2$ (4.6 mg, 0.010 mmol, 2.5 mol%) and (*R*)-Cl-MeO-BIPHEP (15.6 mg, 0.024 mmol, 6.0 mol%) were added to 7 mL vial containing a stirring bar and dissolved in dry THF (4.0 mL) under an argon atmosphere at ambient temperature (23 °C). This solution was stirred for 5 minutes.

p-tolylboronic acid (108.8 mg, 0.80 mmol, 2.0 equiv.), Cs_2CO_3 (130.3 mg, 0.40 mmol, 1.0 equiv.) and $Zn(OTf)_2$ (29.1 mg, 0.08 mmol, 0.2 equiv.) were added to a 7 mL vial containing a stirring bar. To this vial a stock solution of the rhodium hydroxy complex (2.0 mL) was added *via* syringe under an argon atmosphere. The allylic carbonate (±)-**4b** (97.7 mg, 0.40 mmol, 1.0 equiv.) was added *via* microsyringe and the reaction mixture was stirred at ambient temperature (23 °C) for 16 hours.

The mixture was diluted with hexane (4.0 mL) and filtered through a plug of silica.

The resulting crude mixture of products, *Z*-**5d** and *E*-**5d** (*Z:E* = 2.8:1) was reduced: hydrogen (~1 atm, from a balloon) was bubbled through a solution of $[RhCl(PPh_3)_3]$ (37.1 mg, 0.040 mmol, 0.10 equiv.) in THF (0.70 mL) for 5 minutes. A mixture of *Z*-**5d** and *E*-**5d** was dissolved in THF (0.30 mL) was then added *via* syringe to the Rh-catalyst solution. Hydrogen (~1 atm, from a balloon) was bubbled through the reaction mixture for a further 5 minutes. The reaction mixture was equipped with a hydrogen balloon and stirred at ambient temperature (23 °C) for 16 h. Then, the mixture was diluted with hexane (4.0 ml) and filtered through a plug of silica. The crude was loaded onto Chem Tube-Hydromatrix and automated medium-pressure chromatography (E_2tO/hexane = 0/100 to 15/85) was performed to afford ethyl (*R*)-5-methyl-

4-(p-tolyl)hexanoate *red*-**5d** (80.5 mg, 0.32 mmol, 81%) as a colourless oil. SFC analysis showed an enantiomeric excess of >99%.

^{1}H NMR (400 MHz, $CDCl_3$) δ 7.08 (d, *J* = 8.0 Hz, 2H, C(Ar)-H x2), 6.98 (d, *J* = 7.9 Hz, 2H, C(Ar)-H x2), 4.06 (q, *J* = 7.1 Hz, 2H, C(14)-H_2), 2.32 (s, 3H, C(13)-H_3), 2.20 (m, 1H, C(4)-H), 2.12 (m, 1H, C(5)-H), 2.05 (m, 2H, C(2)-H_2), 1.82 (m, 2H, C(3)-H_2), 1.20 (t, *J* = 7.1 Hz, 3H, C(15)-H_3), 0.96 (d, *J* = 6.7 Hz, 3H, C(6)-H_3), 0.72 (d, *J* = 6.7 Hz, 3H, C(6)-H_3).

^{13}C NMR (101 MHz, $CDCl_3$) δ 173.9 (C(1)), 140.3 (C(7)), 135.5 (C(10)), 128.8 (C(Ar) x2), 128.3 (C(Ar) x2), 60.1 (C(14)), 52.2 (C(4)), 33.5 (C(5)), 32.9 (C(2)), 28.2 (C(3)), 21.0 (C(13)), 20.9 (C(6)), 20.8 (C(6)), 14.2 (C(15)).

HRMS (ESI): m/z calculated for $C_{16}H_{25}O_2^+$ $[M+H]^+$ 249.1849 found 249.1849.

IR (CH_3Cl film): 2958 (w), 2873 (w), 1736 (s), 1514 (w), 1452 (w), 1370 (w), 1305 (w), 1246 (m), 1217 (m), 1162 (m), 1038 (w), 812 (w), 764 (s), 669 (s), 641 (w), 618 (w) cm^{-1}.

SFC: Chiralpak® IC, 1500 psi, 30 °C; flow: 0.6 mL/min; 1% to 5% MeOH over 8 min, >99.9:0.1 e.r. (minor enantiomer t_R = 3.80 min, major enantiomer t_R = 3.64 min).

α_D^{25} = +11.1 (c = 1.0, $CHCl_3$).

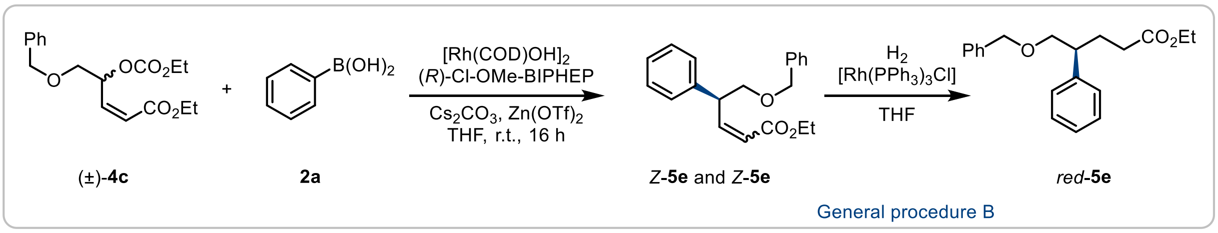

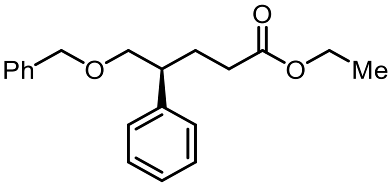

(+)-Ethyl (*S*)-5-(benzyloxy)-4-phenylpent-2-enoate *red*-5e

(+)-Ethyl (*S*)-5-(benzyloxy)-4-phenylpent-2-enoate *red*-**5e** was prepared using General procedure A with phenyl boronic acid **2a**, followed by reduction of the resulting crude mixture of products (Z:E= 2.3:1) using General procedure B. Purification by silica chromatography (Et_2O/hexane = 0/100 to 20/80) afforded (+)-Ethyl (*S*)-5-(benzyloxy)-4-phenylpent-2-enoate *red*-**5e** (96.7 mg, 79%) as a colourless oil. SFC analysis showed an enantiomeric excess of 96%.

^{1}H NMR (400 MHz, $CDCl_3$) δ 7.36 – 7.16 (m, 10H), 4.50 (s, 2H), 4.07 (qd, *J* = 7.1, 0.7 Hz, 2H), 3.67 – 3.55 (m, 2H), 2.92 (ddt, *J* = 11.1, 6.7, 3.8 Hz, 1H), 2.30 – 2.13 (m, 3H), 1.98 – 1.84 (m, 1H), 1.22 (t, *J* = 7.1 Hz, 3H).

^{13}C NMR (101 MHz, $CDCl_3$) δ 173.7, 142.0, 138.5, 128.6, 128.5, 128.1, 127.7, 127.6, 126.8, 74.9, 73.2, 60.4, 45.8, 32.4, 28.1, 14.3.

IR (CH_3Cl film): 3030, 2982, 2865, 2361, 2343, 1735, 1605, 1496, 1454, 1371, 1241, 1205, 1178, 1097, 1030 cm^{-1}.

HRMS (ESI): m/z calculated for $C_{20}H_{24}O_3Na^+$ $[M+Na]^+$ 335.1618 found 335.1619.

SFC: Chiralpak® ID, 1500 psi, 30 °C; flow: 1.0 mL/min; 1% to 5% MeOH over 5 min, 98.0:2.0 e.r. (minor enantiomer t_R = 4.48 min, major enantiomer t_R = 4.61 min).

α^{25}_D = +20.1 (c = 1.00 in $CHCl_3$).

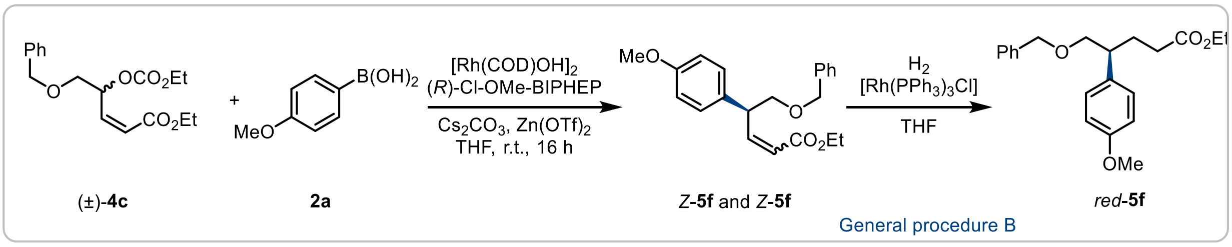

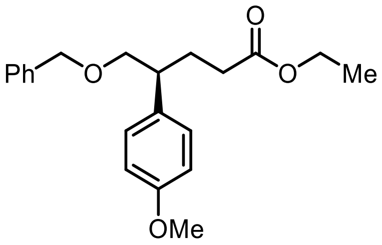

(+)-Ethyl (*S*)-5-(benzyloxy)-4-(4-methoxyphenyl)pent-2-enoate *red*-5f

(+)-Ethyl (*S*)-5-(benzyloxy)-4-(4-methoxyphenyl)pent-2-enoate *red*-**5f** was prepared using General procedure A with *p*-OMephenyl boronic acid **2c**, followed by reduction of the resulting crude mixture of products (Z:E= 2.8:1) using General procedure B. Purification by silica chromatography (Et_2O/hexane = 0/100 to 20/80) afforded (+)-Ethyl (*S*)-5-(benzyloxy)-4-(4-methoxyphenyl)pent-2-enoate *red*-**5f** (105.2 mg, 77%) as a colourless oil. SFC analysis showed an enantiomeric excess of 99%.

^{1}H NMR (400 MHz, $CDCl_3$) δ 7.27 – 7.10 (m, 5H), 7.06 – 6.98 (m, 2H), 6.79 – 6.71 (m, 2H), 4.40 (s, 2H), 4.02 – 3.92 (m, 2H), 3.69 (s, 3H), 3.51 – 3.40 (m, 2H), 2.78 (dtt, *J* = 10.8, 6.6, 3.2 Hz, 1H), 2.18 – 2.05 (m, 3H), 1.85 – 1.68 (m, 1H), 1.12 (t, *J* = 7.1 Hz, 3H).

^{13}C NMR (101 MHz, $CDCl_3$) δ 173.7, 158.4, 138.5, 134.0, 128.9, 128.4, 127.6, 127.6, 114.0, 75.0, 73.1, 60.3, 55.3, 44.9, 32.4, 28.1, 14.3.

IR (CH_3Cl film): 3323, 2939, 2850, 2360, 2343, 1734, 1612, 1584, 1514, 1455, 1422, 1372, 1303, 1250, 1180, 1160, 1101, 1038 cm^{-1}.

HRMS (ESI): m/z calculated for $C_{21}H_{26}O_4Na^+$ $[M+Na]^+$ 365.1725 found 365.1741.

SFC: Chiralpak® IC, 1500 psi, 30 °C; flow: 1.0 mL/min; 1% to 10% MeOH over 5 min, 99.5:0.5 e.r. (minor enantiomer t_R = 5.80 min, major enantiomer t_R = 5.68 min).

α^{25}_D = +21.0 (c = 1.00 in $CHCl_3$).

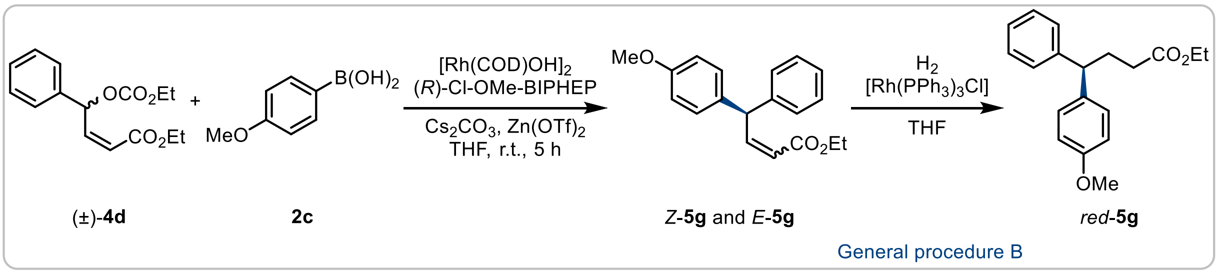

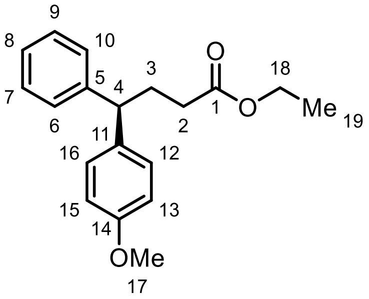

ethyl (*R*)-4-(4-methoxyphenyl)-4-phenylbutanoate *red*-5g

$[Rh(cod)OH]_2$ (4.6 mg, 0.010 mmol, 2.5 mol%) and (*R*)-Cl-MeO-BIPHEP (15.6 mg, 0.024 mmol, 6.0 mol%) were added to 7 mL vial containing a stirring bar and dissolved in dry THF (4.0 mL) under an argon atmosphere at ambient temperature (23 °C). This solution was stirred for 5 minutes.

(4-methoxyphenyl)boronic acid (121.6 mg, 0.80 mmol, 2.0 equiv.), Cs_2CO_3 (130.3 mg, 0.40 mmol, 1.0 equiv.) and $Zn(OTf)_2$ (29.1 mg, 0.08 mmol, 0.2 equiv.) were added to a 7 mL vial containing a stirring bar. To this vial a stock solution of the rhodium hydroxy complex (4.0 mL) was added *via* syringe under an argon atmosphere. The allylic carbonate (±)-**4d** (111.3 mg, 0.40 mmol, 1.0 equiv.) was added *via* microsyringe and the reaction mixture was stirred at ambient temperature (23 °C) for 5 hours.

The mixture was diluted with hexane (4.0 mL) and filtered through a plug of silica.

The resulting crude mixture of products, *Z*-**5e** and *E*-**5e** (*Z*:*E* = 1.7:1) was reduced: hydrogen (~1 atm, from a balloon) was bubbled through a solution of $[RhCl(PPh_3)_3]$ (37.1 mg, 0.040 mmol, 0.10 equiv.) in THF (0.70 mL) for 5 minutes. A mixture of *Z*-**5e** and *E*-**5e** was dissolved in THF (0.30 mL) was then added *via* syringe to the Rh-catalyst solution. Hydrogen (~1 atm, from a balloon) was bubbled through the reaction mixture for a further 5 minutes. The reaction mixture was equipped with a hydrogen balloon and stirred at ambient temperature (23 °C) for 16 h. Then, the mixture was diluted with hexane (4.0 ml) and filtered through a plug of silica. The crude was loaded onto Chem Tube-Hydromatrix and automated medium-pressure

chromatography (E_2tO/hexane = 0/100 to 25/75) was performed to afford ethyl (*R*)-4-(4-methoxyphenyl)-4-phenylbutanoate *red*-**5g** (107.4 mg, 0.36 mmol, 90%) as a colourless oil. SFC analysis showed an enantiomeric excess of 93%. Characterisation data match literature reports.[2]

^{1}H NMR (400 MHz, $CDCl_3$) δ 7.17 (m, 3H), 7.08 (m, 3H), 6.75 (d, *J* = 8.7 Hz, 1H), 4.02 (q, *J* = 7.1 Hz, 2H), 3.80 (t, *J* = 7.8 Hz, 1H), 3.69 (s, 3H), 2.27 (m, 2H), 2.18 (m, 2H), 1.15 (t, *J* = 7.1 Hz, 3H).

^{13}C NMR (101 MHz, $CDCl_3$) δ 173.5, 158.1, 144.6, 136.3, 128.8, 128.5, 127.8, 126.3, 113.9, 60.3, 55.2, 49.7, 32.9, 30.8, 14.3.

HRMS (ESI): m/z calculated for $C_{19}H_{22}O_3Na_2^+$ $[M+Na]^+$ 321.1461 found 321.1461.

IR (CH_3Cl film): 2952 (w), 1732 (s), 1611 (w), 1512 (s), 1453 (w), 1376 (w), 1303 (m), 1250 (s), 1178 (m), 1036 (m), 830 (w), 763 (s), 701 (m), 668 (m), 655 (w) cm^{-1}.

SFC: Chiralpak® IE, 1500 psi, 30 °C; flow: 1.0 mL/min; 1% to 10% MeOH over 5 min, 96.3:3.7 e.r. (minor enantiomer t_R = 5.06 min, major enantiomer t_R = 5.19 min).

α_D^{25} = −3.0 (c = 1.0, $CHCl_3$).

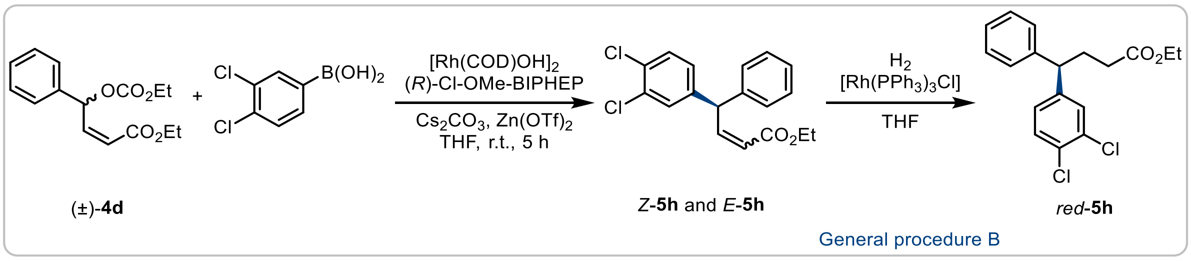

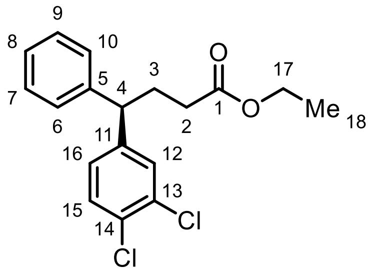

ethyl (*R*)-4-(3,4-dichlorophenyl)-4-phenylbutanoate *red*-5h

$[Rh(cod)OH]_2$ (4.6 mg, 0.010 mmol, 2.5 mol%) and (*R*)-Cl-MeO-BIPHEP (15.6 mg, 0.024 mmol, 6.0 mol%) were added to 7 mL vial containing a stirring bar and dissolved in dry THF (4.0 mL) under an argon atmosphere at ambient temperature (23 °C). This solution was stirred for 5 minutes.

(3,4-dichlorophenyl)boronic acid (152.6 mg, 0.80 mmol, 2.0 equiv.), Cs_2CO_3 (130.3 mg, 0.40 mmol, 1.0 equiv.) and $Zn(OTf)_2$ (29.1 mg, 0.08 mmol, 0.2 equiv.) were added to a 7 mL vial containing a stirring bar. To this vial a stock solution of the rhodium hydroxy complex (2.0 mL) was added *via* syringe under an argon atmosphere. The allylic carbonate (±)-**4d** (111.3 mg, 0.40 mmol, 1.0 equiv.) was added *via* microsyringe and the reaction mixture was stirred at ambient temperature (23 °C) for 5 hours.

The mixture was diluted with hexane (4.0 mL) and filtered through a plug of silica.

The resulting crude mixture of products, *Z*-**5h** and *E*-**5h** (*Z*:*E* = 1.2:1) was reduced: hydrogen (~1 atm, from a balloon) was bubbled through a solution of $[RhCl(PPh_3)_3]$ (37.1 mg, 0.040 mmol, 0.10 equiv.) in THF (0.70 mL) for 5 minutes. A mixture of *Z*-**5h** and *E*-**5h** was dissolved in THF (0.30 mL) was then added *via* syringe to the Rh-catalyst solution. Hydrogen (~1 atm, from a balloon) was bubbled through the reaction mixture for a further 5 minutes. The reaction mixture was equipped with a hydrogen balloon and stirred at ambient temperature (23 °C) for 16 h. Then, the mixture was diluted with hexane (4.0 ml) and filtered through a plug of silica. The crude was loaded onto Chem Tube-Hydromatrix and automated medium-pressure chromatography (E_2tO/hexane = 0/100 to 25/75) was performed to afford ethyl (*R*)-4-(3,4-

dichlorophenyl)-4-phenylbutanoate ***red*-5h** (116.0 mg, 0.34 mmol, 86%) as a colourless oil. SFC analysis showed an enantiomeric excess of 99%. Characterisation data match literature reports.[3]

^{1}H NMR (400 MHz, $CDCl_3$) δ 7.35 (d, *J* = 8.3 Hz, 1H, C(Ar)-H), 7.31 (m, 3H, C(Ar)-H x3), 7.20 (m, 3H, C(Ar)-H x3), 7.07 (ddd, *J* = 8.3, 2.2, 0.5 Hz, 1H, C(Ar)-H), 4.11 (q, *J* = 7.1 Hz, 2H, C(17)-H_2), 3.90 (t, *J* = 7.8 Hz, 1H, C(4)-H), 2.34 (m, 2H, C(2)-H_2), 2.26 (m, 2H, C(3)-H_2), 1.24 (t, *J* = 7.1 Hz, 3H, C(18)-H_3).

^{13}C NMR (101 MHz, $CDCl_3$) δ 173.1 (C(1)), 144.6 (C(Ar)), 142.8 (C(Ar)), 132.5 (C(Ar)), 130.5 (C(Ar)), 130.4 (C(Ar)), 129.8 (C(Ar)), 128.8 (C(Ar) x2), 127.8 (C(Ar) x2), 127.3 (C(Ar)), 126.9 (C(Ar)), 60.5 (C(17)), 49.7 (C(4)), 32.5 (C(2)), 30.3 (C(3)), 14.2 (C(18)).

HRMS (ESI): m/z calculated for $C_{18}H_{19}O_2Cl_2^+$ $[M+H]^+$ 337.0757 found 337.0756.

IR (CH_3Cl film): 3028 (w), 2885 (w), 1732 (s), 1472 (m), 1376 (w), 1239 (m), 1180 (m), 1156 (m), 1030 (m), 886 (m), 763 (s), 700 (m), 678 (w), 654 (w), 642 (w), 618 (w) cm^{-1}.

SFC: Chiralpak® IA, 1500 psi, 30 °C; flow: 1.0 mL/min; 1% to 30% MeOH over 5 min, 99.5:0.5 e.r. (minor enantiomer t_R = 2.52 min, major enantiomer t_R = 2.66 min).

$\boldsymbol{\alpha_D^{25}}$ = −4.5 (c = 1.0, $CHCl_3$).

Absolute configuration was determined by comparing optical rotation of ***red*-5f** to that of (*R*)-methyl 4-(3,4-dichlorophenyl)-4-phenylbutanoate.[4]

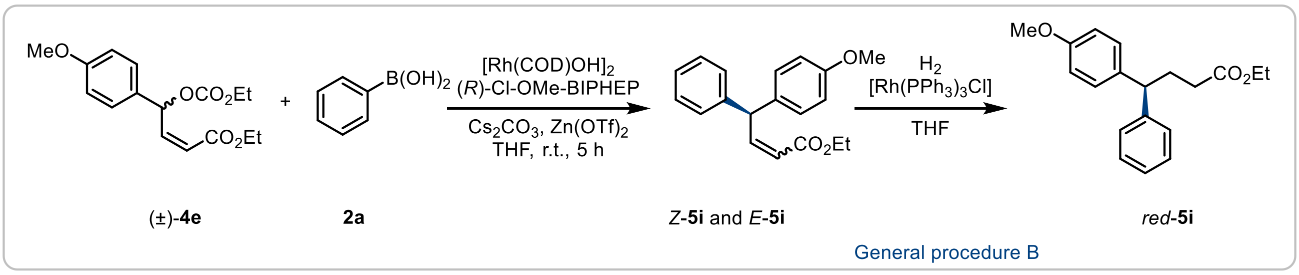

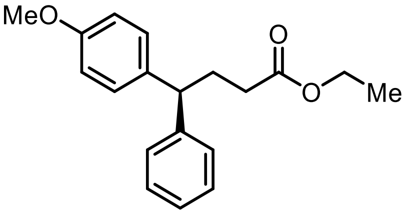

(+)-Ethyl (*S*)-4-(4-methoxyphenyl)-4-phenylbutanoate *red*-5i

(+)-Ethyl (*S*)-4-(4-methoxyphenyl)-4-phenylbutanoate *red*-**5i** was prepared using General procedure A with phenyl boronic acid **2a**, followed by reduction of the resulting crude mixture of products (Z:E= 1:1) using General procedure B. Purification by silica chromatography (Et_2O/hexane = 0/100 to 20/80) afforded (+)-Ethyl (*S*)-4-(4-methoxyphenyl)-4-phenylbutanoate *red*-**5i** (93.5 mg, 78%) as a colourless oil. SFC analysis showed an enantiomeric excess of >99%.

^{1}H NMR (400 MHz, $CDCl_3$) δ 7.35 – 7.22 (m, 4H), 7.22 – 7.13 (m, 3H), 6.88 – 6.81 (m, 2H), 4.11 (q, *J* = 7.1 Hz, 2H), 3.90 (t, *J* = 7.8 Hz, 1H), 3.78 (s, 3H), 2.43 – 2.31 (m, 2H), 2.32 – 2.19 (m, 2H), 1.25 (t, *J* = 7.1 Hz, 3H).

^{13}C NMR (101 MHz, $CDCl_3$) δ 173.6, 158.2, 144.7, 136.4, 128.9, 128.6, 127.9, 126.4, 114.0, 60.4, 55.3, 49.8, 33.0, 30.9, 14.3.

IR (CH_3Cl film): 2952 (w), 1732 (s), 1611 (w), 1512 (s), 1453 (w), 1376 (w), 1303 (m), 1250 (s), 1178 (m), 1036 (m), 830 (w), 763 (s), 701 (m), 668 (m), 655 (w) cm^{-1}.

HRMS (ESI): m/z calculated for $C_{19}H_{22}O_3Na^+$ $[M+Na]^+$ 321.1461 found 321.1469.

SFC: Chiralpak® IE, 1500 psi, 30 °C; flow: 1.0 mL/min; 1% to 10% MeOH over 5 min, 99.8:0.2 e.r. (minor enantiomer t_R = 5.36 min, major enantiomer t_R = 5.23 min).

α^{25}_D = +1.2 (c = 1.00 in $CHCl_3$).

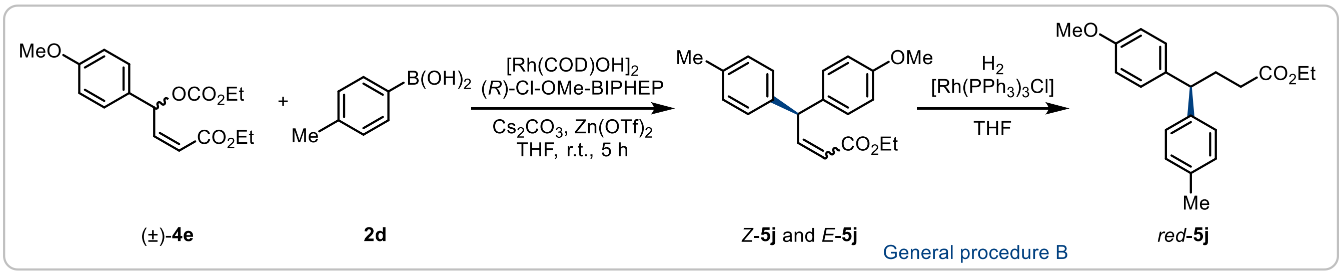

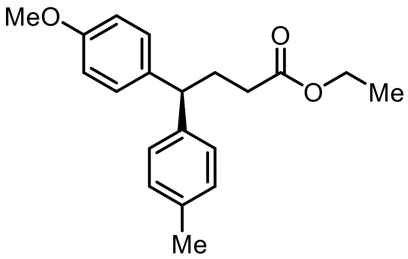

(+)-Ethyl (*S*)-4-(4-methoxyphenyl)-4-(p-tolyl)butanoate *red*-5j

(+)-Ethyl (*S*)-4-(4-methoxyphenyl)-4-(p-tolyl)butanoate *red*-**5j** was prepared using General procedure A with *p*-tolylphenyl boronic acid **2d**, followed by reduction of the resulting crude mixture of products (Z:E= 1:1) using General procedure B. Purification by silica chromatography (Et_2O/hexane = 0/100 to 20/80) afforded (+)-Ethyl (*S*)-4-(4-methoxyphenyl)-4-(p-tolyl)butanoate *red*-**5j** (94.4 mg, 76%) as a colourless oil. SFC analysis showed an enantiomeric excess of 99%.

^{1}H NMR (400 MHz, $CDCl_3$) δ 7.21 – 7.04 (m, 6H), 6.88 – 6.79 (m, 2H), 4.11 (q, *J* = 7.1 Hz, 2H), 3.86 (t, *J* = 7.6 Hz, 1H), 3.78 (s, 3H), 2.34 (ddd, *J* = 8.7, 5.9, 1.3 Hz, 2H), 2.31 (s, 3H), 2.27 (ddd, *J* = 8.6, 6.4, 1.7 Hz, 2H), 1.25 (t, *J* = 7.1 Hz, 3H).

^{13}C NMR (101 MHz, $CDCl_3$) δ 173.6, 158.1, 141.7, 136.7, 135.8, 129.3, 128.8, 127.7, 114.0, 60.4, 55.3, 49.4, 33.0, 30.9, 21.1, 14.3.

IR (CH_3Cl film): 2997, 2936, 2836, 2356, 2338, 1734, 1611, 1584, 1512, 1463, 1420, 1375, 1303, 1250, 1180, 1038 cm^{-1}.

HRMS (ESI): m/z calculated for $C_{20}H_{24}O_3Na^+$ $[M+Na]^+$ 335.1618 found 335.1631.

SFC Chiralpak® IC, 1500 psi, 30 °C; flow: 1.0 mL/min; 0% to 20% MeOH over 10 min, then 20% to 50% MeOH over 1 min, then isocratic at 50% MeOH for 2 min, 99.5:0.5 e.r. (minor enantiomer t_R = 7.35 min, major enantiomer t_R = 7.19 min).

α^{25}_D = +1.3 (c = 1.00 in $CHCl_3$).

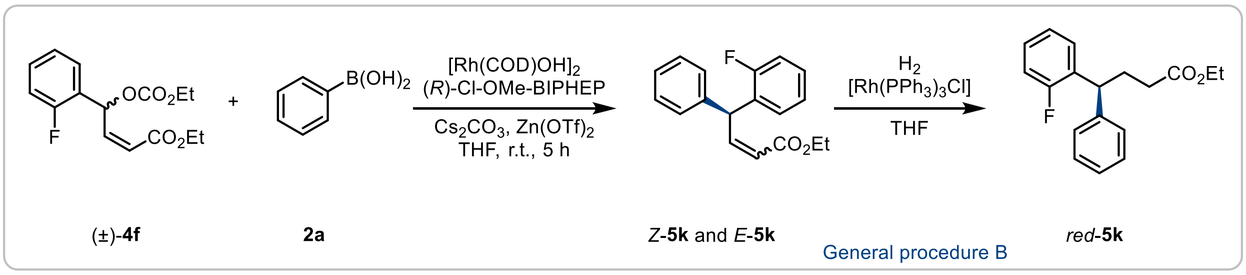

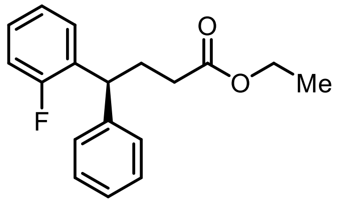

(+)-Ethyl-(*S*)-4-(2-fluorophenyl)-4-phenylbutanoate *red*-5k

(+)-Ethyl-(*S*)-4-(2-fluorophenyl)-4-phenylbutanoate *red*-**5k** was prepared using General procedure A with phenyl boronic acid **2a**, followed by reduction of the resulting crude mixture of products (Z:E= 1:1) using General procedure B. Purification by silica chromatography (Et_2O/hexane = 0/100 to 20/80) afforded (+)-Ethyl-(*S*)-4-(2-fluorophenyl)-4-phenylbutanoate (92.8 mg, 81 %) as a colourless oil. SFC analysis showed an enantiomeric excess of 99%.

^{1}H NMR (400 MHz, $CDCl_3$) δ 7.31 – 7.22 (m, 5H), 7.21 – 7.12 (m, 2H), 7.07 (td, J = 7.5, 1.4 Hz, 1H), 6.98 (ddd, J = 10.4, 8.1, 1.4 Hz, 1H), 4.28 (t, J = 7.9 Hz, 1H), 4.09 (q, J = 7.1 Hz, 2H), 2.47 – 2.32 (m, 2H), 2.32 – 2.25 (m, 2H), 1.22 (t, J = 7.1 Hz, 3H).

^{19}F NMR (376 MHz, $CDCl_3$) δ -117.60 (ddd, J = 10.5, 7.3, 5.1 Hz).

^{13}C NMR (101 MHz, $CDCl_3$) δ 173.4, 160.8 (d, J = 245.5 Hz), 143.1, 131.2 (d, J = 14.4 Hz), 128.6, 128.6 (d, J = 4.5 Hz), 128.0 (d, J = 8.3 Hz), 128.0, 126.6, 124.34 (d, J = 3.6 Hz), 115.6 (d, J = 22.7 Hz), 60.5, 42.9 (d, J = 2.4 Hz), 32.8, 29.8, 14.3.

IR (CH_3Cl film): 3063, 3030, 2982, 2348, 1734, 1602, 1585, 1490, 1455, 1421, 1377, 1342, 1302, 1229, 1183, 1159, 1102, 1035 cm^{-1}.

HRMS (ESI): m/z calculated for $C_{18}H_{19}FO_2Na^+$ $[M+Na]^+$ 309.1261 found 309.1256

SFC: Chiralpak® IG, 1500 psi, 30 °C; flow: 1.0 mL/min; 1% to 10% MeOH over 5 min, 99.5:0.5 e.r. (minor enantiomer t_R = 3.60 min, major enantiomer t_R = 3.44 min).

α^{25}_{D} = +0.7 (c = 1.0, $CHCl_3$).

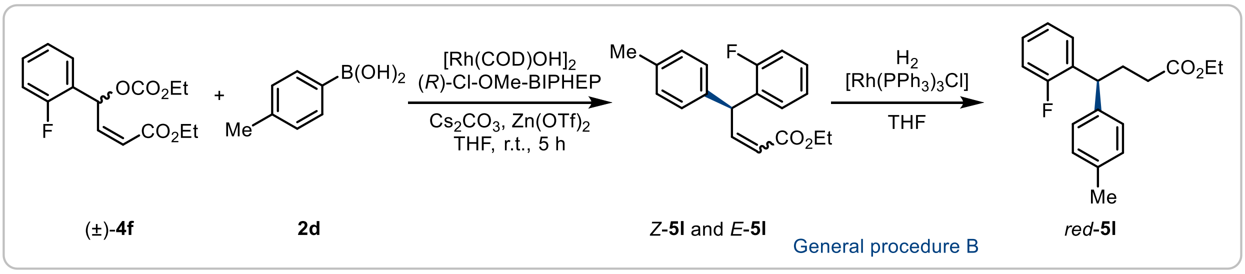

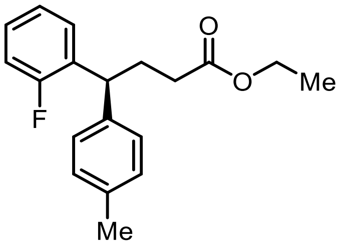

(-)-Ethyl-(*S*)-4-(2-fluorophenyl)-4-(*p*-tolyl)butanoate *red*-5l

(-)-Ethyl-(*S*)-4-(2-fluorophenyl)-4-(*p*-tolyl)butanoate *red*-**5l** was prepared using General procedure A with *p*-tolylphenyl boronic acid **2d**, followed by reduction of the resulting crude mixture of products (Z:E= 2.5:1) using General procedure B. Purification by silica chromatography (Et_2O/hexane = 0/100 to 20/80) afforded (-)-Ethyl-(*S*)-4-(2-fluorophenyl)-4-(*p*-tolyl)butanoate *red*-**5l** (75.7 mg, 63%) as a colourless oil. SFC analysis showed an enantiomeric excess of >99%.

^{1}H NMR (400 MHz, $CDCl_3$) δ 7.32 – 7.24 (m, 1H), 7.21 – 7.13 (m, 3H), 7.13 – 7.07 (m, 3H), 7.00 (ddd, J = 10.4, 8.0, 1.3 Hz, 1H), 4.27 (t, J = 7.6 Hz, 1H), 4.11 (q, J = 7.2 Hz, 2H), 2.48 – 2.34 (m, 2H), 2.31 (d, J = 4.5 Hz, 5H), 1.25 (t, J = 7.1 Hz, 3H).

^{13}C NMR (101 MHz, $CDCl_3$) δ 173.4, 160.8 (d, J = 245.4 Hz), 140.1, 136.2, 131.5 (d, J = 14.4 Hz), 129.3, 128.5 (d, J = 4.4 Hz), 127.93 (d, J = 8.3 Hz), 127.89, 124.3 (d, J = 3.6 Hz), 115.6 (d, J = 22.7 Hz), 60.5, 42.6 (d, J = 2.4 Hz), 32.9, 29.8, 21.1, 14.3.

^{19}F NMR (376 MHz, $CDCl_3$) δ -117.65 – -117.77 (m).

IR (CH_3Cl film): 2982, 2936, 2346, 1734, 1615, 1585, 1514, 1490, 1455, 1420, 1376, 1340, 1301, 1229, 1181, 1157, 1099, 1036, 1023 cm^{-1}.

HRMS (ESI): m/z calculated for $C_{19}H_{21}FO_2Na^+$ $[M+Na]^+$ 323.1418 found 323.1411.

SFC: Chiralpak® IG, 1500 psi, 30 °C; flow: 1.0 mL/min; 1% to 10% MeOH over 5 min, 99.8:0.2 e.r. (minor enantiomer t_R = 4.15 min, major enantiomer t_R = 3.97 min).

$\boldsymbol{\alpha^{25}_{D}}$ = -2.3 (c = 1.0, $CHCl_3$).

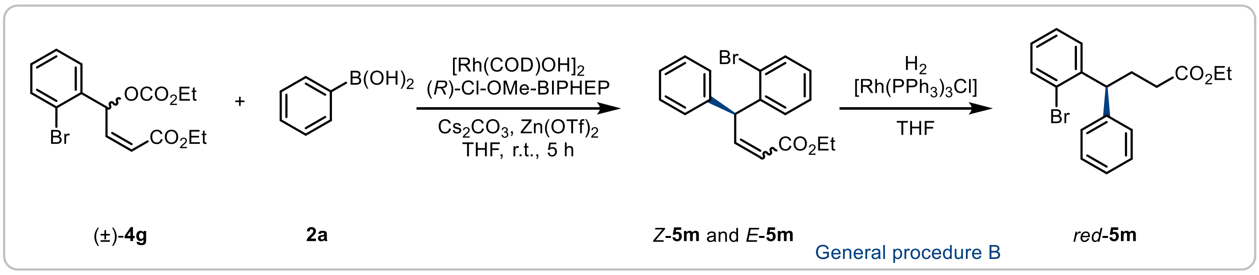

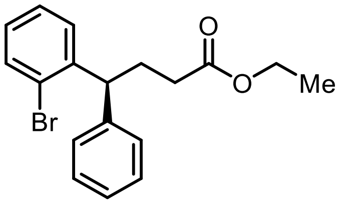

(-)-Ethyl-(*S*)-4-(2-bromophenyl)-4-phenylbutanoate *red*-5m

(-)-Ethyl-(*S*)-4-(2-bromophenyl)-4-phenylbutanoate *red*-**5m** was prepared using General procedure A with phenyl boronic acid **2a**, followed by reduction of the resulting crude mixture of products (Z:E= 1:1) using General procedure B. Purification by silica chromatography (Et_2O/hexane = 0/100 to 20/80) afforded (-)-Ethyl-(*S*)-4-(2-bromophenyl)-4-phenylbutanoate *red*-**5m** was (70.8 mg, 51%) as a colourless oil. SFC analysis showed an enantiomeric excess of >99%.

^{1}H NMR (400 MHz, $CDCl_3$) δ 7.56 (dd, J = 8.0, 1.3 Hz, 1H), 7.34 – 7.26 (m, 6H), 7.24 – 7.18 (m, 1H), 7.06 (ddd, J = 8.0, 7.0, 2.0 Hz, 1H), 4.58 – 4.47 (m, 1H), 4.12 (q, J = 7.1 Hz, 2H), 2.52 – 2.21 (m, 4H), 1.25 (t, J = 7.1 Hz, 3H).

^{13}C NMR (101 MHz, $CDCl_3$) δ 173.4, 143.27, 142.9, 133.2, 128.8, 128.6, 128.3, 128.0, 127.8, 126.6, 125.4, 60.5, 48.6, 32.7, 30.7, 14.4.

IR (CH_3Cl film) 2360, 2341, 1735, 1602, 1566, 1496, 1468, 1453, 1440, 1376, 1287, 1242, 1218, 1190, 1158, 1119, 1023 cm^{-1}.

HRMS (ESI): m/z calculated for $C_{18}H_{19}BrO_2Na^+$ $[M+Na]^+$ 369.0461 found 369.0458.

SFC: Chiralpak® IG, 1500 psi, 30 °C; flow: 1.0 mL/min; 1% to 10% MeOH over 5 min, 99.8:0.2 e.r. (minor enantiomer t_R = 4.72 min, major enantiomer t_R = 4.52 min).

α^{25}_D = -35.1 (c = 1.0, $CHCl_3$).

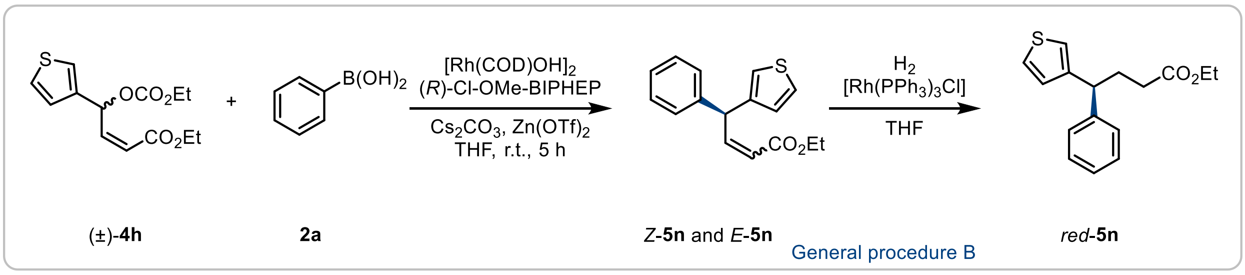

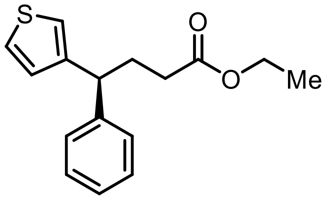

(+)-Ethyl-(*S*)-4-phenyl-4-(thiophen-3-yl)butanoate *red*-5n

(+)-Ethyl-(*S*)-4-phenyl-4-(thiophen-3-yl)butanoate *red*-**5n** was prepared using General procedure A with phenyl boronic acid **2a**, followed by reduction of the resulting crude mixture of products (Z:E= 1:1) using General procedure B. Purification by silica chromatography (Et_2O/hexane = 0/100 to 20/80) afforded (+)-Ethyl-(*S*)-4-phenyl-4-(thiophen-3-yl)butanoate *red*-**5n** (87.8 mg, 80%) as a colourless oil. SFC analysis showed an enantiomeric excess of >99%.

^{1}H NMR (400 MHz, $CDCl_3$) δ 7.35 – 7.27 (m, 2H), 7.25 – 7.17 (m, 4H), 7.02 (dt, J = 2.5, 1.1 Hz, 1H), 6.92 (dd, J = 5.0, 1.3 Hz, 1H), 4.11 (q, J = 7.1 Hz, 2H), 4.00 (t, J = 7.4 Hz, 1H), 2.50 – 2.34 (m, 1H), 2.34 – 2.18 (m, 3H), 1.24 (t, J = 7.1 Hz, 3H).

^{13}C NMR (101 MHz, $CDCl_3$) δ 173.5, 145.3, 143.9, 128.7, 128.0, 127.8, 126.6, 125.7, 120.5, 60.5, 46.3, 32.8, 31.2, 14.4.

IR (CH_3Cl film) 3028, 2984, 2937, 1733, 1602, 1493, 1453, 1375, 1320, 1255, 1181, 1161, 1082, 1032 cm^{-1}.

HRMS (ESI): m/z calculated for $C_{16}H_{18}O_2SNa^+$ $[M+Na]^+$ 297.0920 found 297.0912.

SFC: Chiralpak® IG, 1500 psi, 30 °C; flow: 1.0 mL/min; 1% to 10% MeOH over 5 min, 99.8:0.2 e.r. (minor enantiomer t_R = 5.47 min, major enantiomer t_R = 5.16 min).

α^{25}_D = +31.1 (c = 1.0, $CHCl_3$).

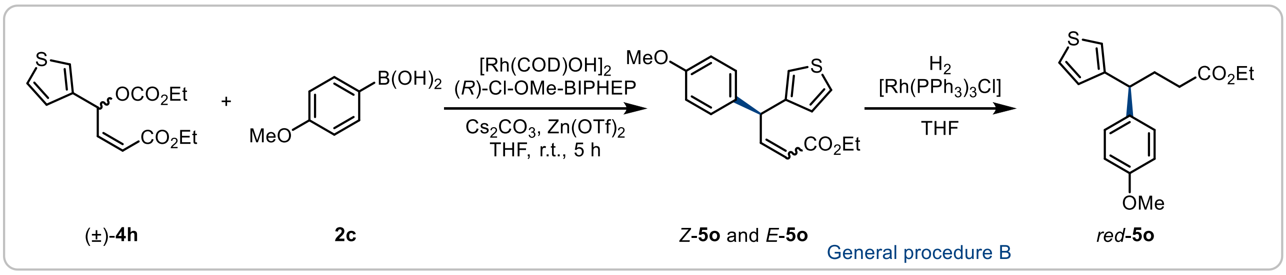

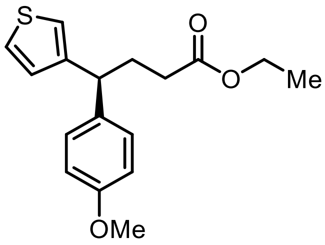

(+)-Ethyl-(*S*)-4-(4-methoxyphenyl)-4-(thiophen-3-yl)butanoate *red*-5o

(+)-Ethyl-(*S*)-4-(4-methoxyphenyl)-4-(thiophen-3-yl)butanoate *red*-**5o** was prepared using General procedure A with *p*-OMephenyl boronic acid **2c**, followed by reduction of the resulting crude mixture of products (Z:E= 1:1) using General procedure B. Purification by silica chromatography (Et_2O/hexane = 0/100 to 20/80) afforded (+)-Ethyl-(*S*)-4-(4-methoxyphenyl)-4-(thiophen-3-yl)butanoate *red*-**5o** (101.1 mg, 83%) as a colourless oil. SFC analysis showed an enantiomeric excess of 99%.

^{1}H NMR (400 MHz, $CDCl_3$) δ 7.23 (dd, J = 5.0, 2.9 Hz, 1H), 7.16 – 7.09 (m, 2H), 6.99 (ddd, J = 3.0, 1.4, 0.9 Hz, 1H), 6.92 – 6.86 (m, 1H), 6.86 – 6.81 (m, 2H), 4.10 (q, J = 7.1 Hz, 2H), 3.98 – 3.90 (m, 1H), 3.78 (s, 3H), 2.47 – 2.15 (m, 4H), 1.23 (t, J = 7.1 Hz, 3H).

^{13}C NMR (101 MHz, $CDCl_3$) δ 173.5, 158.3, 145.7, 135.9, 128.9, 127.7, 125.7, 120.2, 114.0, 60.4, 55.3, 45.5, 32.8, 31.3, 14.3.

IR (CH_3Cl film) 2980, 2957, 2937, 2837, 2357, 1733, 1611, 1584, 1513, 1464, 1375, 1304, 1250, 1180, 1160, 1037 cm^{-1}.

HRMS (ESI): m/z calculated for $C_{17}H_{20}O_3SNa^+$ $[M+Na]^+$ 327.1025 found 327.1017.

SFC: Chiralpak® IG, 1500 psi, 30 °C; flow: 1.0 mL/min; 1% to 10% MeOH over 5 min, 99.4:0.6 e.r. (minor enantiomer t_R = 6.34 min, major enantiomer t_R = 6.20 min).

α^{25}_D = +31.4 (c = 1.0, CHCl3).

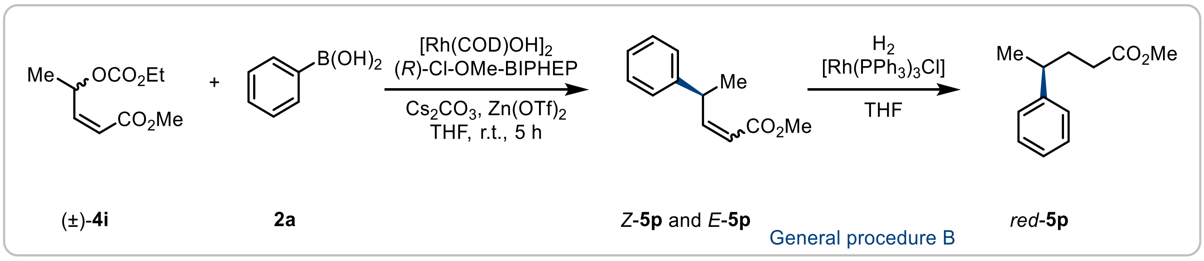

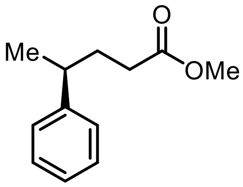

(+)-Methyl (*S*)-4-phenylpentanoate *red*-5p

(+)-Methyl (*S*)-4-phenylpentanoate *red*-**5p** was prepared using General procedure A with phenyl boronic acid **2a**, followed by reduction of the resulting crude mixture of products (Z:E= 3.1:1) using General procedure B. Purification by silica chromatography (Et_2O/hexane = 0/100 to 20/80) (+)-Methyl (*S*)-4-phenylpentanoate *red*-**5p** (68.7 mg, 89%) as a colourless oil. SFC analysis showed an enantiomeric excess of 96%.

^{1}H NMR (400 MHz, $CDCl_3$) δ 7.30 (ddt, *J* = 7.8, 6.6, 0.9 Hz, 2H), 7.23 – 7.13 (m, 3H), 3.63 (s, 3H), 2.72 (dq, *J* = 15.5, 6.9 Hz, 1H), 2.31 – 2.12 (m, 2H), 2.02 – 1.83 (m, 2H), 1.28 (d, *J* = 7.0 Hz, 3H).

^{13}C NMR (101 MHz, $CDCl_3$) δ 174.2, 146.3, 128.6, 127.1, 126.3, 51.6, 39.5, 33.3, 32.4, 22.3.

IR (CH_3Cl film): 3372, 3030, 2958, 2359, 1804, 1738, 1602, 1495, 1454, 1378, 1334, 1258, 1214, 1171, 1120, 1083 cm^{-1}.

HRMS (ESI): m/z calculated for $C_{12}H_{17}O_2^+$ $[M+H]^+$ 193.1223 found 193.1225.

SFC: Chiralpak® IF, 1500 psi, 30 °C; flow: 1.0 mL/min; 1% to 10% MeOH over 5 min, 98.2:1.8 e.r. (minor enantiomer t_R = 2.40 min, major enantiomer t_R = 2.27 min).

α^{25}_D = +17.1 (c = 1.00 in $CHCl_3$).

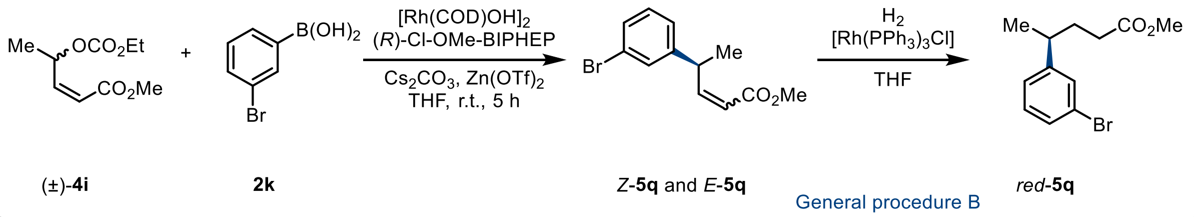

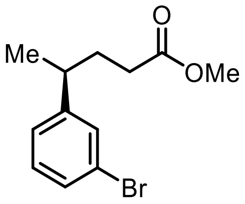

(+)-Methyl (*S*)-4-(3-bromophenyl)pentanoate *red*-5q

(+)-Methyl (*S*)-4-(3-bromophenyl)pentanoate *red*-**5q** was prepared using General procedure A with *m*-bromophenyl boronic acid **2k**, followed by reduction of the resulting crude mixture of products (Z:E= 4.3:1) using General procedure B. Purification by silica chromatography (Et_2O/hexane = 0/100 to 20/80) afforded (+)-Methyl (*S*)-4-(3-bromophenyl)pentanoate *red*-**5q** (102.0 mg, 94%) as a colourless oil. SFC analysis showed an enantiomeric excess of 94%.

^{1}H NMR (400 MHz, $CDCl_3$) δ 7.36 – 7.26 (m, 2H), 7.21 – 7.05 (m, 2H), 3.62 (s, 3H), 2.68 (dp, *J* = 8.7, 6.8 Hz, 1H), 2.28 – 2.10 (m, 2H), 1.99 – 1.79 (m, 2H), 1.24 (d, *J* = 7.0 Hz, 3H).

^{13}C NMR (101 MHz, $CDCl_3$) δ 173.9, 148.8, 130.2, 130.2, 129.4, 125.8, 122.7, 51.6, 39.3, 33.1, 32.2, 22.1.

IR (CH_3Cl film): 2957, 2874, 2349, 1739, 1595, 1568, 1478, 1455, 1436, 1327, 1276, 1256, 1207, 1169, 1074, 1026 cm^{-1}.

HRMS (ESI): m/z calculated for $C_{12}H_{15}{}^{79}BrO_2Na^+$ $[M+Na]^+$ 293.0148 found 293.0145.

SFC: Chiralpak® IF, 1500 psi, 30 °C; flow: 1.0 mL/min; 1% to 10% MeOH over 5 min, 96.9:3.1 e.r. (minor enantiomer t_R = 2.80 min, major enantiomer t_R = 3.00 min).

α^{25}_D = +23.0 (c = 1.00 in $CHCl_3$).

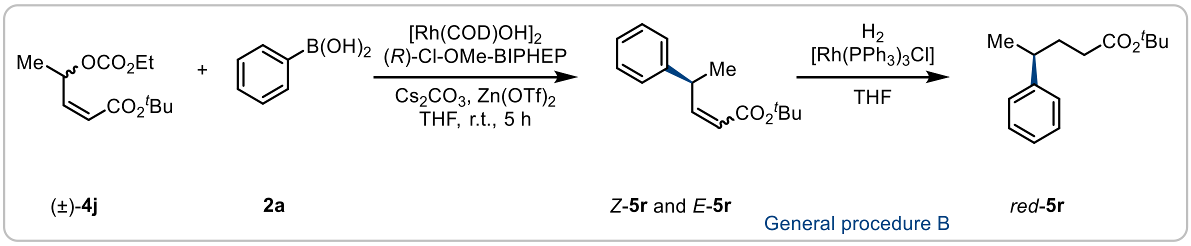

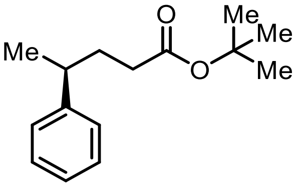

(+)-*Tert*-butyl-(*S*)-4-phenylpentanoate *red*-**5r**

(+)-*Tert*-butyl-(*S*)-4-phenylpentanoate *red*-**5r** was prepared using General procedure A with phenyl boronic acid **2a**, followed by reduction of the resulting crude mixture of products (Z:E= 2:1) using General procedure B. Purification by silica chromatography (Et_2O/hexane = 0/100 to 20/80) afforded (+)-Ethyl-(*S*)-4-(4-methoxyphenyl)-4-(thiophen-3-yl)butanoate *red*-**5r** (81.6 mg, 87%) as a colourless oil. SFC analysis showed an enantiomeric excess of 97%.

^{1}H NMR (400 MHz, $CDCl_3$) δ 7.33 – 7.27 (m, 2H), 7.24 – 7.14 (m, 3H), 2.71 (dp, J = 8.5, 6.8 Hz, 1H), 2.18 – 2.04 (m, 2H), 1.96 – 1.77 (m, 2H), 1.43 (s, 9H), 1.27 (d, J = 6.9 Hz, 3H).

^{13}C NMR (101 MHz, $CDCl_3$) δ 173.2, 146.7, 128.6, 127.2, 126.2, 80.1, 39.5, 33.9, 33.5, 28.2, 22.3.

IR (CH_3Cl film) 3084, 3064, 3029, 2978, 2933, 2874, 1731, 1604, 1495, 1455, 1393, 1368, 1327, 1258, 1152, 1045 cm^{-1}.

HRMS (ESI): m/z calculated for $C_{15}H_{22}O_2Na^+$ $[M+Na]^+$ 257.1512 found 257.1510.

SFC: Chiralpak® IG, 1500 psi, 30 °C; flow: 1.0 mL/min; 1% to 10% MeOH over 5 min, 98.5:1.5 e.r. (minor enantiomer t_R = 1.96 min, major enantiomer t_R = 2.03 min).

α^{25}_D = +15.0 (c = 1.0, $CHCl_3$).

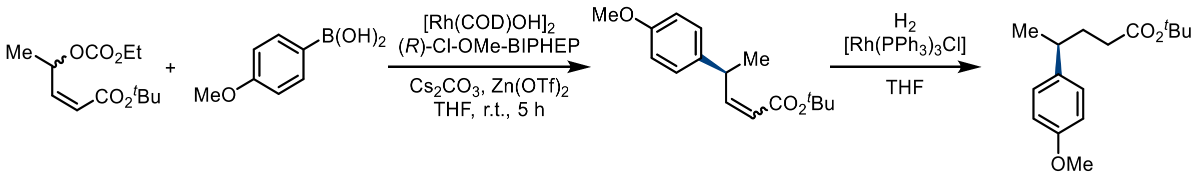

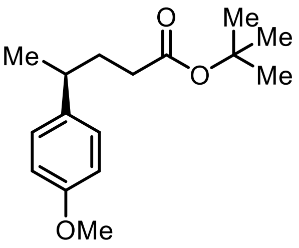

(+)-*Tert*-butyl (*S*)-4-(4-methoxyphenyl)pentanoate *red*-**5s**

(+)-*Tert*-butyl (*S*)-4-(4-methoxyphenyl)pentanoate *red*-**5s** was prepared using General procedure A with *p*-OMephenyl boronic acid **2c**, followed by reduction of the resulting crude mixture of products (Z:E= 10:1) using General procedure B. Purification by silica chromatography (Et_2O/hexane = 0/100 to 20/80) afforded (+)-*Tert*-butyl (*S*)-4-(4-methoxyphenyl)pentanoate *red*-**5s** was (74.0 mg, 70%) as a colourless oil. SFC analysis showed an enantiomeric excess of 96%.

^{1}H NMR (400 MHz, $CDCl_3$) δ 7.13 – 7.05 (m, 2H), 6.89 – 6.80 (m, 2H), 3.79 (s, 3H), 2.66 (dp, *J* = 8.9, 6.8 Hz, 1H), 2.09 (ddd, *J* = 8.2, 7.0, 1.4 Hz, 2H), 1.93 – 1.72 (m, 2H), 1.42 (s, 9H), 1.24 (d, *J* = 7.0 Hz, 3H).

^{13}C NMR (101 MHz, $CDCl_3$) δ 173.3, 158.0, 138.8, 128.0, 113.9, 80.1, 55.4, 38.7, 33.9, 33.7, 28.3, 22.5.

IR (CH_3Cl film) 2977, 2934, 2836, 1729, 1613, 1584, 1514, 1457, 1392, 1368, 1302, 1248, 1178, 1151, 1101, 1038 cm^{-1}.

HRMS (ESI): m/z calculated for $C_{16}H_{24}O_3Na^+$ 287.1618 found 287.1615

SFC: Chiralpak® IC, 1500 psi, 30 °C; flow: 0.6 mL/min; 1% to 5% MeOH over 8 min, 97.9:2.1 e.r. (minor enantiomer t_R = 5.28 min, major enantiomer t_R = 5.20 min).

3. Determination of the absolute stereochemistry

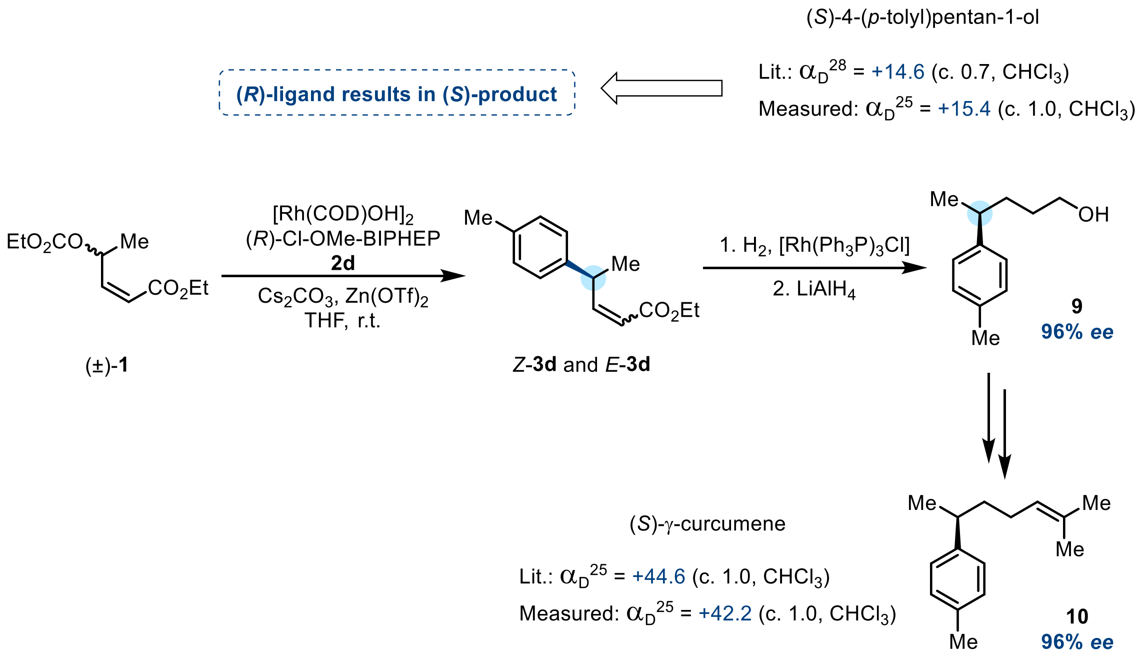

Figure 3.1 Determination of absolute stereochemistry by comparison of the optical rotation of 4-(*p*-tolyl)pentan-1-ol **9** with reported literature value[5]. Optical rotation value for curcumene **10** also matches literature reports.[6]

4. Procedures for the synthesis of starting materials

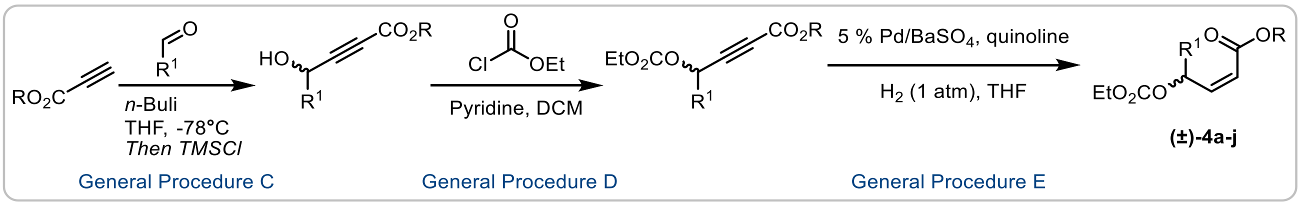

General procedure C:

Alkyne (36.0 mmol, 1.2 equiv.) was dissolved in THF (75 ml, 0.40 M). nBuLi (2.5 M in hexane, 14.4 ml, 36.0 mmol, 1.1 equiv.) was added dropwise at -78°C and the reaction mixture was stirred at -78°C for 30 minutes. Then aldehyde (30.0 mmol, 1.0 equiv.) was added dropwise, the reaction mixture stirred at -78°C for 2 hours. TMSCl (9.52 mL, 75.0 mmol, 2.5 equiv.) was added dropwise at -78°C and the mixture stirred for 1 hour. The mixture was warmed to 0°C and allowed to warm to room temperature over 2 hours. A saturated solution of NH_4Cl was added at 0°C and the aqueous layer was extracted with EtOAc (x3). Combined organic layers were washed with brine, dried over $MgSO_4$, filtered and concentrated *in vacuo*. Purification by flash chromatography (EtOAc/hexane) afforded the products. Alcohol products were fully characterized, unless already known, in which case ^{1}H NMR spectra were taken – in some examples known alcohols contained solvent or other small impurities but were used directly in the next step regardless.[7-10]

General procedure D:

Alcohol (1 equiv.) was dissolved in DCM (0.20 M) under argon and ethyl chloroformate (1.1 equiv.) and pyridine (1.2 equiv.) were added at 0°C. The reaction mixture was allowed to warm to room temperature and stirred for 16 hours. A 1 M aqueous $KHSO_4$ solution was added and the aqueous layer was extracted with DCM (x3). Combined organic layers were washed with saturated aqueous $NaHCO_3$ solution, brine, dried over $MgSO_4$, filtered and concentrated *in vacuo*. Purification by flash chromatography (EtOAc/Hexane) product.

General procedure E:

Rosenmund catalyst (5 % Pd on $BaSO_4$, 265 mg, 1.2 mmol, 0.15 equiv.) and quinoline (194 mg, 1.49 mmol, 0.18 equiv.) was suspended in THF (22 ml, 0.37 M) and the suspension was purged with hydrogen gas (balloon) for 10 minutes. Then a solution of alkyne in THF (2 ml) was added and the reaction mixture was stirred under an atmosphere of H_2 (balloon) for 3 hours

while being monitored. Upon consumption of starting material, the reaction was filtered through a plug of silica eluting with Et_2O and concentrated *in vacuo*. Purification by flash chromatography (Et_2O/hexane) afforded products.

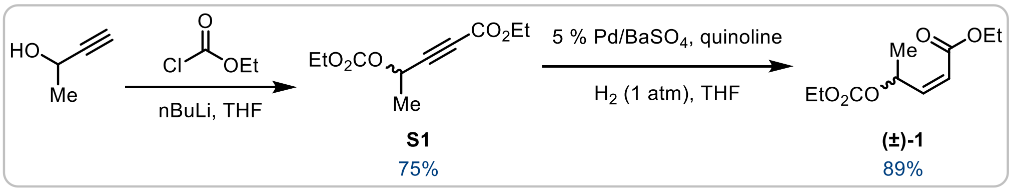

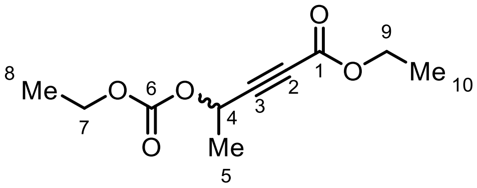

ethyl 4-((ethoxycarbonyl)oxy)pent-2-ynoate S1

nBuLi (2.5 M in hexane, 55.2 ml, 138.0 mmol, 2.3 eq) was added to a solution of but-3-yn-2-ol (4.70 ml, 60.0 mmol, 1.0 eq) in THF (150 ml) at -78°C. The mixture was stirred at -78°C for 30 minutes and ethyl ch3mate (12.6 ml, 132.0 mmol, 2.2 eq) was added dropwise. The reaction mixture was stirred at room temperature (23°C) for 6 hours. A saturated solution of NH_4Cl was added at 0°C and the aqueous layer was extracted with Et_2O (x3). Combined organic layers were washed with brine, dried over Na_2SO_4, filtered and concentrated *in vacuo*. Purification by flash chromatography (Et_2O/hexane) afforded ethyl 4-((ethoxycarbonyl)oxy)pent-2-ynoate **S1** (9.64 g, 45.0 mmol, 75%) as a colourless oil. Characterisation data match literature reports.[11]

^{1}H NMR (400 MHz, $CDCl_3$) δ 5.39 (q, *J* = 6.8 Hz, 1H, C(4)-H), 4.23 (*app* qd, *J* = 7.1, 2.1 Hz, 4H, C(7)-H_2 and C(9)-H_2), 1.59 (d, *J* = 6.9 Hz, 3H, C(5)-H_3), 1.33 (t, *J* = 7.0 Hz, 3H, C(8)-H_3), 1.29 (t, *J* = 7.0 Hz, 3H, C(10)-H_3).

^{13}C NMR (101 MHz, $CDCl_3$) δ 153.9 (C(1)), 152.9 (C(6)), 83.9 (C(3)), 76.9 (C(2)), 64.6 (C(5)), 63.1 (C(7)), 62.2 (C(9)), 20.4 (C(5)), 14.2 (C(8)), 13.9 (C(10)).

HRMS (ESI): m/z calculated for $C_{10}H_{14}O_5Na^+$ $[M+Na]^+$ 237.0733 found 237.0728.

IR (CH_3Cl film): 2979, 1751, 1716, 1468, 1372, 1007, 910, 880, 789, 732 cm^{-1}.

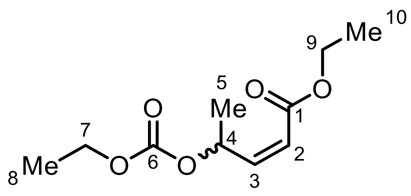

Ethyl (Z)-4-((ethoxycarbonyl)oxy)pent-2-enoate (±)-1

Rosenmund catalyst (Pd on $BaSO_4$, 303.6 mg, 2.9 mmol, 0.15 eq) and quinoline (0.41 ml, 3.5 mmol, 0.18 eq) were suspended in THF (50 ml, 0.37 M) and the suspension was purged with hydrogen gas (balloon) for 10 minutes. Then a solution of ethyl 4-((ethoxycarbonyl)oxy)pent-2-ynoate **S1** (4.07 g, 19.0 mmol, 1.0 eq) was added and the reaction mixture was stirred under an atmosphere of H_2 (balloon) for 3 hours while being monitored. Upon consumption of starting material, the reaction was filtered through a plug of silica eluting with Et_2O and concentrated *in vacuo*. Purification by automated medium-pressure flash chromatography (gradient of 0 to 15% Et_2O/hexane in 10 minutes) afforded ethyl (ethyl (Z)-4-((ethoxycarbonyl)oxy)pent-2-enoate **(±)-1** (3.66 g, 16.9 mmol, 89%) as a colourless oil.

^{1}H NMR (400 MHz, $CDCl_3$) δ 6.15 (m, 2H, C(3)-H and C(4)-H), 5.77 (m, 1H, C(2)-H), 4.15 (*app* qd, J = 7.1, 4.7 Hz, 4H, C(7)-H_2 and C(9)-H_2), 1.39 (d, J = 6.4 Hz, 3H, C(5)-H_3), 1.26 (*app* td, J = 7.1, 2.0 Hz, 6H, C(8)-H_3 and C(10)-H_3).

^{13}C NMR (101 MHz, $CDCl_3$) δ 165.2 (C(1)), 154.3 (C(6)), 147.7 (C(3)), 120.1 (C(2)), 72.0 (C(4)), 63.9 (C(7)), 60.4 (C(9)), 19.5 (C(5)), 14.2 (C(10)), 14.1 (C(8)).

HRMS (ESI): m/z calculated for $C_{10}H_{16}O_5Na^+$ $[M+Na]^+$ 239.0890 found 239.0890.

IR (CH_3Cl film): 2981 (m), 1746 (s), 1719 (s), 1654 (w), 1449 (w), 1371 (m), 1258 (s), 1195 (s), 1124 (m), 1042 (s), 1008 (m), 849 (m), 823 (m), 792 (m) cm^{-1}.

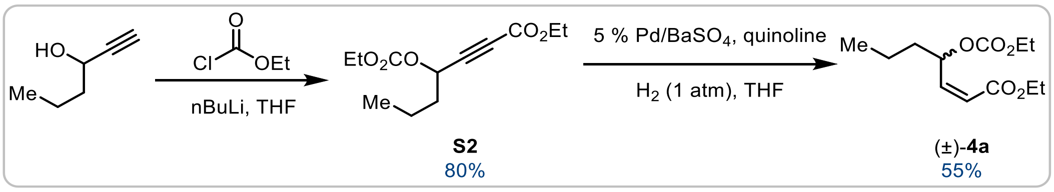

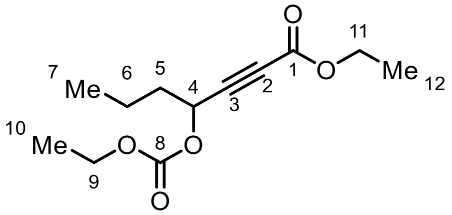

ethyl 4-((ethoxycarbonyl)oxy)hept-2-ynoate S2

nBuLi (2.5 M in hexane, 18.4 mL, 46.0 mmol, 2.3 equiv.) was added to a solution of hex-1-yn-3-ol (2.19 mL, 20.0 mmol, 1.0 equiv.) in THF (50 mL) at -78°C. The mixture was stirred at -78°C for 30 minutes and ethyl chloroformate (4.19 mL, 44.0 mmol, 2.2 equiv.) was added dropwise. The reaction mixture was stirred at room temperature (23°C) for 6 hours. A saturated solution of NH_4Cl was added at 0°C and the aqueous layer was extracted with Et_2O (x3). Combined organic layers were washed with brine, dried over Na_2SO_4, filtered and concentrated *in vacuo*. Purification by flash chromatography (Et_2O/hexane) ethyl 4-((ethoxycarbonyl)oxy)hept-2-ynoate **S2** (3.88 g, 16.0 mmol, 80%) as a yellow oil.

^{1}H NMR (400 MHz, $CDCl_3$) δ 5.30 (t, *J* = 6.7 Hz, 1H, C(4)-H), 4.21 (*app* qd, *J* = 7.1, 1.3 Hz, 4H, C(9)-H_2 and C(11)-H_2), 1.83 (m, 2H, C(5)-H_2), 1.49 (h, *J* = 7.5 Hz, 2H, C(6)-H_2), 1.30 (*app* q, *J* = 7.2 Hz, 6H, C(10)-H_3 and C(12)-H_3), 0.94 (t, *J* = 7.4 Hz, 3H, C(7)-H_3).

^{13}C NMR (101 MHz, $CDCl_3$) δ 154.1 (C(1)), 153.0 (C(8)), 83.4 (C(3)), 77.5 (C(2)), 66.7 (C(4)), 64.6 (C(9)), 62.2 (C(11)), 36.0 (C(5)), 18.1 (C(6)), 14.2 (C(10)), 13.9 (C(12)), 13.4 (C(7)).

HRMS (ESI): m/z calculated for $C_{12}H_{18}O_5Na^+$ $[M+Na]^+$ 265.1046 found 265.1040.

IR (CH_3Cl film): 2965 (w), 1751 (m), 1717 (m), 1467 (w), 1372 (w), 1242 (s), 1074 (w), 1008 (m), 951 (w), 913 (w), 878 (w), 790 (w), 733 (m) cm^{-1}.

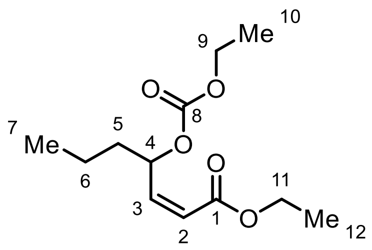

ethyl (*Z*)-4-((ethoxycarbonyl)oxy)hept-2-enoate (±)-4a

Rosenmund catalyst (Pd on $BaSO_4$, 255.5 mg, 2.4 mmol, 0.15 equiv.) and quinoline (0.34 mL, 2.9 mmol, 0.18 equiv.) were suspended in THF (40 mL, 0.37 M) and the suspension was purged with hydrogen gas (balloon) for 10 minutes. Then a solution of ethyl 4-((ethoxycarbonyl)oxy)hept-2-ynoate **S2** (3.88 g, 16.0 mmol, 1.0 equiv.) in THF (2 mL) was added and the reaction mixture was stirred under an atmosphere of H_2 (balloon) for 3 hours while being monitored. Upon consumption of starting material, the reaction was filtered through a plug of silica eluting with E_2O and concentrated *in vacuo*. Purification by automated medium-pressure flash chromatography (gradient of 0 to 20% Et_2O/hexane in 10 minutes) afforded ethyl (*Z*)-4-((ethoxycarbonyl)oxy)hept-2-enoate (±)-**4a** (2.15 g, 8.80 mmol, 55%) as a colourless oil.

^{1}H NMR (400 MHz, $CDCl_3$) δ 6.12 (m, 2H, C(3)-H and C(4)-H), 5.83 (m, 1H, C(2)-H), 4.18 (*app* p, *J* = 7.1 Hz, 4H, C(9)-H_2 and C(11)-H_2), 1.75 (m, 1H, C(5)-H), 1.63 (m, 1H, C(5)-H), 1.44 (m, 2H, C(6)-H_2), 1.29 (td, *J* = 7.1, 1.3 Hz, 6H, C(10)-H_3 and C(12)-H_3), 0.93 (t, *J* = 7.3 Hz, 3H, C(7)-H_3).

^{13}C NMR (101 MHz, $CDCl_3$) δ 165.3 (C(1)), 154.7 (C(8)), 146.9 (C(3)), 120.7 (C(2)), 75.1 (C(4)), 63.9 (C(9)), 60.4 (C(11)), 36.0 (C(5)), 18.3 (C(6)), 14.2 (C(10) and C(12)), 13.8 (C(7)).

HRMS (ESI): m/z calculated for $C_{12}H_{20}O_5Na^+$ $[M+Na]^+$ 267.1203 found 267.1202.

IR (CH_3Cl film): 2964 (w), 1746 (m), 1719 (m), 1372 (w), 1257 (s), 1192 (m), 1010 (w), 954 (w), 910 (m), 825 (w), 750 (s), 649 (w) cm^{-1}.

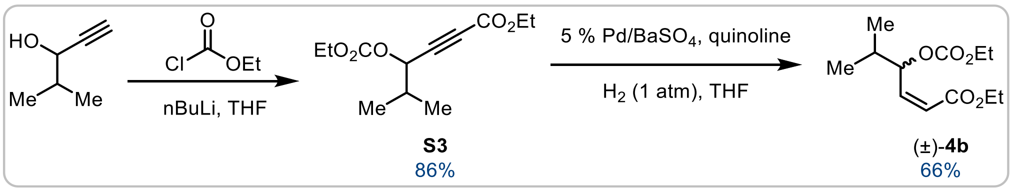

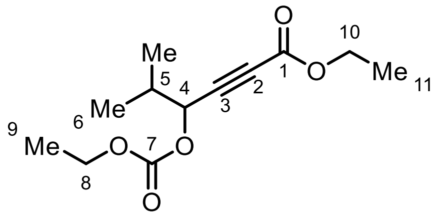

ethyl 4-((ethoxycarbonyl)oxy)-5-methylhex-2-ynoate S3

nBuLi (2.5 M in hexane, 9.2 mL, 23.0 mmol, 2.3 equiv.) was added to a solution of 4-methylpent-1-yn-3-ol (1.00 g, 10.0 mmol, 1.0 equiv.) in THF (25 mL) at -78°C. The mixture was stirred at -78°C for 30 minutes and ethyl chloroformate (2.1 mL, 22.0 mmol, 2.2 equiv.) was added dropwise. The reaction mixture was stirred at room temperature (23°C) for 7 hours. A saturated solution of NH_4Cl was added at 0°C and the aqueous layer was extracted with Et_2O (x3). Combined organic layers were washed with brine, dried over Na_2SO_4, filtered and concentrated *in vacuo*. Purification by flash chromatography (Et_2O/hexane) ethyl 4-((ethoxycarbonyl)oxy)-5-methylhex-2-ynoate **S3** (2.09 g, 8.62 mmol, 86%) as a colourless oil.

^{1}H NMR (400 MHz, $CDCl_3$) δ 5.14 (d, *J* = 6.3 Hz, 1H, C(4)-H), 4.22 (q, *J* = 7.2 Hz, 4H, C(8)-H_2 and C(10)-H_2), 2.11 (dh, *J* = 7.0, 6.5 Hz, 1H, C(5)-H), 1.30 (*app* q, *J* = 7.6, Hz, 6H, C(9)-H_3 and C(11)-H_3), 1.05 (m, 6H, C(6)-H_3 x2).

^{13}C NMR (101 MHz, $CDCl_3$) δ 154.3 (C(1)), 153.0 (C(7)), 82.4 (C(3)), 78.2 (C(2)), 71.9 (C(4)), 64.6 (C(8)), 62.2 (C(10)), 32.4 (C(5)), 18.0 (C(6)), 17.6 (C(6)), 14.2 (C(9)), 14.0 (C(11)).

HRMS (ESI): m/z calculated for $C_{12}H_{18}O_5Na^+$ $[M+Na]^+$ 265.1046 found 265.1048.

IR (CH_3Cl film): 2979 (w), 1751 (m), 1716 (m), 1468 (w), 1372 (w), 1241 (s), 1007 (m), 910 (m), 880 (w), 789 (w), 732 (m) cm^{-1}.

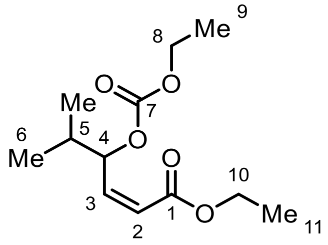

ethyl (Z)-4-((ethoxycarbonyl)oxy)-5-methylhex-2-enoate (±)-4b

Rosenmund catalyst (Pd on $BaSO_4$, 69.0 mg, 0.60 mmol, 0.15 equiv.) and quinoline (0.092 mL, 0.80 mmol, 0.18 equiv.) were suspended in THF (12 mL, 0.37 M) and the suspension was purged with hydrogen gas (balloon) for 10 minutes. Then a solution of ethyl 4-((ethoxycarbonyl)oxy)-5-methylhex-2-ynoate **S3** (1.05 g, 4.32 mmol, 1.0 equiv.) in THF (2 mL) was added and the reaction mixture was stirred under an atmosphere of H_2 (balloon) for 3 hours while being monitored. Upon consumption of starting material, the reaction was filtered through a plug of silica eluting with Et_2O and concentrated *in vacuo*. Purification by automated medium-pressure flash chromatography (gradient of 0 to 20% Et_2O/hexane in 10 minutes) afforded ethyl (Z)-4-((ethoxycarbonyl)oxy)-5-methylhex-2-enoate (±)-**4b** (0.795 g, 2.9 mmol, 66%) as a colourless oil.

^{1}H NMR (400 MHz, $CDCl_3$) δ 6.09 (dd, *J* = 11.6, 8.4 Hz, 1H, C(3)-H), 6.02 (ddd, *J* = 8.4, 5.3, 1.0 Hz, 1H, C(4)-H), 5.91 (dd, *J* = 11.6, 1.0 Hz, 1H, C(2)-H), 4.18 (m, 4H, C(8)-H_2 and C(10)-H_2), 2.04 (heptd, *J* = 6.9, 5.2 Hz, 1H, C(5)-H), 1.29 (*app* td, *J* = 7.2, 0.6 Hz, 6H, C(9)-H_3 and C(11)-H_3), 0.98 (*app* dd, *J* = 6.9, 1.1 Hz, 6H, C(6)-H_3 x2).

^{13}C NMR (101 MHz, $CDCl_3$) δ 165.3 (C(1)), 154.8 (C(7)), 145.3 (C(3)), 122.0 (C(2)), 78.6 (C(4)), 63.9 (C(8)), 60.4 (C(10)), 32.4 (C(5)), 18.3 (C(9)), 17.2 (C(11)), 14.2 (C(6)), 14.1 (C(6)).

HRMS (ESI): m/z calculated for $C_{12}H_{20}O_5Na^+$ $[M+Na]^+$ 267.1203 found 267.1206.

IR (CH_3Cl film): 2981 (w), 1745 (w), 1372 (w), 1258 (m), 1196 (m), 1008 (w), 945 (w), 908 (s), 650 (w) cm^{-1}.

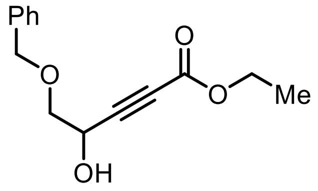

Ethyl 5-(benzyloxy)-4-hydroxypent-2-ynoate S4 contaminated with 2-(benzyloxy)acetaldehyde

From 2-(benzyloxy)acetaldehyde (3.70 g, 30 mmol) following General Procedure C. Flash column chromatography using silica gel (10-40% EtOAc: Hexane) gave ethyl 5-(benzyloxy)-4-hydroxypent-2-ynoate as a yellow oil which was contaminated with ~30% of the 2-(benzyloxy)acetaldehyde starting material, and was used directly in the next step without further purification (calculated yield 4.62 g, 18.6 mmol, 62%).

^{1}H NMR (400 MHz, $CDCl_3$) δ 7.43 – 7.28 (m, 5H), 4.64 – 4.61 (m, 2H), 4.24 (q, J = 7.1 Hz, 2H), 4.10 (d, J = 0.9 Hz, 1H), 3.77 – 3.67 (m, 1H), 3.63 (dd, J = 9.8, 6.4 Hz, 1H), 2.63 (s, 1H), 1.31 (t, J = 7.1 Hz, 3H).

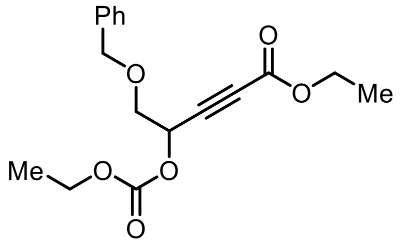

Ethyl 5-(benzyloxy)-4-((ethoxycarbonyl)oxy)pent-2-ynoate S5

From ethyl 5-(benzyloxy)-4-hydroxypent-2-ynoate (4.62 g, 18.6 mmol, 1.0 equiv.) according to General Procedure D. Purification by silica chromatography (10% EtOAc: Hexane) afforded ethyl 5-(benzyloxy)-4-((ethoxycarbonyl)oxy)pent-2-ynoate (3.49 g, 10.9 mmol, 59%) as a pale yellow oil.

^{1}H NMR (400 MHz, $CDCl_3$) δ 7.39 – 7.27 (m, 5H), 5.54 (dd, J = 6.9, 4.6 Hz, 1H), 4.61 (s, 2H), 4.24 (qdd, J = 7.2, 4.1, 2.9 Hz, 4H), 3.91 – 3.66 (m, 2H), 1.39 – 1.25 (m, 6H).

^{13}C NMR (101 MHz, $CDCl_3$) δ 154.2, 152.8, 137.4, 128.6, 128.1, 127.9, 80.7, 78.4, 73.6, 70.2, 66.17, 65.0, 62.5, 14.3, 14.1.

IR (CH_3Cl film): 2986, 2871, 2362, 2249, 1753, 1716, 1497, 1455, 1395, 1371, 1339, 1237, 1118, 1095, 1010 cm^{-1}.

HRMS (ESI): m/z calculated for $C_{17}H_{20}O_6Na^+$ $[M+Na]^+$ 343.1152 found 343.1142.

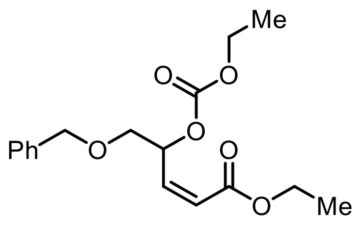

Ethyl (*Z*)-5-(benzyloxy)-4-((ethoxycarbonyl)oxy)pent-2-enoate (±)-4c

From ethyl 5-(benzyloxy)-4-((ethoxycarbonyl)oxy)pent-2-ynoate (2.66 g, 8.3 mmol, 1.0 equiv.) according to General Procedure E. Purification by silica chromatography (gradient of 0 to 30% Et_2O/hexane) afforded ethyl (*Z*)-5-(benzyloxy)-4-((ethoxycarbonyl)oxy)pent-2-enoate (2.05 g, 6.36 mmol, 77%) as a colourless oil.

^{1}H NMR (400 MHz, $CDCl_3$) δ 7.36 – 7.24 (m, 5H), 6.38 (dddd, J = 7.6, 5.0, 4.2, 1.5 Hz, 1H), 6.21 (dd, J = 11.7, 7.5 Hz, 1H), 5.91 (dd, J = 11.7, 1.5 Hz, 1H), 4.66 (d, J = 12.1 Hz, 1H), 4.56 (d, J = 12.1 Hz, 1H), 4.19 (qd, J = 7.1, 3.5 Hz, 4H), 3.80 – 3.68 (m, 2H), 1.29 (dt, J = 7.9, 7.1 Hz, 6H).

^{13}C NMR (101 MHz, $CDCl_3$) δ 165.3, 154.7, 143.9, 138.1, 128.4, 127.8, 127.7, 122.2, 74.4, 73.1, 70.9, 64.3, 60.7, 14.3, 14.3.

IR (CH_3Cl film) 3032, 2986, 2907, 2869, 2360, 2341, 1747, 1718, 1684, 1653, 1577, 1558, 1540, 1507, 1497, 1455, 1416, 1389, 1372, 1326, 1261, 1195, 1094, 1027 cm^{-1}.

HRMS (ESI): m/z calculated for $C_{17}H_{22}O_6Na^+$ $[M+Na]^+$ 345.1309 found 345.1300.

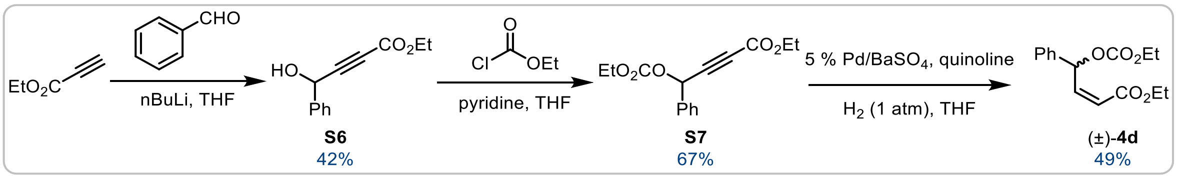

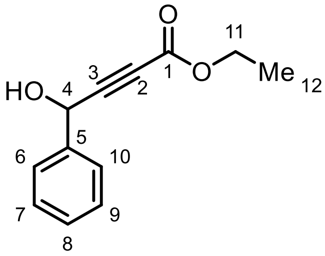

ethyl 4-hydroxy-4-phenylbut-2-ynoate S6

Ethyl propiolate (3.04 mL, 30.0 mmol, 1.0 equiv.) was dissolved in THF (75 mL, 0.40 M). nBuLi (2.5 M in hexane, 13.20 mL, 33.0 mmol, 1.1 equiv.) was added dropwise at -78°C and the reaction mixture was stirred at -78°C for 30 minutes. Then, benzaldehyde (3.35 mL, 33.0 mmol, 1.1 equiv.) was added dropwise, the reaction mixture was warmed to room temperature and stirred for 6 hours. A saturated solution of NH_4Cl was added at 0°C and the aqueous layer was extracted with EtOAc (x3). Combined organic layers were washed with brine, dried over Na_2SO_4, filtered and concentrated *in vacuo*. Purification by flash chromatography (EtOAc/hexane) afforded ethyl 4-hydroxy-4-phenylbut-2-ynoate **S5** (2.55 g, 12.5 mmol, 42%) as a yellow oil. Characterisation data match literature reports.[12]

^{1}H NMR (400 MHz, $CDCl_3$) δ 7.51 (m, 2H, C(7)-H and C(9)-H), 7.38 (m, 3H, C(6)-H, , C(8)-H and , C(10)-H), 5.55 (d, *J* = 5.5 Hz, 1H, C(4)-H), 4.24 (q, *J* = 7.1 Hz, 2H, C(11)-H_2), 2.92 (d, *J* = 6.0 Hz, 1H, -OH), 1.30 (t, *J* = 7.1 Hz, 3H, C(12)-H_3).

^{13}C NMR (101 MHz, $CDCl_3$) δ 153.4 (C(1)), 138.6 (C(5)), 128.9 (C(8)), 128.8 (C(7) and C(9)), 126.7 (C(6) and C(10)), 86.2 (C(3)), 77.9 (C(2)), 64.3 (C(4)), 62.3 (C(11)), 14.0 (C(12)).

HRMS (ESI): m/z calculated for $C_{12}H_{12}O_3Na^+$ $[M+Na]^+$ 227.0679 found 227.0678.

IR (CH_3Cl film): 3412 (m), 2990 (w), 2239 (m), 1713 (s), 1455 (m), 1369 (m), 1018 (s), 752 (s), 699 (s) cm^{-1}.

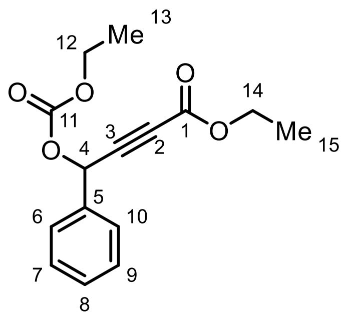

ethyl 4-((ethoxycarbonyl)oxy)-4-phenylbut-2-ynoate S7

Following a modified procedure by Lindner[13], ethyl 4-hydroxy-4-phenylbut-2-ynoate **S5** (2.55 g, 12.5 mmol, 1.0 equiv.) and ethyl chloroformate (1.31 mL, 13.7 mmol, 1.1 equiv.) were dissolved in DCM (62 mL, 0.20 M). To this mixture, pyridine (1.21 mL, 15.0 mmol, 1.2 equiv.) was added at 0°C. The reaction mixture was allowed to warm to room temperature and stirred for 3 hours. A 1 M aqueous $KHSO_4$ solution was added and the aqueous layer was extracted with DCM (x3). Combined organic layers were washed with saturated aqueous $NaHCO_3$ solution, brine, dried over Na_2SO_4, filtered and concentrated *in vacuo*. Purification by flash chromatography (Et_2O/hexane) afforded ethyl 4-((ethoxycarbonyl)oxy)-4-phenylbut-2-ynoate **S6** (2.00g, 8.3 mmol, 67%) as a colourless oil.

^{1}H NMR (400 MHz, $CDCl_3$) δ 7.52 (m, 2H, C(7)-H and C(9)-H), 7.39 (m, 3H, C(6)-H, C(8)-H and C(10)-H), 6.37 (s, 1H, C(4)-H), 4.23 (m, 4H, C(12)-H_2 and C(14)-H_2), 1.30 (*app* td, J = 7.1, 3.4 Hz, 6H, C(13)-H_3 and C(15)-H_3).

^{13}C NMR (101 MHz, $CDCl_3$) δ 153.9 (C(1)), 152.9 (C(11)), 134.6 (C(5)), 129.7 (C(8)), 128.9 (C(7) and C(9)), 127.8 (C(6) and C(10)), 82.1 (C(3)), 79.0 (C(2)), 68.5 (C(4)), 64.9 (C(12)), 62.3 (C(14)), 14.2 (C(13)), 14.0 (C(15)).

HRMS (ESI): m/z calculated for $C_{15}H_{16}O_5Na^+$ $[M+Na]^+$ 299.0890 found 299.0891.

IR (CH_3Cl film): 1750 (m), 1713 (m), 1371 (w), 1239 (m), 1006 (w), 908 (s), 730 (s), 697 (w), 650 (w) cm^{-1}.

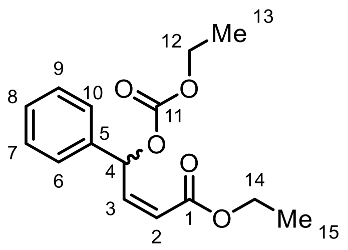

ethyl (Z)-4-((ethoxycarbonyl)oxy)-4-phenylbut-2-enoate (±)-4d

Rosenmund catalyst (Pd on $BaSO_4$, 132.4 mg, 1.2 mmol, 0.15 equiv.) and quinoline (0.17 mL, 1.4 mmol, 0.18 equiv.) were suspended in THF (22 mL, 0.37 M) and the suspension was purged with hydrogen gas (balloon) for 10 minutes. Then a solution of ethyl 4-((ethoxycarbonyl)oxy)-4-phenylbut-2-ynoate **S6** (2.29 g, 8.3 mmol, 1.0 equiv.) in THF (2 mL) was added and the reaction mixture was stirred under an atmosphere of H_2 (balloon) for 3 hours while being monitored. Upon consumption of starting material, the reaction was filtered through a plug of silica eluting with Et_2O and concentrated *in vacuo*. Purification by automated medium-pressure flash chromatography (gradient of 0 to 15% Et_2O/hexane in 10 minutes) afforded ethyl (Z)-4-((ethoxycarbonyl)oxy)-4-phenylbut-2-enoate (±)-**4d** (1.12g, 4.0mmol, 49%) as a colourless oil.

^{1}H NMR (400 MHz, $CDCl_3$) δ 7.51 (m, 2H, C(7)-H and C(9)-H), 7.33 (m, 4H, C(4)-H, C(6)-H, C(8)-H and C(10)-H), 6.39 (dd, J = 11.5, 8.7 Hz, 1H, C(3)-H), 5.90 (dd, J = 11.6, 1.2 Hz, 1H, C(2)-H), 4.20 (m, 4H, C(12)-H_2 and C(14)-H_2), 1.29 (*app* td, J = 7.1, 2.2 Hz, 6H, C(13)-H_3 and C(15)-H_3).

^{13}C NMR (101 MHz, $CDCl_3$) δ 165.3, 154.1, 144.4, 138.2, 128.7, 128.5, 127.1, 120.9, 75.2, 64.2, 60.6, 14.2, 14.2.

HRMS (ESI): m/z calculated for $C_{15}H_{18}O_5Na^+$ $[M+Na]^+$ 301.1046 found 301.1047.

IR (CH_3Cl film): 1748 (m), 1718 (m), 1653 (w), 1455 (w), 1371 (w), 1254 (s), 1199 (m), 1005 (w), 909 (s), 790 (w), 731 (s), 701 (m), 650 (w) cm^{-1}.

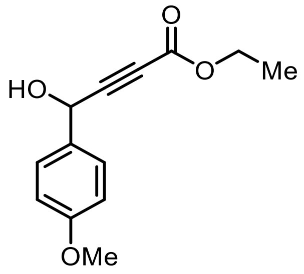

Ethyl 4-hydroxy-4-(4-methoxyphenyl)but-2-ynoate S8

From *p*-anisaldehyde (4.08 g, 30 mmol) following General Procedure C. Purification by silica chromatography (10-40% EtOAc: Hexane) gave ethyl 4-hydroxy-4-(4-methoxyphenyl)but-2-ynoate (5.15 g, 21.9 mmol, 73%) as a yellow oil. Characterisation data match the literature reports.[8]

^{1}H NMR (400 MHz, $CDCl_3$): δ 7.45 (d, J = 8.9 Hz, 2H), 6.92 (d, J = 8.9 Hz, 2H), 5.52 (d, J = 5.6 Hz, 1H), 4.25 (q, J = 7.1 Hz, 2H), 3.82 (s, 3H), 2.29 (d, J = 6.0 Hz, 1H), 1.32 (t, J = 7.2 Hz, 3H).

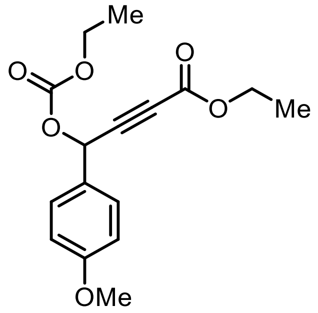

Ethyl 4-((ethoxycarbonyl)oxy)-4-(4-methoxyphenyl)but-2-ynoate S9

From ethyl 4-hydroxy-4-(4-methoxyphenyl)but-2-ynoate (5.15 g, 21.9 mmol) following General Procedure D. Purification by silica chromatography (10% EtOAc: Hexane) afforded ethyl 4-((ethoxycarbonyl)oxy)-4-(4-methoxyphenyl)but-2-ynoate (4.31 g, 14.1 mmol, 64%) as a pale yellow oil.

^{1}H NMR (400 MHz, $CDCl_3$) δ 7.50 – 7.41 (m, 2H), 6.95 – 6.87 (m, 2H), 6.32 (s, 1H), 4.23 (dq, J = 11.3, 7.1 Hz, 4H), 3.82 (s, 3H), 1.31 (td, J = 7.1, 0.9 Hz, 6H).

^{13}C NMR (126 MHz, $CDCl_3$) δ 160.8, 154.1, 153.1, 129.7, 126.9, 114.4, 82.5, 79.0, 68.5, 64.9, 62.5, 55.5, 14.3, 14.1.

IR (CH_3Cl film): 3018, 2940, 2841, 2360, 2246, 1751, 1716, 1613, 1515, 1469, 1379, 1235, 1177, 1114, 1007 cm^{-1}

HRMS (ESI): m/z calculated for $C_{16}H_{18}O_6Na^+$ $[M+Na]^+$ 329.0996 found 329.1007.

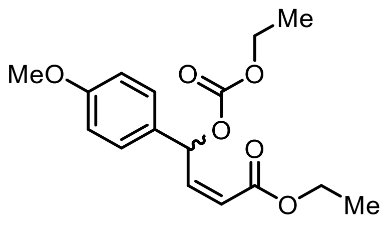

Ethyl (*Z*)-4-((ethoxycarbonyl)oxy)-4-(4-methoxyphenyl)but-2-enoate (±)-4e

From ethyl 4-((ethoxycarbonyl)oxy)-4-(4-methoxyphenyl)but-2-ynoate following General Procedure E. Purification by silica chromatography (gradient of 0 to 20% Et_2O/hexane) afforded ethyl (Z)-4-((ethoxycarbonyl)oxy)-4-(4-methoxyphenyl)but-2-enoate (1.71 g, 69%) as a colourless oil.

^{1}H NMR (400 MHz, $CDCl_3$) δ 7.47 – 7.39 (m, 2H), 7.20 (dd, *J* = 8.6, 1.3 Hz, 1H), 6.93 – 6.84 (m, 2H), 6.42 (dd, *J* = 11.6, 8.6 Hz, 1H), 5.86 (dd, *J* = 11.6, 1.3 Hz, 1H), 4.26 – 4.09 (m, 4H), 3.79 (s, 3H), 1.28 (td, *J* = 7.1, 2.1 Hz, 6H).

^{13}C NMR (101 MHz, $CDCl_3$) δ 165.4, 159.9, 154.3, 144.8, 130.5, 128.8, 120.5, 114.2, 75.2, 64.2, 60.7, 55.4, 14.3, 14.3.

IR (CH_3Cl film): 2985, 2839, 1748, 1719, 1652, 1612, 1587, 1515, 1466, 1412, 1389, 1371, 1306, 1248, 1190, 1176, 1032, 1006 cm^{-1}.

HRMS (ESI): m/z calculated for $C_{32}H_{40}O_{12}Na^+$ $[2M+Na]^+$ 639.2412 found 639.2409.

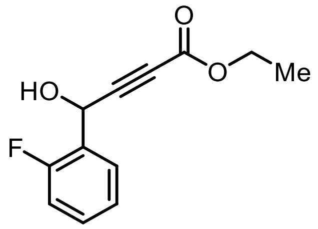

Ethyl 4-(2-fluorophenyl)-4-hydroxybut-2-ynoate S10

From 2-fluorobenzaldehyde (3.16 mL, 30 mmol) following General Procedure C. Purification by silica chromatography (10-40% EtOAc: Hexane) gave ethyl 4-(2-fluorophenyl)-4-hydroxybut-2-ynoate (5.13 g, 23.1 mmol, 77%) as a pale-yellow oil. Characterisation data match the literature reports.[7]

^{1}H NMR (400 MHz, $CDCl_3$) δ 7.61 (td, J = 7.6, 1.8 Hz, 1H), 7.41 – 7.31 (m, 1H), 7.20 (td, J = 7.6, 1.2 Hz, 1H), 7.14 – 7.05 (m, 1H), 5.84 (s, 1H), 4.25 (q, J = 7.1 Hz, 2H), 2.51 (s, 1H), 1.31 (t, J = 7.1 Hz, 3H).

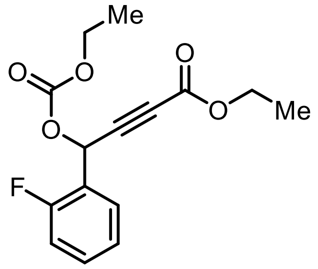

Ethyl 4-((ethoxycarbonyl)oxy)-4-(2-fluorophenyl)but-2-ynoate S11

From ethyl 4-(2-fluorophenyl)-4-hydroxybut-2-ynoate (5.13 g, 23.1 mmol, 1.0 equiv) following General Procedure D. Purification by silica chromatography (10% EtOAc: Hexane) gave ethyl 4-((ethoxycarbonyl)oxy)-4-(2-fluorophenyl)but-2-ynoate (4.59 g, 14.5 mmol, 63%) as a pale yellow oil.

^{1}H NMR (400 MHz, $CDCl_3$) δ 7.62 (td, J = 7.5, 1.8 Hz, 1H), 7.39 (dddd, J = 8.3, 7.2, 5.3, 1.8 Hz, 1H), 7.20 (td, J = 7.6, 1.2 Hz, 1H), 7.09 (ddd, J = 9.7, 8.3, 1.2 Hz, 1H), 6.65 (s, 1H), 4.32 – 4.16 (m, 4H), 1.30 (td, J = 7.2, 4.5 Hz, 6H).

^{19}F NMR (376 MHz, $CDCl_3$) δ -117.09 (ddd, J = 10.1, 7.3, 5.3 Hz).

^{13}C NMR (101 MHz, $CDCl_3$) δ 160.1 (d, J = 251.2 Hz), 153.8, 152.9, 131.8 (d, J = 8.4 Hz), 129.7 (d, J = 2.4 Hz), 124.7 (d, J = 3.7 Hz), 122.1 (d, J = 13.3 Hz), 116.0 (d, J = 20.8 Hz), 81.2, 78.9, 65.1, 62.7 (d, J = 5.2 Hz), 62.5, 14.3, 14.

IR (CH_3Cl film): 2988, 2249, 1755, 1718, 1618, 1592, 1494, 1461, 1371, 1244, 1177, 1100, 1007 cm^{-1}

HRMS (ESI): m/z calculated for $C_{15}H_{15}FO_5Na^+$ $[M+Na]^+$ 317.0796 found 317.0789.

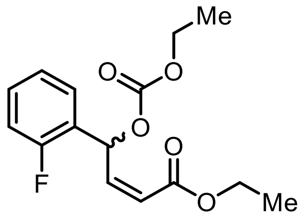

Ethyl-(*Z*)-4-((ethoxycarbonyl)oxy)-4-(2-fluorophenyl)but-2-enoate (±)-4f

From ethyl 4-((ethoxycarbonyl)oxy)-4-(2-fluorophenyl)but-2-ynoate (2.44 g, 8.3 mmol, 1.0 equiv.) following General Procedure E. Purification by silica chromatography (gradient of 0 to 30% Et_2O/hexane) afforded ethyl (Z)-4-((ethoxycarbonyl)oxy)-4-(2-fluorophenyl)but-2-enoate (2.16 g, 7.3 mmol, 88%) as a colourless oil.

^{1}H NMR (400 MHz, $CDCl_3$) δ 7.50 (td, J = 7.4, 1.8 Hz, 1H), 7.35 – 7.27 (m, 2H), 7.12 (td, J = 7.6, 1.2 Hz, 1H), 7.05 (ddd, J = 10.5, 8.3, 1.2 Hz, 1H), 6.59 (ddd, J = 11.5, 8.2, 2.7 Hz, 1H), 5.92 (dd, J = 11.6, 1.4 Hz, 1H), 4.26 – 4.09 (m, 4H), 1.26 (qd, J = 7.2, 0.9 Hz, 6H).

^{19}F NMR (376 MHz, $CDCl_3$) δ -114.88 – -115.03 (m).

^{13}C NMR (101 MHz, $CDCl_3$) δ 165.1, 160.8 (d, J = 250.6 Hz), 154.2, 143.5 (d, J = 2.3 Hz), 130.7 (d, J = 7.5 Hz), 130.4 (d, J = 4.0 Hz), 125.2 (d, J = 13.4 Hz), 124.2 (d, J = 3.6 Hz), 121.8, 116.1 (d, J = 21.3 Hz), 71.9 (d, J = 1.6 Hz), 64.4, 60.7, 14.3, 14.2.

IR (CH_3Cl film): 2986, 2940, 2912, 2361, 2334, 1749, 1719, 1653, 1617, 1590, 1494, 1458, 1412, 1389, 1372, 1256, 1230, 1195, 1107, 1027, 1005 cm^{-1}

HRMS (ESI): m/z calculated for $C_{15}H_{17}FO_5Na^+$ $[M+Na]^+$ 319.0952 found 319.0946.

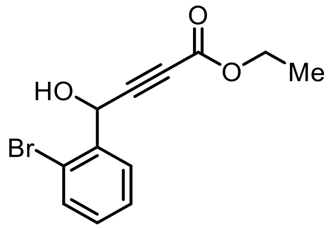

Ethyl 4-(2-bromophenyl)-4-hydroxybut-2-ynoate S12

From 2-bromobenzaldehyde (3.50 mL, 30 mmol) following General Procedure C. Purification by silica chromatography (10-40% EtOAc: Hexane) ethyl 4-(2-bromophenyl)-4-hydroxybut-2-ynoate (4.15 g, 14.7 mmol, 49%) as a pale-yellow oil. Characterisation data match the literature reports.[8]

^{1}H NMR (400 MHz, $CDCl_3$) δ 7.72 (dd, J = 7.7, 1.7 Hz, 1H), 7.59 (dd, J = 8.0, 1.2 Hz, 1H), 7.39 (td, J = 7.6, 1.3 Hz, 1H), 7.26 – 7.21 (m, 1H), 5.91 (s, 1H), 4.25 (q, J = 7.1 Hz, 2H), 2.56 (s, 1H), 1.32 (t, J = 7.1 Hz, 3H).

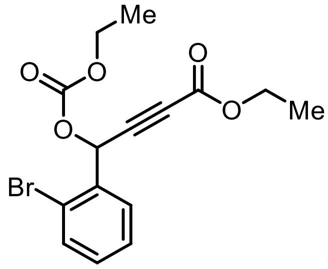

Ethyl 4-(2-bromophenyl)-4-((ethoxycarbonyl)oxy)but-2-ynoate S13

From ethyl 4-(2-bromophenyl)-4-hydroxybut-2-ynoate (4.06 g, 14.3 mmol, 1.0 equiv.) following General Procedure D. Purification by silica chromatography (10% EtOAc: Hexane) afforded (4.56 g, 12.8 mmol, 90%) ethyl 4-(2-bromophenyl)-4-((ethoxycarbonyl)oxy)but-2-ynoate) as a pale-yellow oil.

^{1}H NMR (400 MHz, $CDCl_3$**)** δ 7.73 (dt, J = 7.7, 1.4 Hz, 1H), 7.61 (dt, J = 8.0, 1.3 Hz, 1H), 7.40 (tt, J = 7.6, 1.2 Hz, 1H), 7.32 – 7.23 (m, 1H), 6.73 (s, 1H), 4.34 – 4.20 (m, 4H), 1.38 – 1.28 (m, 6H).

^{13}C NMR (101 MHz, $CDCl_3$**)** δ 153.7, 152.9, 134.1, 133.3, 131.2, 129.7, 128.1, 123.2, 81.3, 79.1, 67.9, 65.1, 62.5, 14.3, 14.1.

IR (CH_3Cl film): 2986, 2360, 2246, 1755, 1716, 1573, 1471, 1445, 1371, 1262, 1234, 1194, 1080, 1006 cm^{-1}

HRMS (ESI): m/z calculated for $C_{15}H_{15}BrO_5Na^+$ $[M+Na]^+$ 376.9995 found 376.9991.

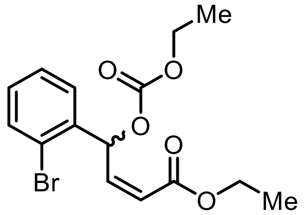

Ethyl (*Z*)-4-(2-bromophenyl)-4-((ethoxycarbonyl)oxy)but-2-enoate (±)-4g

From ethyl 4-(2-bromophenyl)-4-((ethoxycarbonyl)oxy)but-2-ynoate (2.95 g, 8.3 mmol, 1.0 equiv.) following General Procedure E. Purification by silica chromatography (gradient of 0 to 30% Et_2O/hexane) afforded ethyl (Z)-4-(2-bromophenyl)-4-((ethoxycarbonyl)oxy)but-2-enoate (1.40 g, 3.9 mmol, 47%) as a pale-yellow oil.

^{1}H NMR (400 MHz, $CDCl_3$**)** δ 7.57 (dd, J = 8.0, 1.3 Hz, 1H), 7.45 (dd, J = 7.7, 1.7 Hz, 1H), 7.38 (dd, J = 8.1, 1.4 Hz, 1H), 7.30 (td, J = 7.6, 1.3 Hz, 1H), 7.17 (ddd, J = 8.0, 7.4, 1.7 Hz, 1H), 6.46 (dd, J = 11.6, 8.1 Hz, 1H), 6.01 (dd, J = 11.6, 1.4 Hz, 1H), 4.26 – 4.11 (m, 4H), 1.26 (dt, J = 18.1, 7.1 Hz, 6H).

^{13}C NMR (101 MHz, $CDCl_3$**)** δ 165.0, 154.1, 142.8, 137.7, 133.5, 130.2, 129.4, 127.7, 123.7, 123.2, 74.9, 64.5, 60.8, 14.3, 14.2.

IR (CH_3Cl film): 2985, 2360, 2341, 1749, 1720, 1654, 1571, 1473, 1444, 1371, 1249, 1200, 1188, 1027, 1004 cm^{-1}.

HRMS (ESI): m/z calculated for $C_{15}H_{17}BrO_5Na^+$ $[M+Na]^+$ 379.0152 found 379.0145.

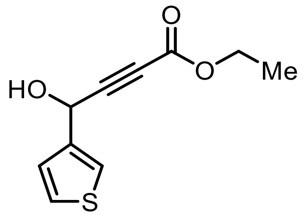

Ethyl 4-hydroxy-4-(thiophen-3-yl)but-2-ynoate S14

From 3-Thiophenecarboxaldehyde (2.63 mL, 30 mmol) following General Procedure C. Purification by silica chromatography (10-40% EtOAc: Hexane) ethyl 4-hydroxy-4-(thiophen-3-yl)but-2-ynoate (4.62 g, 22.0 mmol, 74%) as a pale-yellow oil. Characterisation data match the literature reports.[9]

^{1}H NMR (400 MHz, $CDCl_3$) δ 7.48 – 7.42 (m, 1H), 7.35 (m, 1H), 7.20 (dd, J = 5.0, 1.3 Hz, 1H), 5.65 – 5.60 (m, 1H), 4.26 (q, J = 7.1 Hz, 2H), 2.29 (s, 1H), 1.32 (t, J = 7.1 Hz, 3H)

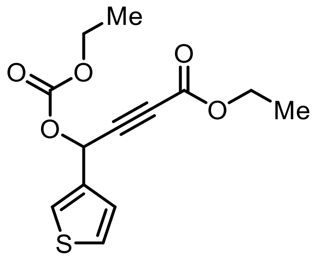

Ethyl 4-((ethoxycarbonyl)oxy)-4-(thiophen-3-yl)but-2-ynoate S15

From ethyl 4-hydroxy-4-(thiophen-3-yl)but-2-ynoate (4.62 g, 22.0 mmol, 1.0 equiv.) following General Procedure D. Purification by silica chromatography (10% EtOAc: Hexane) gave ethyl 4-((ethoxycarbonyl)oxy)-4-(thiophen-3-yl)but-2-ynoate (5.75 g, 20.4 mmol, 93%) as a pale yellow oil.

^{1}H NMR (400 MHz, $CDCl_3$) δ 7.51 (ddt, J = 3.0, 1.4, 0.7 Hz, 1H), 7.37 – 7.30 (m, 1H), 7.20 (dd, J = 5.1, 1.3 Hz, 1H), 6.44 (s, 1H), 4.32 – 4.15 (m, 4H), 1.30 (tdd, J = 7.2, 2.7, 0.9 Hz, 6H).

^{13}C NMR (101 MHz, $CDCl_3$) δ 154.0, 152.9, 135.1, 127.1, 126.6, 125.8, 81.8, 78.4, 65.0, 64.0, 62.5, 14.3, 14.1.

IR (CH_3Cl film): 3109, 2979, 2971, 2361, 2341, 2248, 1751, 1716, 1468, 1447, 1401, 1370, 1239, 1158, 1077, 1008 cm^{-1}.

HRMS (ESI): m/z calculated for $C_{13}H_{14}O_5SNa^+$ $[M+Na]^+$ 305.0454 found 305.0444.

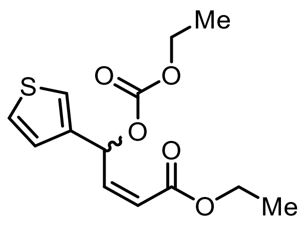

Ethyl (*Z*)-4-((ethoxycarbonyl)oxy)-4-(thiophen-3-yl)but-2-enoate (±)-4h

Ethyl 4-((ethoxycarbonyl)oxy)-4-(thiophen-3-yl)but-2-ynoate (2.34 g, 8.3 mmol, 1.0 equiv.) following General Procedure E. Purification by silica chromatography (gradient of 0 to 30% Et_2O/hexane) afforded ethyl (Z)-4-((ethoxycarbonyl)oxy)-4-(thiophen-3-yl)but-2-enoate (1.19 g, 4.2 mmol, 50%) as a pale yellow oil.

^{1}H NMR (400 MHz, $CDCl_3$) δ 7.40 (ddd, J = 3.1, 1.3, 0.7 Hz, 1H), 7.34 (dt, J = 8.5, 1.0 Hz, 1H), 7.30 – 7.26 (m, 1H), 7.16 (dd, J = 5.0, 1.3 Hz, 1H), 6.37 (dd, J = 11.5, 8.5 Hz, 1H), 5.90 (dd, J = 11.5, 1.3 Hz, 1H), 4.26 – 4.13 (m, 4H), 1.28 (td, J = 7.1, 2.8 Hz, 6H).

^{13}C NMR (101 MHz, $CDCl_3$) δ 170.6, 159.5, 149.4, 143.7, 131.5, 131.5, 128.8, 126.3, 77.2, 69.6, 66.0, 19.6, 19.5.

IR (CH_3Cl film): 3115, 2984, 2939, 2908, 2360, 2342, 1748, 1718, 1653, 1467, 1447, 1412, 1388, 1371, 1254, 1192, 1087, 1026, 1006 cm^{-1}.

HRMS (ESI): m/z calculated for $C_{13}H_{16}O_5SNa^+$ $[M+Na]^+$ 307.0611 found 307.0606.

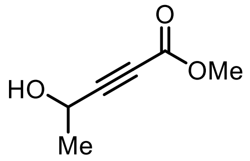

Methyl 4-hydroxypent-2-ynoate S16

From acetaldehyde (1.7 ml, 30.0 mmol, 1.0 equiv.) following General Procedure C. Purification by silica chromatography (10-40% EtOAc: Hexane) methyl 4-hydroxypent-2-ynoate (2.38 g, 18.6 mmol, 62%) as a brown oil. Characterisation data match the literature reports.[10]

^{1}H NMR (400 MHz, $CDCl_3$): δ 4.64 (q, J = 5.1 Hz, 1H), 3.78 (s, 3H), 2.19 (br, 1H), 1.51 (d, J = 6.7 Hz, 3H).

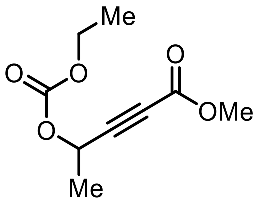

Methyl 4-((ethoxycarbonyl)oxy)pent-2-ynoate S17

From methyl 4-hydroxypent-2-ynoate (2.38 g, 18.6 mmol, 1.0 equiv.) following General Procedure D. Purification by silica chromatography (EtOAc/hexane) afforded methyl 4-((ethoxycarbonyl)oxy)pent-2-ynoate (2.47 g, 12.3 mmol, 66%) as a pale yellow oil.

^{1}H NMR (400 MHz, $CDCl_3$) δ 5.38 (q, *J* = 6.8 Hz, 1H), 4.22 (q, *J* = 7.1 Hz, 2H), 3.77 (s, 3H), 1.58 (d, *J* = 6.8 Hz, 3H), 1.31 (t, *J* = 7.1 Hz, 3H).

^{13}C NMR (101 MHz, $CDCl_3$) δ 154.0, 153.5, 84.5, 76.7, 64.8, 63.2, 53.0, 20.5, 14.3.

HRMS (ESI): m/z calculated for $C_9H_{12}O_5Na^+$ $[M+Na]^+$ 223.0577 found 223.0573.

IR (CH_3Cl film): 2994, 2249, 1753, 1722, 1437, 1374, 1309, 1242, 1096, 1045, 1007 cm^{-1}.

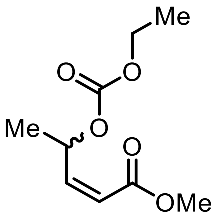

Methyl (*Z*)-4-((ethoxycarbonyl)oxy)pent-2-enoate (±)-4i

From methyl 4-((ethoxycarbonyl)oxy)pent-2-ynoate (1.20 g, 6 mmol) following General Procedure E. Purification by silica chromatography (gradient of 0 to 20% Et_2O/hexane) afforded methyl (Z)-4-((ethoxycarbonyl)oxy)pent-2-enoate (1.02 g, 84%) as a pale yellow oil.

^{1}H NMR (400 MHz, $CDCl_3$) δ 6.25 – 6.13 (m, 2H), 5.87 – 5.77 (m, 1H), 4.18 (q, *J* = 7.1 Hz, 2H), 3.73 (s, 3H), 1.48 – 1.37 (m, 3H), 1.30 (t, *J* = 7.1 Hz, 3H).

^{13}C NMR (101 MHz, $CDCl_3$) δ 165.9, 154.5, 148.2, 119.8, 72.1, 64.1, 51.7, 19.7, 14.4.

IR (CH_3Cl film): 2989, 2955, 1748, 1726, 1656, 1439, 1405, 1373, 1328, 1306, 1264, 1206, 1184, 1126, 1044, 1007 cm^{-1}.

HRMS (ESI): m/z calculated for $C_9H_{14}O_5Na^+$ $[M+Na]^+$ 225.0734 found 225.0728.

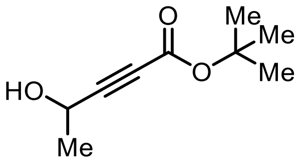

***Tert*-butyl 4-hydroxypent-2-ynoate S18**

From *tert*-butyl propiolate (4.94 ml, 36.0 mmol, 1.2 equiv.) and acetaldehyde (1.7 ml, 30.0 mmol, 1.0 equiv.) following General Procedure C. Purification by silica chromatography (EtOAc/hexane) afforded the product (5.11 g, 30.0 mmol, 100%) as a colourless oil.

^{1}H NMR (400 MHz, $CDCl_3$) δ 4.61 (q, J = 6.7 Hz, 1H), 2.06 (s, 1H), 1.50 (d, J = 7.0 Hz, 3H), 1.50 (s, 9H).

^{13}C NMR (101 MHz, $CDCl_3$) δ 152.6, 86.1, 83.9, 77.2, 58.2, 28.1, 23.5.

IR (CH_3Cl film): 3400, 2984, 2937, 2362, 2232, 1709, 1479, 1458, 1396, 1371, 1276, 1258, 1158, 1126, 1064 cm^{-1}.

HRMS (ESI): m/z calculated for $C_9H_{14}O_3Na^+$ $[M+Na]^+$ 193.0835 found 193.0837.

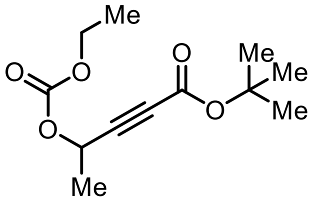

***Tert*-butyl 4-((ethoxycarbonyl)oxy)pent-2-ynoate S19**

From *tert*-butyl 4-hydroxypent-2-ynoate (5.60 g, 33.0 mmol, 1.0 equiv.) following General Procedure D. Purification by silica chromatography (10% EtOAc: Hexane) gave *tert*-butyl 4-((ethoxycarbonyl)oxy)pent-2-ynoate (7.40 g, 20.4 mmol, 93%) as a colourless oil.

^{1}H NMR (400 MHz, $CDCl_3$) δ 5.35 (q, J = 6.8 Hz, 1H), 4.19 (q, J = 7.1 Hz, 2H), 1.54 (d, J = 6.8 Hz, 3H), 1.45 (s, 9H), 1.28 (t, J = 7.1 Hz, 3H).

^{13}C NMR (101 MHz, CDCl3) δ 154.0, 152.0, 83.9, 81.7, 78.2, 64.6, 63.3, 28.0, 20.6, 14.2.

IR (CH_3Cl film): 2985, 2941, 2241, 1752, 1711, 1478, 1458, 1396, 1372, 1340, 1307, 1280, 1247, 1159, 1114, 1096, 1046, 1035, 1006 cm^{-1}.

HRMS (ESI): m/z calculated for $C_{12}H_{18}O_5Na^+$ $[M+Na]^+$ 265.1047 found 265.1039.

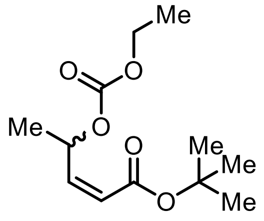

***Tert*-butyl (*Z*)-4-((ethoxycarbonyl)oxy)pent-2-enoate (±)-4j**

From *tert*-butyl 4-((ethoxycarbonyl)oxy)pent-2-ynoate (2.01 g, 8.3 mmol, 1.0 equiv.) following General Procedure E. Purification by silica chromatography (gradient of 0 to 20% Et_2O/hexane) afforded *tert*-butyl (Z)-4-((ethoxycarbonyl)oxy)pent-2-enoate 1.67 g, 6.81 mmol, 82%) as a colourless oil.

^{1}H NMR (400 MHz, $CDCl_3$) δ 6.16 (dqd, J = 7.3, 6.3, 1.1 Hz, 1H), 6.09 (dd, J = 11.5, 7.6 Hz, 1H), 5.72 (dd, J = 11.5, 1.2 Hz, 1H), 4.18 (q, J = 7.1 Hz, 2H), 1.42 (d, J = 6.4 Hz, 3H), 1.30 (t, J = 7.1 Hz, 3H).

^{13}C NMR (101 MHz, $CDCl_3$) δ 164.9, 154.5, 146.2, 122.2, 81.2, 72.2, 64.0, 28.3, 19.9, 14.4.

IR (CH_3Cl film): 2982, 2938, 2910, 1748, 1714, 1651, 1456, 1409, 1371, 1325, 1305, 1260, 1240, 1218, 1161, 1123, 1043, 1007 cm^{-1}.

HRMS (ESI): m/z calculated for $C_{12}H_{20}O_5Na$ $[M+Na]^+$ 267.1203 found 267.1197.

5. Procedures for the product derivatization

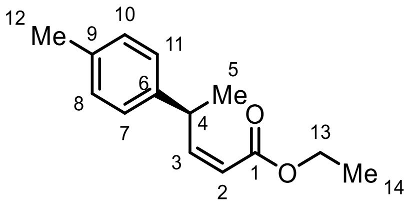

Ethyl (*S*,*Z*)-4-(p-tolyl)pent-2-enoate *Z*-3d

General Procedure A was carried out using (4-methyl)phenylboronic acid **2d** (108.8 mg, 0.80 mmol, 2.0 equiv.) and only Z-product was purified by medium-pressure automated flash chromatography to afford ethyl (*S*,*Z*)-4-(p-tolyl)pent-2-enoate (70.7 mg, 32.4 mmol, 81%) as a colourless oil. SFC analysis showed an enantiomeric excess of 96%.

^{1}H NMR (400 MHz, $CDCl_3$) δ 7.20 (d, *J* = 8.1 Hz, 2H, C(7)-H and C(11)-H), 7.13 (d, *J* = 7.9 Hz, 2H, C(8)-H and C(10)-H), 6.25 (dd, *J* = 11.4, 10.4 Hz, 1H, C(3)-H), 5.72 (dd, *J* = 11.4, 1.0 Hz, 1H, C(2)-H), 4.87 (dq, *J* = 10.4, 7.0 Hz, 1H, C(4)-H), 4.20 (q, *J* = 7.2 Hz, 2H, C(13)-H_2), 2.32 (s, 3H, C(12)-H_3), 1.39 (d, *J* = 6.9 Hz, 3H, C(5)-H_3), 1.31 (t, *J* = 7.1 Hz, 3H, C(14)-H_3).

^{13}C NMR (101 MHz, $CDCl_3$) δ 166.3 (C(1)), 154.0 (C(3)), 141.7 (C(6)), 136.0 (C(9)), 129.3 (C(8) and C(10)), 126.9 (C(7) and C(11)), 117.6 (C(2)), 59.9 (C(13)), 37.3 (C(4)), 21.0 (C(12)), 20.9 (C(5)), 14.3 (C(14)).

IR (CH_3Cl film): 2978 (w), 1718 (s), 1642 (m), 1513 (m), 1455(w), 1441 (w), 1100 (s), 1098 (w), 1033 (m), 815 (m), 755 (s) cm^{-1}.

HRMS (ESI): m/z calculated for $C_{14}H_{19}O_2^+$ $[M+H]^+$ 219.1380 found 219.1382.

SFC: Chiralpak® IG, 1500 psi, 30 °C; flow: 1.0 mL/min; 1% to 30% MeOH over 5 min, 97.8:2.2 e.r. (minor enantiomer t_R = 1.72 min, major enantiomer t_R = 1.52 min).

α_D^{25} = +337.5 (c = 1.0, $CHCl_3$)

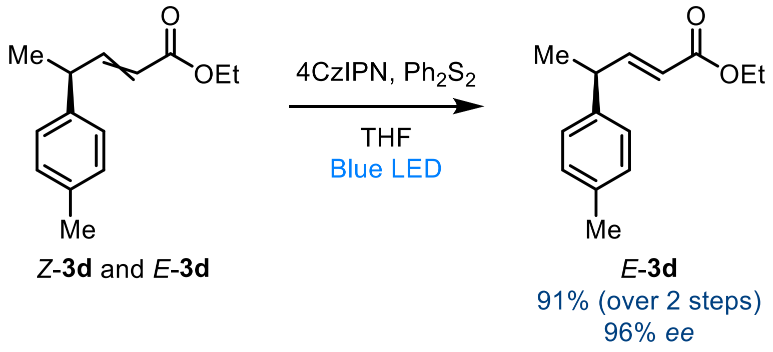

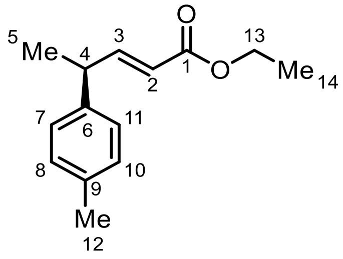

ethyl (*S*,*E*)-4-(*p*-tolyl)pent-2-enoate *E*-3d

General Procedure A was carried out using (*p*-tolyl)phenylboronic acid **2d** (108.8 mg, 0.80 mmol, 2.0 equiv.) and crude product (containing *Z*-**3d** and *E*-**3d** with Z:E ratio of 4.4:1) was used in the next step.

4CzIPN (15.8 mg, 0.020 mmol, 0.05 equiv.), diphenyl disulphide (96.1 mg, 0.44 mmol, 1.1 equiv.) and THF (4.0 ml) were added to the crude product (containing *Z*-**3d** and *E*-**3d**) and the reaction mixture was irradiated with blue light (Evoluchem 450PF 450 nm CREE XPE (part number HCK1012-01-002), 18 W power consumption and 34 mW/cm^2 output relative irradiance) for 2 h. The mixture was filtered through a plug of silica with Et_2O and the solvent was removed in vacuo. Purification by flash chromatography afforded ethyl (*S*,*E*)-4-(*p*-tolyl)pent-2-enoate *E*-**3d** (79.5 mg, 0.36 mmol, 91%) as a colourless oil. SFC analysis showed an enantiomeric excess of 96%. Characterisation data match literature reports.[14]

^{1}H NMR (400 MHz, $CDCl_3$) δ 7.10 (m, 5H, C(3)-H, C(7)-H, C(8)-H, C(10)-H and C(11)-H), 5.79 (dd, *J* = 15.7, 1.6 Hz, 1H, C(2)-H), 4.17 (q, *J* = 7.1 Hz, 2H, C(13)-H_2), 3.58 (m, 1H, C(4)-H), 2.33 (s, 3H, C(12)-H_3), 1.41 (d, *J* = 7.0 Hz, 3H, C(5)-H_3), 1.27 (t, *J* = 7.1 Hz, 3H, C(14)-H_3).

^{13}C NMR (101 MHz, $CDCl_3$) δ 166.8 (C(1)), 152.8 (C(3)), 140.3 (C(6)), 136.3 (C(9)), 129.4 (C(8) and C(10)), 127.2 (C(7) and C(11)), 120.0 (C(2)), 60.3 (C(13)), 41.7 (C(4)), 21.0 (C(12)), 20.3 (C(5)), 14.3 (C(14)).

IR (CH_3Cl film): 2971 (w), 1716 (m), 1651 (w), 1514 (w), 1455(w), 1368 (w), 1268 (m), 1216 (m), 1174 (m), 1131 (w), 1036 (m), 1016 (w), 981 (w), 866 (w), 818 (m), 754 (s), 668 (m), cm^{-1}.

HRMS (ESI): m/z calculated for $C_{14}H_{19}O_2^+$ $[M+H]^+$ 219.1380 found 219.1379.

SFC: Chiralpak® IG, 1500 psi, 30 °C; flow: 1.0 mL/min; 1% to 30% MeOH over 5 min, 97.9:2.1 e.r. (minor enantiomer t_R = 2.27 min, major enantiomer t_R = 1.86 min).

$\boldsymbol{\alpha_D^{25}}$ = −11.2 (c = 1.0, $CHCl_3$) (Lit.[5]: α_D^{30} = −13.6 (c = 1.3, $CHCl_3$)).

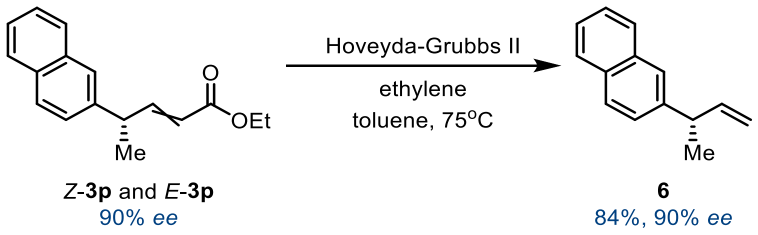

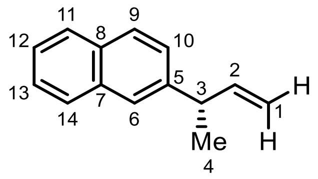

(*S*)-2-(but-3-en-2-yl)naphthalene 6

A mixture of (*S*,*Z*)-and (*S*,*E*)-4-(naphthalen-2-yl)pent-2-enoate *Z*-**3p** and *E*-**3p** resulting from the Rh-catalyzed coupling reaction (0.4 mmol, 1.0 equiv.) was dissolved in toluene (2.0 ml) in a 10 ml ampoule fitted with a Young's valve. The ampoule was cycled onto a Schlenk line, carefully evacuated, and then pressurised to 2 Bar with ethylene. The reaction mixture was stirred at 80°C for 24 h, after which the ampoule was vented carefully. The reaction mixture was diluted with hexane, filtered through a short plug of silica eluting with hexane and concentrated *in vacuo*. Purification by flash column chromatography (hexane, 100%) afforded (*S*)-2-(but-3-en-2-yl)naphthalene **6** (61.2 mg, 0.34 mmol, 84%) as a colourless oil. SFC analysis showed an enantiomeric excess of 90%. Characterisation data match literature reports.[15]

^{1}H NMR (400 MHz, $CDCl_3$) δ 7.80 (ddd, *J* = 8.3, 4.7, 1.9 Hz, 3H, C(Ar)-H x3), 7.65 (dd, *J* = 1.7, 0.9 Hz, 1H, C(Ar)-H), 7.44 (m, 2H, C(Ar)-H x2), 7.37 (dd, *J* = 8.5, 1.8 Hz, 1H, C(Ar)-H), 6.09 (ddd, *J* = 17.2, 10.3, 6.4 Hz, 1H, C(2)-H), 5.10 (m, 2H, C(1)-H x2), 3.64 (p, *J* = 7.0 Hz, 1H, C(3)-H), 1.46 (d, *J* = 7.0 Hz, 3H, C(4)-H_3).

^{13}C NMR (126 MHz, $CDCl_3$) δ 143.1 C(2), 143.0 C(Ar), 133.6 C(Ar), 132.2 C(Ar), 128.0 C(Ar), 127.6 C(Ar), 127.6 C(Ar), 126.3 C(Ar), 125.9 C(Ar), 125.3 C(Ar), 125.2 C(Ar), 113.4 C(1), 43.3 C(3), 20.7 C(4).

IR (CH_3Cl film): 3056 (w), 2928 (w), 1632 (m), 1600 (w), 1506 (m), 1453 (m), 1412 (w), 1373 (w), 1271 (w) cm^{-1}.

HRMS (GC EI MS): m/z calculated for $C_{14}H_{14}^+$ $[M]^+$ 182.10900 found 182.11142.

SFC: Chiralpak® IG, 1500 psi, 30 °C; flow: 1.0 mL/min; 0% MeOH for 3min; 0% to 10% MeOH over 5 min, 94.8:5.2 e.r. (minor enantiomer t_R = 4.72 min, major enantiomer t_R = 4.38 min).

α_D^{25} = +10.6 (c = 1.0, $CHCl_3$) (Lit[16].: α_D^{28} = +12.6 (*ee* = 96%, c = 1.0, $CHCl_3$)).

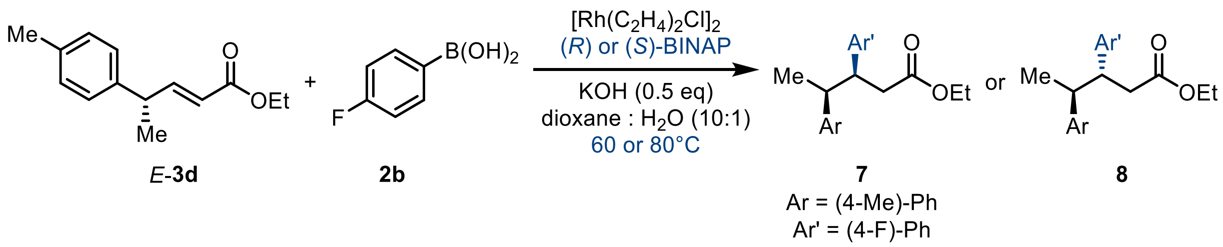

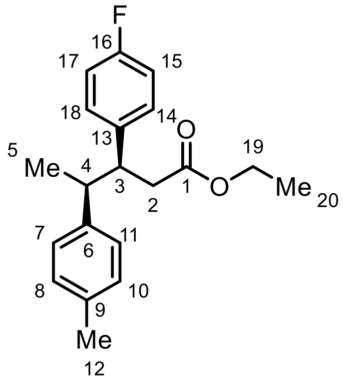

ethyl (3*S*,4*S*)-3-(4-fluorophenyl)-4-(p-tolyl)pentanoate 7

$[Rh(C_2H_4)_2Cl]_2$ (1.9 mg, 0.005 mmol, 0.025 equiv.) and (*S*)-BINAP (7.5 mg, 0.012 mmol, 0.06 equiv.) were dissolved in dioxane (1.0 ml, 0.2 M) and stirred for 10 minutes. KOH (1 M/H_2O, 0.10 ml, 0.10 mmol, 0.5 equiv.) was added; the mixture was stirred for further 5 minutes and transferred into a flask containing (4-fluoro)phenylboronic acid **2b** (84.0 mg, 0.60 mmol, 3.0 equiv.). Ethyl (*S*,*E*)-4-(p-tolyl)pent-2-enoate *E*-**3d** (43.7 mg, 0.20 mmol, 1.0 equiv.) was added and the reaction mixture was stirred at 60°C for 14 h. The reaction was quenched with hexane (5 ml) and filtered through a plug of silica. The solvent was evaporated in vacuo and the residue was purified by column chromatography to give ethyl (3*S*,4*S*)-3-(4-fluorophenyl)-4-(p-tolyl)pentanoate **7** (49.0 mg, 0.16 mmol, 78%) as a colourless oil.

^{1}H NMR (400 MHz, $CDCl_3$) δ 6.98 (dd, *J* = 7.5, 0.8 Hz, 2H, C(Ar)-H x2), 6.86 (m, 6H, C(Ar)-H x6), 3.97 (m, 2H, C(19)-H_2), 3.34 (ddd, *J* = 10.0, 7.2, 5.7 Hz, 1H, C(3)-H), 2.97 (p, *J* = 7.1 Hz, 1H, C(4)-H), 2.76 (dd, *J* = 15.3, 5.7 Hz, 1H, C(2)-H), 2.57 (dd, *J* = 15.3, 10.0 Hz, 1H, C(2)-H), 2.27 (s, 3H, C(12)-H_3), 1.27 (d, *J* = 7.1 Hz, 3H, C(5)-H_3), 1.07 (t, *J* = 7.1 Hz, 3H, C(20)-H_3).

^{13}C NMR (126 MHz, $CDCl_3$) δ 172.4 ((C(1)), 161.4 (d, *J* = 243.9 Hz, C(16)), 140.7 (C(Ar)), 137.4 (d, *J* = 3.2 Hz, C(13)), 135.7 (C(Ar)), 129.9 (d, *J* = 7.8 Hz, C(14) and C(18)), 128.6 (C(Ar) x2), 128.0 (C(Ar) x2), 114.5 (d, *J* = 21.2 Hz, C(15) and C(17)), 60.3 (C(19)), 47.8 (C(3)), 44.5 (C(4)), 38.2 (C(2)), 21.0 (C(12)), 18.8 (C(5)), 14.1 (C(20)).

^{19}F (13C)NMR (470 MHz, $CDCl_3$) δ −116.6.

IR (CH_3Cl film): 3968 (br), 1732 (s), 1605 (m), 1511 (s), 1375 (m), 1219 (s), 1159 (s), 1103 (w), 1017 (w), 834 (m), 756 (s), 668 (w), 629 (w) cm^{-1}.

HRMS (ESI): m/z calculated for $C_{20}H_{23}O_2FNa^+$ $[M+Na]^+$ 337.1574 found 337.1574.

$\boldsymbol{\alpha_D^{25}}$ = +25.3 (c = 1.0, $CHCl_3$)

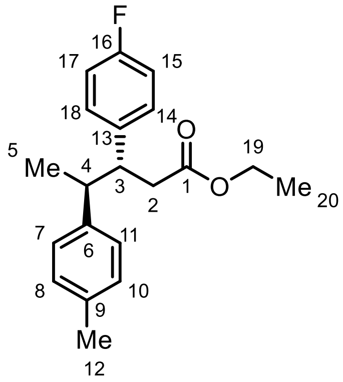

ethyl (3*R*,4*S*)-3-(4-fluorophenyl)-4-(p-tolyl)pentanoate 8

$[Rh(C_2H_4)_2Cl]_2$ (3.9 mg, 0.010 mmol, 0.05 equiv.) and (*R*)-BINAP (14.9 mg, 0.024 mmol, 0.12 equiv.) were dissolved in dioxane (1.0 ml, 0.2 M) and stirred for 10 minutes. KOH (1 M/H_2O, 0.10 ml, 0.10 mmol, 0.5 equiv.) was added; the mixture was stirred for further 5 minutes and transferred into a flask containing (4-fluoro)phenylboronic acid **2b** (84.0 mg, 0.60 mmol, 3.0 equiv.). Ethyl (*S*,*E*)-4-(p-tolyl)pent-2-enoate *E*-**3d** (43.7 mg, 0.20 mmol, 1.0 equiv.) was added and the reaction mixture was stirred at 80°C for 14 h. The reaction was quenched with hexane (5 ml) and filtered through a plug of silica. The solvent was evaporated in vacuo and the residue was purified by column chromatography to give ethyl (3*R*,4*S*)-3-(4-fluorophenyl)-4-(p-tolyl)pentanoate **8** (42.8 mg, 0.14 mmol, 68%) as a colourless oil.

^{1}H NMR (400 MHz, $CDCl_3$) δ 7.19 (m, 2H), 7.12 (m, 4H), 7.00 (m, 2H), 3.84 (q, *J* = 7.2 Hz, 2H), 3.20 (td, *J* = 10.0, 5.6 Hz, 1H), 2.80 (dq, *J* = 10.4, 6.9 Hz, 1H), 2.40 (d, *J* = 2.2 Hz, 1H), 2.38 (d, *J* = 6.4 Hz, 1H), 2.34 (s, 3H), 1.00 (m, 6H).

^{13}C NMR (126 MHz, $CDCl_3$) δ 172.3, 161.6 (d, *J* = 244.1 Hz), 142.1, 138.4 (d, *J* = 3.5 Hz), 136.1, 129.6 (d, *J* = 7.7 Hz), 129.3, 127.4, 115.1 (d, *J* = 21.0 Hz), 60.1, 48.6, 45.5, 40.2, 21.0, 20.6, 14.0.

^{19}F (13C)NMR (470 MHz, $CDCl_3$) δ −116.6.

IR (CH_3Cl film): 2974 (m), 2361 (w), 1733 (s), 1605 (m), 1510 (s), 1455 (m), 1374 (m), 1224 (s), 1159 (s), 1103 (m) cm^{-1}.

HRMS (ESI): m/z calculated for $C_{20}H_{24}O_2F^+$ $[M+H]^+$ 315.1755 found 315.1755.

α_D^{25} = +4.5 (c = 1.0, $CHCl_3$)

Absolute stereochemistry was assigned by comparing ^{1}H NMR and α_D of **8** to those of ethyl (3S,4R)-3,4-diphenylpentanoate.[17]

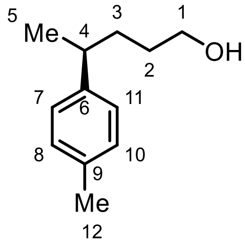

(*S*)-4-(p-tolyl)pentan-1-ol 9

$LiAlH_4$ (0.44 ml, 0.88 mmol, 2.2 eq, 2.0 M solution in THF) was added to a solution of ethyl (*S*)-4-(*p*-tolyl)pentanoate *red*-**3d** (81.7 mg, 0.40 mmol, 1.0 equiv.) in THF (1.10 ml) at 0°C and the mixture was refluxed for 5 hours. Reaction mixture was cooled to 0°C and diluted with EtOAc. The reaction was quenched by slow addition of water and 10% NaOH. Organic and aqueous layers were separated and aqueous layer was extracted with Et_2O (x3). Combined organic phases were washed with brine and dried over Na_2SO_4. The solvent was evaporated in vacuo and the residue was purified by column chromatography to give (*S*)-4-(*p*-tolyl)pentan-1-ol **9** (62.4 mg, 0.35 mmol, 88%) as a colourless liquid. SFC analysis showed an enantiomeric excess of 96%. Characterisation data match literature reports.[18]

^{1}H NMR (400 MHz, $CDCl_3$) δ 7.11 (m, 4H, C(Ar)-H x4), 3.59 (t, *J* = 6.5 Hz, 2H, C(1)-H_2), 2.68 (h, *J* = 7.0 Hz, 1H, C(4)-H), 2.33 (s, 3H, C(12)-H_3), 1.63 (m, 2H, C(2)-H_2), 1.48 (m, 2H, C(3)-H_2), 1.26 (dd, *J* = 6.9, 1.1 Hz, 3H, C(5)-H_3).

^{13}C NMR (101 MHz, $CDCl_3$) δ 144.3 (C(6)), 135.4 (C(9)), 129.1 (C(Ar) x2), 126.9 (C(Ar) x2), 63.1 (C(1)), 39.4 (C(4)), 34.4 (C(2)), 31.0 (C(3)), 22.5 (C(5)), 21.0 (C(12)).

IR (CH_3Cl film): 3334 (br), 2955 (s), 2930 (s), 2870 (s), 1515 (s), 1455 (m), 1059 (s), 1023 (m), 816 (s), 757 (s) cm^{-1}.

HRMS (ESI): m/z calculated for $C_{12}H_{18}ONa^+$ $[M+Na]^+$ 201.1250 found 201.1250.

SFC: Chiralpak® IG, 1500 psi, 30 °C; flow: 1.0 mL/min; 1% to 30% MeOH over 5 min, 97.8:2.2 e.r. (minor enantiomer t_R = 3.69 min, major enantiomer t_R = 3.59 min).

α_D^{25} = +15.4 (c = 1.0, $CHCl_3$) (Lit.[5]: α_D^{28} = +14.6 (c = 0.7, $CHCl_3$)).

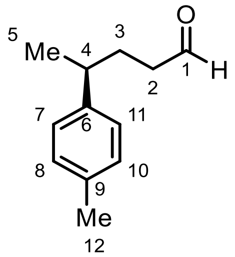

(*S*)-4-(p-tolyl)pentanal S20

(*S*)-4-(p-tolyl)pentan-1-ol **9** (28.7 mg, 0.16 mmol, 1.0 equiv.) in CH_2Cl_2 (0.6 ml) was added to a solution of Dess-Martin periodinane (81.8 mg, 0.19 mmol, 1.2 equiv.) in CH_2Cl_2 (1.0 ml) at 0°C. The reaction mixture was stirred at ambient temperature (23°C) for 1 h, then diluted with Et_2O and poured into a solution of sodium thiosulfate in saturated aqueous $NaHCO_3$. The aqueous layer was extracted with Et_2O (x2). Combined organic phases were washed with brine and dried over Na_2SO_4. The solvent was evaporated in vacuo and the residue was purified by column chromatography to give (*S*)-4-(p-tolyl)pentanal **S20** (23.7 mg, 0.13 mmol, 84%) as a colourless oil. Characterisation data match literature reports.[19]

^{1}H NMR (400 MHz, $CDCl_3$) δ 9.68 (t, *J* = 1.6 Hz, 1H, -COH), 7.12 (m, 2H, C(Ar)-H x2), 7.06 (m, 2H C(Ar)-H x2), 2.68 (m, 1H, C(4)-H), 2.32 (m, 5H, C(2)-H and C(12)-H_3), 1.89 (m, 2H, C(3)-H), 1.27 (d, *J* = 6.9 Hz, 3H, C(5)-H_3).

^{13}C NMR (101 MHz, $CDCl_3$) δ 202.5 (C(1)), 143.0 (C(6)), 135.8 (C(9)), 129.2 (C(Ar) x2), 126.9 (C(Ar) x2), 42.2 (C(2)), 38.9 (C(4)), 30.4 (C(3)), 22.4 (C(5)), 21.0 (C(12)).

IR (CH_3Cl film): 3020 (w), 2960 (w), 2926 (w), 2720 (w), 1724 (s), 1515 (w), 1456 (w), 1377 (w), 1217 (w), 1019 (w), 817 (m), 756 (s), 668 (w) cm^{-1}.

HRMS (GC/EI-MS): m/z calculated for $C_{12}H_{16}O^+$ $[M]^+$ 176.11957 found 176.11867.

$\boldsymbol{\alpha_D^{25}}$ = +17.4 (c = 1.0, $CHCl_3$) (Lit[19].: α_D^{20} = +17.9 (c = 1.4, $CHCl_3$)).

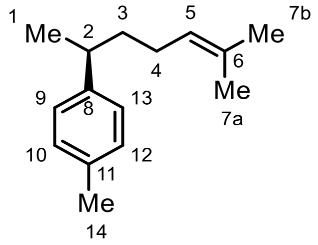

(*S*)-curcumene 10

nBuLi (2.5 M in hexane, 0.13 ml, 0.33 mmol, 3.0 equiv.) was added dropwise to a stirred suspension of isopropyltriphenylphosphonium iodide (141.4 mg, 0.33 mmol, 3.0 equiv.) in THF (ml) at -10°C. The mixture was stirred at -10°C for 30 min. (*S*)-4-(p-tolyl)pentanal **S20** (19.2 mg, 0.11 mmol, 1.0 equiv.) in THF (ml) was added slowly at 0°C and the resulting mixture was stirred at ambient temperature (23°C) for 1 h. The reaction was quenched by slow addition of saturated aqueous NH_4Cl. The aqueous layer was extracted with Et_2O (x3). Combined organic phases were washed with brine and dried over Na_2SO_4. The solvent was evaporated in vacuo and the residue was purified by column chromatography to give (*S*)-curcumene **10** (17.4 mg, 0.086 mmol, 79%) as a colourless oil. SFC analysis showed an enantiomeric excess of 96%. Characterisation data match literature reports.[20]

^{1}H NMR (500 MHz, $CDCl_3$) δ 7.09 (m, 4H, C(Ar)-H x4), 5.10 (tt, *J* = 7.2, 1.5 Hz, 1H, C(5)-H), 2.66 (h, *J* = 7.0 Hz, 1H, C(2)-H), 2.32 (s, 3H, C(14)-H_3), 1.88 (m, *J* = 7.4 Hz, 2H, C(4)-H_2), 1.68 (s, 3H, C(7b)-H_3), 1.59 (m, 2H), 1.53 (s, 3H, C(7a)-H_3), 1.22 (d, *J* = 6.9 Hz, 3H, C(1)-H_3).

^{13}C NMR (126 MHz, $CDCl_3$) δ 144.7 (C(8)), 135.2 (C(11)), 131.4 (C(6)), 129.0 (C(Ar)-H x2), 126.9 (C(Ar)-H x2), 124.6 (C(5)), 39.0 (C(2)), 38.5 (C(3)), 26.2 (C(4)), 25.7 (C(7b)), 22.5 (C(14)), 21.0 (C(1)), 17.7 (C(7a)).

IR (CH_3Cl film): 3657 (w), 2981 (s), 2926 (m), 2888 (m), 1733 (w), 1514 (w), 1455 (w), 1380 (m), 1252 (w), 1217 (w), 1152 (w), 1073 (w), 955 (w), 816 (w), 757 (s), 668 (w) cm^{-1}.

HRMS (GC/EI-MS): m/z calculated for $C_{15}H_{22}^+$ $[M]^+$ 202.17160 found 202.17255.

SFC: Chiralpak® ID, 1500 psi, 30 °C; flow: 1.0 mL/min; 0% MeOH for 3 min, 0% to 10% MeOH over 5 min, 97.9:2.1 e.r. (minor enantiomer t_R = 2.08 min, major enantiomer t_R = 2.19 min).

α_D^{25} = +42.2 (c = 1.0, $CHCl_3$) (Lit[6].: α_D^{25} = +44.6 (c = 1.0, $CHCl_3$)).

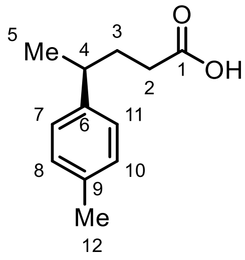

(S)-4-(p-tolyl)pentanoic acid S21

A solution LiOH (38.3 mg, 1.60 mmol, 4 equiv.) in water (0.10 ml) was added to of ethyl (*S*)-4-(p-tolyl)pentanoate *red*-**3d** (81.7 mg, 0.40 mmol, 1.0 equiv.) in THF (0.20 ml) and MeOH (0.10 ml) at 0°C. The reaction mixture was stirred at 50°C for 3h. The reaction was quenched by slow addition of 1 M HCl solution at 0°C. The aqueous layer was extracted with Et_2O (x3). Combined organic phases were washed with brine and dried over Na_2SO_4. The solvent was evaporated in vacuo and the residue was purified by column chromatography to give (*S*)-4-(p-tolyl)pentanoic acid **S21** (59.0 mg, 0.31 mmol, 77%) as a pale-yellow oil. Characterisation data match literature reports.[21]

^{1}H NMR (400 MHz, $CDCl_3$) δ 7.13 (d, *J* = 8.1 Hz, 2H, C(Ar)-H x2), 7.09 (d, *J* = 8.2 Hz, 2H, C(Ar)-H x2), 2.71 (m, 1H, C(4)-H), 2.34 (s, 3H, C(12)-H_3), 2.25 (m, 2H, C(2)-H_2), 1.91 (m, 2H, C(3)-H_2), 1.28 (d, *J* = 6.9 Hz, 3H, C(5)-H_3).

^{13}C NMR (101 MHz, $CDCl_3$) δ 180.3 (C(1)), 143.0 (C(6)), 135.8 (C(9)), 129.2 (C(Ar) x2), 126.9 (C(Ar) x2), 38.9 (C(4)), 33.0 (C(2)), 32.4 (C(3)), 22.3 (C(12)), 21.0 (C(5)).

IR (CH_3Cl film): 2960 (w), 2926 (w), 1707 (s), 1515 (w), 1455 (w), 1414 (w), 1285 (w), 940 (w), 818 (m), 757 (m) cm^{-1}.

HRMS (ESI): m/z calculated for $C_{12}H_{15}O_2^-$ $[M-H]^-$ 191.1078 found 191.1071.

α_D^{25} = +15.6 (c = 1.0, $CHCl_3$) (Lit[21].: α_D^{25} = +14.2 (c = 1.0, $CHCl_3$)).

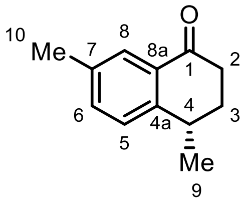

(*S*)-4,7-dimethyl-1-tetralone 11

A solution of trifluoroacetic anhydride (0.17 ml, 1.23 mmol, 4.0 equiv.) and trifluoroacetic acid (0.17 ml, 2.15 mmol, 7.0 equiv.) was added to (*S*)-4-(p-tolyl)pentanoic acid **S21** (59.0 mg, 0.31 mmol, 1.0 equiv.) at 0°C. The reaction mixture was stirred at ambient temperature (23°C) for 10 h, then saturated aqueous $NaHCO_3$ was added to the reaction mixture at 0°C. The aqueous layer was extracted with Et_2O (x3). Combined organic phases were washed with brine and dried over Na_2SO_4. The solvent was evaporated in vacuo and the residue was purified by column chromatography to give (*S*)-4,7-dimethyl-1-tetralone **11** (35.6 mg, 0.20 mmol, 66%) as a colourless oil. SFC analysis showed an enantiomeric excess of 96%. Characterisation data match literature reports.[22]

^{1}H NMR (400 MHz, $CDCl_3$) δ 7.84 (d, *J* = 1.3 Hz, 1H, C(8)-H), 7.33 (ddd, *J* = 7.9, 2.0, 0.7 Hz, 1H, C(6)-H), 7.23 (d, *J* = 7.9 Hz, 1H, C(5)-H), 3.06 (pd, *J* = 7.1, 4.2 Hz, 1H, C(4)-H), 2.78 (ddd, *J* = 17.4, 8.5, 4.5 Hz, 1H, C(2)-H), 2.58 (ddd, *J* = 17.3, 8.8, 4.8 Hz, 1H, C(2)-H), 2.36 (s, 3H, C(10)-H_3), 2.23 (ddt, *J* = 13.2, 8.5, 4.6 Hz, 1H, C(3)-H), 1.88 (dddd, *J* = 13.4, 8.9, 7.4, 4.6 Hz, 1H, C(3)-H), 1.38 (d, *J* = 7.0 Hz, 3H, C(9)-H_3).

^{13}C NMR (126 MHz, $CDCl_3$) δ 198.7 (C(1)), 146.1 (C(8a)), 136.2 (C(4a)), 134.6 (C(Ar)), 131.6 (C(7)), 127.4 (C(Ar) x2), 36.5 (C(2)), 32.5 (C(4)), 30.7 (C(3)), 20.9 (C(10)), 20.7 (C(9)).

HRMS (ESI): m/z calculated for $C_{12}H_{15}O^+$ $[M+H]^+$ 175.1117 found 175.1117.

IR (CH_3Cl film): 3023 (w), 2927 (w), 1684 (s), 1612 (w), 1495 (w), 1458 (w), 1409 (w), 1304 (m), 1181 (m), 943 (w), 817 (w), 754 (w) cm^{-1}.

SFC: Chiralpak® IG, 1500 psi, 30 °C; flow: 1.0 mL/min; 0% MeOH for 3 min, 0% to 6% MeOH over 3 min, 6% to 9% MeOH over 10 min, 98:2 e.r. (minor enantiomer t_R = 7.66 min, major enantiomer t_R = 7.72 min).

α_D^{25} = –9.3 (c = 1.0, $CHCl_3$) (Lit[21].: α_D^{25} = −10.0 (c = 1.0, $CHCl_3$)).

6. Reaction optimisation

6.1. Screening of reaction additives

Table 6.1 Additive screening.

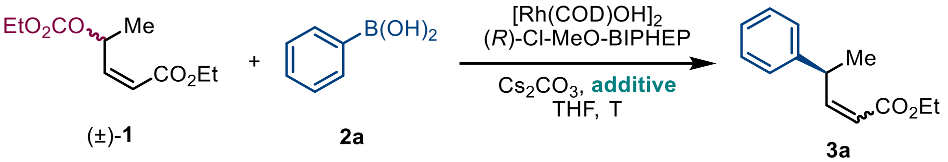

Entry	Additive	T (°C)	*ee* of ***Z*** / %	NMR yield of ***Z*** / %	NMR yield of ***E*** / %	Total yield/%	Ratio (***Z***:***E***)	SM / %
1	AgOTf	60	98	29	7	36	4.1	58
2	$Er(OTf)_3$	60	97	65	24	89	2.7	0
3	$Yt(OTf)_3$	60	97	62	25	87	2.5	0
4	$Zn(OTf)_2$	60	97	67	25	92	2.7	0
5	$ZnCl_2$	60	-	0	0	0	-	85
6	$ZnBr_2$	60	-	0	0	0	-	89
7	ZnI_2	60	-	0	0	0	-	100
8	$ZnCl_2$ (in Et_2O)	60	98	56	19	75	2.9	26
9	$AgNTf_2$	60	96	57	23	80	2.5	13
10	$AgSbF_6$	60	98	42	9	51	4.7	47
11	$AgBF_4$	60	98	50	14	64	3.6	36
12	$AgPF_6$	60	99	42	11	53	3.8	43
13	$AgIO_4$	60	-	0	0	0	-	0
14	$AgClO_4$	60	96	57	17	74	3.4	10
15	TfOH	60	97	55	18	73	3.1	0
16	$Cu(OTf)_2$	60	97	34	8	42	4.3	5
17	$Zn(OTf)_2$	r.t.	98	75	19	94	4.0	0
18	AgOTf	r.t.	98	28	7	36	4.1	54
19	TfOH	r.t.	97	55	18	73	3.1	20

Conditions: $[Rh(COD)OH]_2$ (2.5 mol %), (*R*)-Cl-MeO-BIPHEP (6.0 mol %), Cs_2CO_3 (1.0 equiv.), additive (0.2 equiv.), phenylboronic acid (2.0 equiv.), 16 h.

6.2. Variation of additive equivalents

Table 6.2 Effect of $Zn(OTf)_2$ equivalents on the reaction outcome.

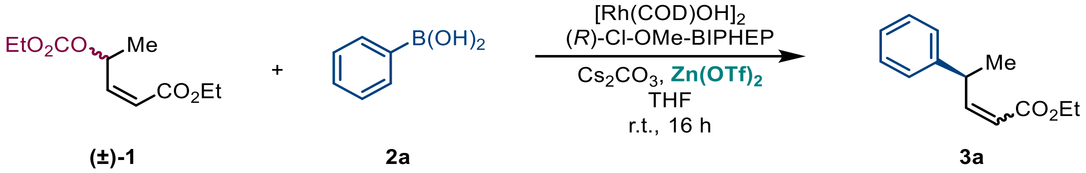

Entry	$Zn(OTf)_2$ equiv.	yield of **3a** / %	*ee* of **3a** / %	*Z*/*E* of **3a**
1	0.1 equiv.	89	97	3.9 : 1
2	0.2 equiv.	94	98	4.0 : 1
3	0.5 equiv.	91	98	3.4 : 1
4	1 equiv.	56	85	2.6 : 1

Reaction conditions: $[Rh(cod)OH]_2$ (2.5 mol %), (*R*)-Cl-MeO-BIPHEP (6.0 mol %), (±)-**1** (0.4 mmol, 1.0 equiv.), **2a** (2.0 equiv.), Cs_2CO_3 (1.0 equiv.), $Zn(OTf)_2$, THF (0.1 M), r.t., 16 h. All experiments were performed on 0.20 mmol scale. All compounds were isolated as single regioisomers (rr > 99:1). Enantiomeric ratios were determined by subsequent hydrogenation of the product mixture and SFC analysis on a chiral non-racemic stationary phase.

6.3. Variation of base

Table 6.3 Effect varying base on the reaction outcome.

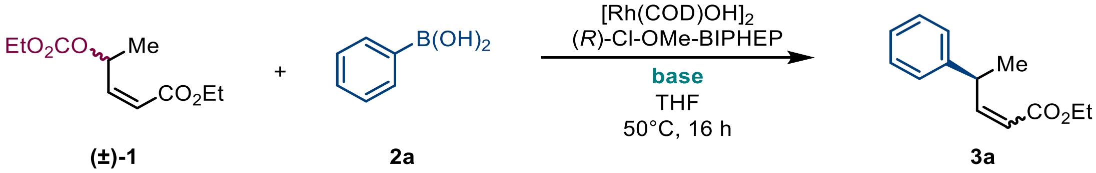

Entry	Base	yield of **3a** / %	*ee* of **3a** / %	*Z*/*E* of **3a**
1	Cs_2CO_3	82	98	3.4 : 1
2	CsOH (50wt% aq.)	5	97	N/A
3	K_3PO_4	63	97	3.2 : 1
4	KOAc	35	96	3.8 : 1
5	NaOMe	25	96	3.2 : 1
6	KO^tBu	18	95	2.5 : 1
7	KOH	19	95	3.7 : 1
8	NEt_3	0	N/A	N/A

Reaction conditions: $[Rh(cod)OH]_2$ (2.5 mol %), (*R*)-Cl-MeO-BIPHEP (6.0 mol %), (±)-**1** (0.4 mmol, 1.0 equiv.), **2a** (2.0 equiv.), base (1.0 equiv.), THF (0.1 M), 50°C, 16 h. All experiments were performed on 0.20 mmol scale. All compounds were isolated as single regioisomers (rr > 99:1). Enantiomeric ratios were determined by subsequent hydrogenation of the product mixture and SFC analysis on a chiral non-racemic stationary phase.

7. Optimisation of Rh-catalyzed 1,4-addition to *E*-3d

Table 7.1 Optimization of 1,4-addition to obtain **7**.

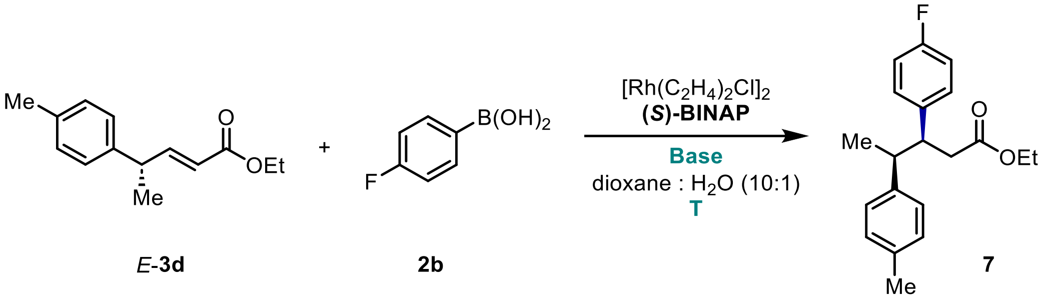

No.	Base	T / °C	Equiv. of **2b**	Conversion / %	d.r.
1	None	r.t.	3	-	-
2	None	60	3	-	-
3	KOH (0.5 eq.)	r.t.	3	-	-
4	KOH (0.5 eq.)	60	3	81 (78)	>20:1
5	KOH (0.5 eq.)	80	3	67	>20:1
6	KOH (1 eq.)	60	3	55	>20:1
7	KOH (0.5 eq.)	60	5	46	>20:1

Table 7.2 Optimization of 1,4-addition to obtain **8**.

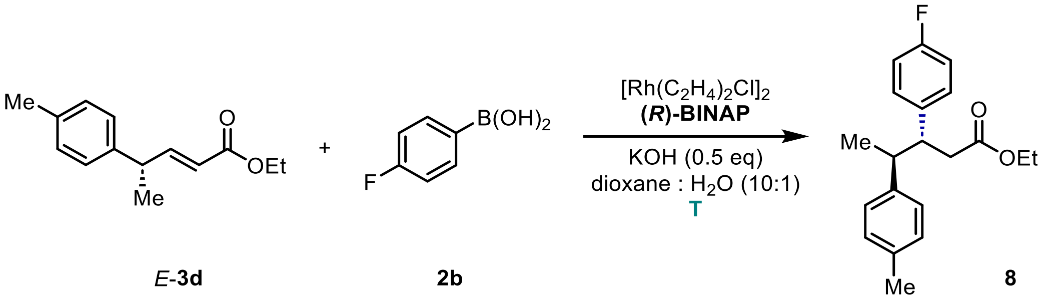

No.	Rh / %	T / °C	Equiv. of **2b**	Conversion / %	d.r.
1	2.5	60	3	12	>20:1
2	5	60	3	37	>20:1
3	5	80	3	72 (68)	>20:1
4	5	80	5	65	>20:1

8. Procedures for the synthesis of starting materials used in mechanistic studies

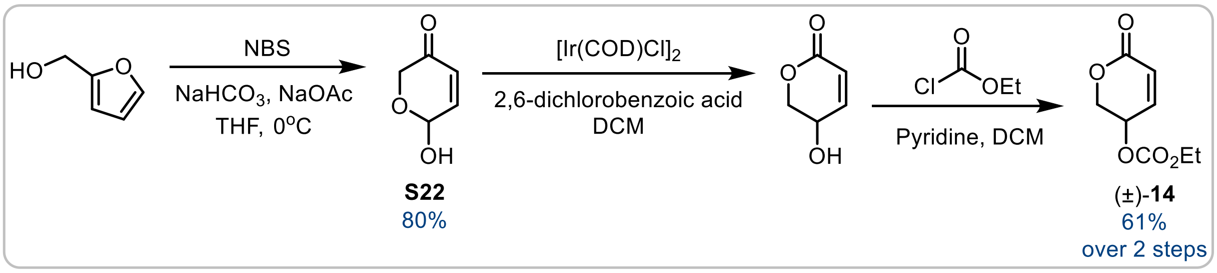

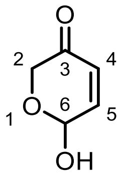

6-hydroxy-2*H*-pyran-3(6*H*)-one S22

Following a procedure by Waldmann[23], NBS (4.58 g, 25.7 mmol, 1.05 equiv.) was added to a solution of furfuryl alcohol (2.13 mL, 24.5 mmol, 1.0 equiv.), $NaHCO_3$ (4.12 g, 49.0 mmol, 2.0 equiv.) and NaOAc (2.01 g, 24.5 mmol, 1.0 equiv.) in THF/H_2O (4/1, 40 ml) at 0 °C. After 10 min, the reaction mixture was extracted with EtOAc (x3). The combined organic phases were dried over Na_2SO_4, filtered and concentrated *in vacuo*. Purification by automated medium-pressure chromatography (EtOAc/hexane = 10/90 to 60/40) afforded 6-hydroxy-2*H*-pyran-3(6*H*)-one **S22** (2.24 g, 19.6 mmol, 80%) as a white solid. Characterisation data match literature reports.[24]

^{1}H NMR (400 MHz, $CDCl_3$) δ 6.95 (dd, *J* = 10.4, 3.0 Hz, 1H, C(5)-H), 6.16 (dd, *J* = 10.4, 1.1 Hz, 1H, C(4)-H), 5.63 (m, 1H, C(6)-H), 4.56 (dd, *J* = 16.9, 1.1 Hz, 1H, C(2)-H), 4.13 (dd, *J* = 16.5, 0.8 Hz, 1H, C(2)-H), 3.53 (br s, 1H, -OH).

^{13}C NMR (101 MHz, $CDCl_3$) δ 195.2 (C(3)), 146.4 (C(5)), 127.7 (C(4)), 88.2 (C(6)), 66.6 (C(2)).

HRMS (ESI): m/z calculated for $C_5H_5O_3^-$ [M-H]$^-$ 113.0244 found 113.0244.

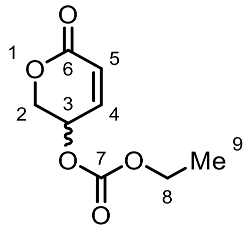

ethyl (6-oxo-3,6-dihydro-2*H*-pyran-3-yl) carbonate (±)-14

Following a procedure by Tang[25], 6-hydroxy-2*H*-pyran-3(6*H*)-one **S22** (2.24 g, 19.6 mmol, 1.0 equiv.), [Ir(COD)Cl$_2$] (329.7 mg, 0.50 mmol, 0.025 equiv.) and 2,6-dichlorobenzoic acid (1.88 mg, 9.8 mmol, 0.5 equiv.) were dissolved in anhydrous $CHCl_3$ (196 mL) under argon. The reaction mixture was stirred at room temperature (23°C) for 3 hours and the solvent was removed under reduced pressure. Purification by automated medium-pressure chromatography (EtOAc/hexane = 20/80 to 80/20) afforded 5-hydroxy-5,6-dihydro-2*H*-pyran-2-one (1.95 g, 17.1 mmol, 87%) as a yellow oil.

5-hydroxy-5,6-dihydro-2*H*-pyran-2-one (1.95 mg, 17.1 mmol, 1.0 equiv.) and ethyl chloroformate (1.79 mL, 18.8 mmol, 1.1 equiv.) were dissolved in dry CH_2Cl_2 (85 mL). Pyridine (1.65 mL, 20.5 mmol, 1.2 equiv.) was added dropwise at 0 °C. The reaction mixture was stirred at room temperature (23°C) for 4 hours and then quenched with 1M $KHSO_4$ solution. The aqueous layer was extracted with DCM (x3). Combined organic layers were washed with saturated aqueous $NaHCO_3$ and brine, dried over Na_2SO_4, filtered and concentrated *in vacuo*. Purification by medium-pressure automated flash chromatography (EtOAc/hexane = 10/90 to 60/40) afforded ethyl ethyl (6-oxo-3,6-dihydro-2*H*-pyran-3-yl) carbonate (±)-**14** (2.08 g, 11.2 mmol, 70%) as a yellow oil.

^{1}H NMR (400 MHz, $CDCl_3$) δ 6.95 (ddt, *J* = 9.9, 4.8, 0.8 Hz, 1H, C(4)-H), 6.20 (ddt, *J* = 9.8, 1.7, 0.7 Hz, 1H, C(5)-H), 5.22 (dtt, *J* = 4.6, 3.8, 0.8 Hz, 1H, C(3)-H), 4.55 (dt, *J* = 3.8, 0.8 Hz, 2H, C(2)-H$_2$), 4.23 (qt, *J* = 7.1, 1.1 Hz, 2H, C(8)-H$_2$), 1.32 (tt, *J* = 7.1, 1.1 Hz, 3H, C(9)-H$_3$).

^{13}C NMR (101 MHz, $CDCl_3$) δ 161.6 (C(6)), 154.1 (C(7)), 140.4 (C(4)), 124.9 (C(5)), 68.8 (C(3)), 64.9 (C(2) and C(8)), 14.1 (C(9)).

IR (CH_3Cl film): 2982 (w), 1734 (s), 1372 (m), 1250 (s), 1103 (m), 1061 (m), 1006 (m), 841 (m), 788 (m) cm^{-1}.

HRMS (ESI): m/z calculated for $C_8H_{10}O_5Na^+$ [M+Na]$^+$ 209.0420 found 209.0421.

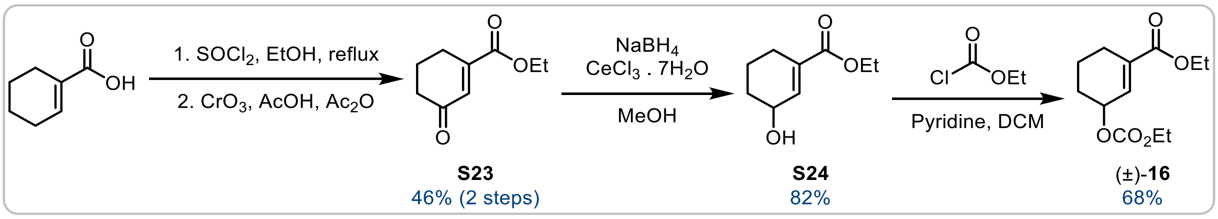

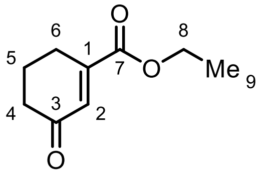

ethyl 3-oxocyclohex-1-ene-1-carboxylate S23

Thionyl chloride (0.18 mL, 2.5 mmol, 0.5 equiv.) was added to a solution of cyclohex-1-ene-1-carboxylic acid (0.57 mL, 5.0 mmol, 1.0 equiv.) in anhydrous EtOH (10 mL). The reaction mixture was refluxed for 5 hours and concentrated *in vacuo*. Filtration through a plug of silica afforded ethyl cyclohex-1-ene-1-carboxylate (740.2 mg, 4.8 mmol, 96%) as a colourless oil, and it was used in the next step without further purification.

A solution of CrO_3 (1.32 g, 13.2 mmol, 2.8 equiv.) in acetic acid (2.75 mL) and acetic anhydride (1.25 mL) was added to a solution of ethyl ethyl cyclohex-1-ene-1-carboxylate (740.2 mg, 4.8 mmol, 1.0 equiv.) in DCM (9.6 mL) dropwise at 0°C. The reaction mixture was stirred at room temperature (23°C) for 2 hours and then quenched with 1M KOH solution at 0°C. The aqueous layer was extracted with DCM (x3). Combined organic layers were washed with water and brine, dried over Na_2SO_4, filtered and concentrated *in vacuo*. Purification by medium-pressure automated flash chromatography (Et_2O/hexane = 10/90 to 50/50) afforded ethyl 3-oxocyclohex-1-ene-1-carboxylate **S23** (384.4 mg, 2.3 mmol, 48%) as a colourless oil. Characterisation data match literature reports.[26]

^{1}H NMR (400 MHz, $CDCl_3$) δ 6.74 (t, *J* = 1.9 Hz, 1H, C(2)-H), 4.27 (q, *J* = 7.1 Hz, 2H, C(8)-H_2), 2.58 (td, *J* = 6.0, 1.9 Hz, 2H, C(4)-H_2), 2.45 (m, 2H, C(6)-H_2), 2.06 (m, 2H, C(5)-H_2), 1.32 (t, *J* = 7.1 Hz, 3H, C(9)-H_3).

^{13}C NMR (101 MHz, $CDCl_3$) δ 200.2 (C(3)), 166.5 (C(7)), 149.2 (C(1)), 132.9 (C(2)), 61.7 (C(8)), 37.7 (C(4)), 24.8 (C(6)), 22.2 (C(5)), 14.1 (C(9)).

HRMS (ESI): m/z calculated for $C_9H_{13}O_3{}^+$ $[M+H]^+$ 169.0859 found 169.0860.

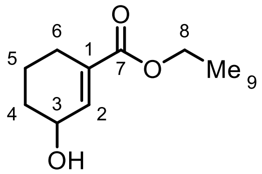

ethyl 3-hydroxycyclohex-1-ene-1-carboxylate S24

A solution of $NaBH_4$ (86.5 g, 2.3 mmol, 1.0 equiv.) in MeOH (5 mL) was added to a solution of ethyl 3-oxocyclohex-1-ene-1-carboxylate **S23** (384.4 mg, 2.3 mmol, 1.0 equiv.) and $CeCl_3 \cdot 7H_2O$ (600.0 mg, 2.3 mmol, 1.0 equiv.) in MeOH (10 mL) dropwise at 0°C. The reaction mixture was stirred at room temperature (23°C) for 2 hours and then quenched with 1M HCl solution at 0°C. The aqueous layer was extracted with Et_2O (x3). Combined organic layers were washed with brine, dried over Na_2SO_4, filtered and concentrated *in vacuo*. Purification by medium-pressure automated flash chromatography (EtOAc/hexane = 0/100 to 40/60) afforded ethyl 3-hydroxycyclohex-1-ene-1-carboxylate **S24** (321.0 mg, 1.9 mmol, 82%) as a colourless oil. Characterisation data match literature reports.[27]

^{1}H NMR (400 MHz, $CDCl_3$) δ 6.86 (dtd, *J* = 2.7, 1.9, 0.7 Hz, 1H, C(2)-H), 4.35 (d, *J* = 8.4 Hz, 1H, C(3)-H), 4.19 (q, *J* = 7.1 Hz, 2H, C(8)-H_2), 2.25 (m, 2H, C(6)-H_2), 1.86 (m, 3H, C(4)-H_3 and -OH), 1.58 (m, 2H, C(5)-H_2), 1.28 (t, *J* = 7.1 Hz, 3H, C(9)-H_3).

^{13}C NMR (101 MHz, $CDCl_3$) δ 167.3 (C(7)), 139.5 (C(2)), 132.7 (C(1)), 66.0 (C(3)), 60.6 (C(8)), 31.2 (C(6)), 24.2 (C(4)), 19.1 (C(5)), 14.2 (C(9)).

HRMS (ESI): m/z calculated for $C_9H_{15}O_3^+$ $[M+H]^+$ 171.1016 found 171.1016.

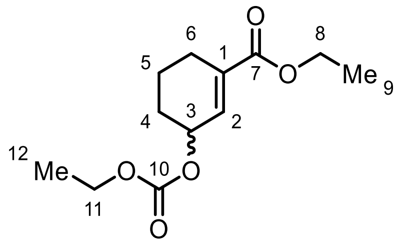

ethyl 3-((ethoxycarbonyl)oxy)cyclohex-1-ene-1-carboxylate (±)-16

Ethyl 3-hydroxycyclohex-1-ene-1-carboxylate **S18** (321.0 mg, 1.9 mmol, 1.0 equiv.) and ethyl chlorofomate (0.20 mL, 2.1 mmol, 1.1 equiv.) were dissolved in DCM (15 mL). Pyridine (0.18 mL, 2.3 mmol, 1.2 equiv.) was added dropwise at 0°C. The mixture was stirred at room temperature (23°C) for 3 hours and then quenched with 1M $KHSO_4$ solution. The aqueous layer was extracted with DCM (x3). Combined organic layers were washed with saturated aqueous $NaHCO_3$ and brine, dried over Na_2SO_4, filtered and concentrated *in vacuo* at 0°C. Purification by medium-pressure automated flash chromatography (EtOAc/hexane = 0/100 to 55/45) afforded ethyl 3-((ethoxycarbonyl)oxy)cyclohex-1-ene-1-carboxylate (±)-**16** (313.0 mg, 1.3 mmol, 68%) as a colourless oil.

^{1}H NMR (400 MHz, $CDCl_3$) δ 6.85 (dt, *J* = 3.7, 2.0 Hz, 1H, C(2)-H), 5.25 (dt, *J* = 8.5, 3.2 Hz, 1H, C(3)-H), 4.20 (*app* qd, *J* = 7.1, 4.4 Hz, 4H, C(8)-H_2 and C(11)-H_2), 2.33 (m, 1H, C(6)-H), 2.23 (m, 1H, C(6)-H), 1.93 (m, 1H, C(4)-H), 1.74 (m, 3H, C(4)-H and C(5)-H_2), 1.29 (*app* dt, *J* = 12.0, 7.1 Hz, 6H, C(9)-H_3 and C(12)-H_3).

^{13}C NMR (101 MHz, $CDCl_3$) δ 166.7 (C(7)), 154.7 (C(10)), 135.1 (C(1)), 134.4 (C(2)), 71.5 (C(3)), 64.1 (C(11)), 60.7 (C(8)), 27.5 (C(6)), 24.1 (C(4)), 18.8 (C(5)), 14.3 (C(9)), 14.2 (C(12)).

IR (CH_3Cl film): 2982 (w), 1742 (s), 1716 (s), 1373 (w), 1235 (s), 1084 (m), 1013 (m), 941 (w), 874 (w), 791 (m), 749 (m) cm^{-1}.

HRMS (ESI): m/z calculated for $C_{12}H_{18}O_5Na^+$ $[M+Na]^+$ 265.1046 found 265.1045.

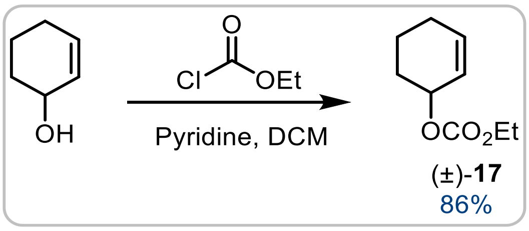

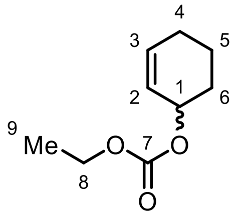

cyclohex-2-en-1-yl ethyl carbonate (±)-17

Cyclohex-2-en-1-ol (294.4 mg, 3.0 mmol, 1.0 equiv.) and ethyl chlorofomate (0.31 mL, 3.3 mmol, 1.1 equiv.) were dissolved in DCM (15 mL). Pyridine (0.29 mL, 3.6 mmol, 1.2 equiv.) was added dropwise at 0°C. The mixture was stirred at room temperature (23°C) for 2 hours and then quenched with 1M $KHSO_4$ solution. The aqueous layer was extracted with DCM (x3). Combined organic layers were washed with saturated aqueous $NaHCO_3$ and brine, dried over Na_2SO_4, filtered and concentrated *in vacuo* at 0°C. Purification by medium-pressure automated flash chromatography (EtOAc/hexane = 10/90 to 60/40) afforded cyclohex-2-en-1-yl ethyl carbonate (±)-**17** (439.1 mg, 2.58 mmol, 86%) as a colourless oil. Characterisation data match literature reports.[28]

^{1}H NMR (500 MHz, $CDCl_3$) δ 5.96 (dddd, *J* = 10.1, 4.3, 3.4, 1.2 Hz, 1H, C(2)-H), 5.77 (ddt, *J* = 10.1, 4.1, 2.2 Hz, 1H, C(3)-H), 5.11 (tdq, *J* = 5.2, 3.3, 1.7 Hz, 1H, C(1)-H), 4.18 (q, *J* = 7.1 Hz, 2H, C(8)-H_2), 2.08 (m, 1H), 1.98 (m, 1H), 1.82 (m, 3H), 1.63 (m, 1H), 1.30 (t, *J* = 7.1 Hz, 3H, C(9)-H_3).

^{13}C NMR (126 MHz, $CDCl_3$) δ 154.9 (C(7)), 133.3 (C(2)), 125.0 (C(3)), 71.6 (C(1)), 63.7 (C(8)), 28.2 (C(6)), 24.8 (C(4)), 18.6 (C(5)), 14.3 (C(9)).

HRMS (ESI): m/z calculated for $C_9H_{14}O_3Na^+$ $[M+Na]^+$ 193.0835 found 193.0836.

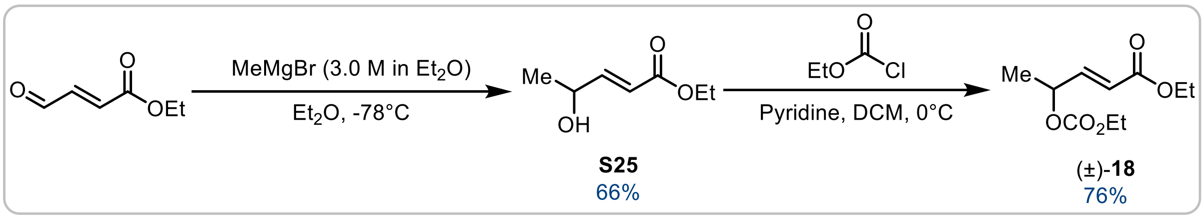

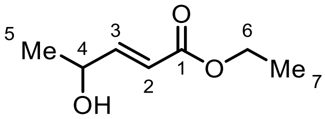

ethyl (E)-4-hydroxypent-2-enoate S25

Following a modified procedure by Fu[29], MeMgBr (3.0 M in Et2O, 13.3 mL, 44.0 mmol, 1.1 equiv.) was added dropwise to a solution of ethyl (E)-4-oxo-2-butenoate (5.13 g, 40.0 mmol, 1.0 equiv.) in Et_2O (100 mL) at –78 °C. The reaction mixture was stirred at –78°C for 3 hours. A saturated solution of NH_4Cl was added at 0°C, and the aqueous layer was extracted with Et_2O (x3). Combined organic layers were washed with brine, dried over Na_2SO_4, filtered and concentrated *in vacuo*. Purification by medium-pressure automated flash chromatography (Et_2O/hexane = 0/100 to 30/70) afforded ethyl (E)-4-hydroxypent-2-enoate **S25** (951.5 mg, 6.6 mmol, 66%) as a colourless oil. Characterisation data match literature reports.[30]

^{1}H NMR (400 MHz, $CDCl_3$) δ 6.96 (dd, *J* = 15.7, 4.7 Hz, 1H, C(3)-H), 6.02 (dd, *J* = 15.7, 1.7 Hz, 1H, C(2)-H), 4.49 (qtd, *J* = 6.6, 4.7, 1.7 Hz, 1H, C(4)-H), 4.20 (q, *J* = 7.1 Hz, 2H, C(6)-H_2), 1.67 (d, *J* = 4.8 Hz, 1H, -OH), 1.34 (d, *J* = 6.6 Hz, 3H, C(5)-H_3), 1.29 (t, *J* = 7.1 Hz, 3H, C(7)-H_3).

^{13}C NMR (126 MHz, $CDCl_3$) δ 166.7 (C(1)), 151.0 (C(2)), 119.6 (C(3)), 67.1 (C(4)), 60.5 (C(6)), 22.7 (C(5)), 14.2 (C(7)).

HRMS (ESI): m/z calculated for $C_7H_{13}O_3^+$ $[M+H]^+$ 145.0859 found 145.0859.

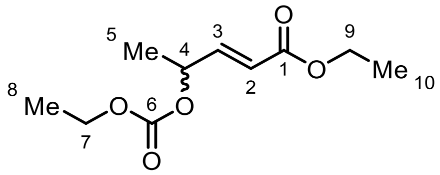

ethyl (*E*)-4-((ethoxycarbonyl)oxy)pent-2-enoate (±)-18

Ethyl (*E*)-4-hydroxypent-2-enoate **S25** (778.5 mg, 5.4 mmol, 1.0 equiv.) and ethyl chloroformate (0.57 mL, 5.9 mmol, 1.1 equiv.) were dissolved in DCM (25 mL). Pyridine (0.52 mL, 6.5 mmol, 1.2 equiv.) was added dropwise at 0°C. The mixture was stirred at room temperature (23°C) for 5 hours and then quenched with 1M $KHSO_4$ aqueous solution. The aqueous layer was extracted with DCM (x3). Combined organic layers were washed with saturated aqueous $NaHCO_3$ and brine, dried over Na_2SO_4, filtered and concentrated *in vacuo*. Purification by medium-pressure automated flash chromatography (Et_2O/hexane = 0/100 to 20/80) afforded ethyl (*E*)-4-((ethoxycarbonyl)oxy)pent-2-enoate (±)-**18** (887.4 mg, 4.10 mmol, 76%) as a colourless oil. Characterisation data match literature reports.[31]

^{1}H NMR (400 MHz, $CDCl_3$) δ 6.85 (dd, *J* = 15.8, 5.1 Hz, 1H, C(3)-H), 5.98 (dd, *J* = 15.8, 1.5 Hz, 1H, C(2)-H), 5.31 (qdd, *J* = 6.7, 5.1, 1.6 Hz, 1H, C(4)-H), 4.17 (q, *J* = 7.1 Hz, 4H, C(7)-H_2 and C(9)-H_2), 1.39 (d, *J* = 6.7 Hz, 3H, C(5)-H_3), 1.28 (*app* dt, *J* = 11.3, 7.1 Hz, 6H, C(8)-H_3 and C(10)-H_3).

^{13}C NMR (101 MHz, $CDCl_3$) δ 165.9 (C(1)), 154.2 (C(6)), 145.5 (C(3)), 121.4 (C(2)), 72.5 (C(4)), 64.1 (C(7)), 60.5 (C(9)), 19.7 (C(5)), 14.2 (C(8)), 14.1 (C(10)).

HRMS (ESI): m/z calculated for $C_{10}H_{16}O_5Na^+$ $[M+Na]^+$ 239.0890 found 239.0889.

9. Mechanistic studies

9.1. Reactions of enantiopure substrate (*S*)-1

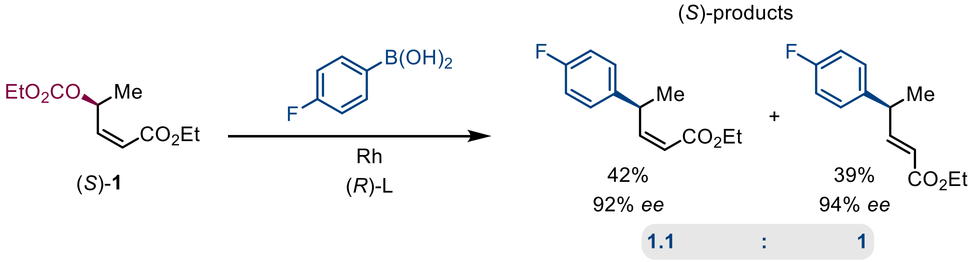

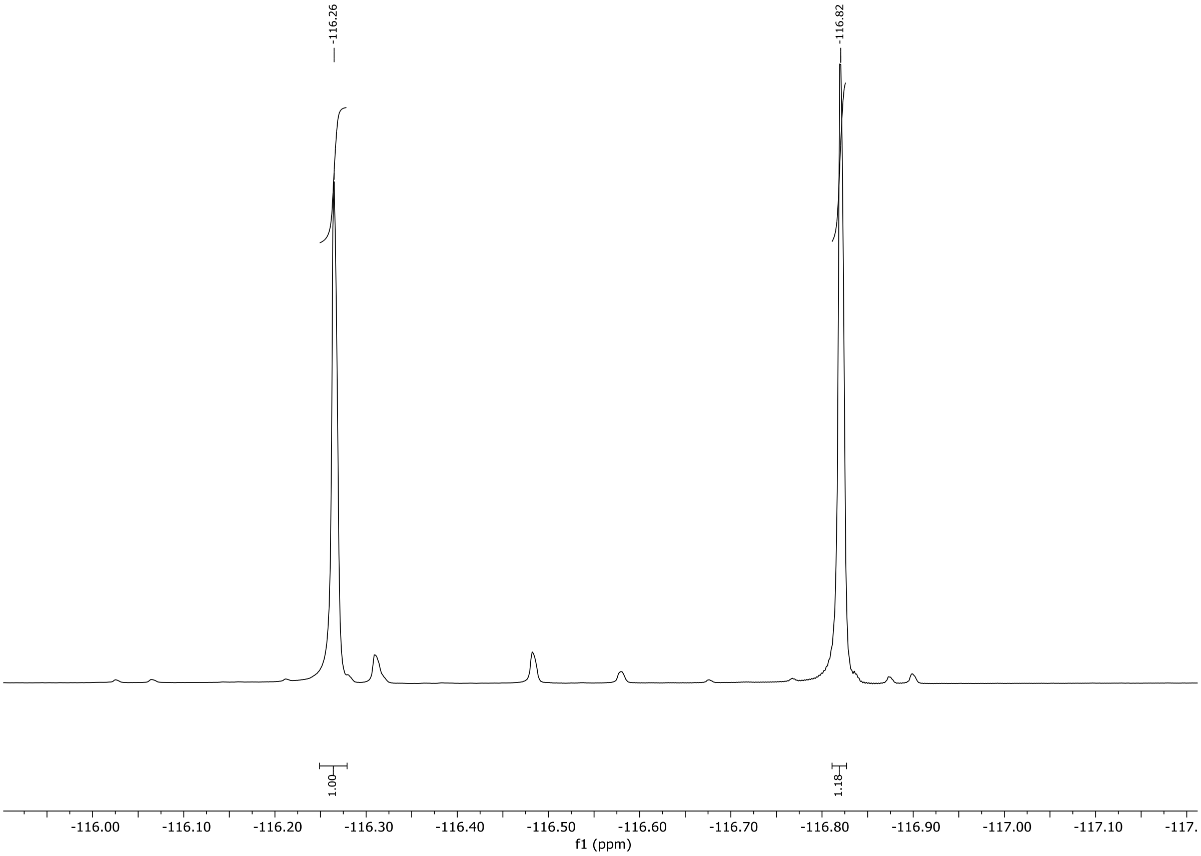

Figure 9.1 ^{19}F (^{13}C)NMR (376 MHz) spectrum of a crude reaction mixture obtained using (*S*)-**1** and (*R*)-Cl-MeO-BIPHEP.

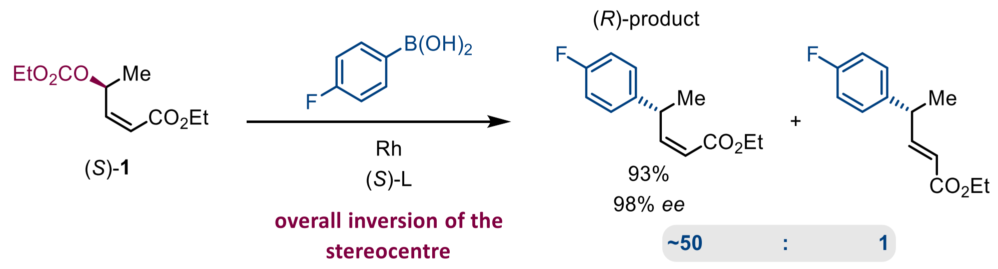

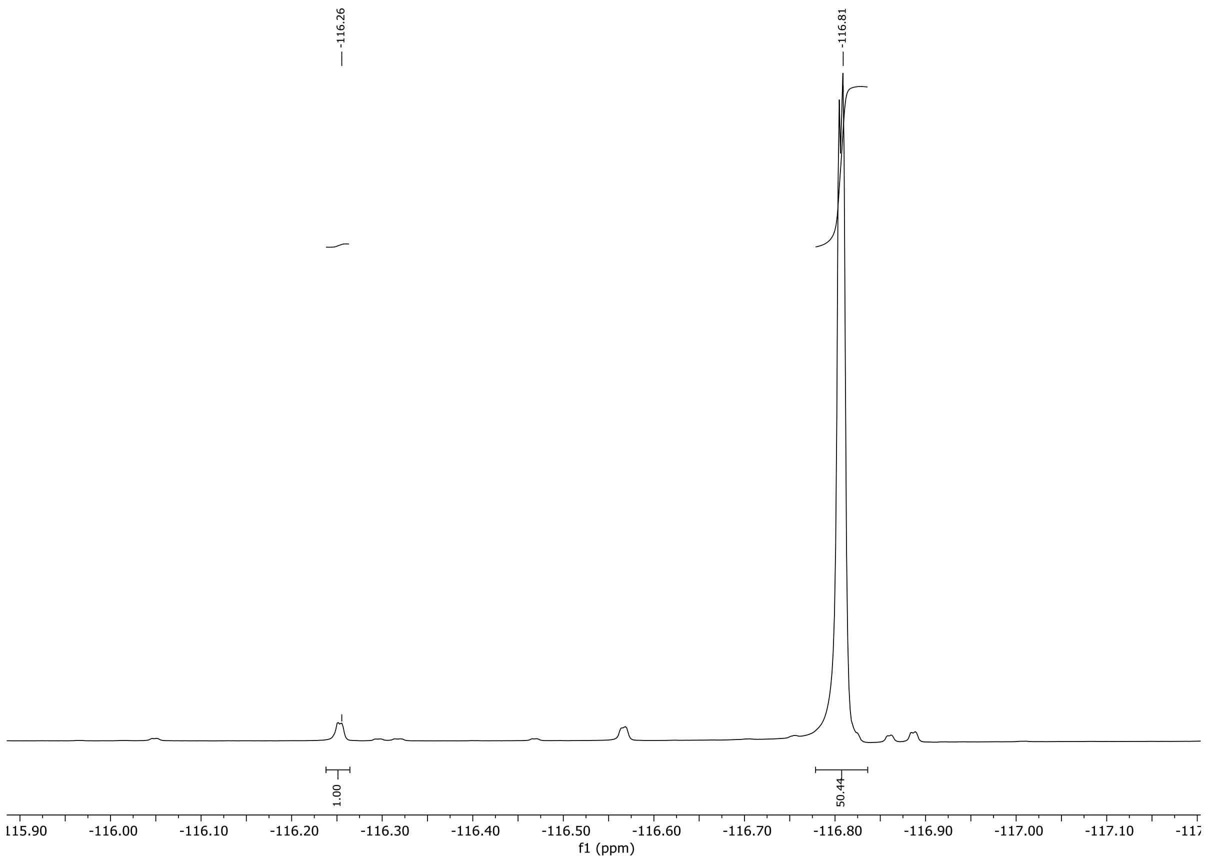

Figure 9.2 ^{19}F (^{13}C)NMR (376 MHz) spectrum of a crude reaction mixture obtained using (*S*)-**1** and (*S*)-Cl-MeO-BIPHEP.

9.2. (*S*)- and (*R*)-ligands result in opposite absolute stereochemistry

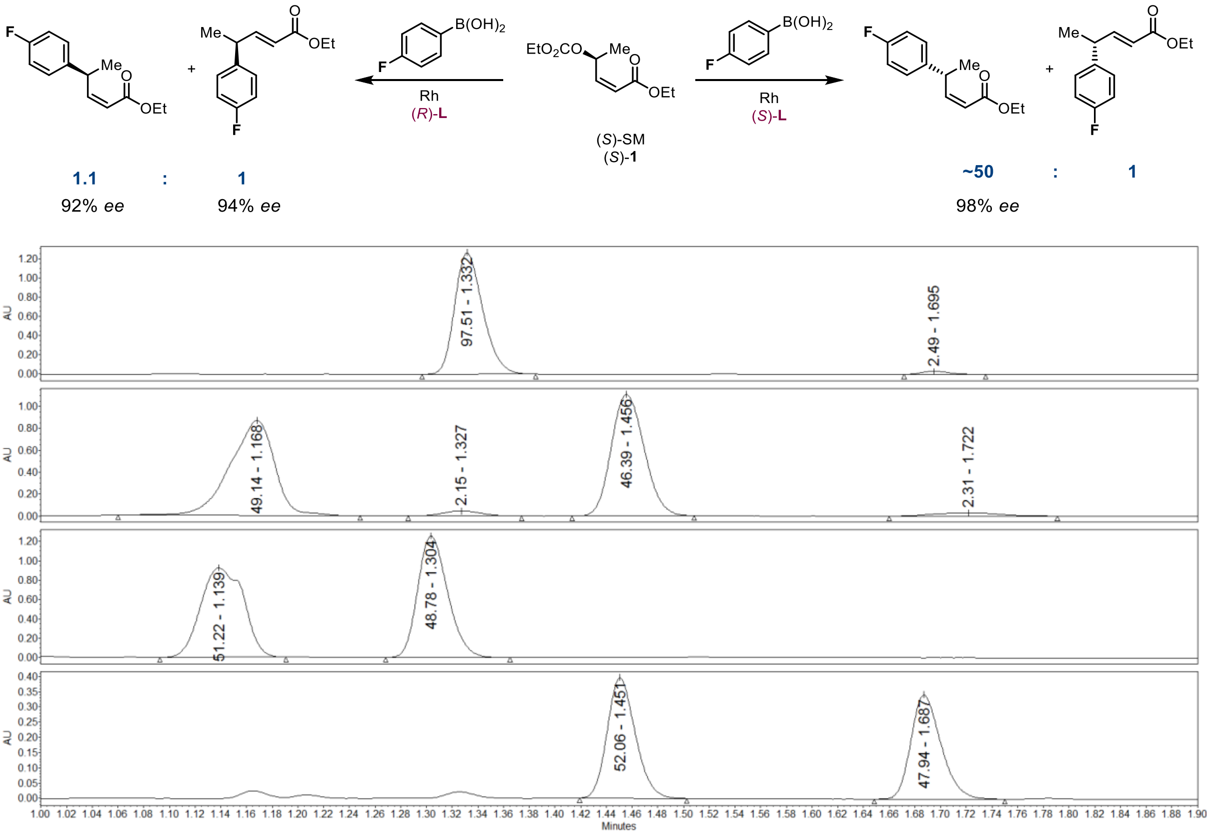

Figure 9.3 SFC traces comparing outcomes of reaction using enantiopure starting material (*S*)-**1** and ligands with opposite absolute stereochemistry. Reaction conditions: $[Rh(cod)OH]_2$ (2.5 mol%), Cl-MeO-BIPHEP (6.0 mol%), (*S*)-**1** (0.4 mmol, 1.0 equiv.), **2b** (2.0 equiv.), Cs_2CO_3 (1.0 equiv.), $Zn(OTf)_2$ (20 mol%), THF (0.1 M), r.t., 14 h.

9.3. Reaction monitoring

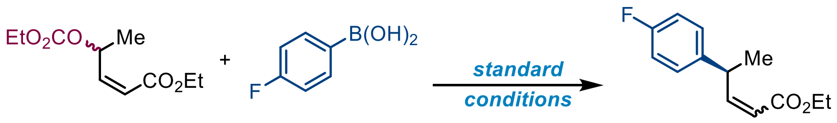

Time / min	Z/E of **3b**	**1**/*Z*-**3b**	*ee* of SM / %	Conversion / %
0	N/A	N/A	N/A	N/A
2	6.6	5.2	14	18.1
5	5.9	4.3	18	21.2
15	5.5	3.3	24	26.5
60	5	1.8	36	40.5
180	4.6	0.8	54	59.8
360	4.5	0.2	72	83.6

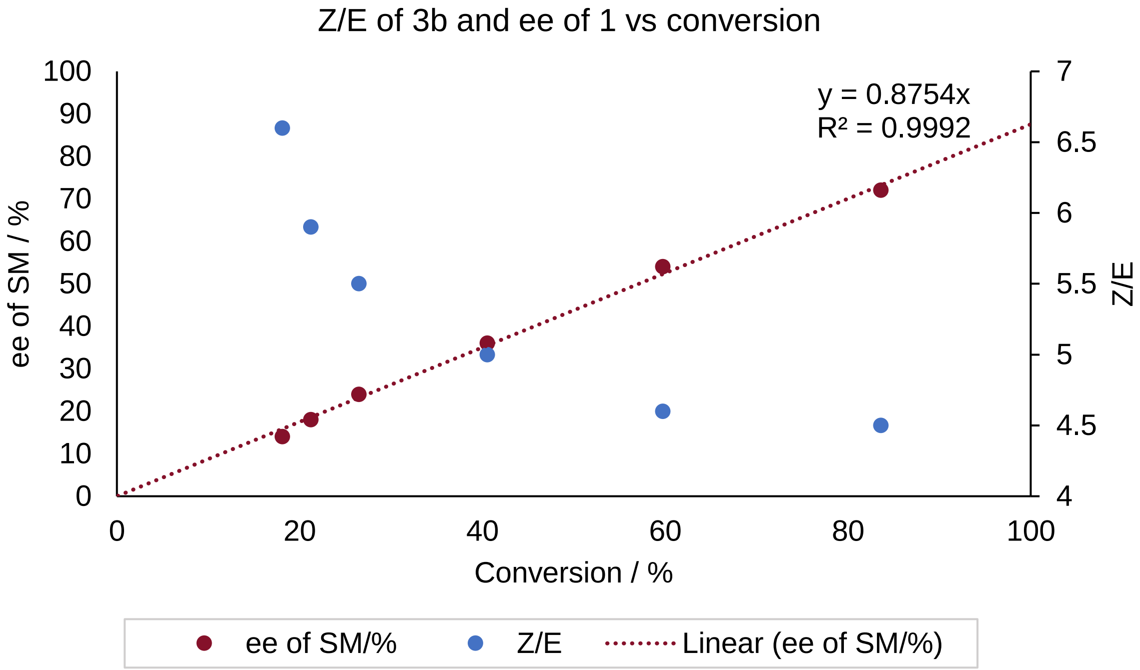

Figure 9.4 Changes of product *Z*/*E* ratio and *ee* of starting material in time. Reaction conditions: [Rh(cod)OH]$_2$ (2.5 mol%), (*R*)-Cl-MeO-BIPHEP (6.0 mol%), (*rac*)-**1** (0.4 mmol, 1.0 equiv.), **2b** (2.0 equiv.), Cs_2CO_3 (1.0 equiv.), $Zn(OTf)_2$ (20 mol%), THF (0.1 M), r.t.

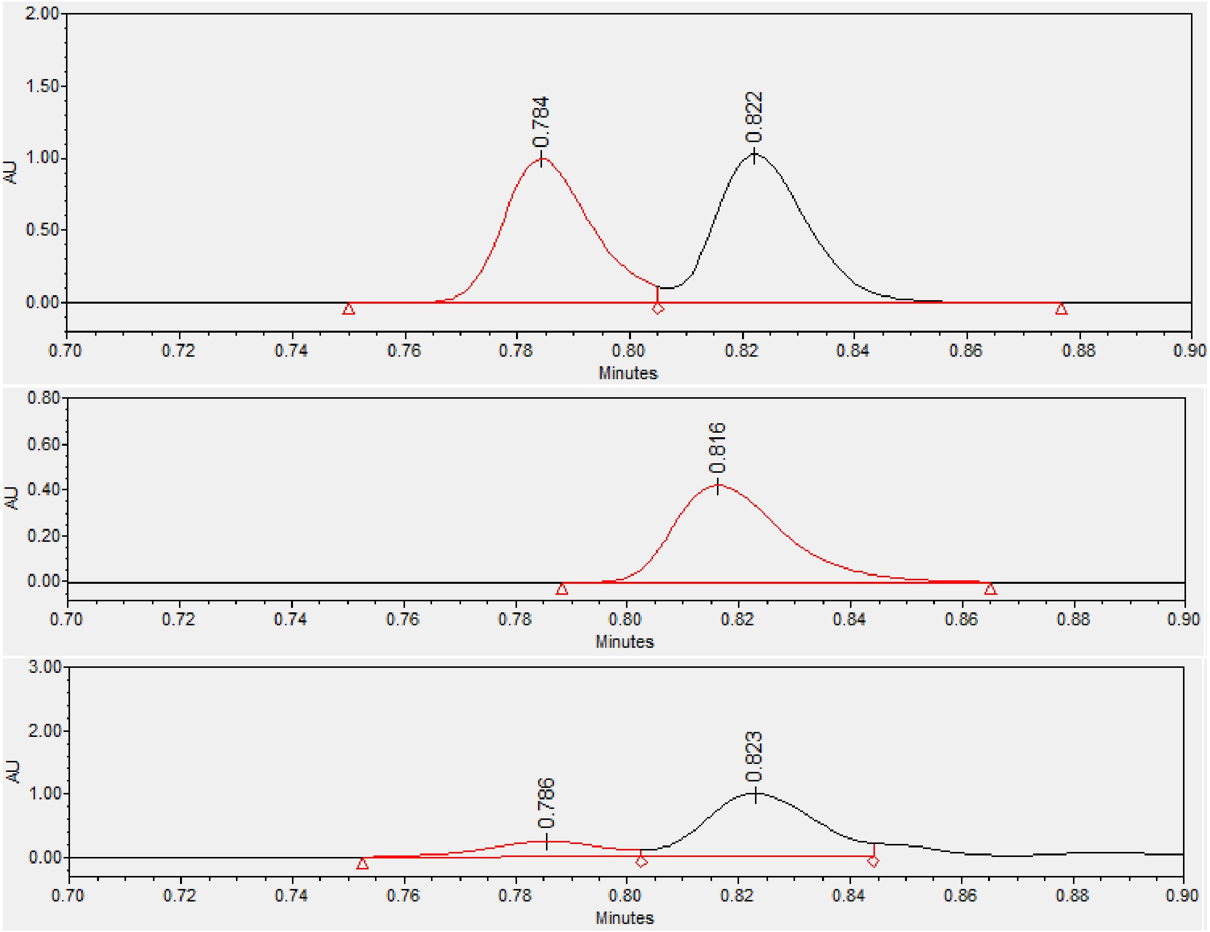

Figure 9.5 Monitoring the enantiomeric ratio of **1** during a reaction. Traces from top to bottom: a) *racemic* starting material b) enantiopure (*S*)-**1**. c) After ~1h, the starting material is enriched in the (*S*)-enantiomer.

10. NMR spectra

10.1. Spectra of the starting materials

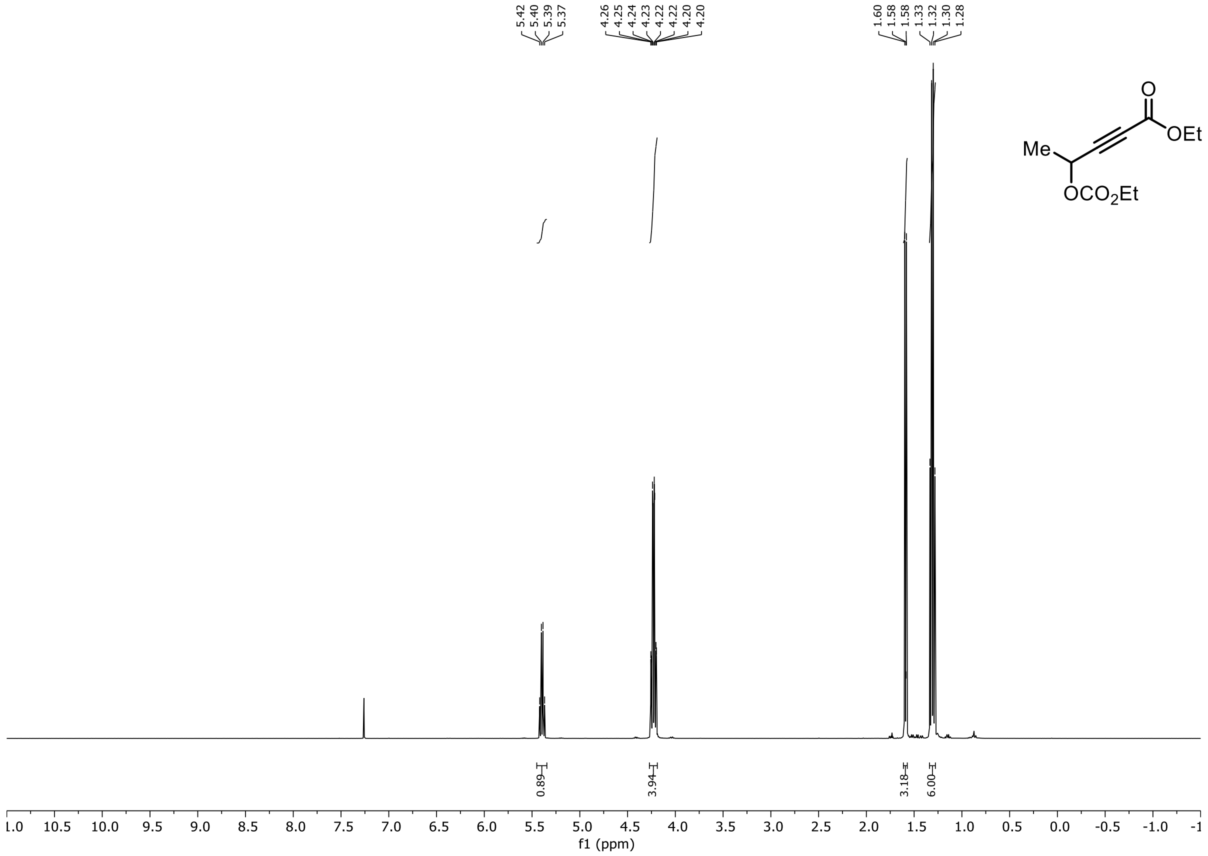

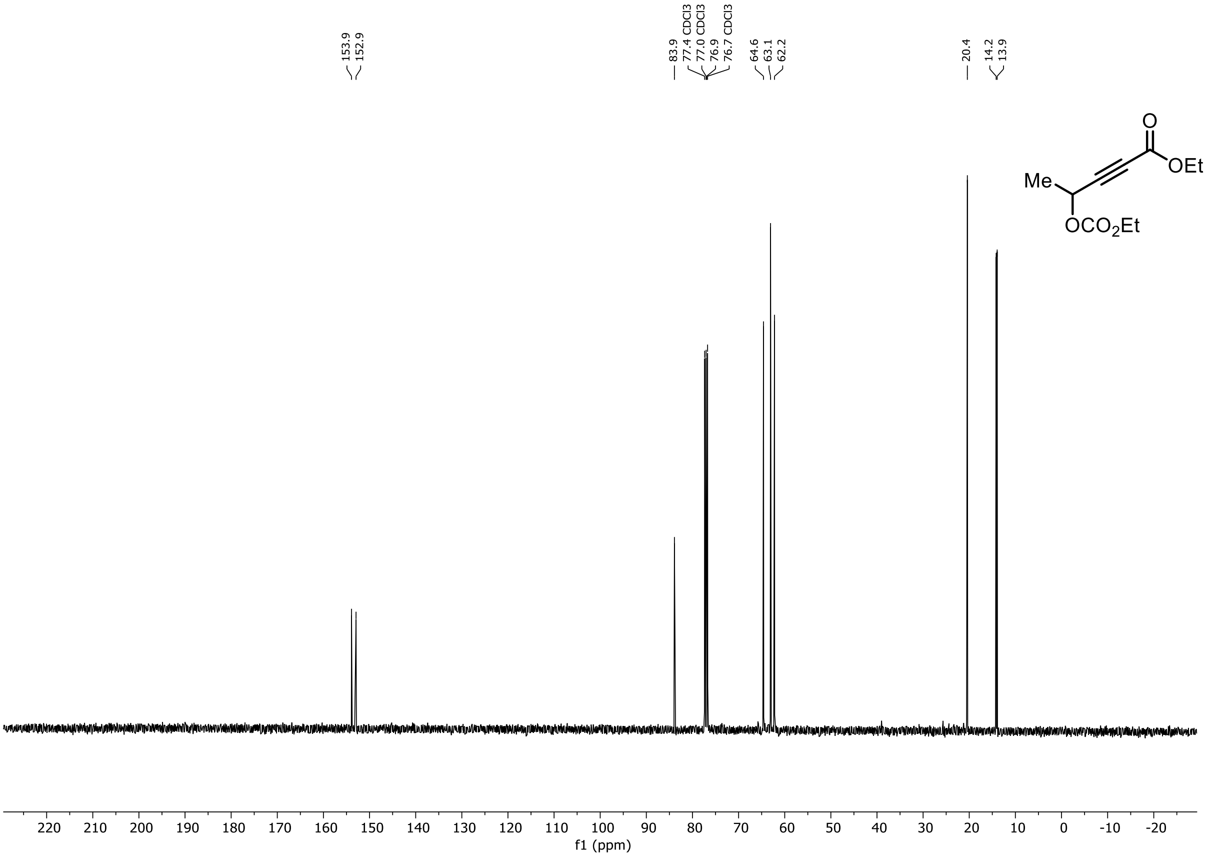

Figure 10.1 (top) ^{1}H NMR (400 MHz) and (bottom) ^{13}C NMR (101 MHz) spectra of **S1**.

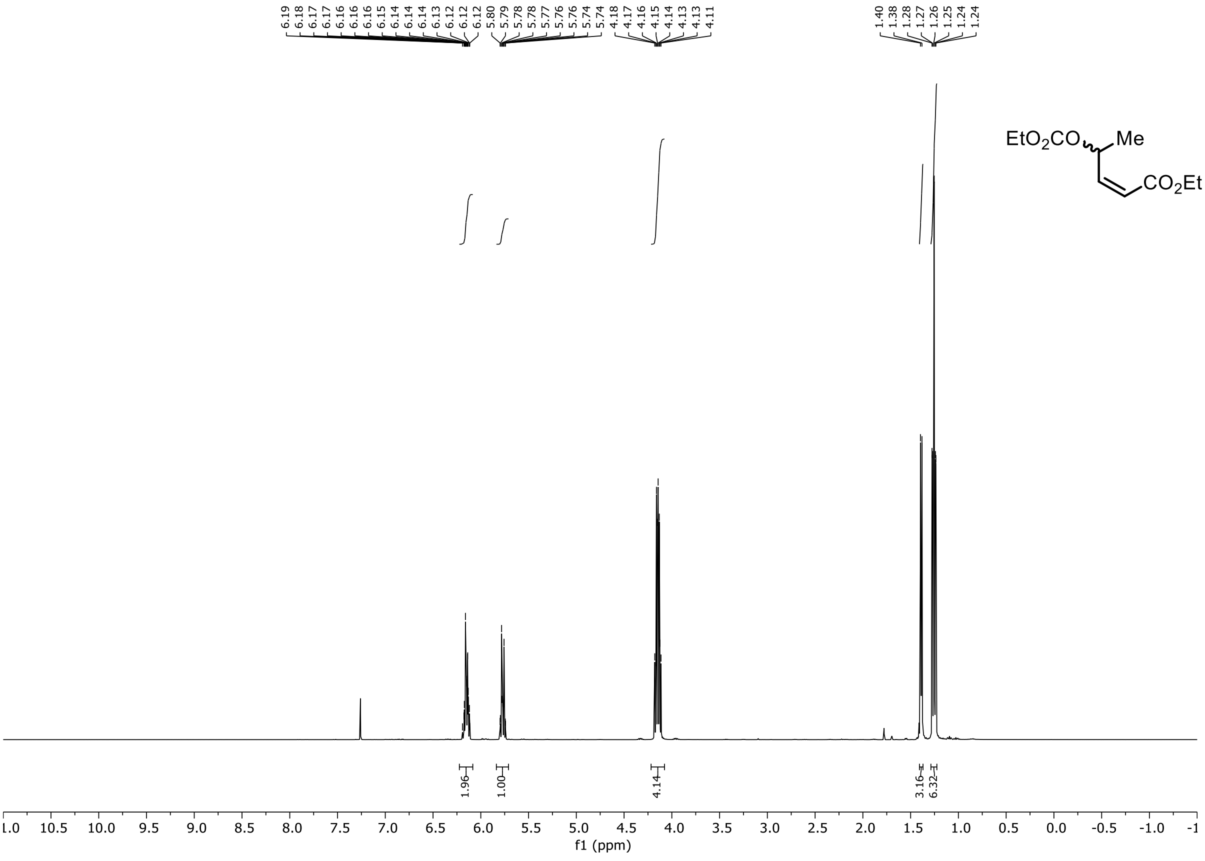

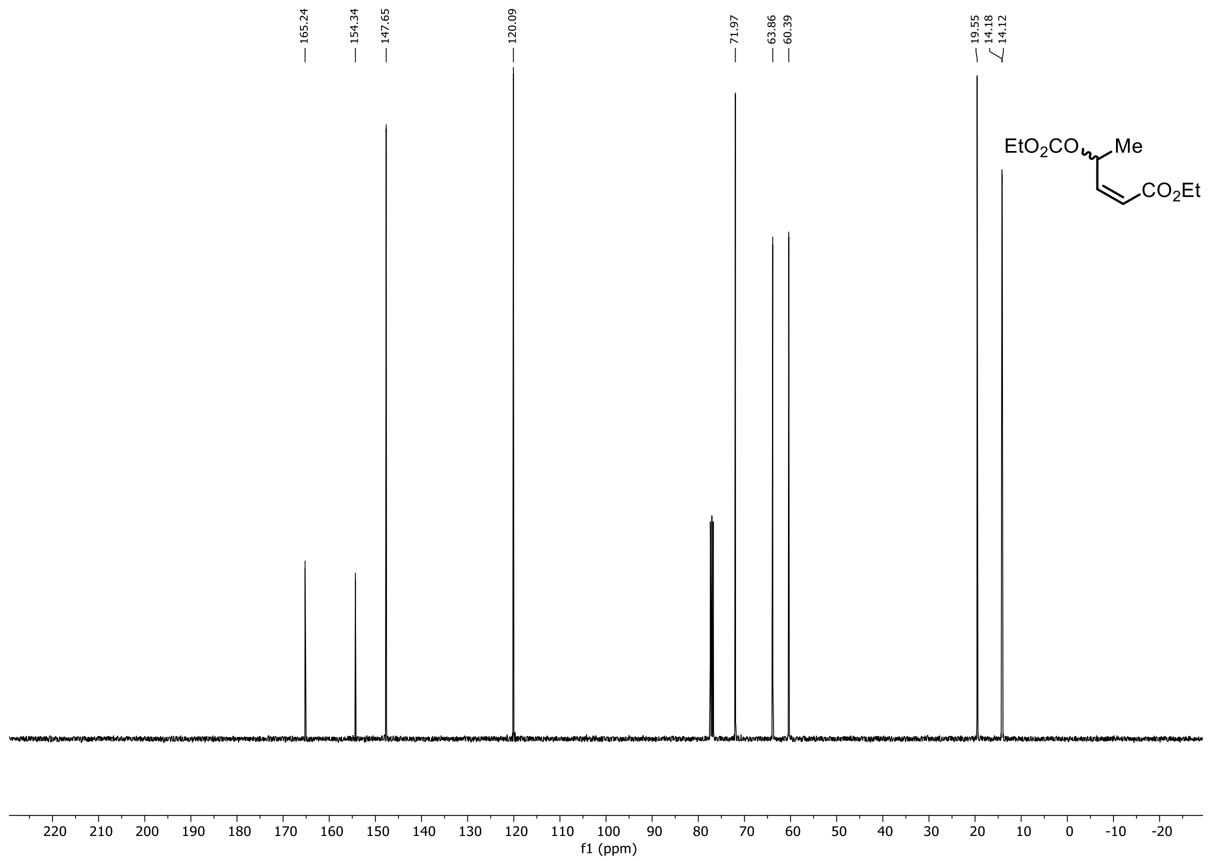

Figure 10.2 (top) ^{1}H NMR (400 MHz) and (bottom) ^{13}C NMR (101 MHz) spectra of (±)-**1**.

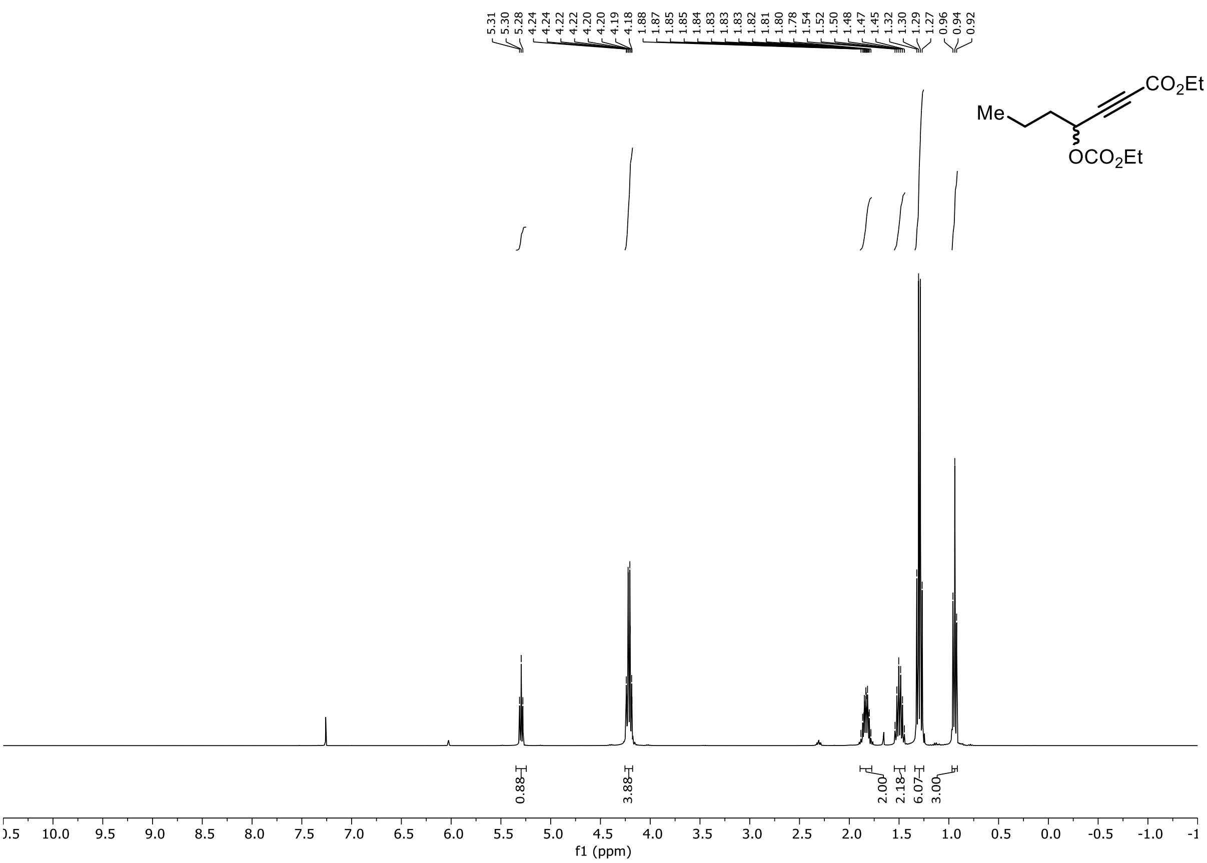

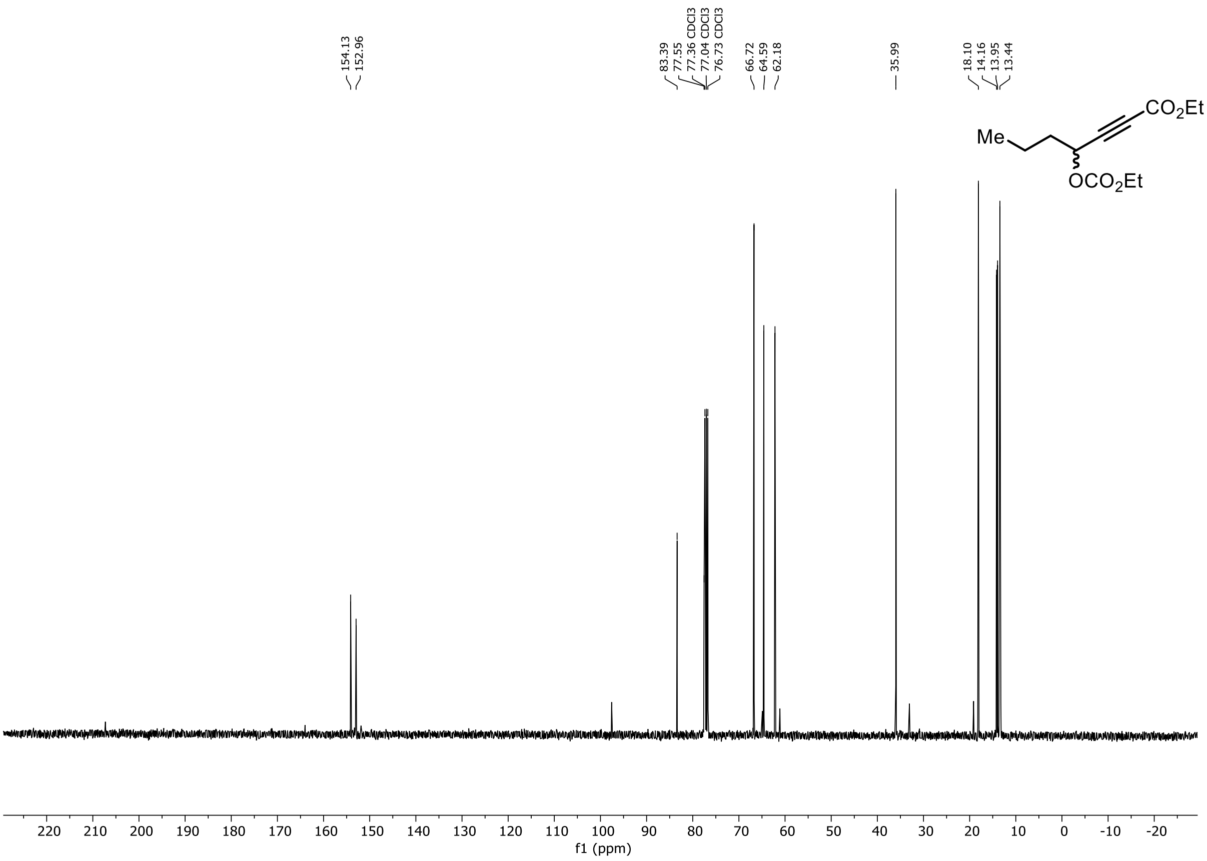

Figure 10.3 (top) ^{1}H NMR (400 MHz) and (bottom) ^{13}C NMR (101 MHz) spectra of **S2**.

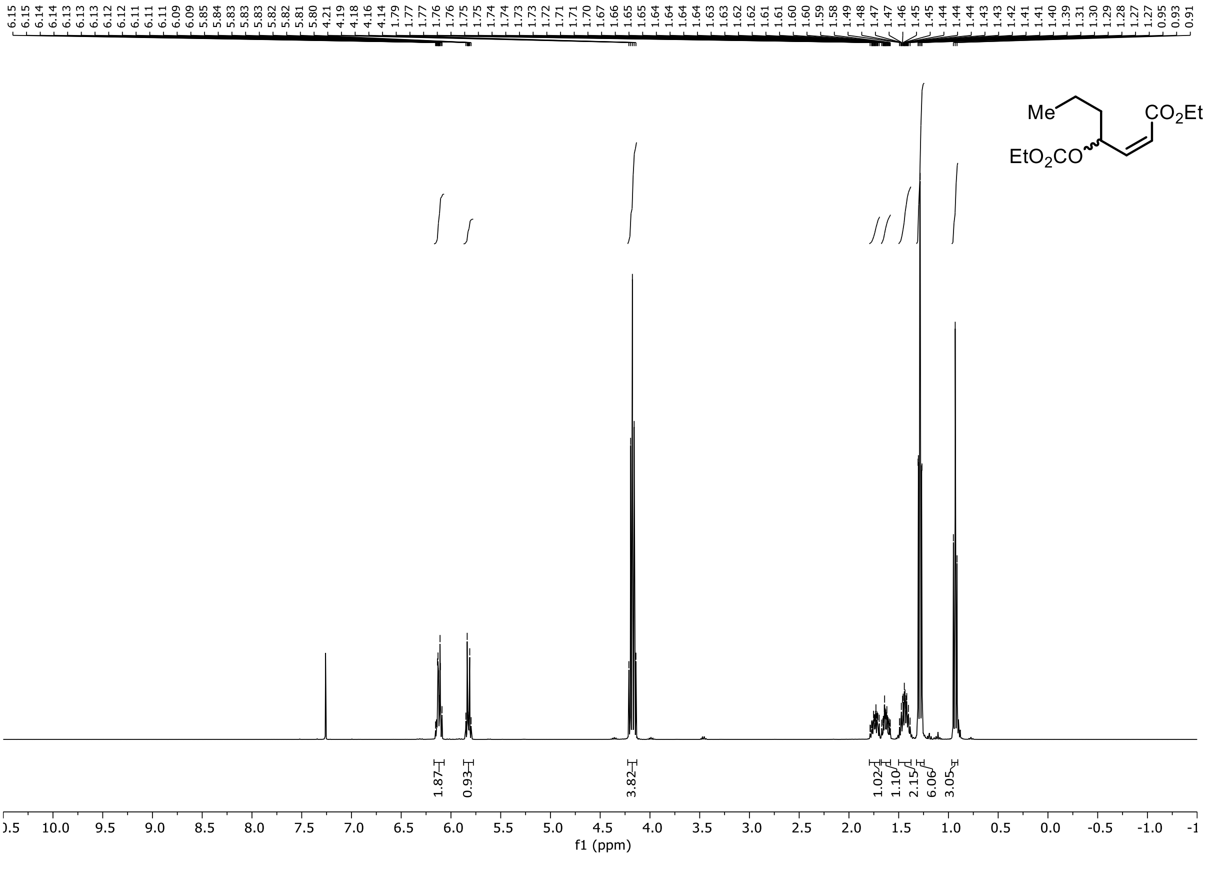

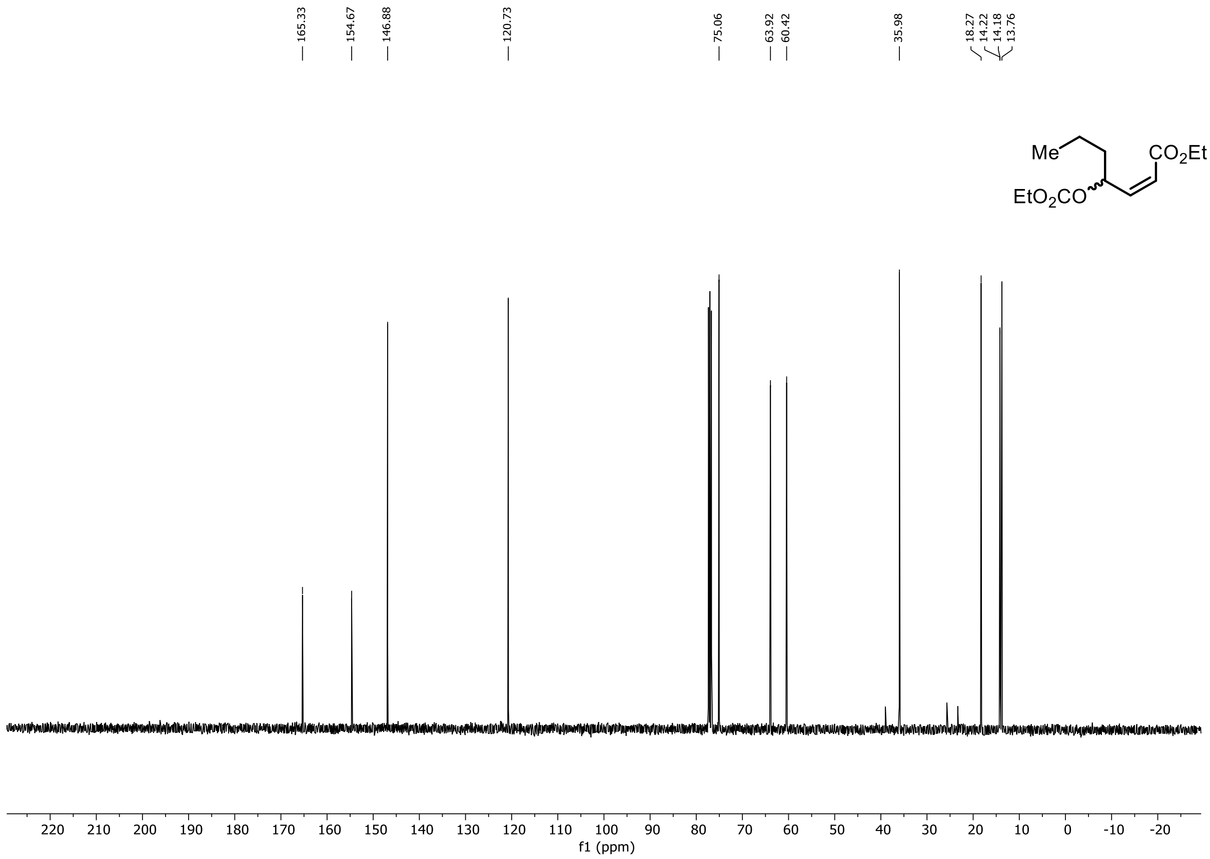

Figure 10.4 (top) ^{1}H NMR (400 MHz) and (bottom) ^{13}C NMR (101 MHz) spectra of (±)-**4a**.

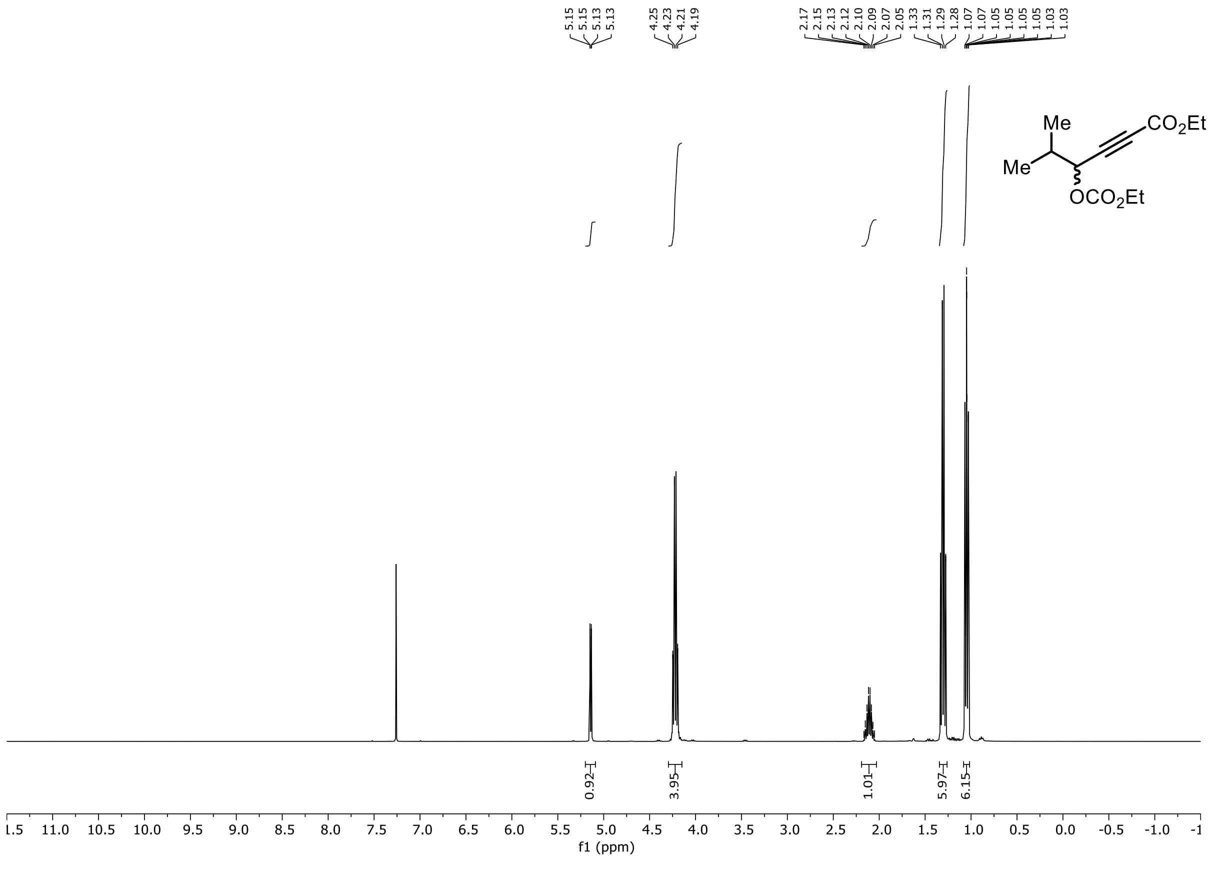

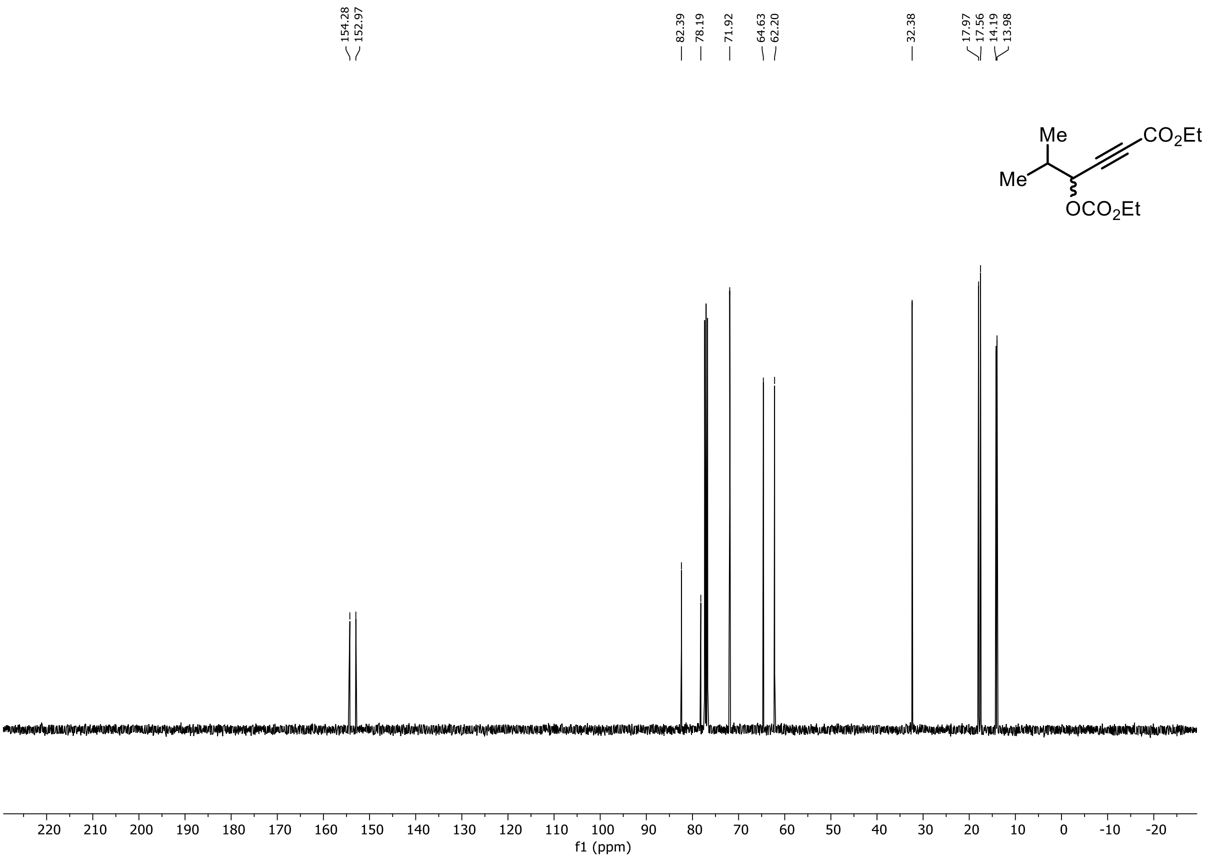

Figure 10.5 (top) ^{1}H NMR (400 MHz) and (bottom) ^{13}C NMR (101 MHz) spectra of **S3**.

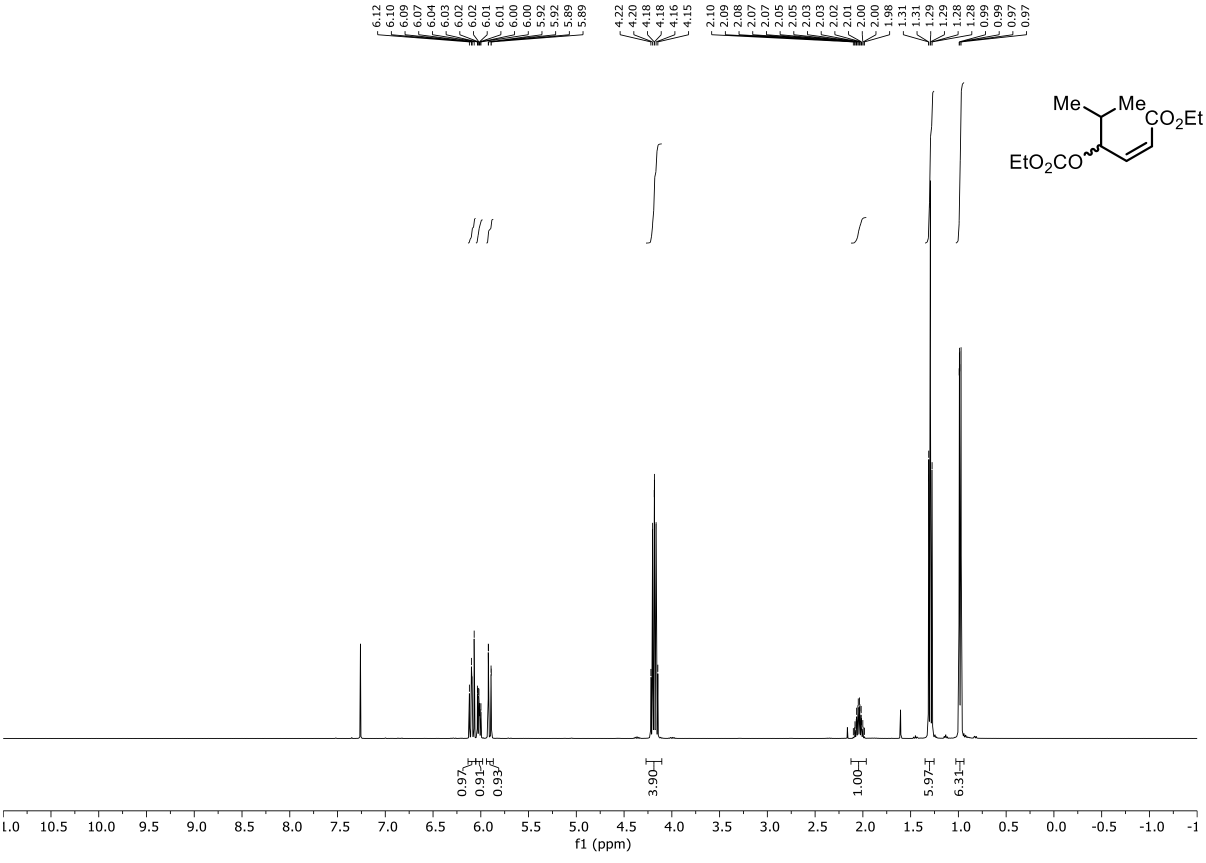

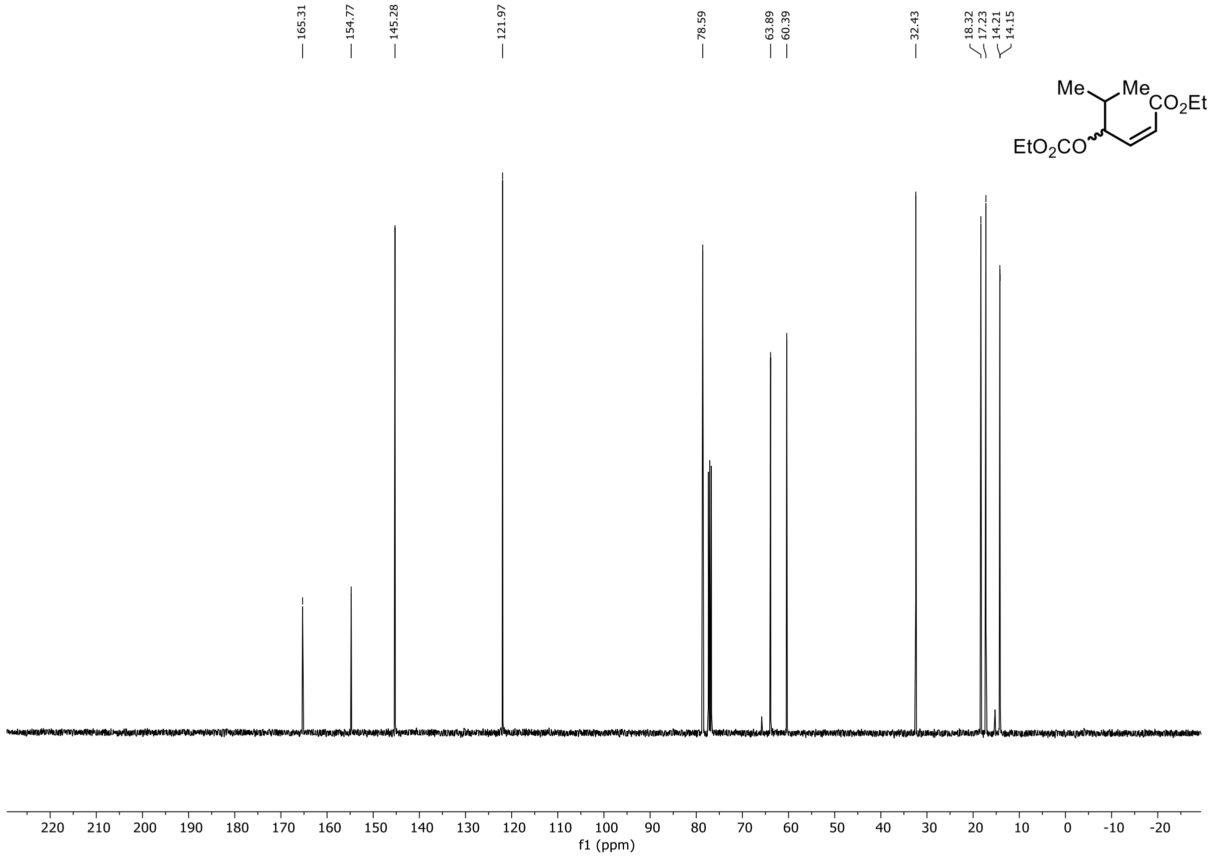

Figure 10.6 (top) ^{1}H NMR (400 MHz) and (bottom) ^{13}C NMR (101 MHz) spectra of (±)-**4b**.

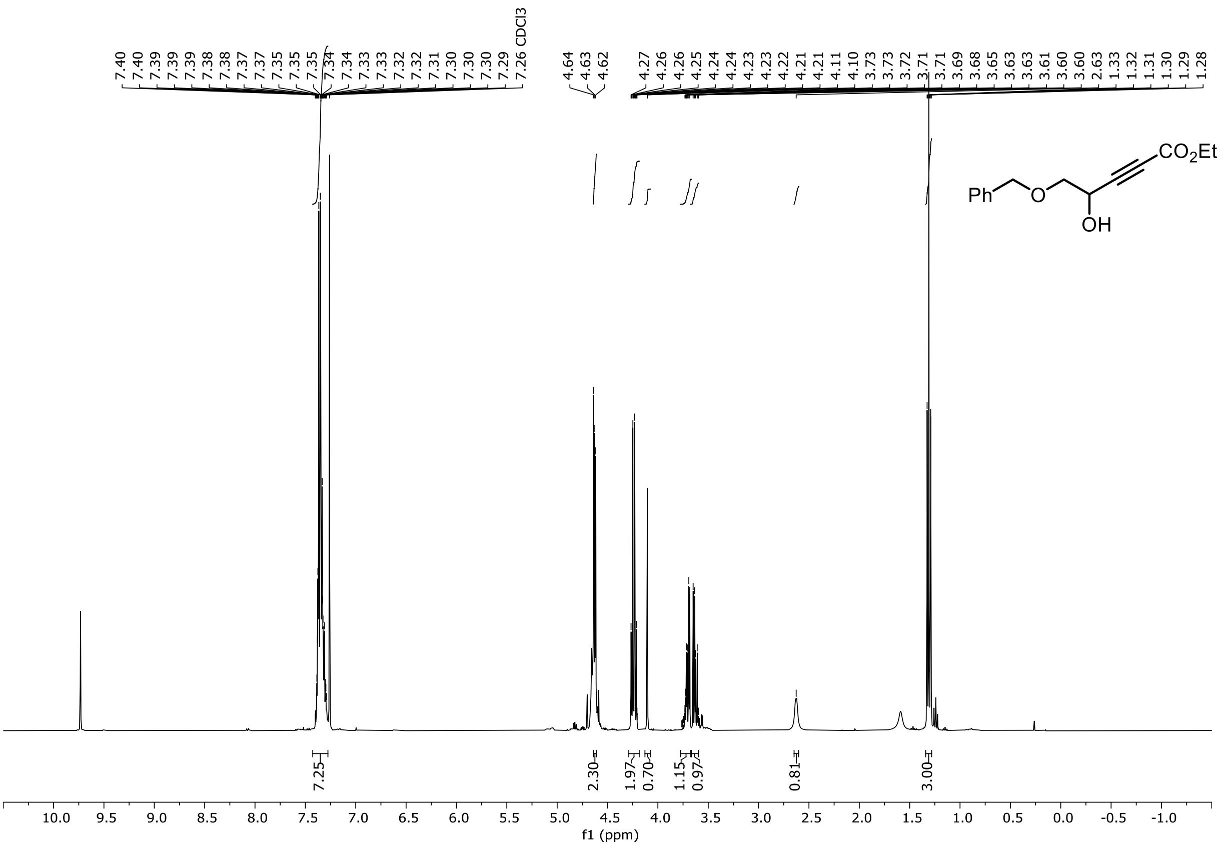

Figure 10.7 ^{1}H NMR (400 MHz) spectrum of **S4** (contains aldehyde starting material impurity).

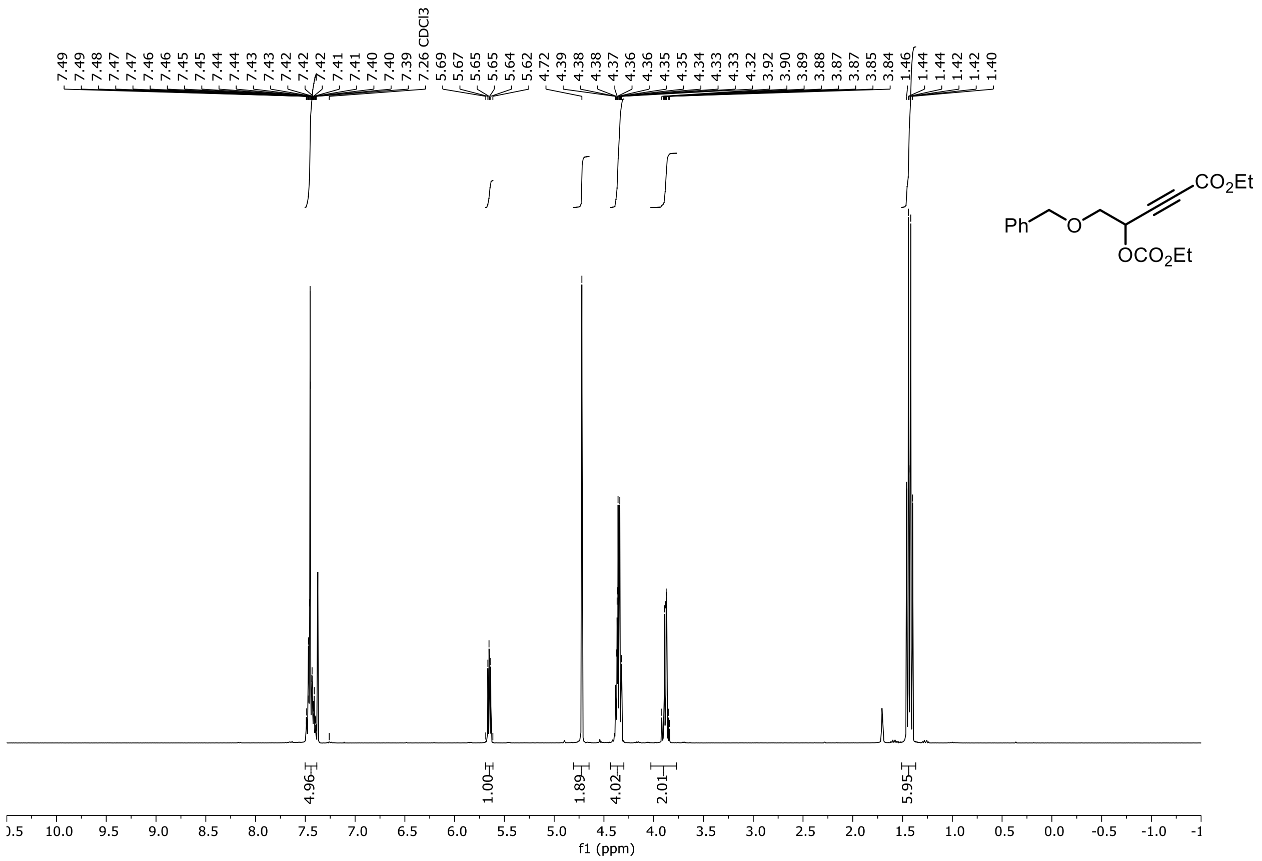

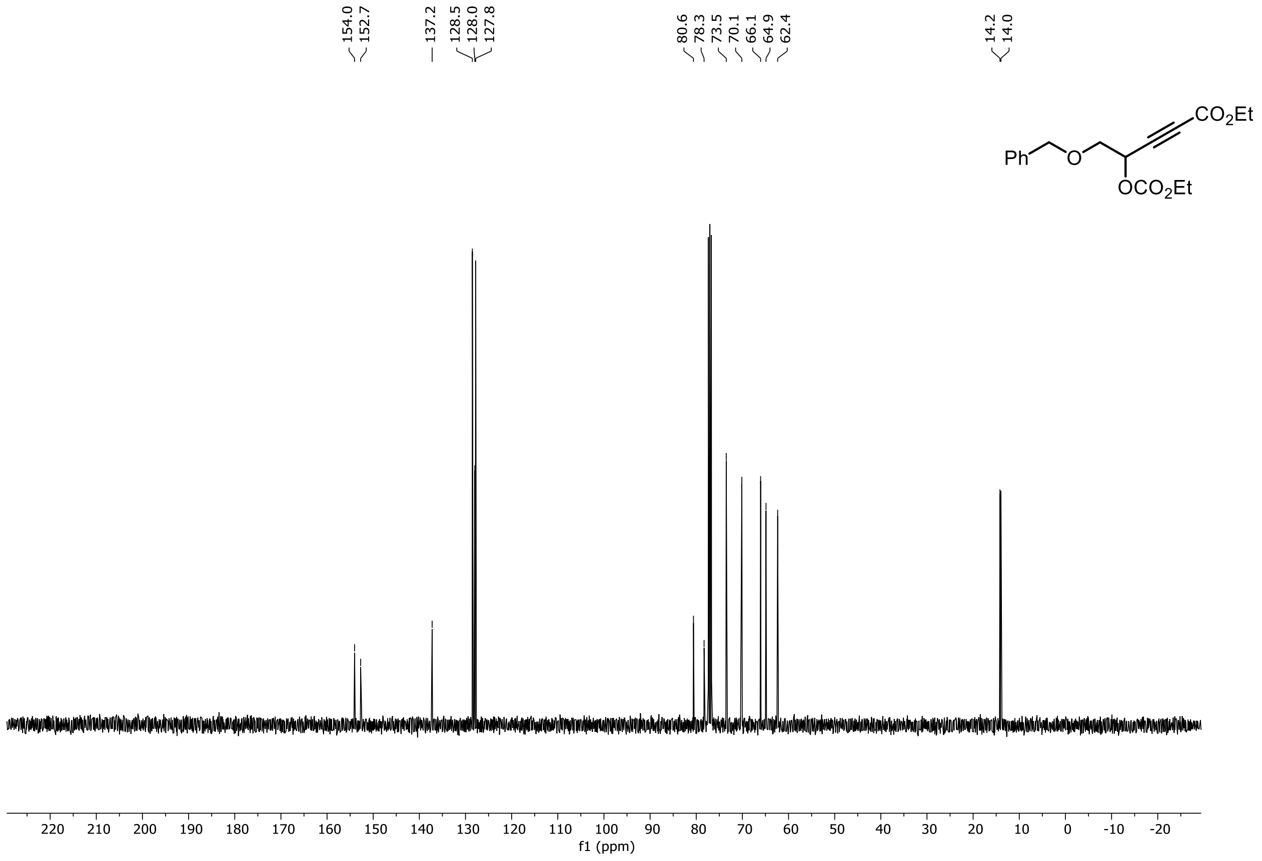

Figure 10.8 (top) ^{1}H NMR (400 MHz) and (bottom) ^{13}C NMR (101 MHz) spectra of **S5**.

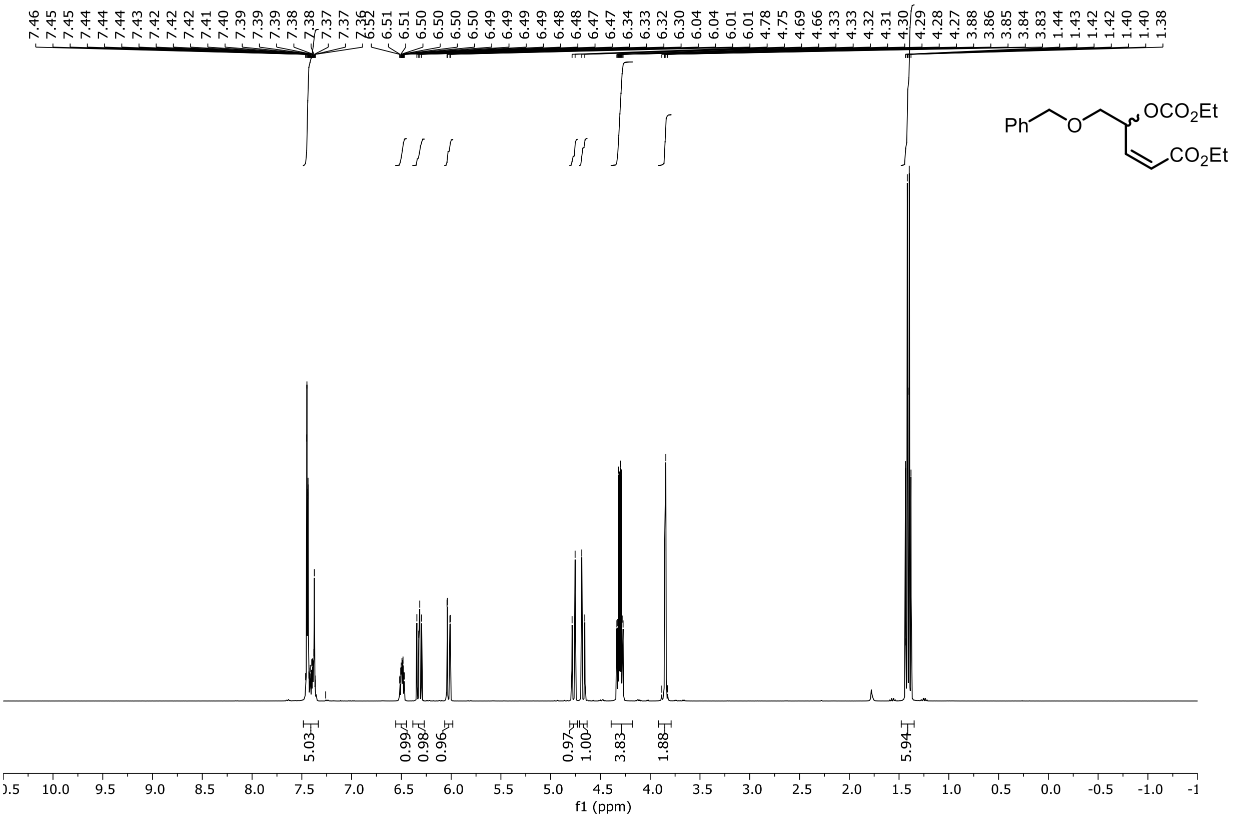

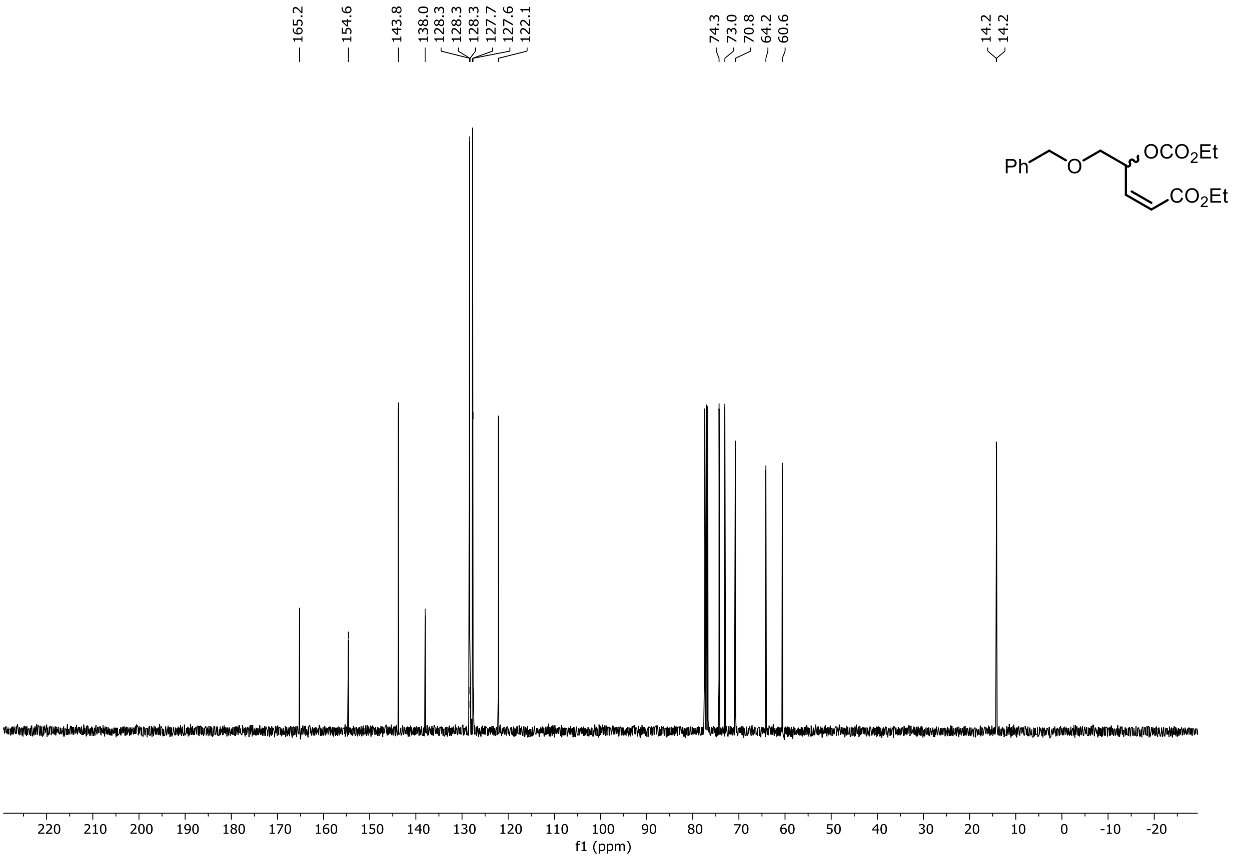

Figure 10.9 (top) ^{1}H NMR (400 MHz) and (bottom) ^{13}C NMR (101 MHz) spectra of (±)-**4c**.

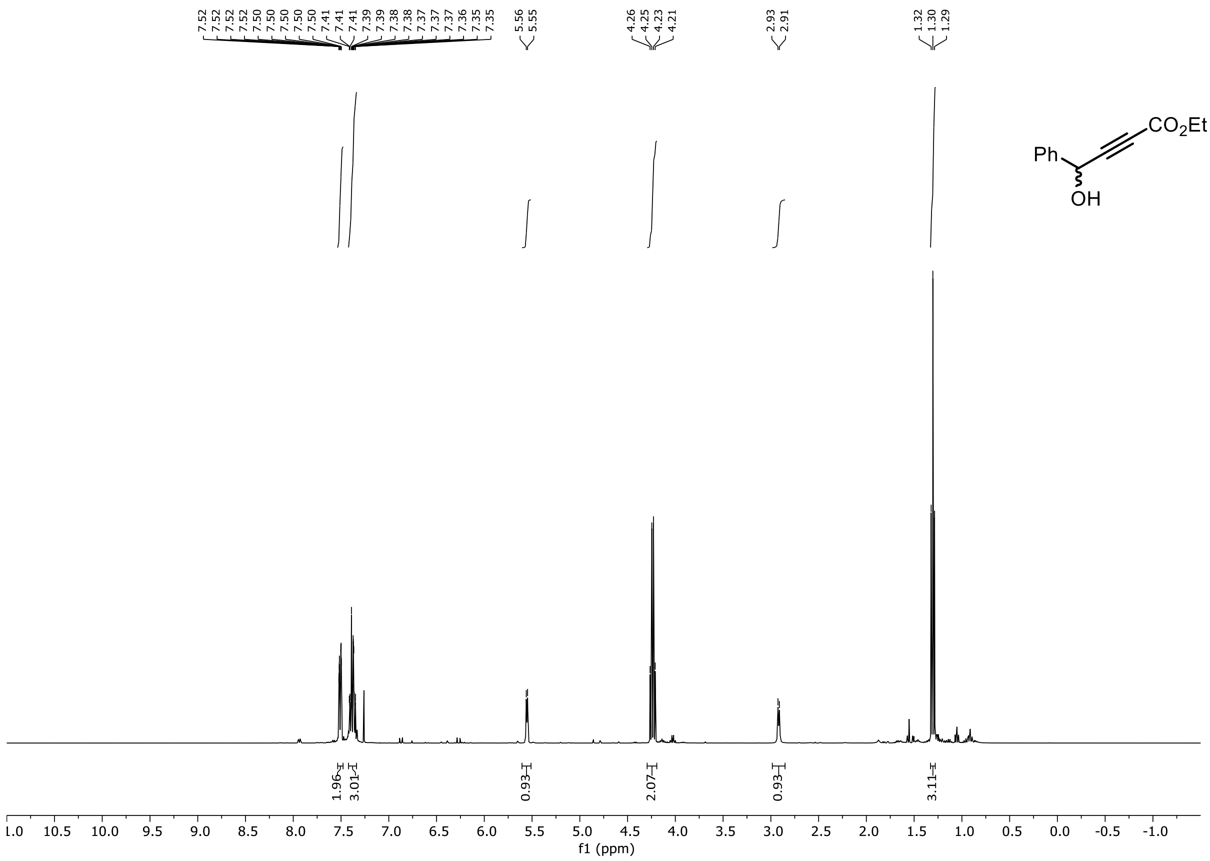

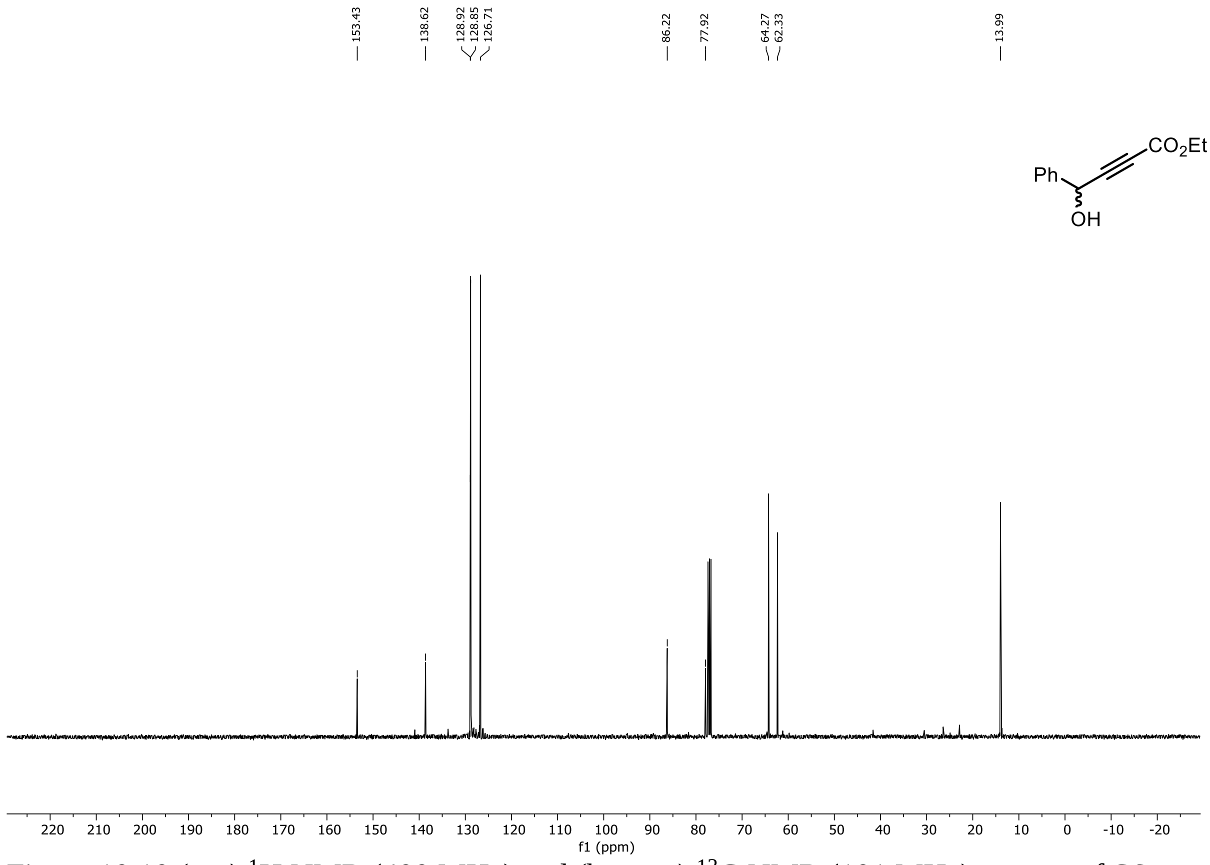

Figure 10.10 (top) ^{1}H NMR (400 MHz) and (bottom) ^{13}C NMR (101 MHz) spectra of **S6**.

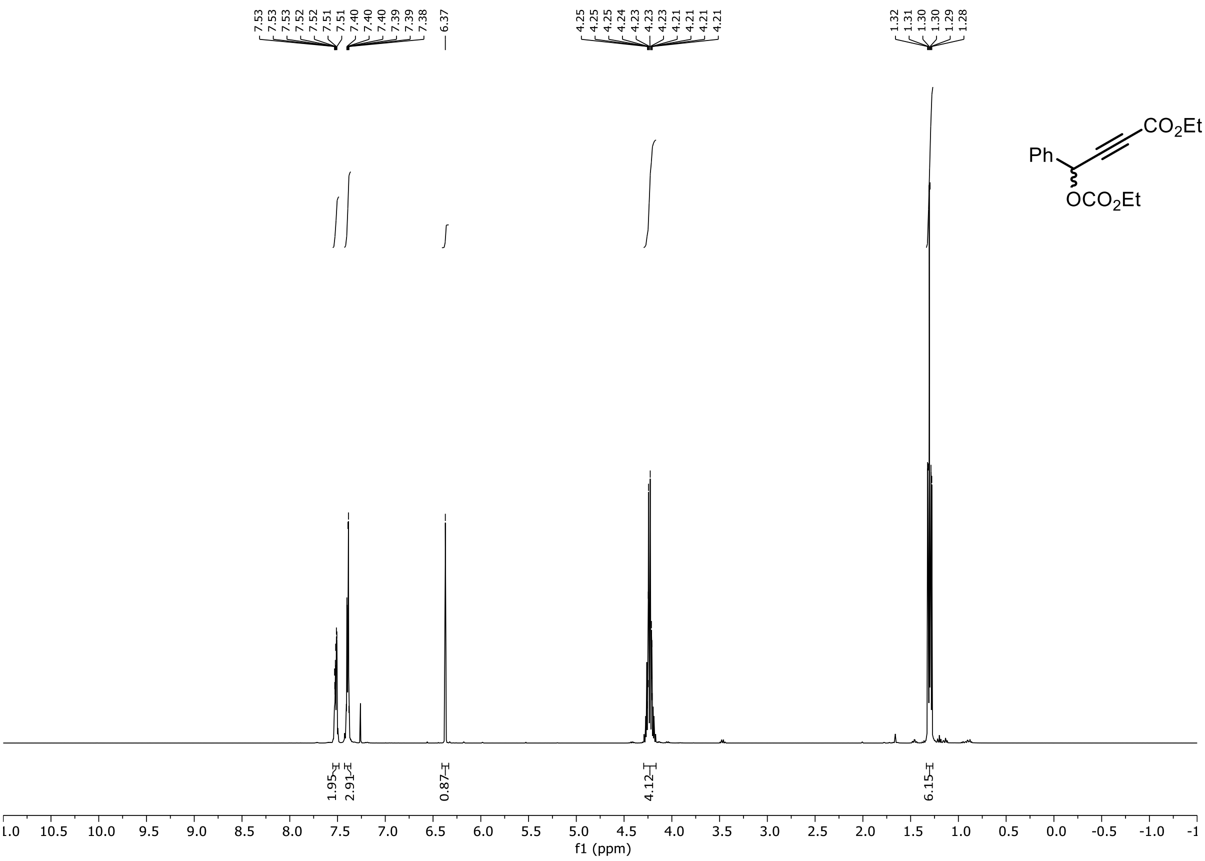

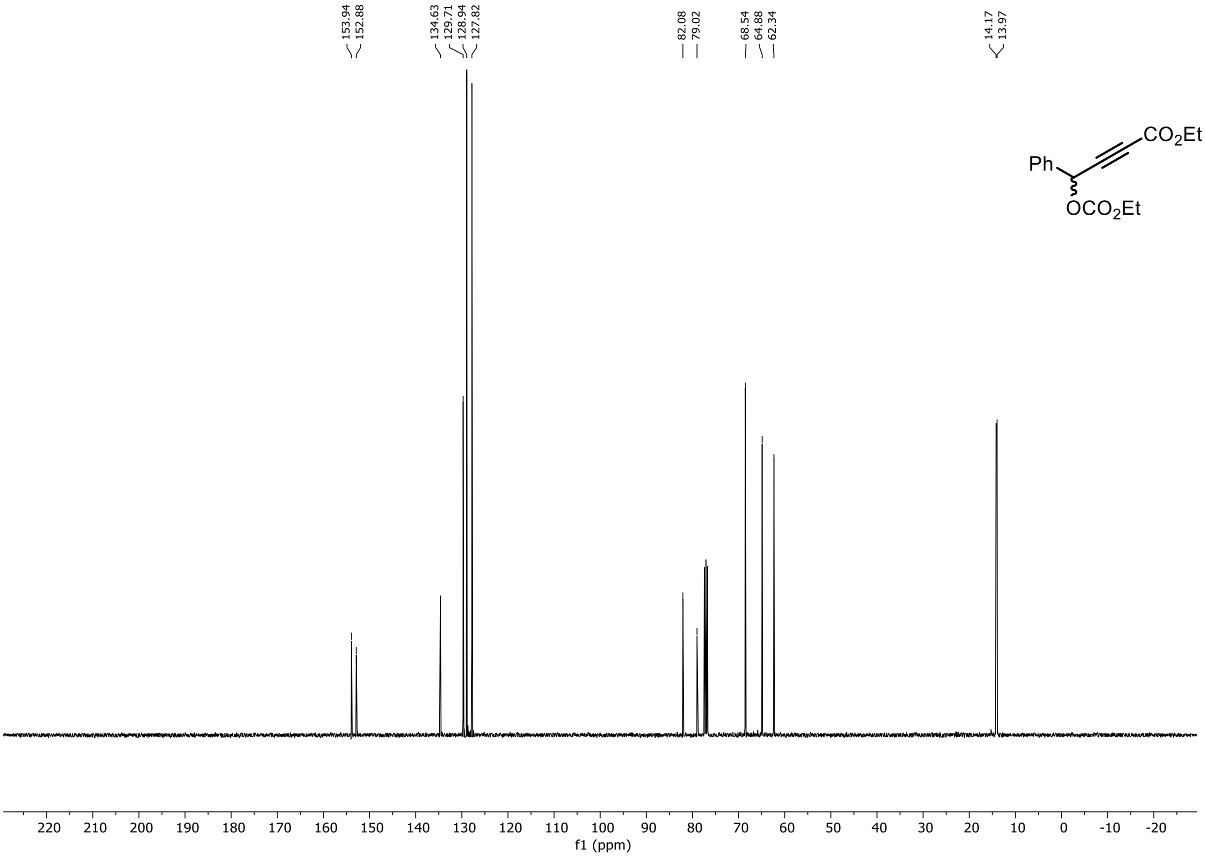

Figure 10.11 (top) ^{1}H NMR (400 MHz) and (bottom) ^{13}C NMR (101 MHz) spectra of **S7**.

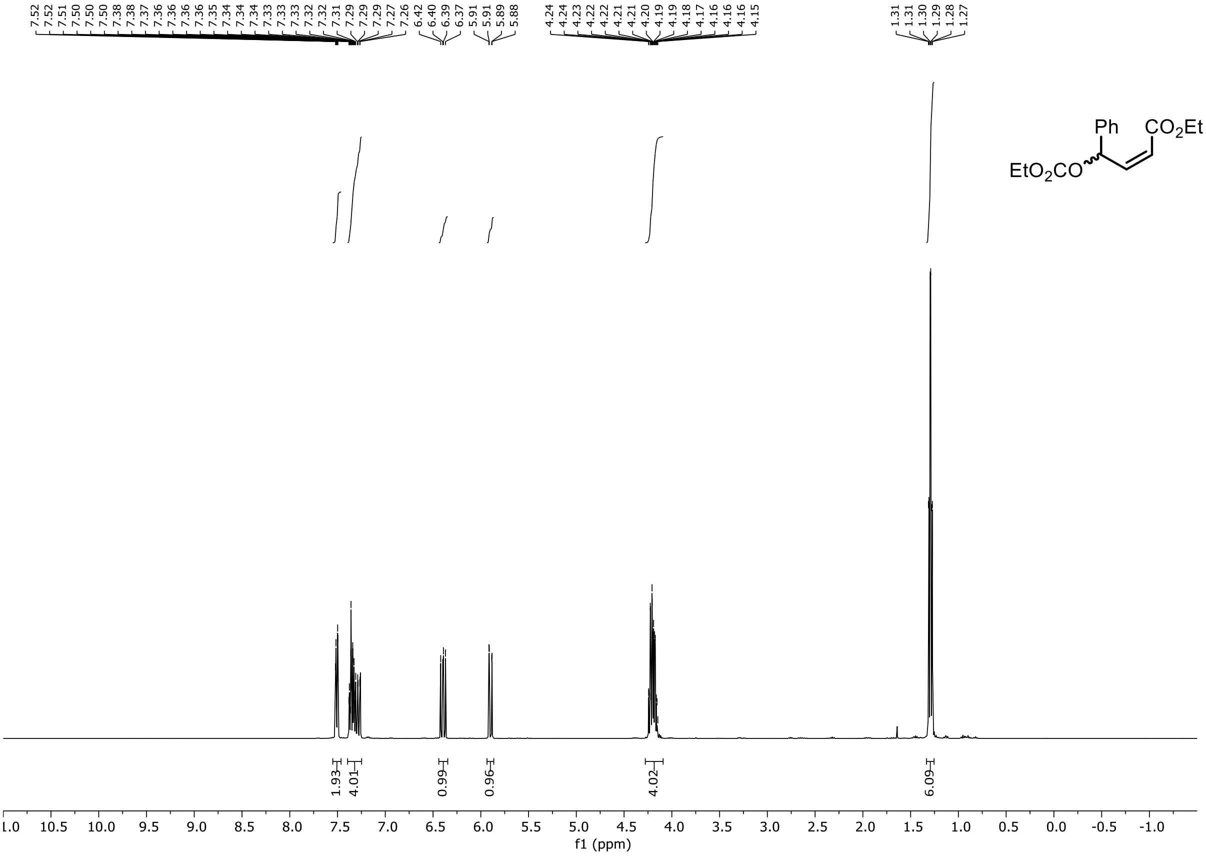

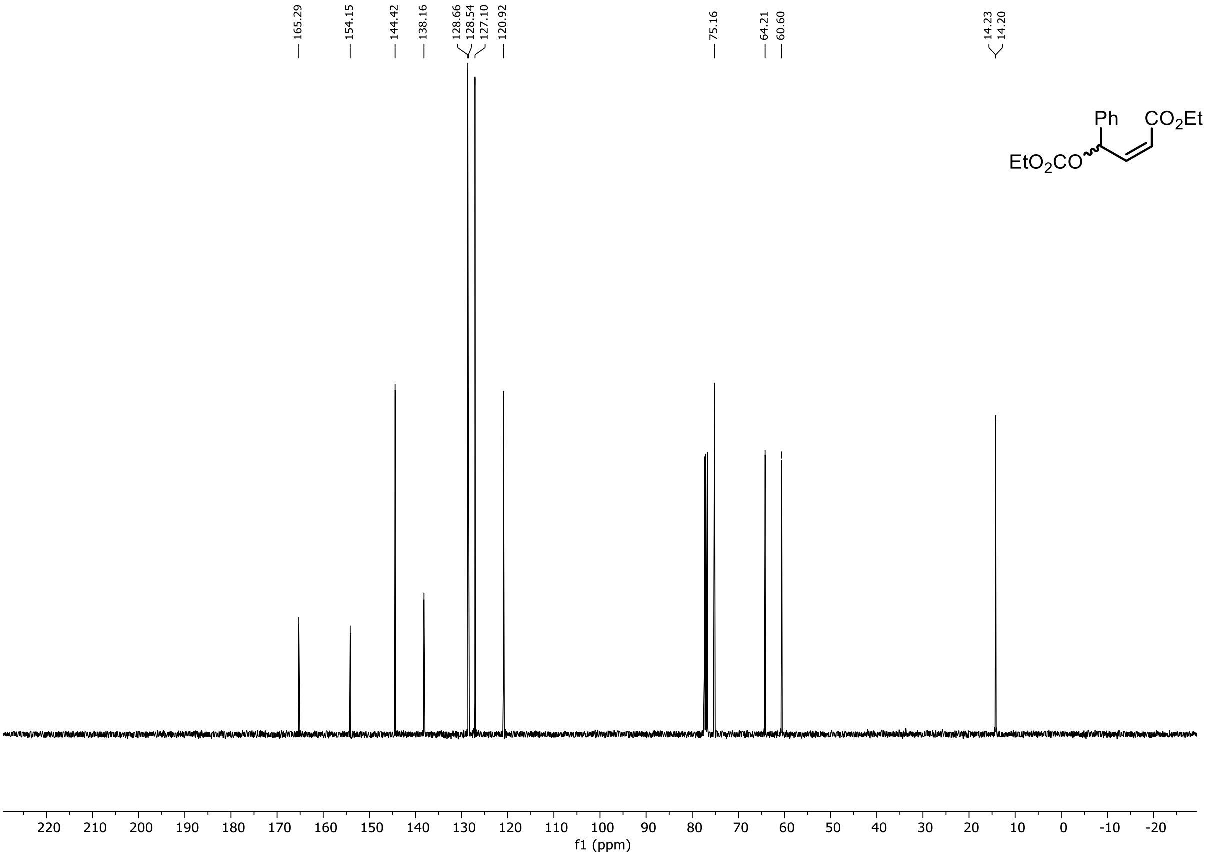

Figure 10.12 (top) ^{1}H NMR (400 MHz) and (bottom) ^{13}C NMR (101 MHz) spectra of (±)-**4d**.

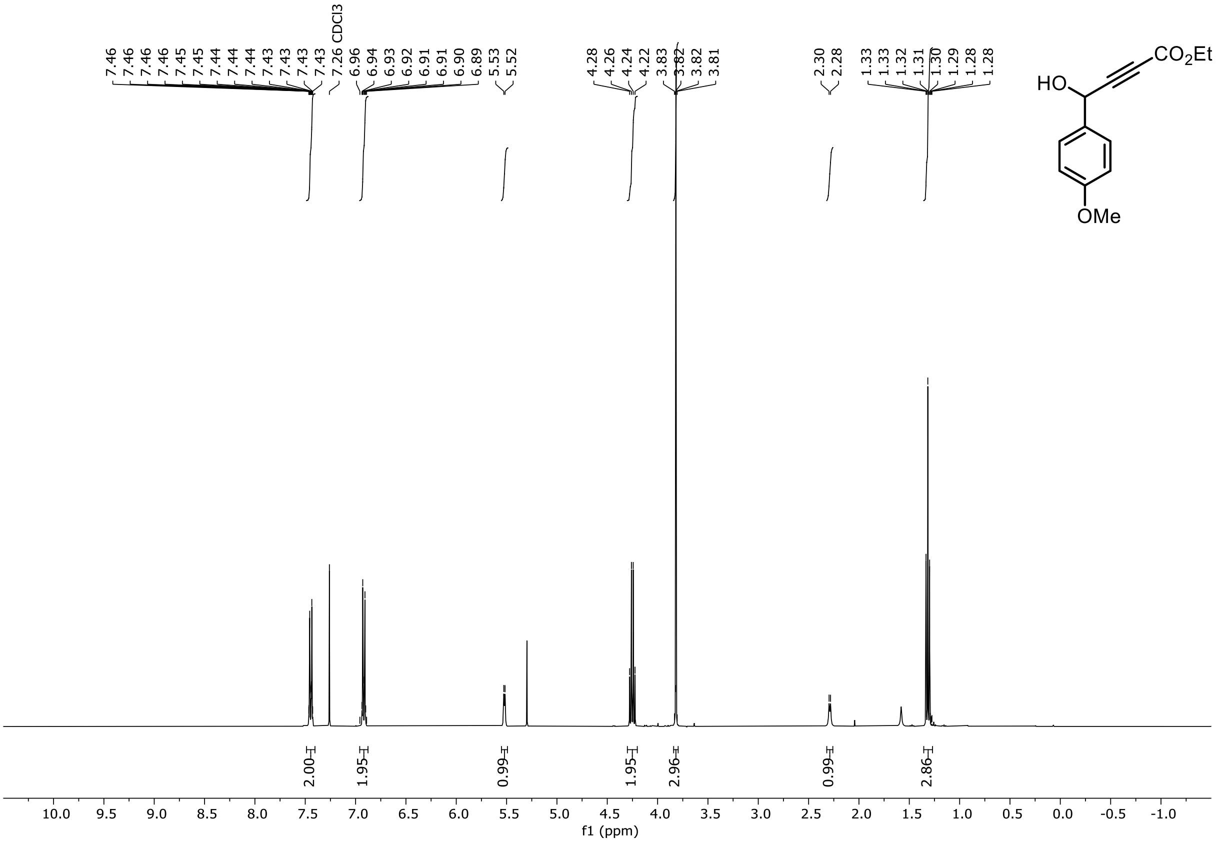

Figure 10.13 ^{1}H NMR (400 MHz) spectrum of **S8.**

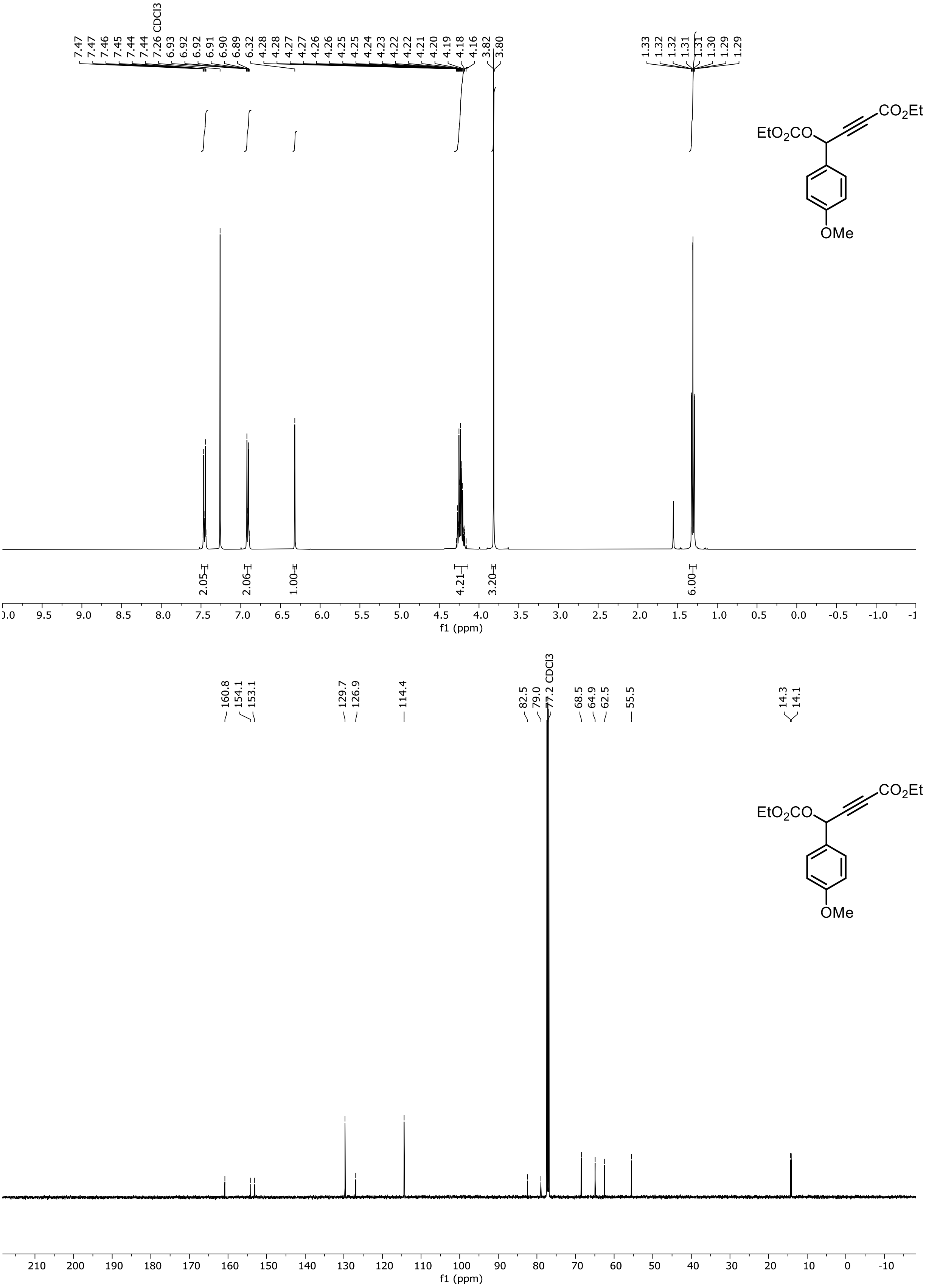

Figure 10.14 (top) ^{1}H NMR (400 MHz) and (bottom) ^{13}C NMR (101 MHz) spectra of **S9**.

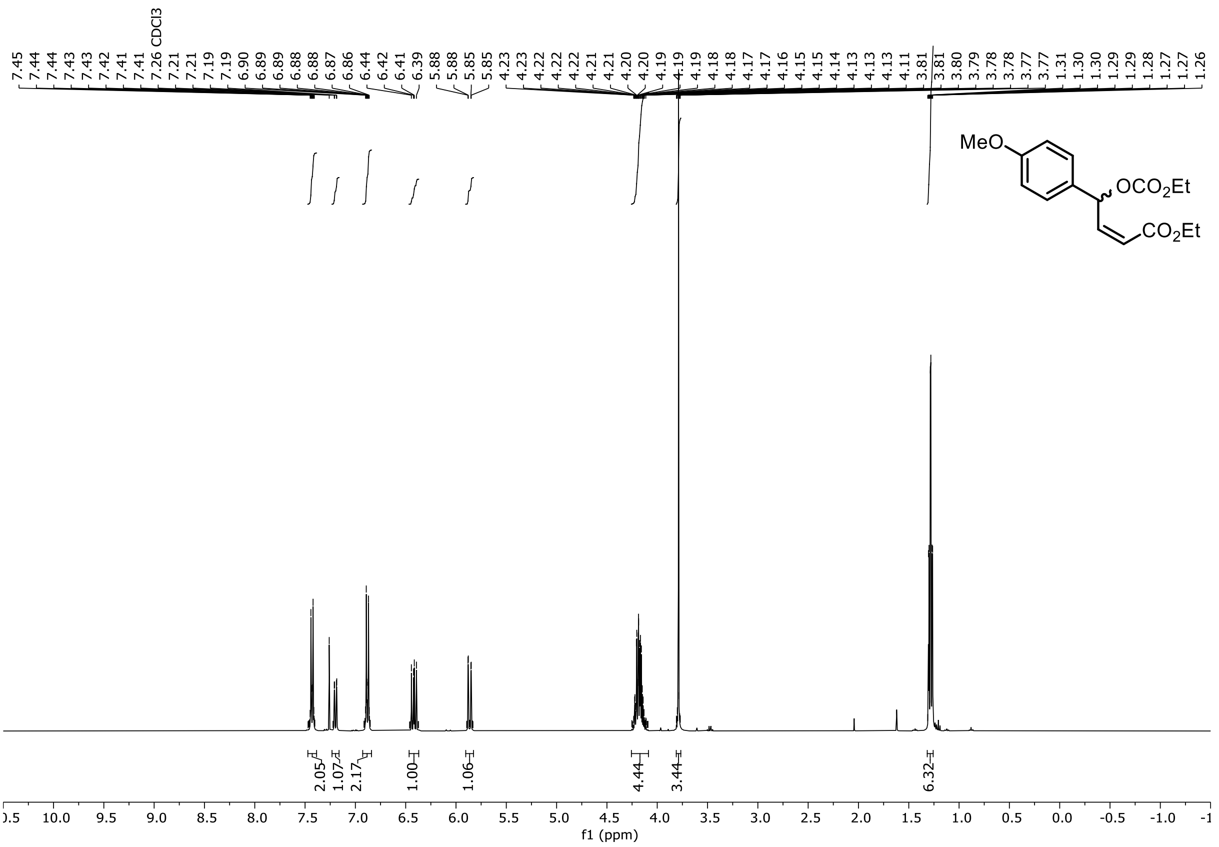

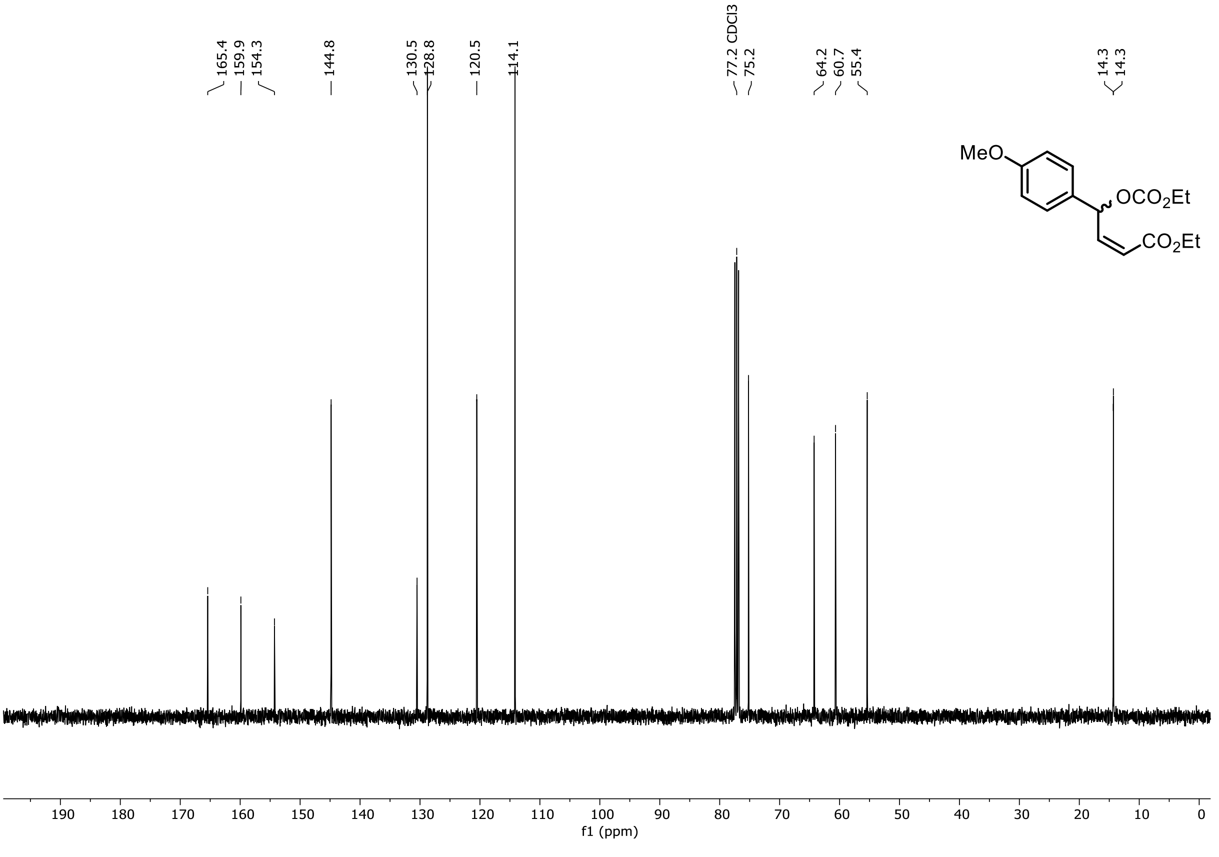

Figure 10.15 (top) ^{1}H NMR (400 MHz) and (bottom) ^{13}C NMR (101 MHz) spectra of (±)-**4e**.

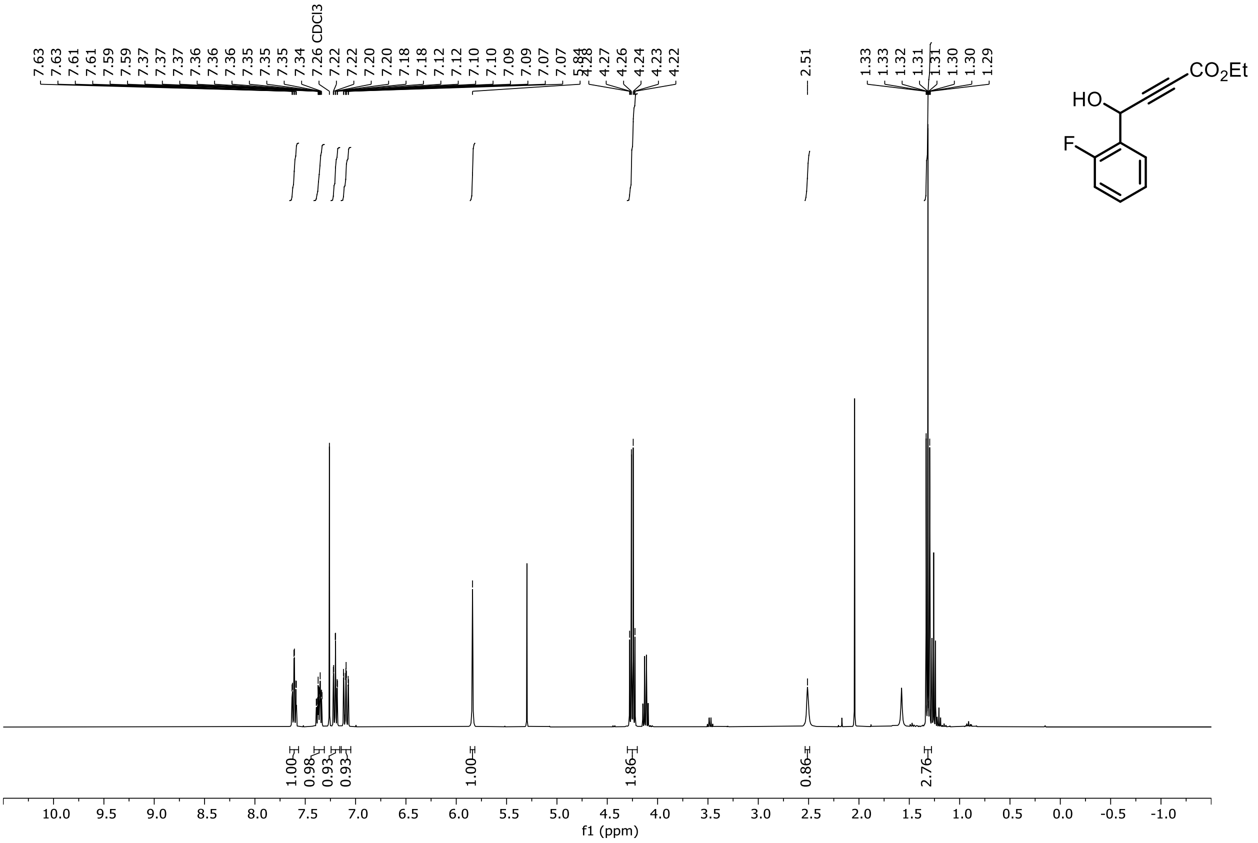

Figure 10.16 ^{1}H NMR (400 MHz) spectrum of **S10**

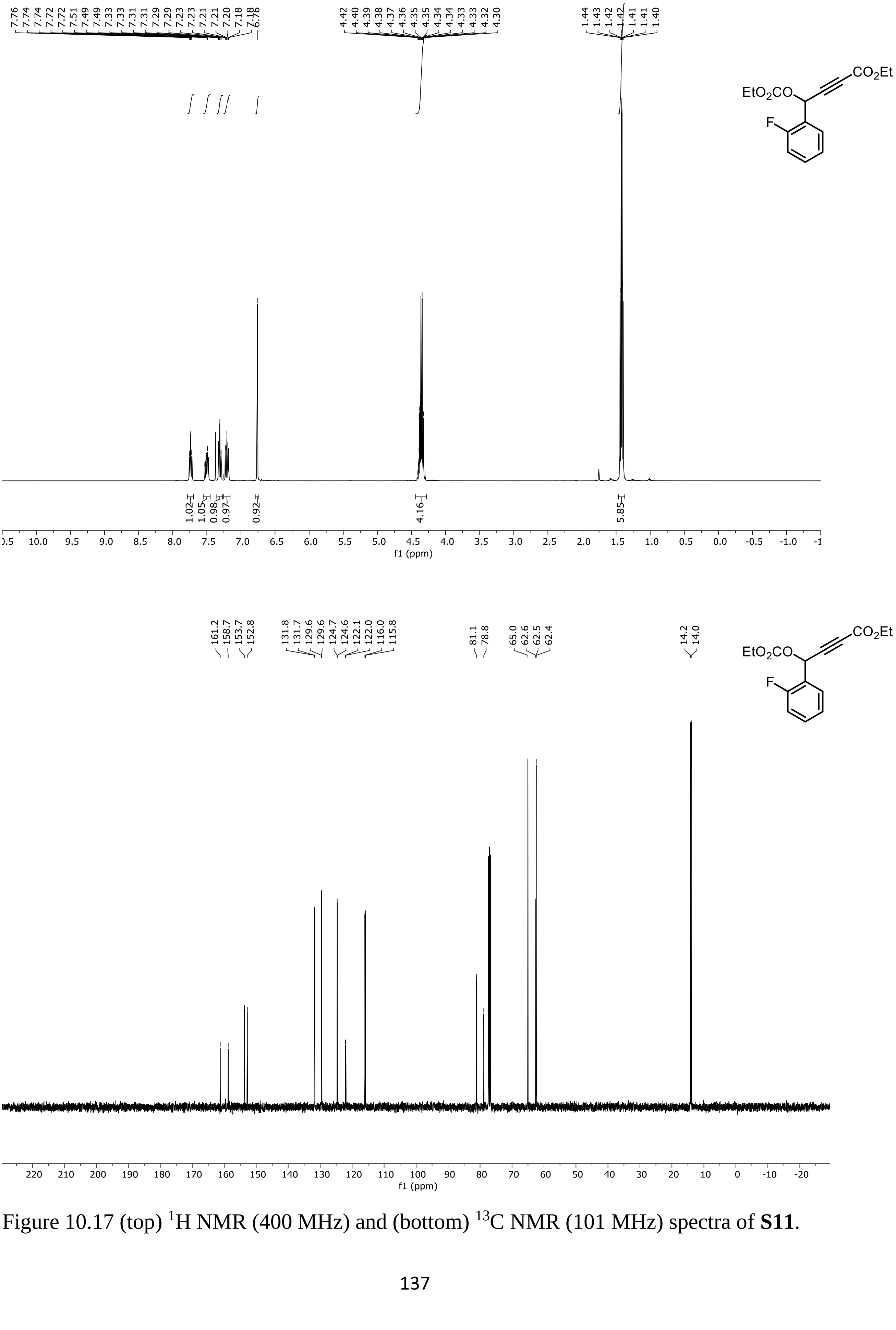

Figure 10.17 (top) ^{1}H NMR (400 MHz) and (bottom) ^{13}C NMR (101 MHz) spectra of **S11**.

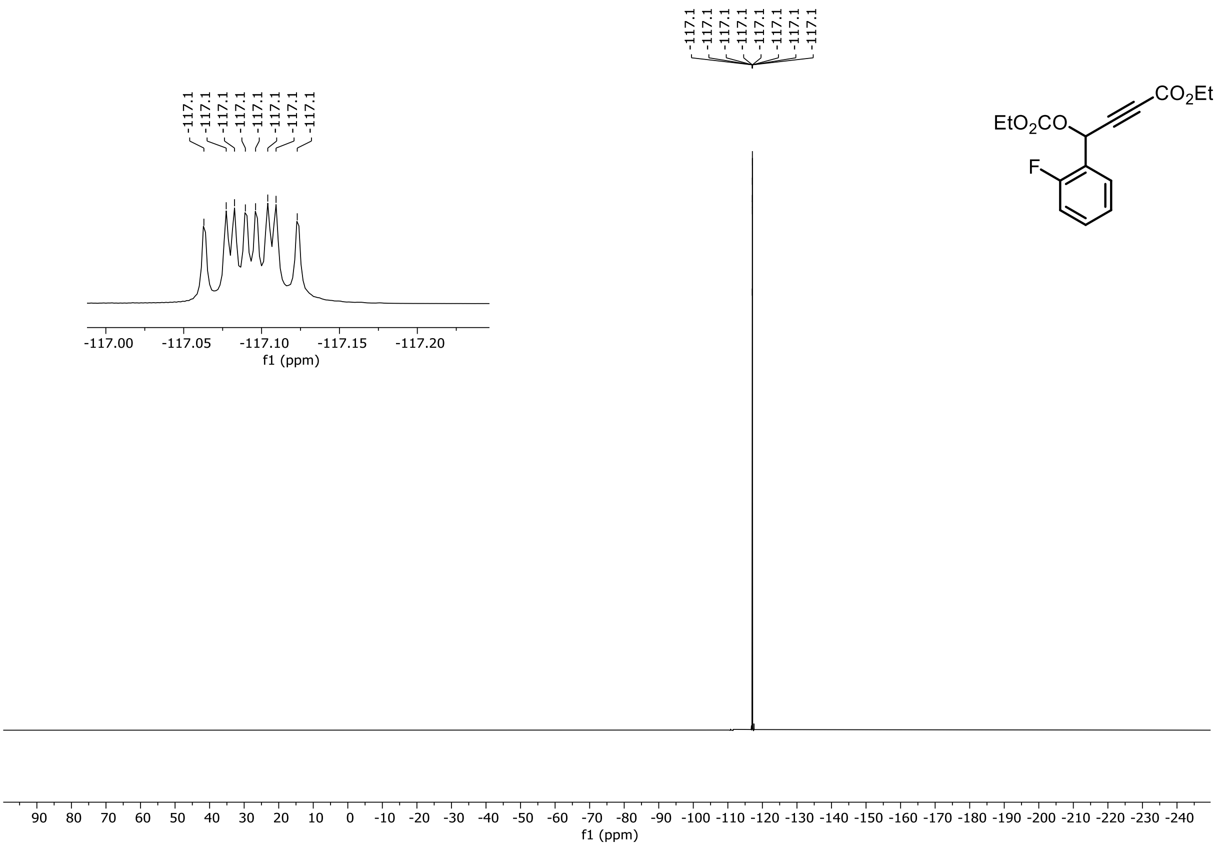

Figure 10.18 ^{19}F NMR (376 MHz) spectrum of **S11**.

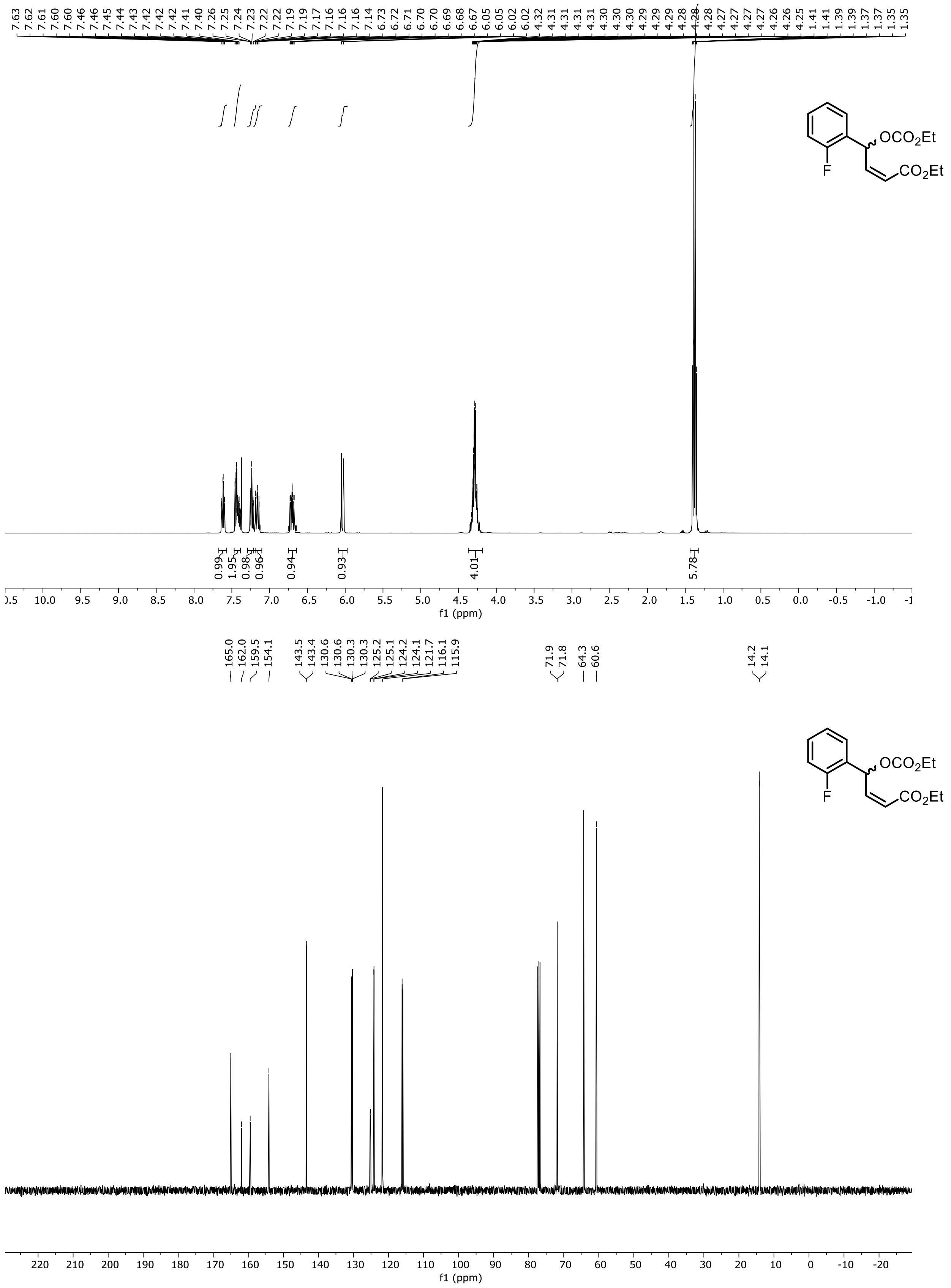

Figure 10.19 (top) ^{1}H NMR (400 MHz) and (bottom) ^{13}C NMR (101 MHz) spectra of (±)-**4f**.

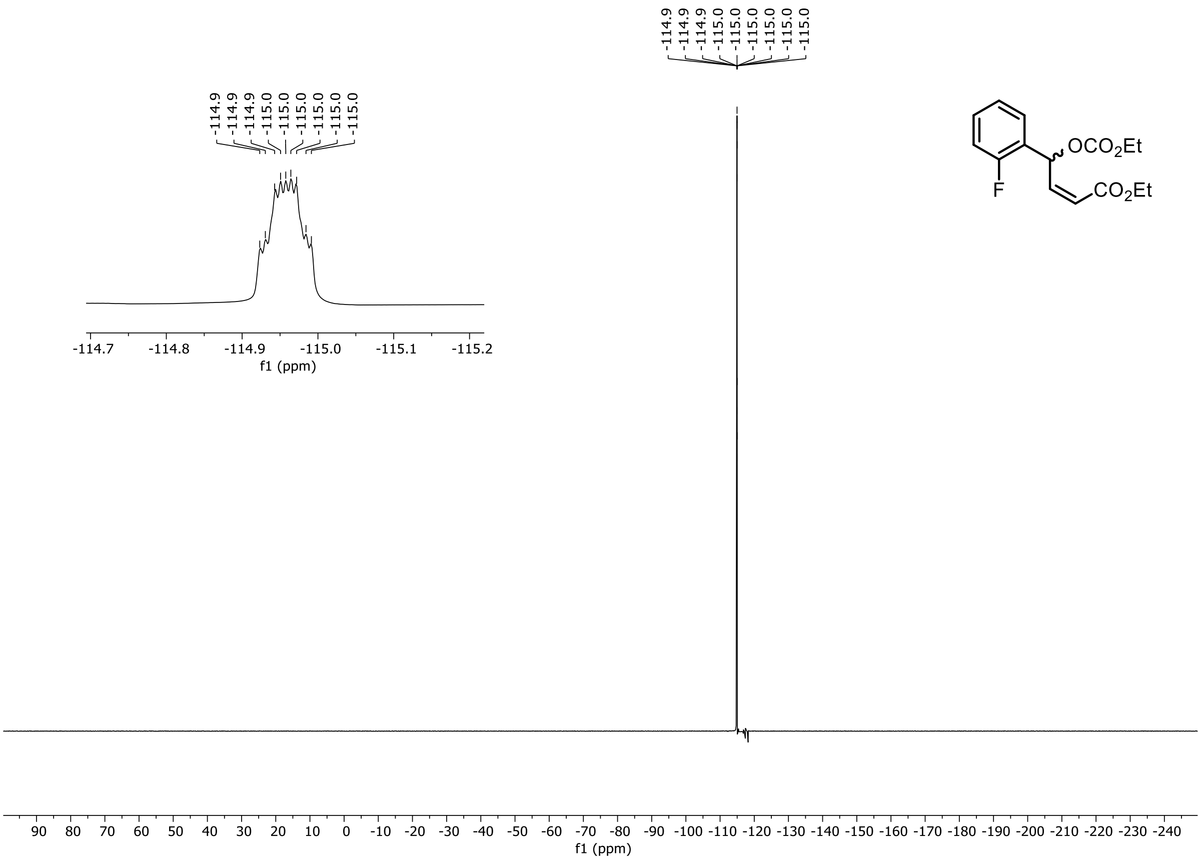

Figure 10.20 ^{19}F NMR (376 MHz) spectrum of (±)-**4f**.

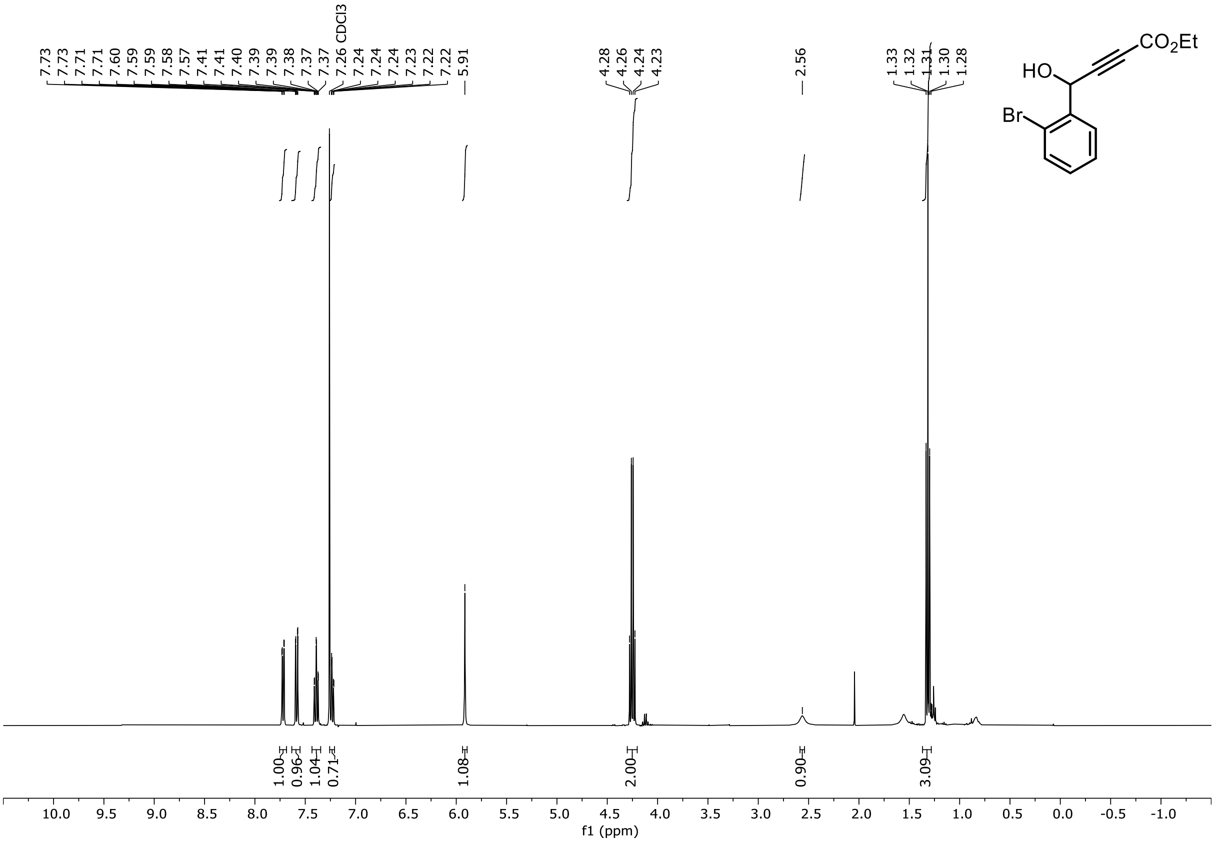

Figure 10.21 ^{1}H NMR (400 MHz) spectrum of **S12**.

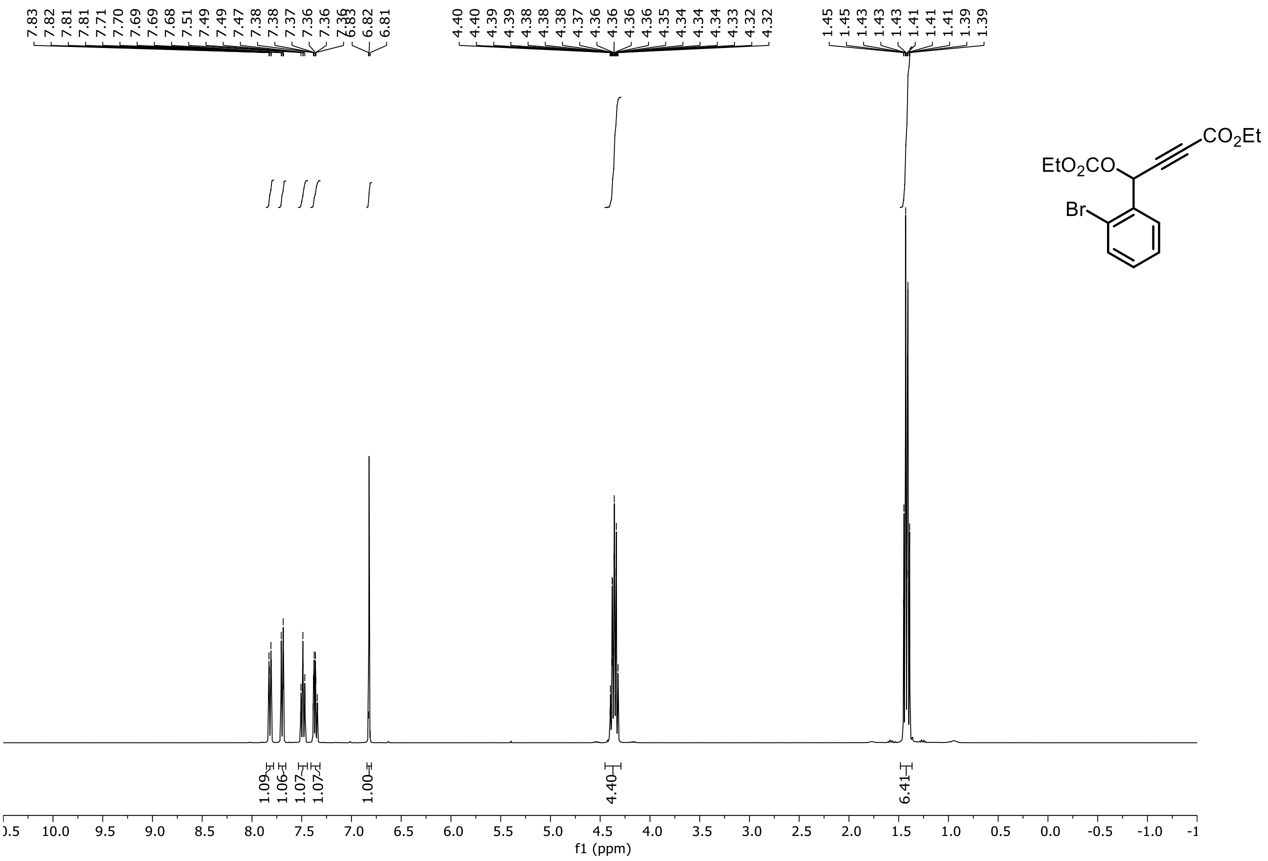

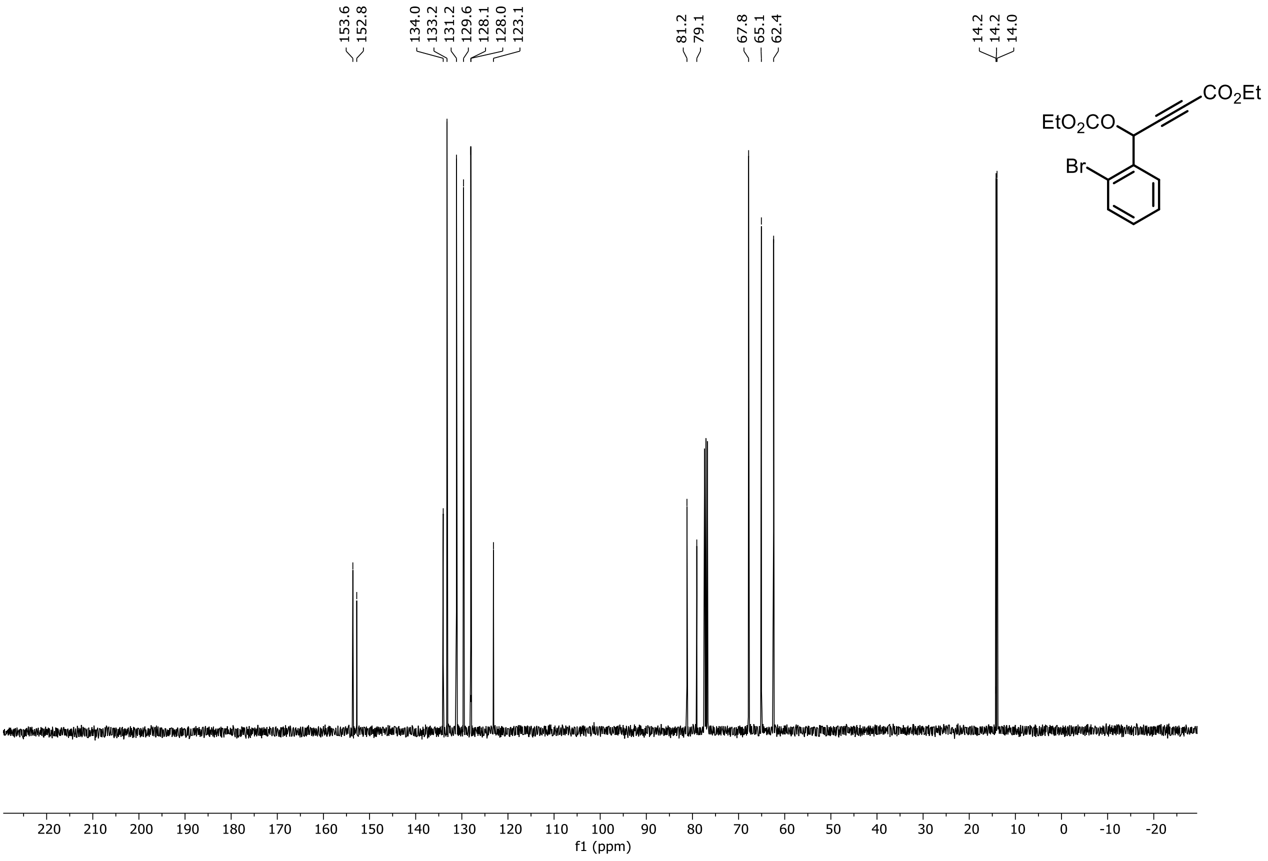

Figure 10.22 (top) ^{1}H NMR (400 MHz) and (bottom) ^{13}C NMR (101 MHz) spectra of **S13**.

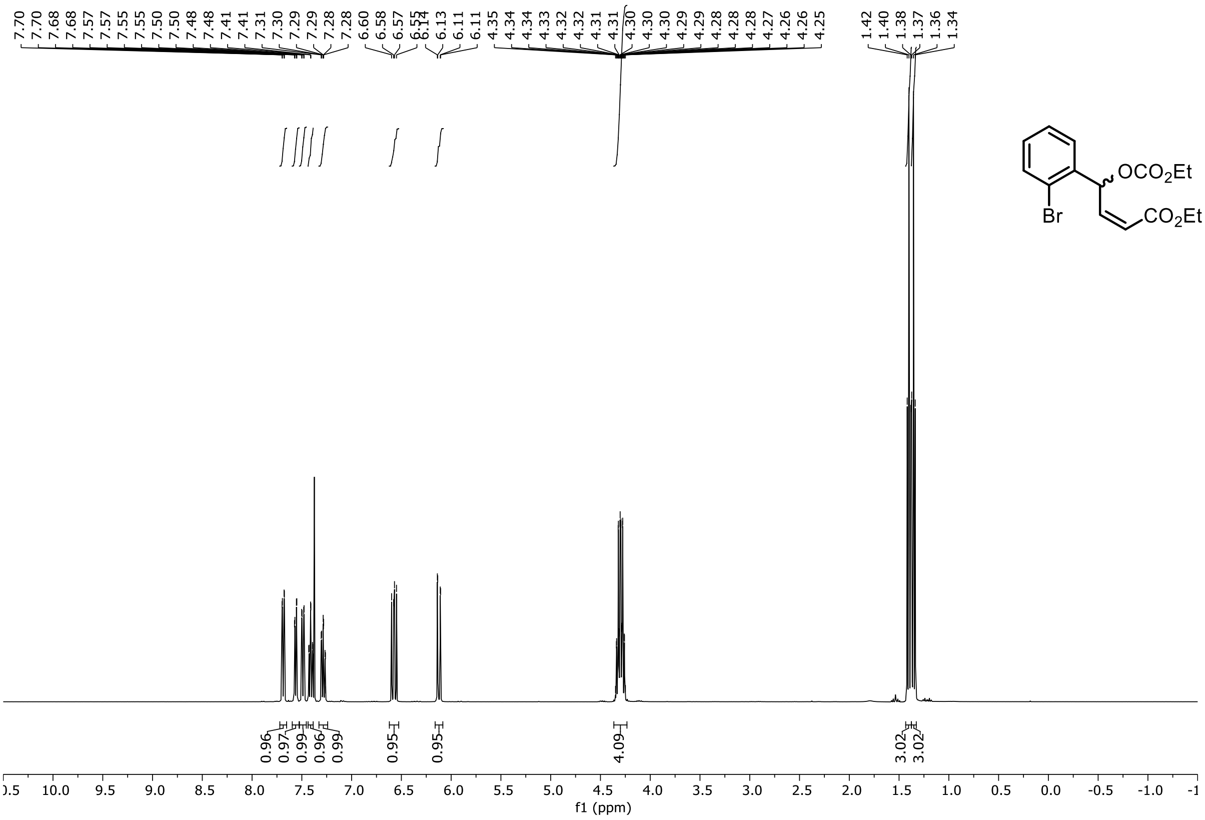

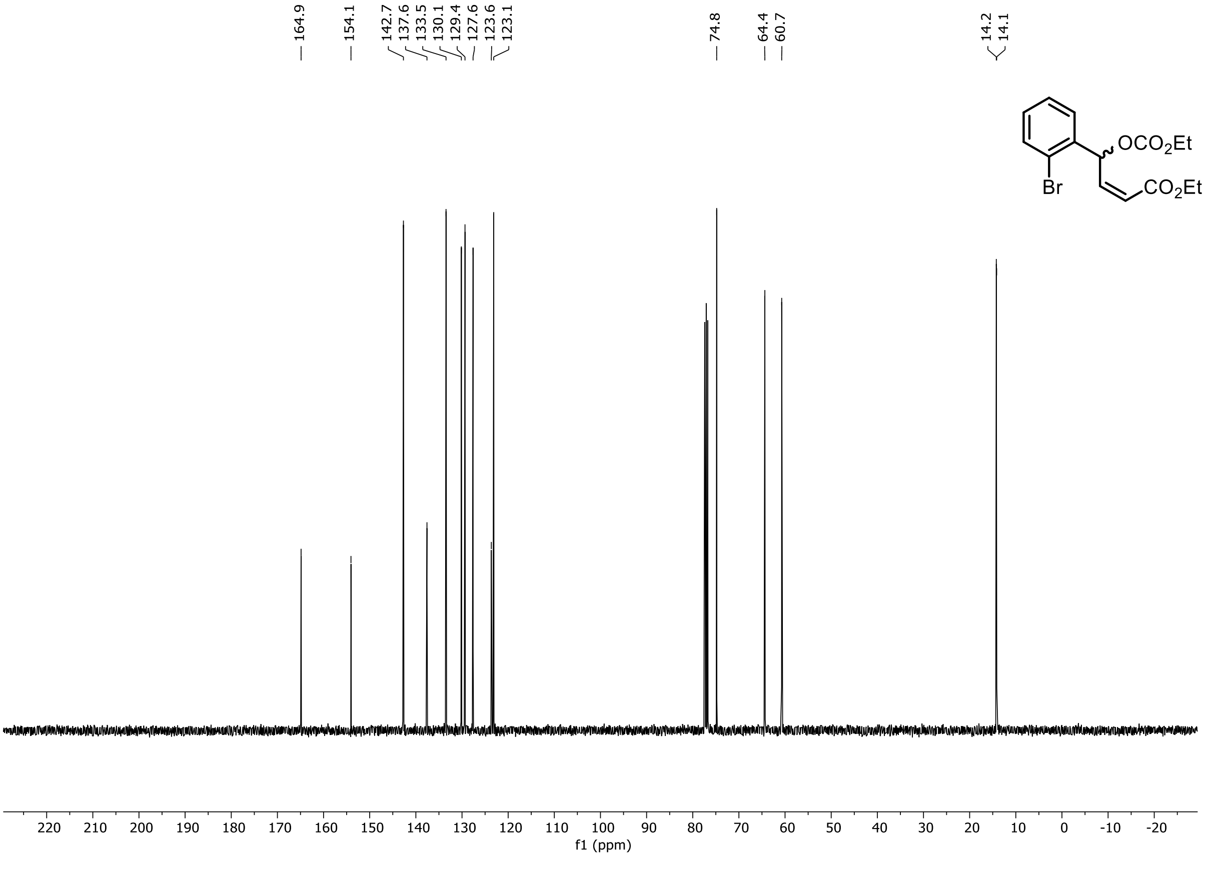

Figure 10.23 (top) ^{1}H NMR (400 MHz) and (bottom) ^{13}C NMR (101 MHz) spectra of (±)-**4**

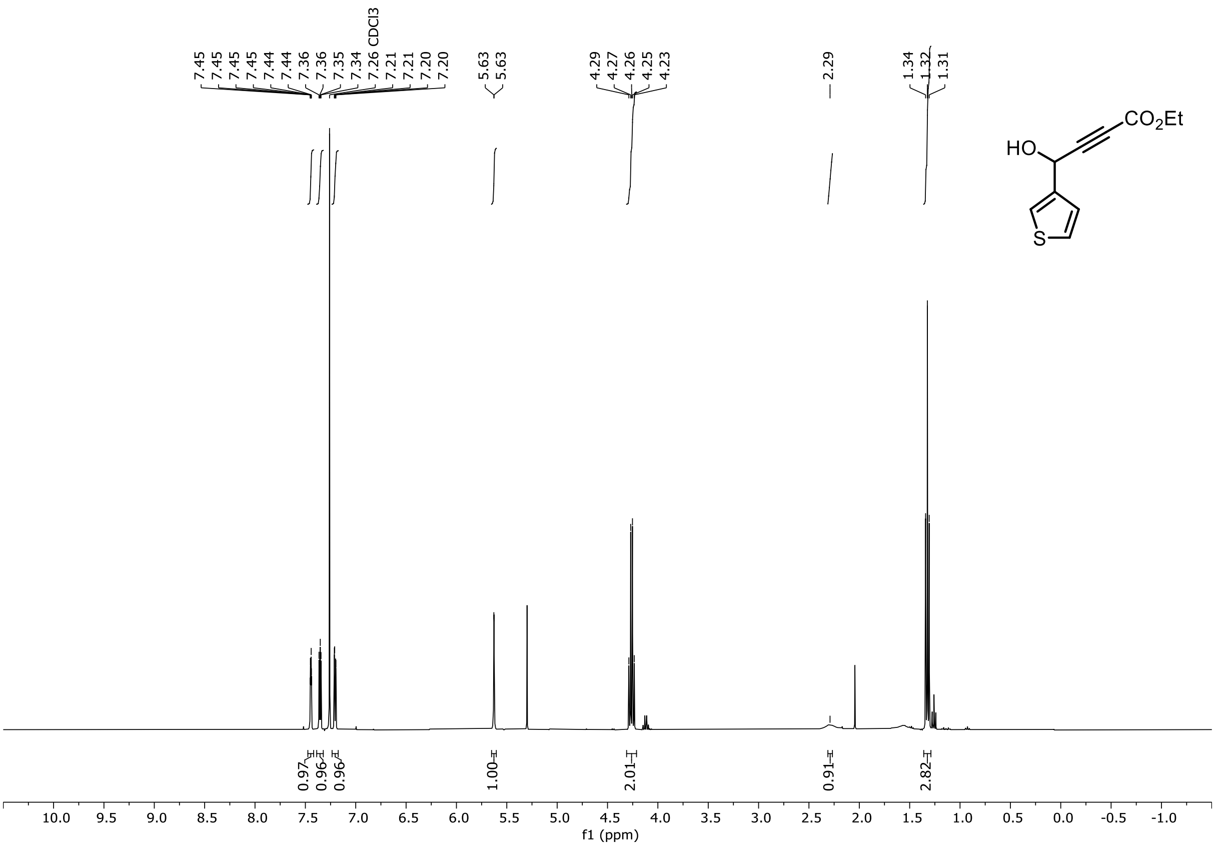

Figure 10.24 ^{1}H NMR (400 MHz) spectrum of **S14**.

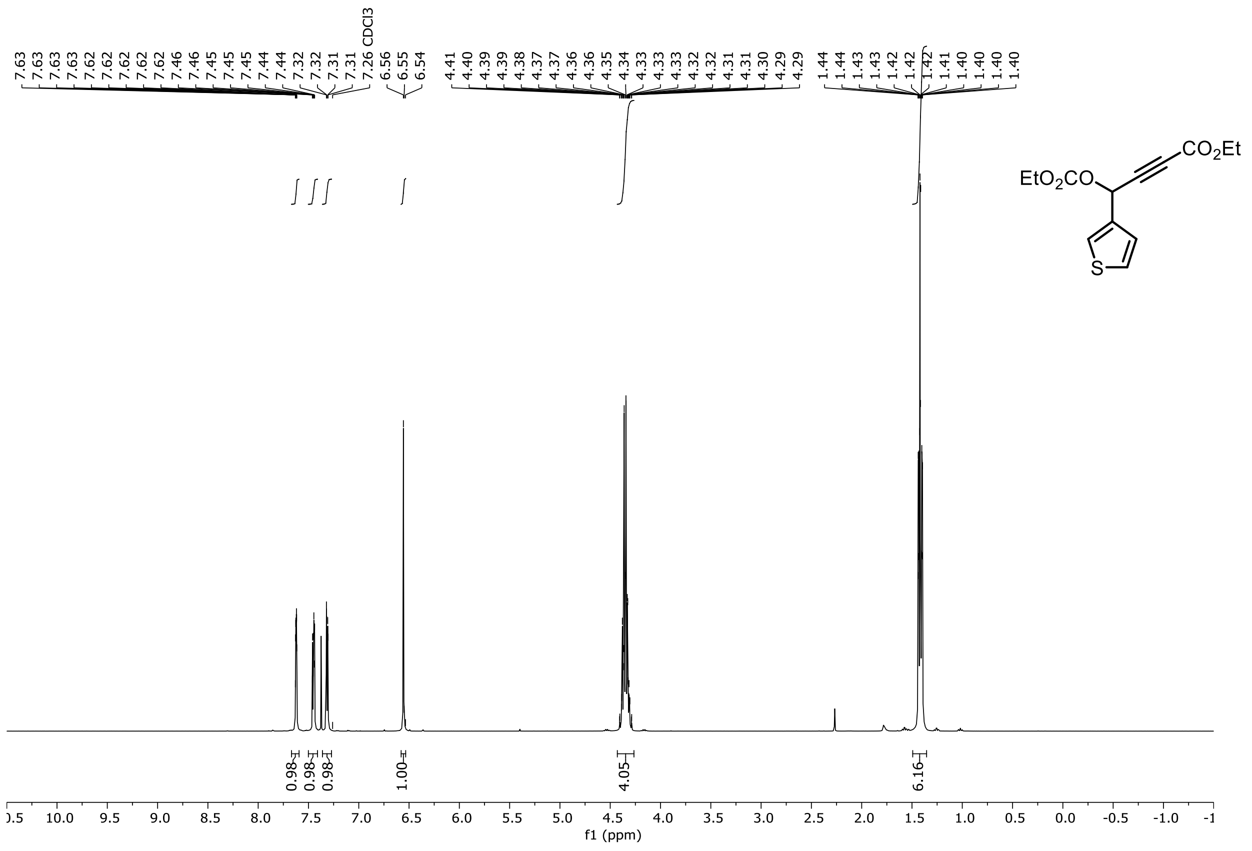

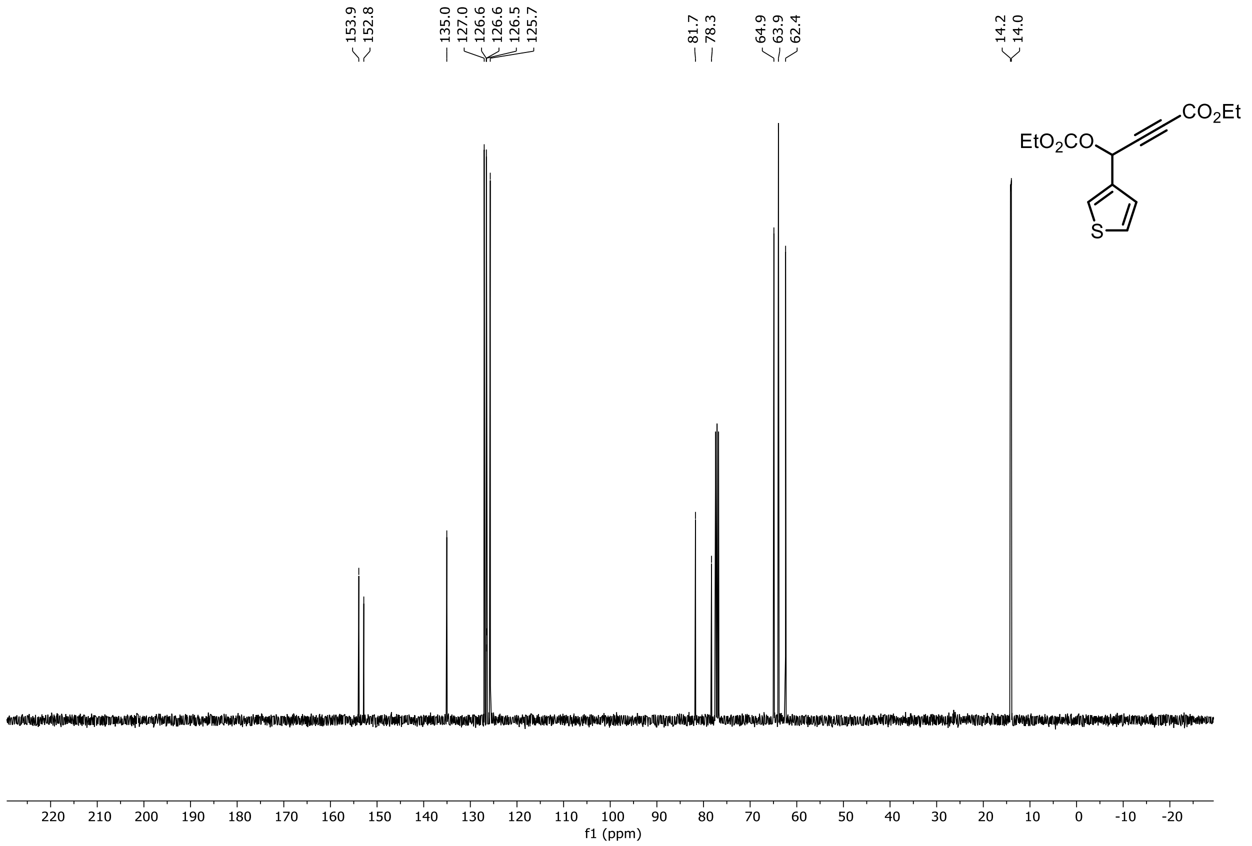

Figure 10.25 (top) ^{1}H NMR (400 MHz) and (bottom) ^{13}C NMR (101 MHz) spectra of **S15**.

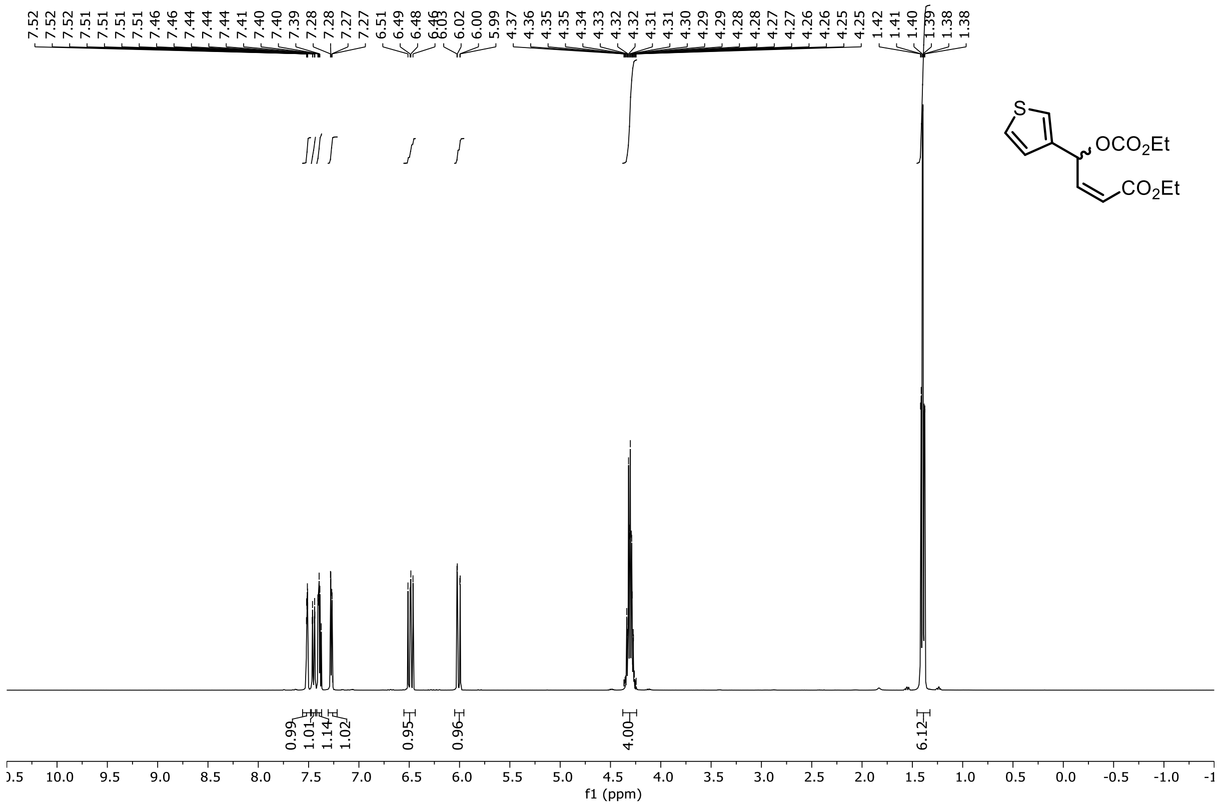

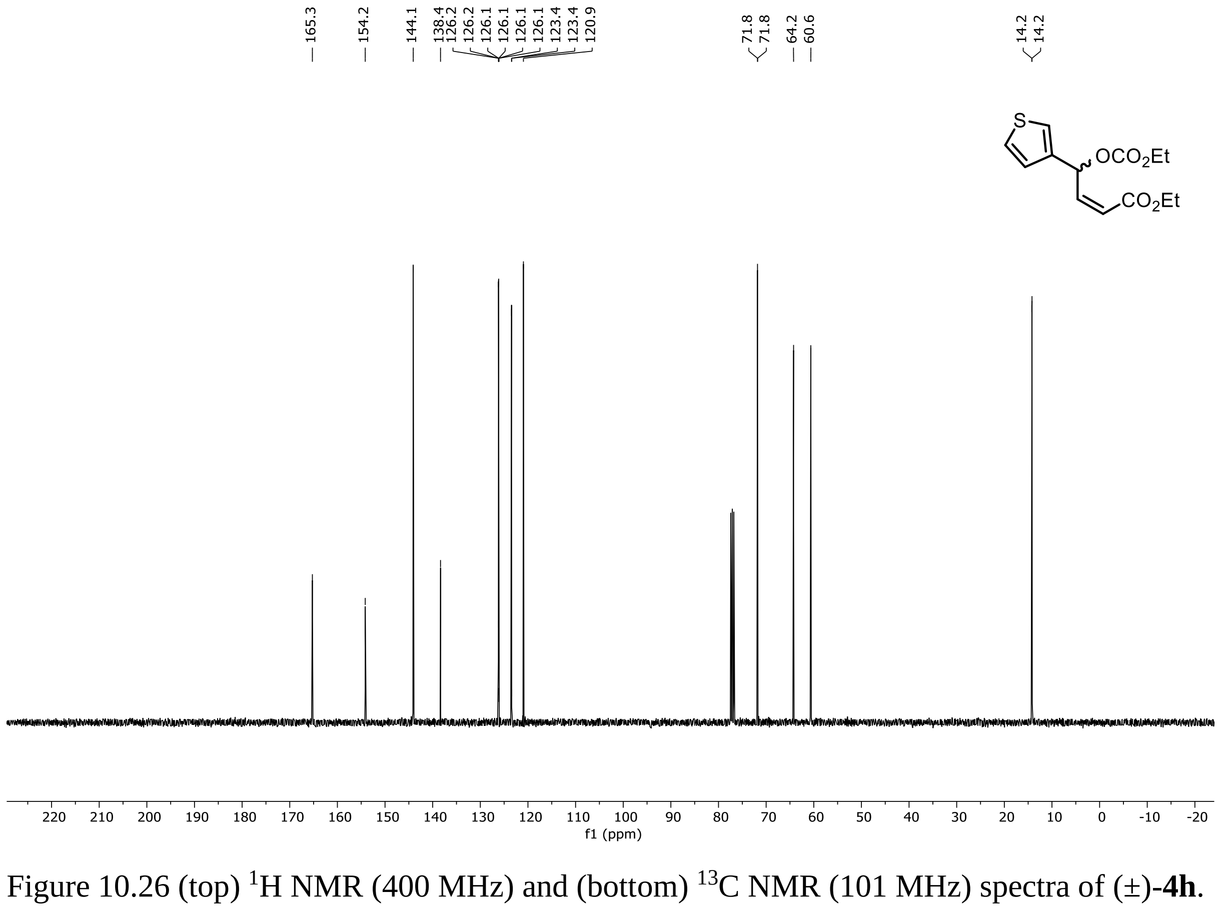

Figure 10.26 (top) ^{1}H NMR (400 MHz) and (bottom) ^{13}C NMR (101 MHz) spectra of (±)-**4h**.

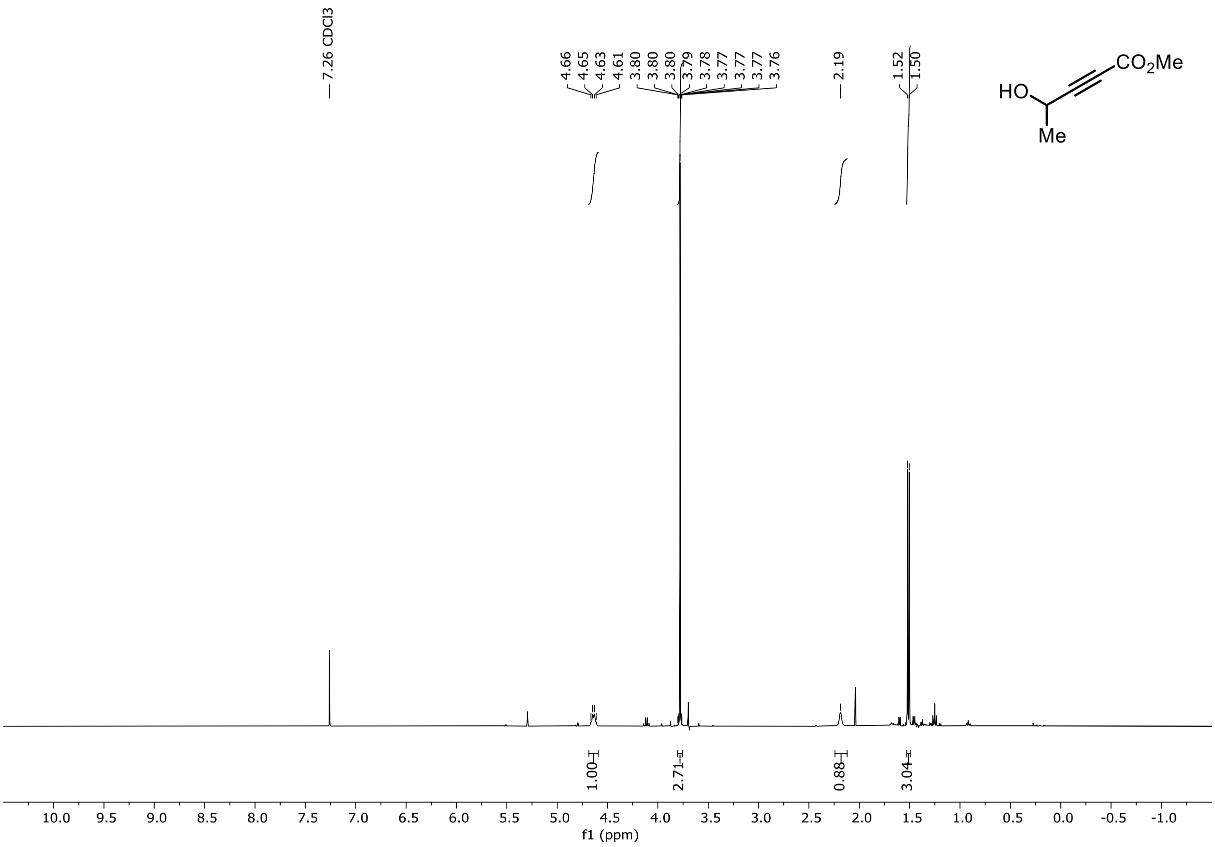

Figure 10.27 ^{1}H NMR (400 MHz) spectrum of **S16**.

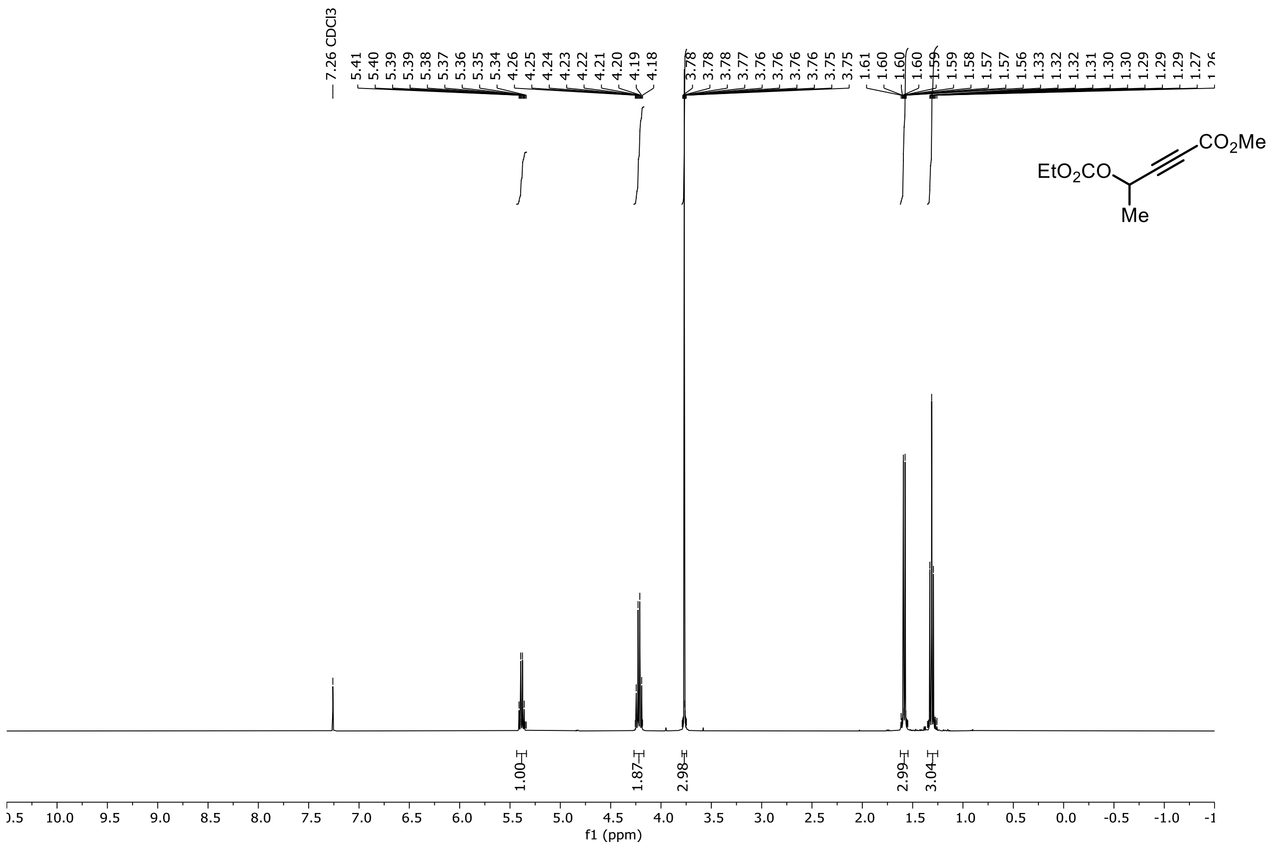

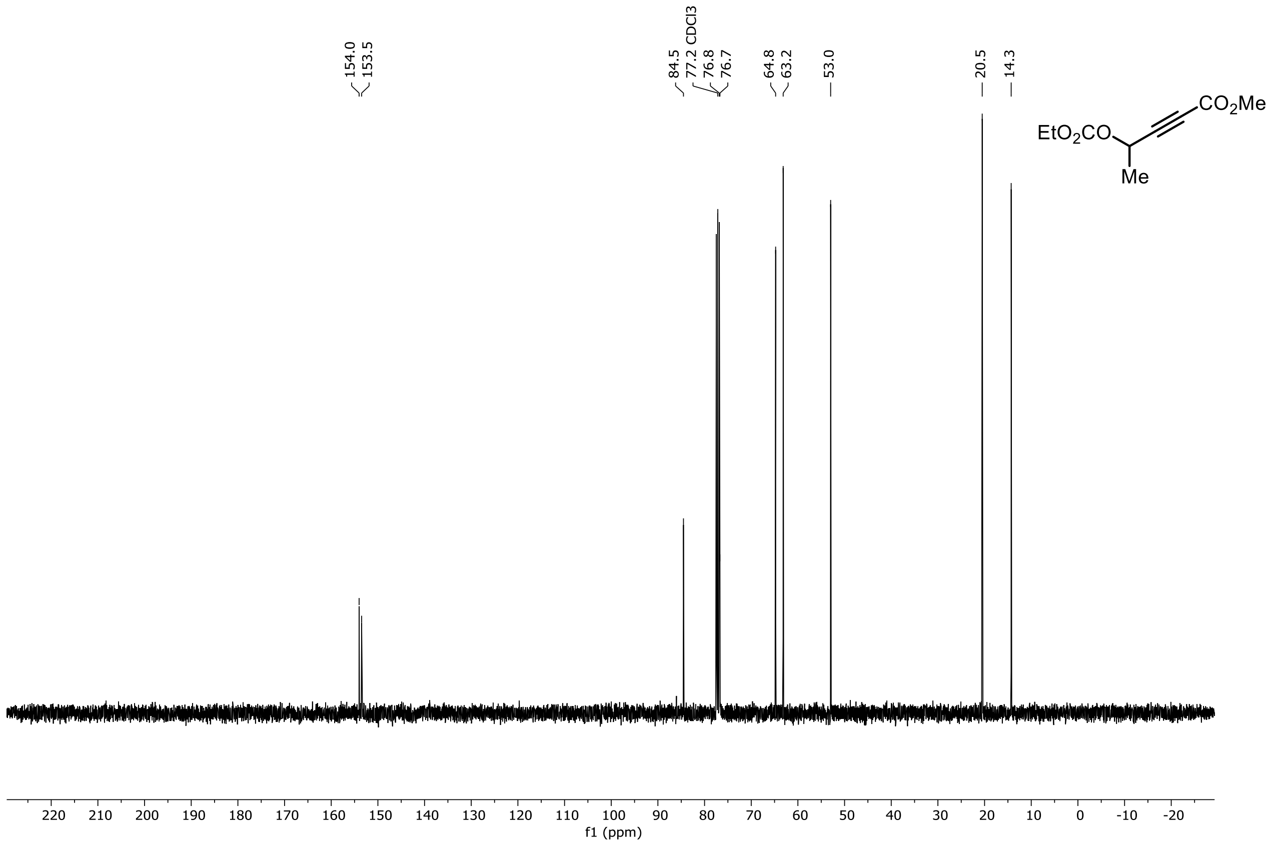

Figure 10.28 (top) ^{1}H NMR (400 MHz) and (bottom) ^{13}C NMR (101 MHz) spectra of **S17**.

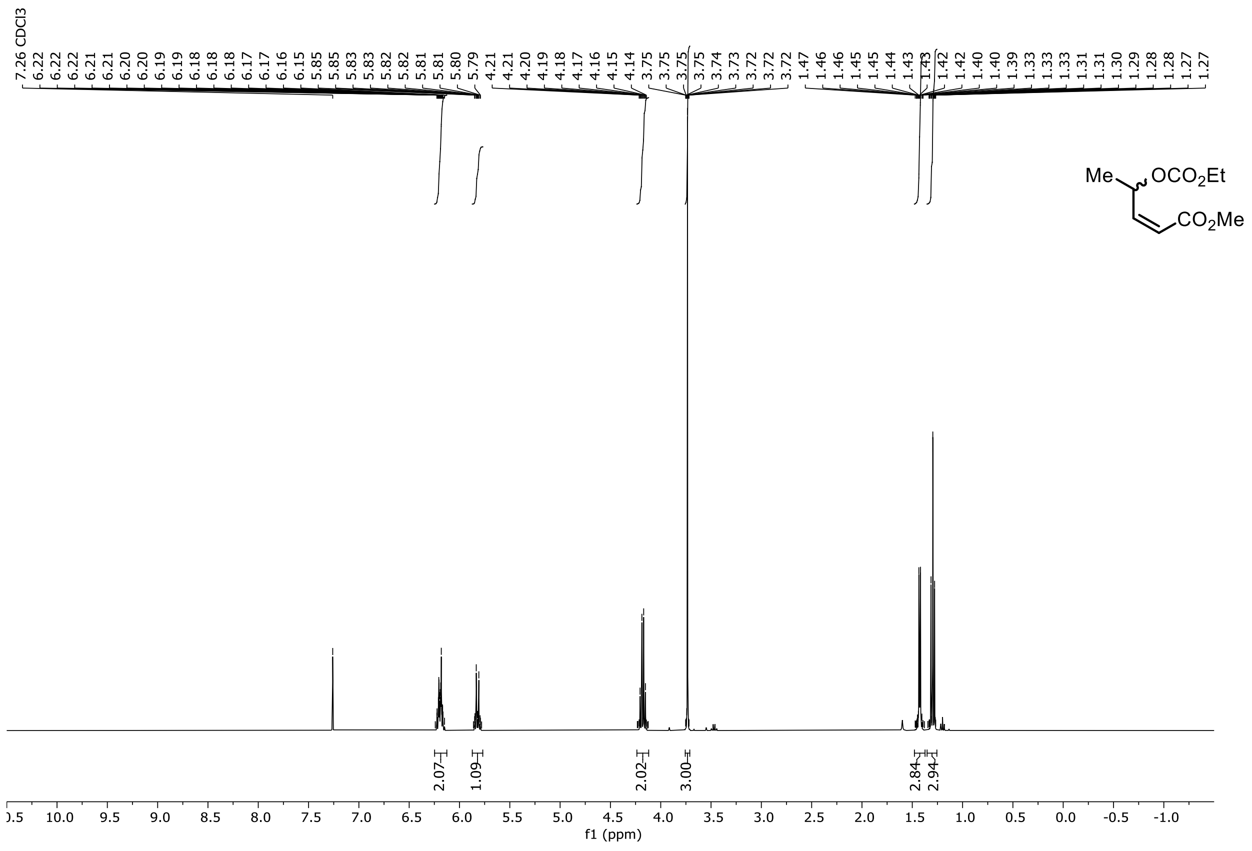

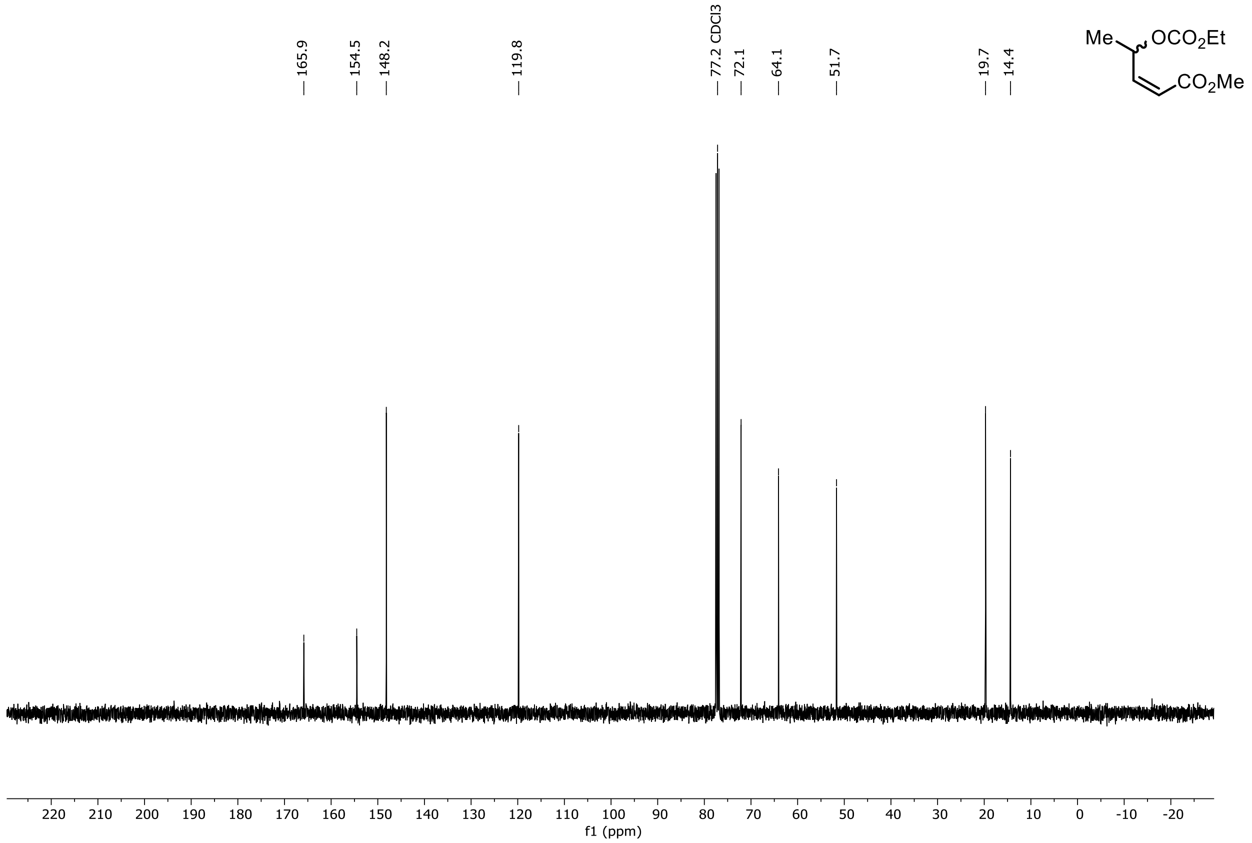

Figure 10.29 (top) ^{1}H NMR (400 MHz) and (bottom) ^{13}C NMR (101 MHz) spectra of (±)-**4i**.

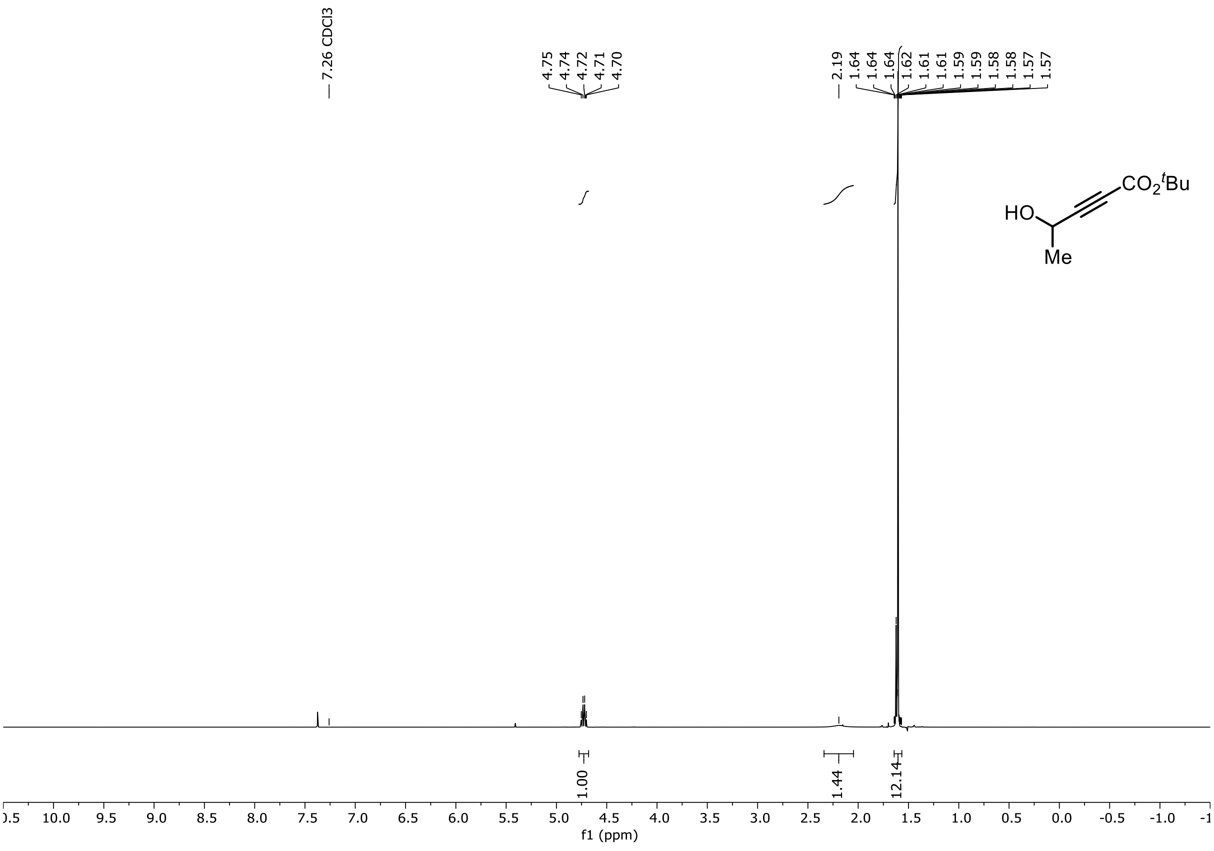

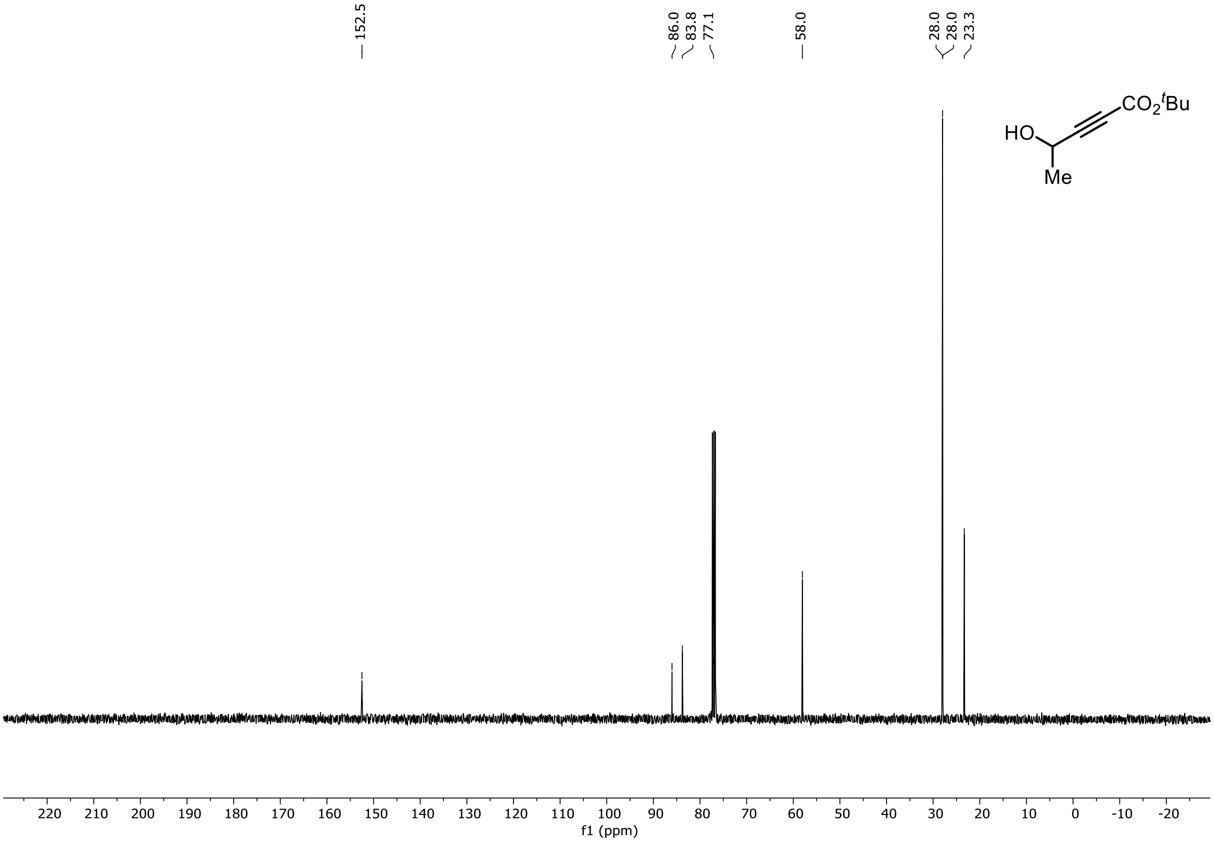

Figure 10.30 (top) ^{1}H NMR (400 MHz) and (bottom) ^{13}C NMR (101 MHz) spectra of **S18**.

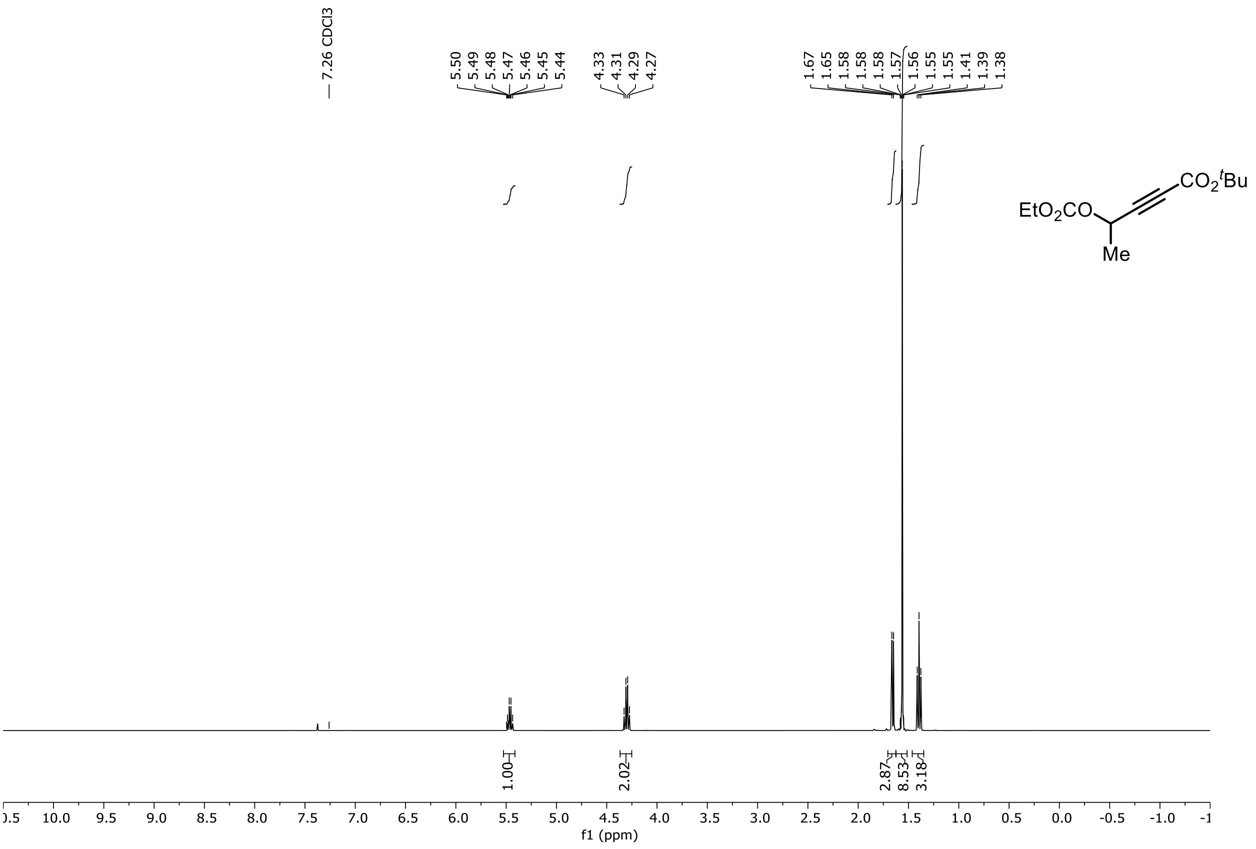

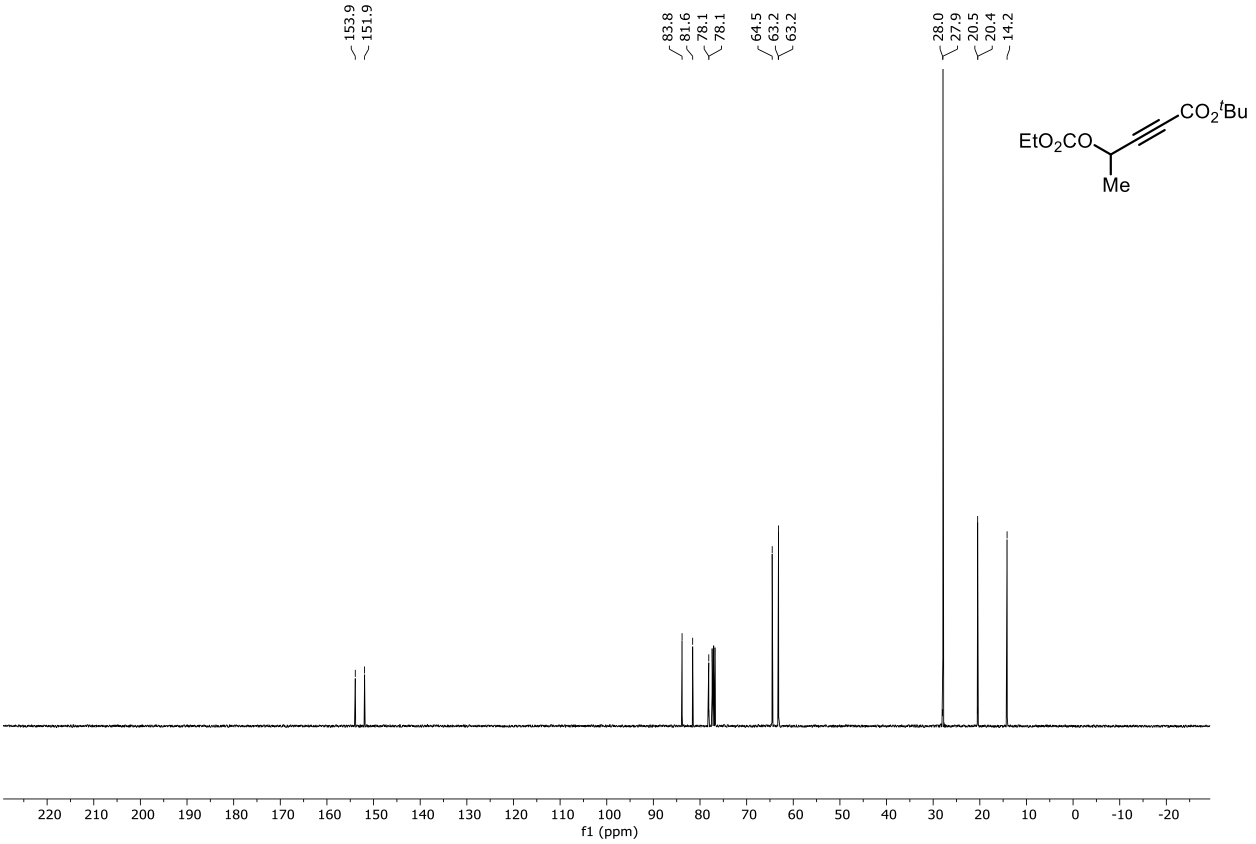

Figure 10.31 (top) ^{1}H NMR (400 MHz) and (bottom) ^{13}C NMR (101 MHz) spectra of **S19**.

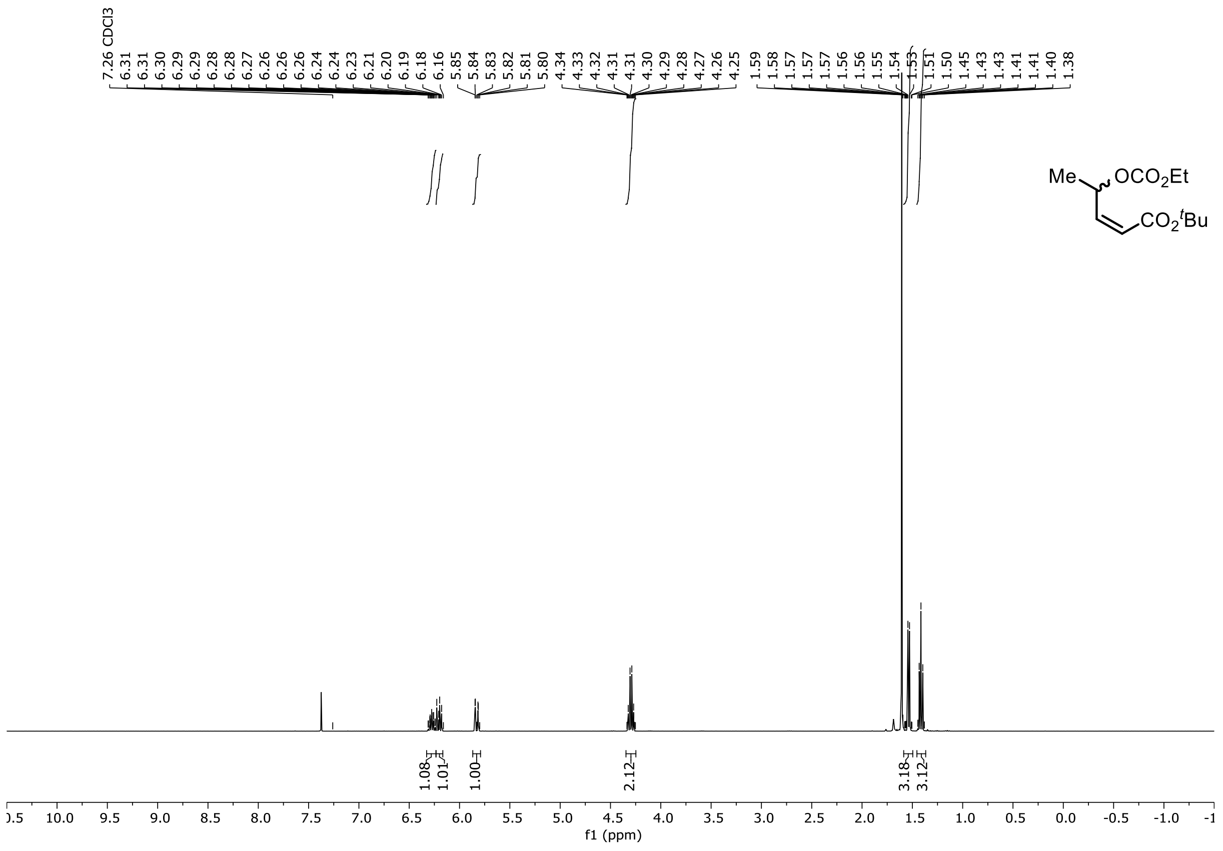

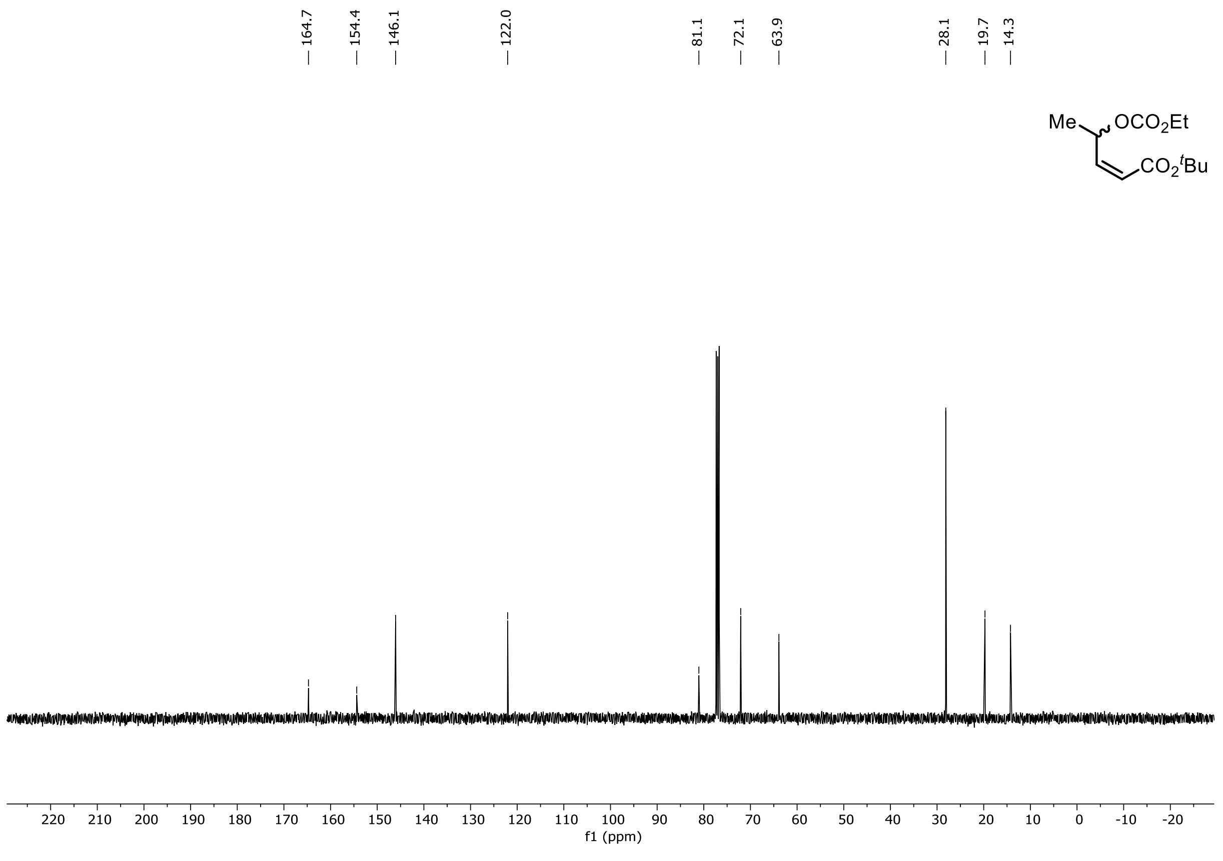

Figure 10.32 (top) ^{1}H NMR (400 MHz) and (bottom) ^{13}C NMR (101 MHz) spectra of (±)-**4j**.

10.2. Spectra of the coupling products

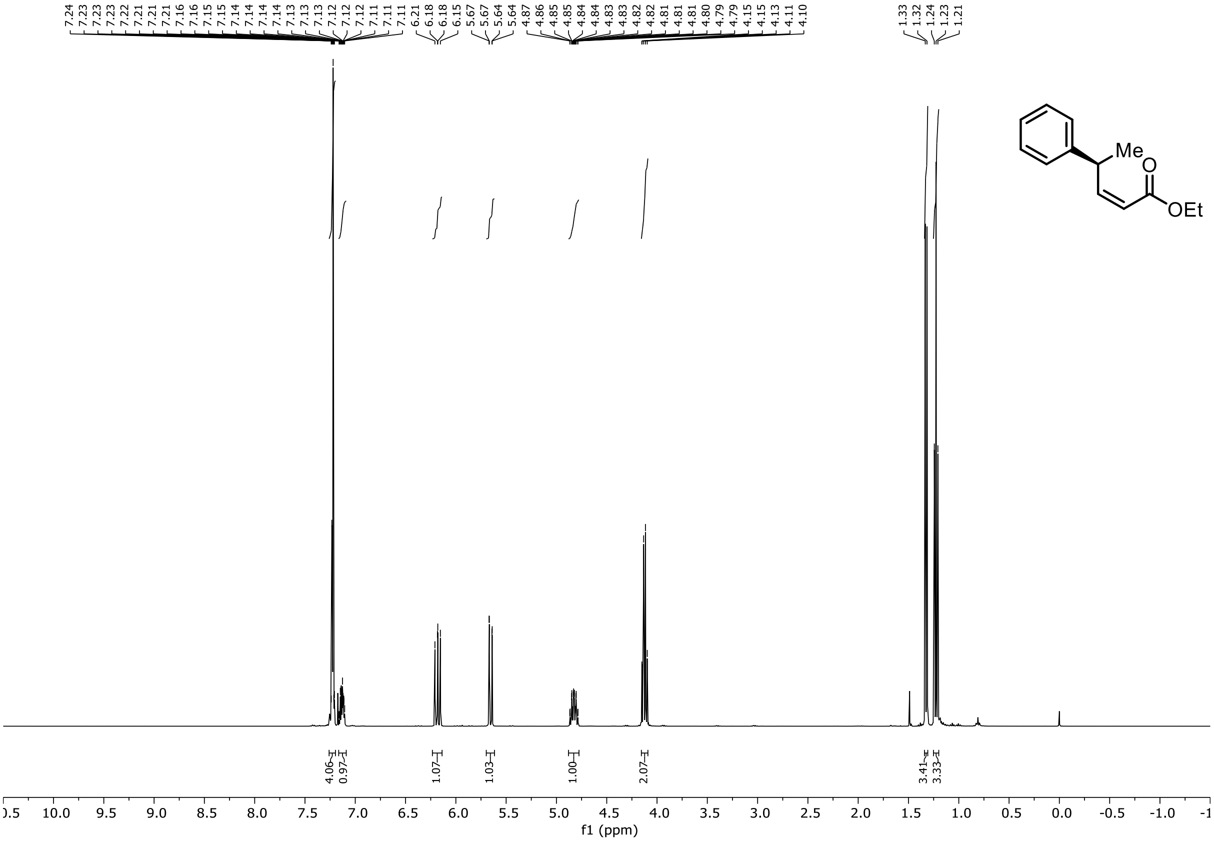

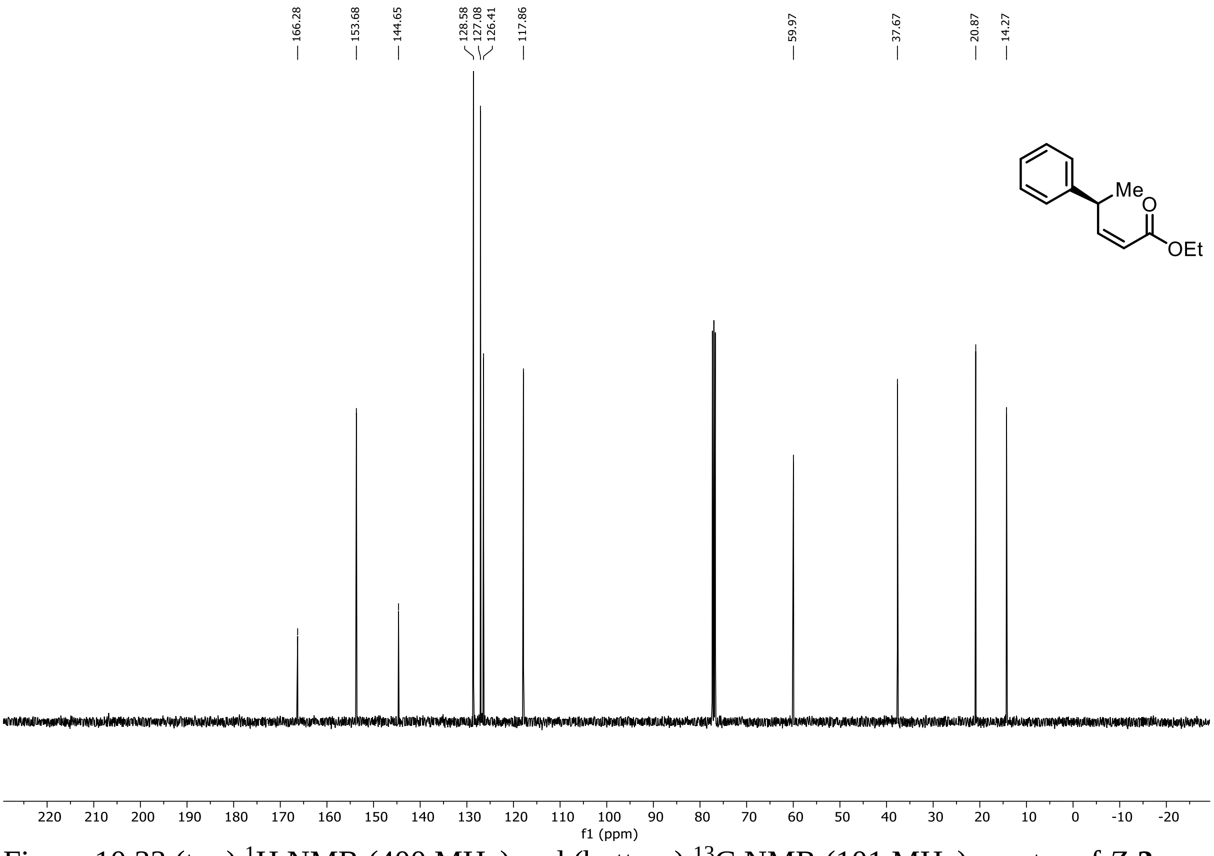

Figure 10.33 (top) ^{1}H NMR (400 MHz) and (bottom) ^{13}C NMR (101 MHz) spectra of *Z*-**3a**.

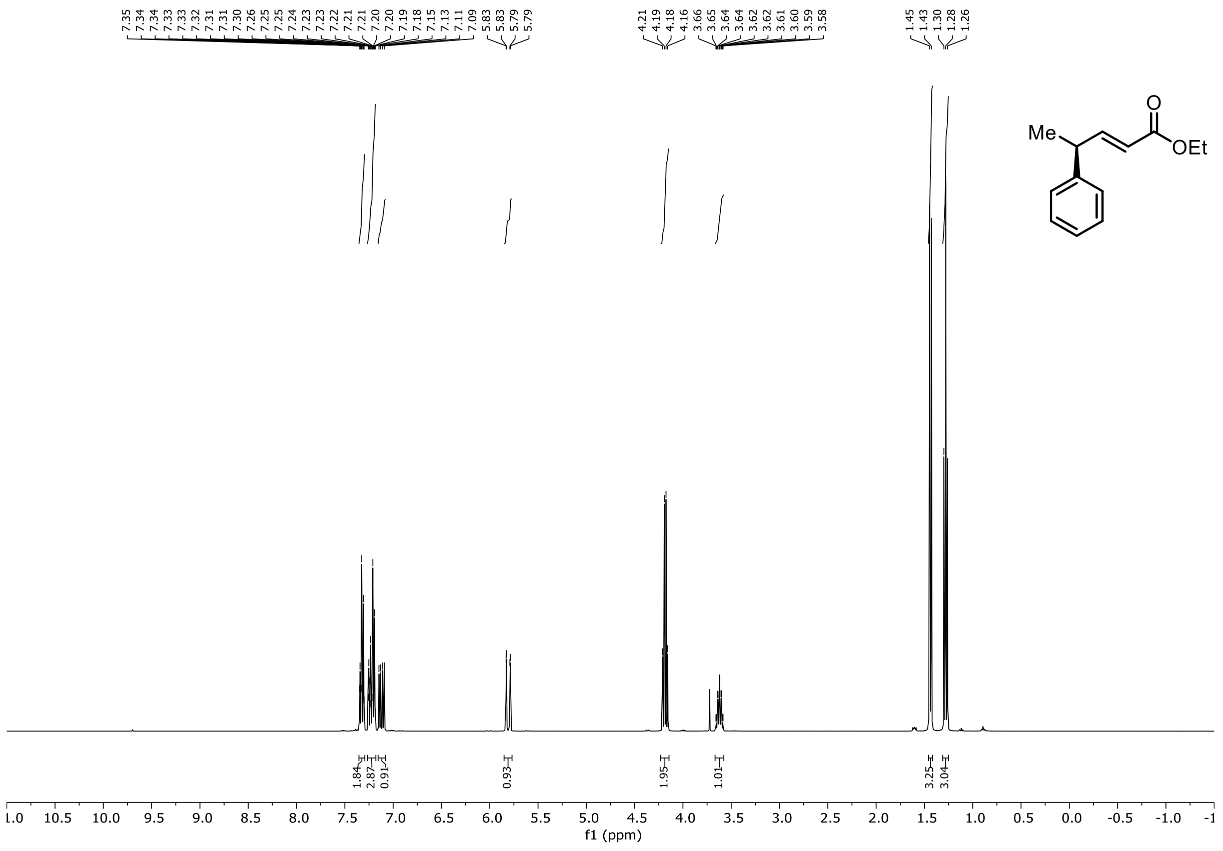

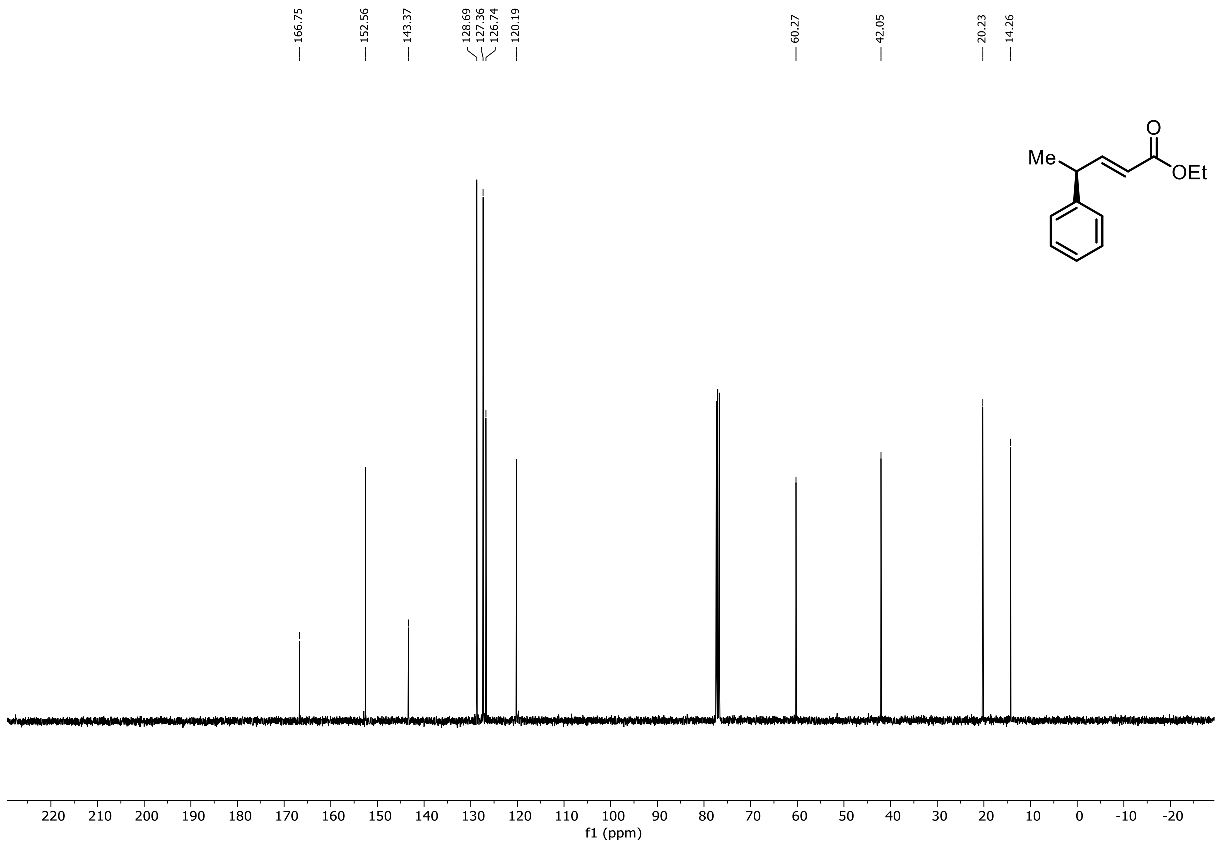

Figure 10.34 (top) ^{1}H NMR (400 MHz) and (bottom) ^{13}C NMR (101 MHz) spectra of *E*-**3a**.

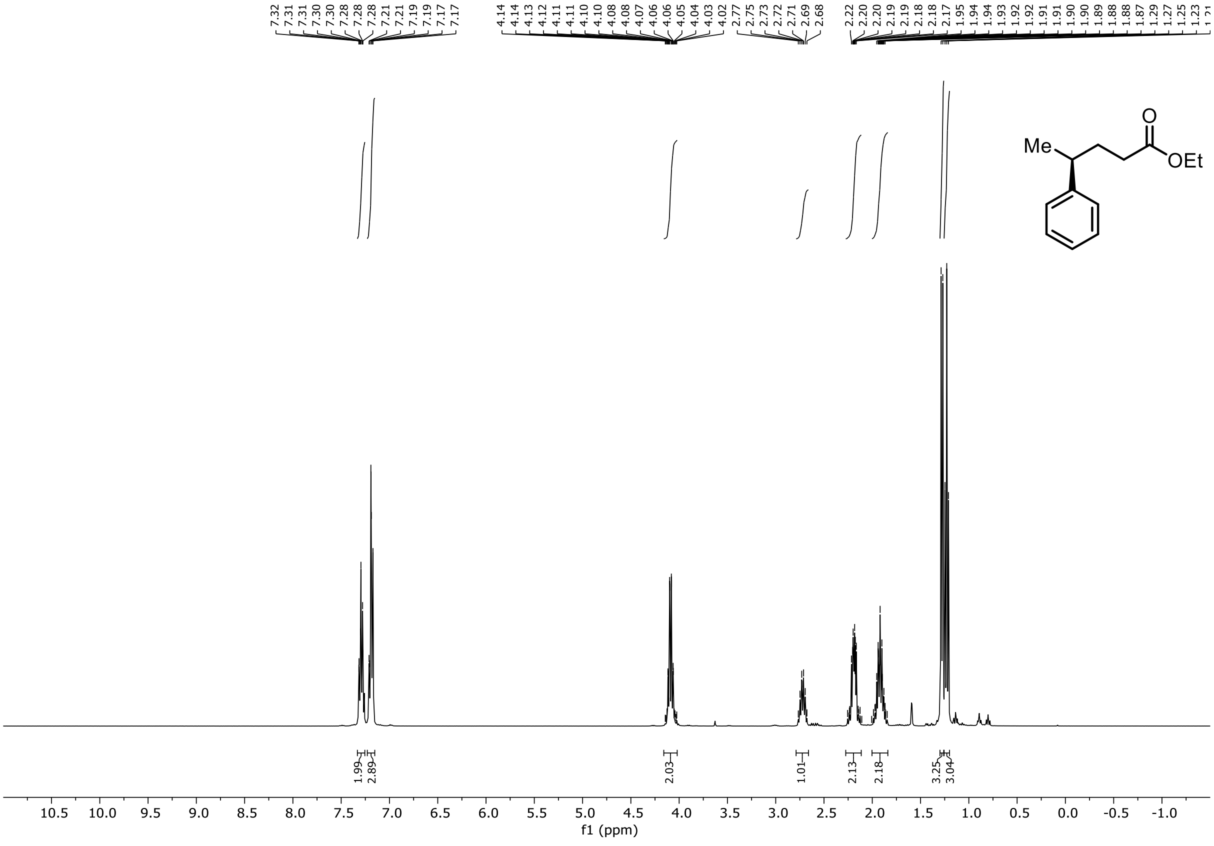

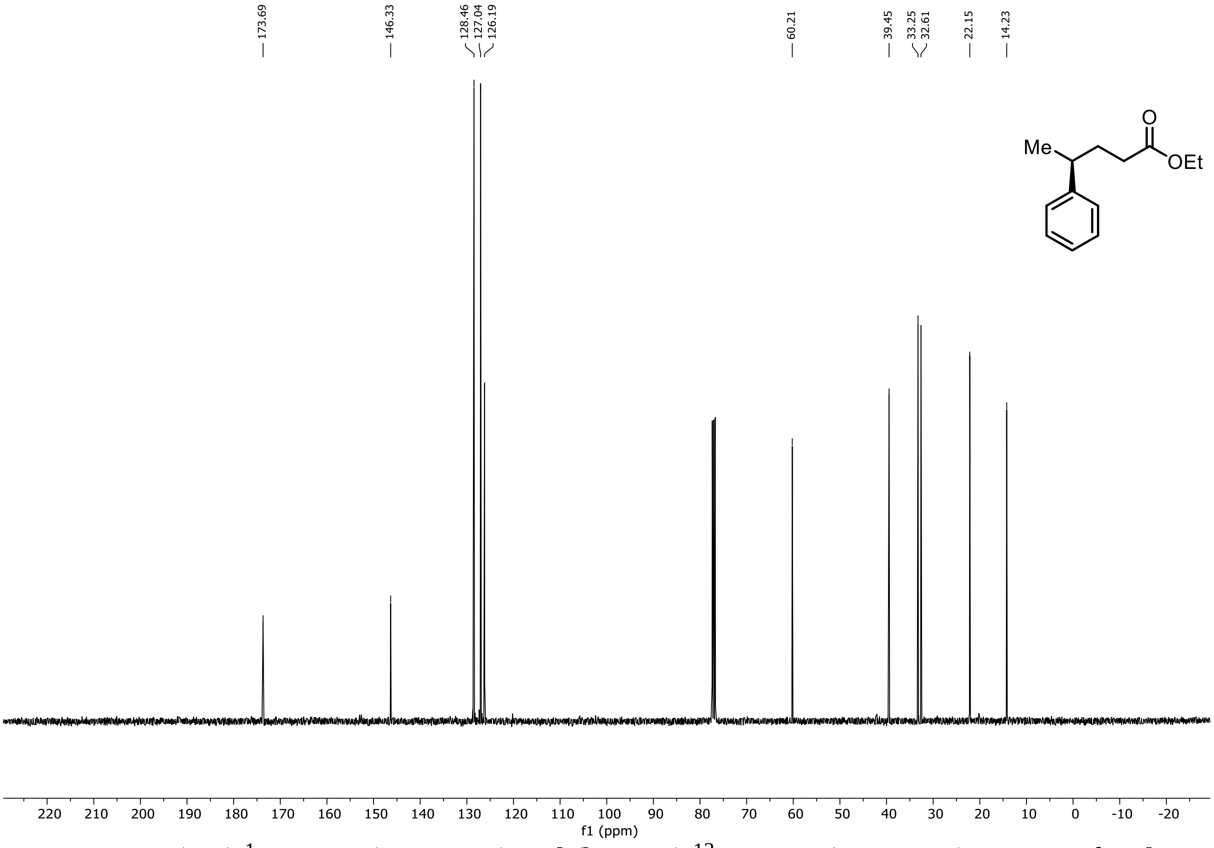

Figure 10.35 (top) ^{1}H NMR (400 MHz) and (bottom) ^{13}C NMR (101 MHz) spectra of *red*-**3a**.

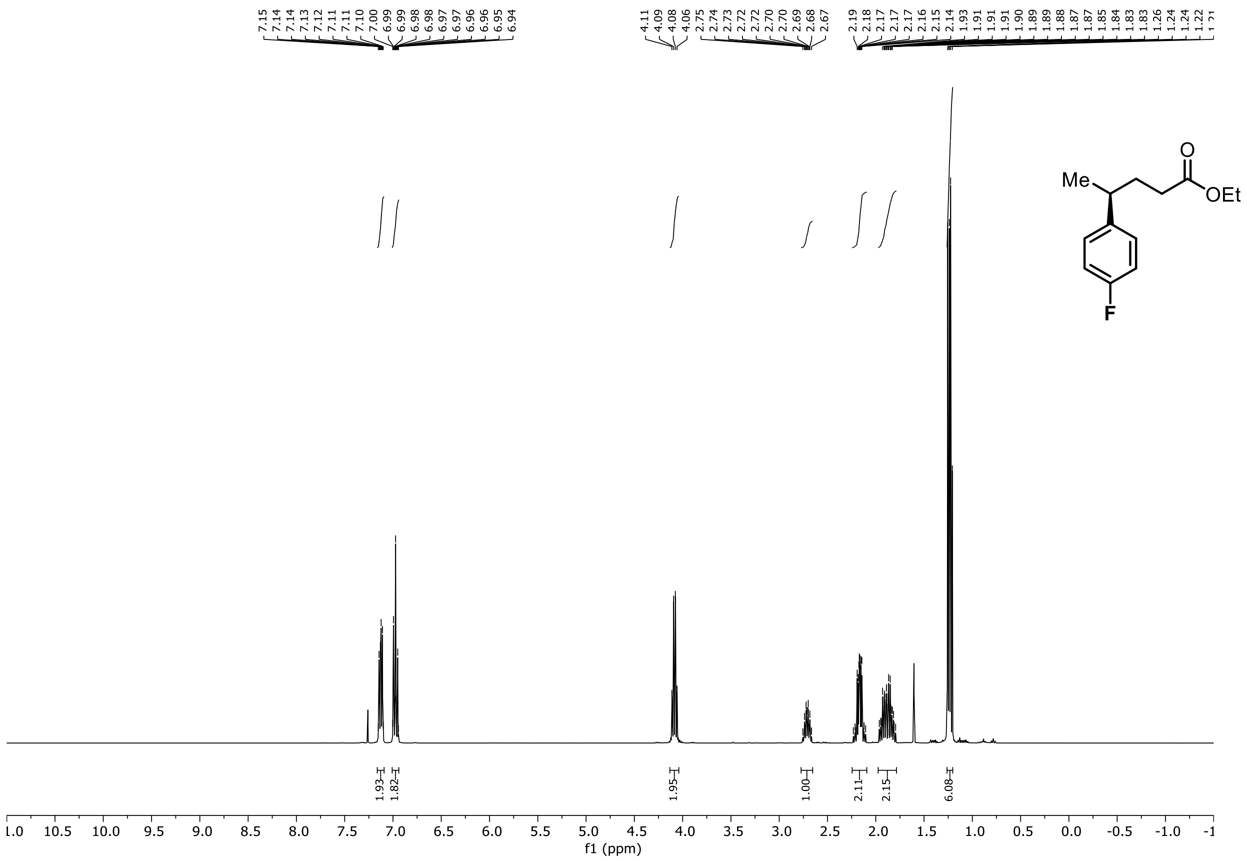

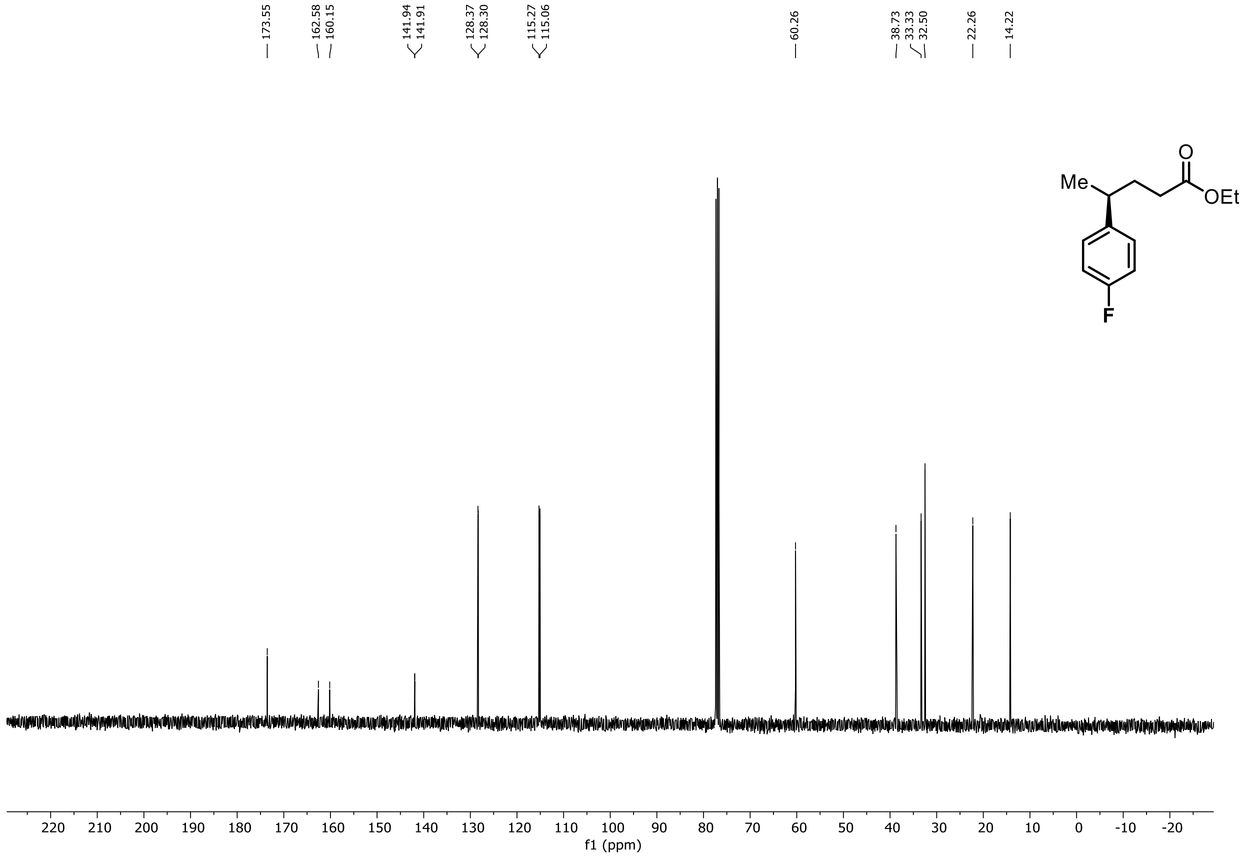

Figure 10.36 (top) ^{1}H NMR (400 MHz) and (bottom) ^{13}C NMR (101 MHz) spectra of *red*-**3b**.

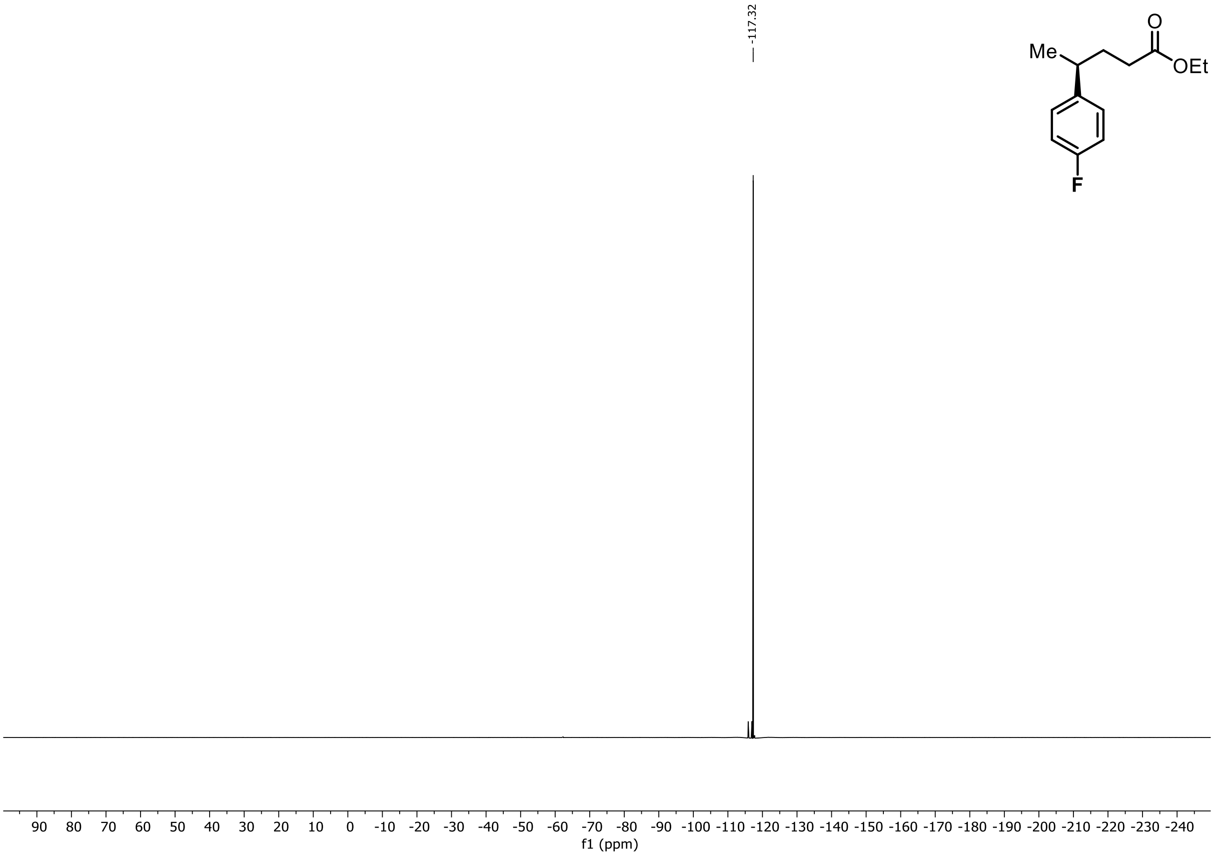

Figure 10.37 ^{19}F (13C)NMR (376 MHz) spectrum of *red*-**3b**.

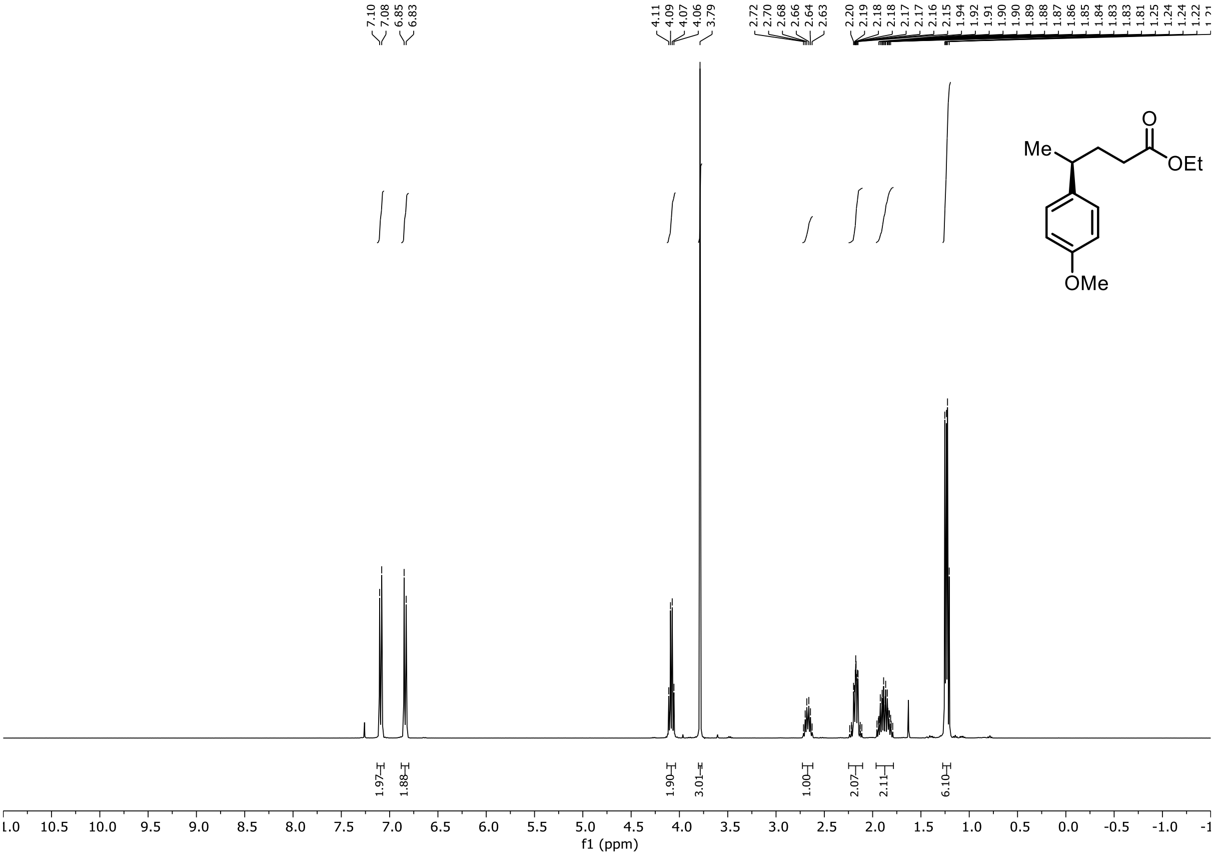

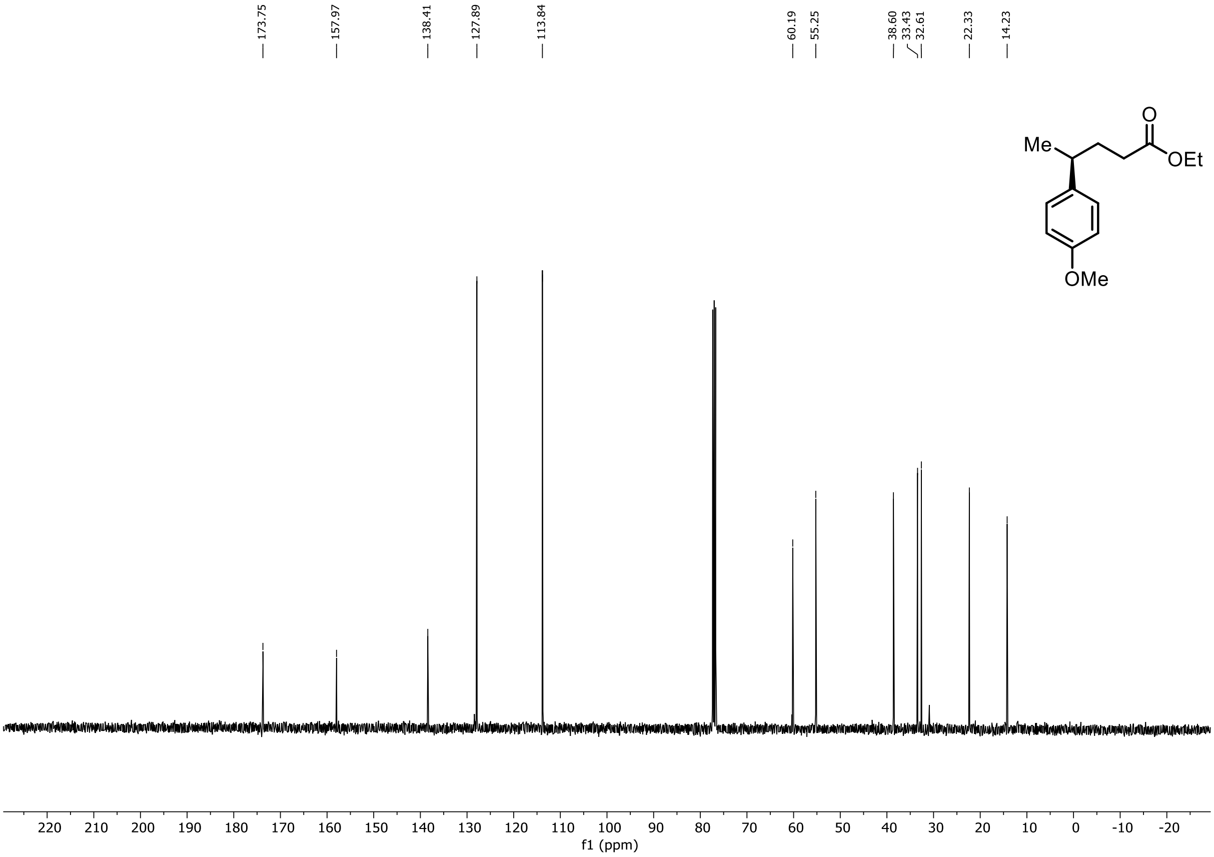

Figure 10.38 (top) ^{1}H NMR (400 MHz) and (bottom) ^{13}C NMR (101 MHz) spectra of *red*-**3c**.

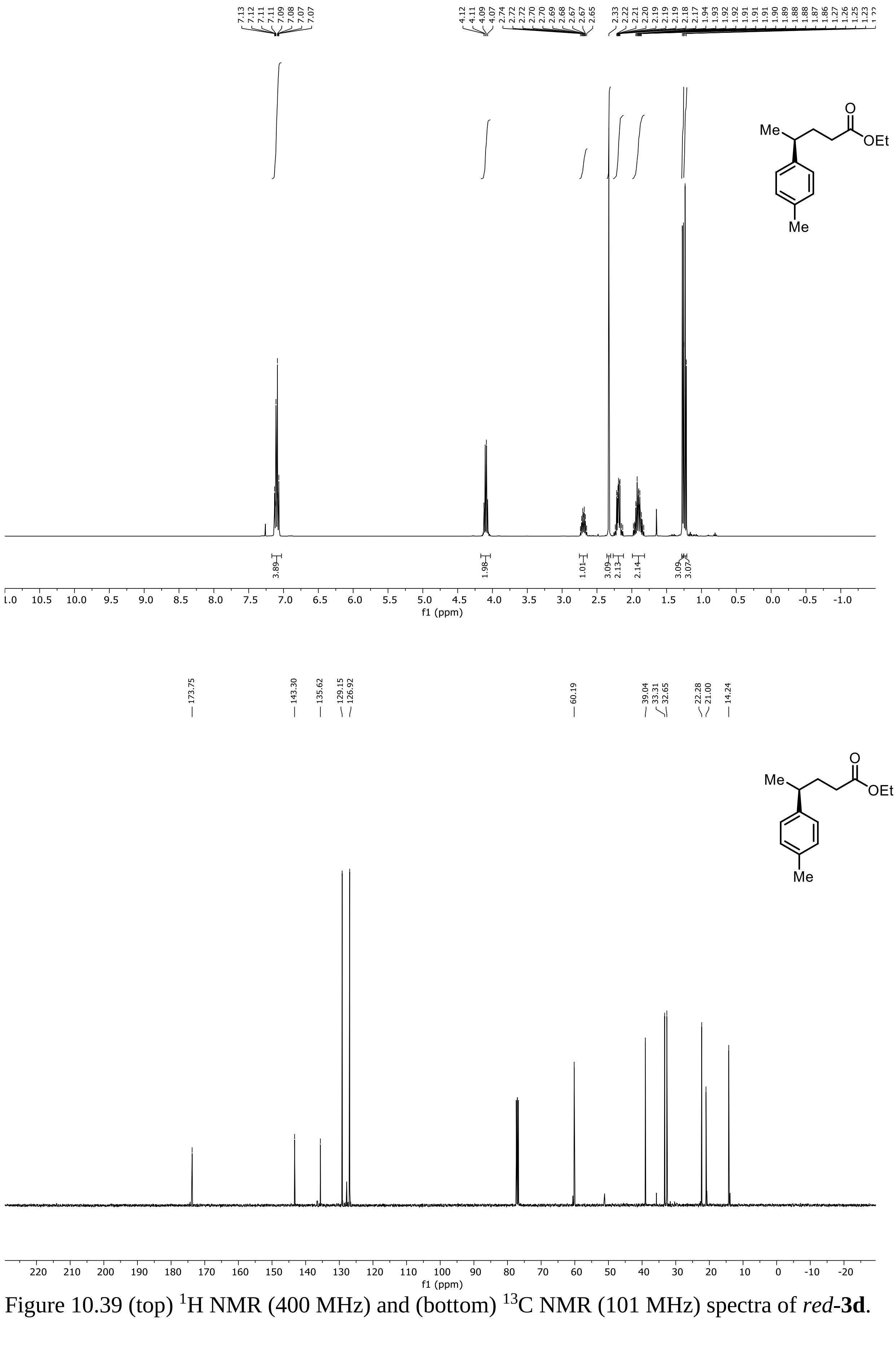

Figure 10.39 (top) ^{1}H NMR (400 MHz) and (bottom) ^{13}C NMR (101 MHz) spectra of *red*-**3d**.

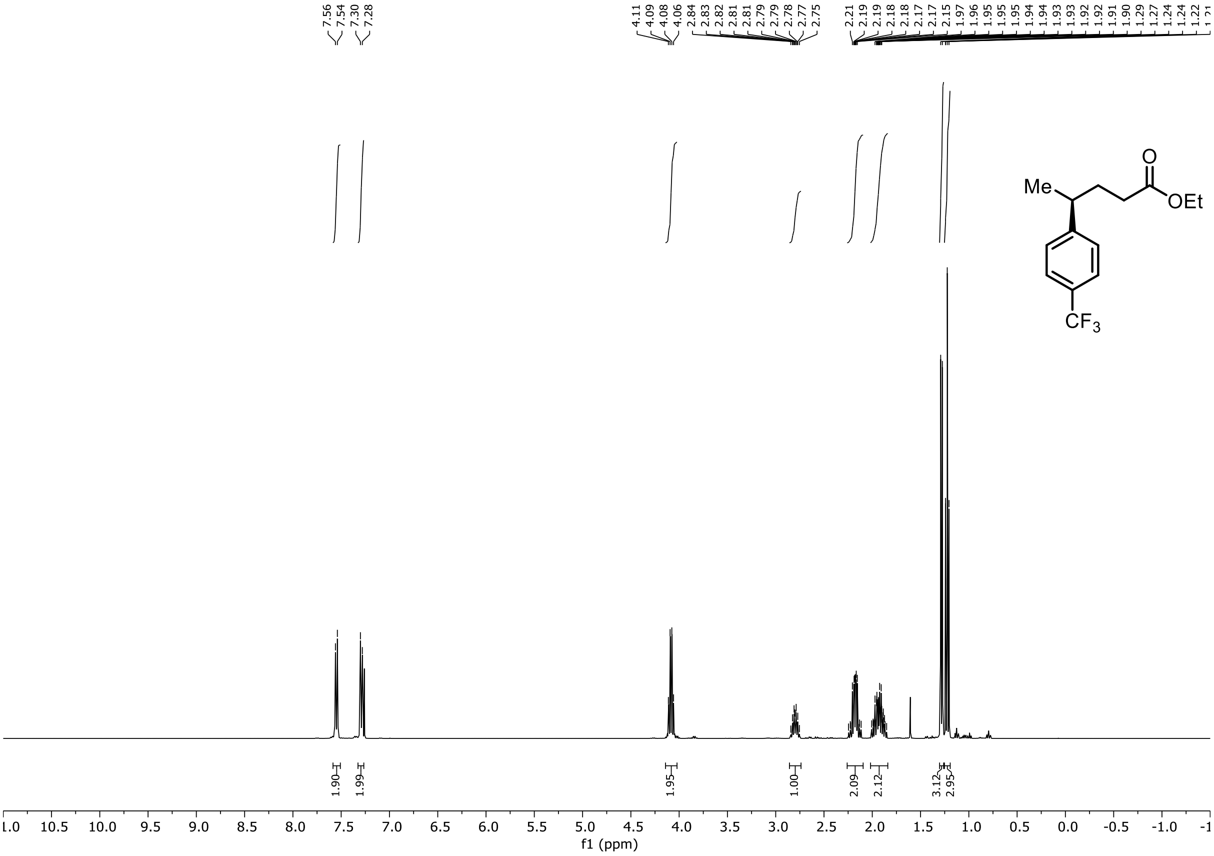

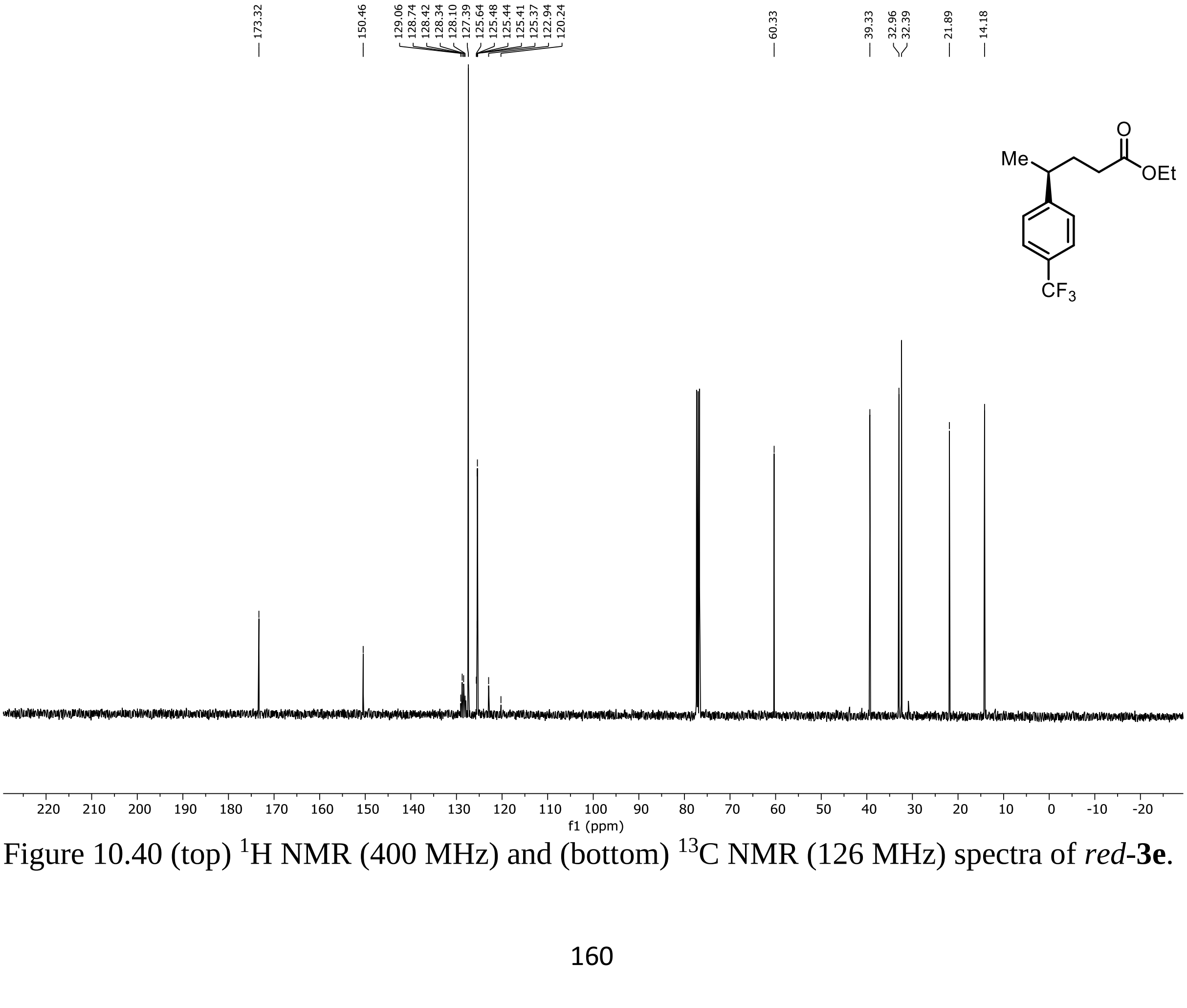

Figure 10.40 (top) ^{1}H NMR (400 MHz) and (bottom) ^{13}C NMR (126 MHz) spectra of *red*-**3e**.

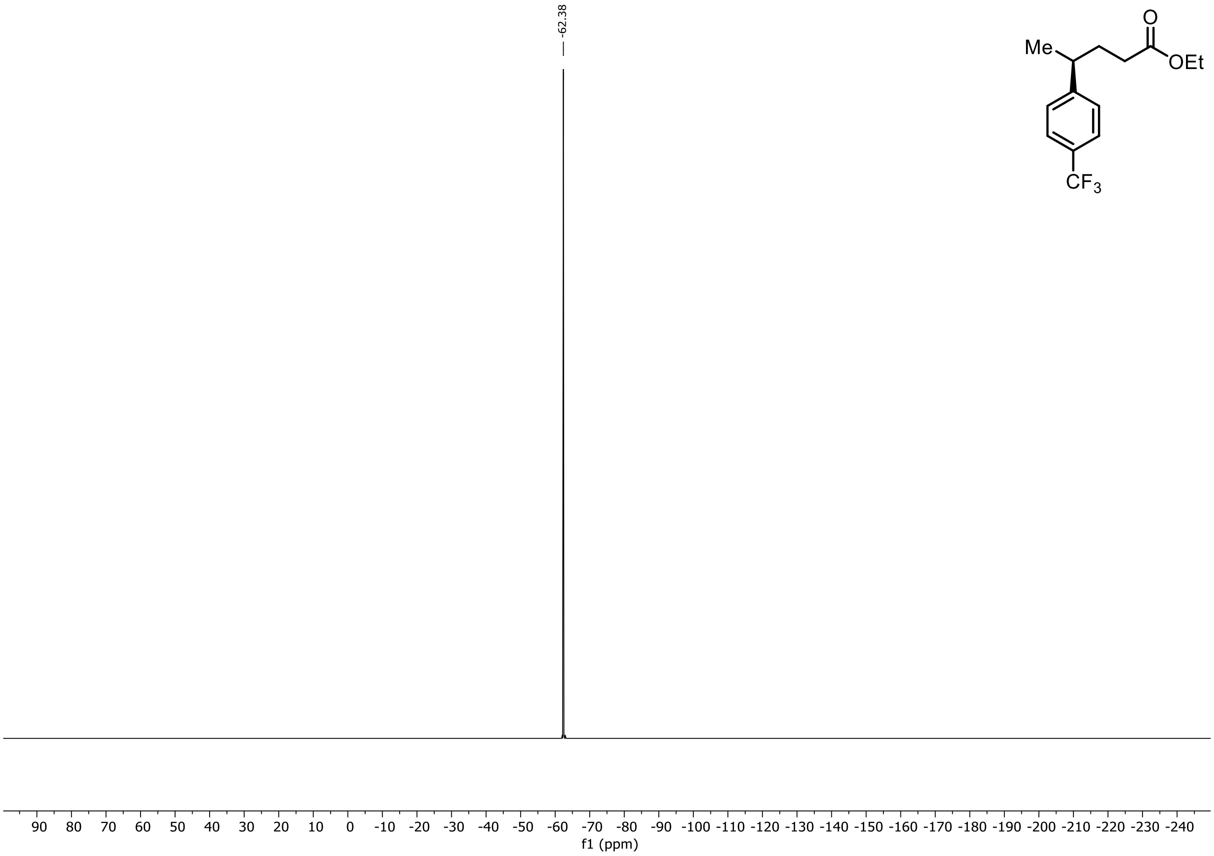

Figure 10.41 ^{19}F (13C)NMR (376 MHz) spectrum of *red*-**3e**.

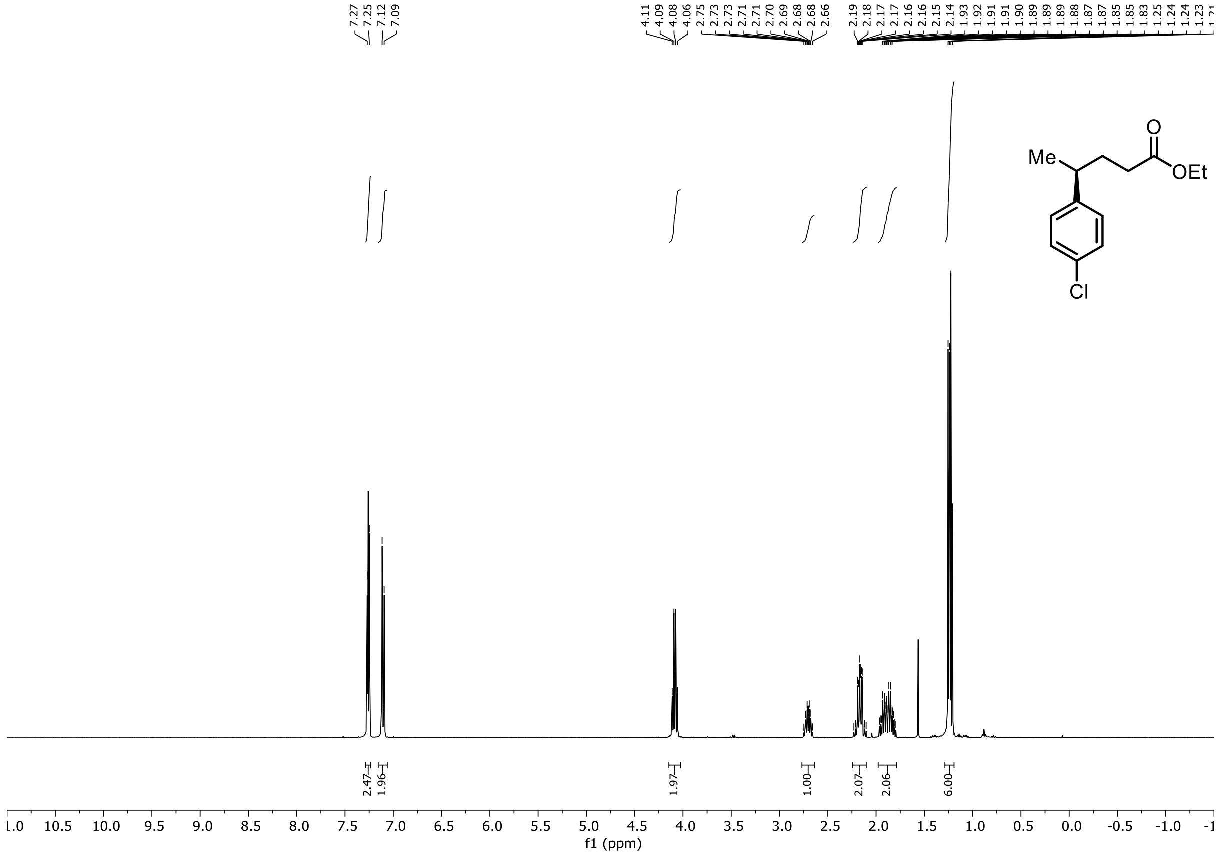

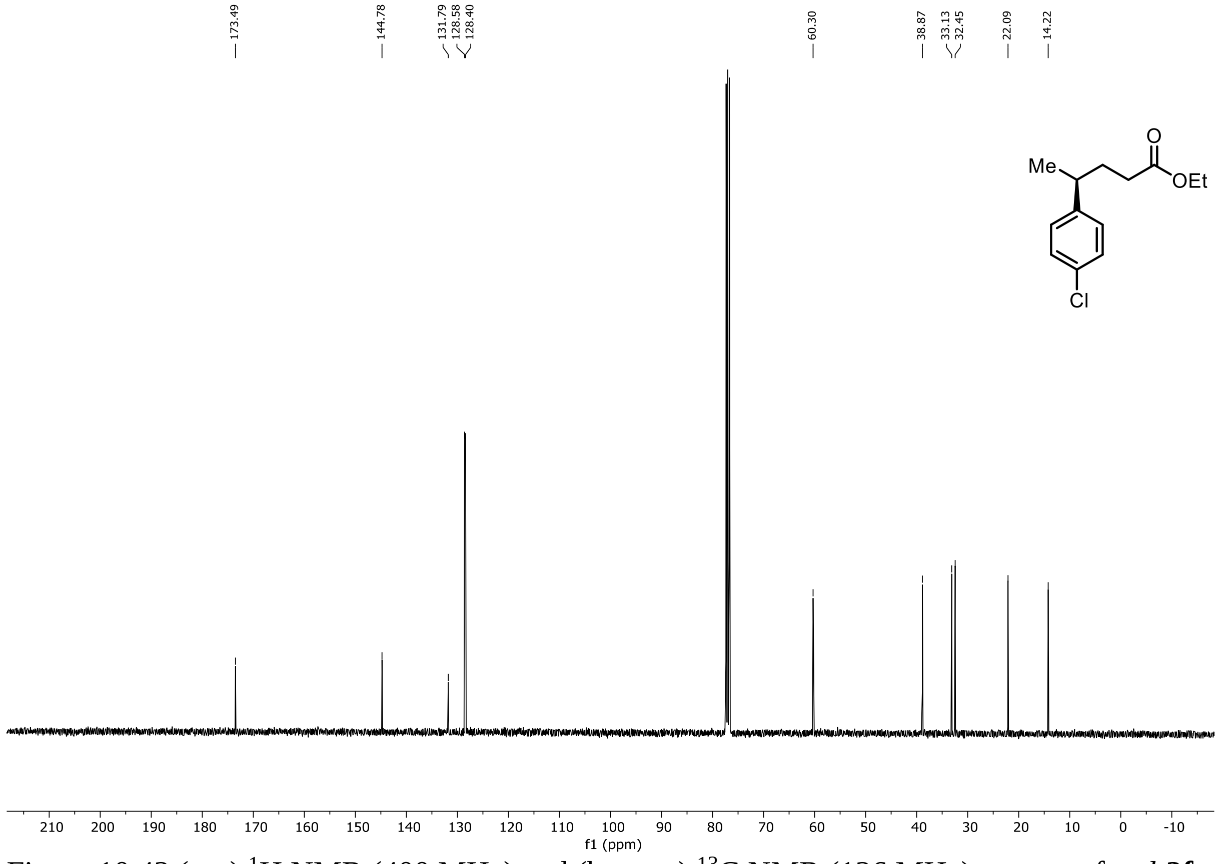

Figure 10.42 (top) ^{1}H NMR (400 MHz) and (bottom) ^{13}C NMR (126 MHz) spectra of *red*-**3f**.

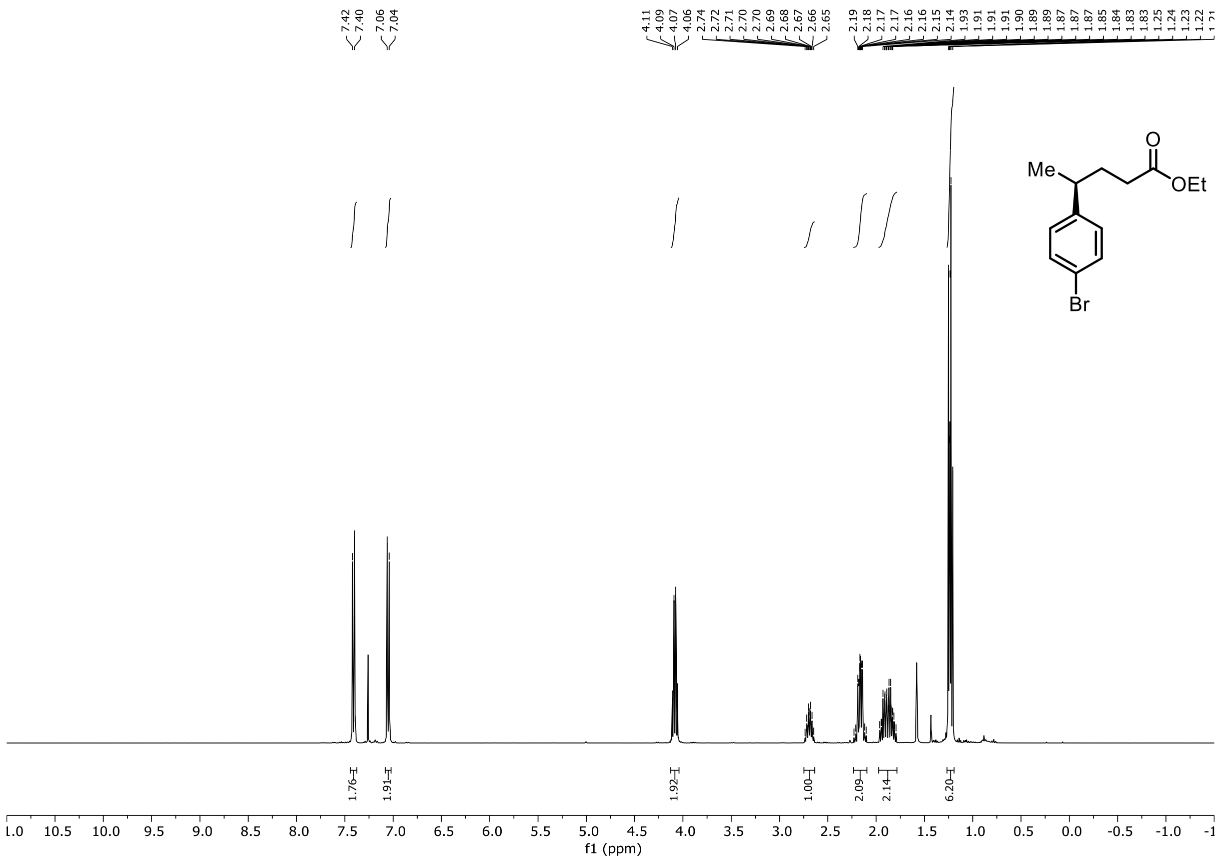

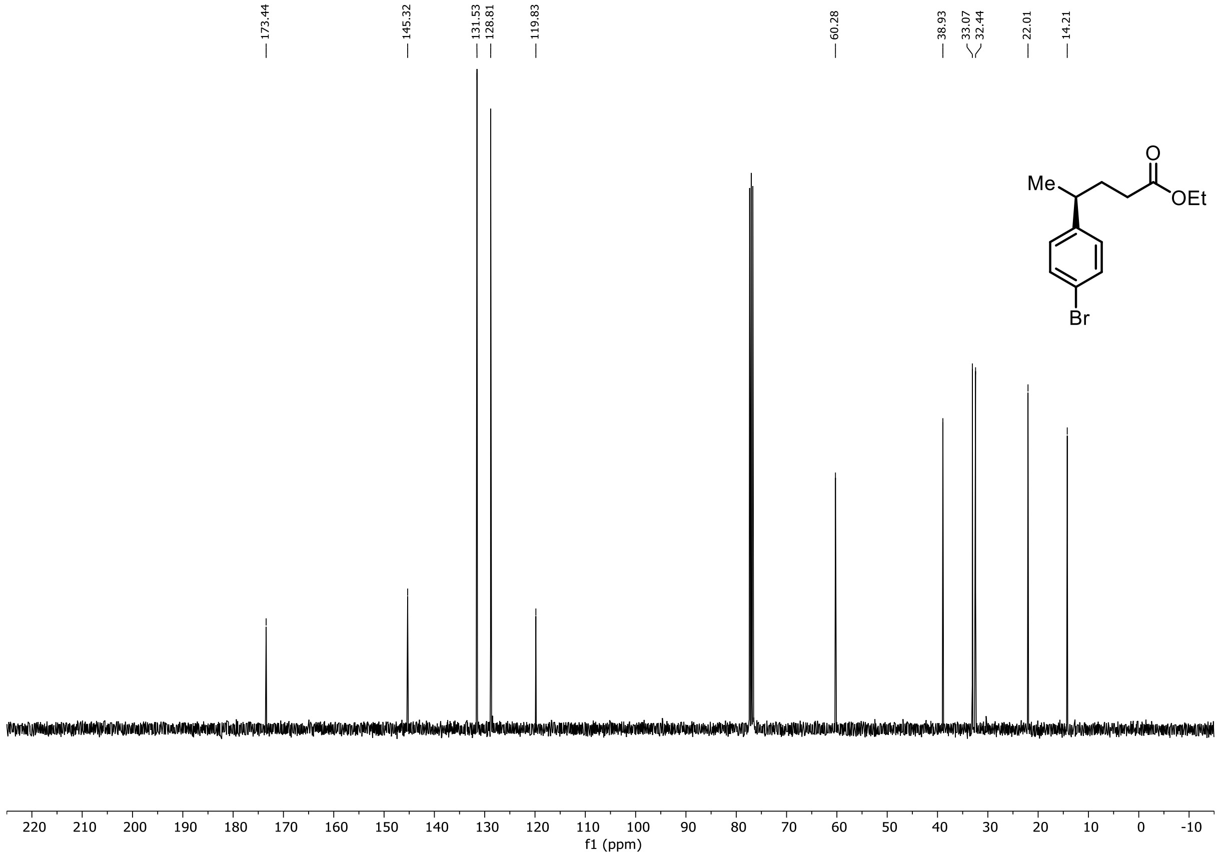

Figure 10.43 (top) ^{1}H NMR (400 MHz) and (bottom) ^{13}C NMR (101 MHz) spectra of *red*-**3g**.

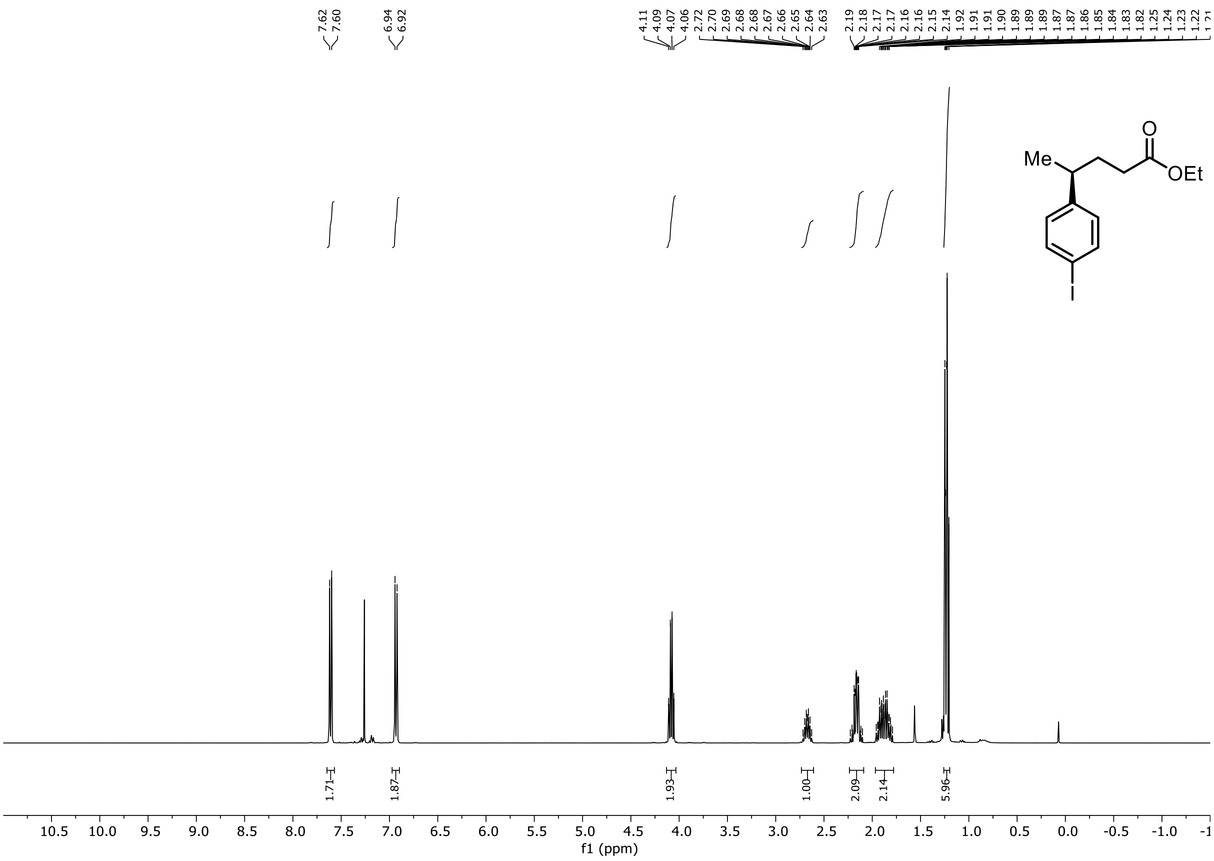

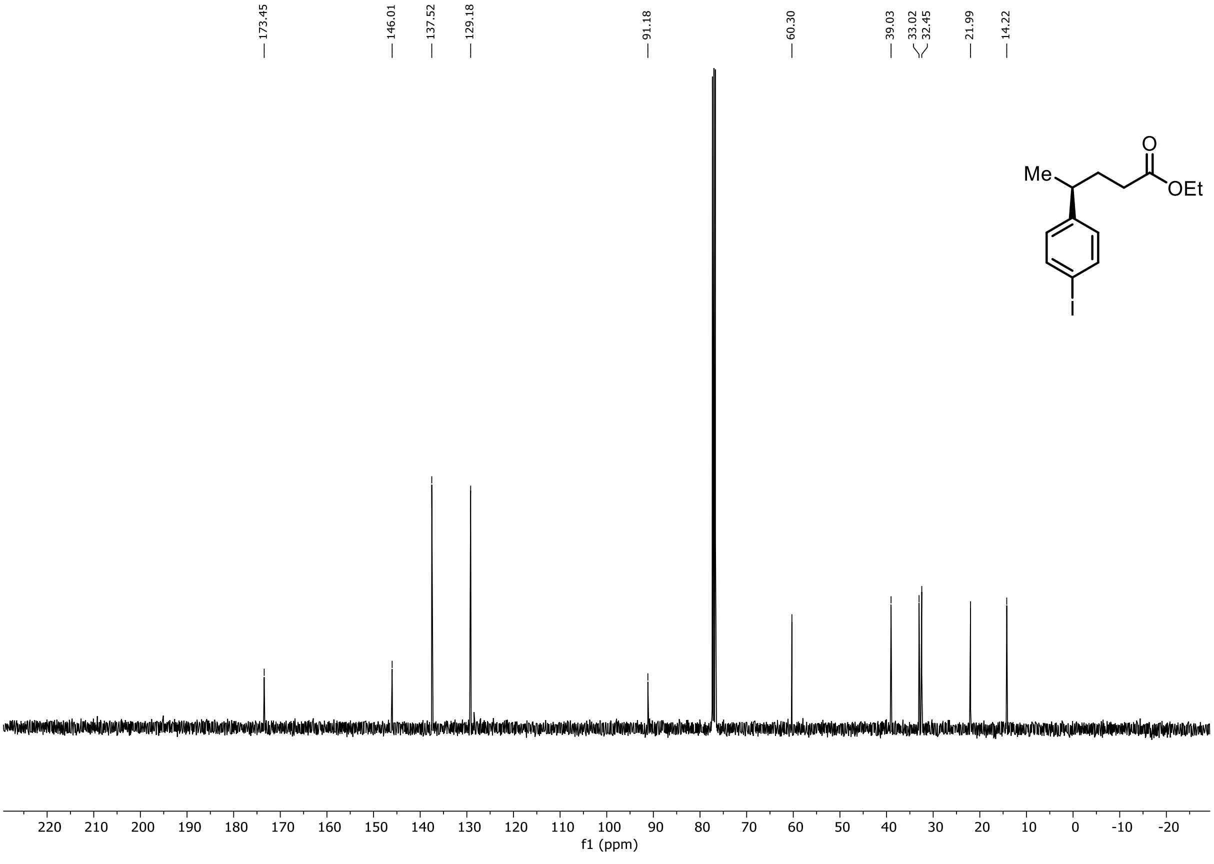

Figure 10.44 (top) ^{1}H NMR (400 MHz) and (bottom) ^{13}C NMR (101 MHz) spectra of *red*-**3h**.

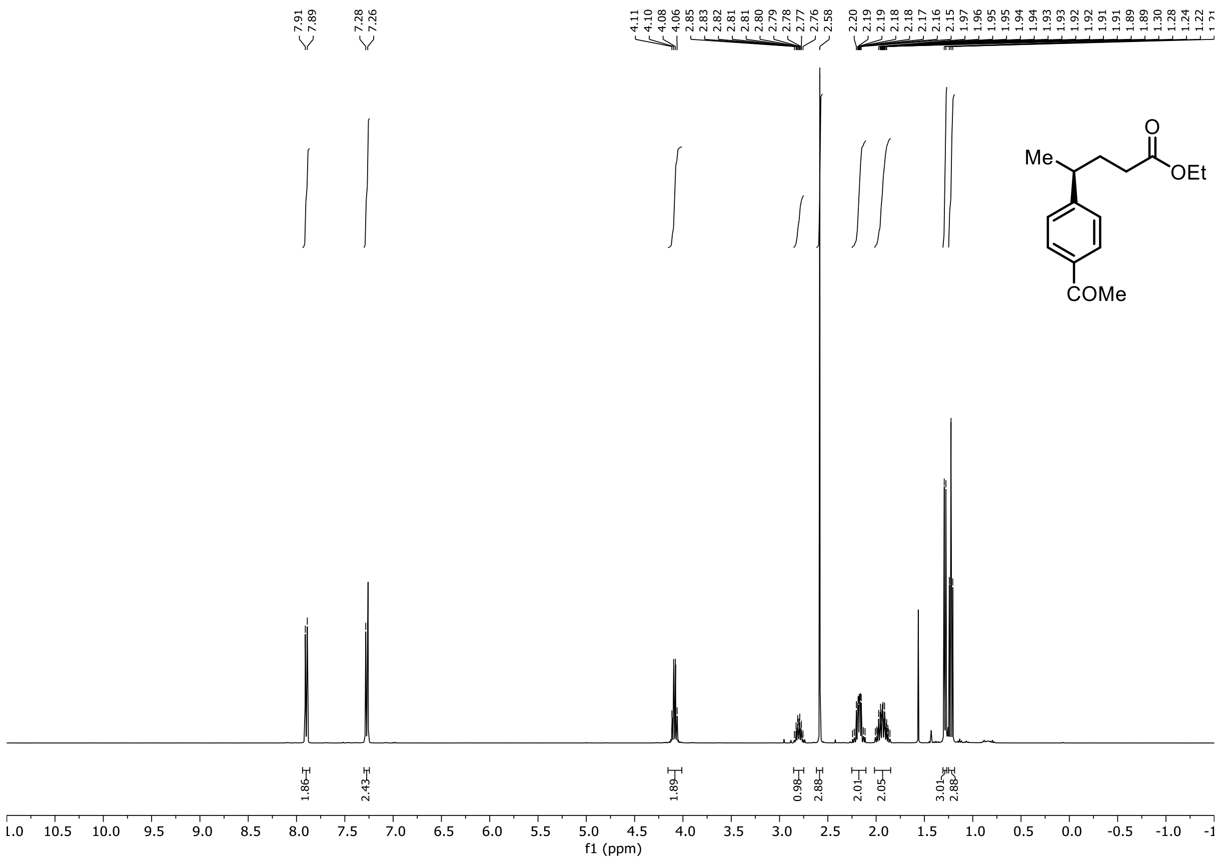

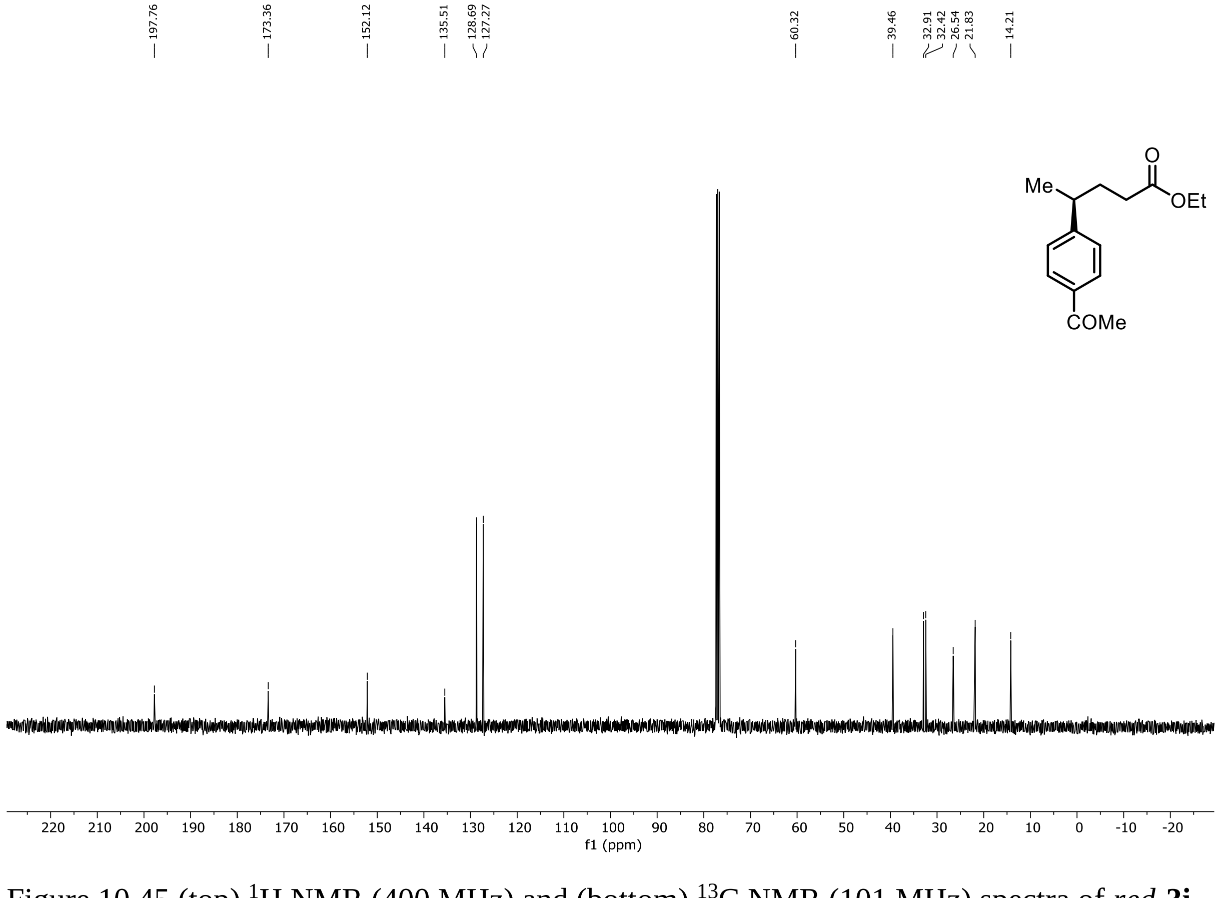

Figure 10.45 (top) ^{1}H NMR (400 MHz) and (bottom) ^{13}C NMR (101 MHz) spectra of *red*-**3i**.

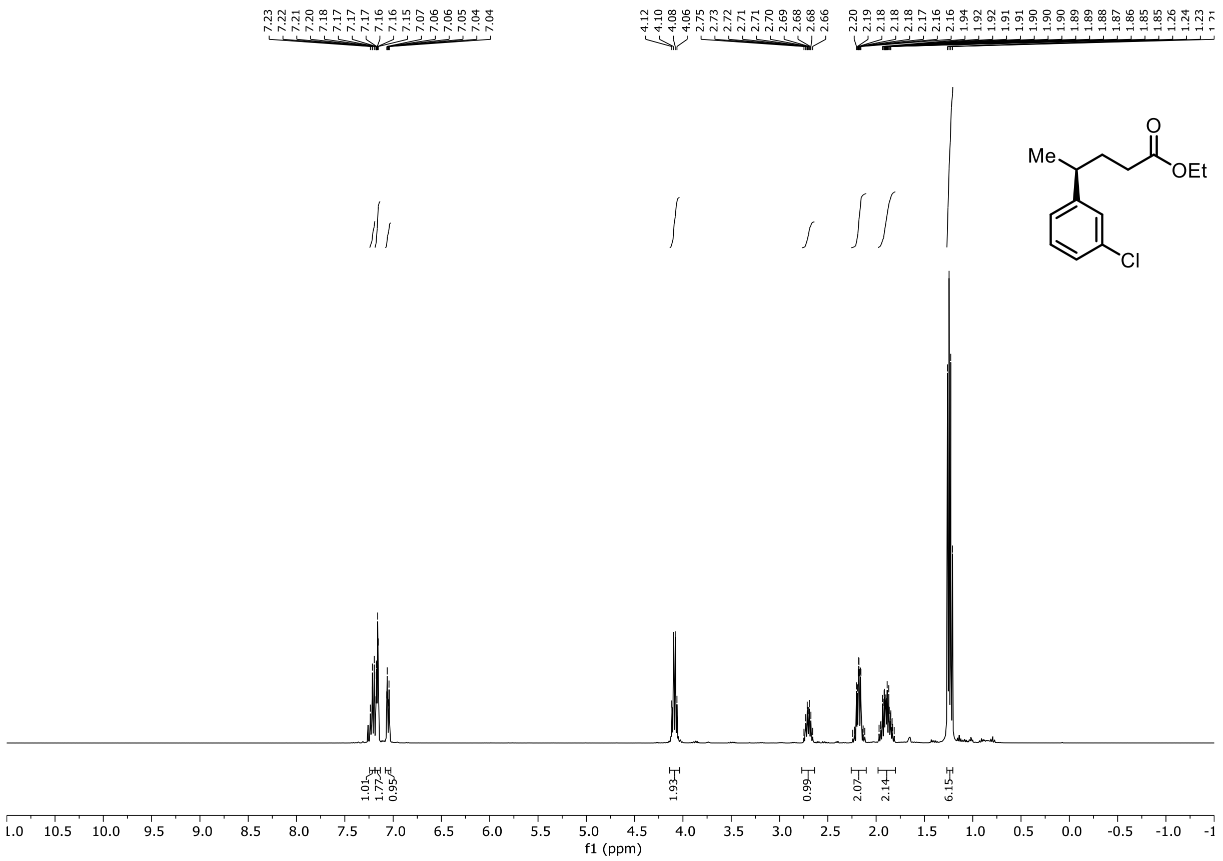

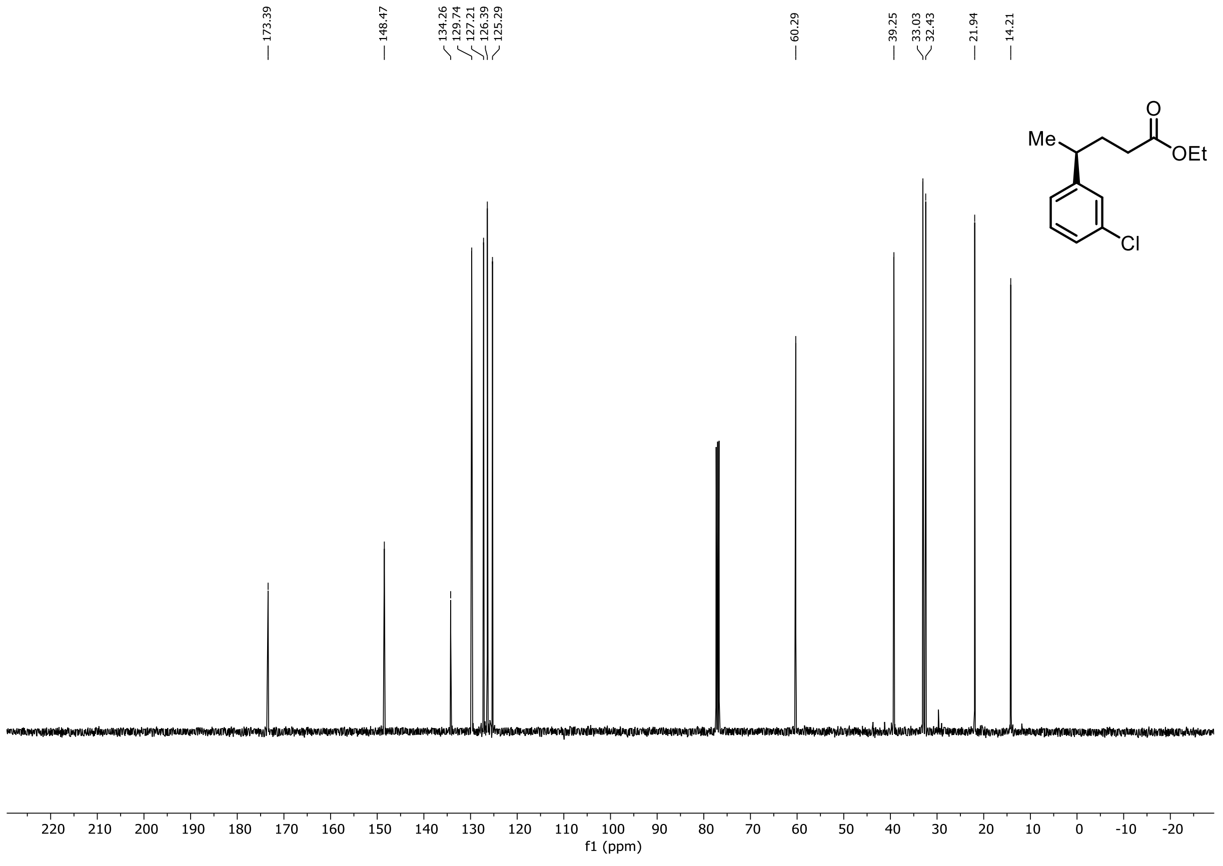

Figure 10.46 (top) ^{1}H NMR (400 MHz) and (bottom) ^{13}C NMR (101 MHz) spectra of *red*-**3j**.

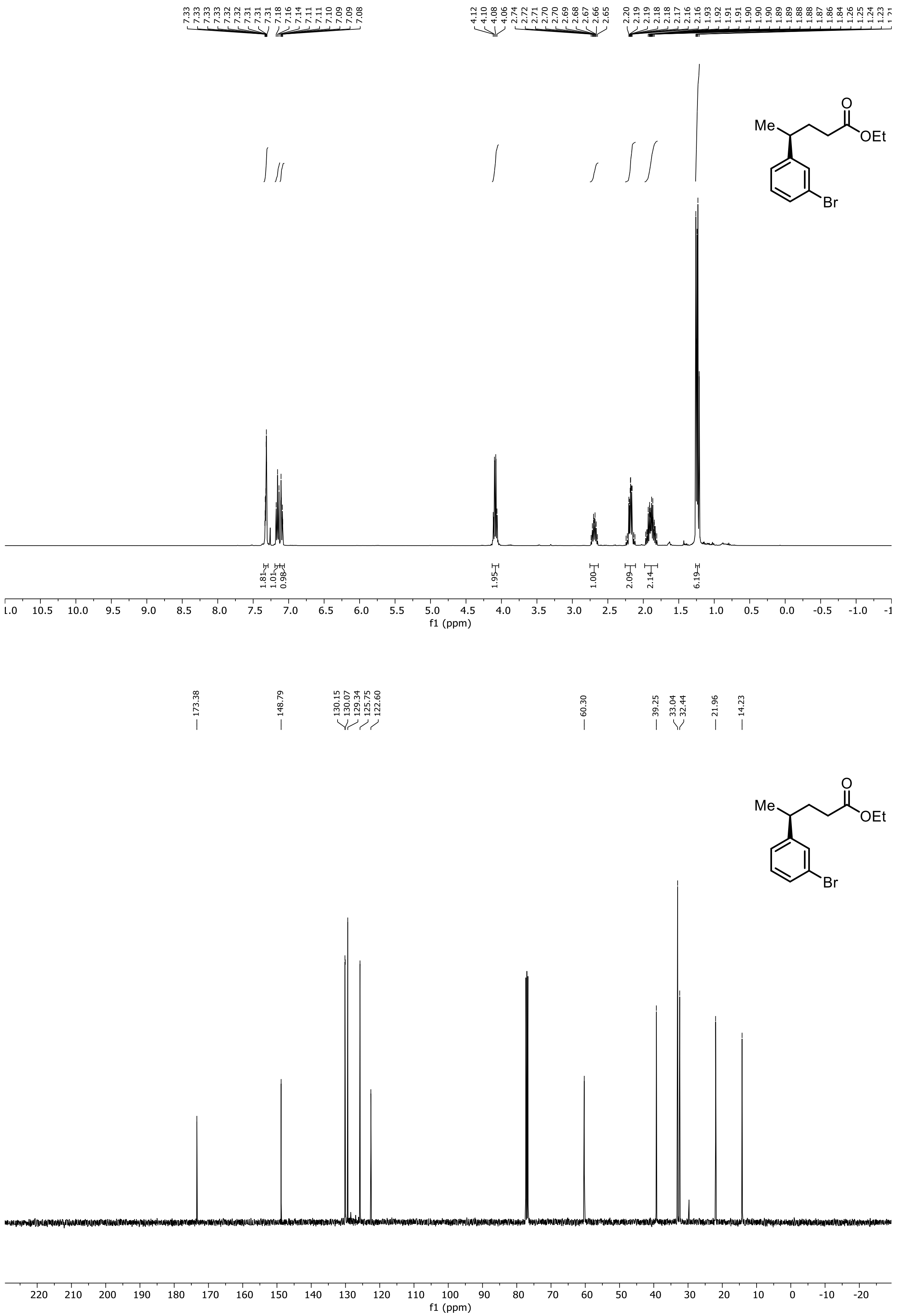

Figure 10.47 (top) ^{1}H NMR (400 MHz) and (bottom) ^{13}C NMR (101 MHz) spectra of *red*-**3k**.

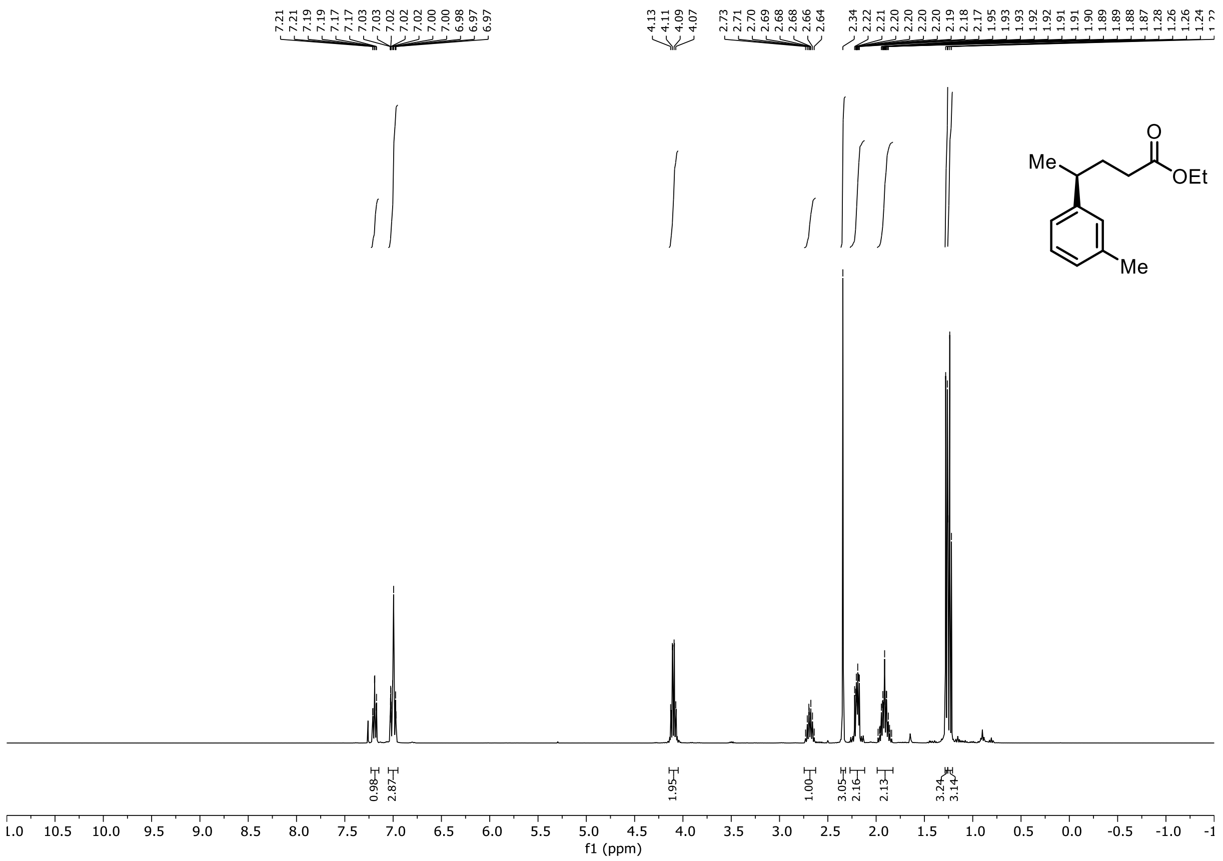

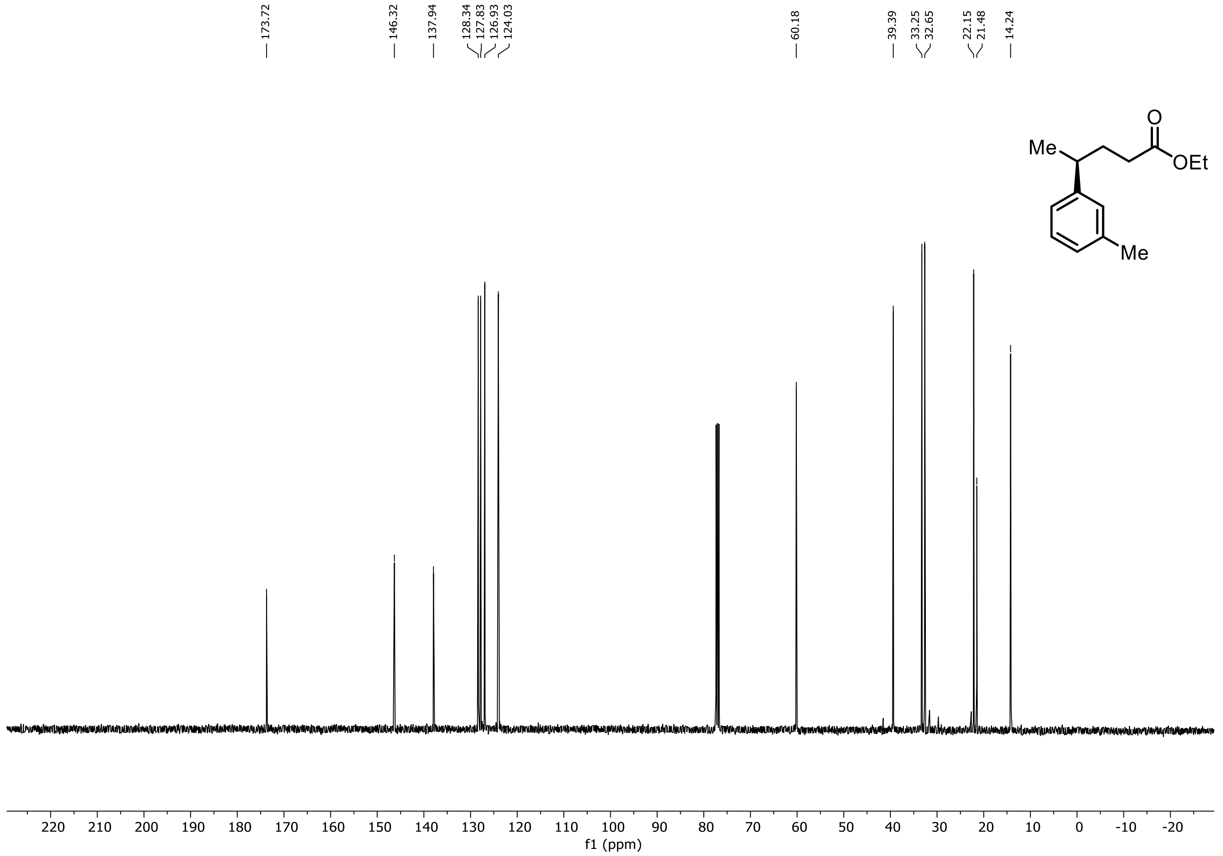

Figure 10.48 (top) ^{1}H NMR (400 MHz) and (bottom) ^{13}C NMR (101 MHz) spectra of *red*-**3l**.

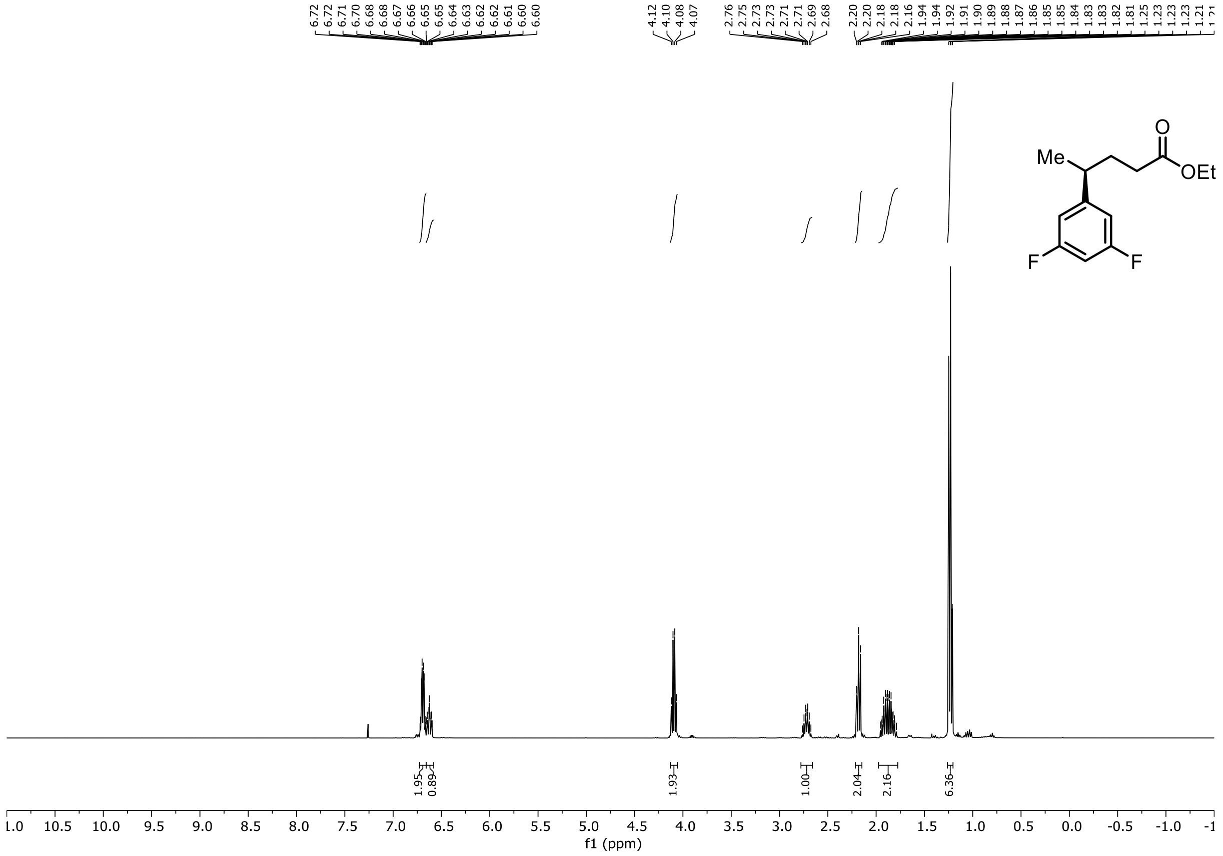

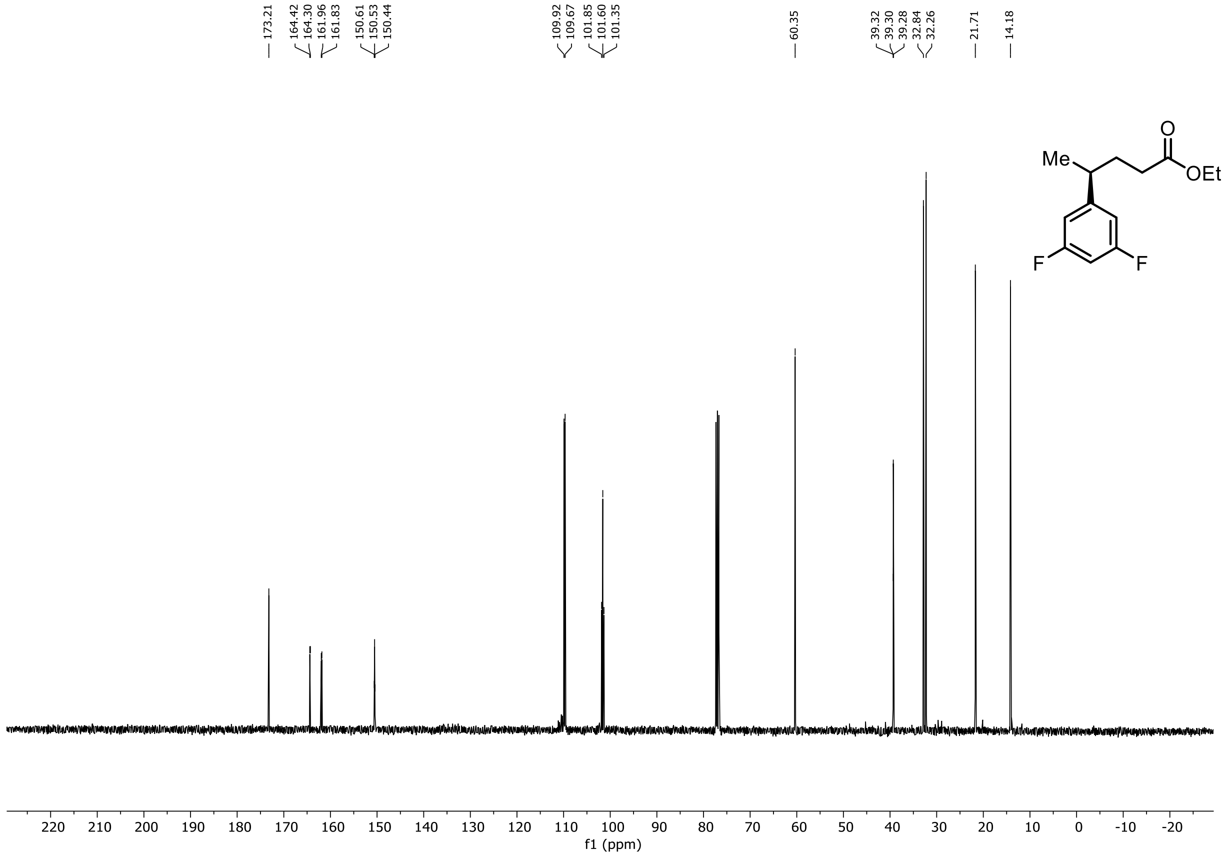

Figure 10.49 (top) ^{1}H NMR (400 MHz) and (bottom) ^{13}C NMR (101 MHz) spectra of *red*-**3m**.

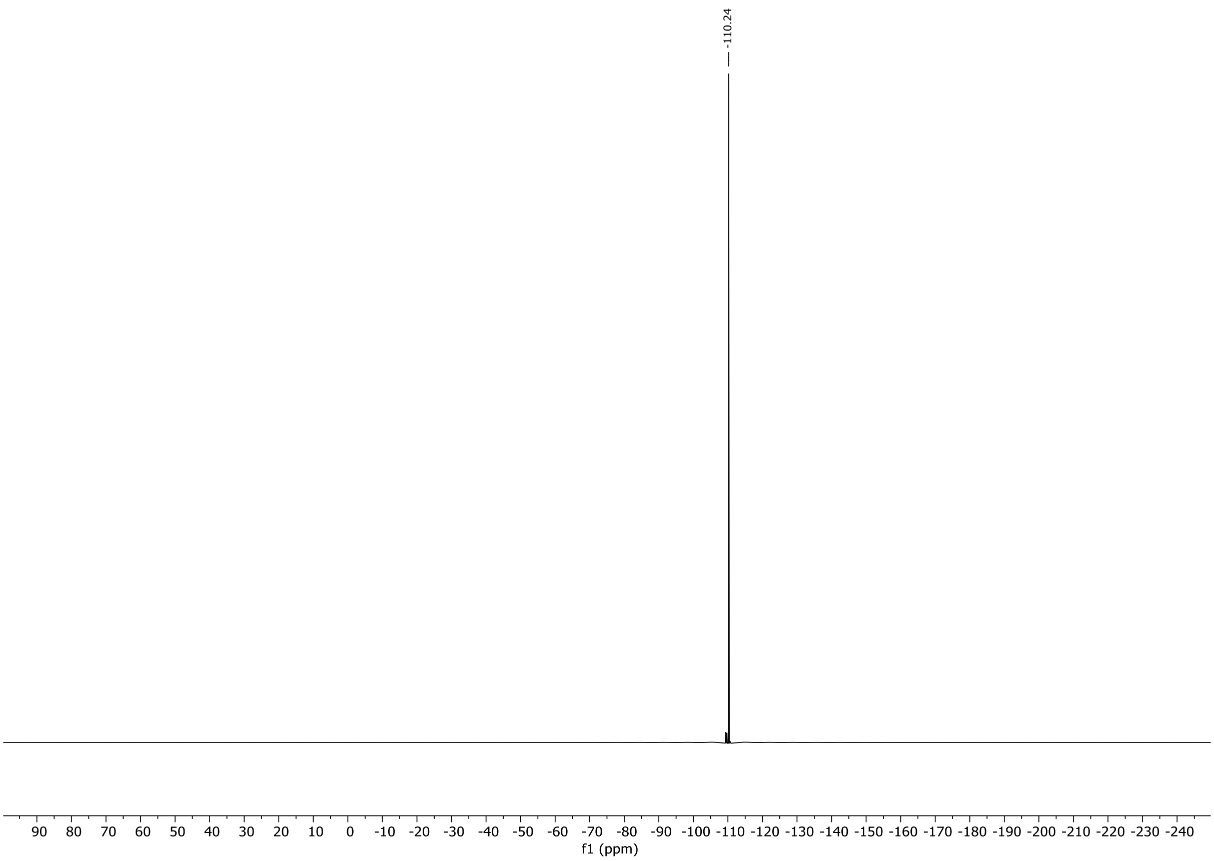

Figure 10.50 ^{19}F (13C)NMR (376 MHz) spectrum of *red*-**3m**.

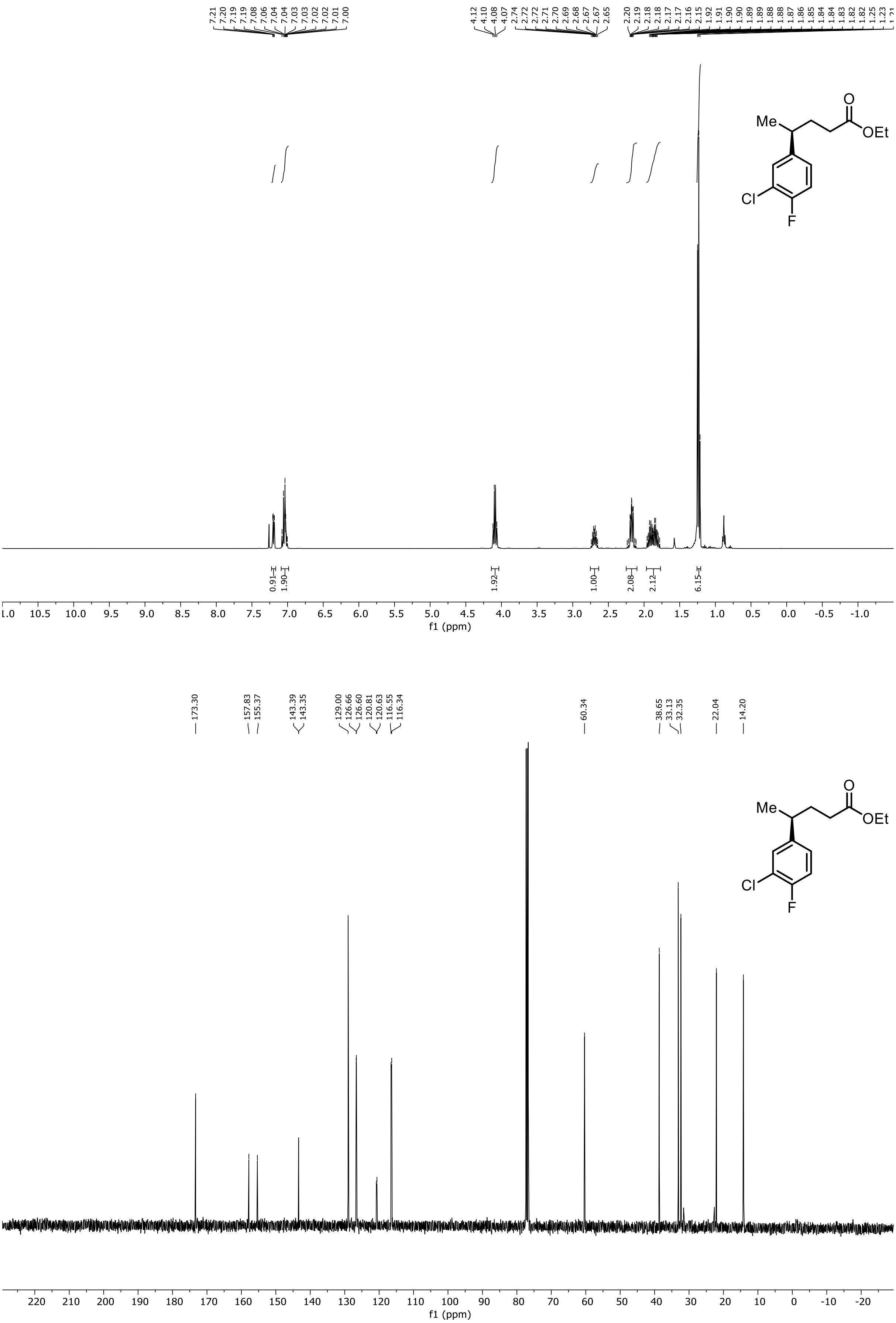

Figure 10.51 (top) ^{1}H NMR (400 MHz) and (bottom) ^{13}C NMR (101 MHz) spectra of *red*-**3n**.

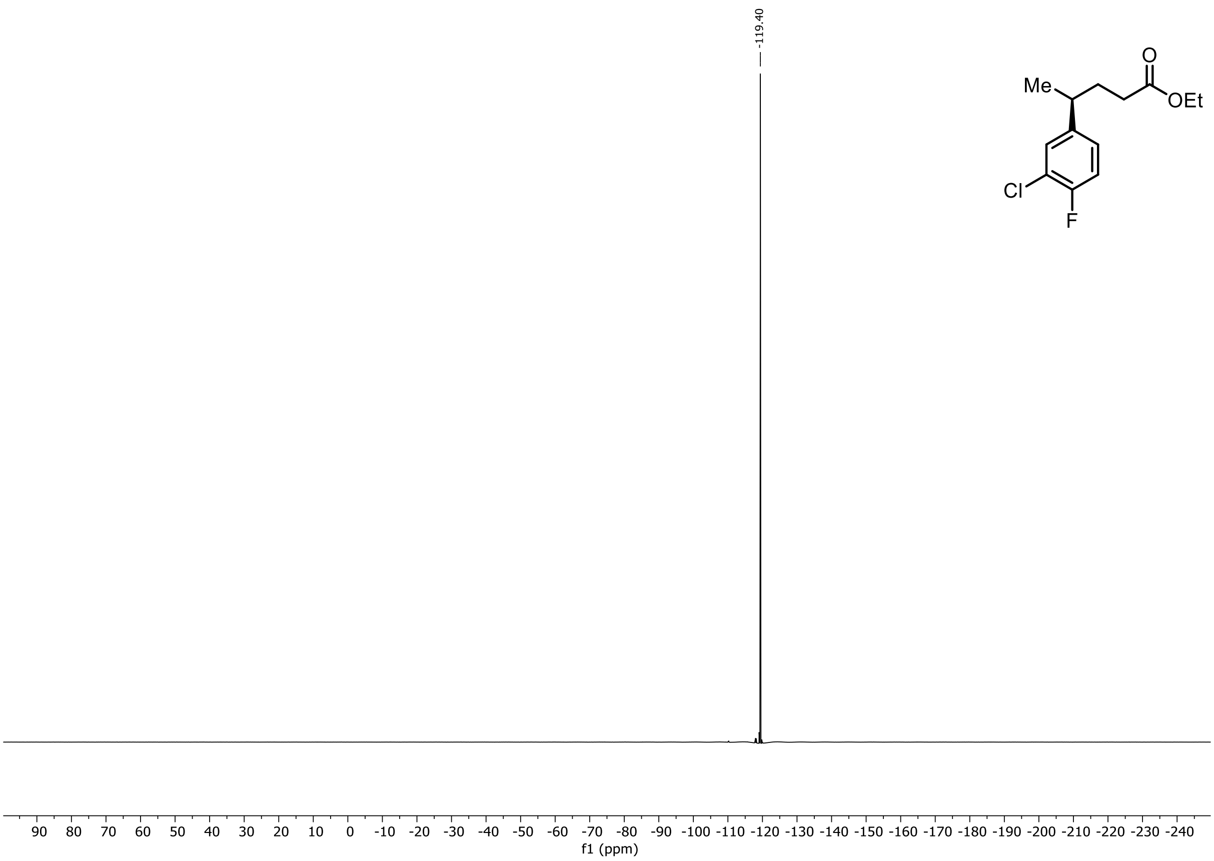

Figure 10.52 ^{19}F (13C)NMR (376 MHz) spectrum of *red*-**3n**.

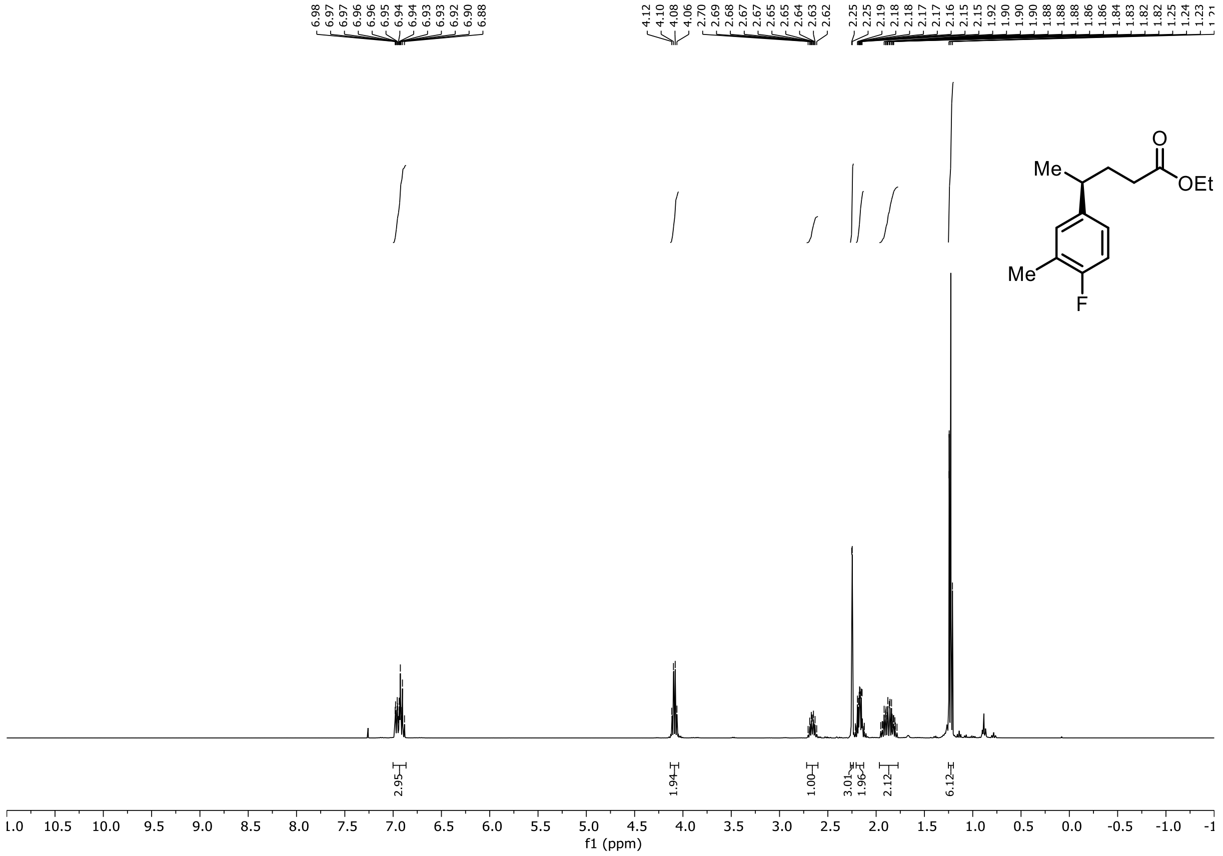

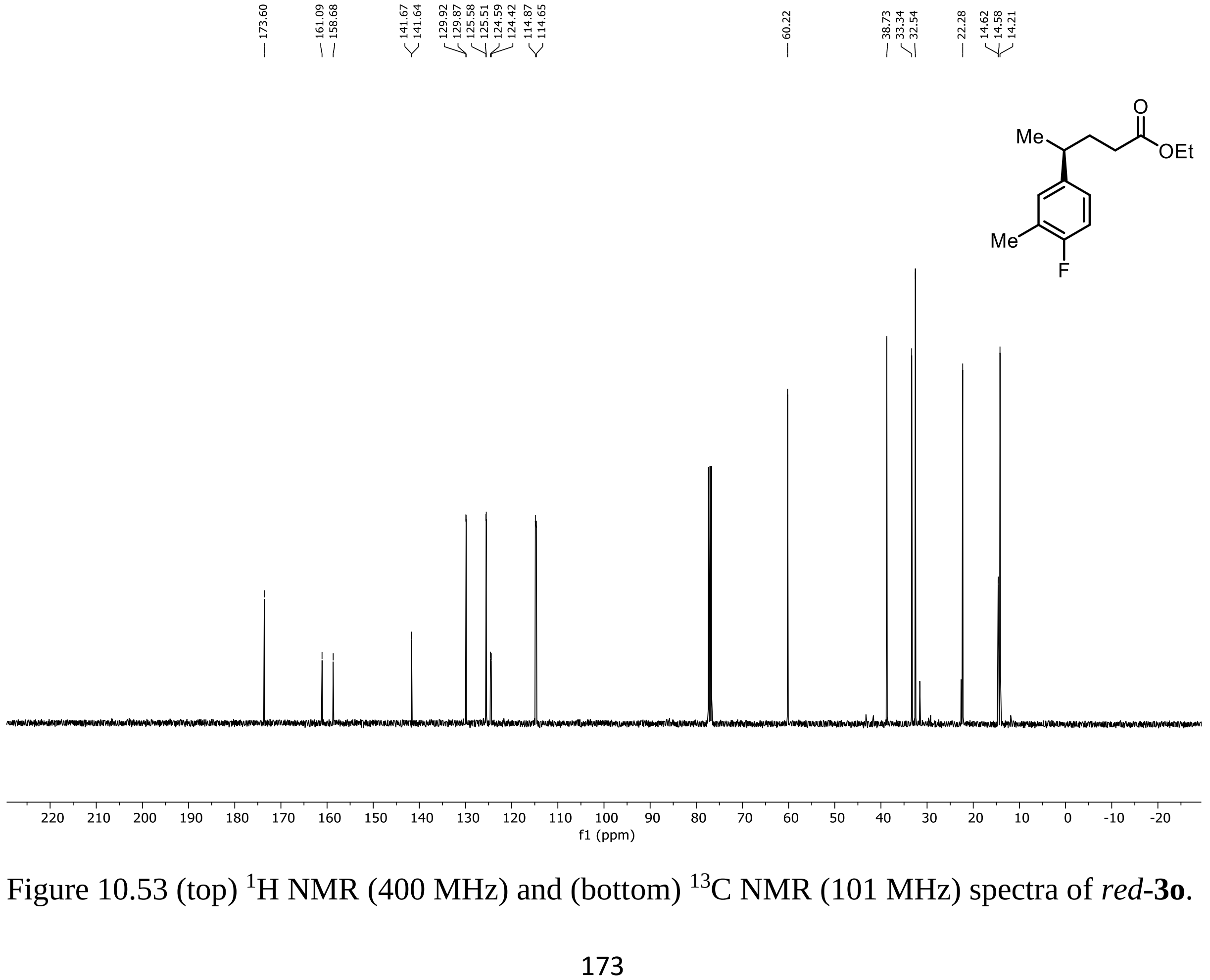

Figure 10.53 (top) ^{1}H NMR (400 MHz) and (bottom) ^{13}C NMR (101 MHz) spectra of *red*-**3o**.

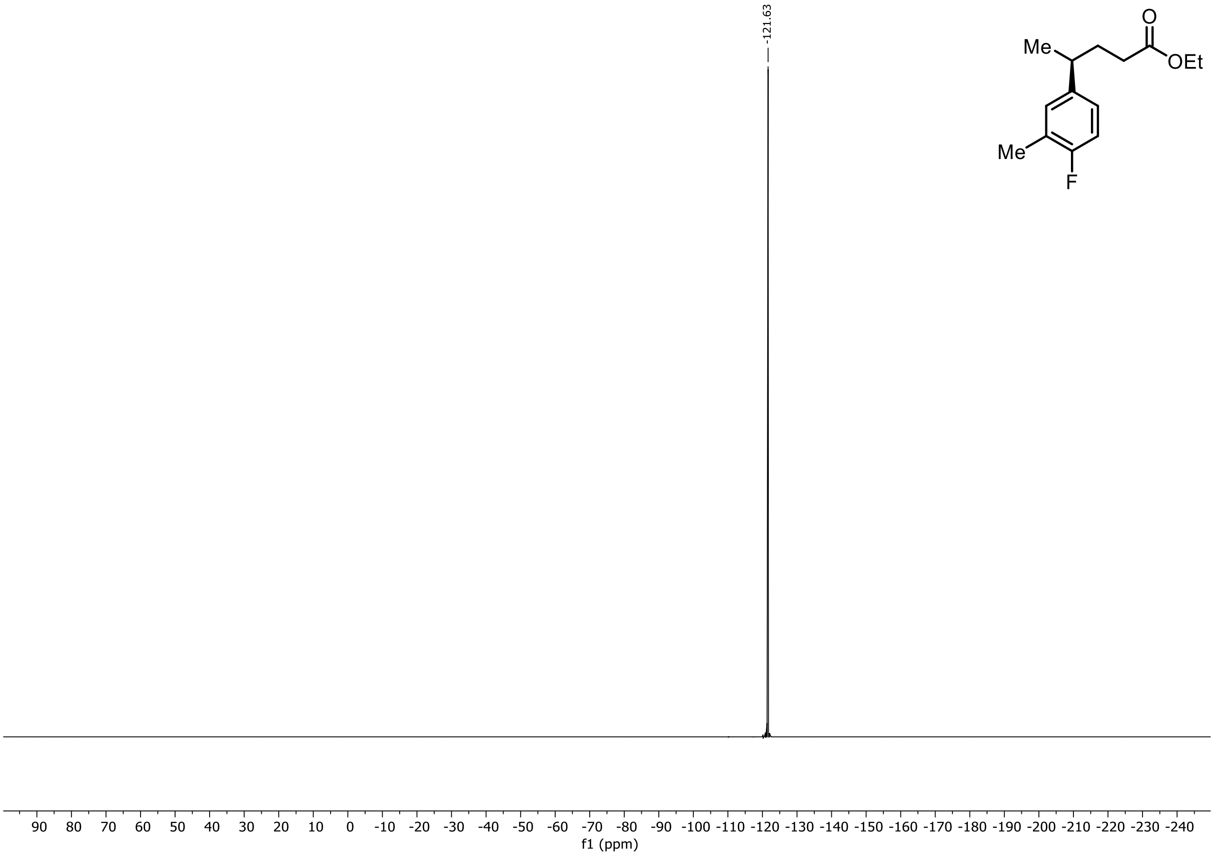

Figure 10.54 ^{19}F (13C)NMR (376 MHz) spectrum of *red*-**3o**.

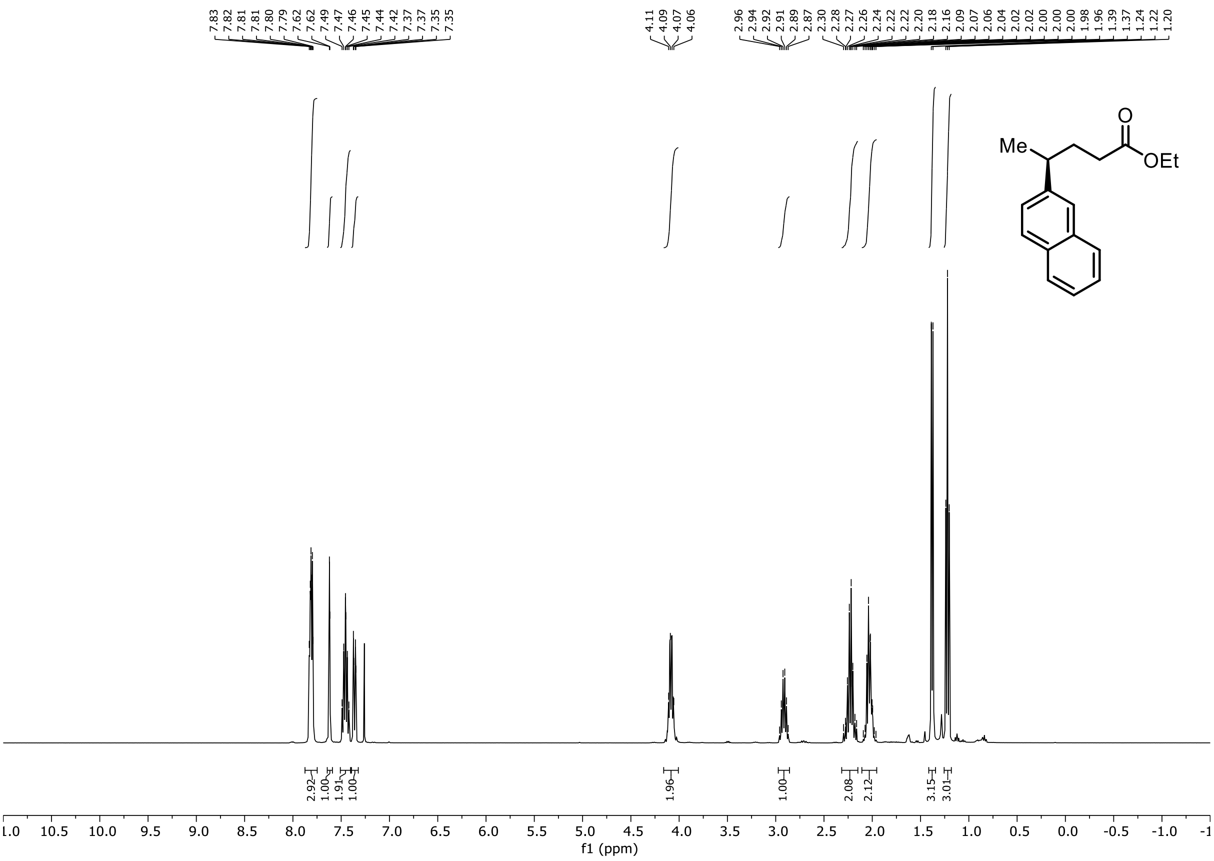

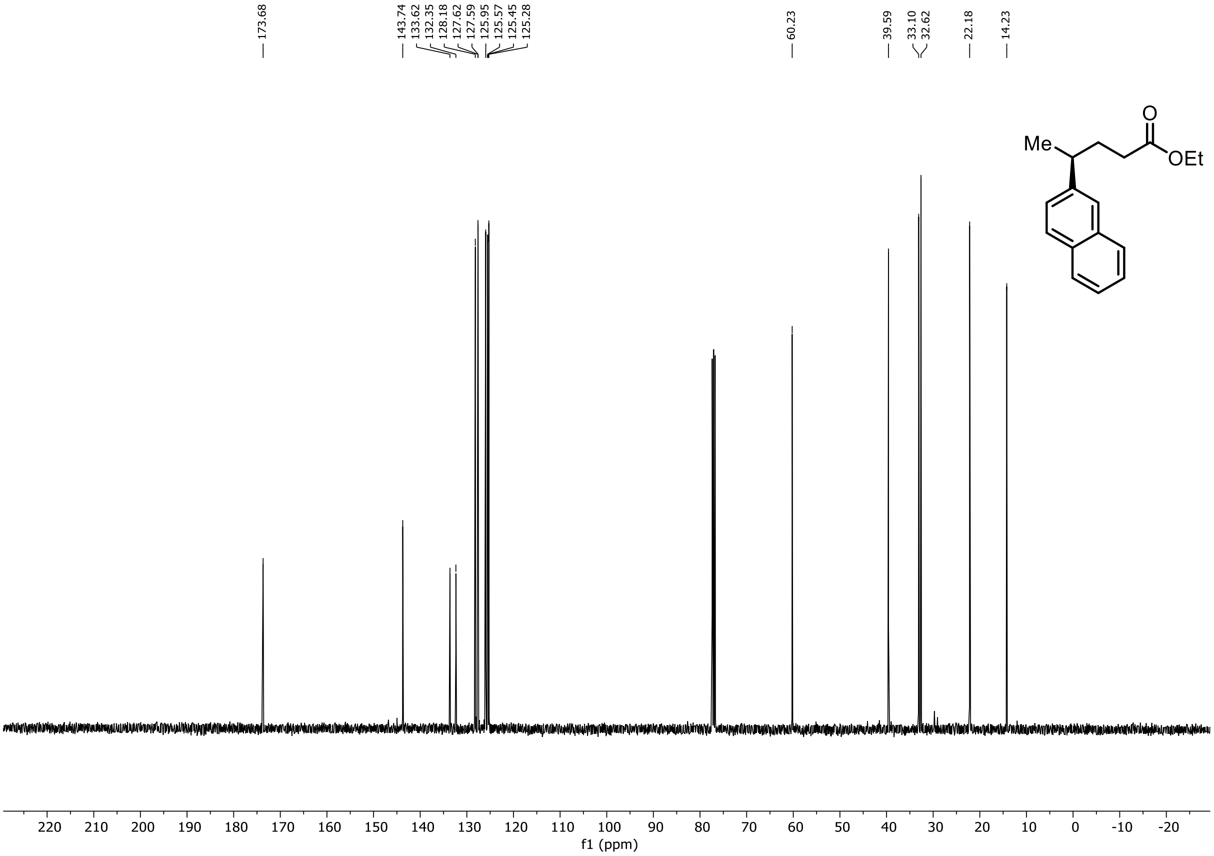

Figure 10.55 (top) ^{1}H NMR (400 MHz) and (bottom) ^{13}C NMR (101 MHz) spectra of *red*-**3p**.

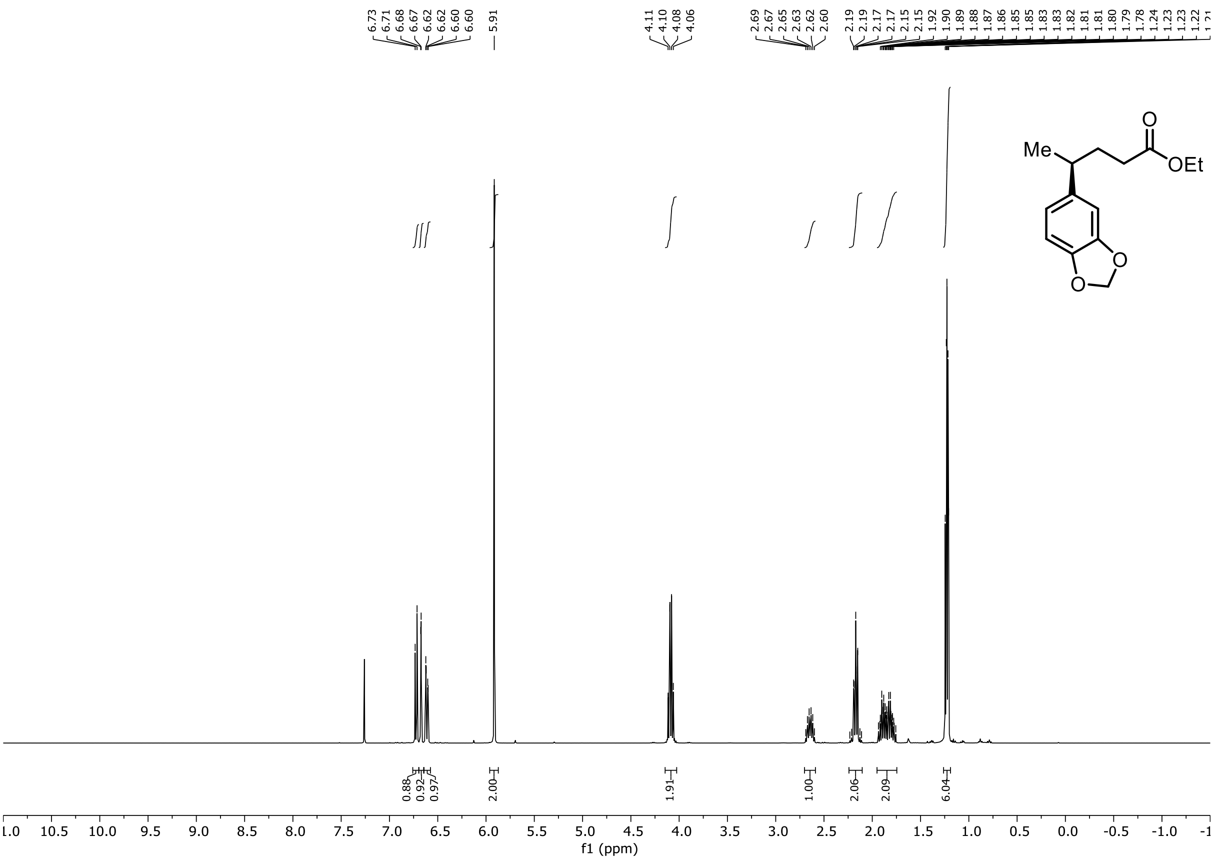

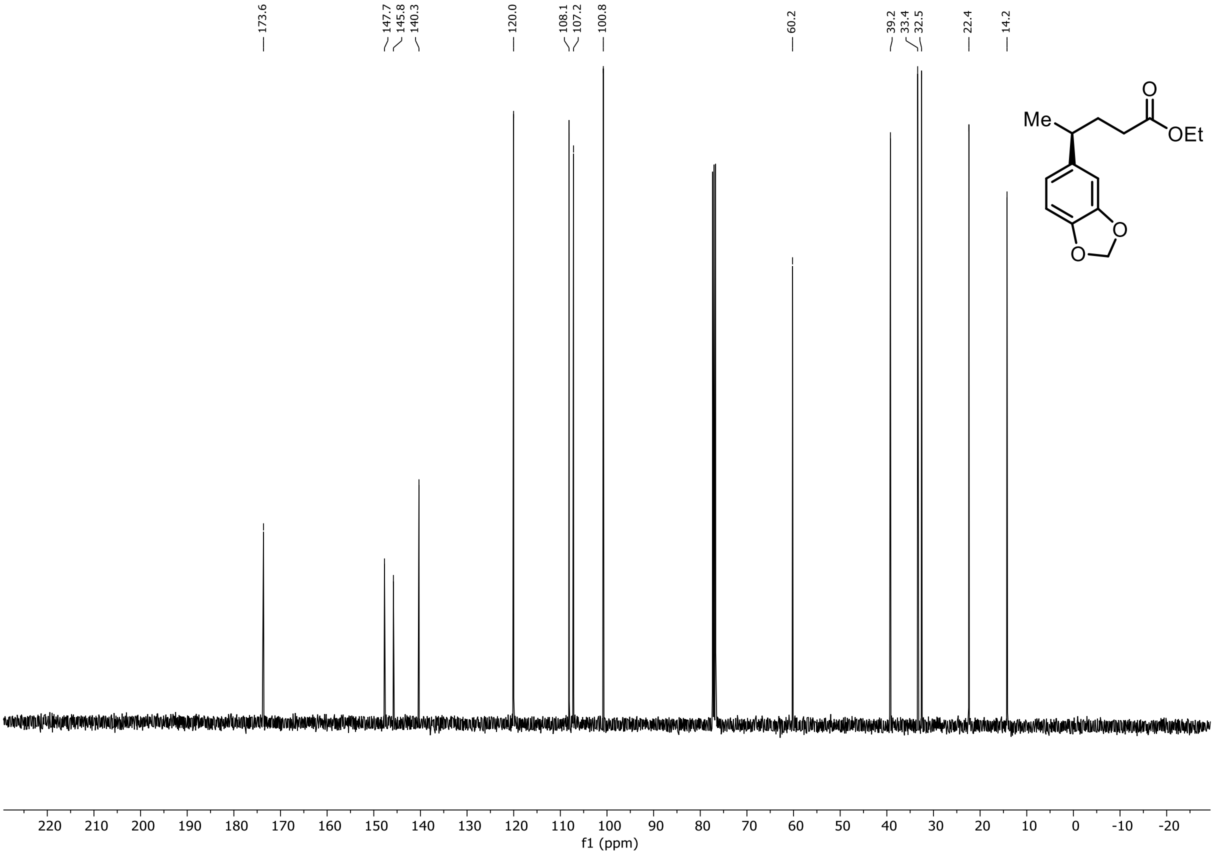

Figure 10.56 (top) ^{1}H NMR (400 MHz) and (bottom) ^{13}C NMR (101 MHz) spectra of *red*-**3q**.

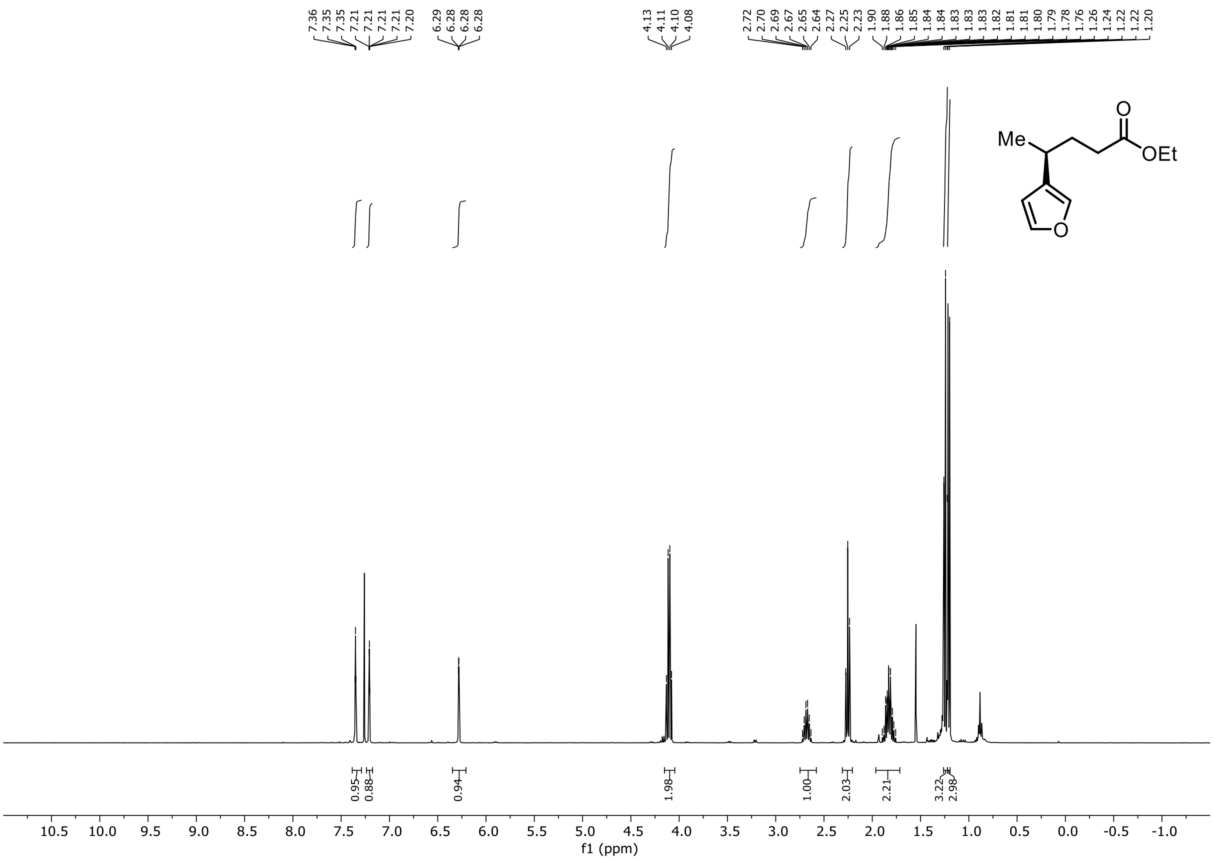

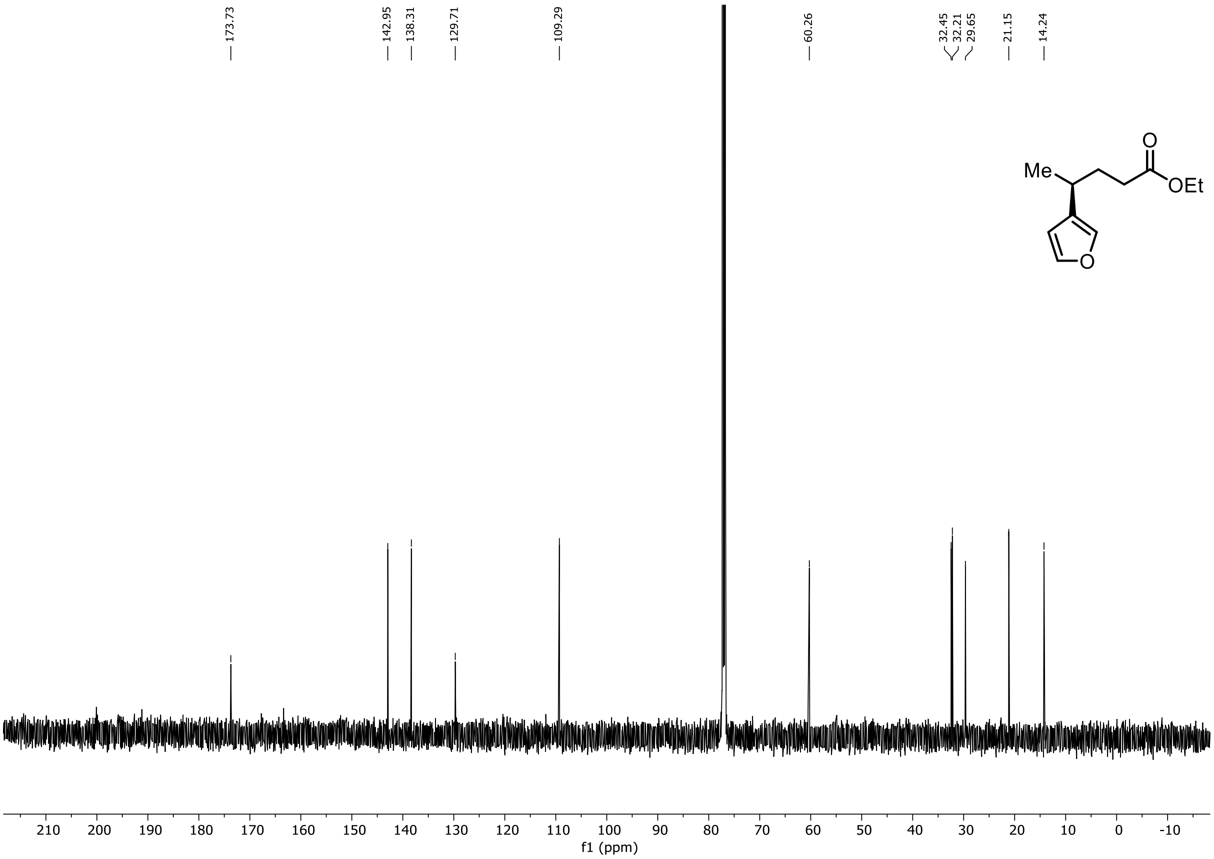

Figure 10.57 (top) ^{1}H NMR (400 MHz) and (bottom) ^{13}C NMR (101 MHz) spectra of *red*-**3r**.

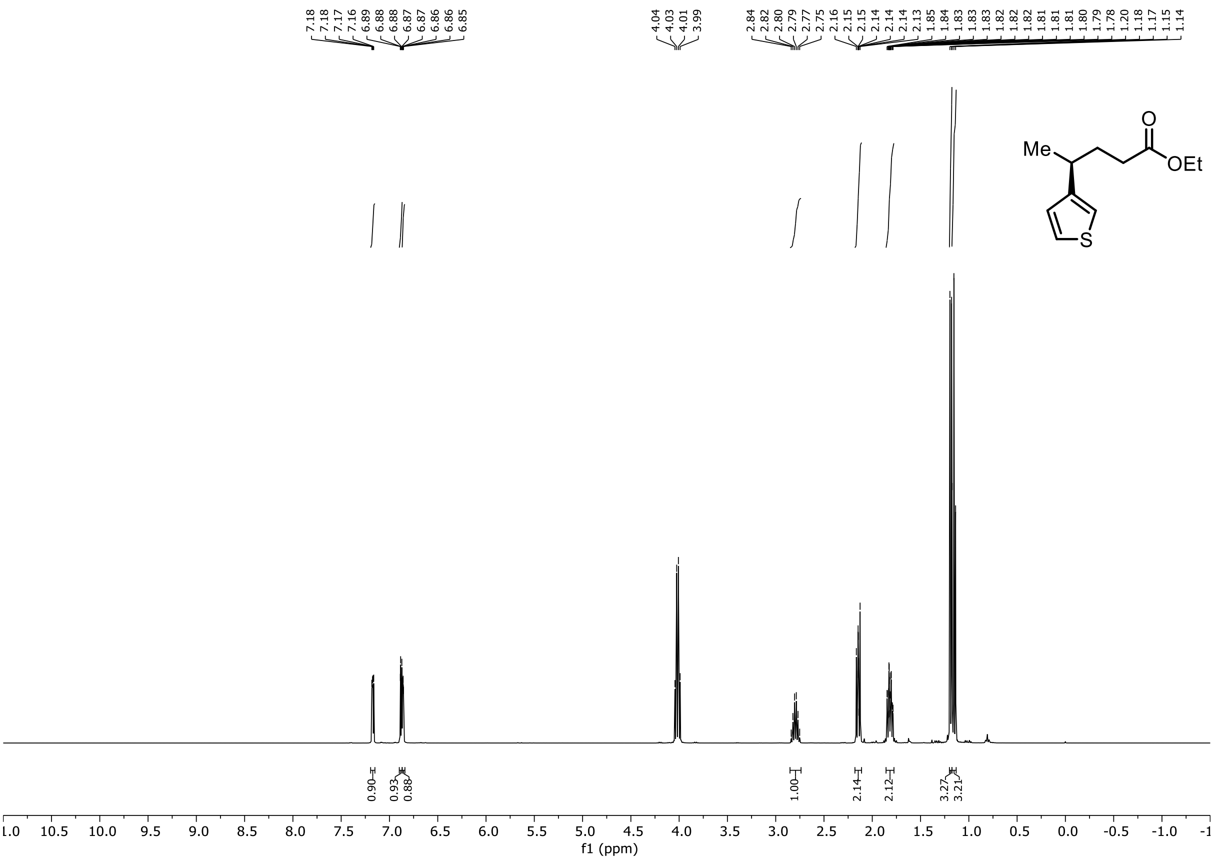

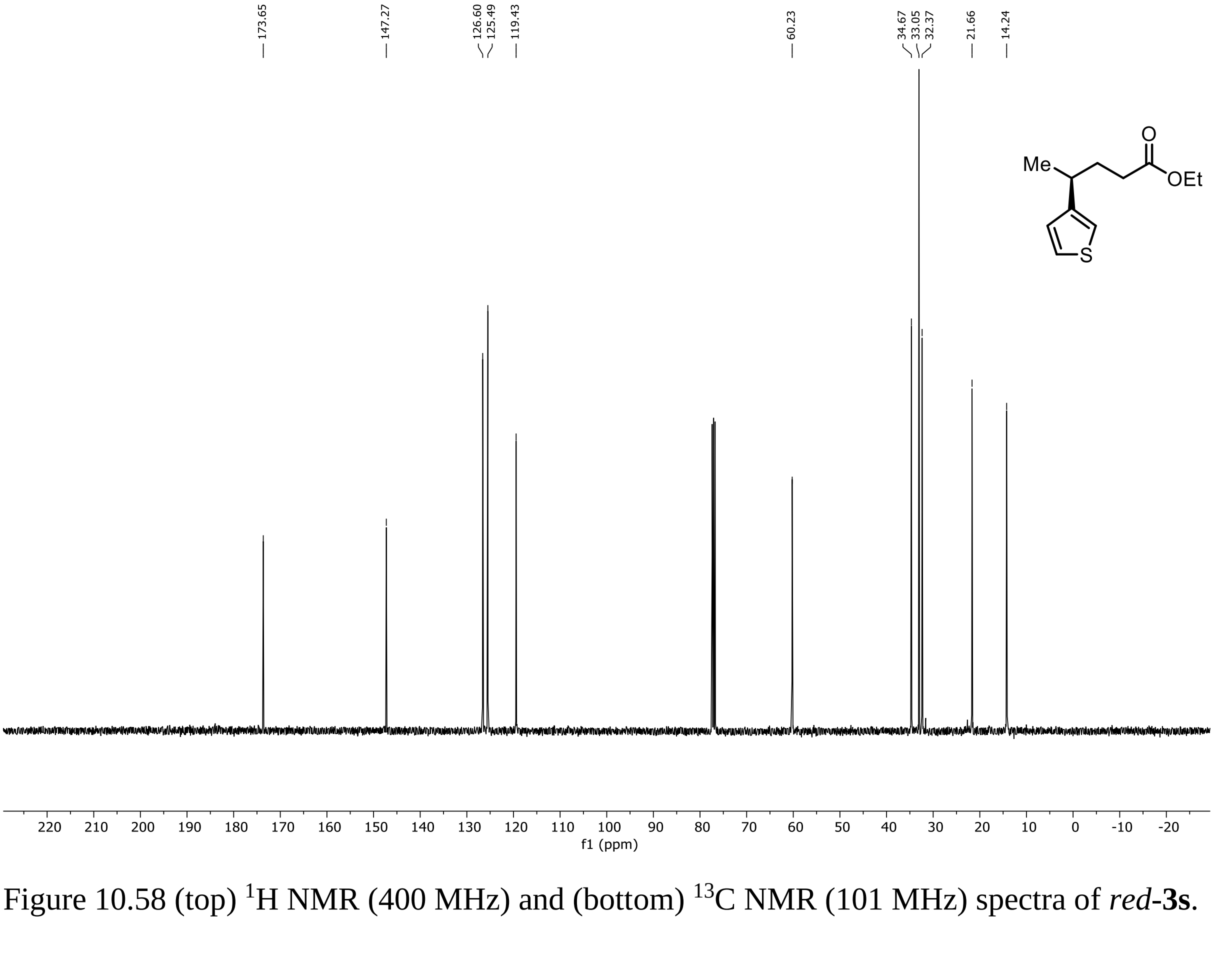

Figure 10.58 (top) ^{1}H NMR (400 MHz) and (bottom) ^{13}C NMR (101 MHz) spectra of *red*-**3s**.

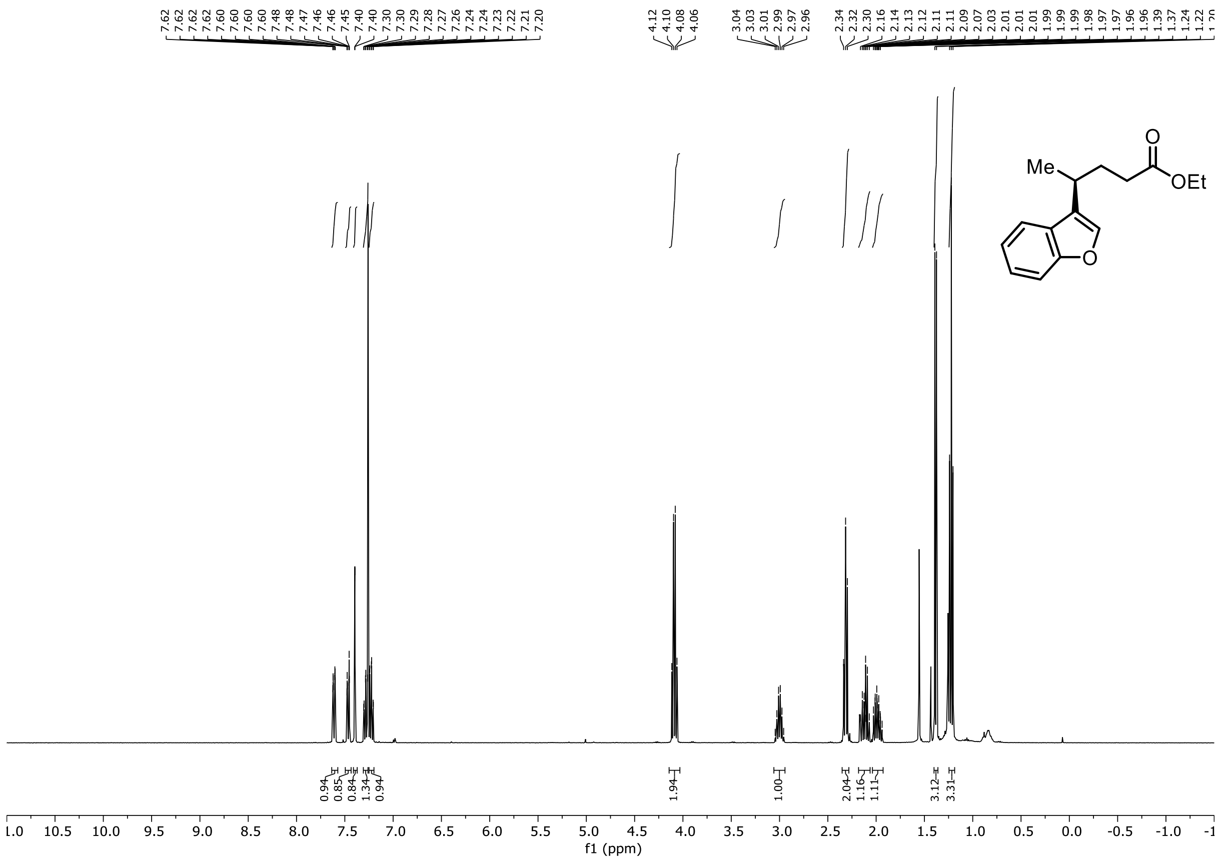

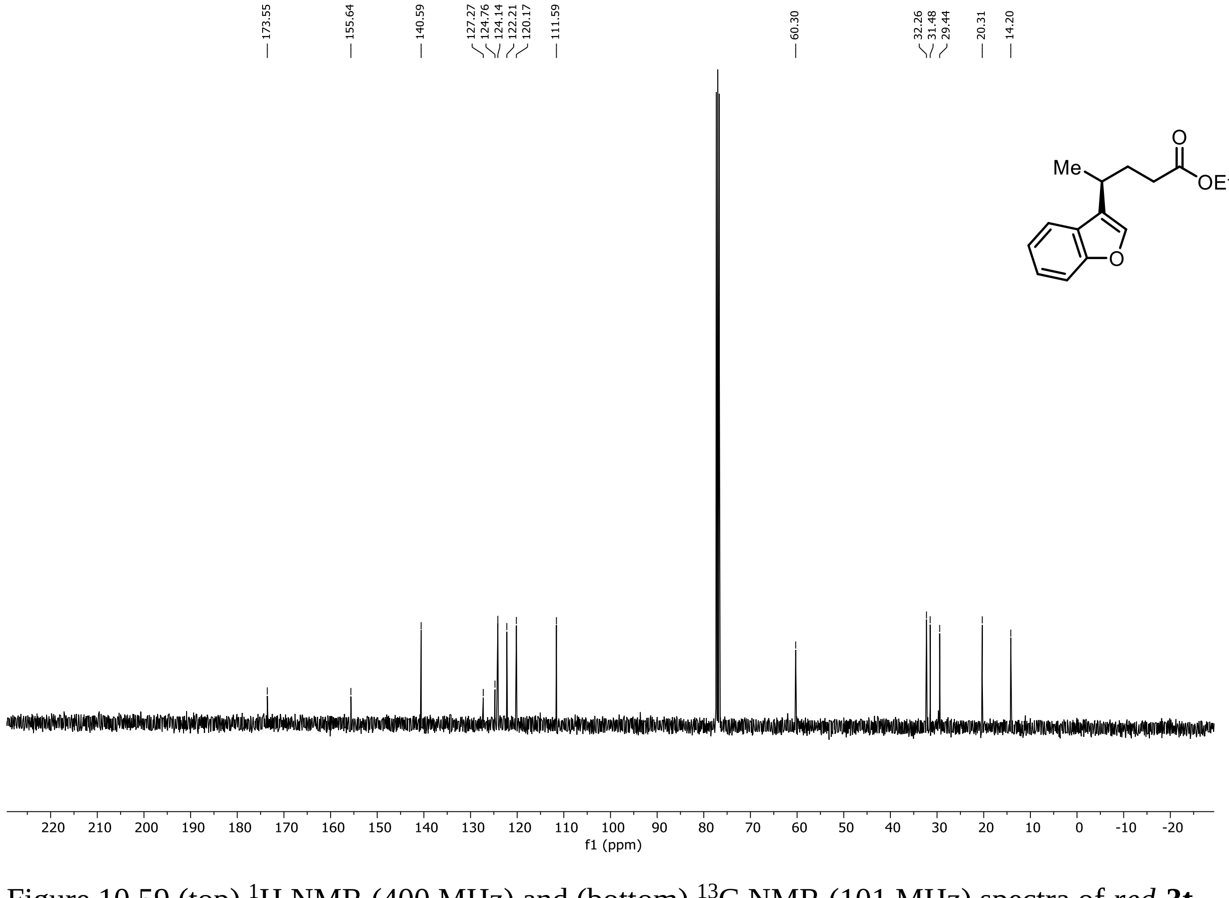

Figure 10.59 (top) ^{1}H NMR (400 MHz) and (bottom) ^{13}C NMR (101 MHz) spectra of *red*-**3t**.

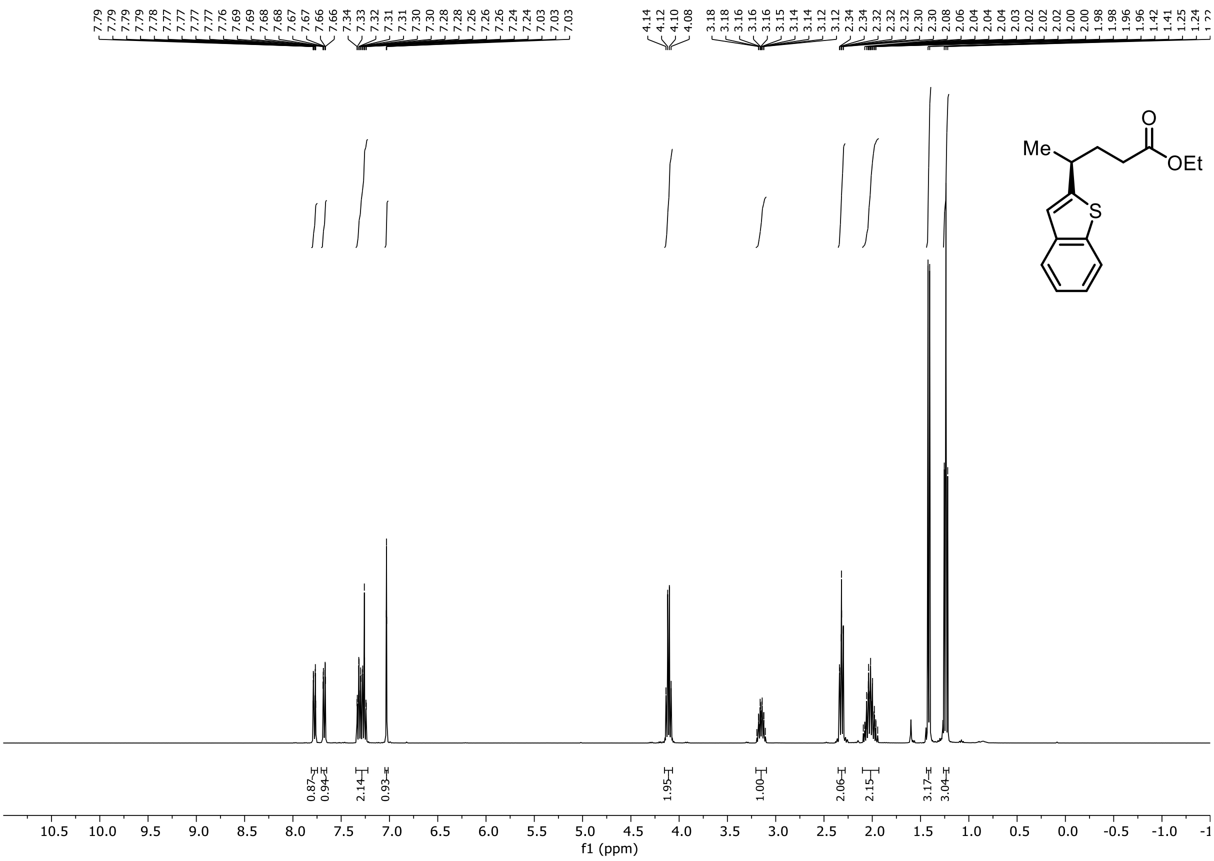

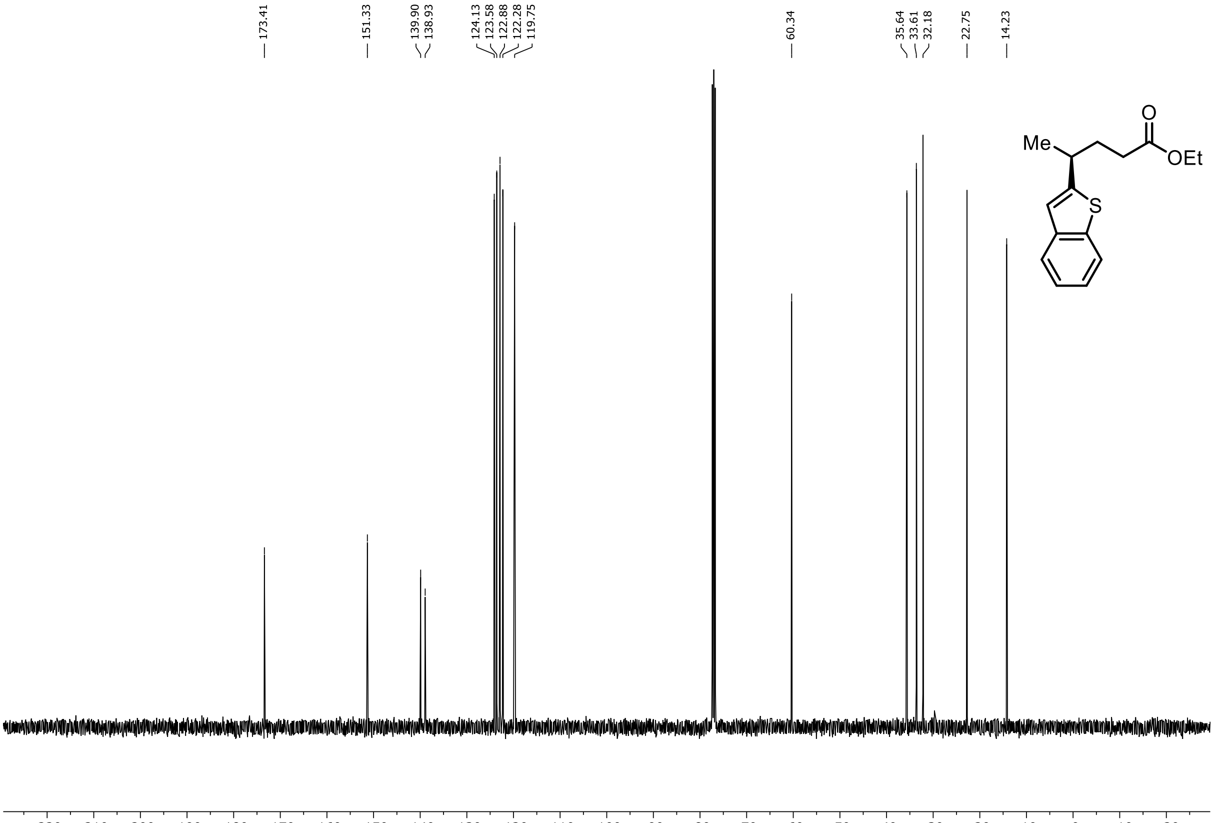

Figure 10.60 (top) ^{1}H NMR (400 MHz) and (bottom) ^{13}C NMR (101 MHz) spectra of *red*-**3u**.

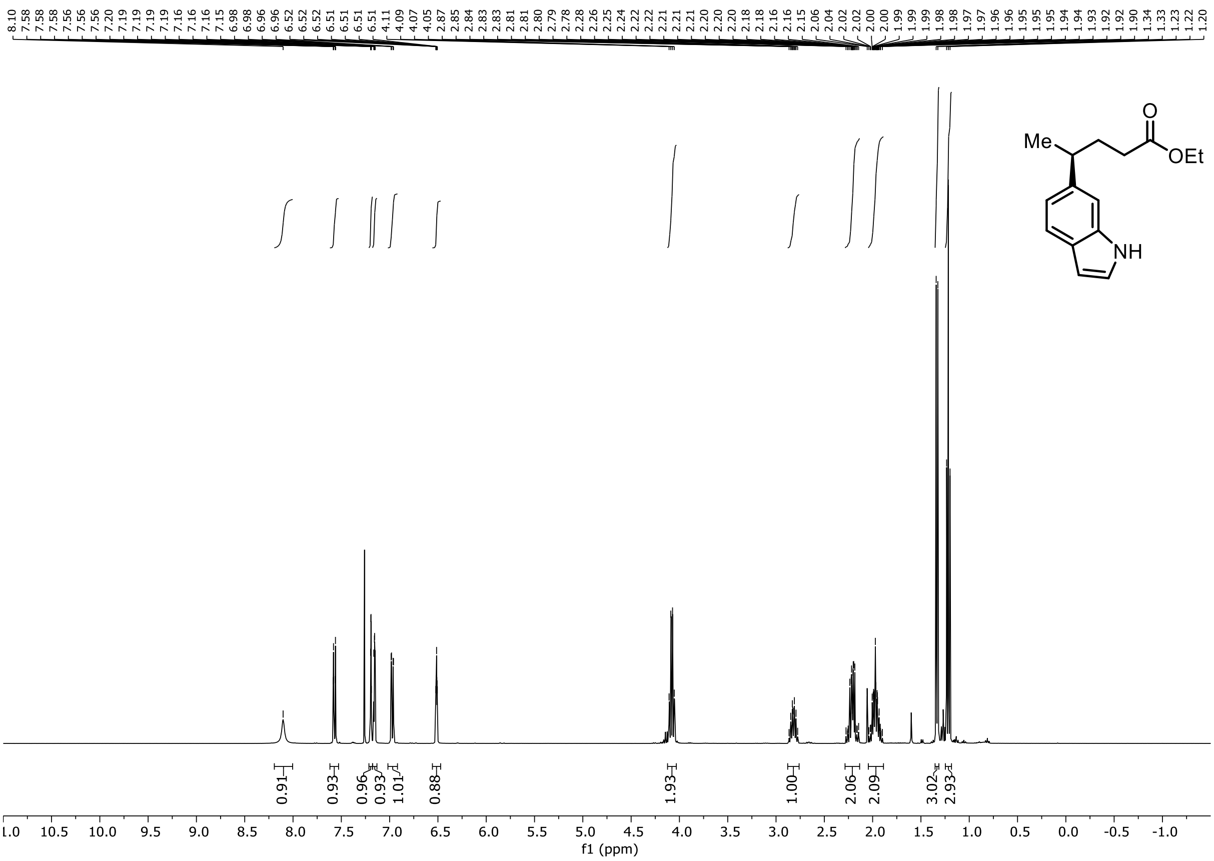

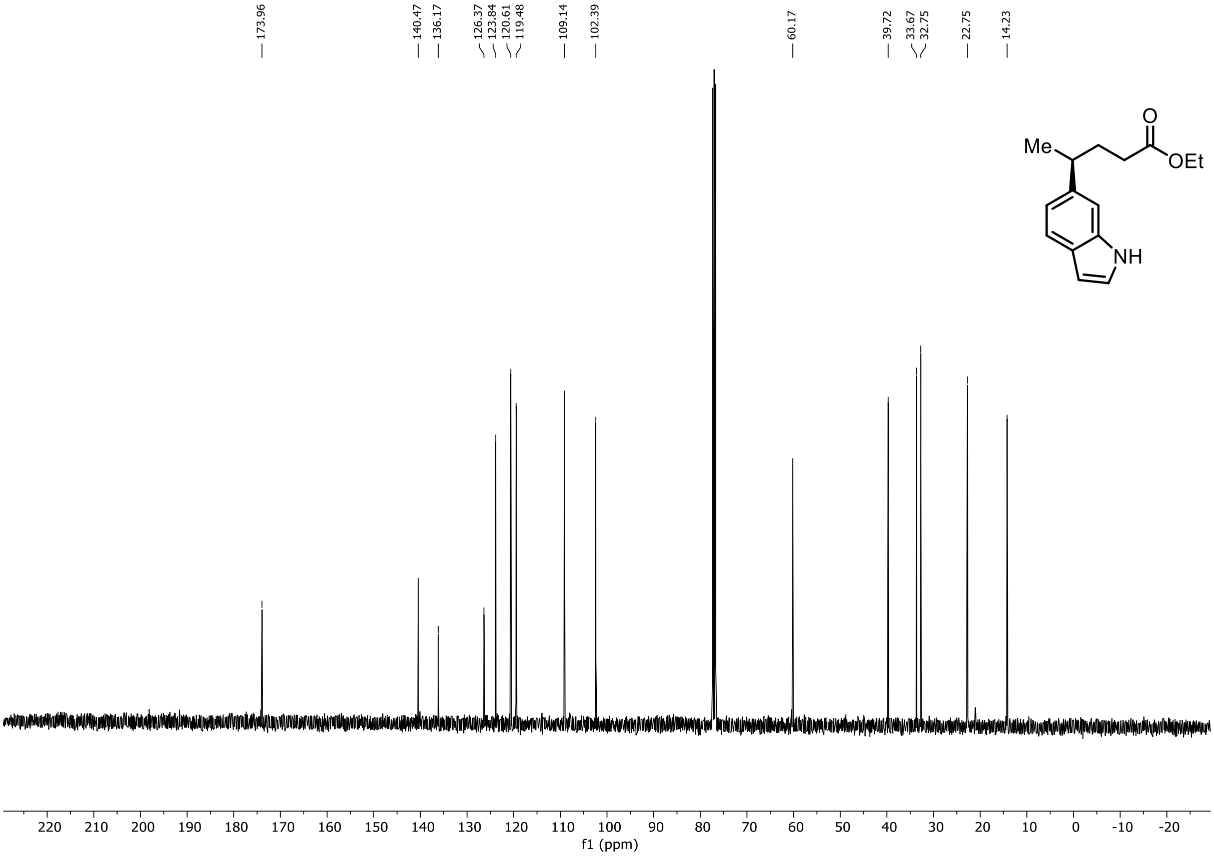

Figure 10.61 (top) ^{1}H NMR (400 MHz) and (bottom) ^{13}C NMR (101 MHz) spectra of *red*-**3v**.

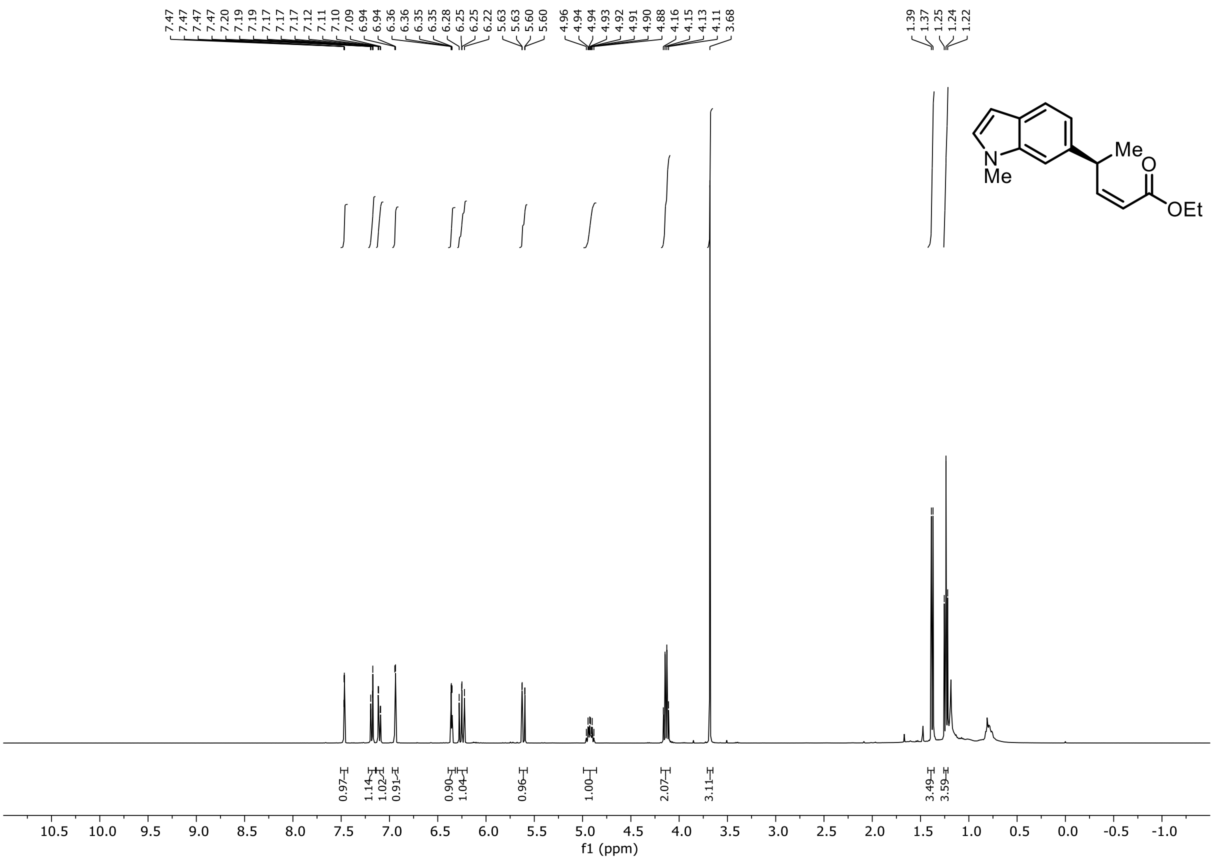

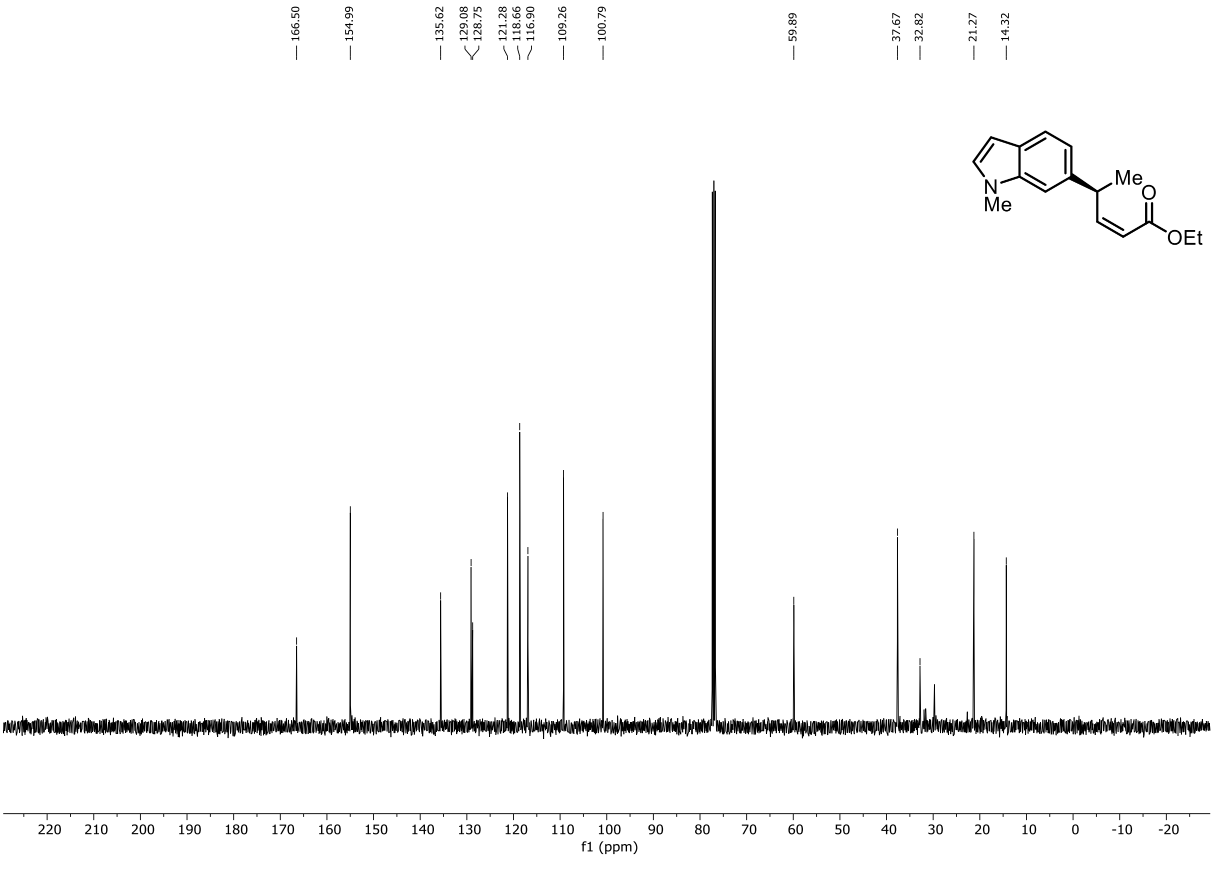

Figure 10.62 (top) ^{1}H NMR (400 MHz) and (bottom) ^{13}C NMR (101 MHz) spectra of *Z*-**3w**.

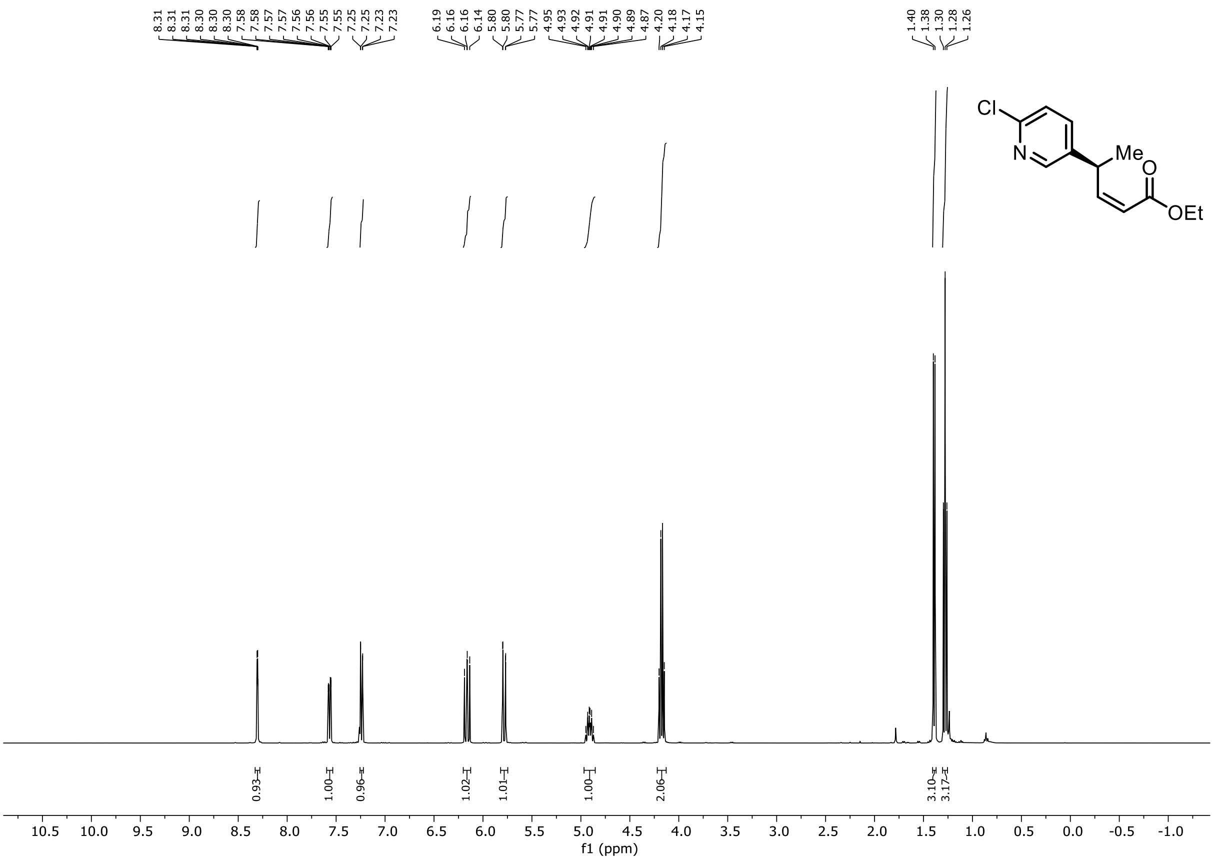

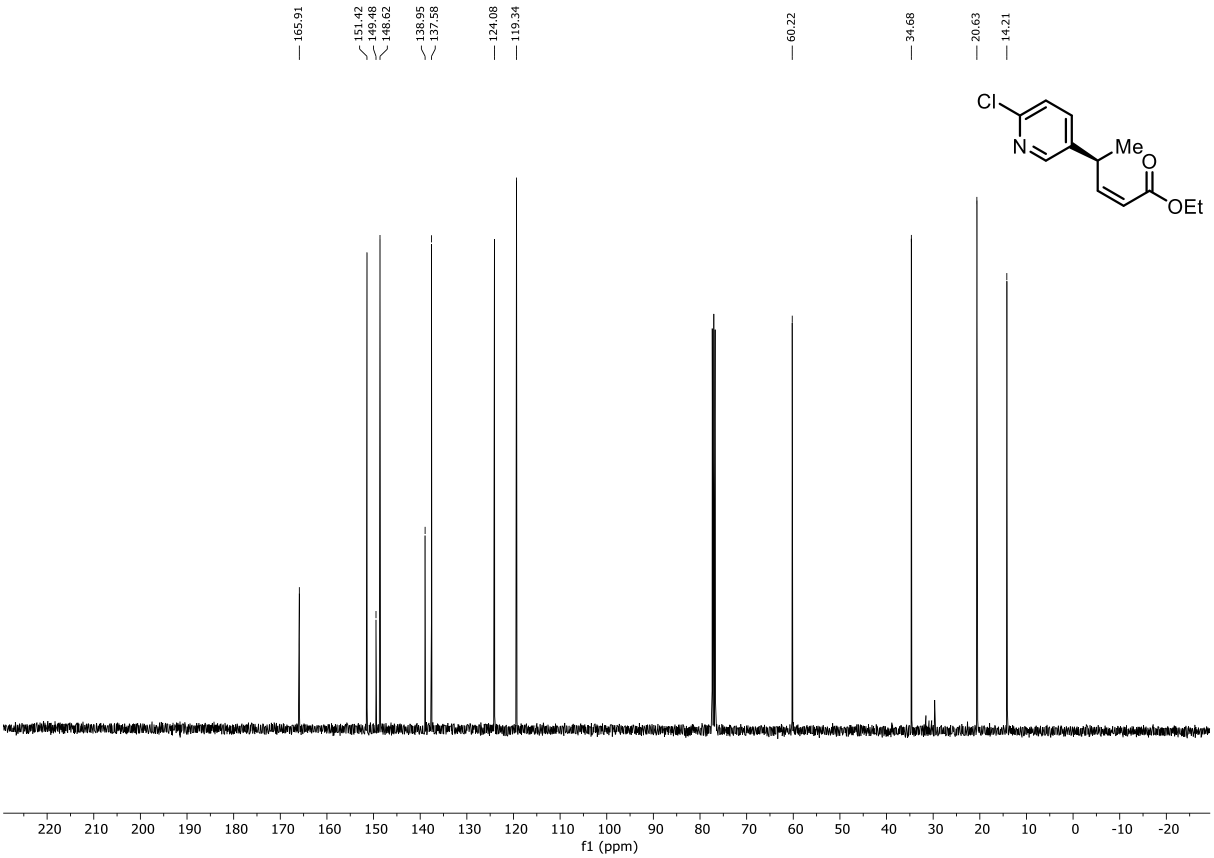

Figure 10.63 (top) ^{1}H NMR (400 MHz) and (bottom) ^{13}C NMR (101 MHz) spectra of *Z*-**3x**.

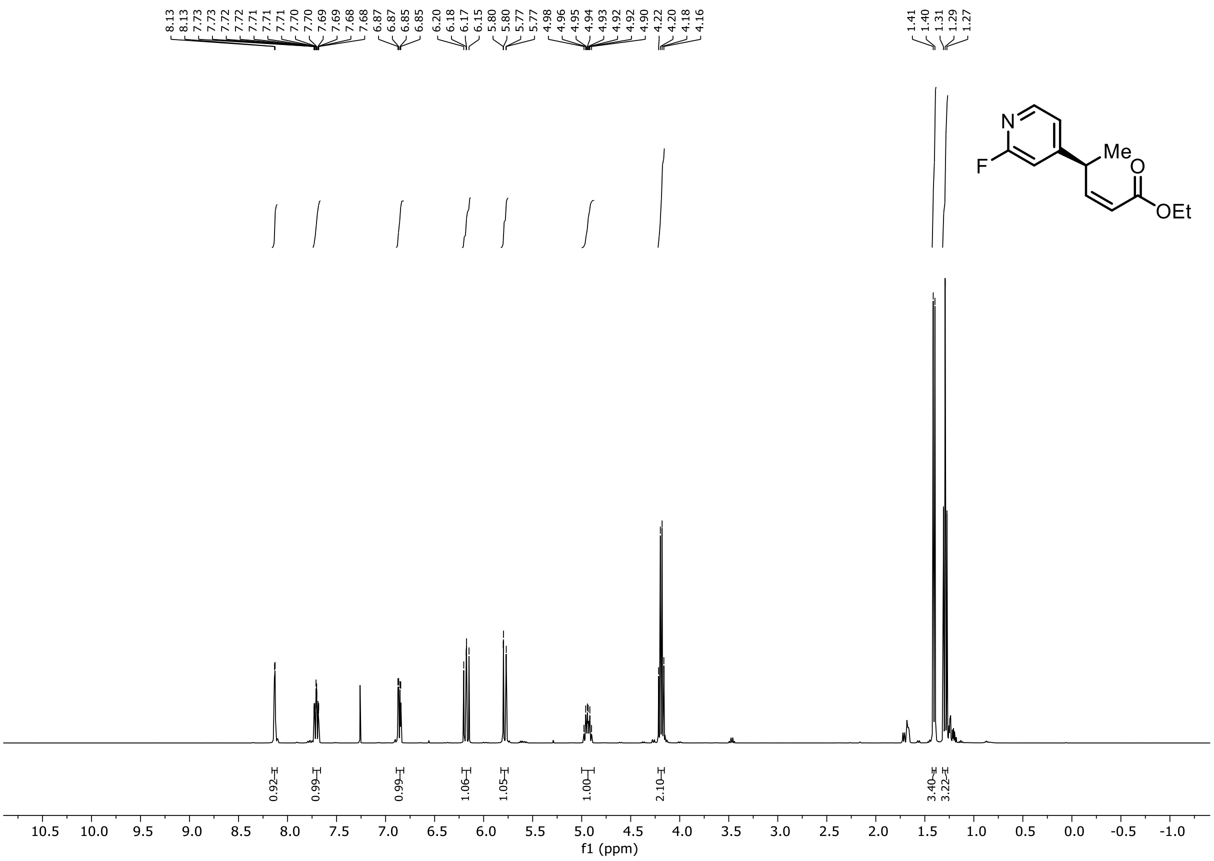

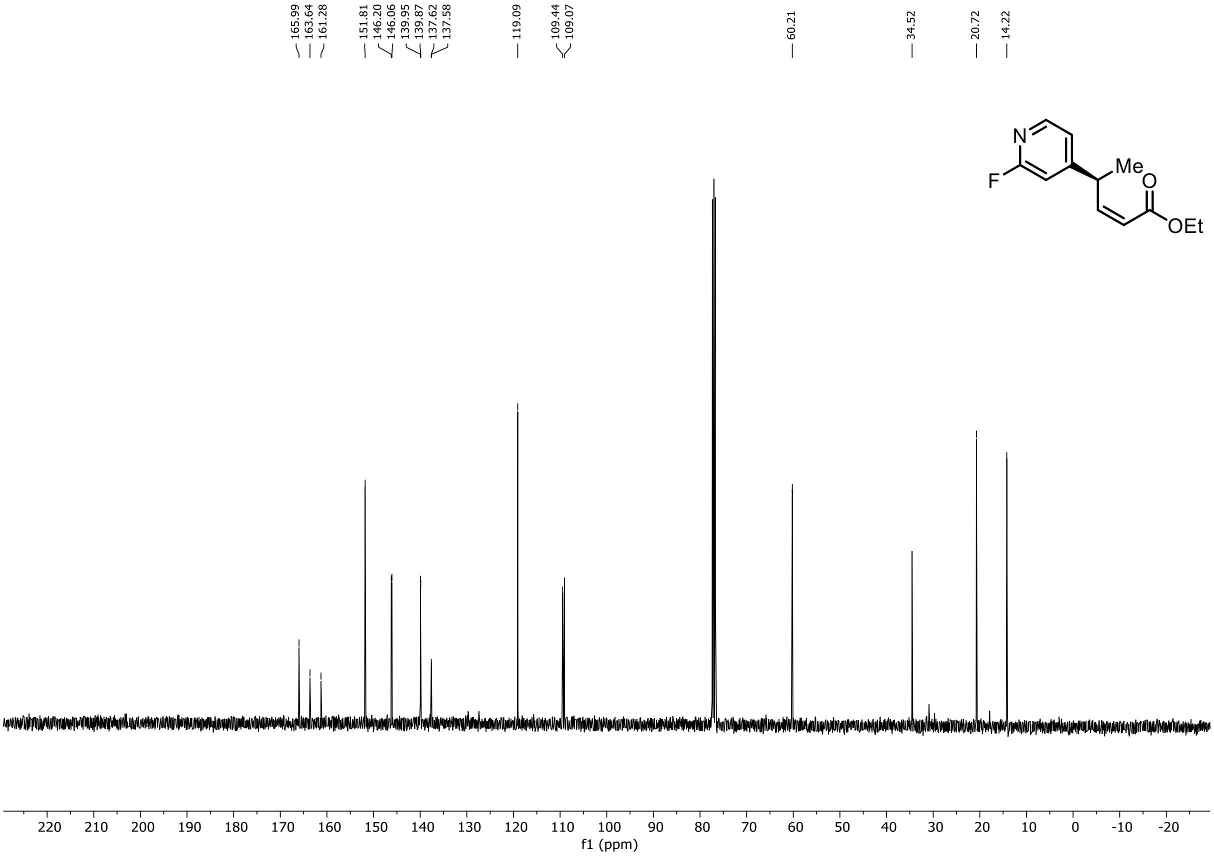

Figure 10.64 (top) ^{1}H NMR (400 MHz) and (bottom) ^{13}C NMR (101 MHz) spectra of *Z*-**3y**.

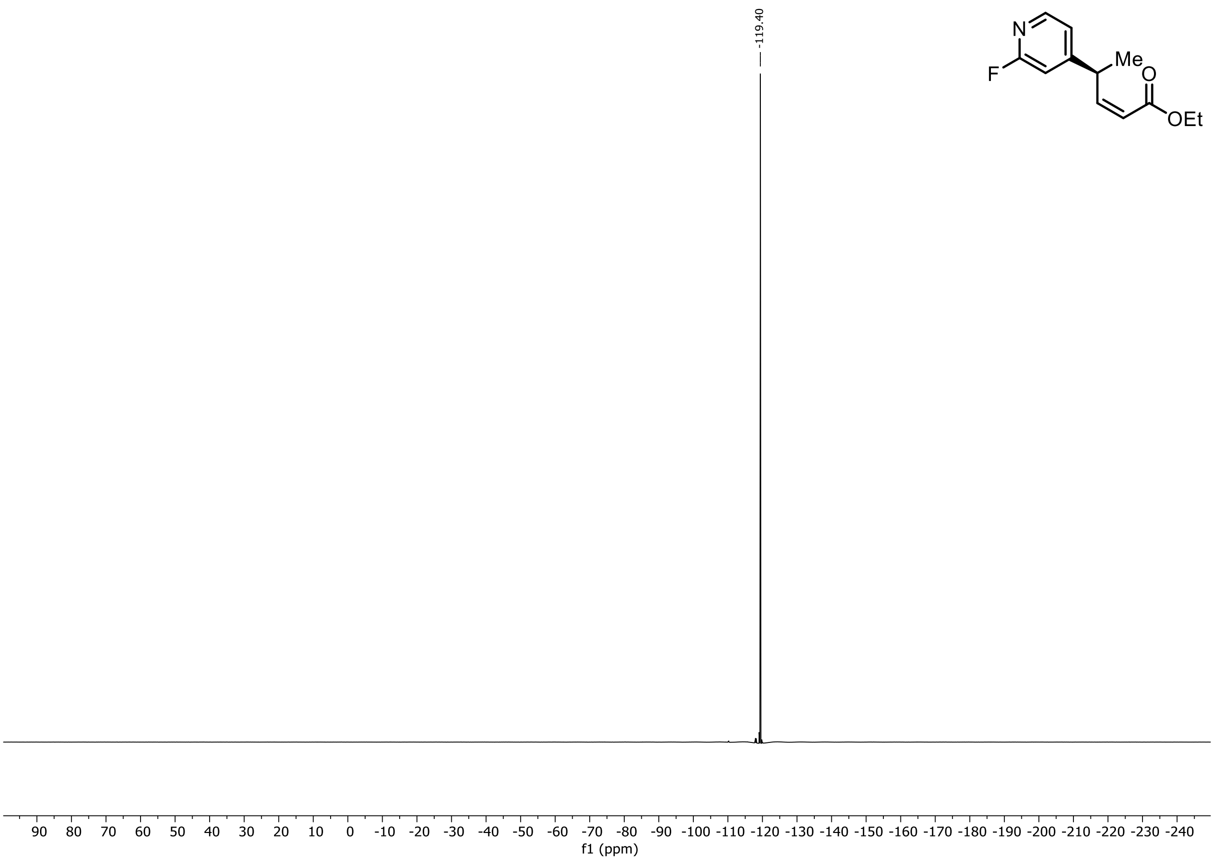

Figure 10.65 ^{19}F (13C)NMR (376 MHz) spectrum of *Z*-**3y**.

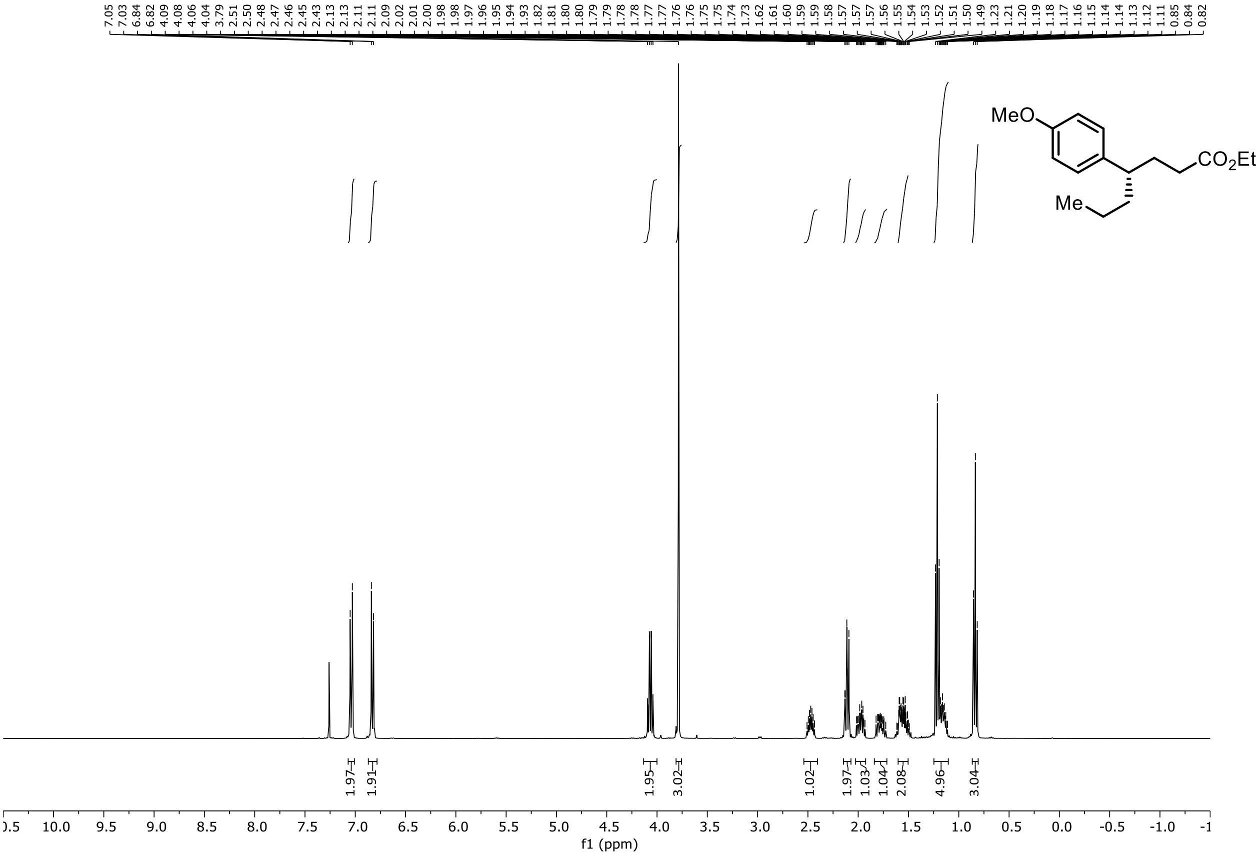

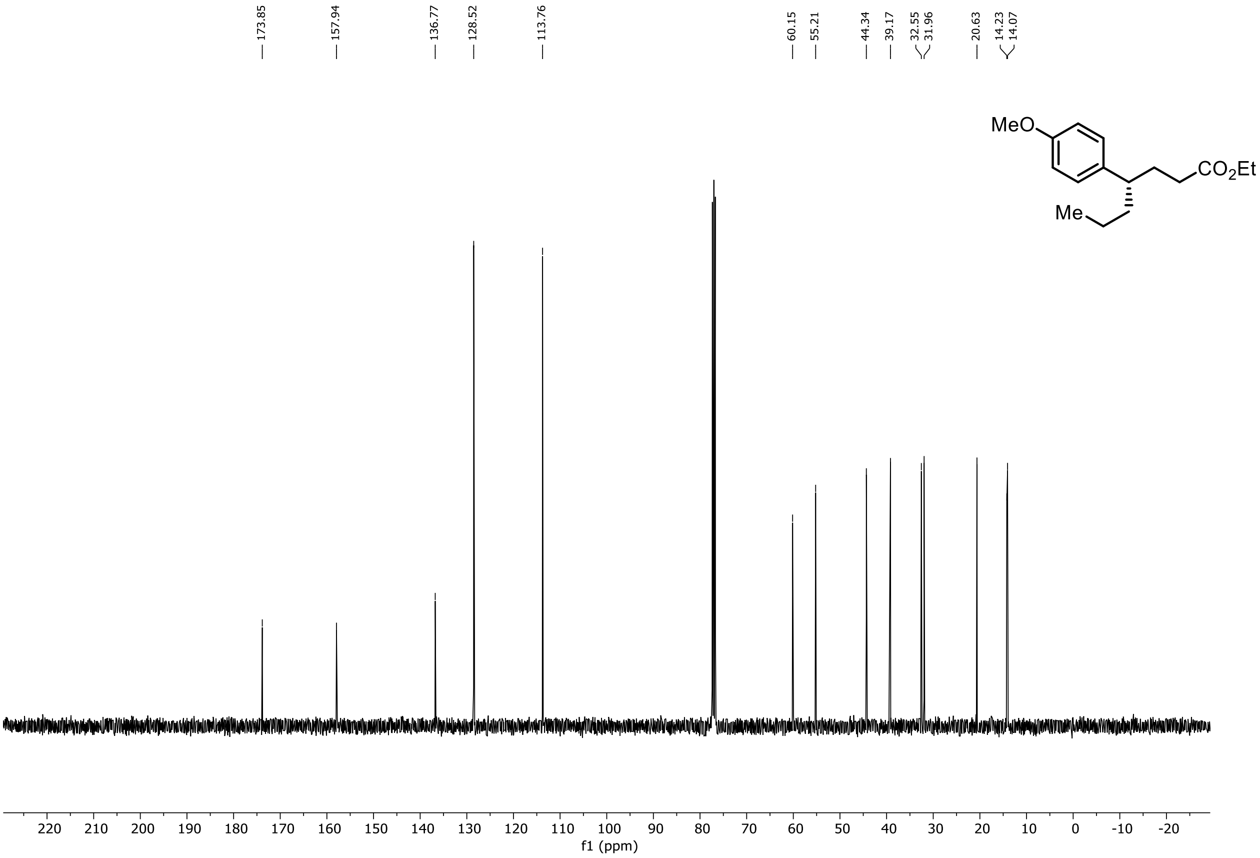

Figure 10.66 (top) ^{1}H NMR (400 MHz) and (bottom) ^{13}C NMR (101 MHz) spectra of *red*-**5a**.

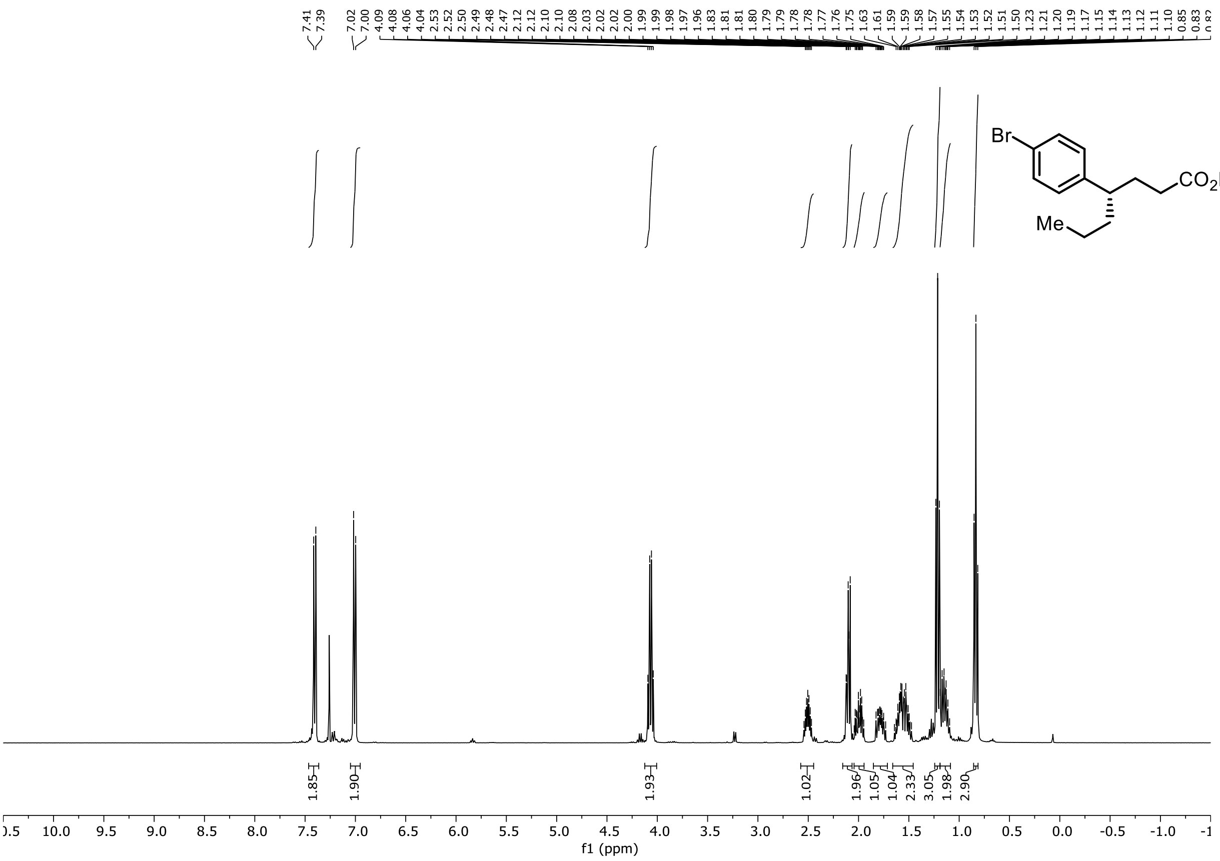

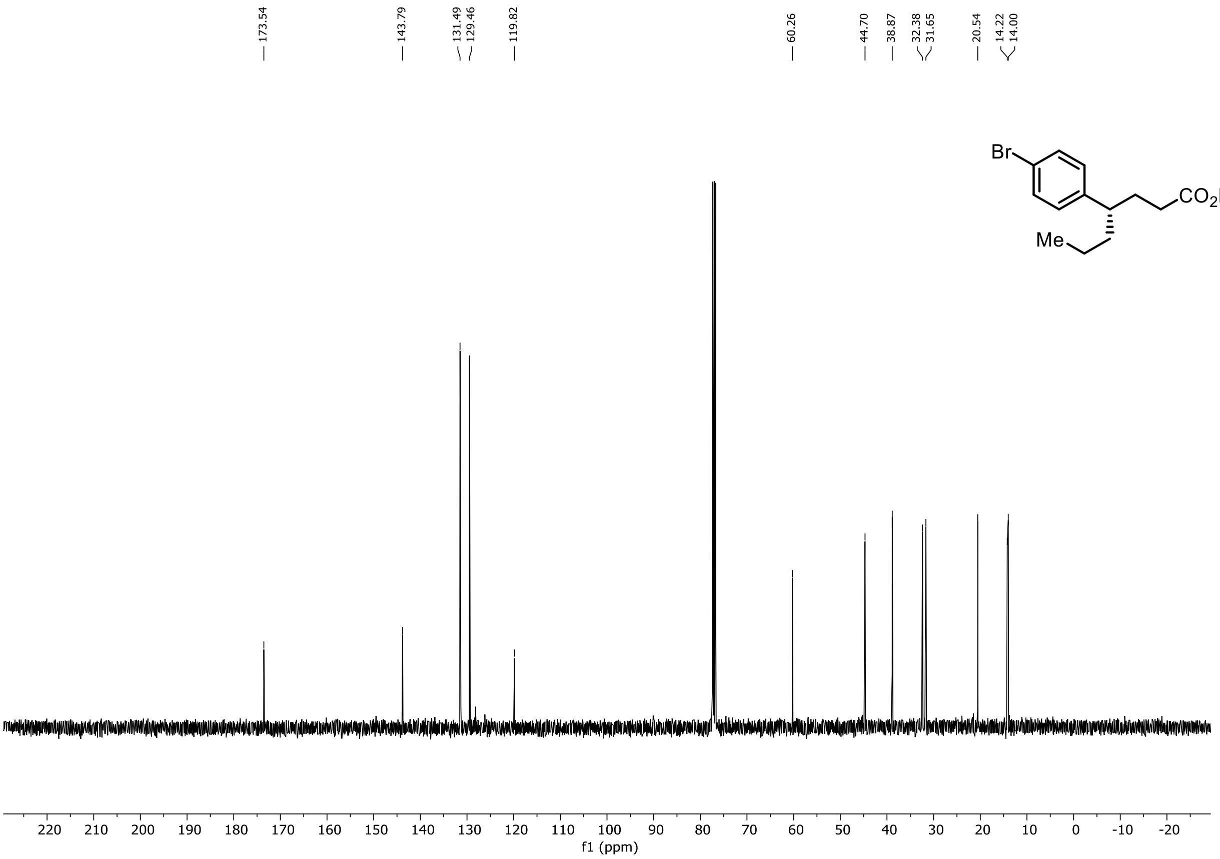

Figure 10.67 (top) ^{1}H NMR (400 MHz) and (bottom) ^{13}C NMR (101 MHz) spectra of *red*-**5b**.

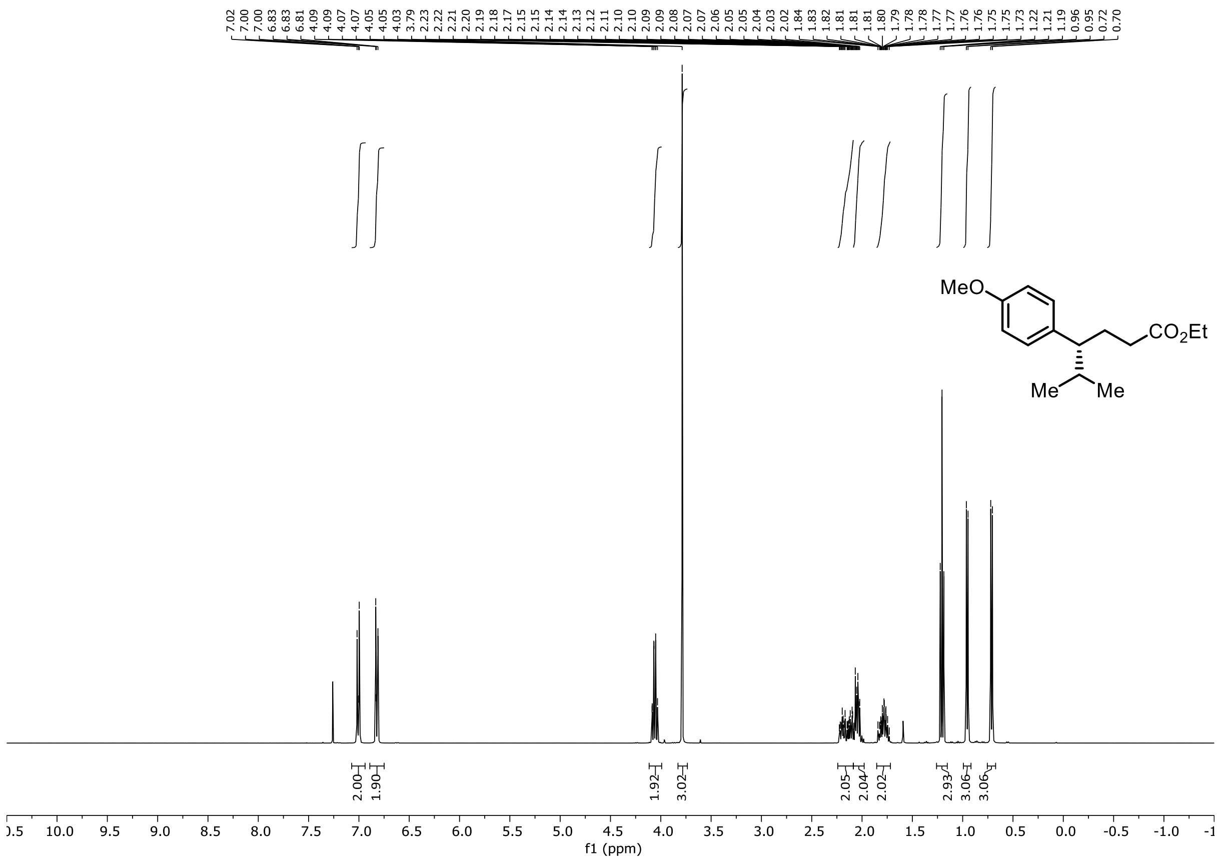

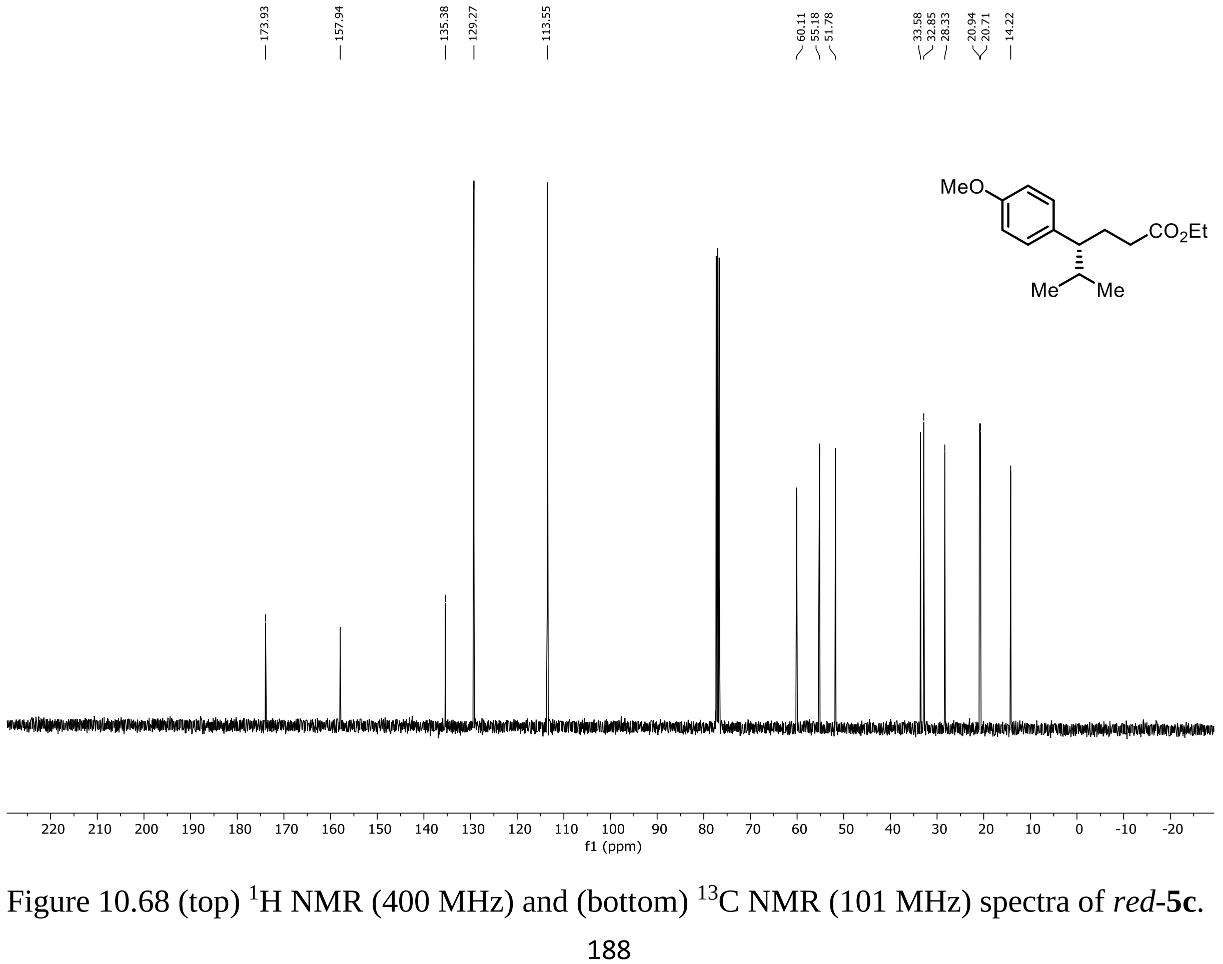

Figure 10.68 (top) ^{1}H NMR (400 MHz) and (bottom) ^{13}C NMR (101 MHz) spectra of *red*-**5c**.

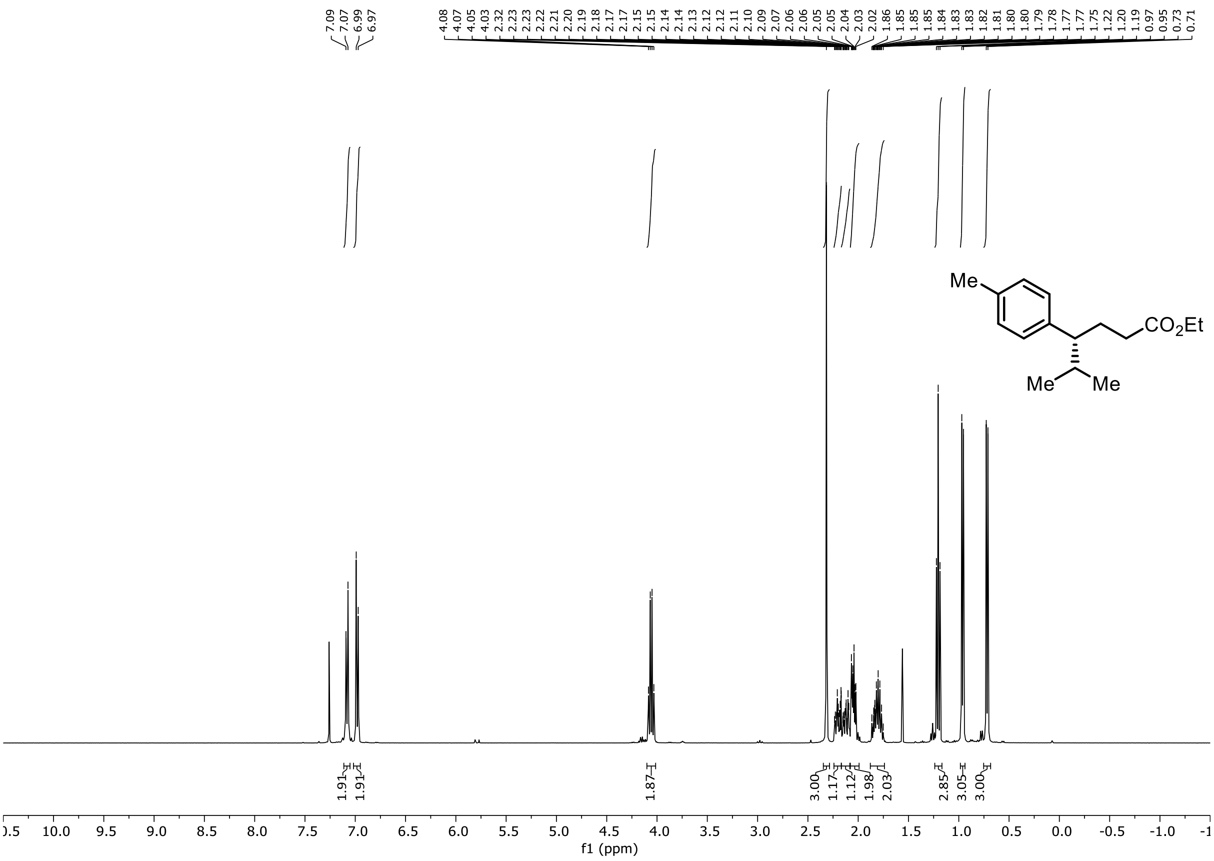

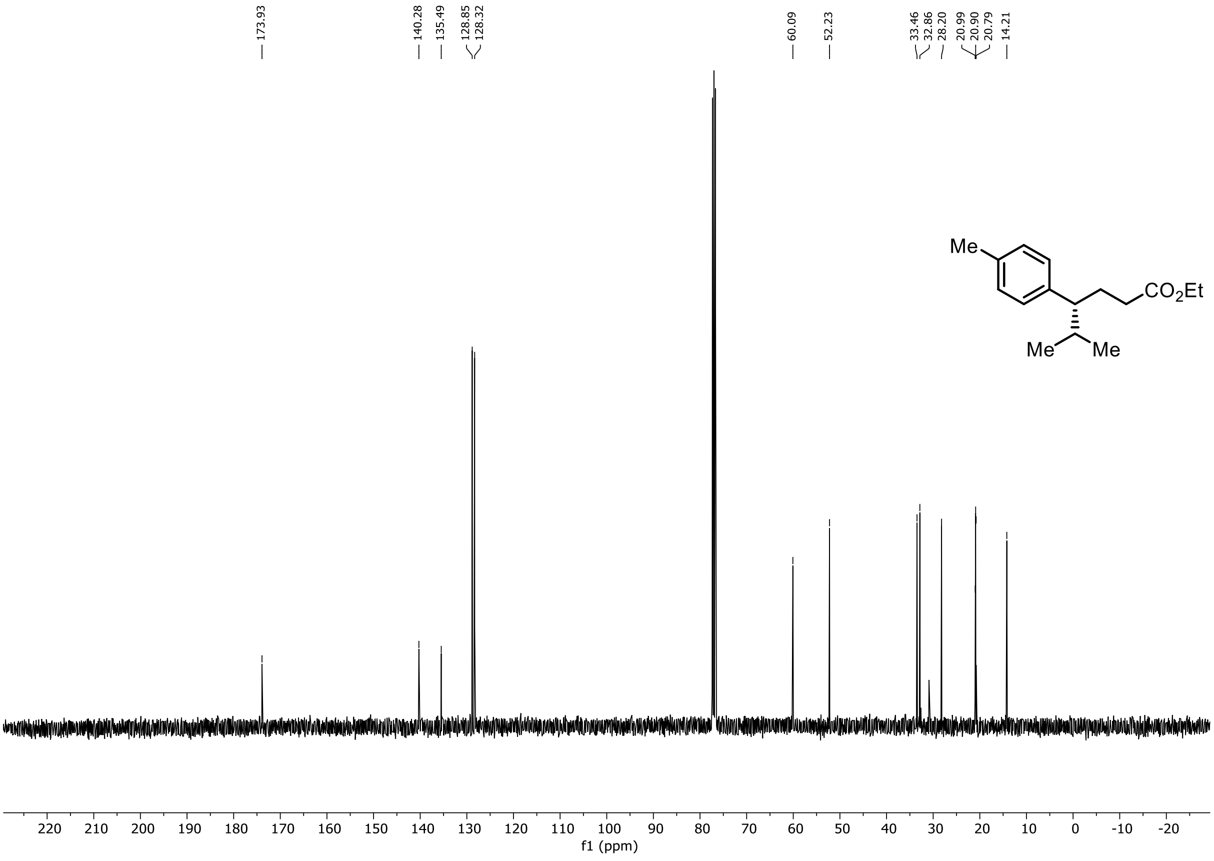

Figure 10.69 (top) ^{1}H NMR (400 MHz) and (bottom) ^{13}C NMR (101 MHz) spectra of *red*-**5d**.

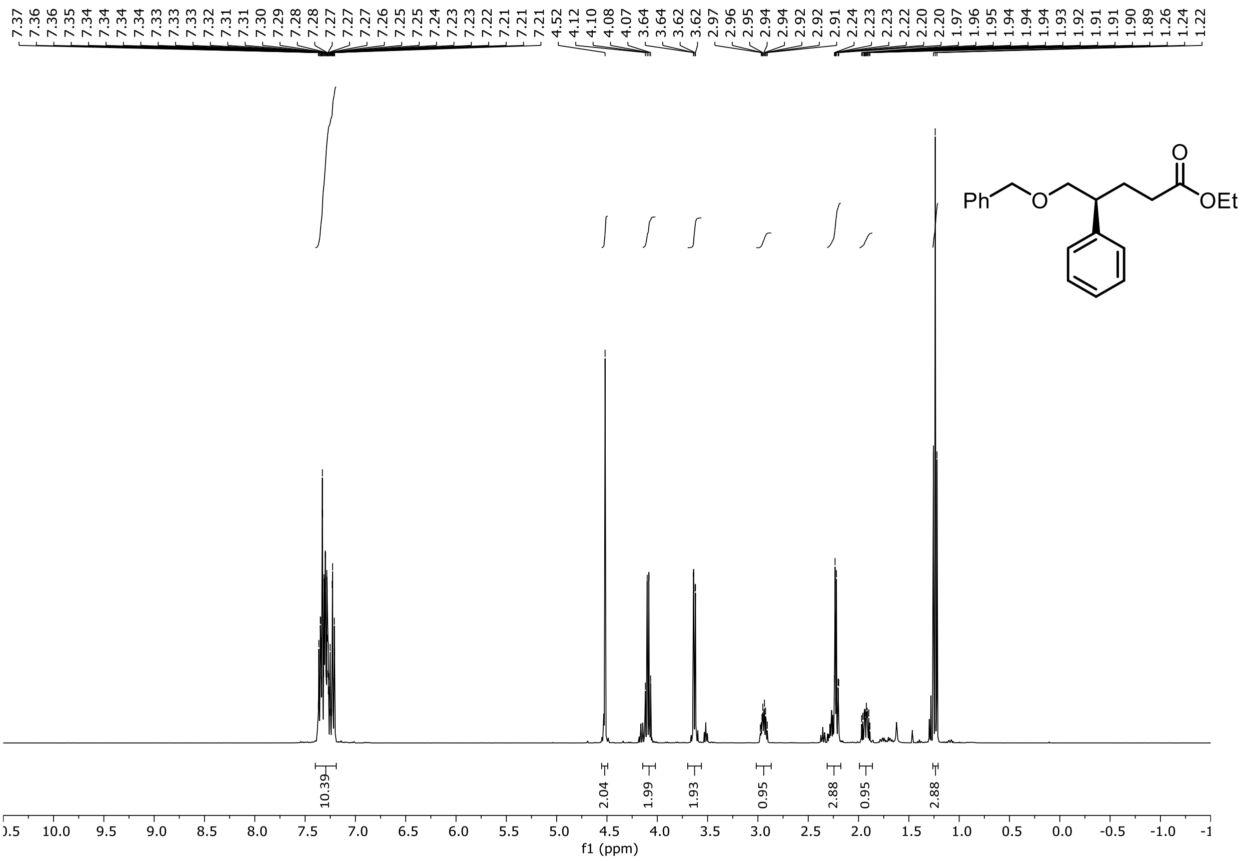

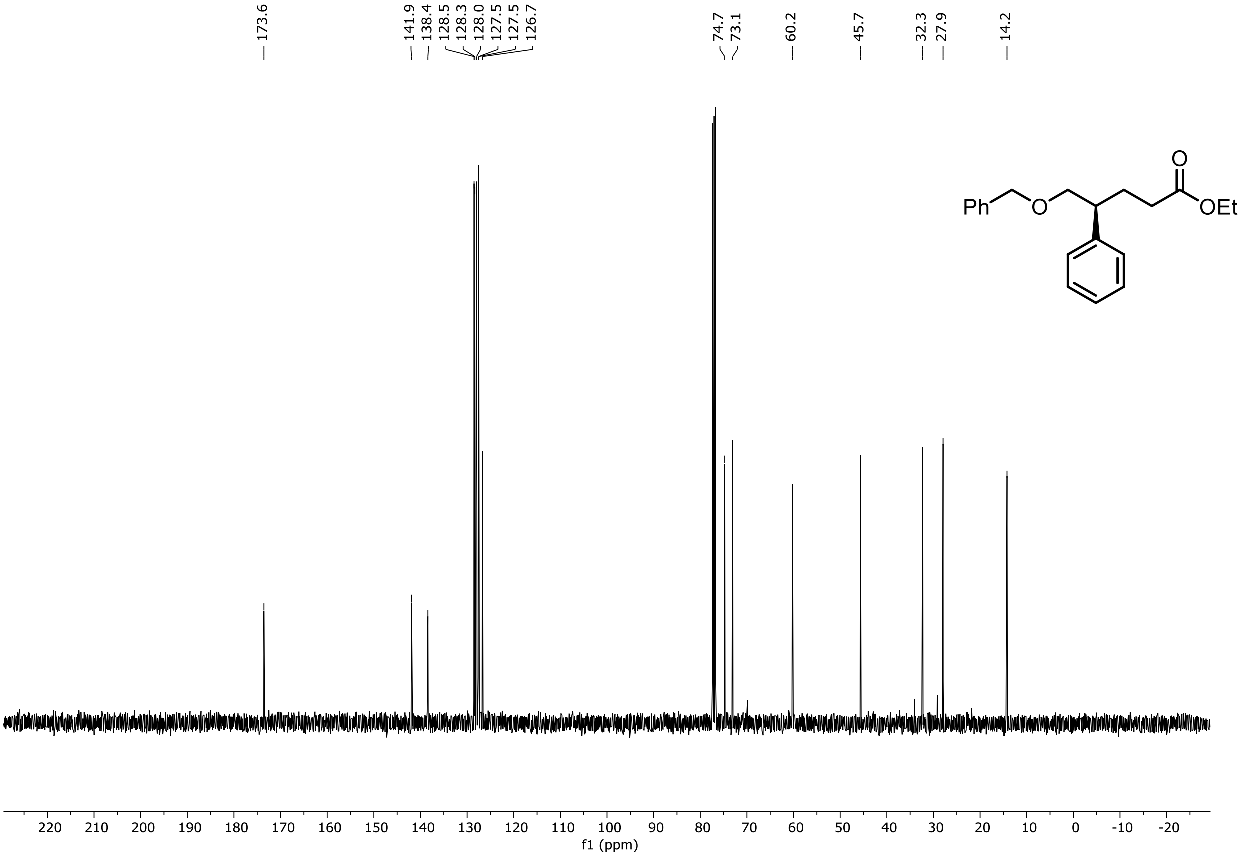

Figure 10.70 (top) ^{1}H NMR (400 MHz) and (bottom) ^{13}C NMR (101 MHz) spectra of *red*-**5e**.

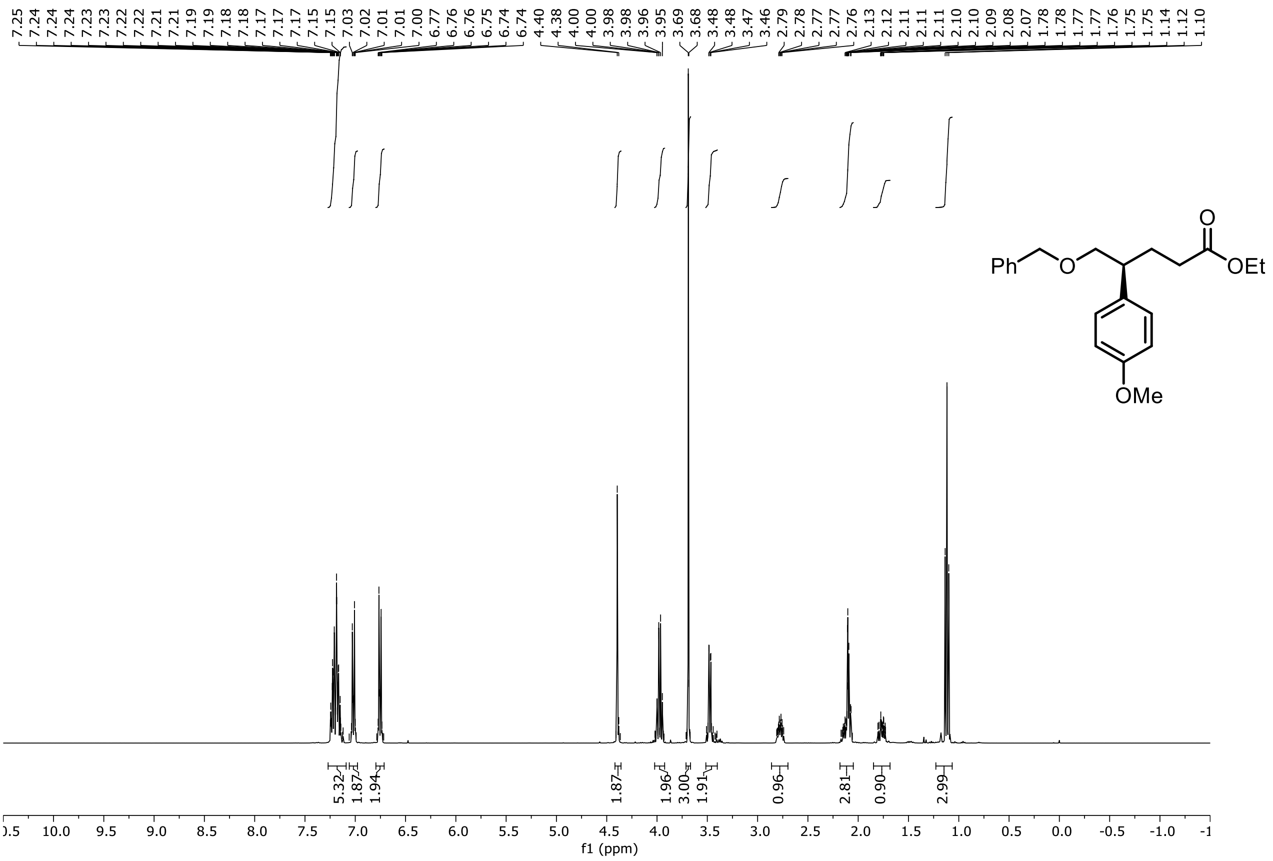

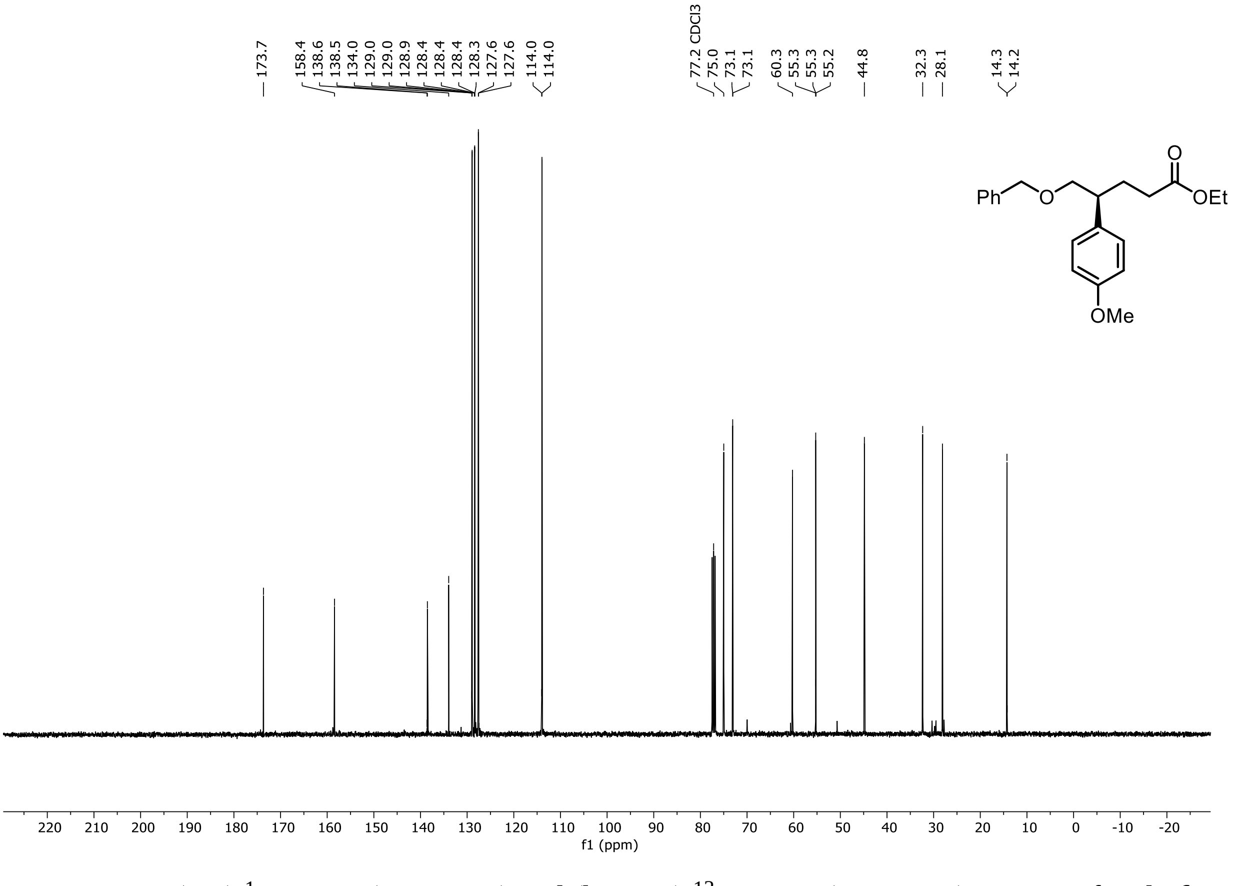

Figure 10.71 (top) ^{1}H NMR (400 MHz) and (bottom) ^{13}C NMR (101 MHz) spectra of *red*-**5f**.

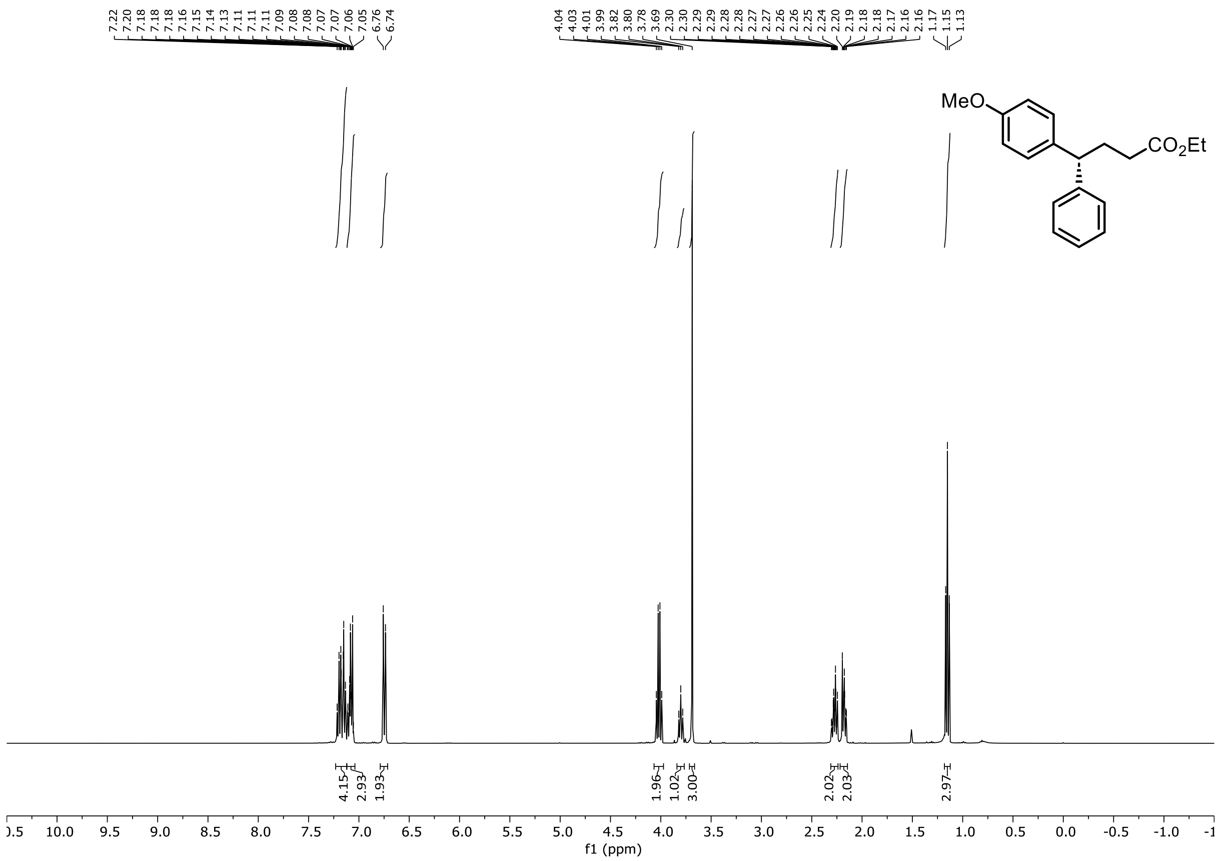

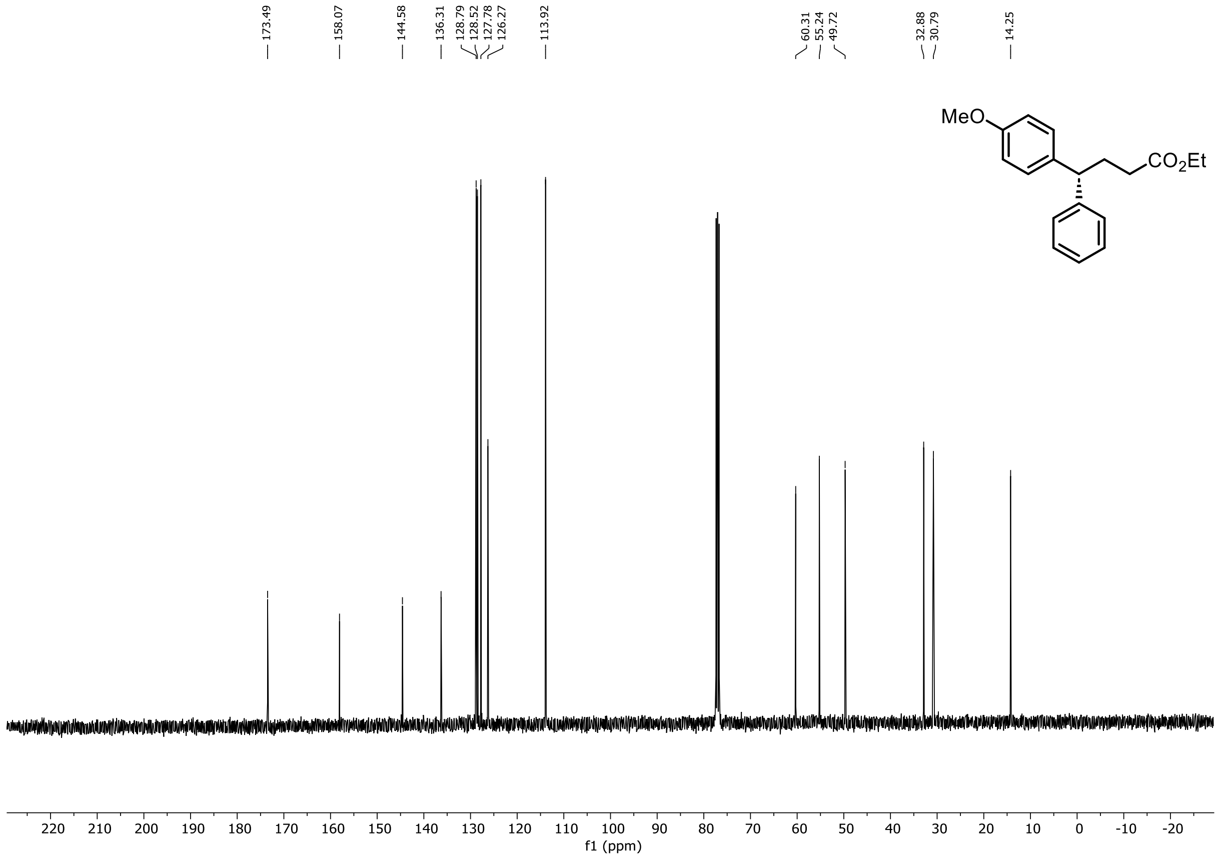

Figure 10.72 (top) ^{1}H NMR (400 MHz) and (bottom) ^{13}C NMR (101 MHz) spectra of *red*-**5g**.

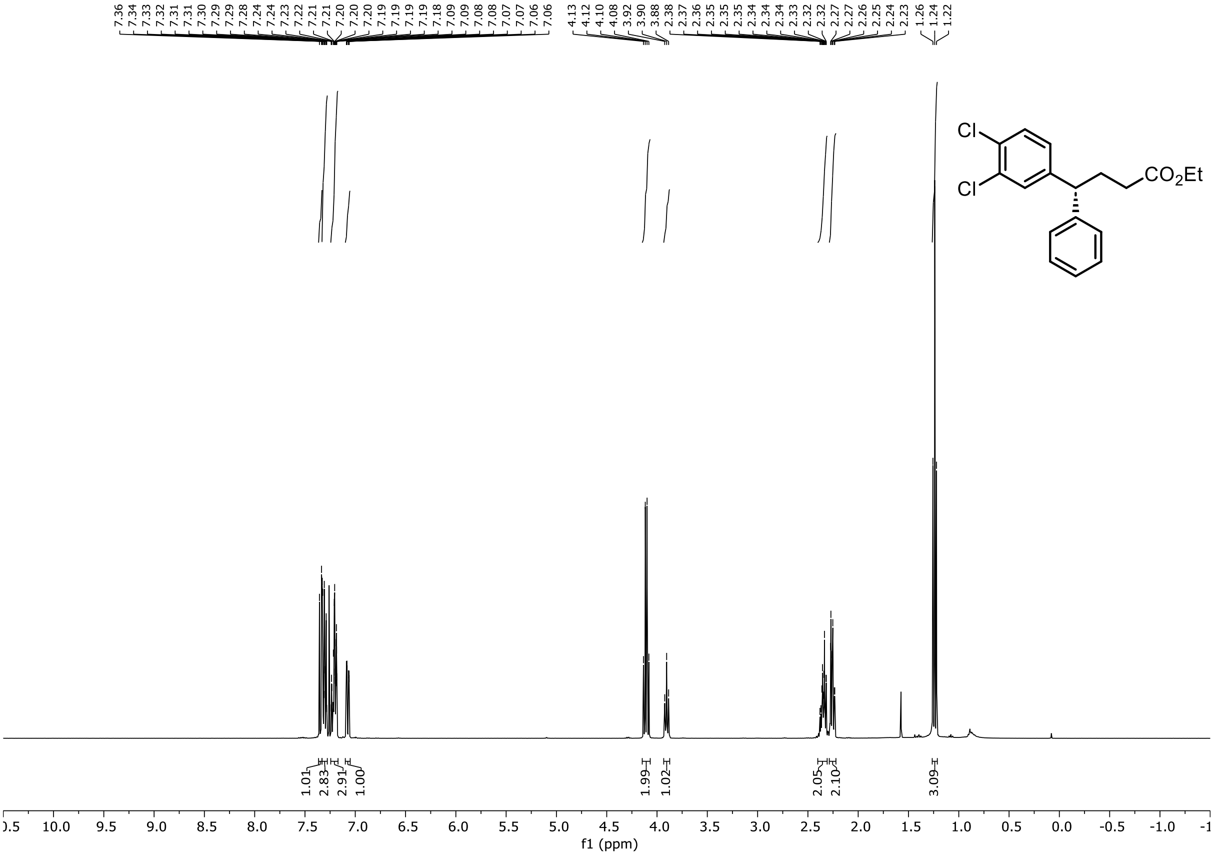

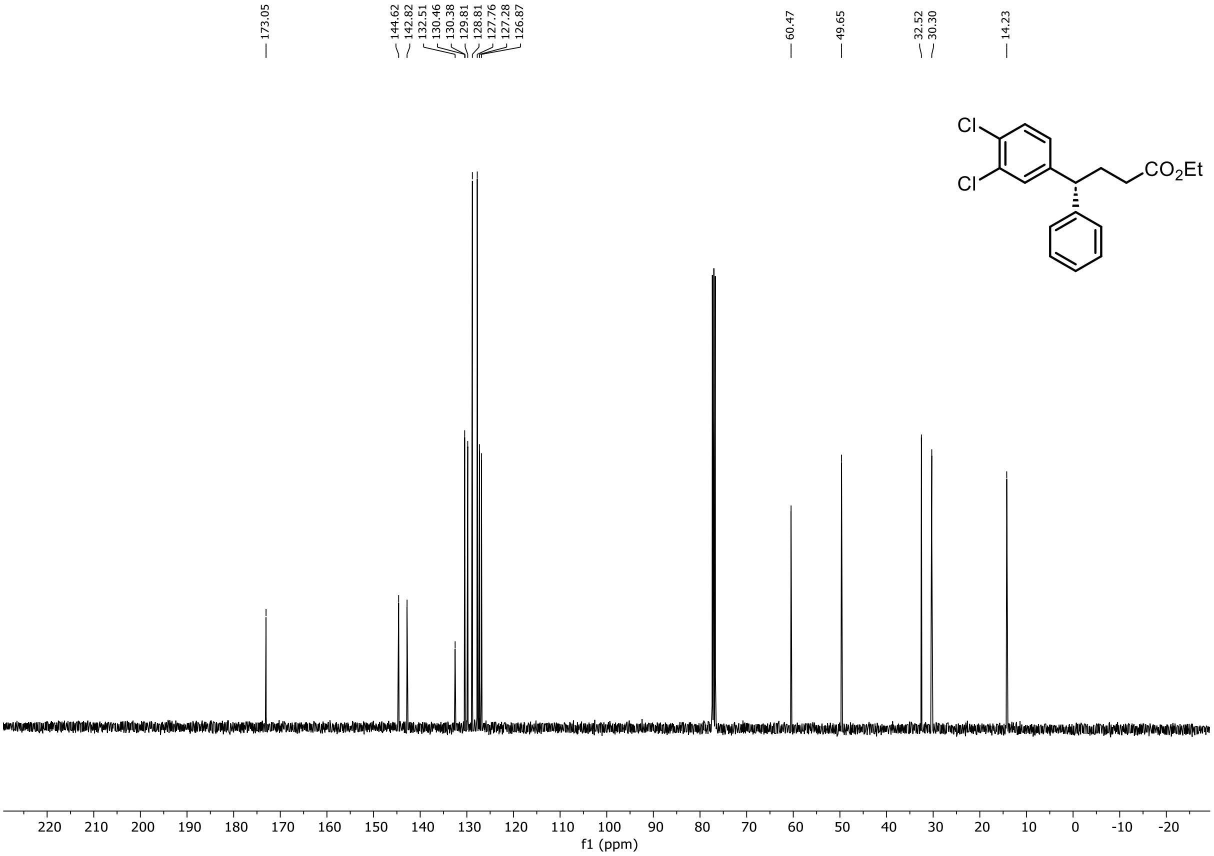

Figure 10.73 (top) ^{1}H NMR (400 MHz) and (bottom) ^{13}C NMR (101 MHz) spectra of *red*-**5h**.

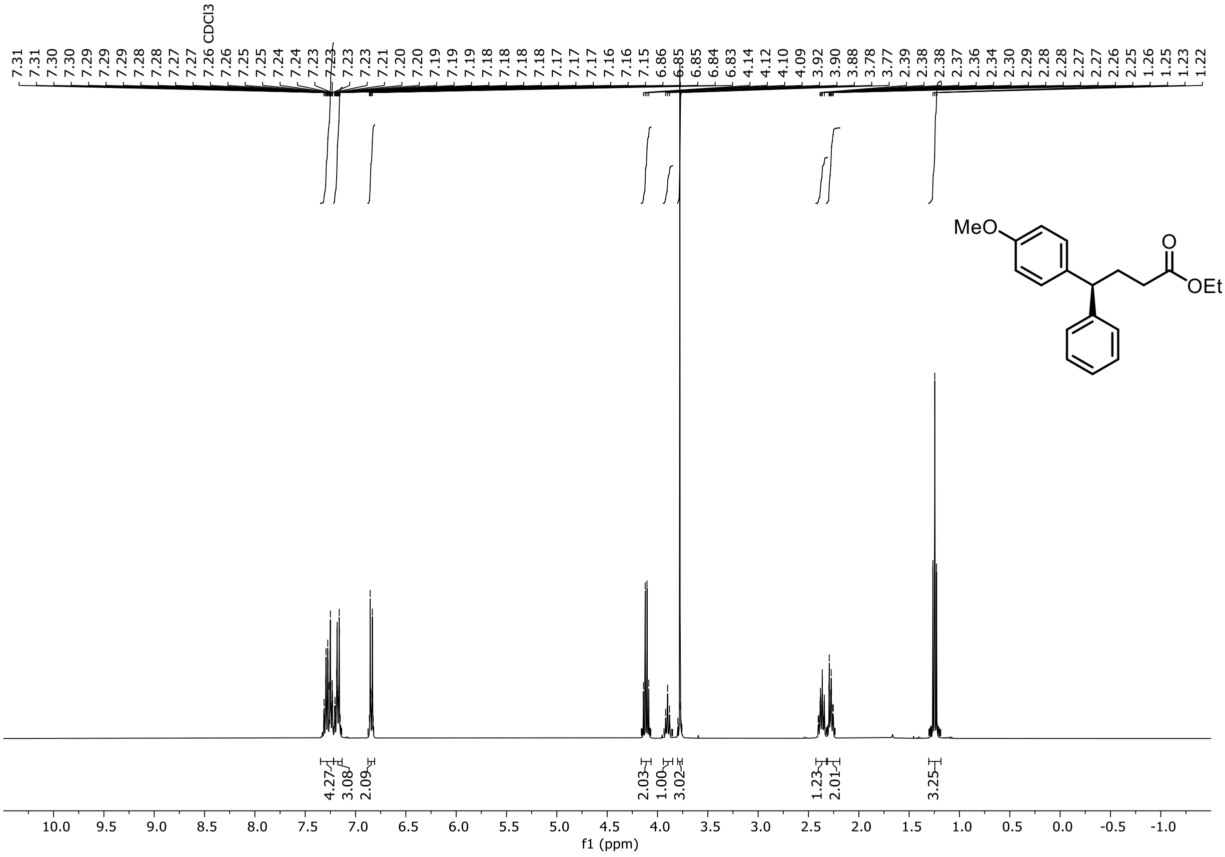

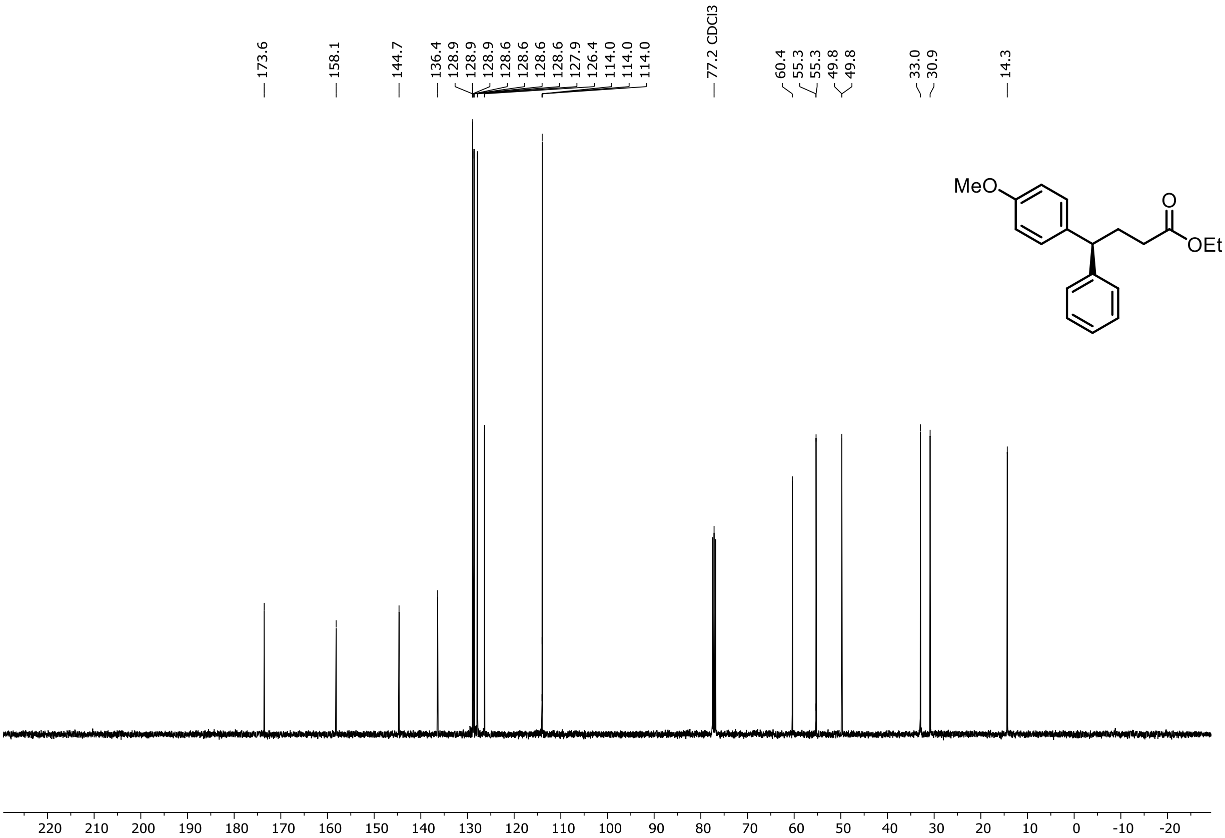

Figure 10.74 (top) ^{1}H NMR (400 MHz) and (bottom) ^{13}C NMR (101 MHz) spectra of *red*-**5i**.

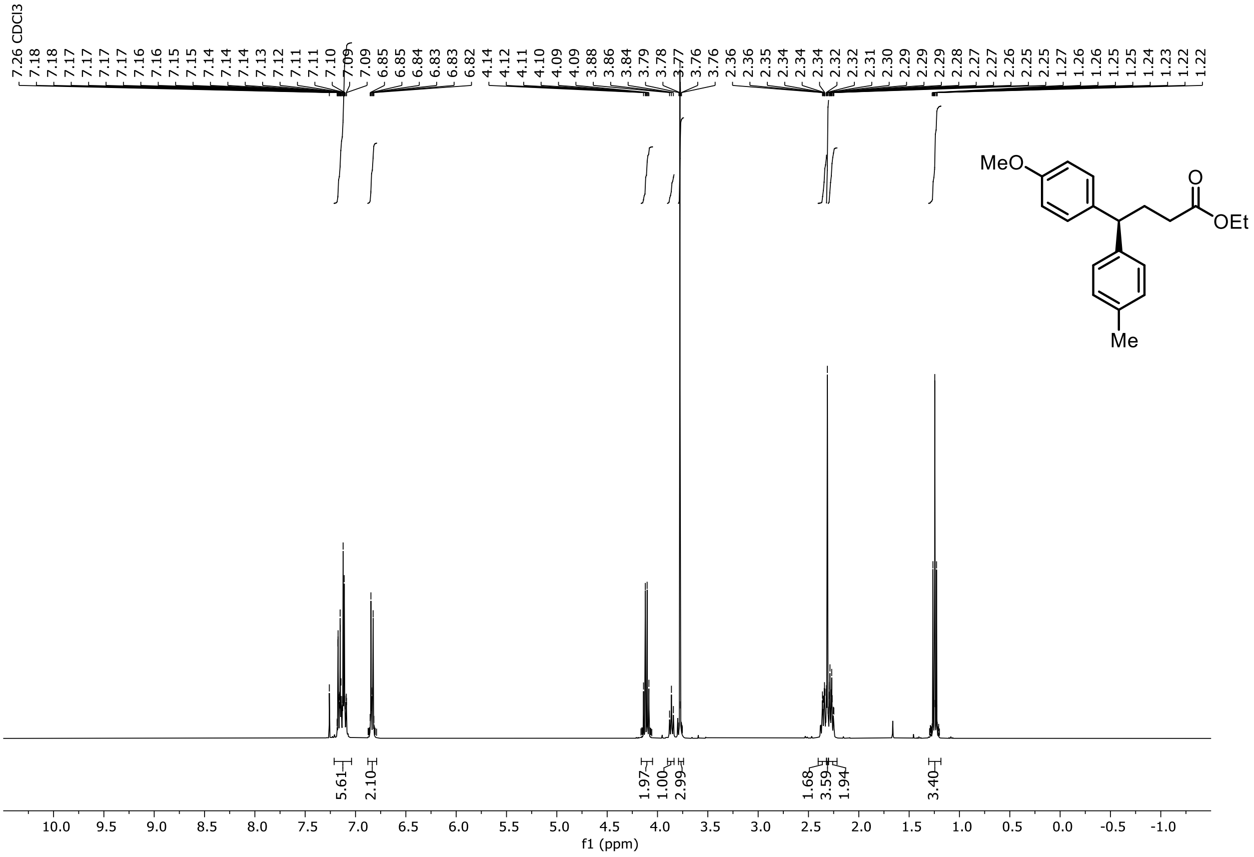

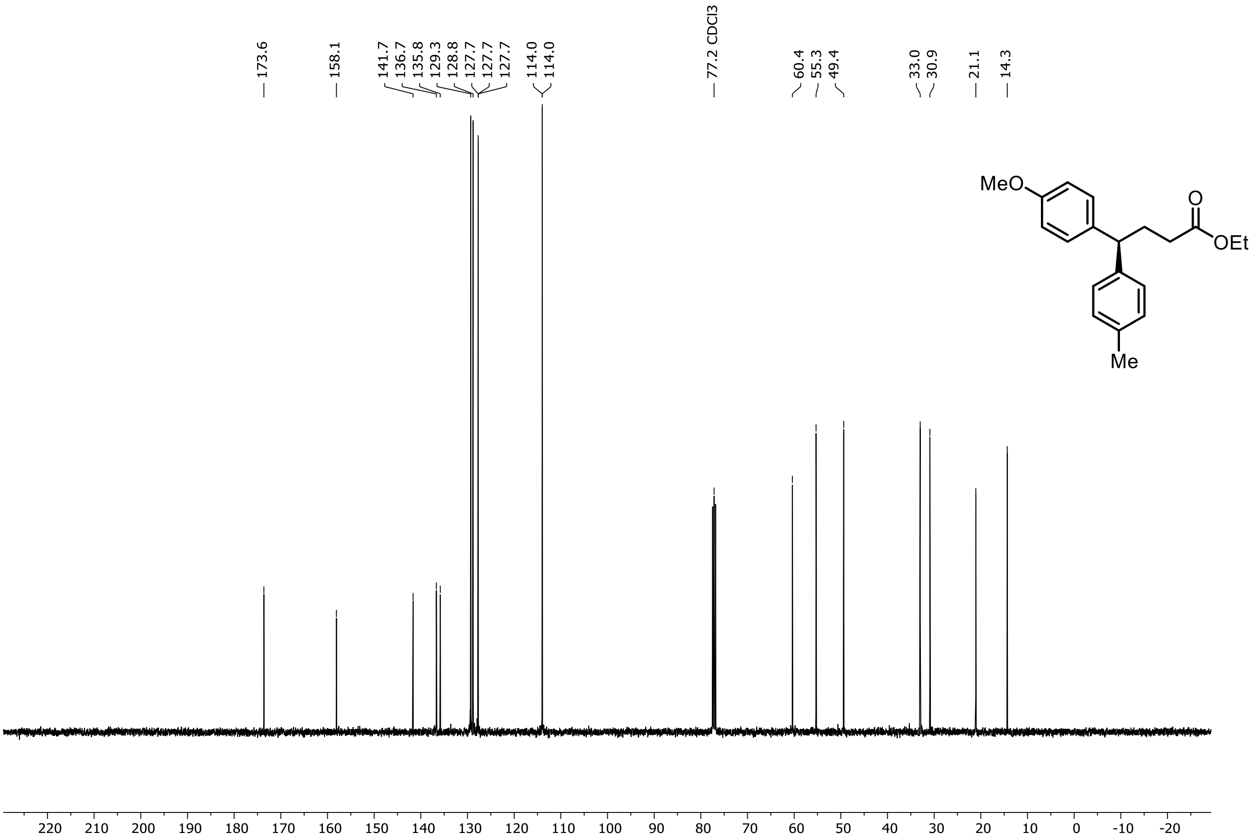

Figure 10.75 (top) ^{1}H NMR (400 MHz) and (bottom) ^{13}C NMR (101 MHz) spectra of *red*-**5j**.

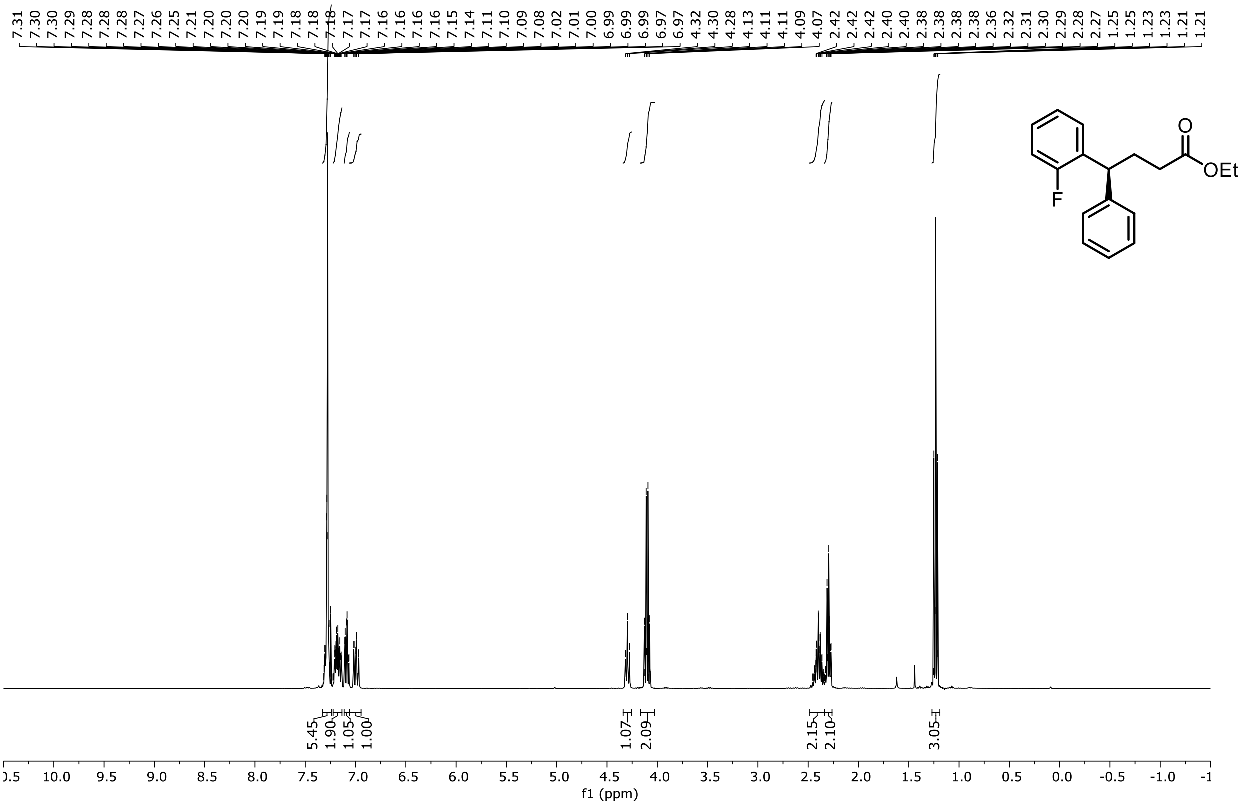

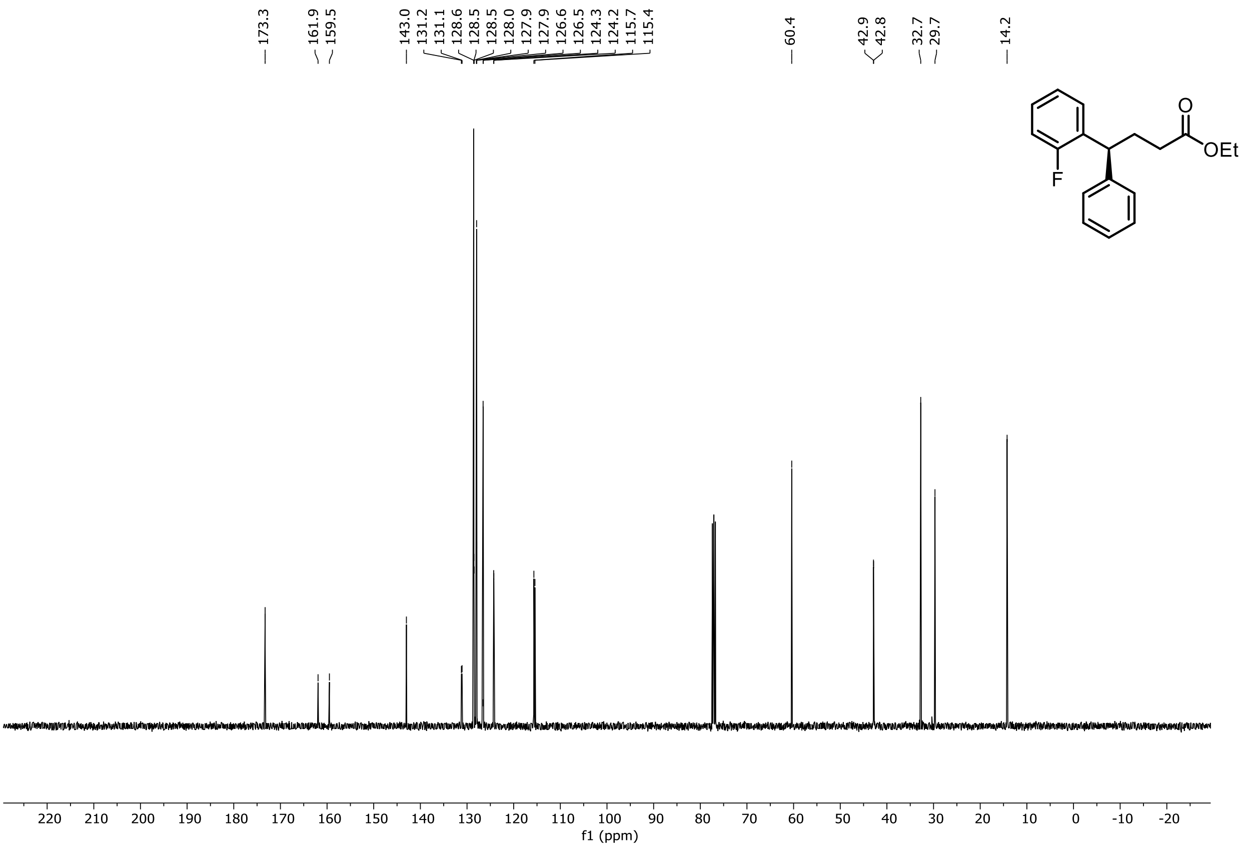

Figure 10.76 (top) ^{1}H NMR (400 MHz) and (bottom) ^{13}C NMR (101 MHz) spectra of *red*-**5k**.

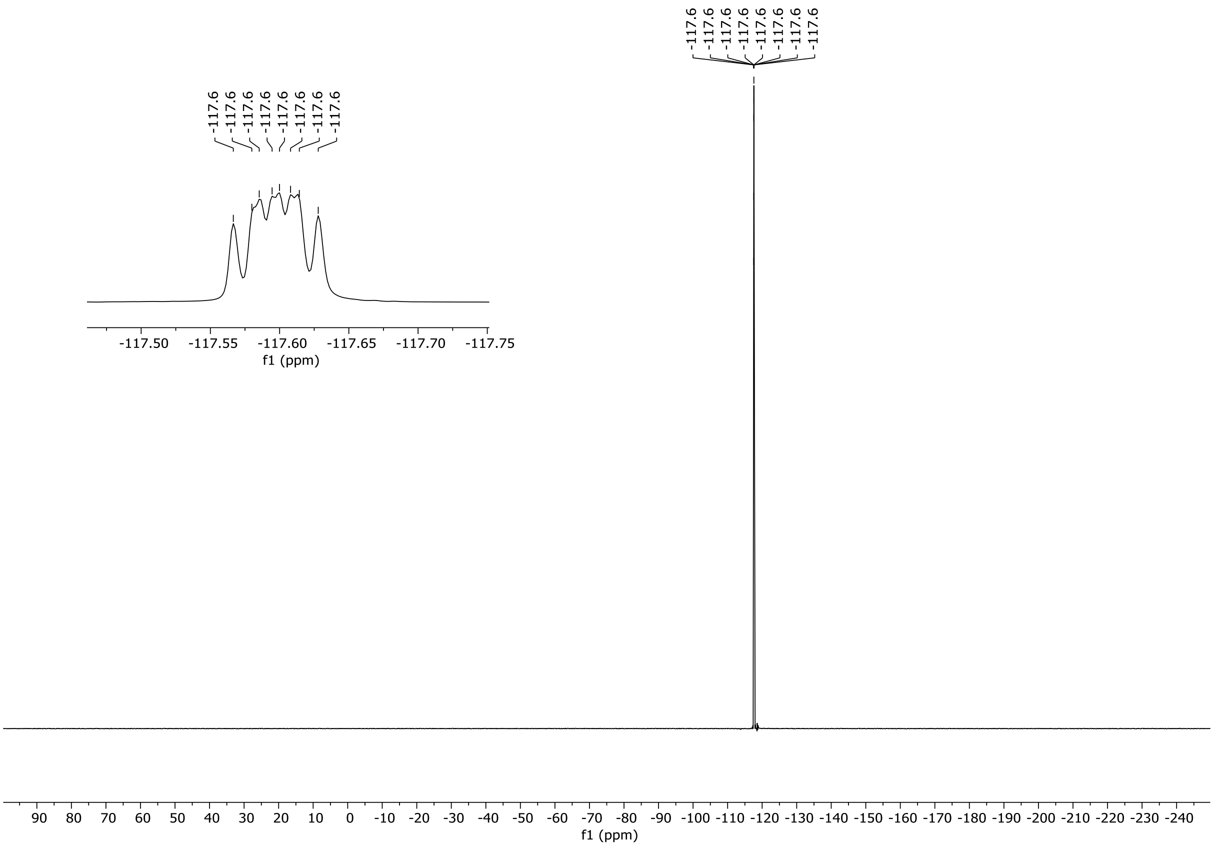

Figure 10.77 ^{19}F NMR (376 MHz) spectrum of *red*-**5k**.

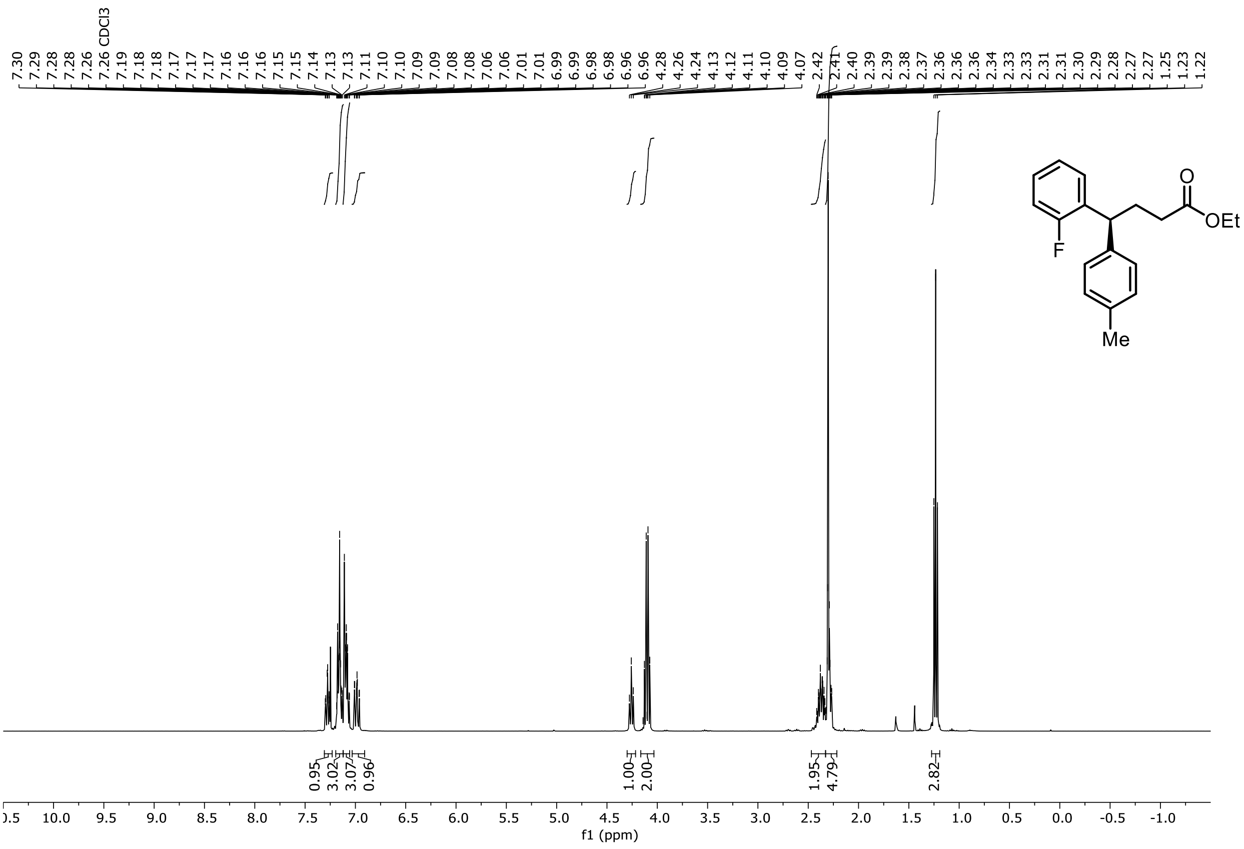

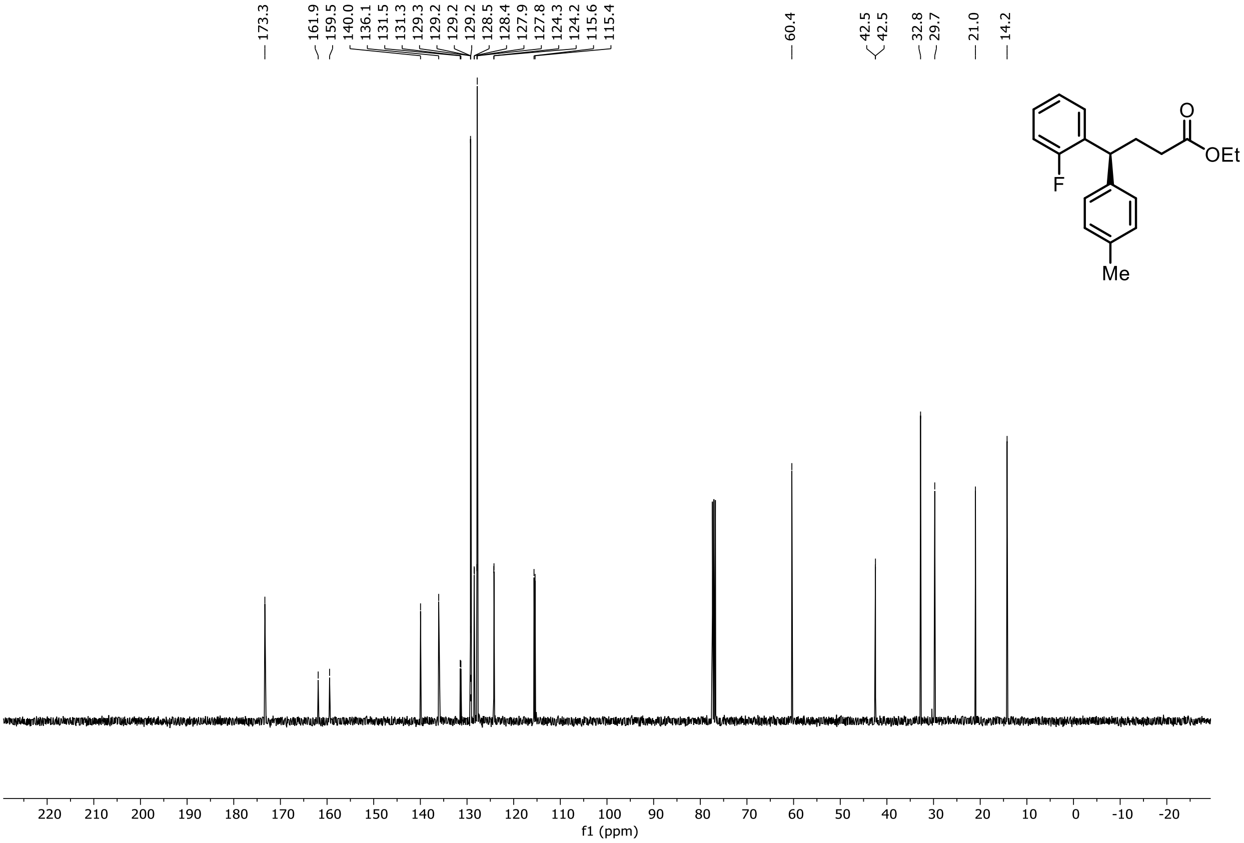

Figure 10.78 (top) ^{1}H NMR (400 MHz) and (bottom) ^{13}C NMR (101 MHz) spectra of *red*-**5l**.

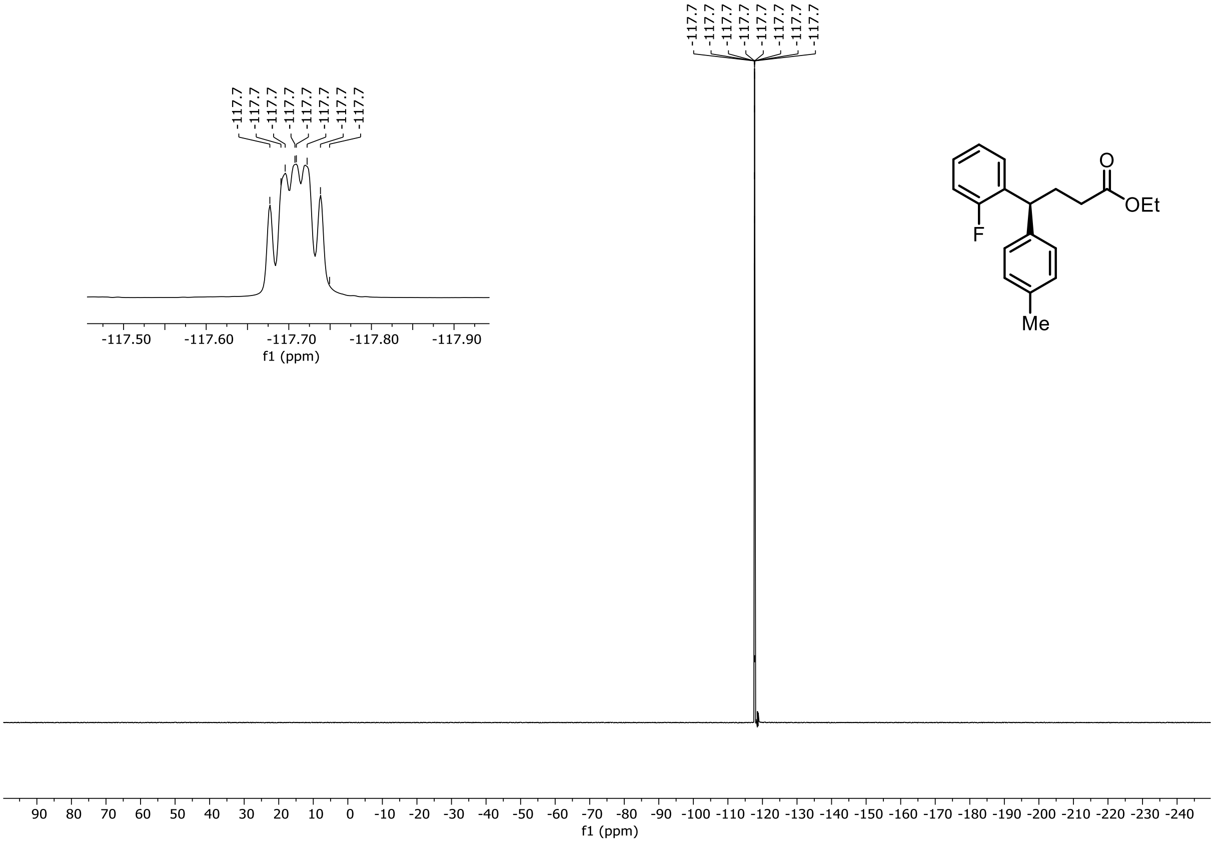

Figure 10.79 ^{19}F NMR (376 MHz) spectrum of *red*-**5l**.

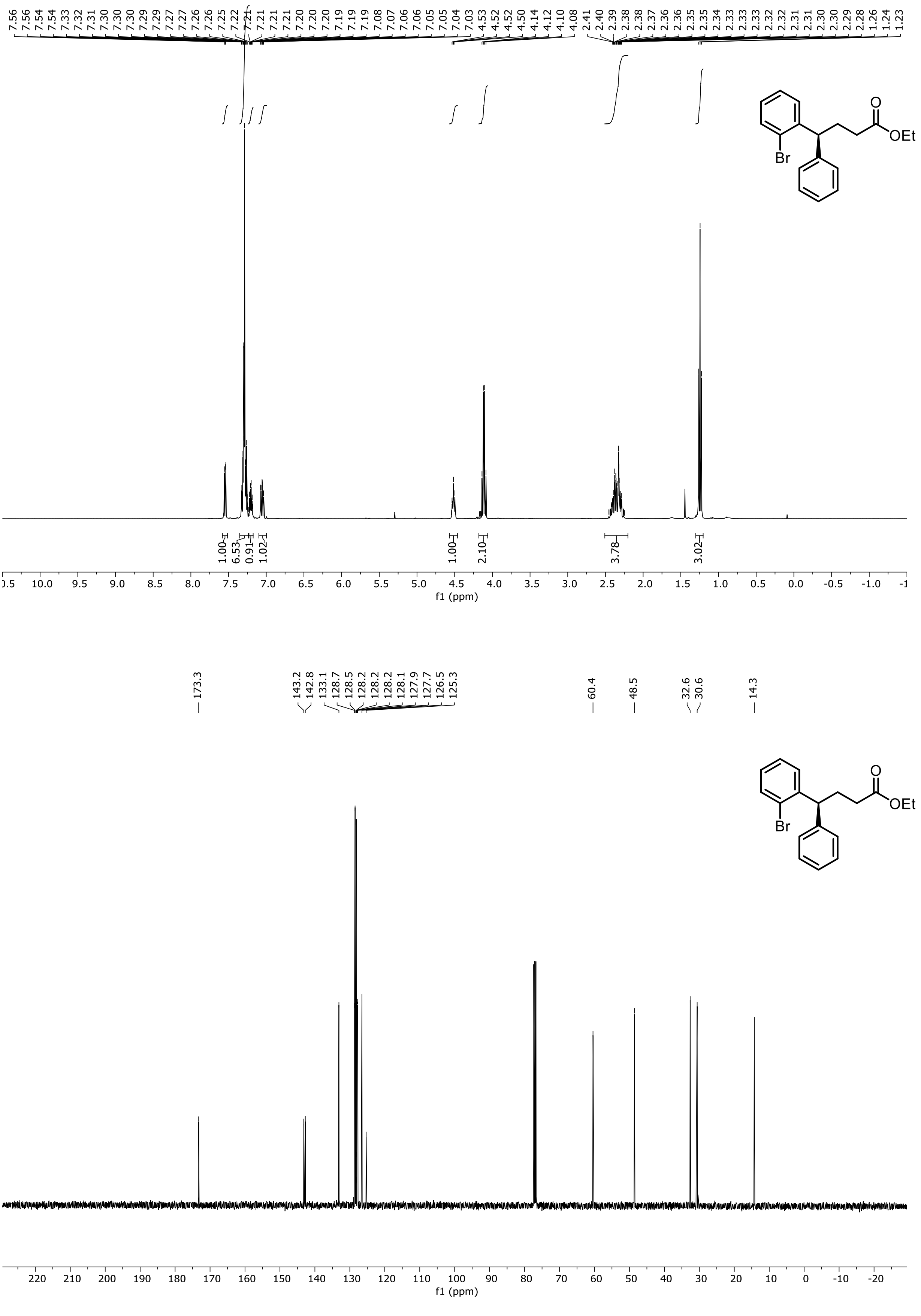

Figure 10.80 (top) ^{1}H NMR (400 MHz) and (bottom) ^{13}C NMR (101 MHz) spectra of *red*-**5m**.

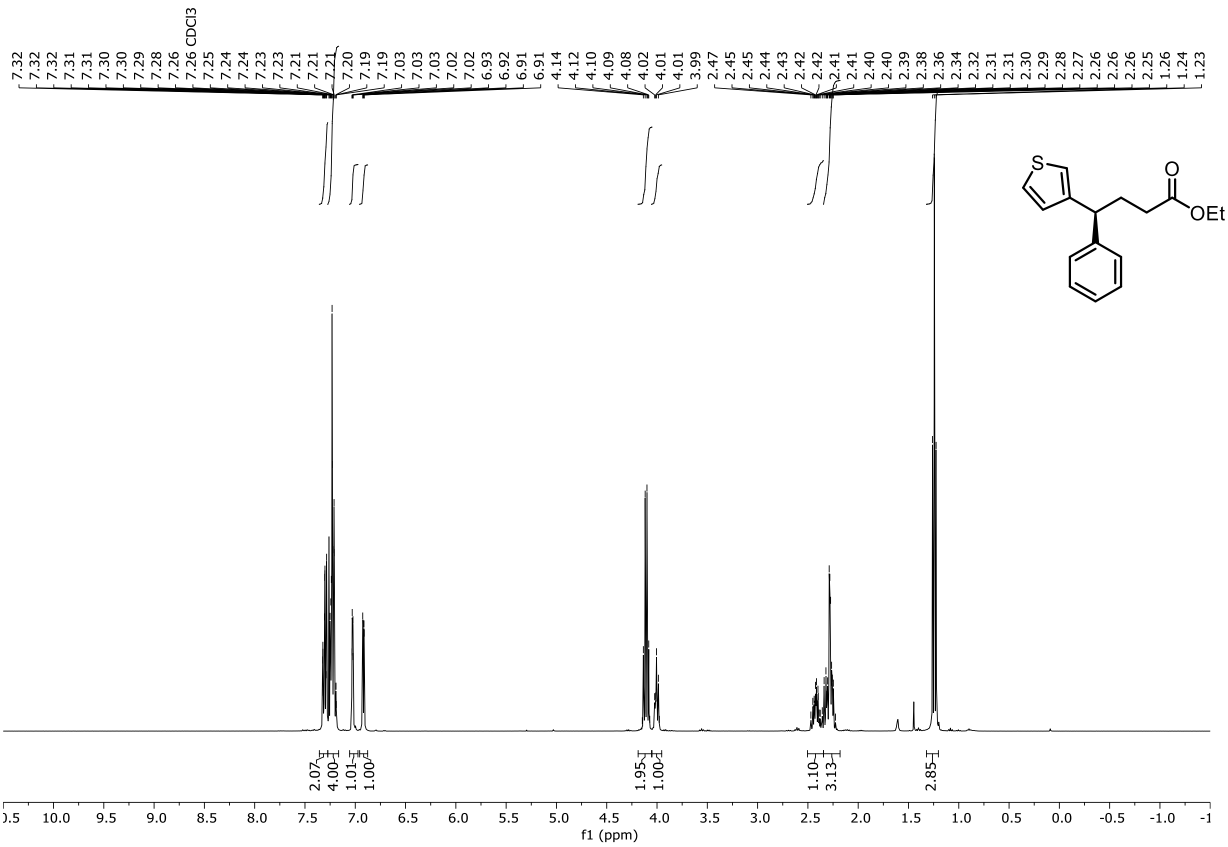

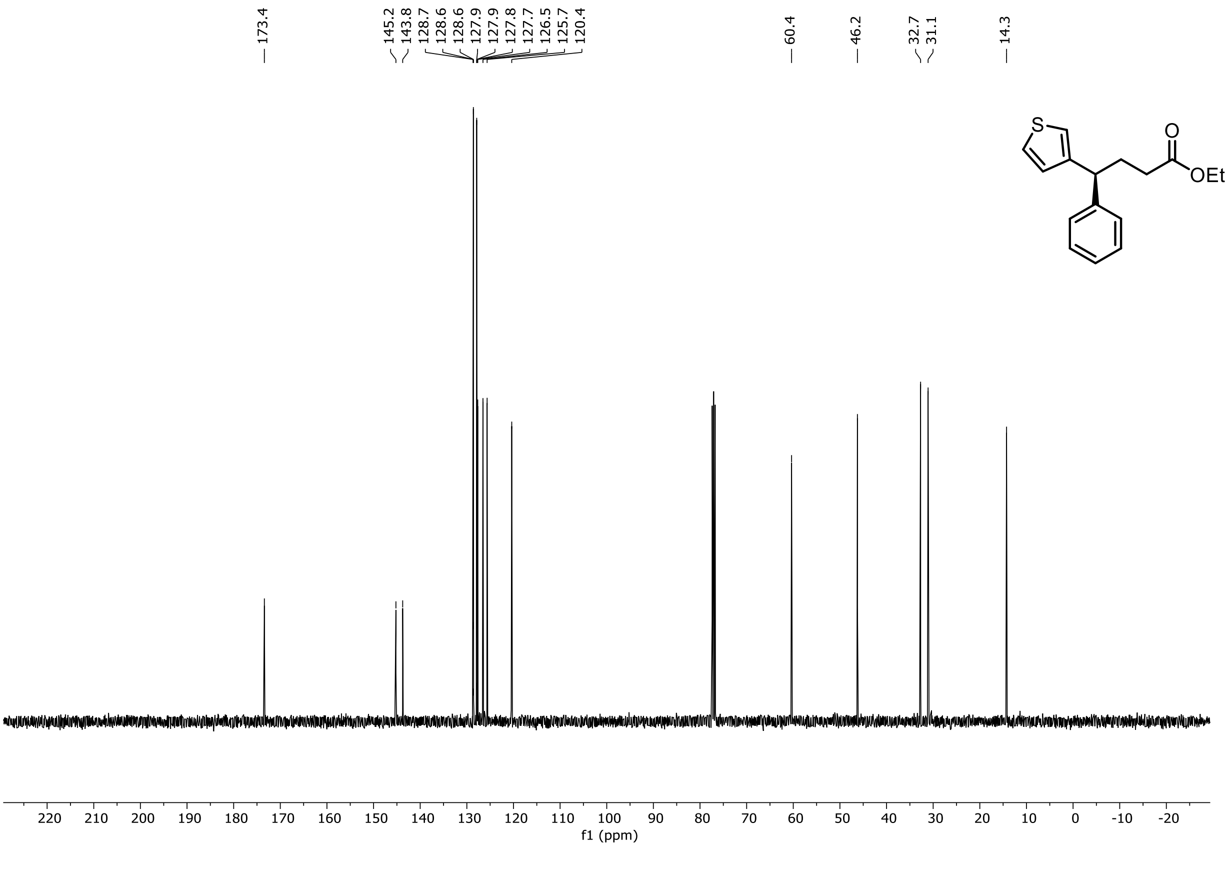

Figure 10.81 (top) ^{1}H NMR (400 MHz) and (bottom) ^{13}C NMR (101 MHz) spectra of *red*-**5n**.

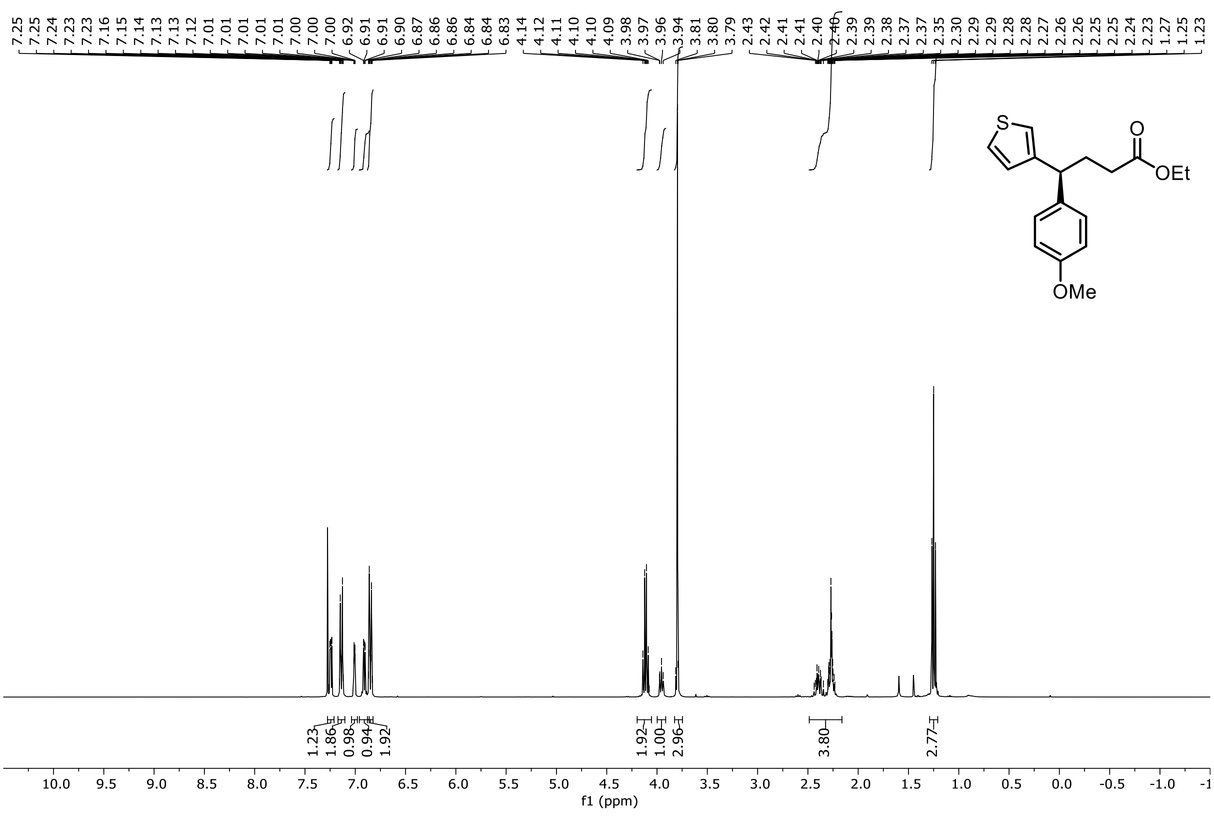

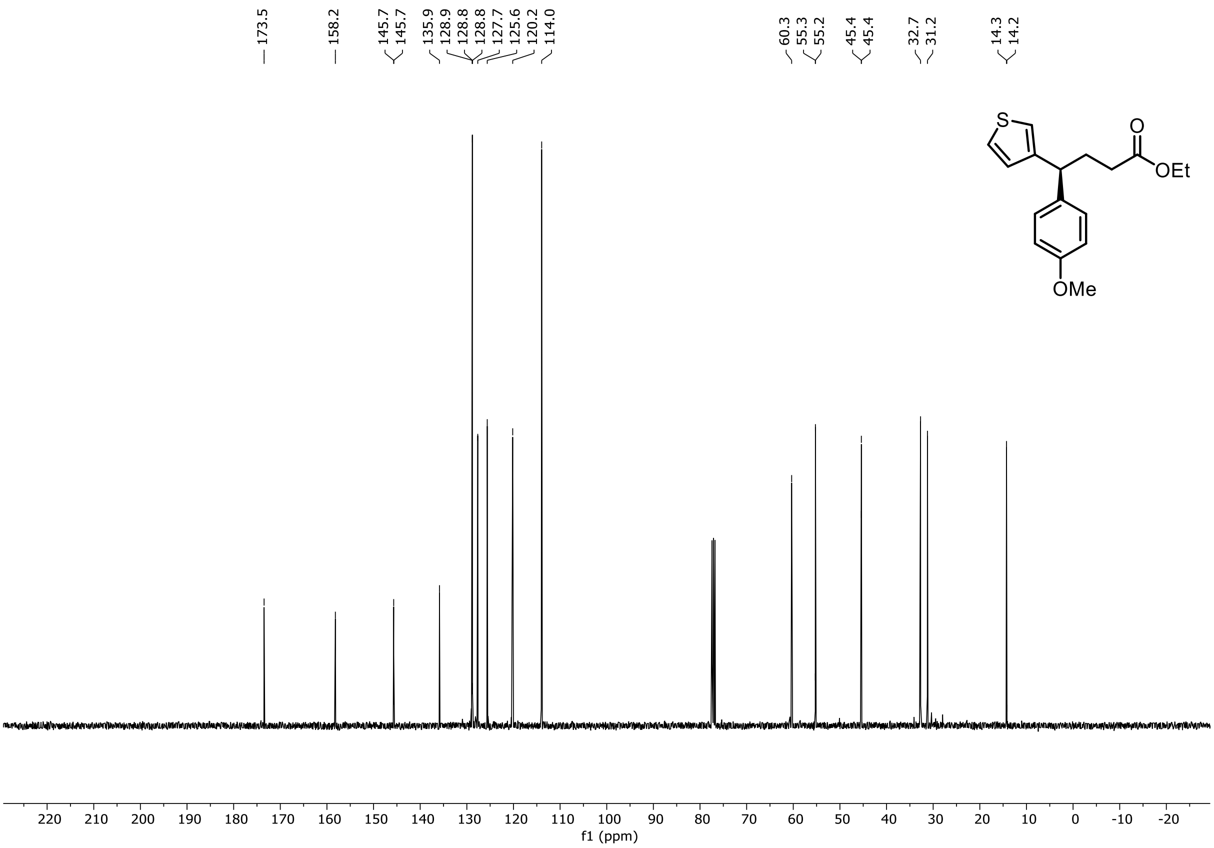

Figure 10.82 (top) ^{1}H NMR (400 MHz) and (bottom) ^{13}C NMR (101 MHz) spectra of *red*-**5o**.

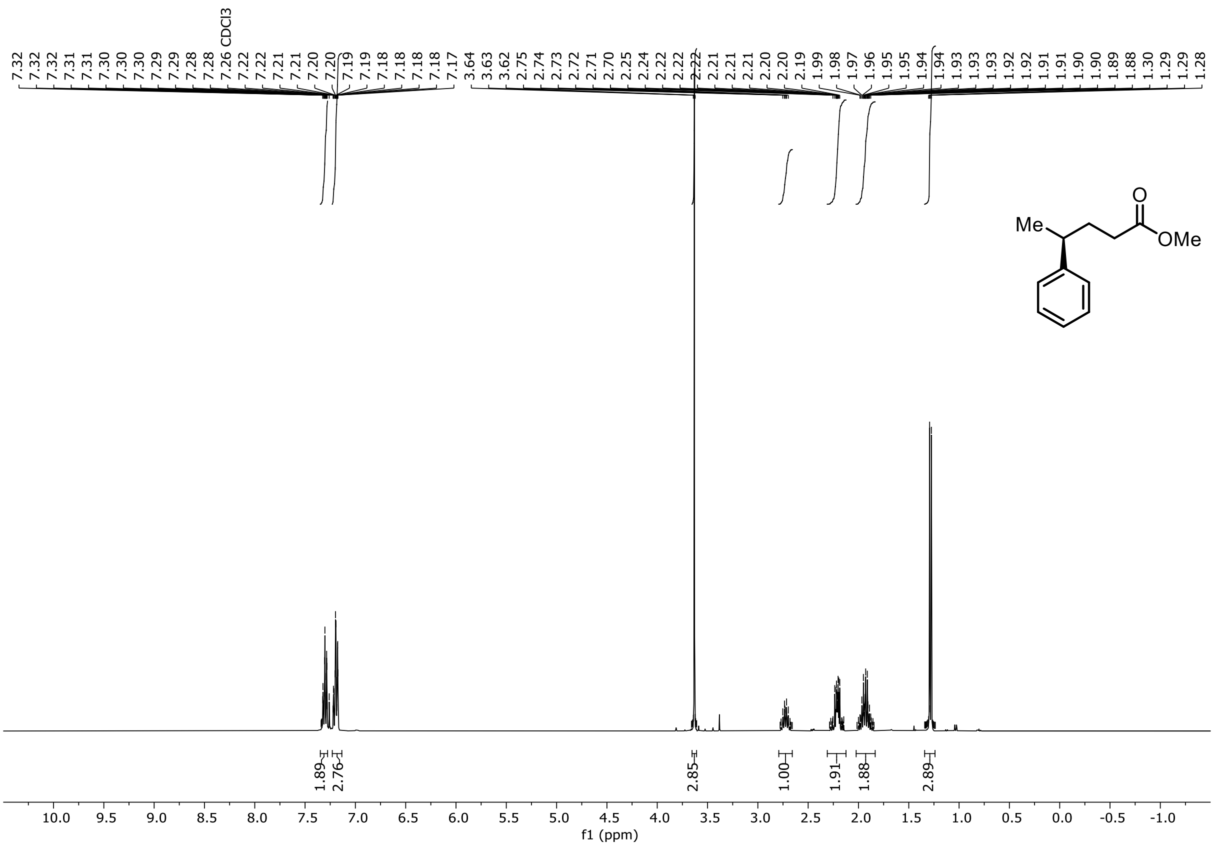

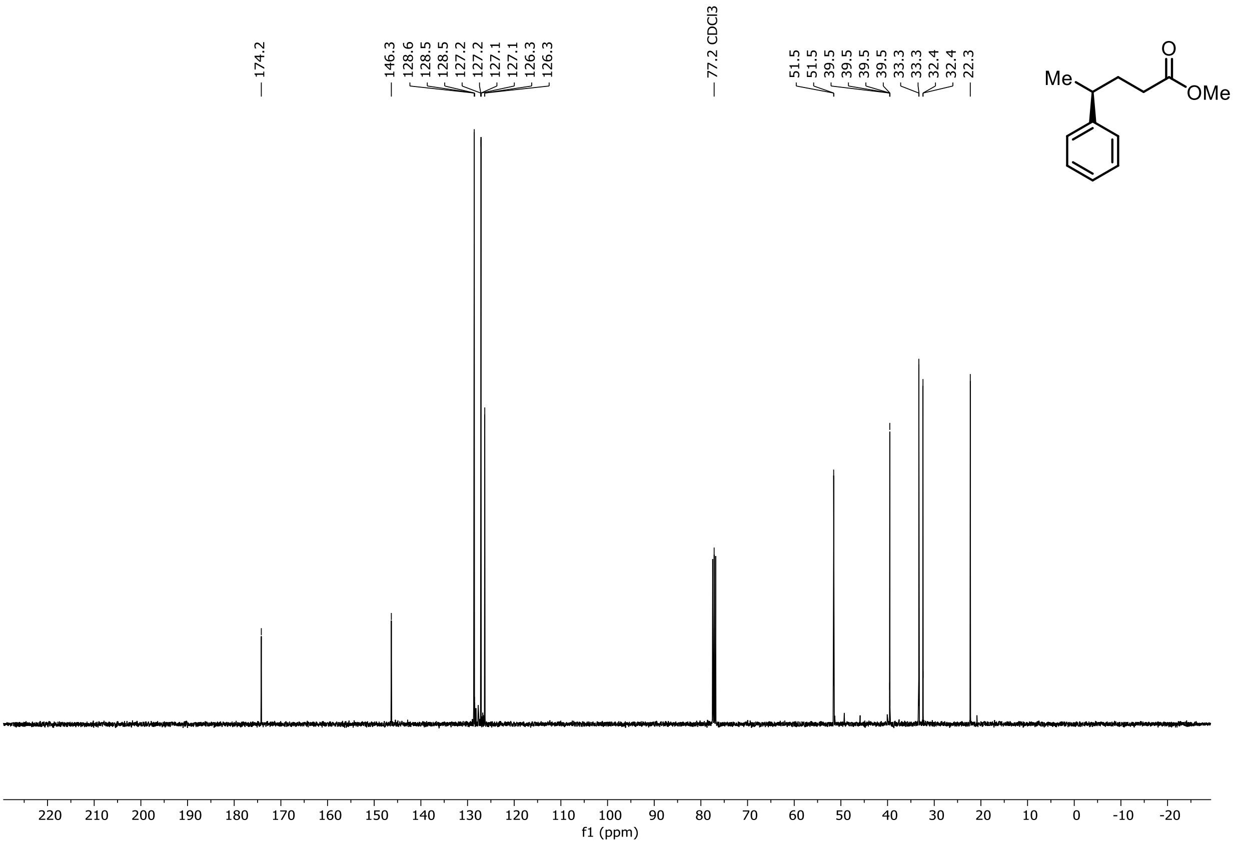

Figure 10.83 (top) ^{1}H NMR (400 MHz) and (bottom) ^{13}C NMR (101 MHz) spectra of *red*-**5p**.

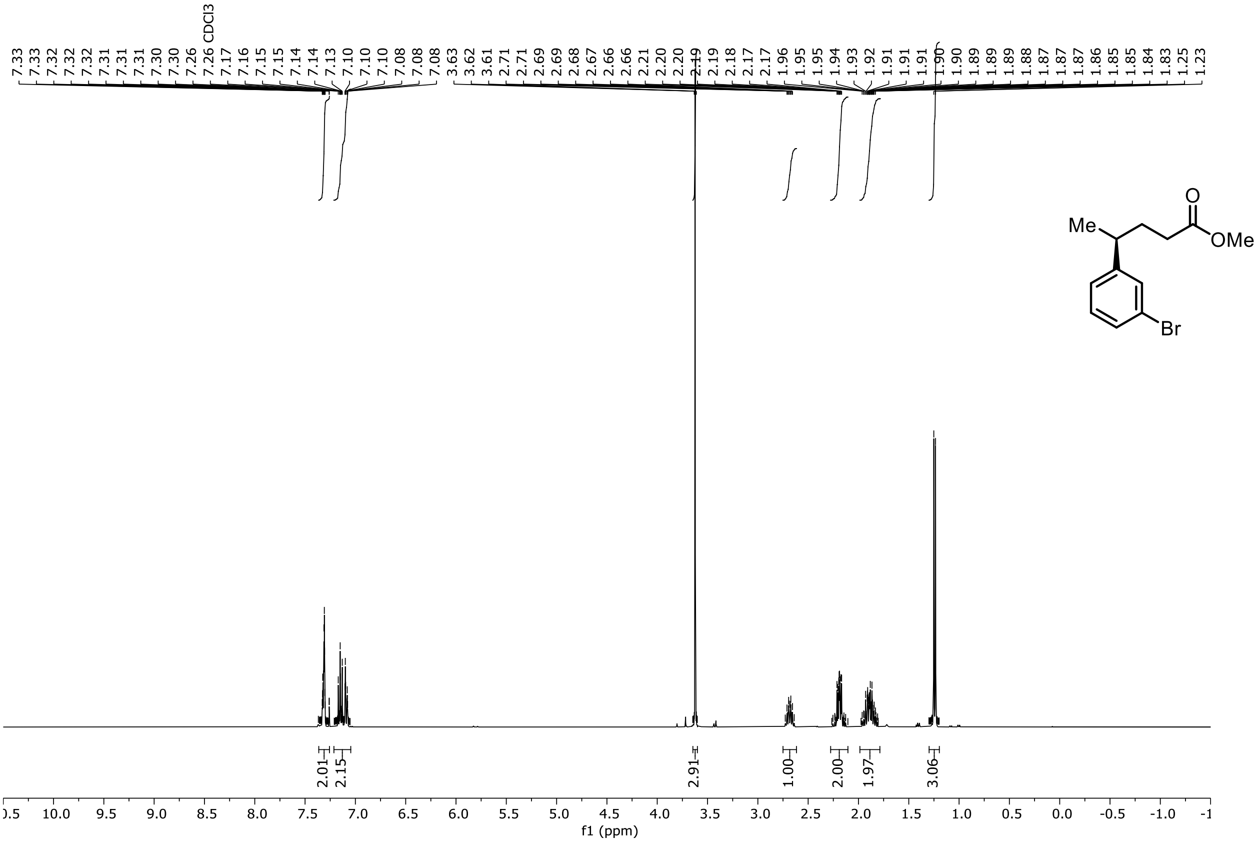

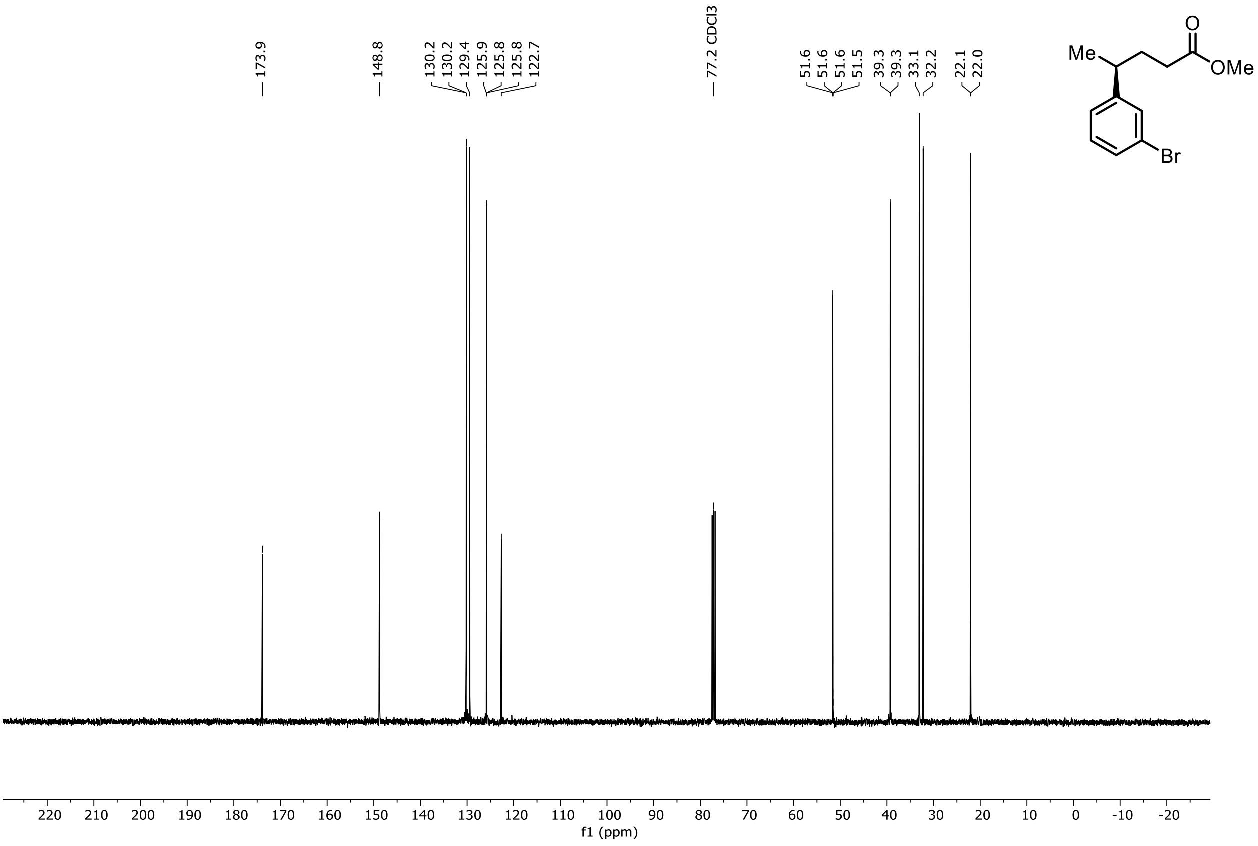

Figure 10.84 (top) ^{1}H NMR (400 MHz) and (bottom) ^{13}C NMR (101 MHz) spectra of *red*-**5q**.

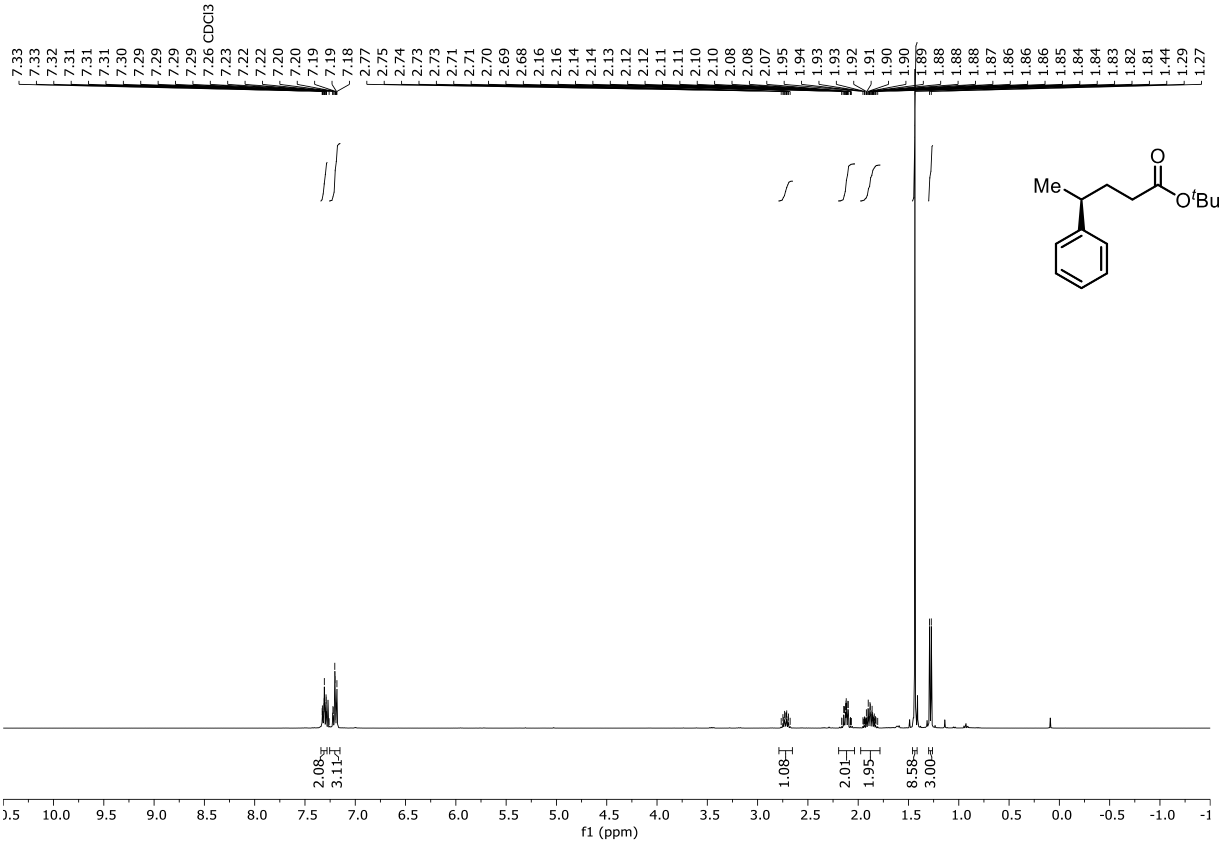

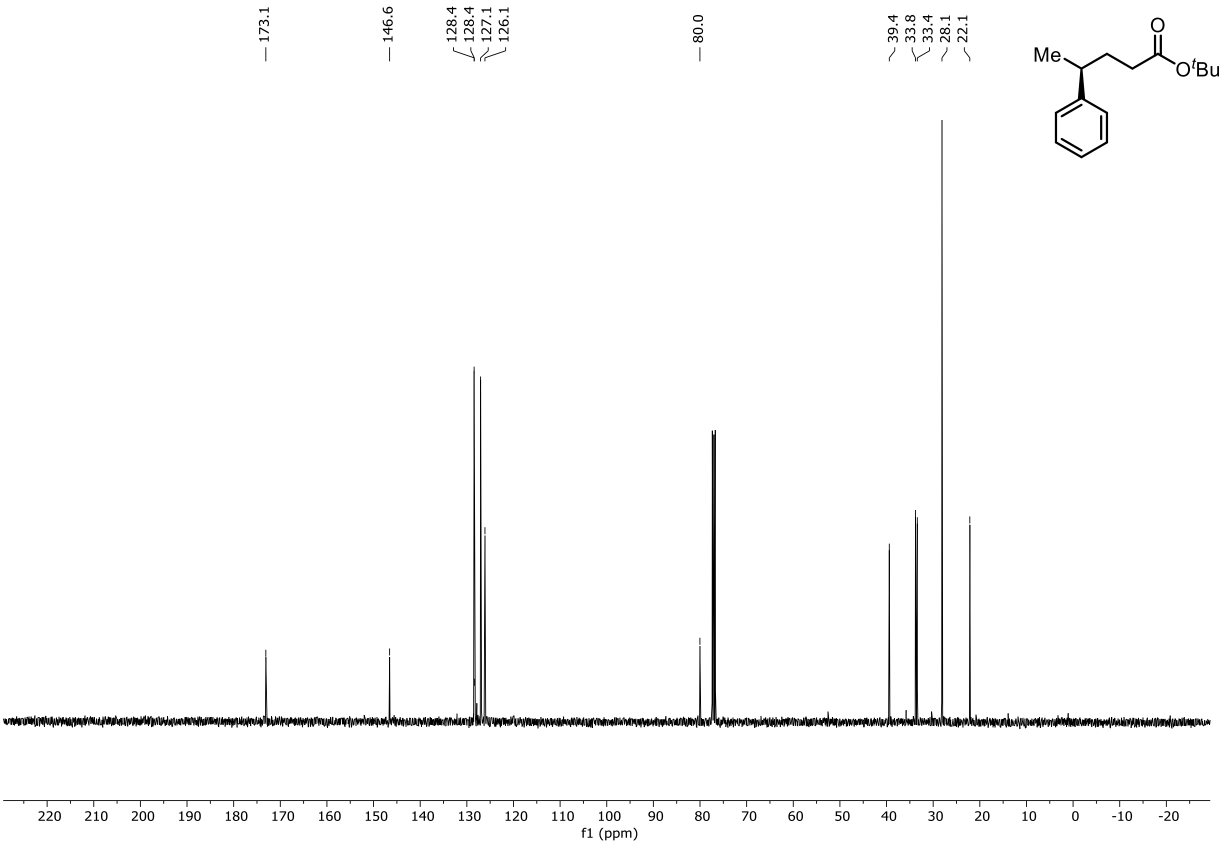

Figure 10.85 (top) ^{1}H NMR (400 MHz) and (bottom) ^{13}C NMR (101 MHz) spectra of *red*-**5r**.

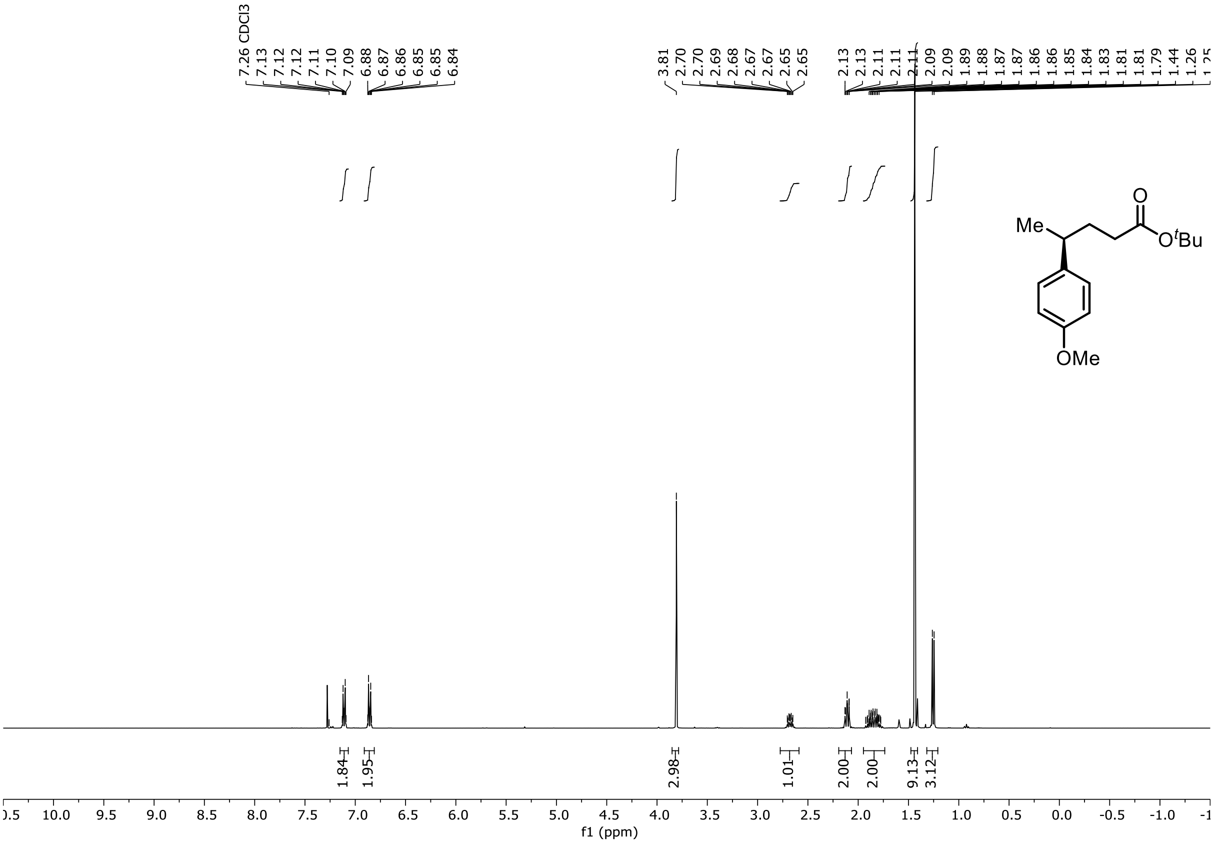

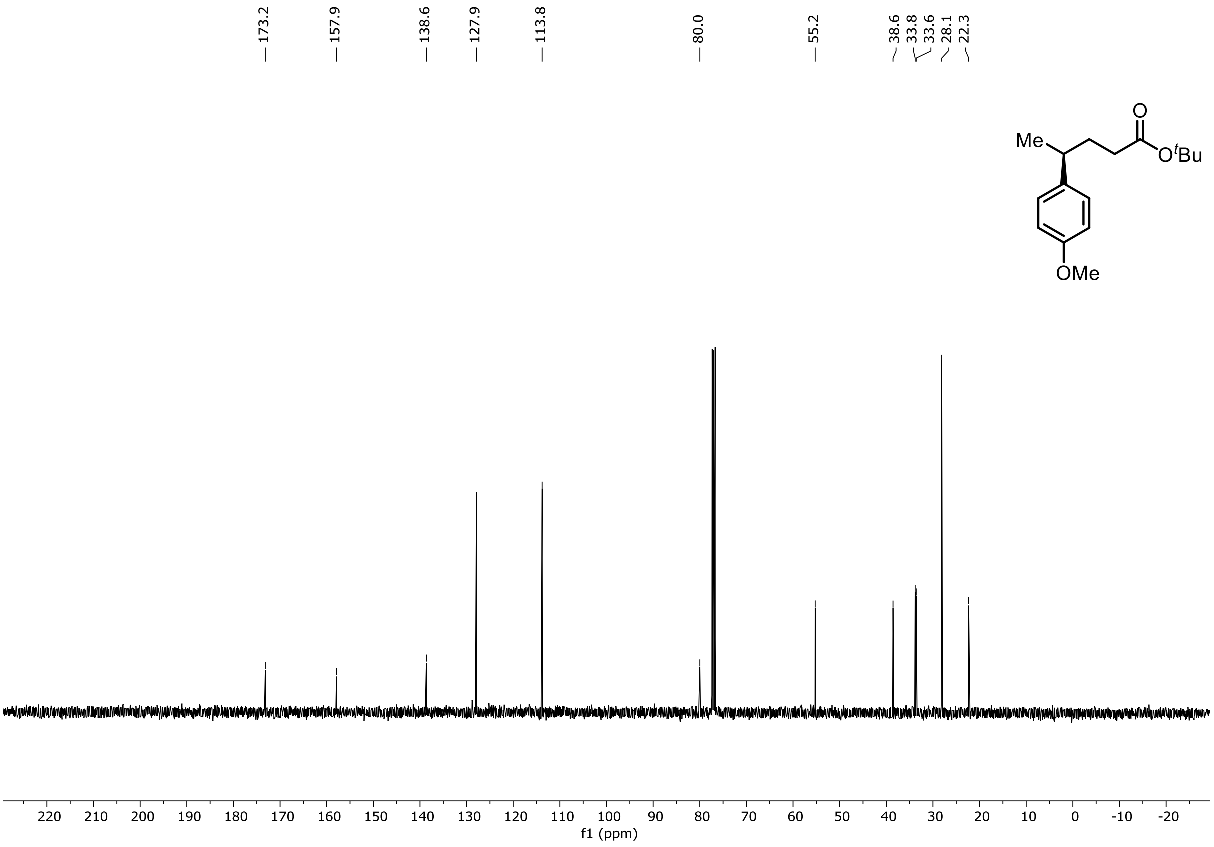

Figure 10.86 (top) ^{1}H NMR (400 MHz) and (bottom) ^{13}C NMR (101 MHz) spectra of *red*-**5s**.

10.3. Spectra of the derivatization products

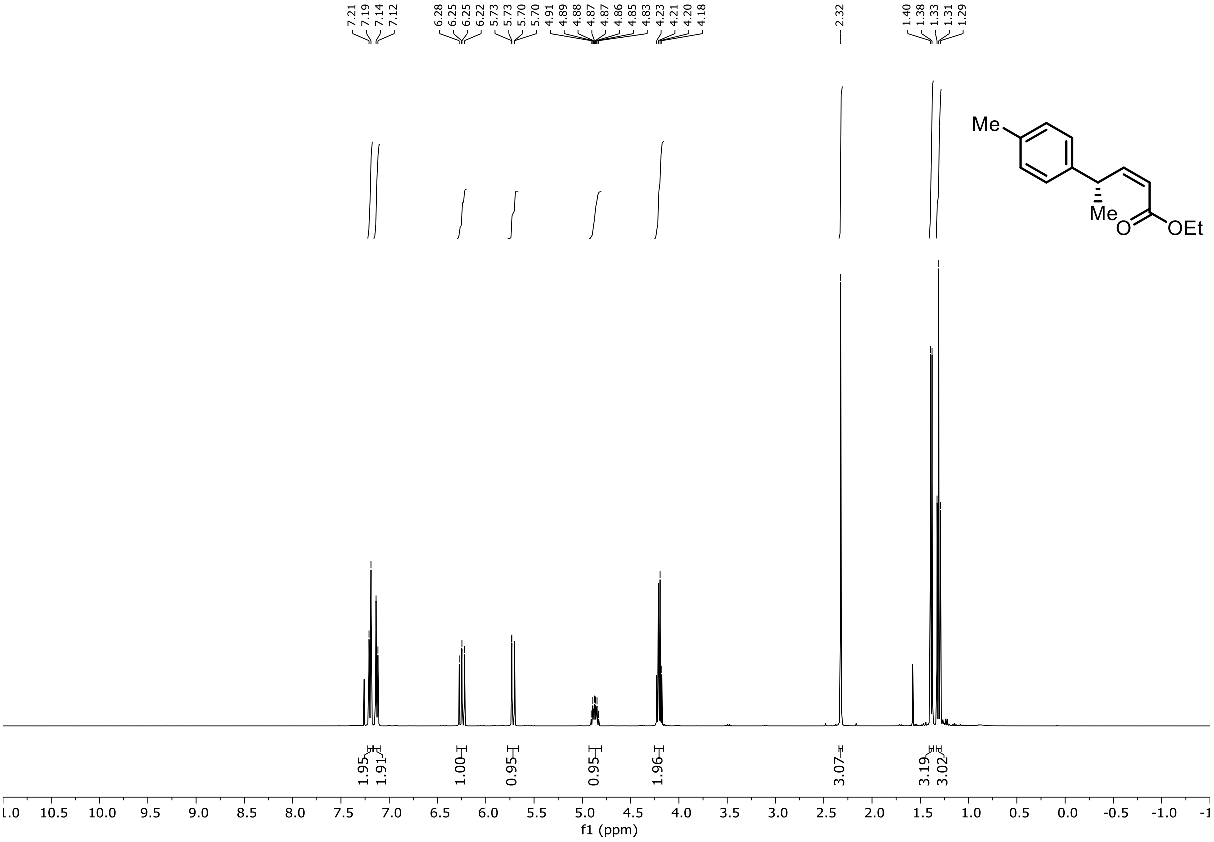

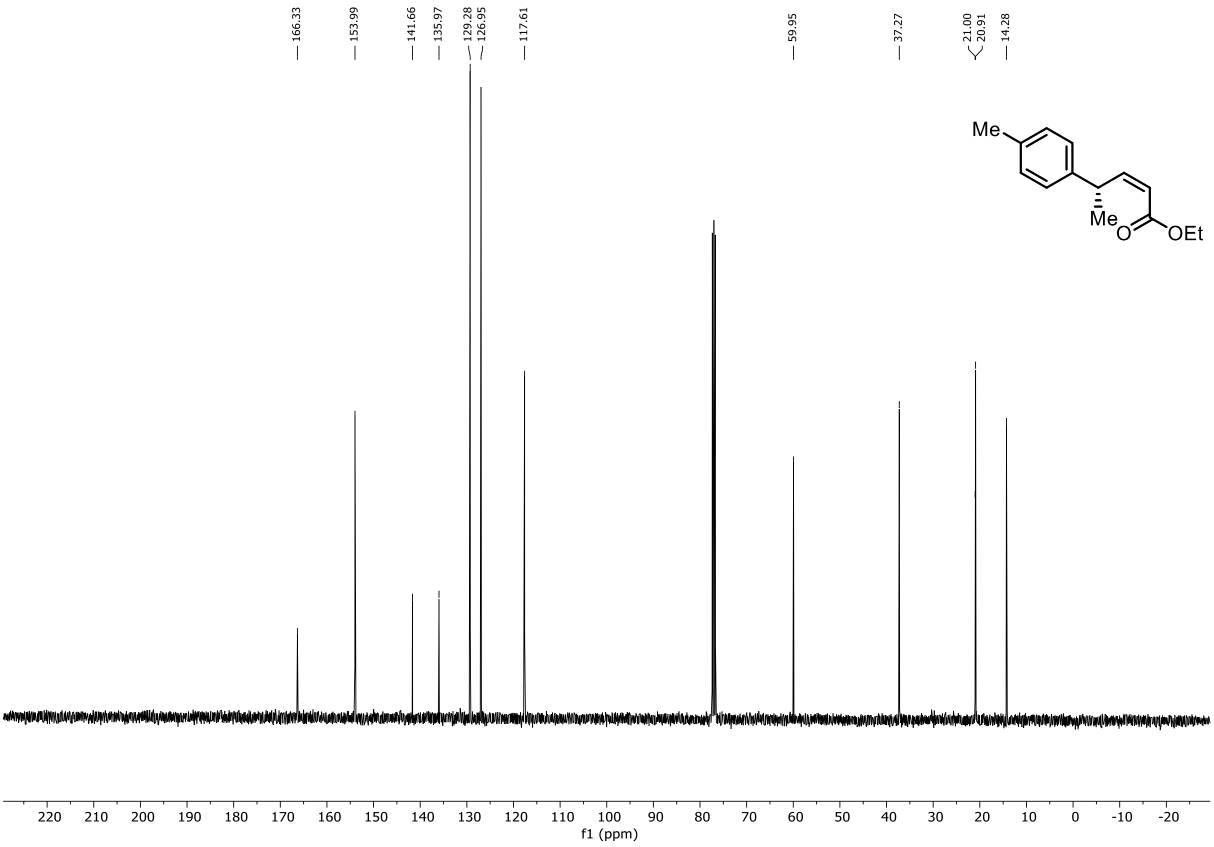

Figure 10.87 (top) ^{1}H NMR (400 MHz) and (bottom) ^{13}C NMR (101 MHz) spectra of *Z*-**3d**.

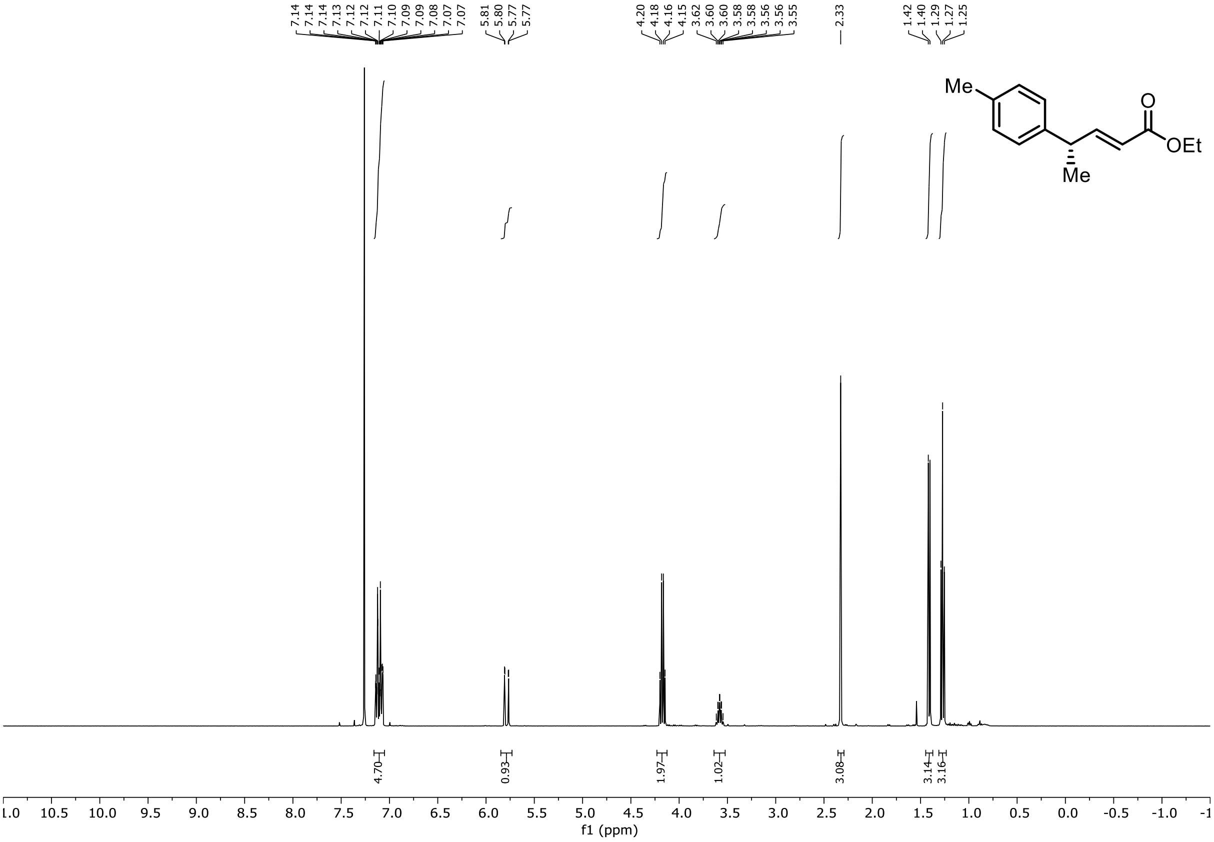

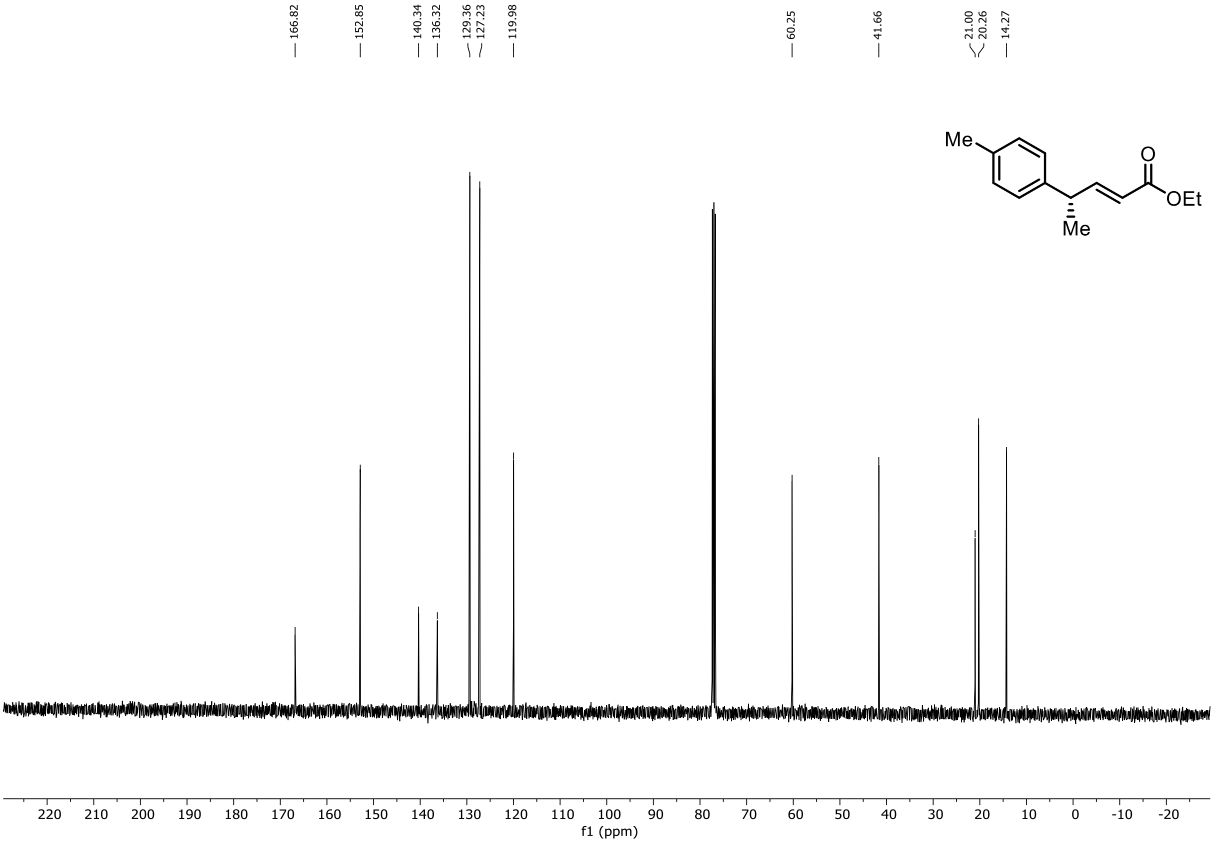

Figure 10.88 (top) ^{1}H NMR (400 MHz) and (bottom) ^{13}C NMR (101 MHz) spectra of *E*-**3d**.

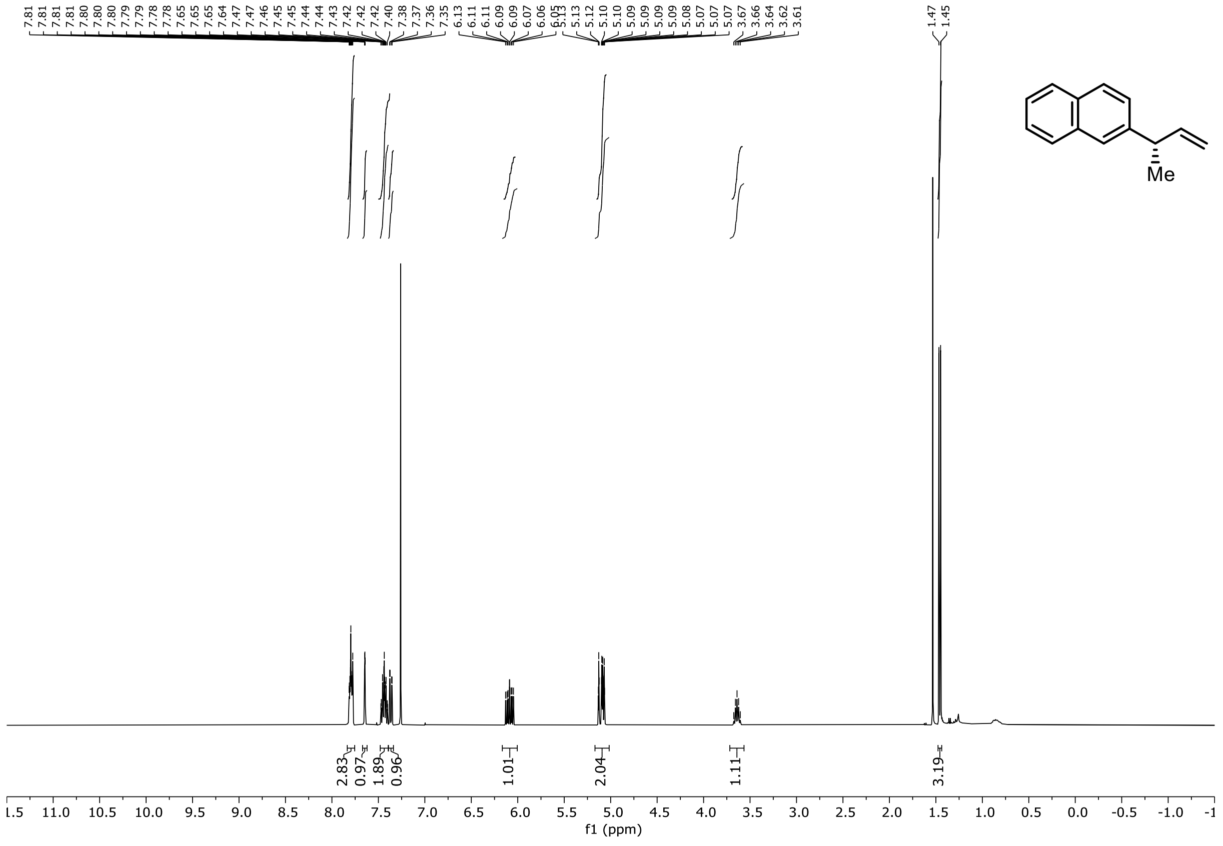

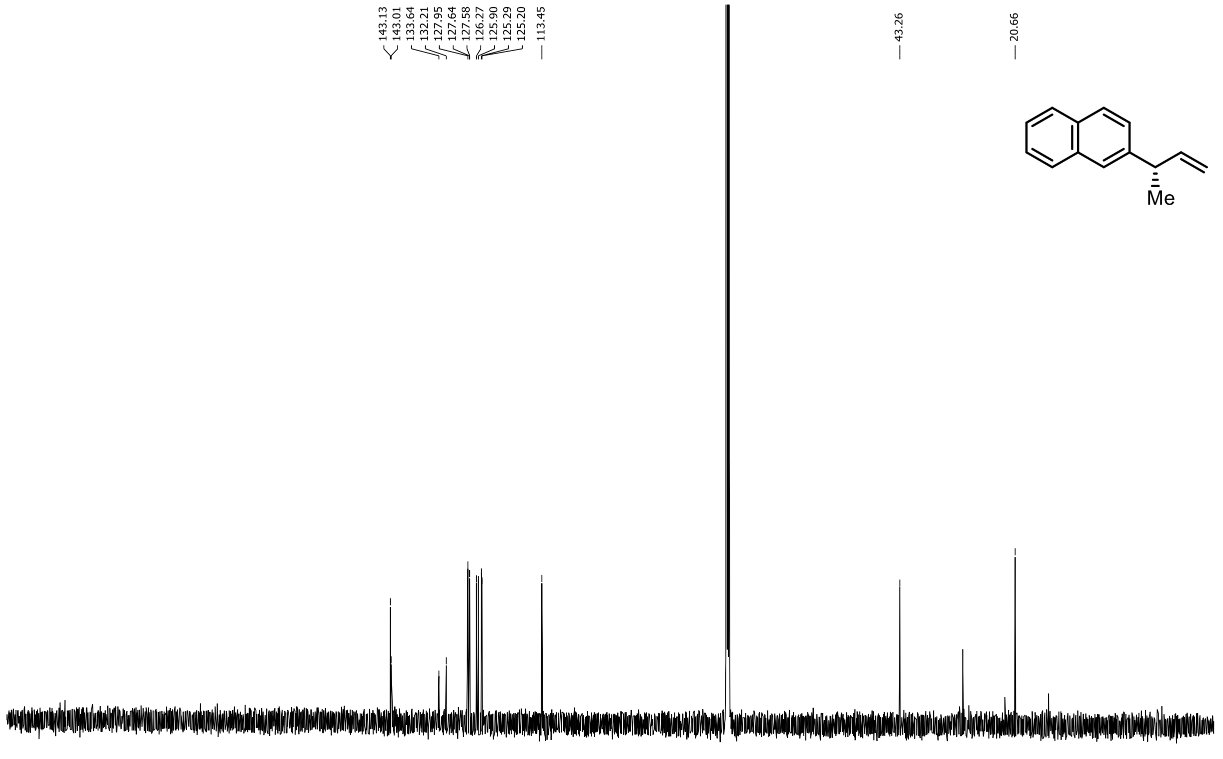

Figure 10.89 (top) ^{1}H NMR (400 MHz) and (bottom) ^{13}C NMR (101 MHz) spectra of **6**.

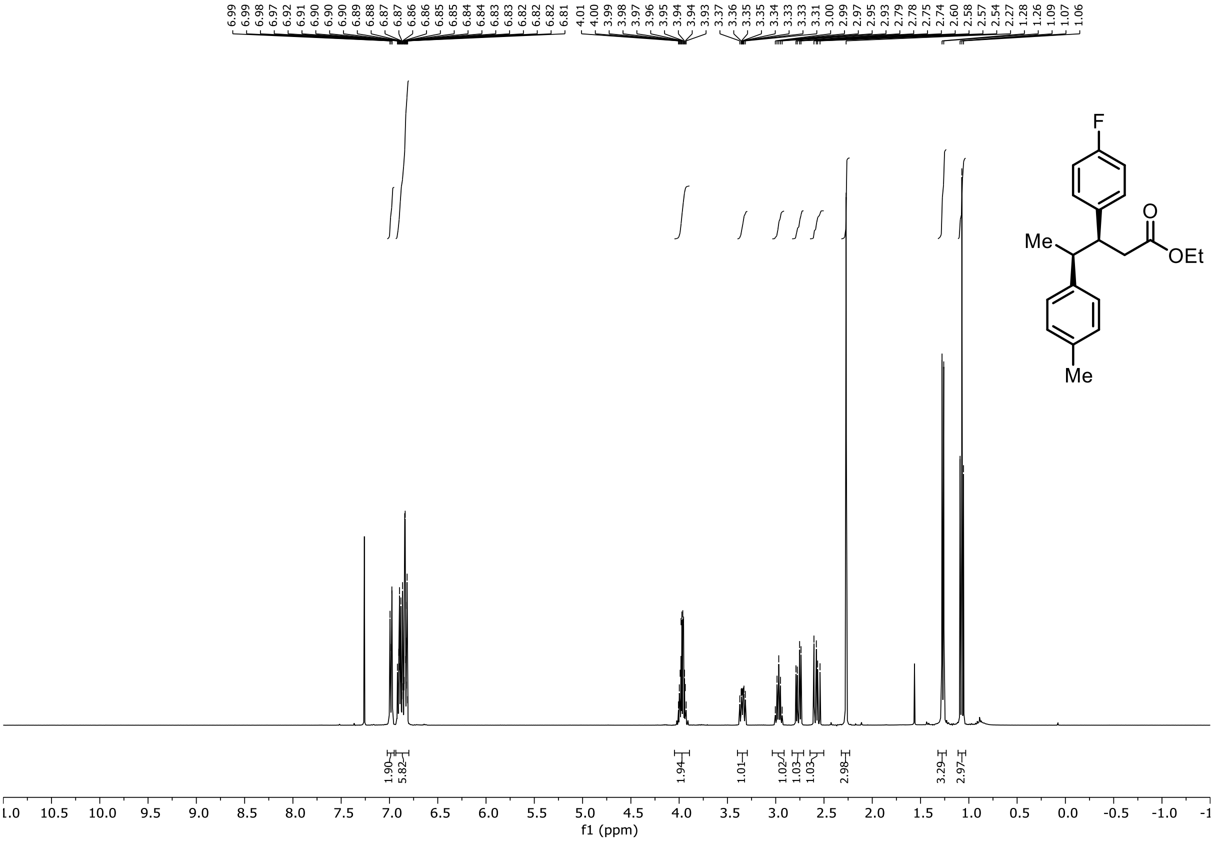

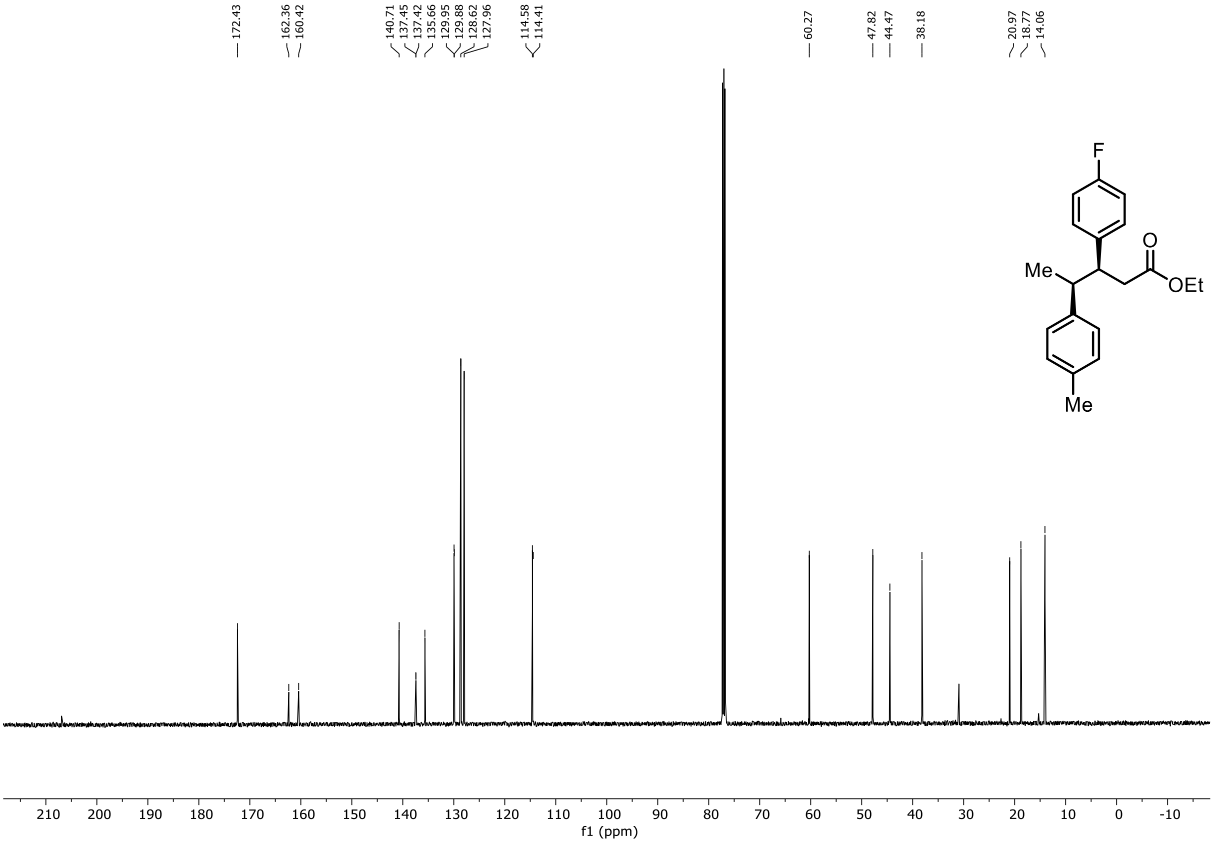

Figure 10.90 (top) ^{1}H NMR (400 MHz) and (bottom) ^{13}C NMR (101 MHz) spectra of **7**.

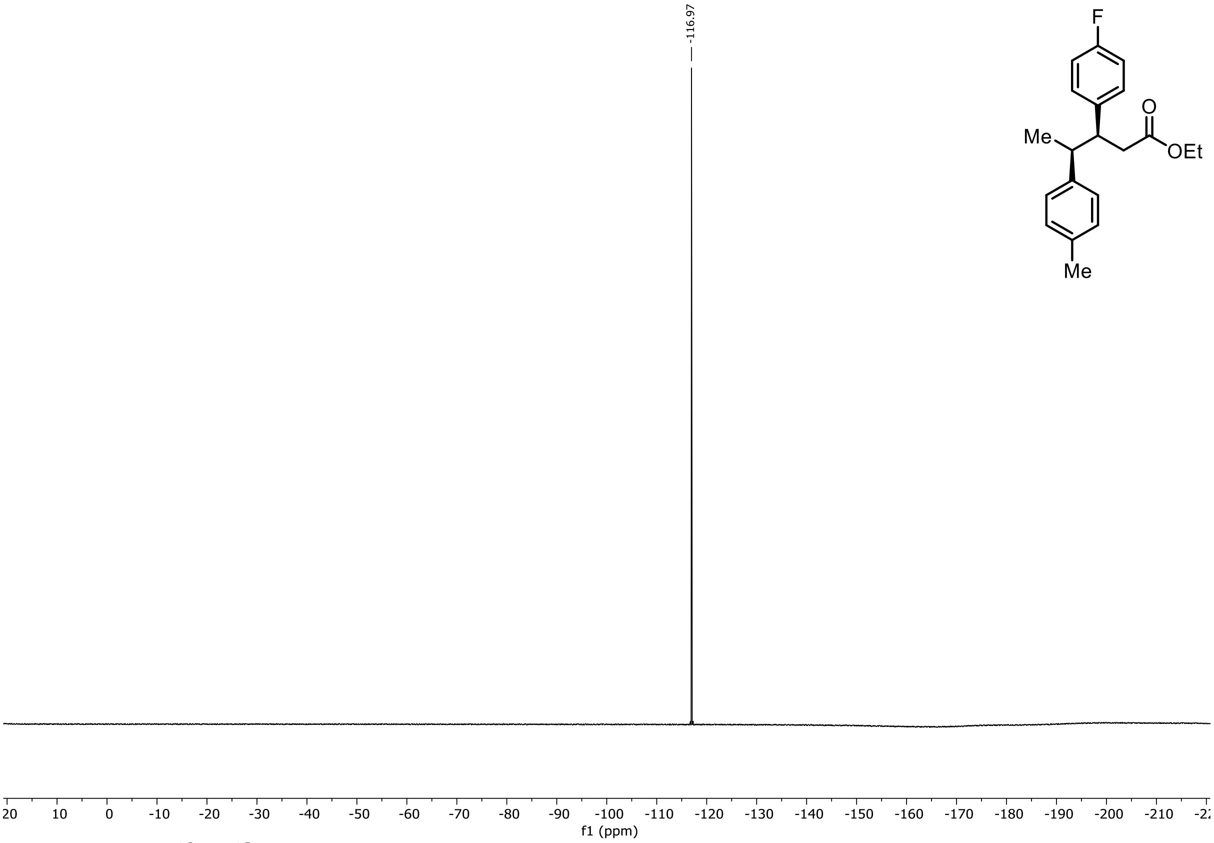

Figure 10.91 ^{19}F (^{13}C)NMR (470 MHz) spectrum of **7**.

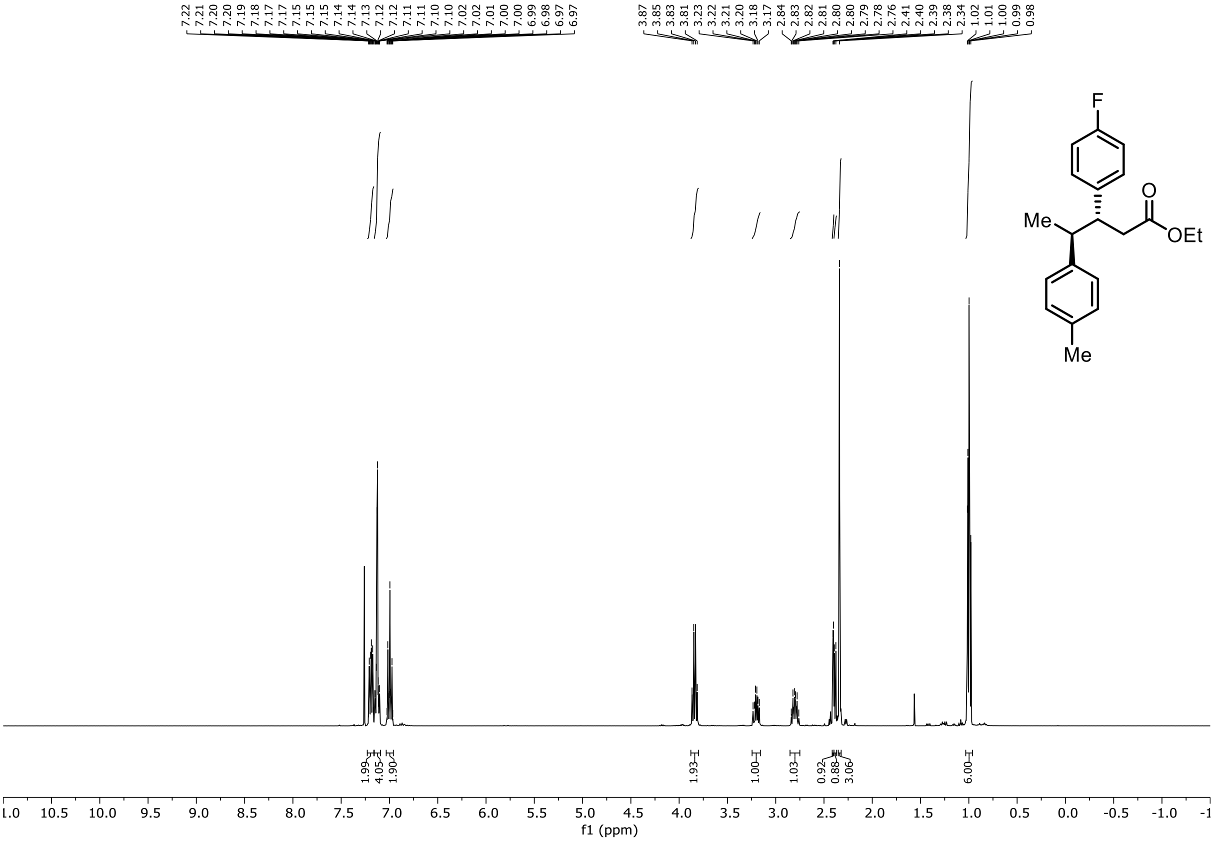

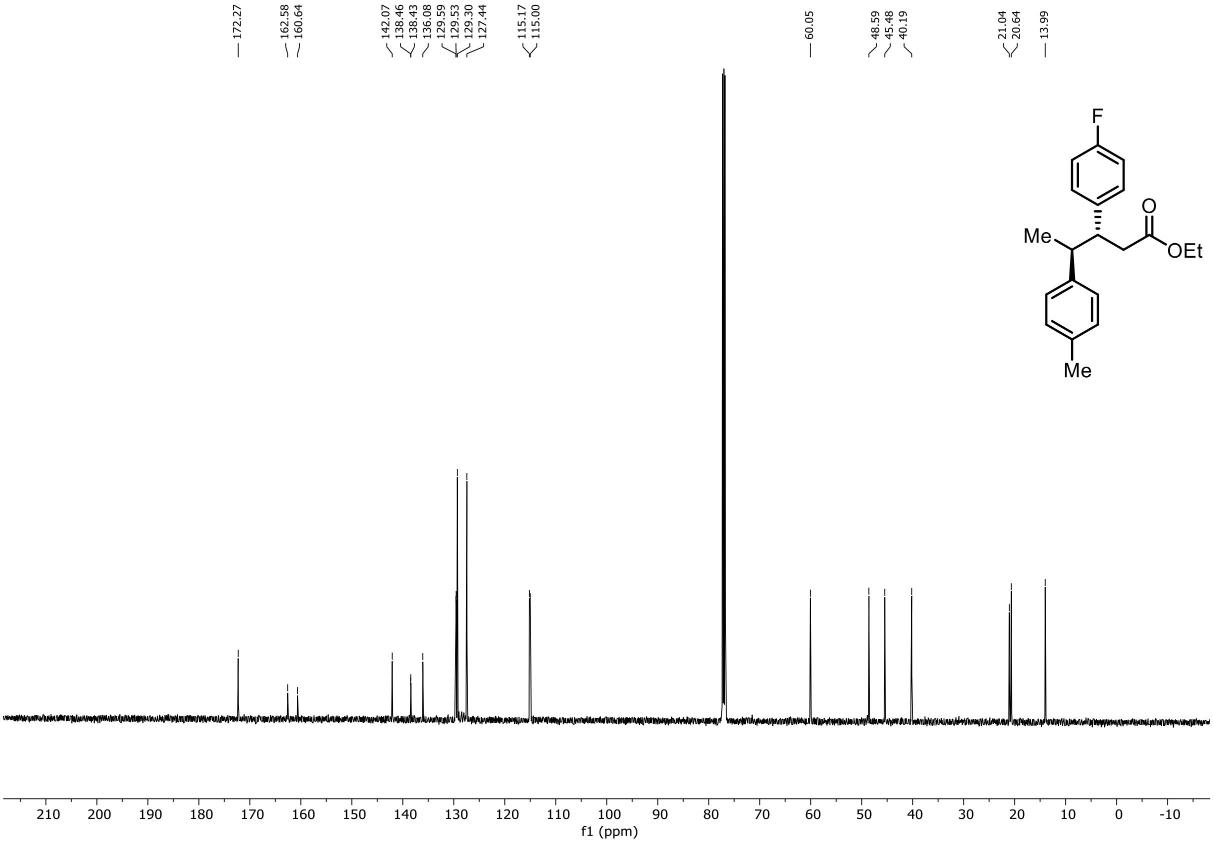

Figure 10.92 (top) ^{1}H NMR (400 MHz) and (bottom) ^{13}C NMR (101 MHz) spectra of **8**.

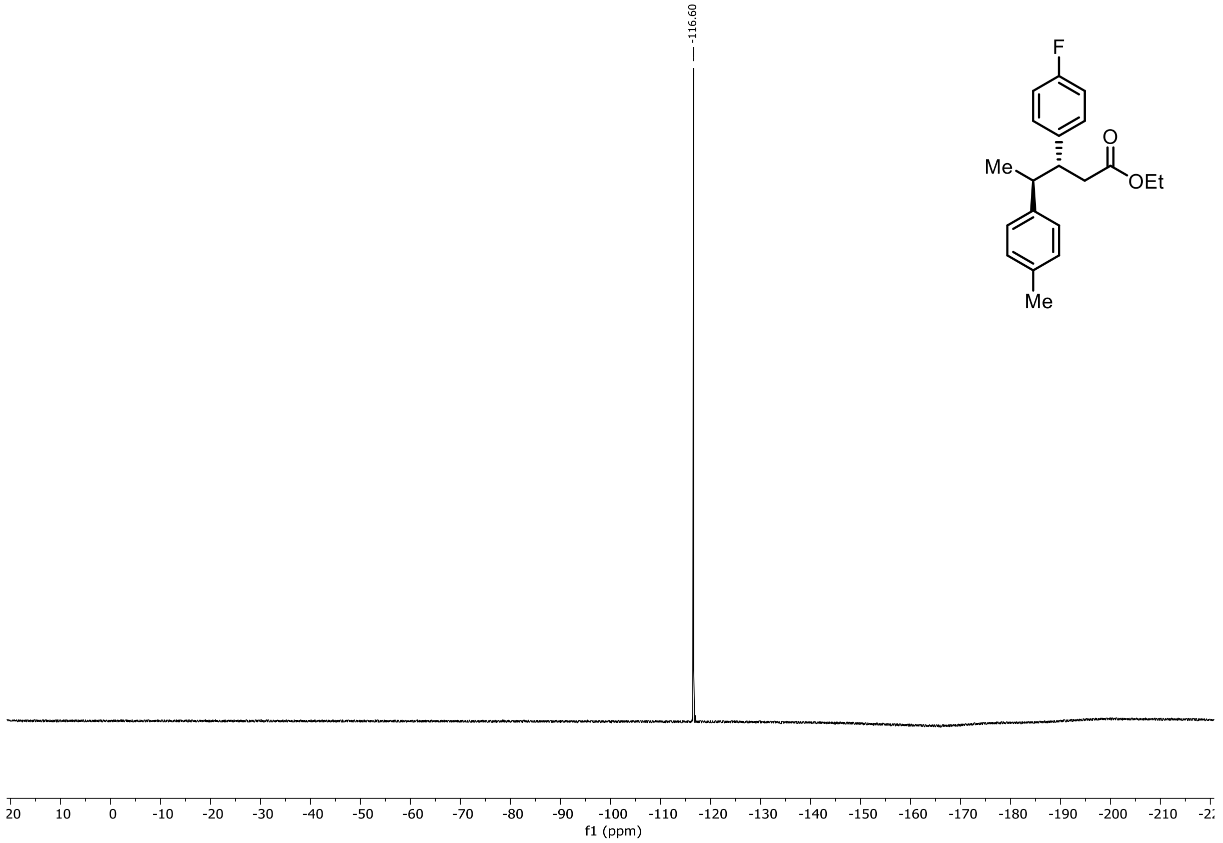

Figure 10.93 ^{19}F (^{13}C)NMR (470 MHz) spectrum of **8**.

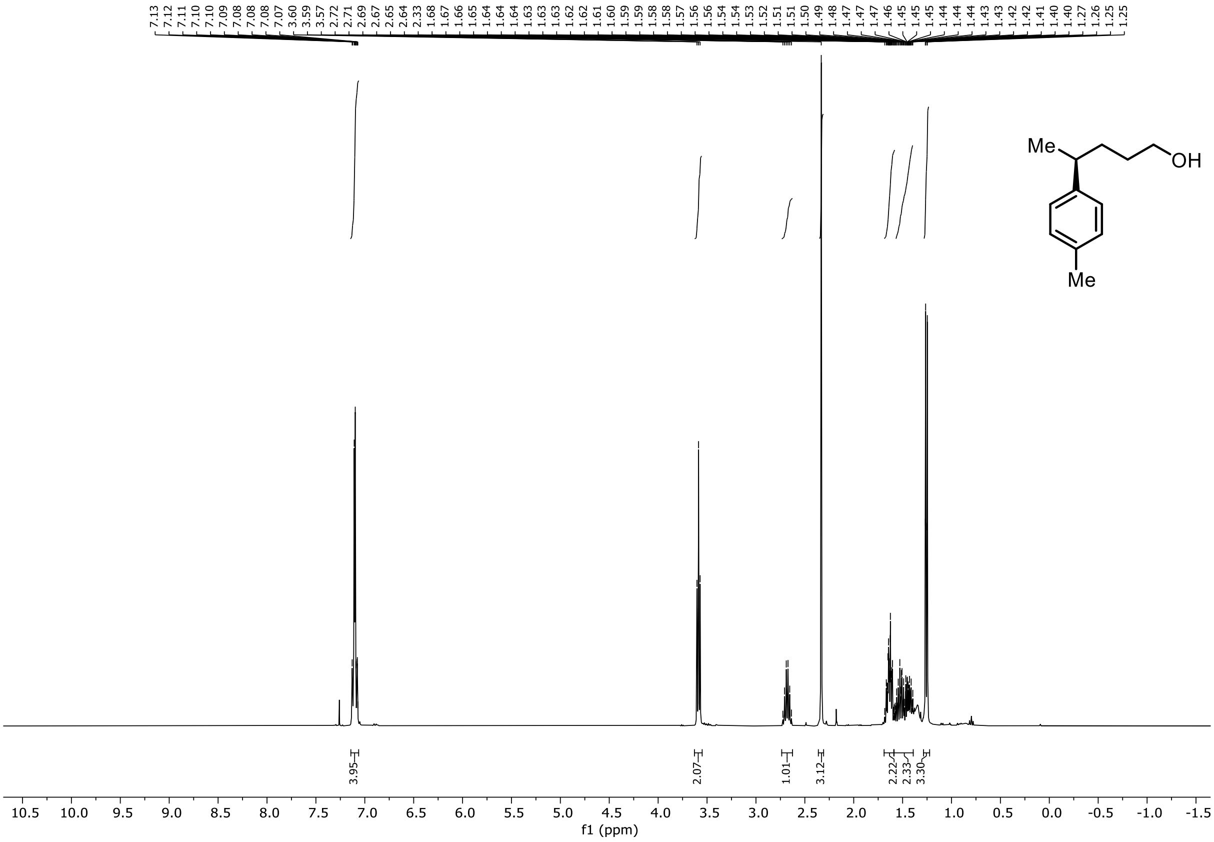

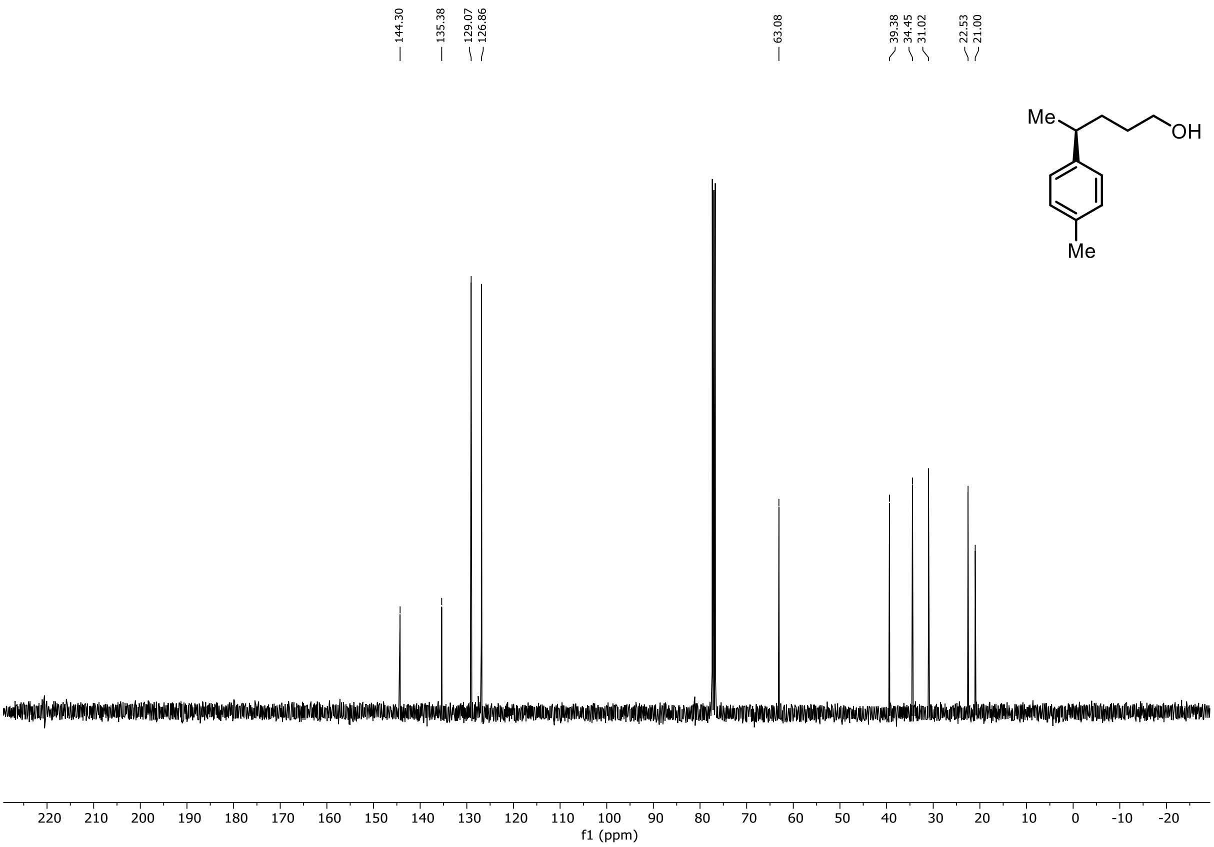

Figure 10.94 (top) ^{1}H NMR (400 MHz) and (bottom) ^{13}C NMR (101 MHz) spectra of **9**.

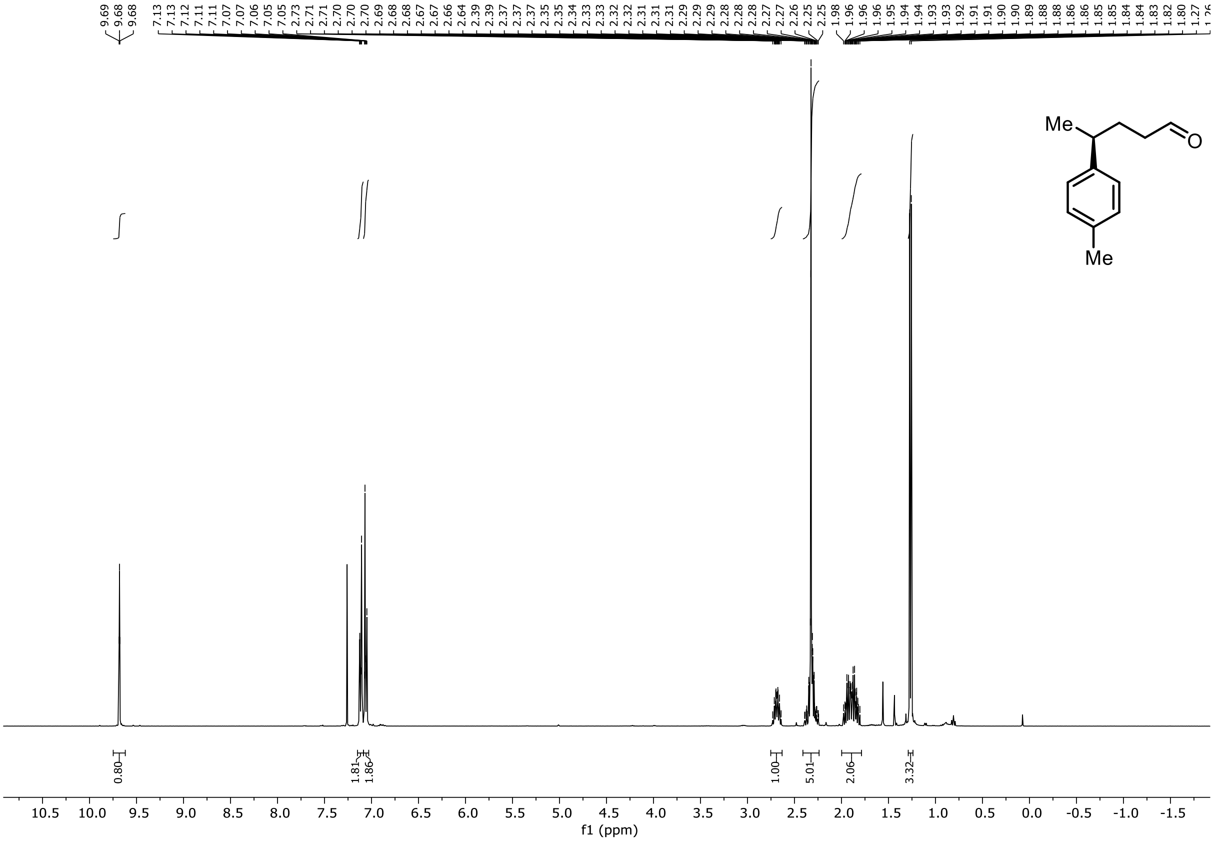

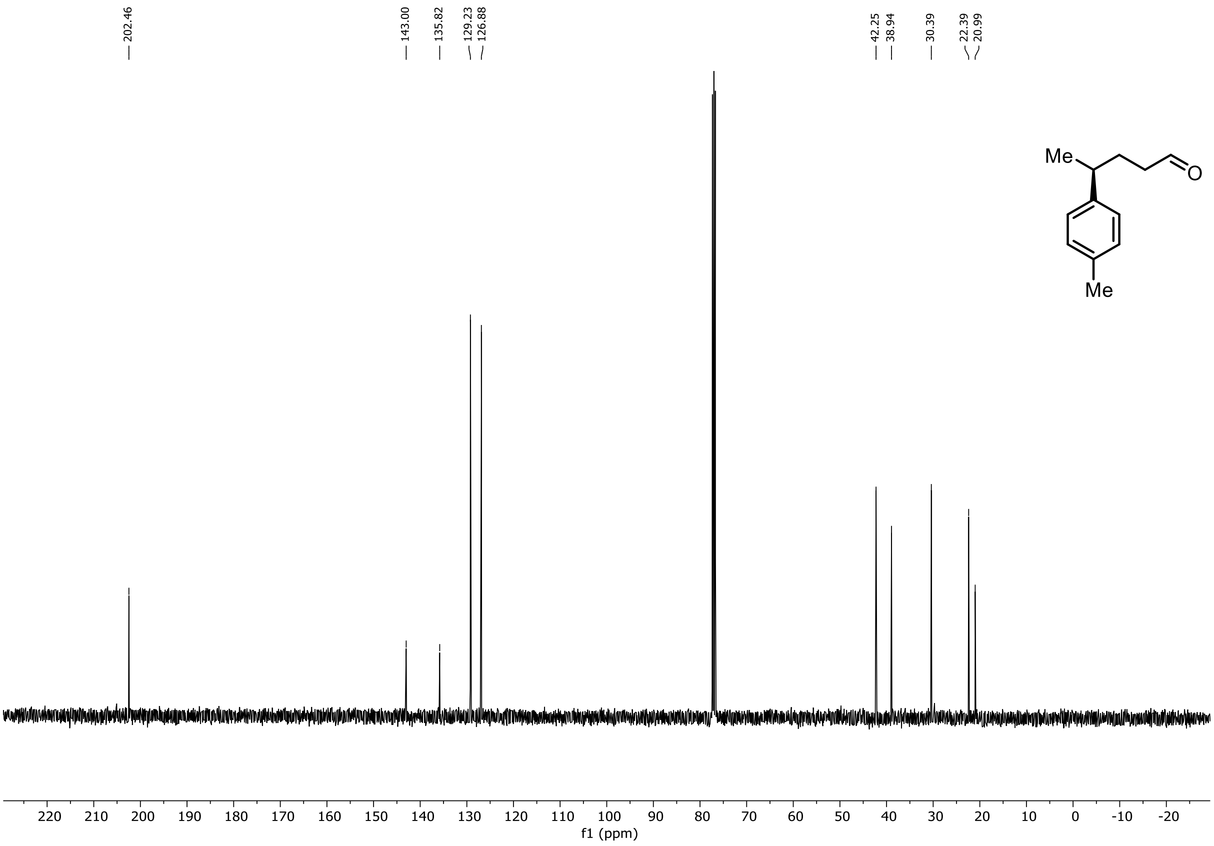

Figure 10.95 (top) ^{1}H NMR (400 MHz) and (bottom) ^{13}C NMR (101 MHz) spectra of **S20**.

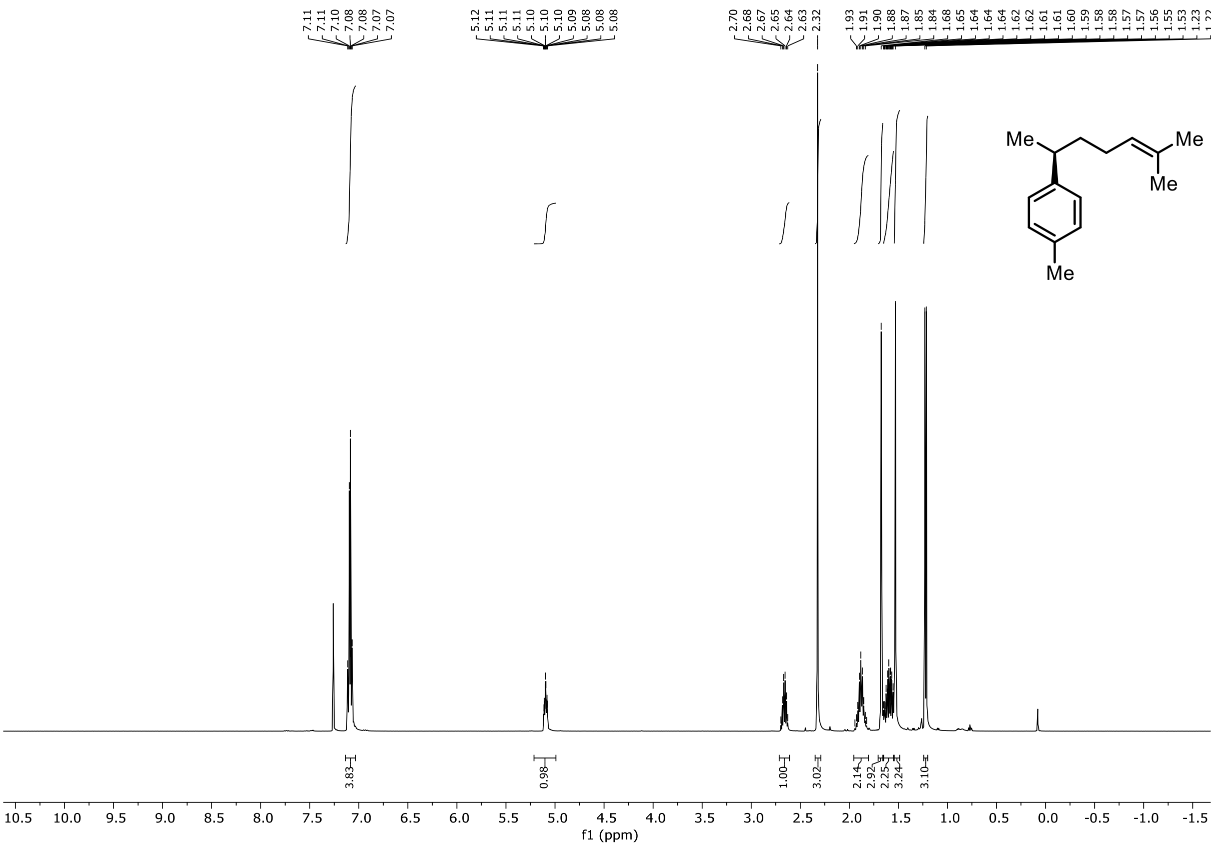

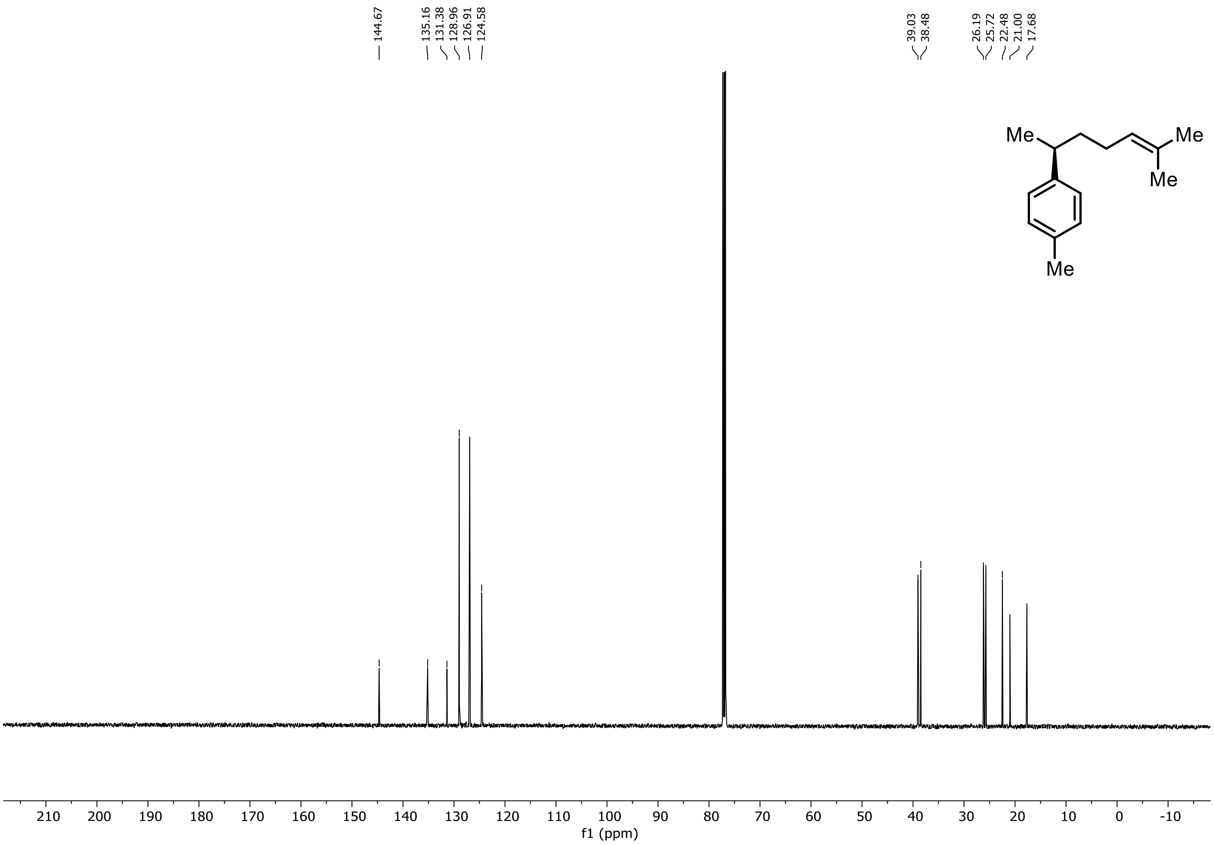

Figure 10.96 (top) ^{1}H NMR (400 MHz) and (bottom) ^{13}C NMR (101 MHz) spectra of **10**.

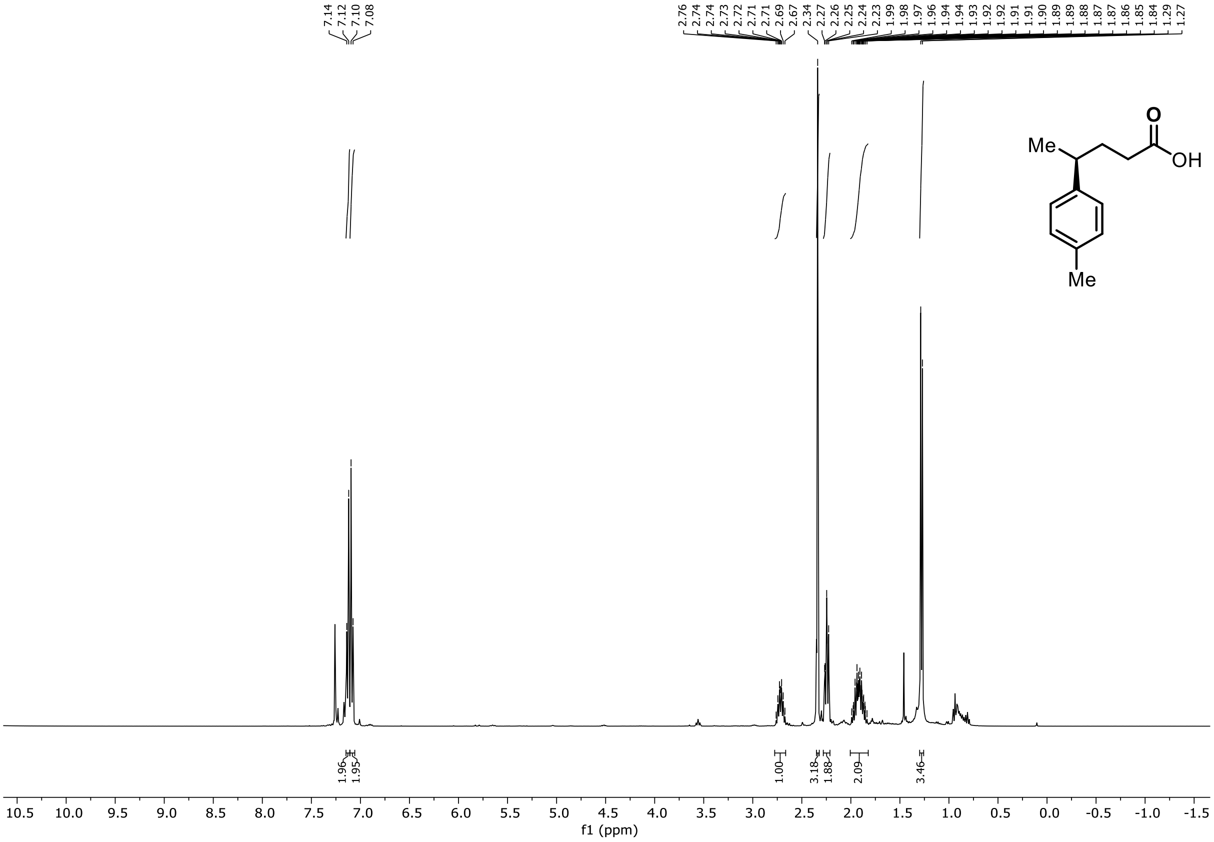

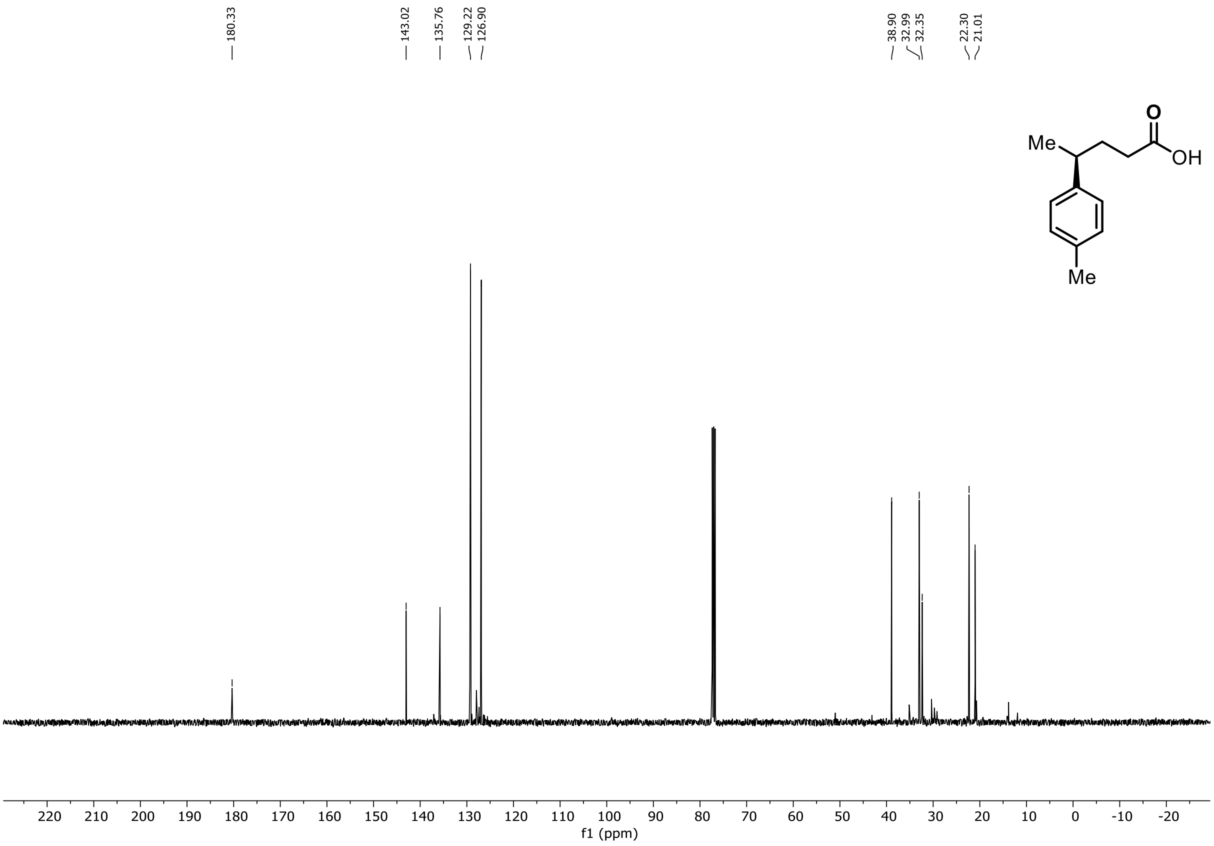

Figure 10.97 (top) ^{1}H NMR (400 MHz) and (bottom) ^{13}C NMR (101 MHz) spectra of **S21**.

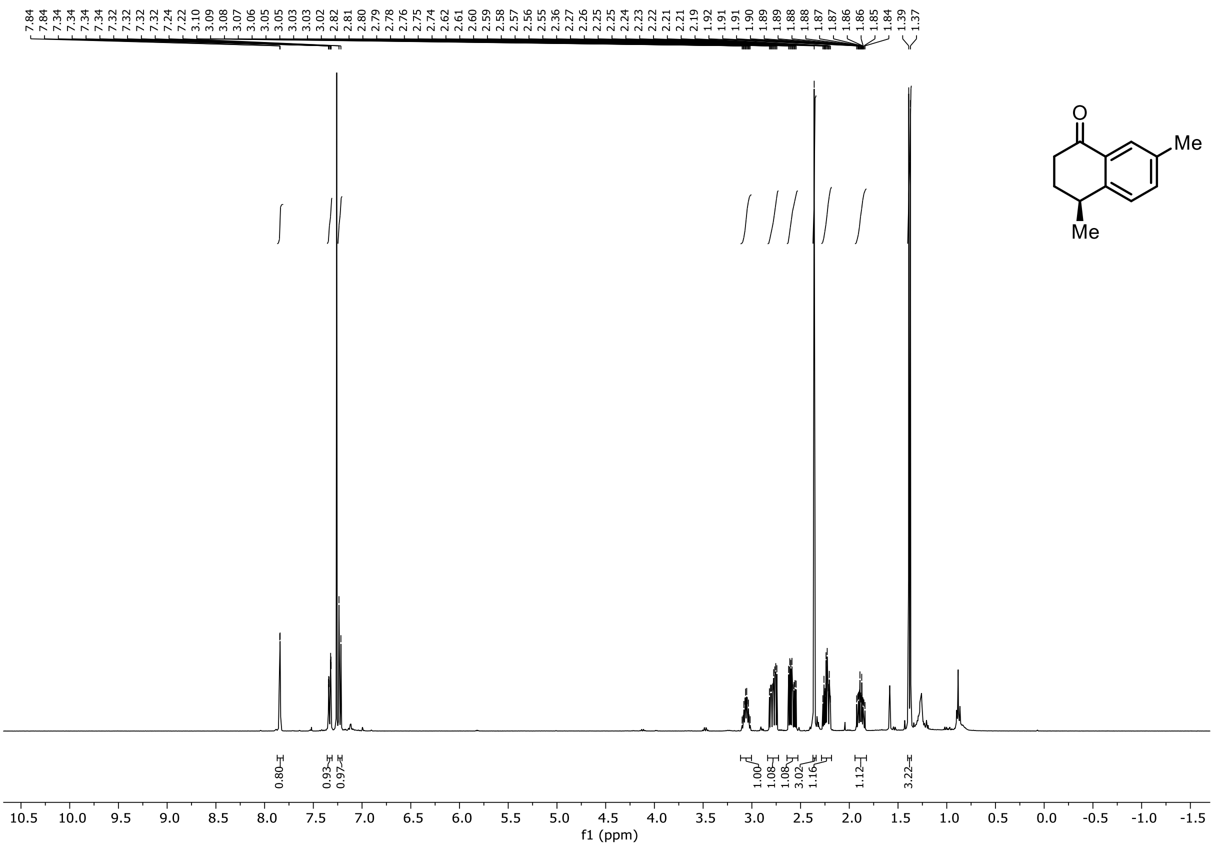

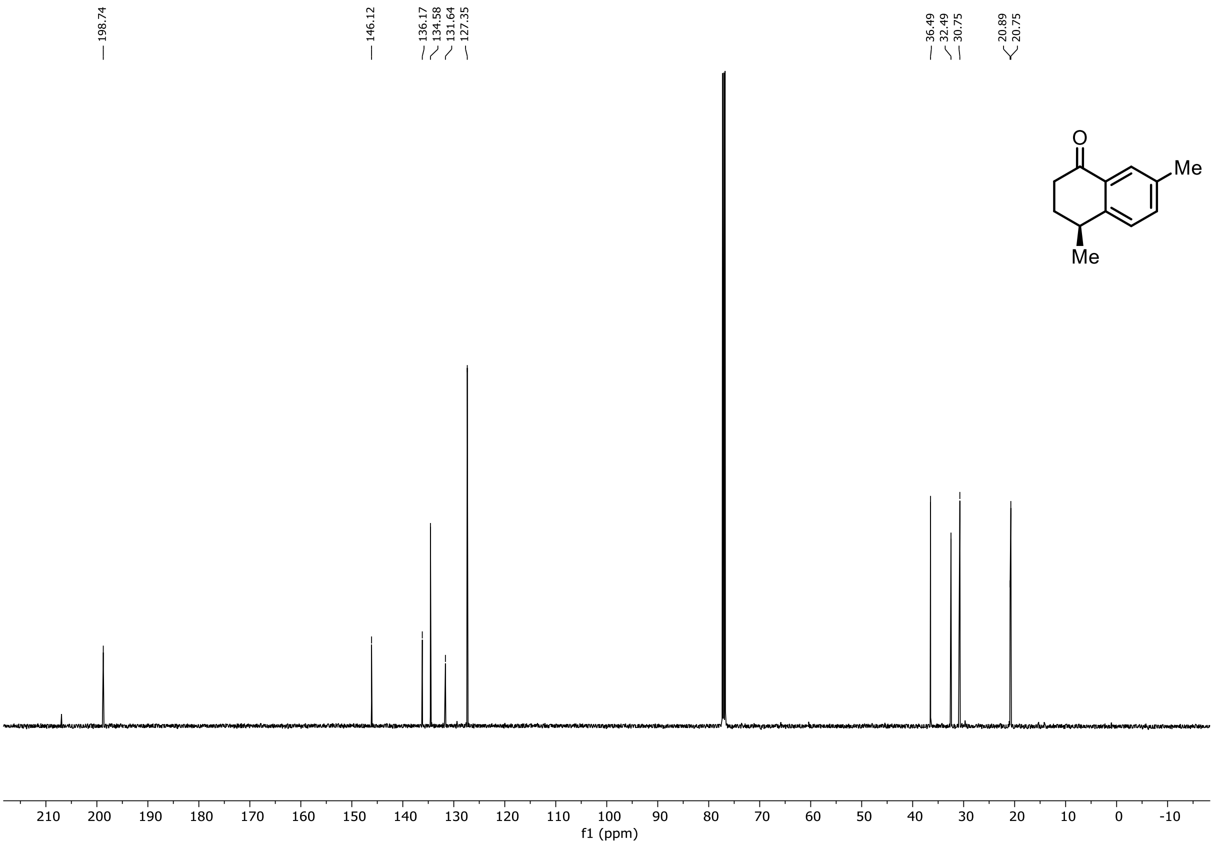

Figure 10.98 (top) ^{1}H NMR (400 MHz) and (bottom) ^{13}C NMR (101 MHz) spectra of **11**.

10.4. Spectra of the alternative starting materials

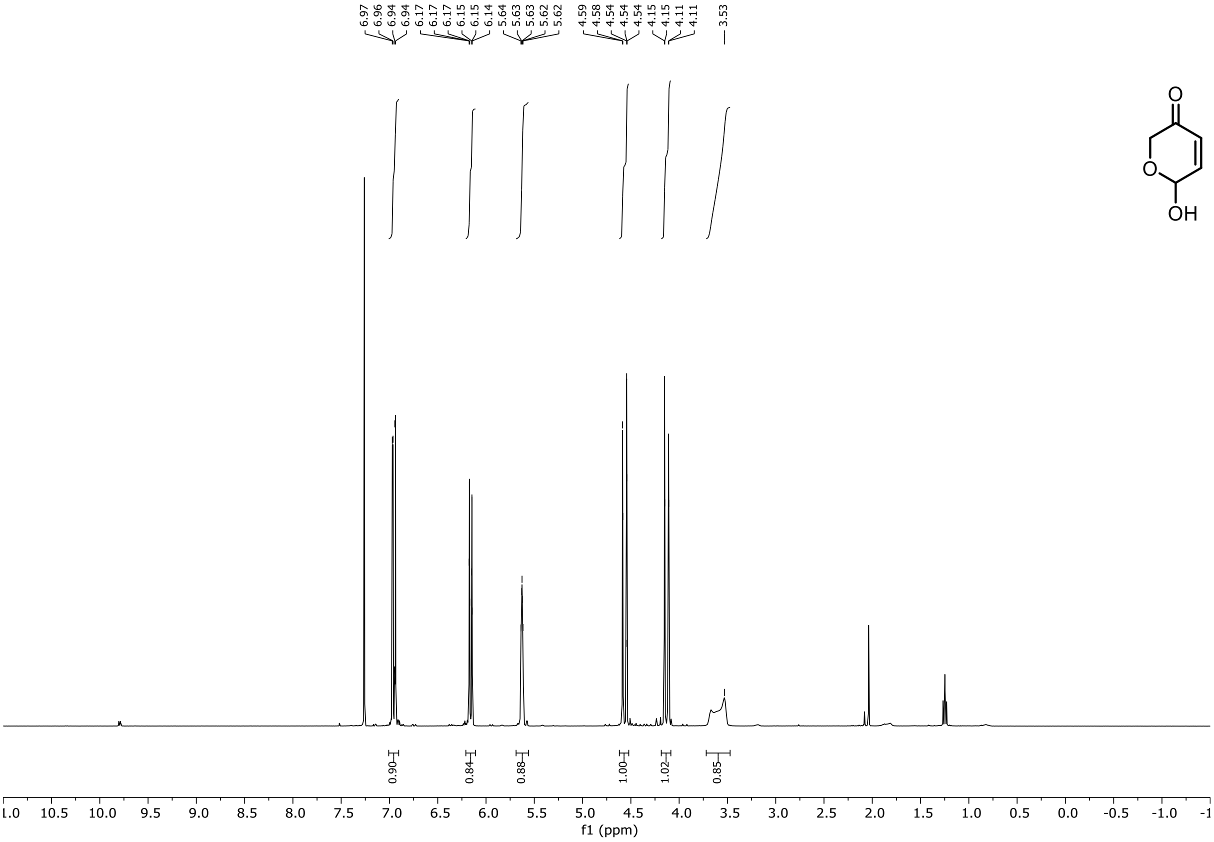

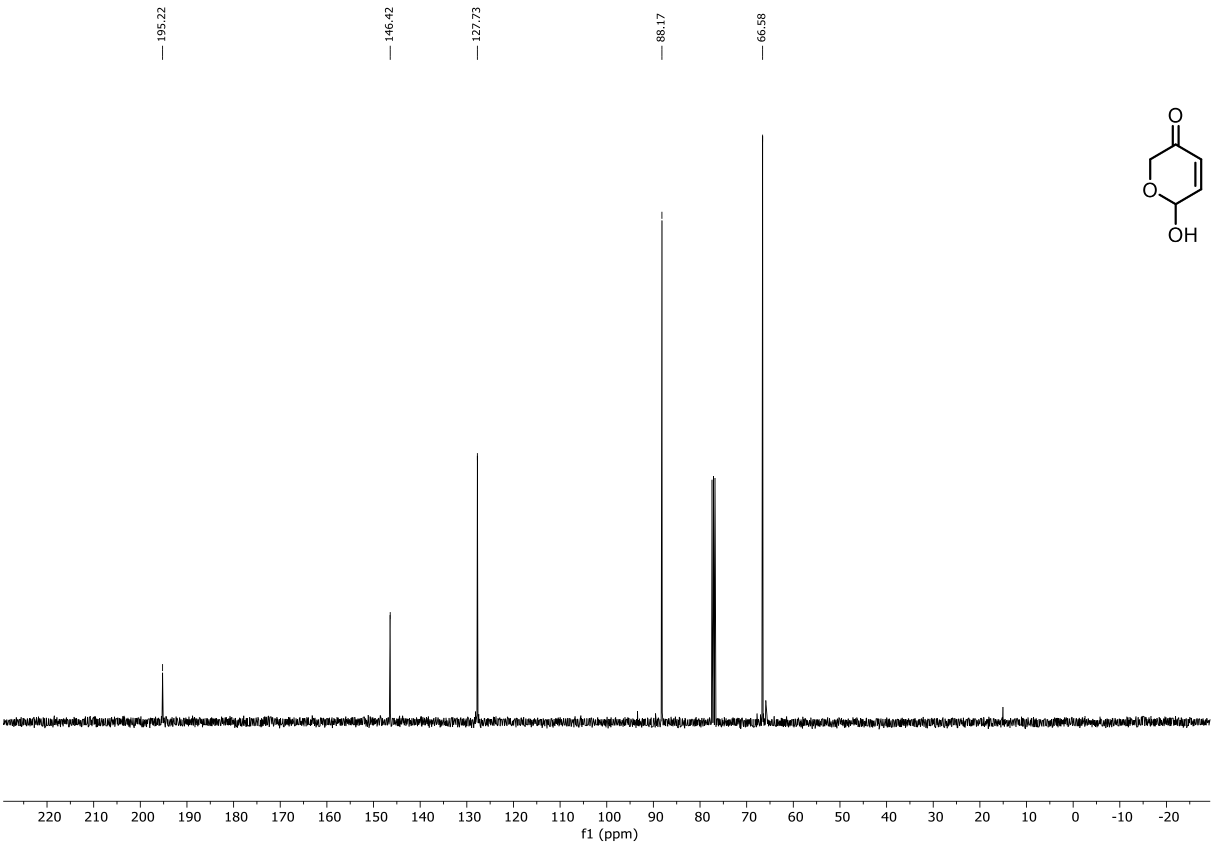

Figure 10.99 (top) ^{1}H NMR (400 MHz) and (bottom) ^{13}C NMR (101 MHz) spectra of **S22**.

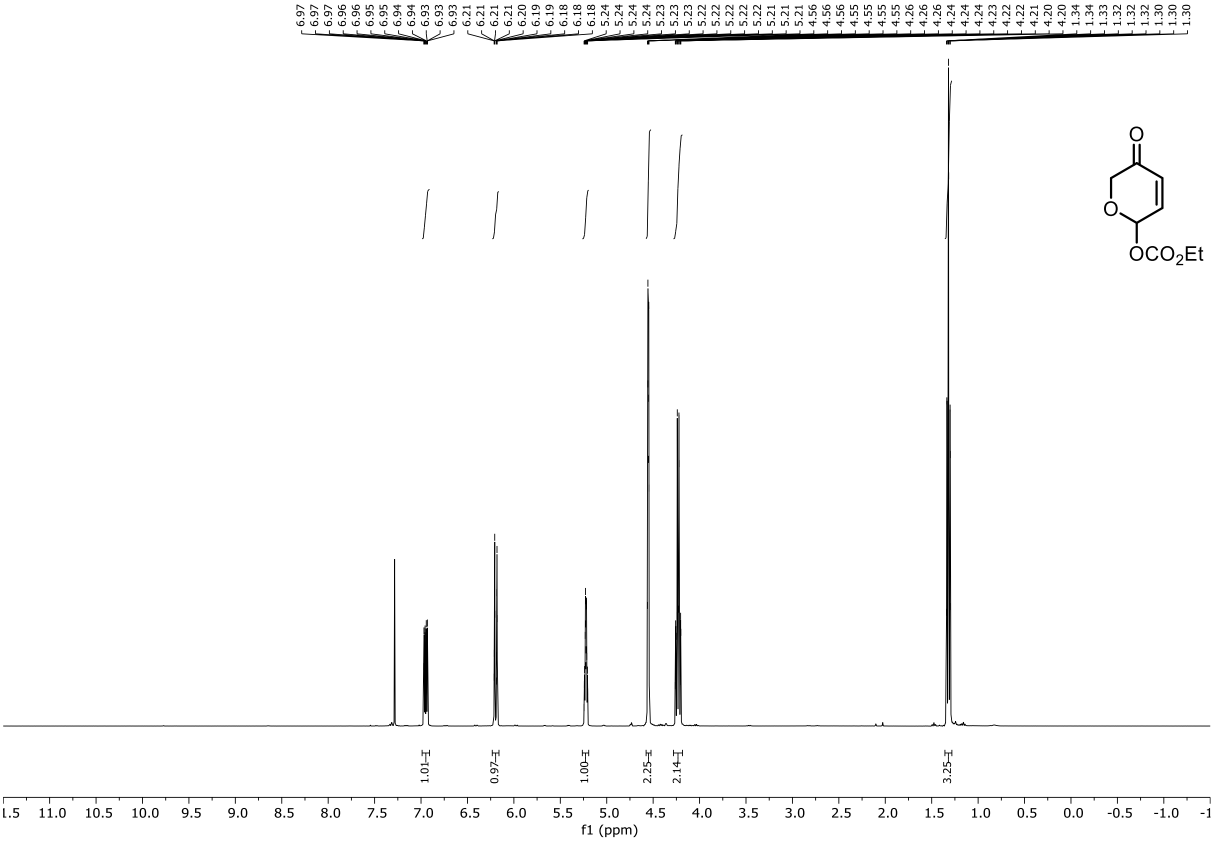

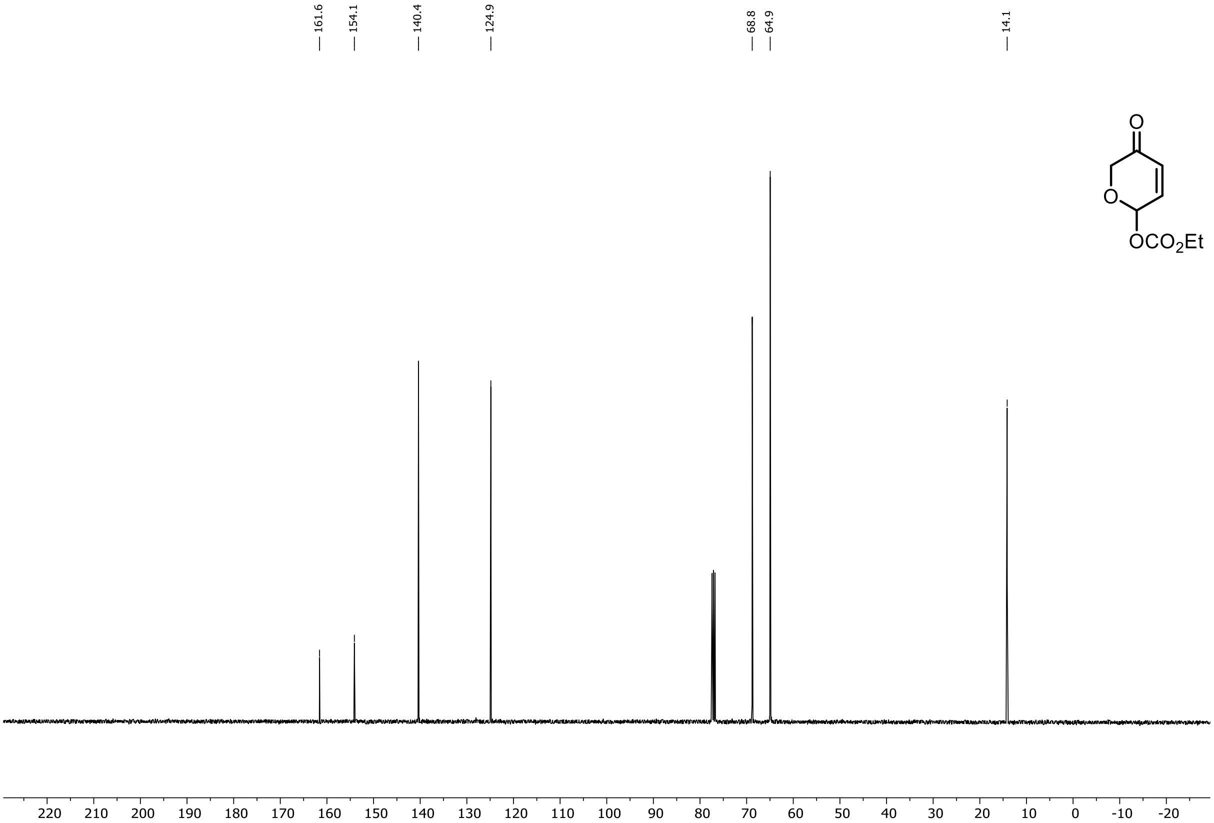

Figure 10.100 (top) ^{1}H NMR (400 MHz) and (bottom) ^{13}C NMR (101 MHz) spectra of (±)-**14**.

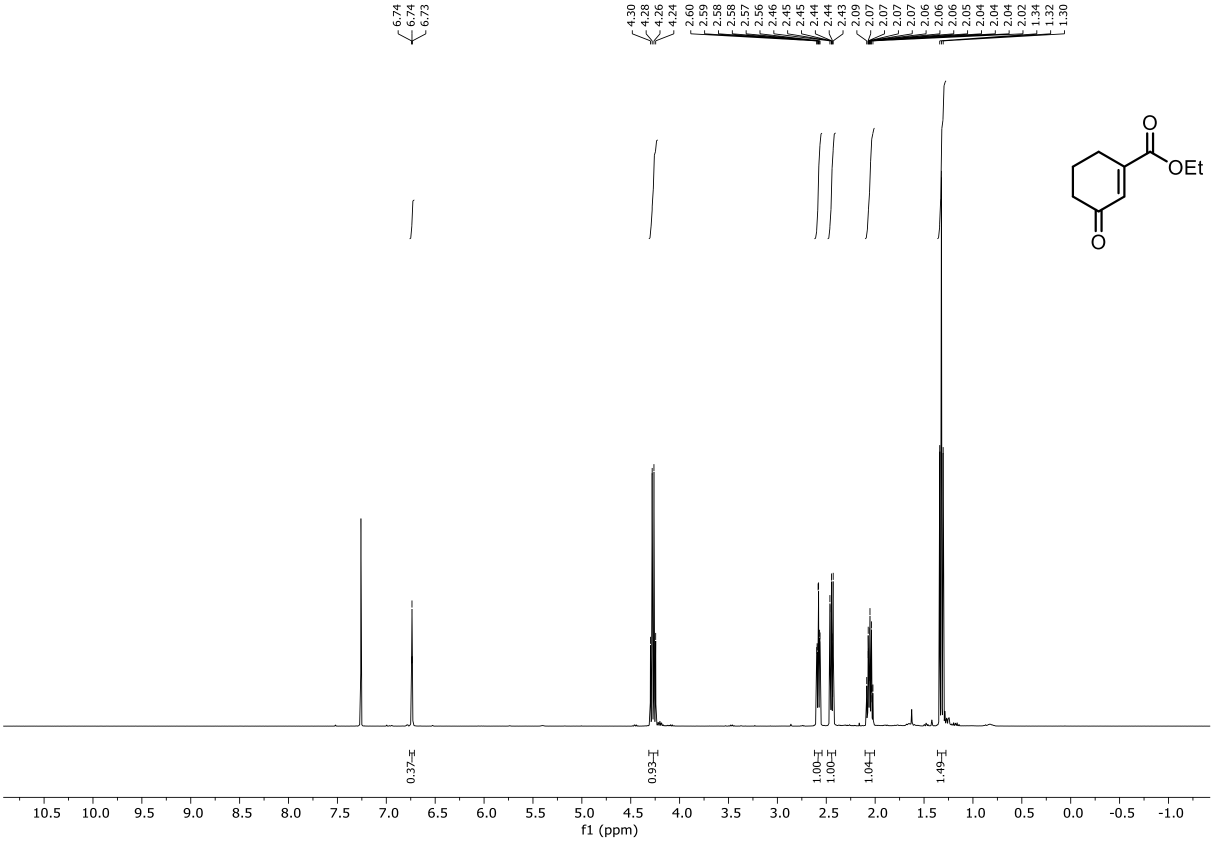

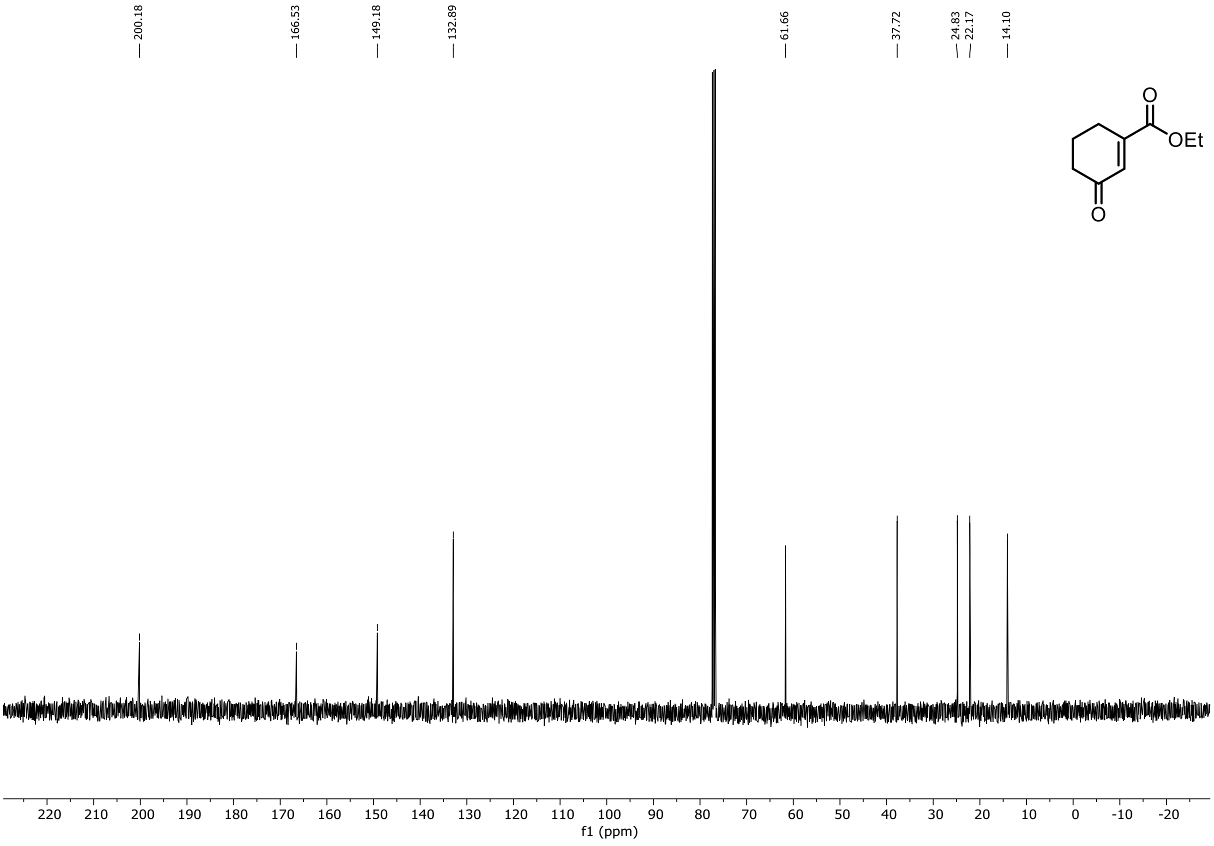

Figure 10.101 (top) ^{1}H NMR (400 MHz) and (bottom) ^{13}C NMR (101 MHz) spectra of **S23**.

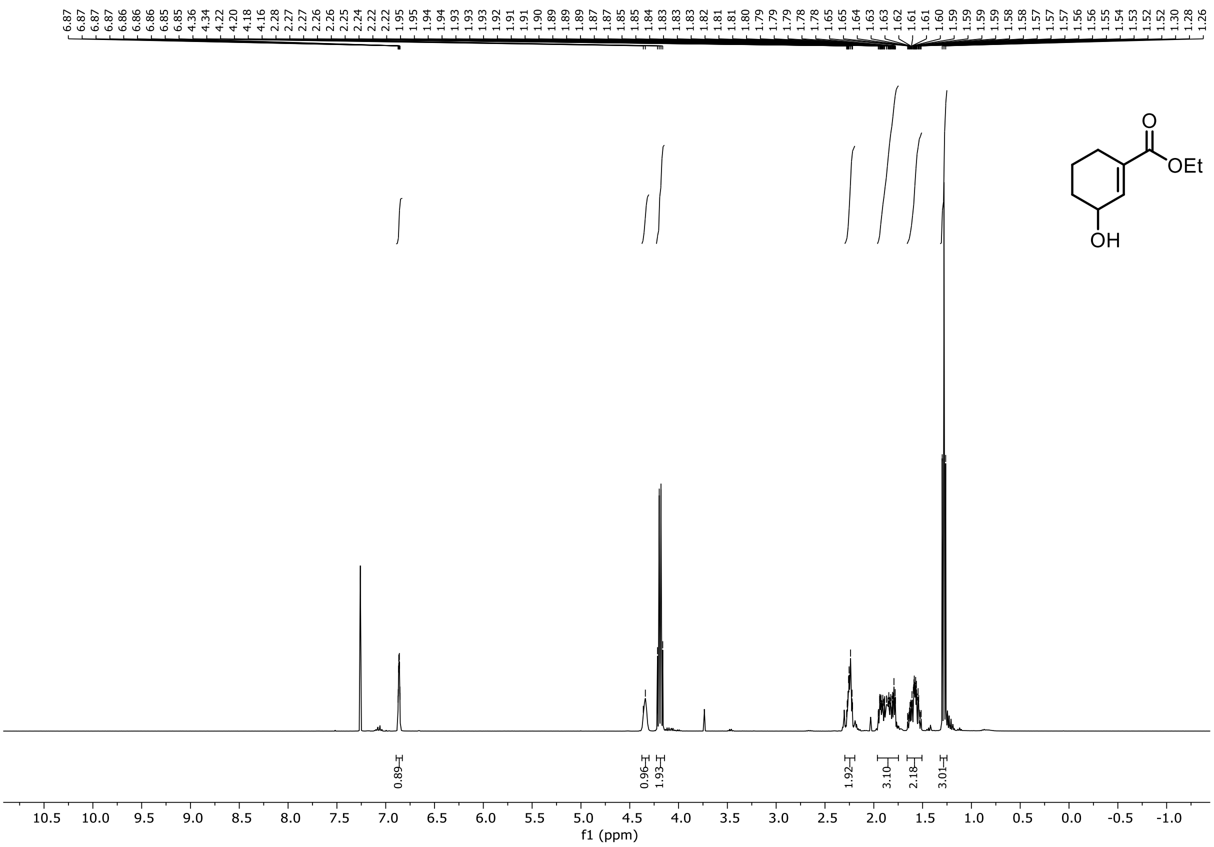

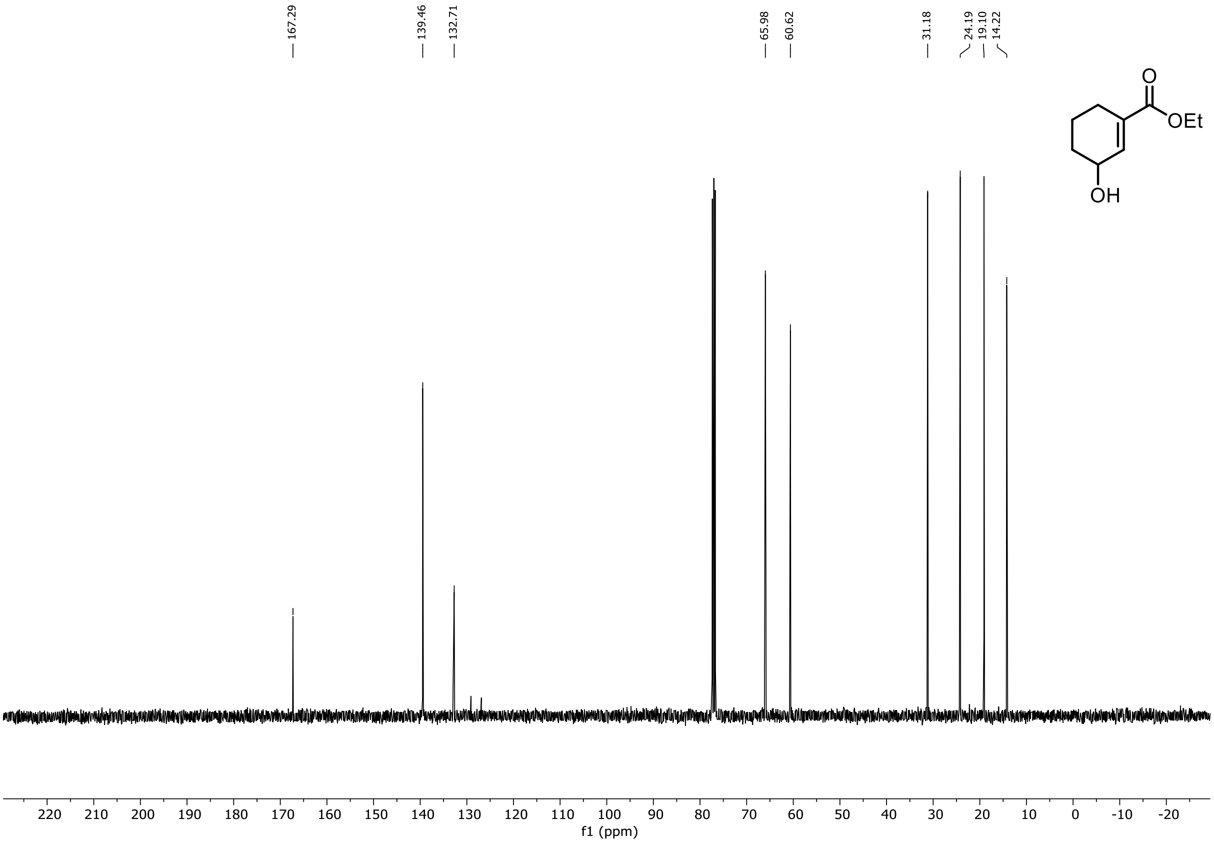

Figure 10.102 (top) ^{1}H NMR (400 MHz) and (bottom) ^{13}C NMR (101 MHz) spectra of **S24**.

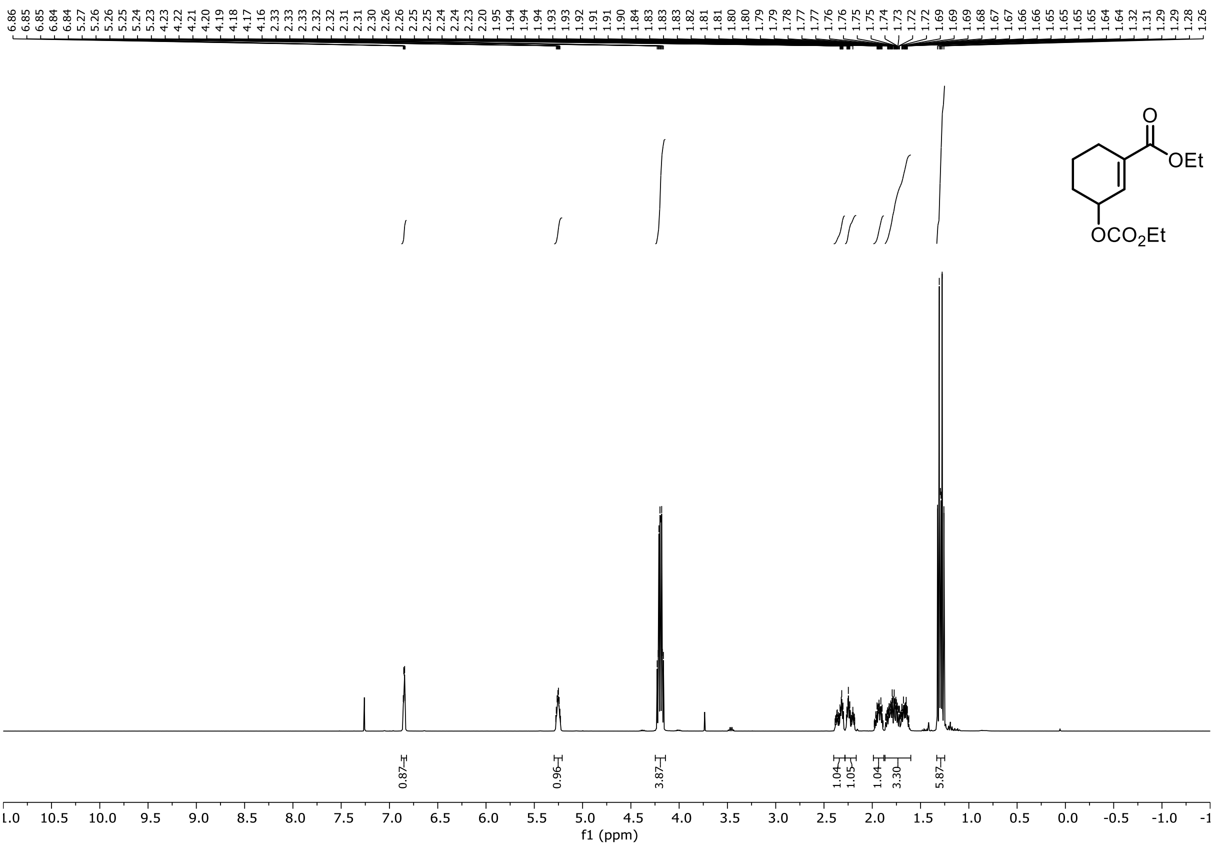

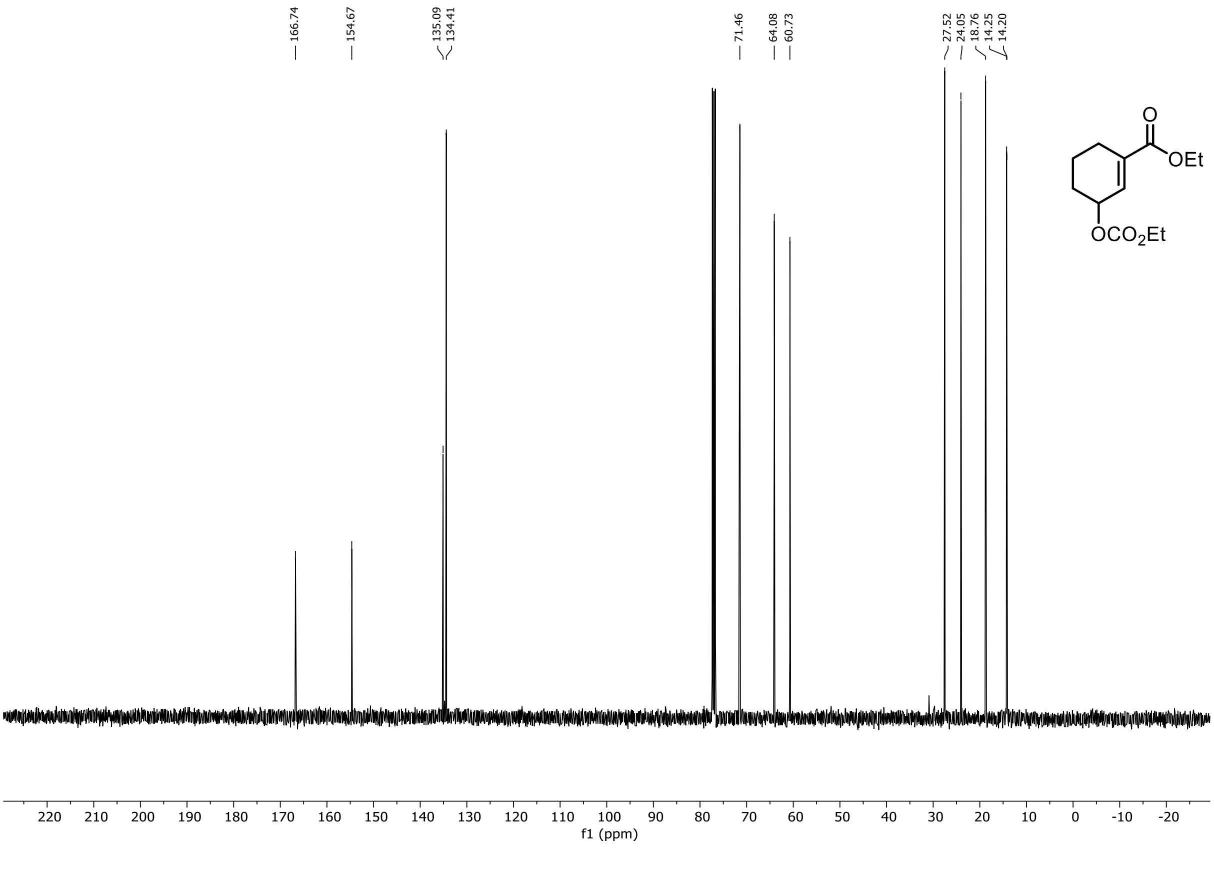

Figure 10.103 (top) ^{1}H NMR (400 MHz) and (bottom) ^{13}C NMR (101 MHz) spectra of (±)-**16**.

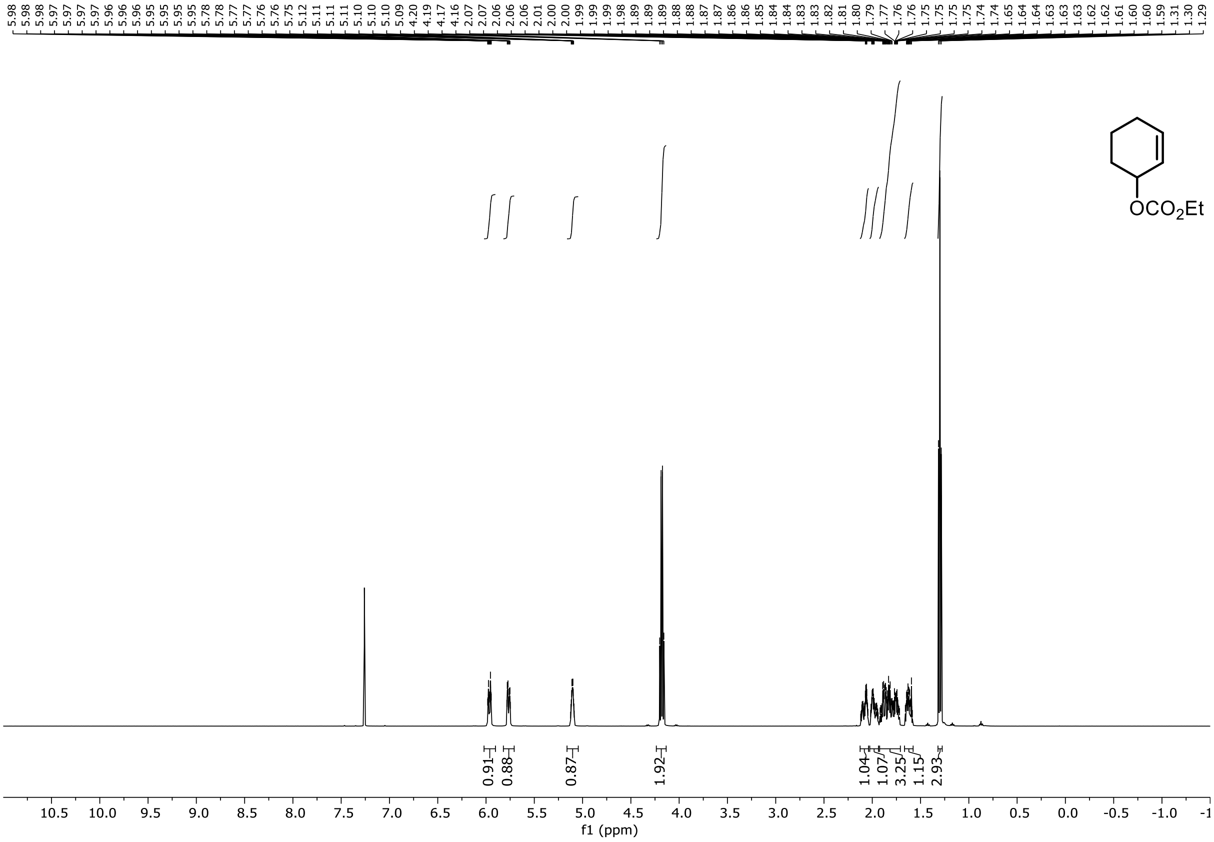

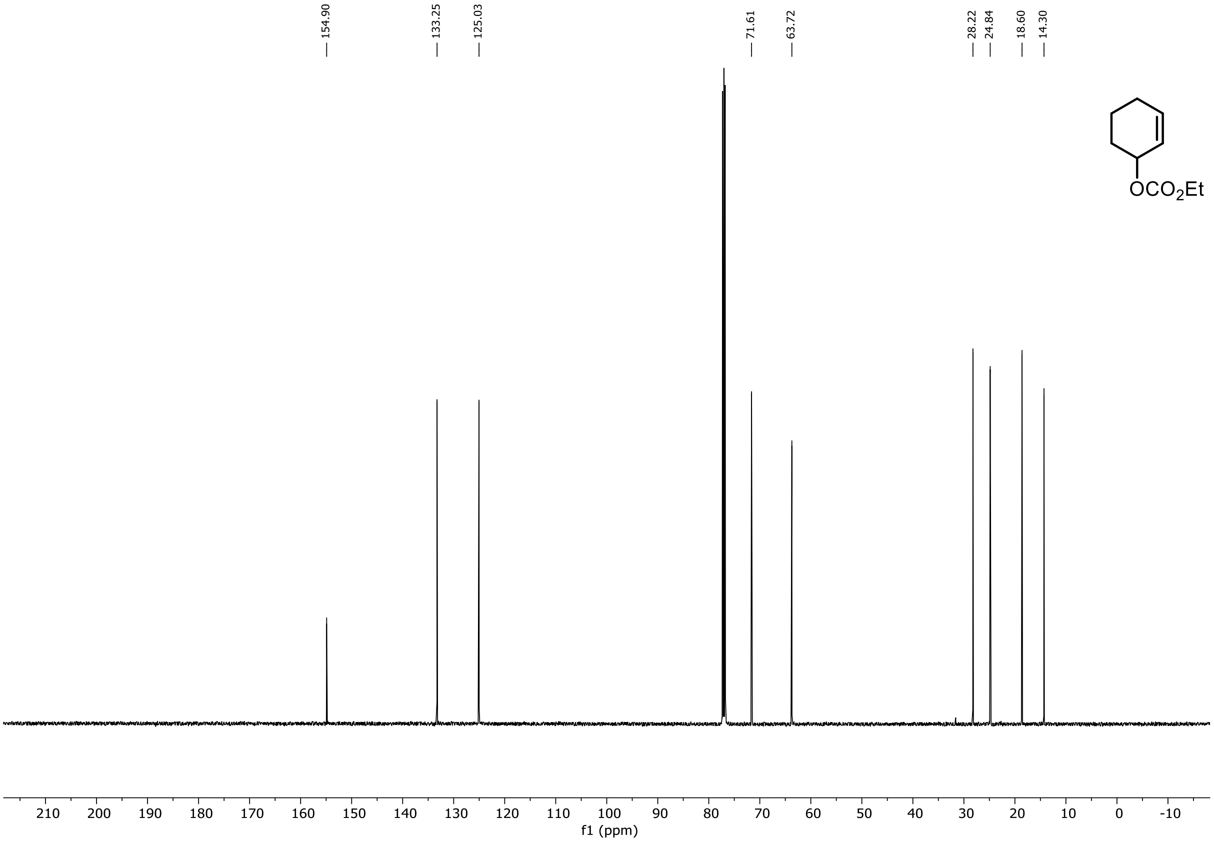

Figure 10.104 (top) ^{1}H NMR (400 MHz) and (bottom) ^{13}C NMR (101 MHz) spectra of (±)-**17**.

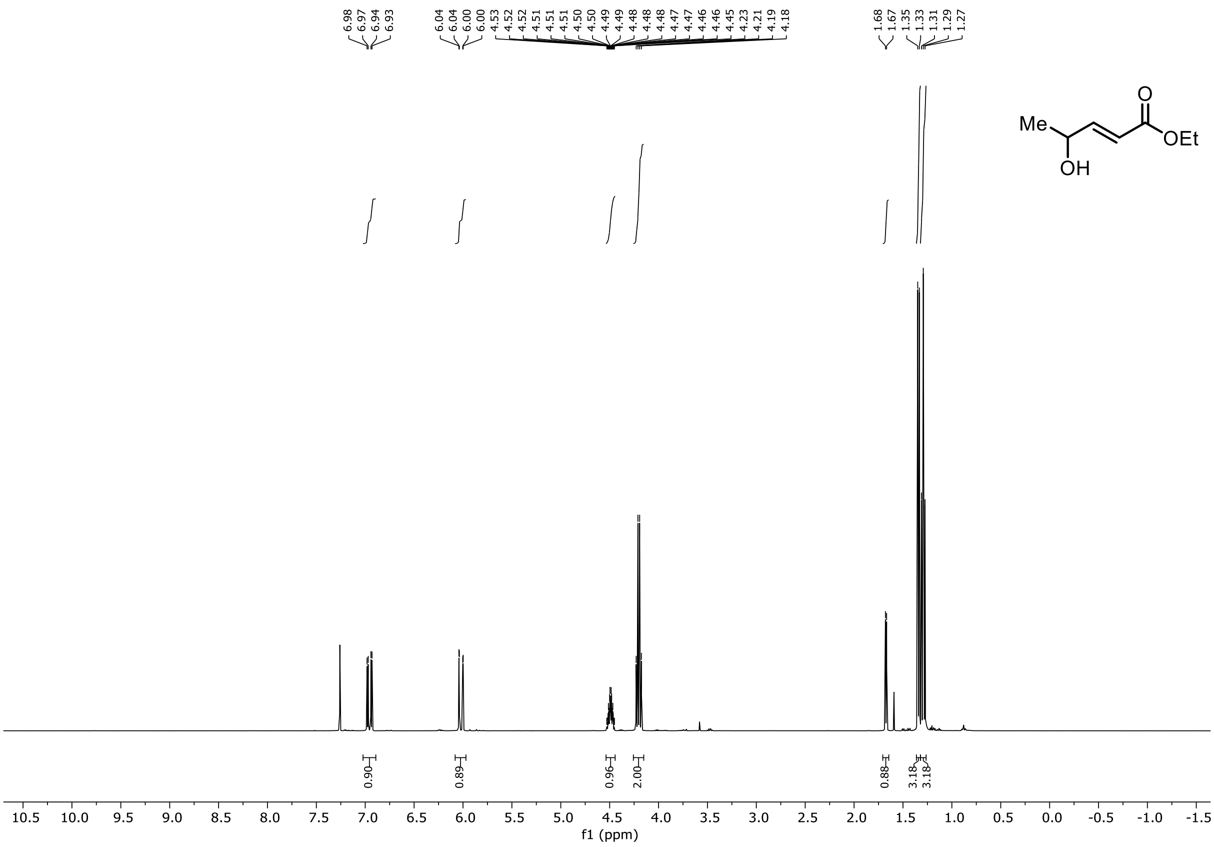

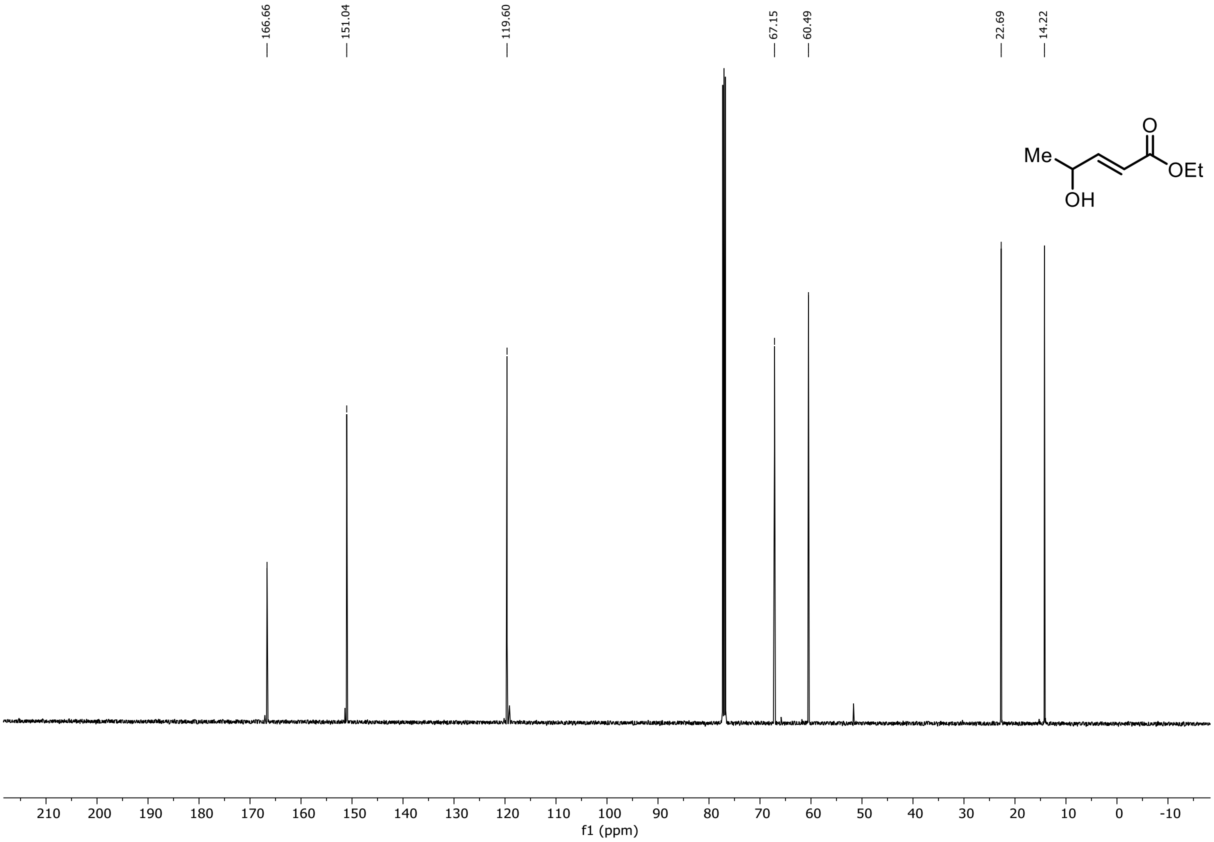

Figure 10.105 (top) ^{1}H NMR (400 MHz) and (bottom) ^{13}C NMR (101 MHz) spectra of **S25**.

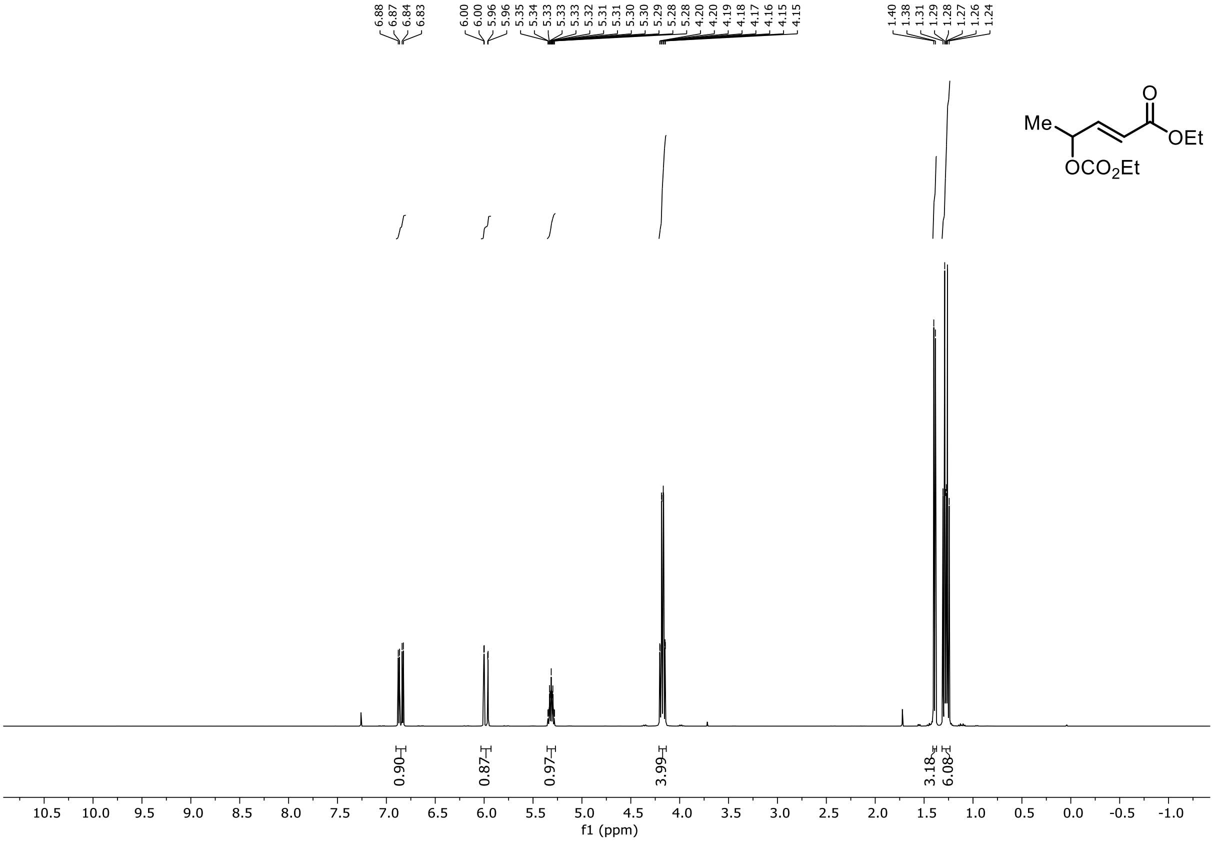

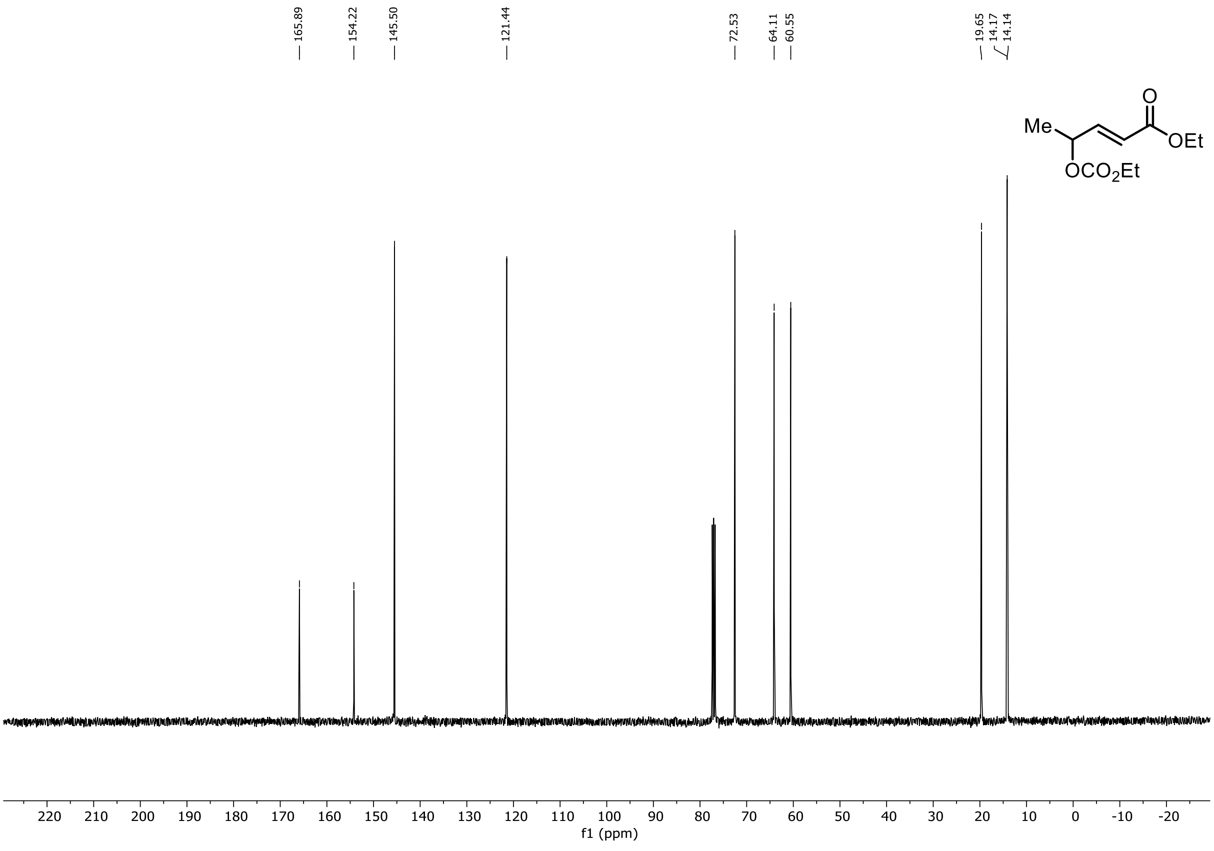

Figure 10.106 (top) ^{1}H NMR (400 MHz) and (bottom) ^{13}C NMR (101 MHz) spectra of (±)-**18**.

11. SFC traces

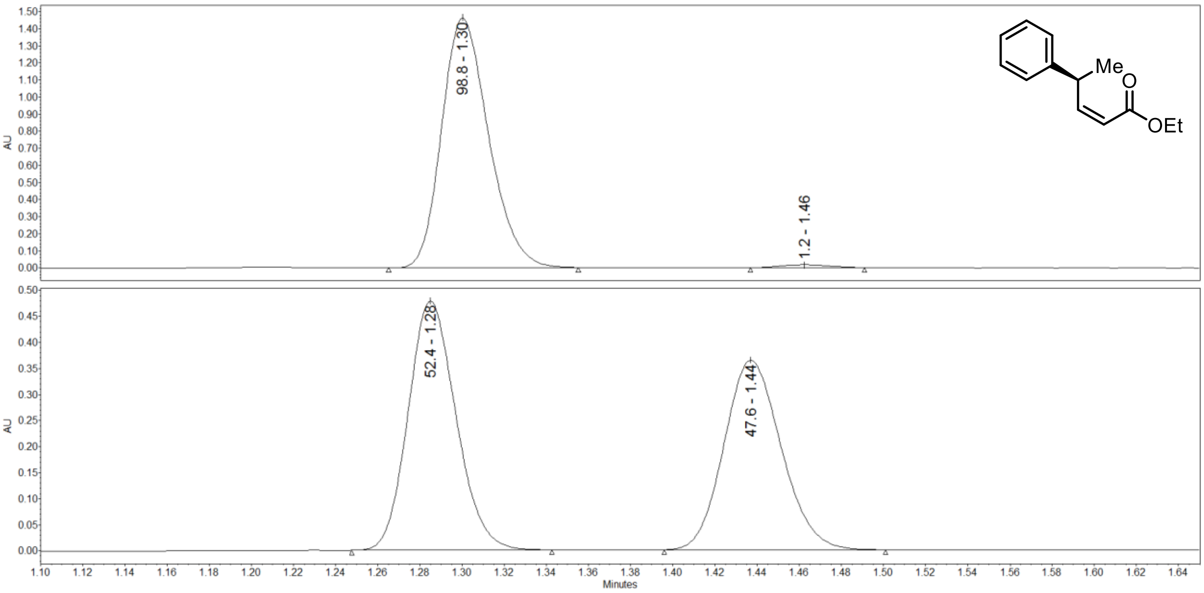

Figure 11.1 SFC trace for (*S*)-*Z*-**3a** and (±)-*Z*-**3a**.

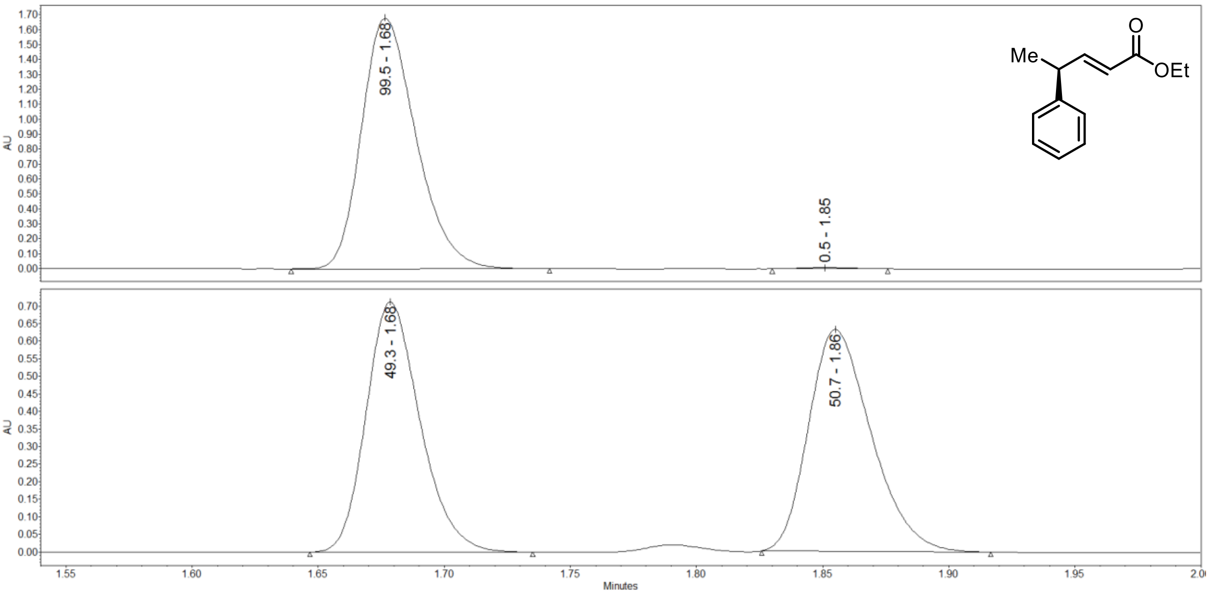

Figure 11.2 SFC trace for (*S*)-*E*-**3a** and (±)-*E*-**3a**.

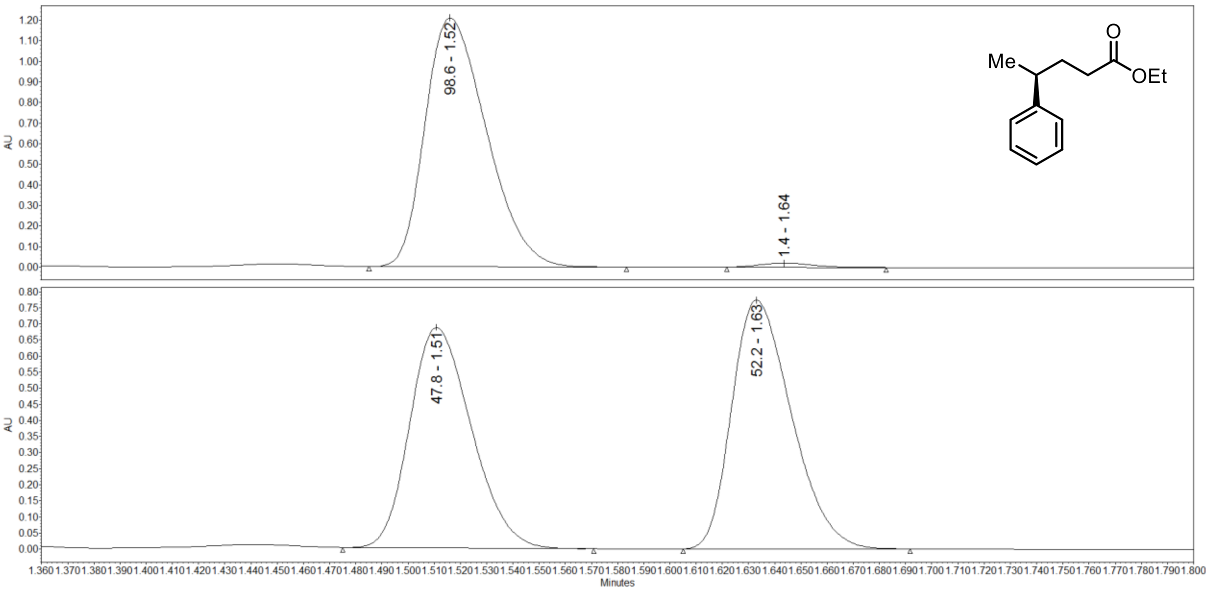

Figure 11.3 SFC trace for (*S*)-*red*-**3a** and (±)-*red*-**3a**.

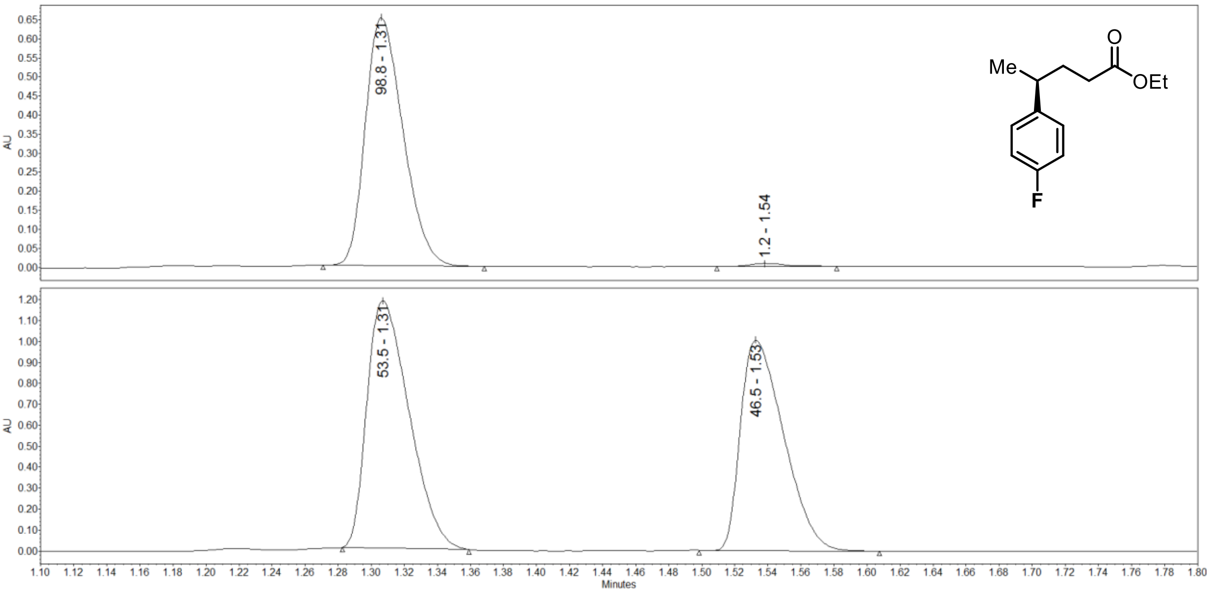

Figure 11.4 SFC trace for (*S*)-*red*-**3b** and (±)-*red*-**3b**.

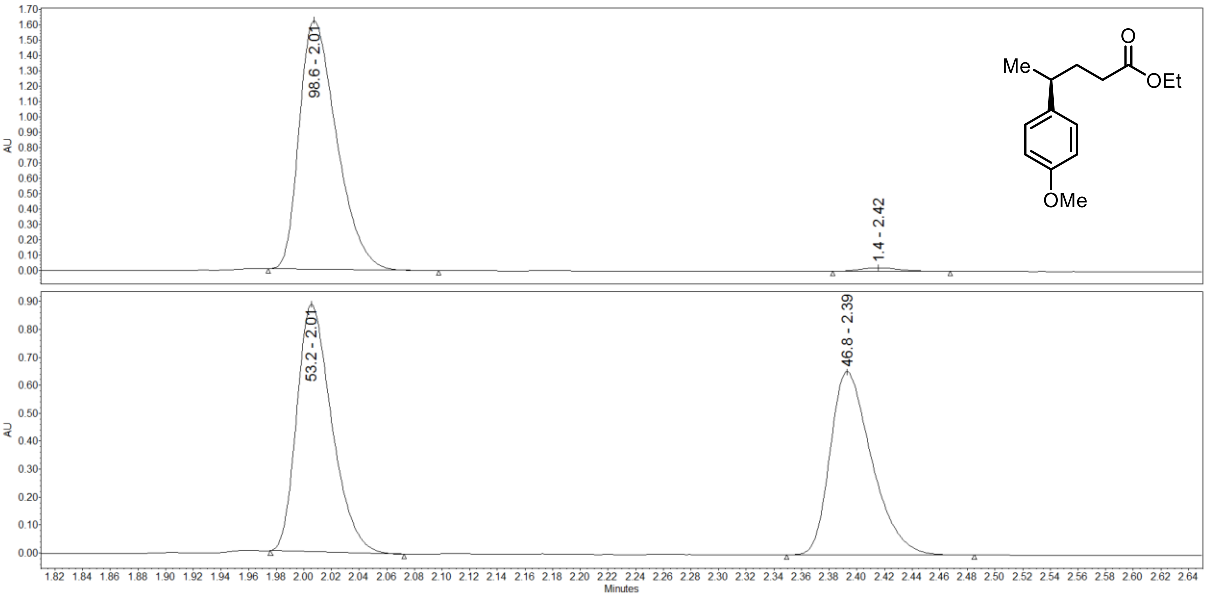

Figure 11.5 SFC trace for (*S*)-*red*-**3c** and (±)-*red*-**3c**.

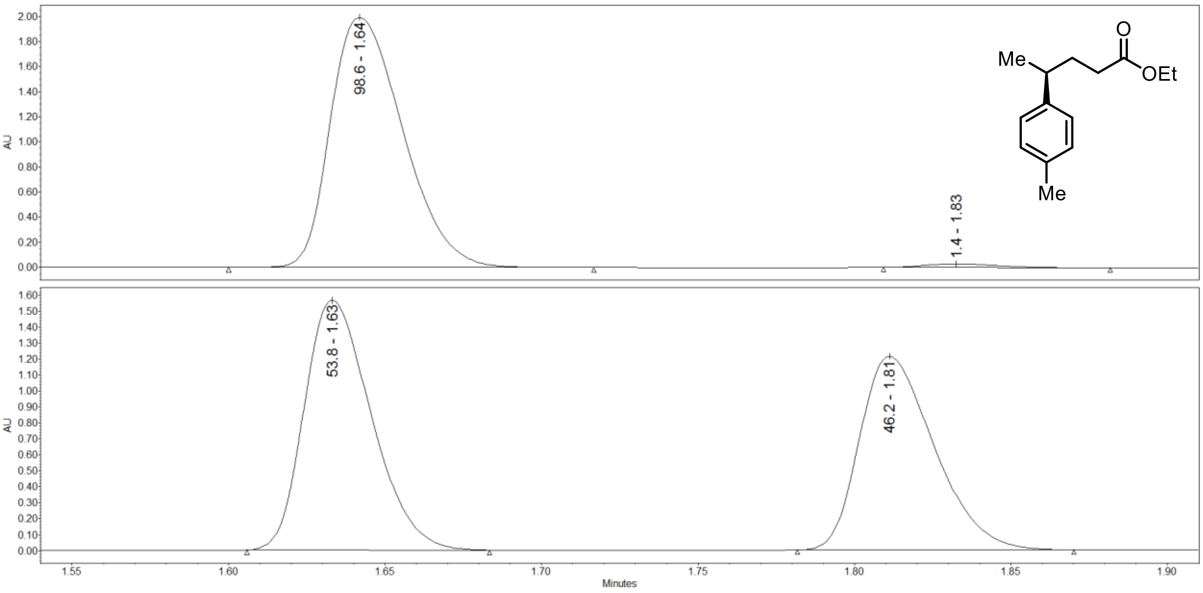

Figure 11.6 SFC trace for (*S*)-*red*-**3d** and (±)-*red*-**3d**.

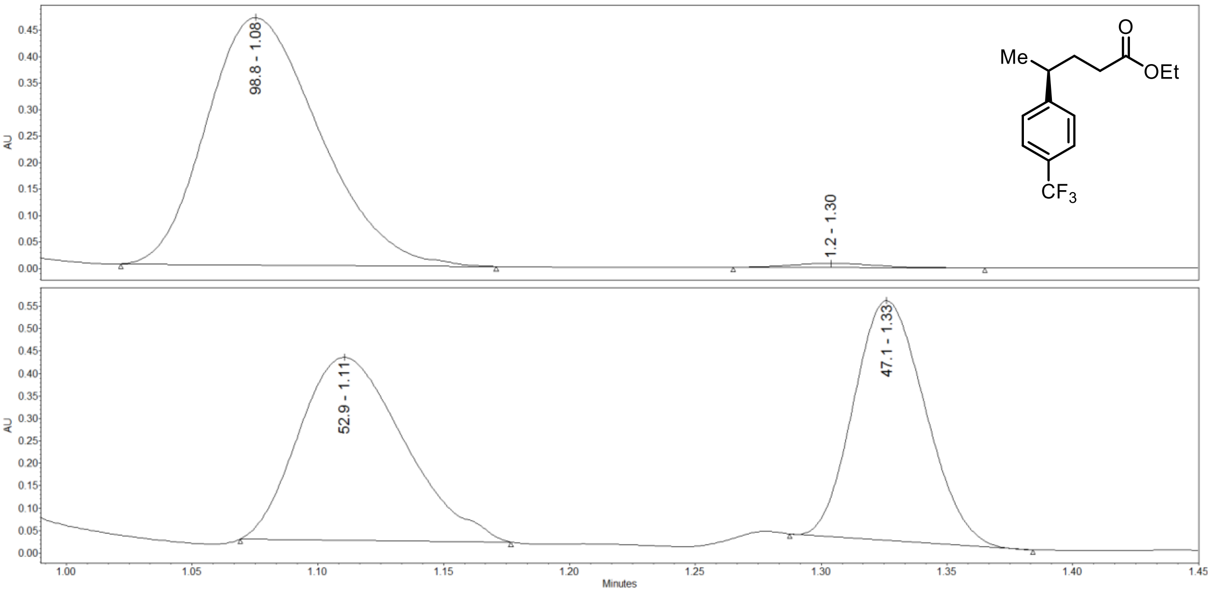

Figure 11.7 SFC trace for (*S*)-*red*-**3e** and (±)-*red*-**3e**.

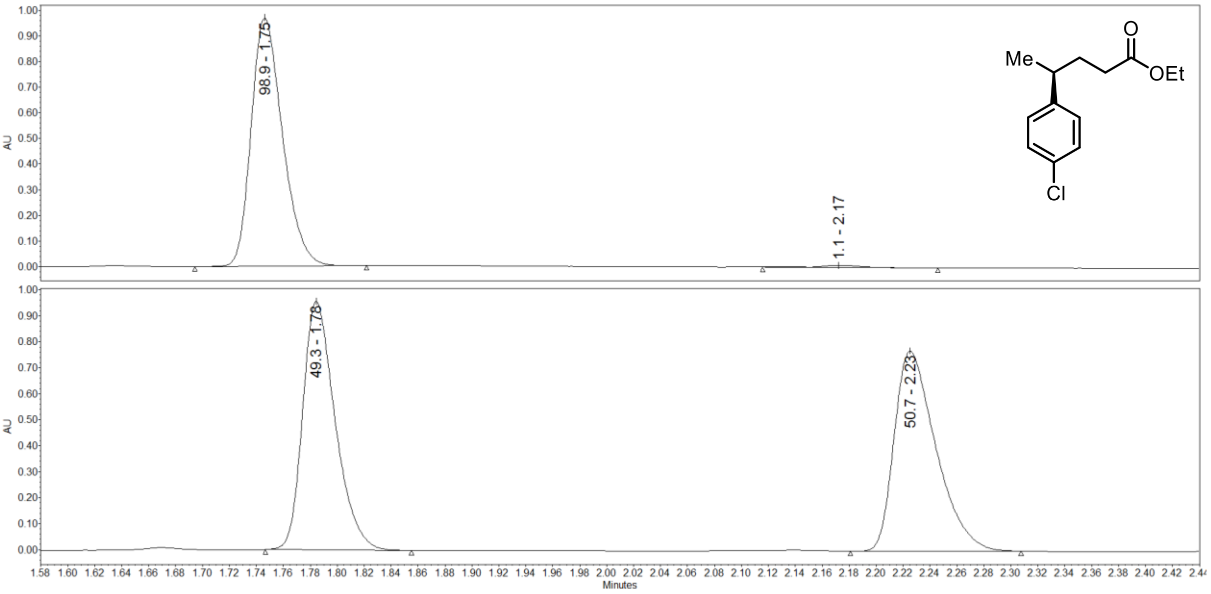

Figure 11.8 SFC trace for (*S*)-*red*-**3f** and (±)-*red*-**3f**.

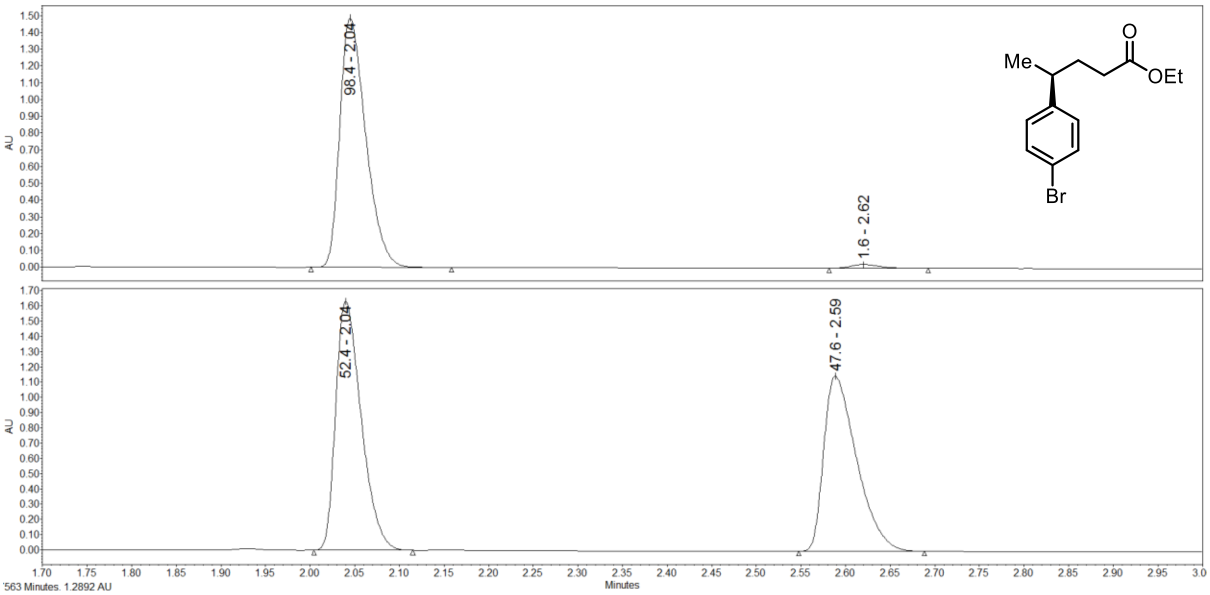

Figure 11.9 SFC trace for (*S*)-*red*-**3g** and (±)-*red*-**3g**.

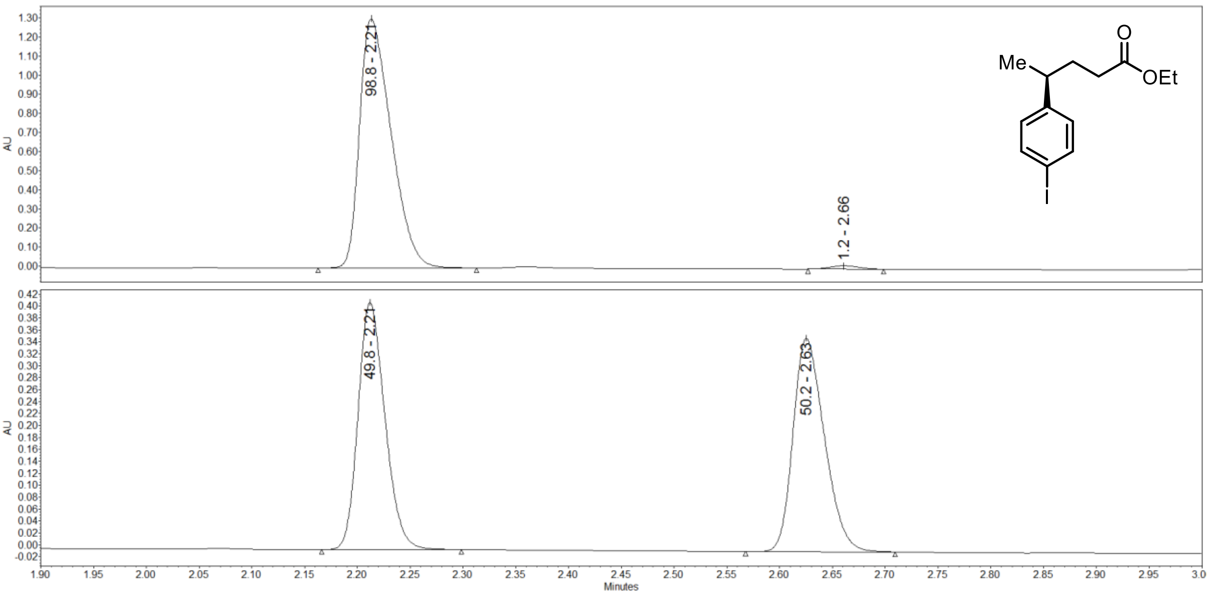

Figure 11.10 SFC trace for (*S*)-*red*-**3h** and (±)-*red*-**3h**.

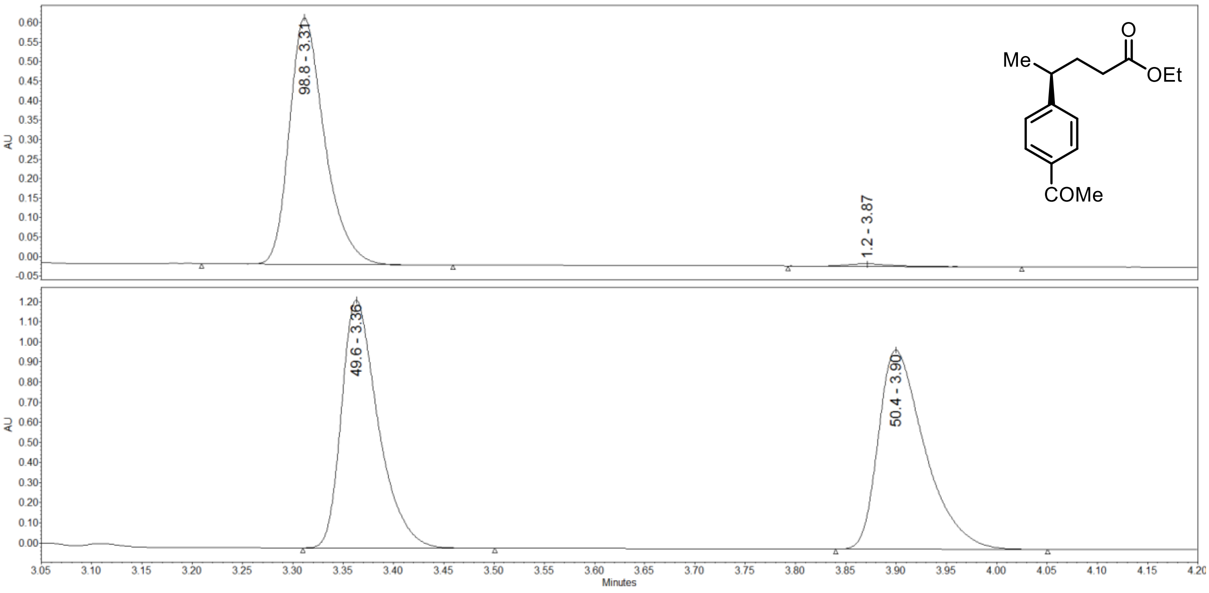

Figure 11.11 SFC trace for (*S*)-*red*-**3i** and (±)-*red*-**3i**.

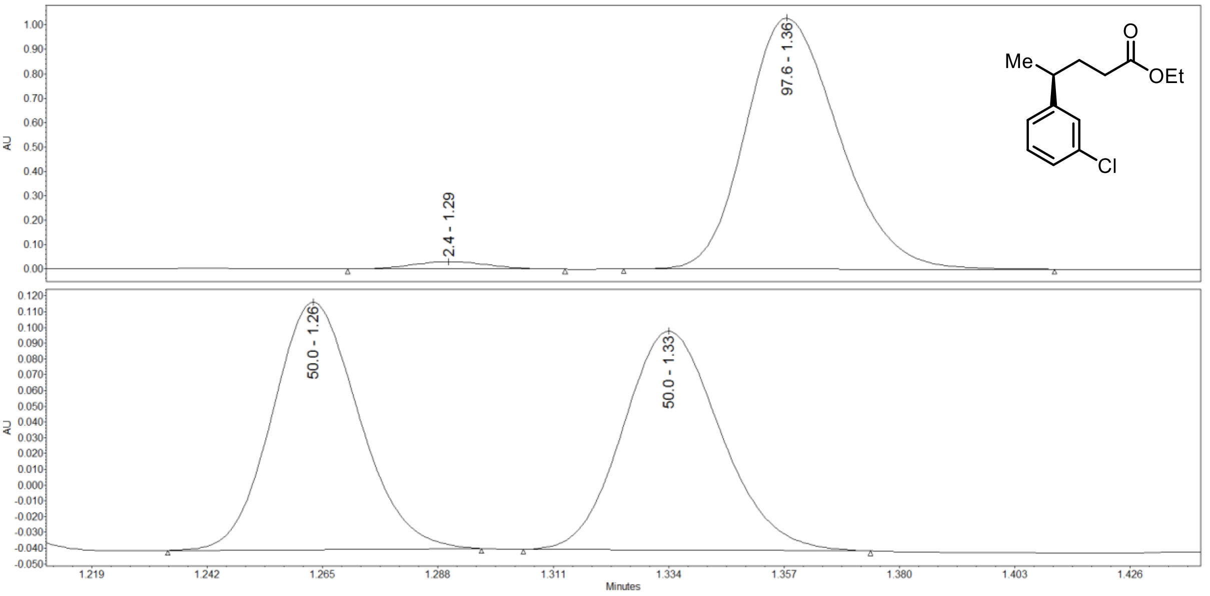

Figure 11.12 SFC trace for (*S*)-*red*-**3j** and (±)-*red*-**3j**.

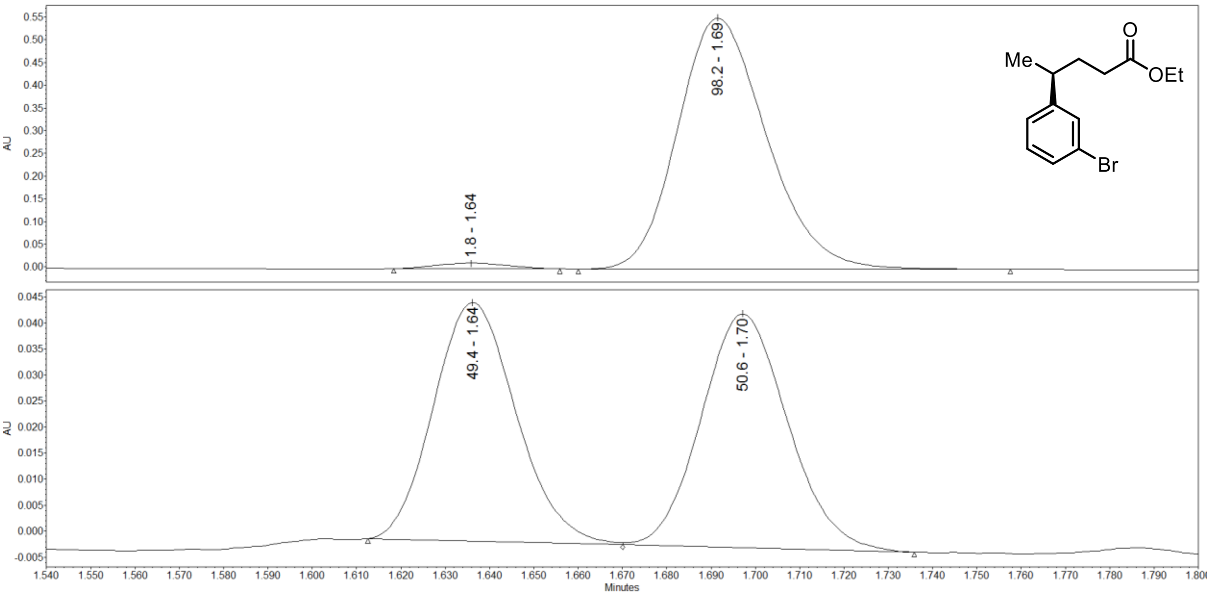

Figure 11.13 SFC trace for (*S*)-*red*-**3k** and (±)-*red*-**3k**.

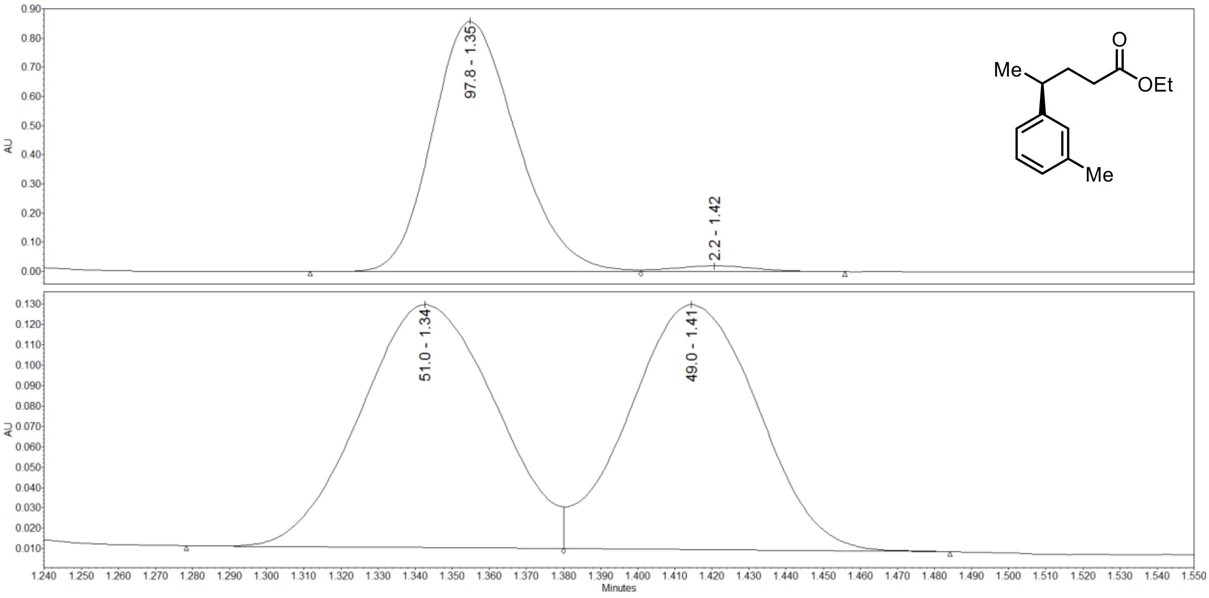

Figure 11.14 SFC trace for (*S*)-*red*-**3l** and (±)-*red*-**3l**.

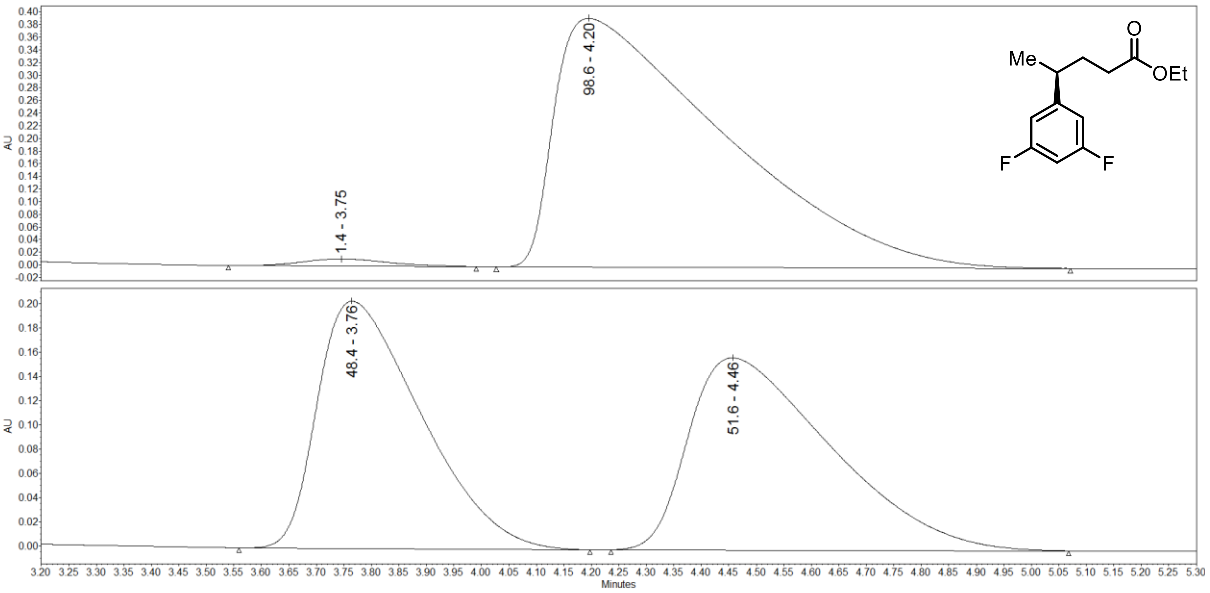

Figure 11.15 SFC trace for (*S*)-*red*-**3m** and (±)-*red*-**3m**.

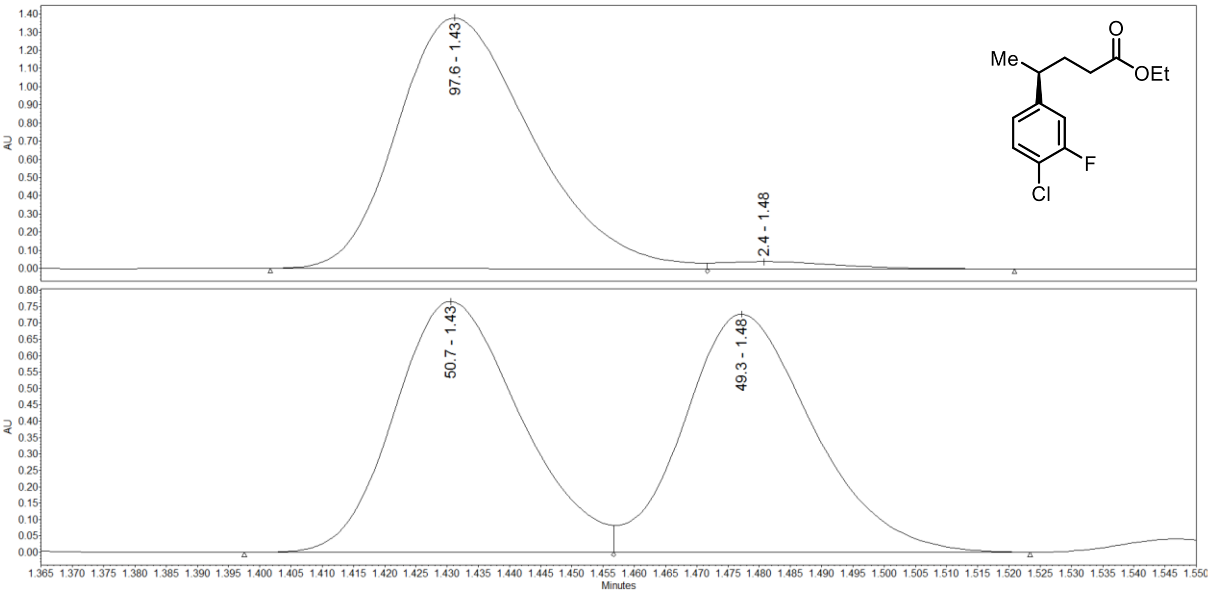

Figure 11.16 SFC trace for (*S*)-*red*-**3n** and (±)-*red*-**3n**.

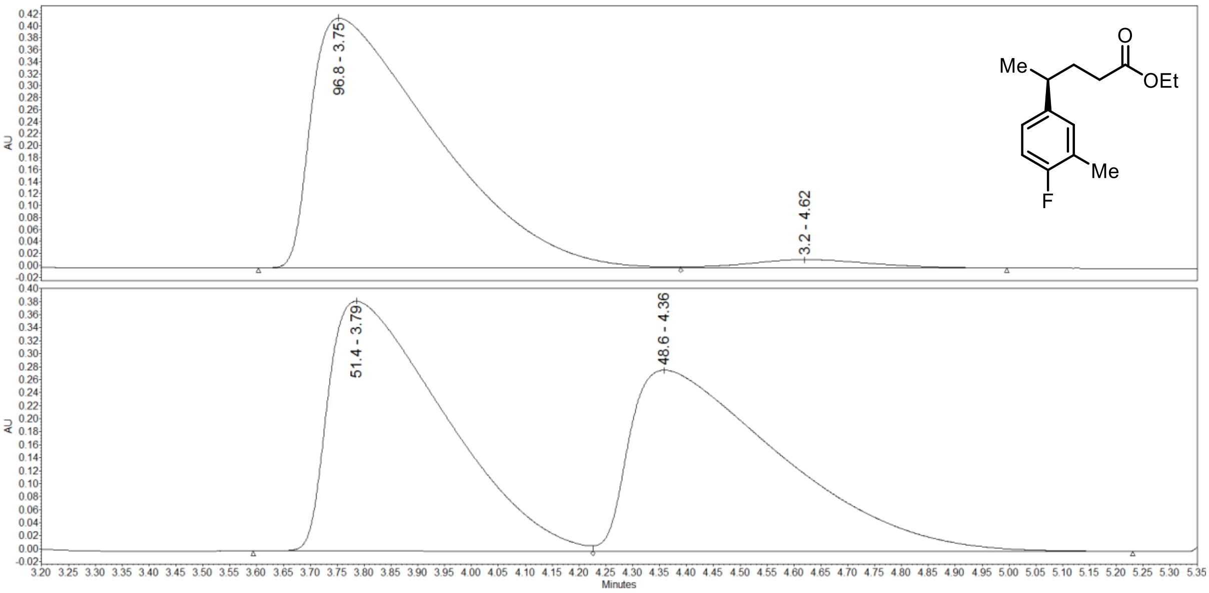

Figure 11.17 SFC trace for (*S*)-*red*-**3o** and (±)-*red*-**3o**.

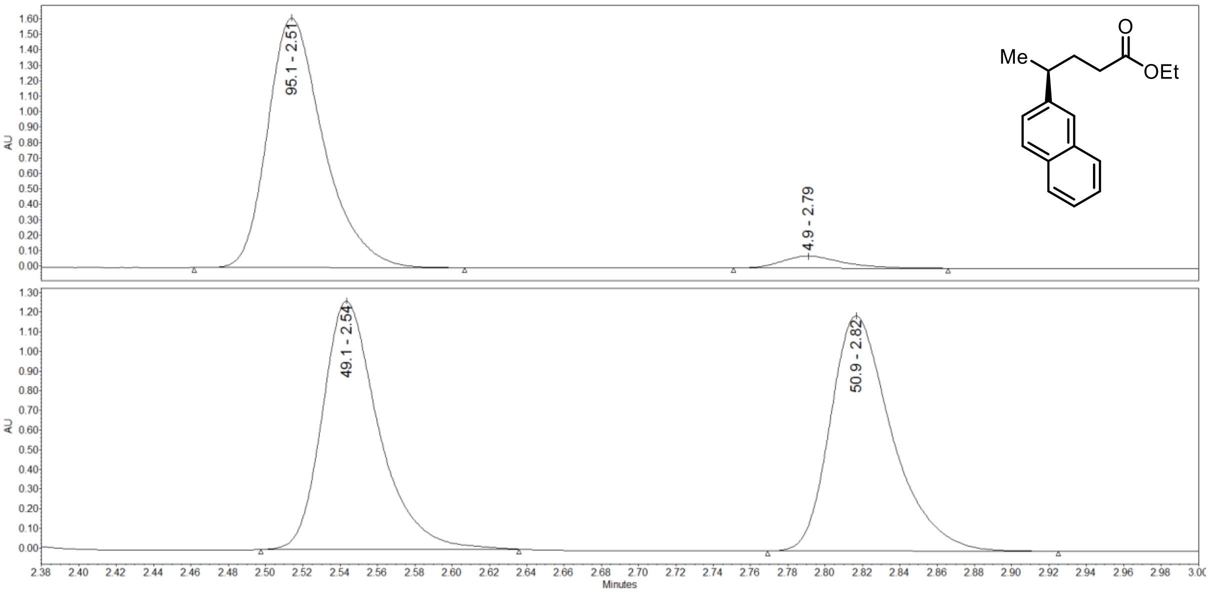

Figure 11.18 SFC trace for (*S*)-*red*-**3p** and (±)-*red*-**3p**.

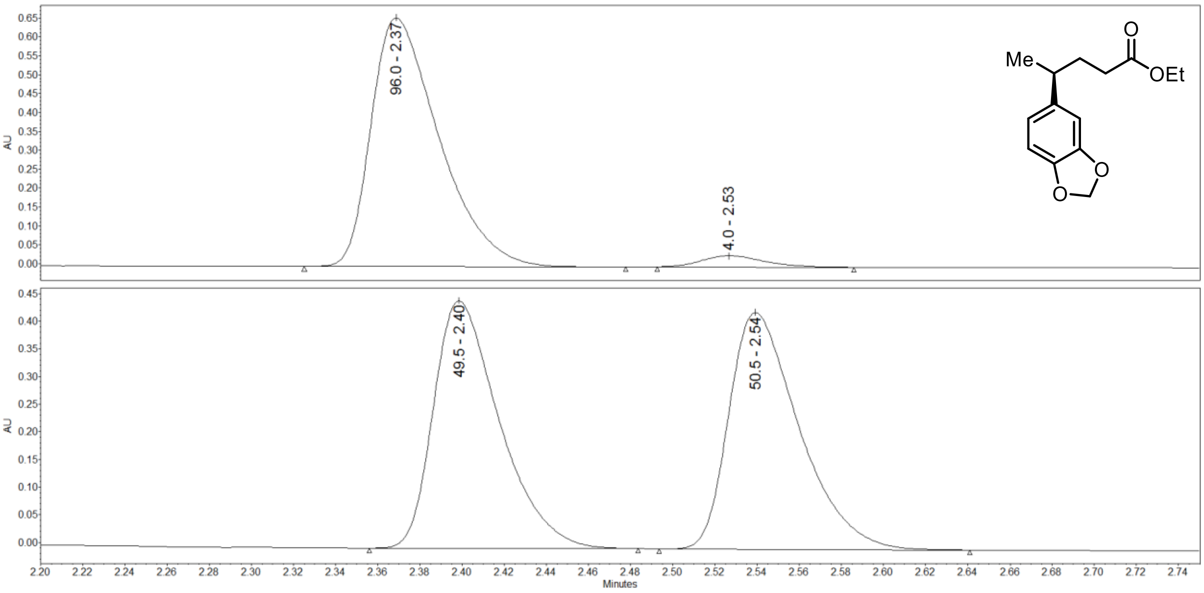

Figure 11.19 SFC trace for (*S*)-*red*-**3q** and (±)-*red*-**3q**.

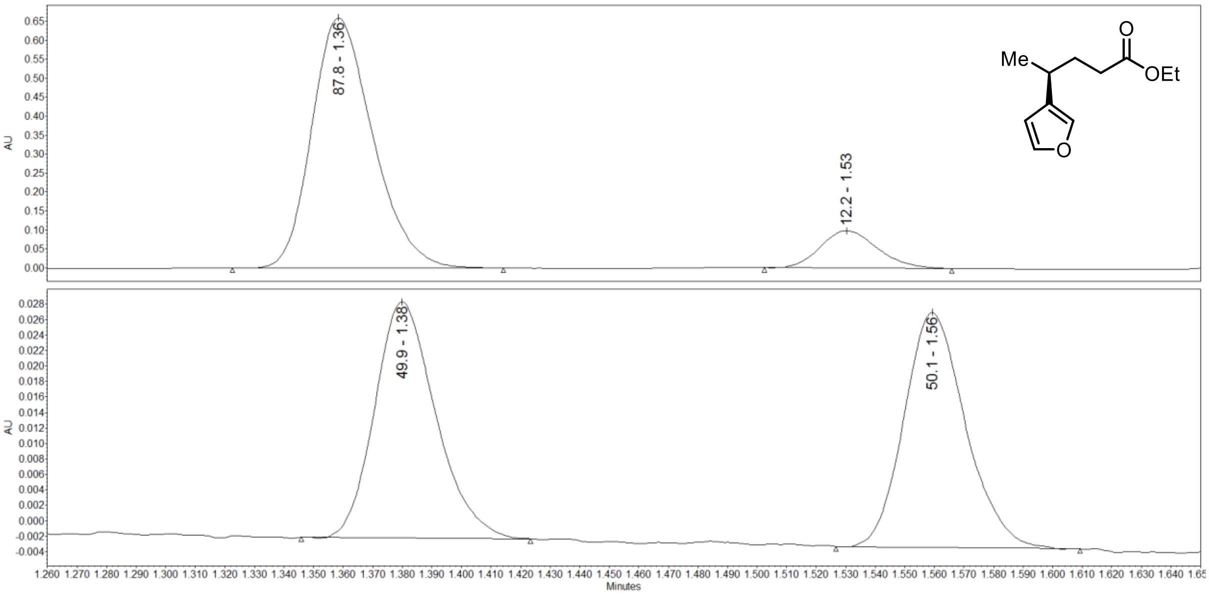

Figure 11.20 SFC trace for (*S*)-*red*-**3r** and (±)-*red*-**3r**.

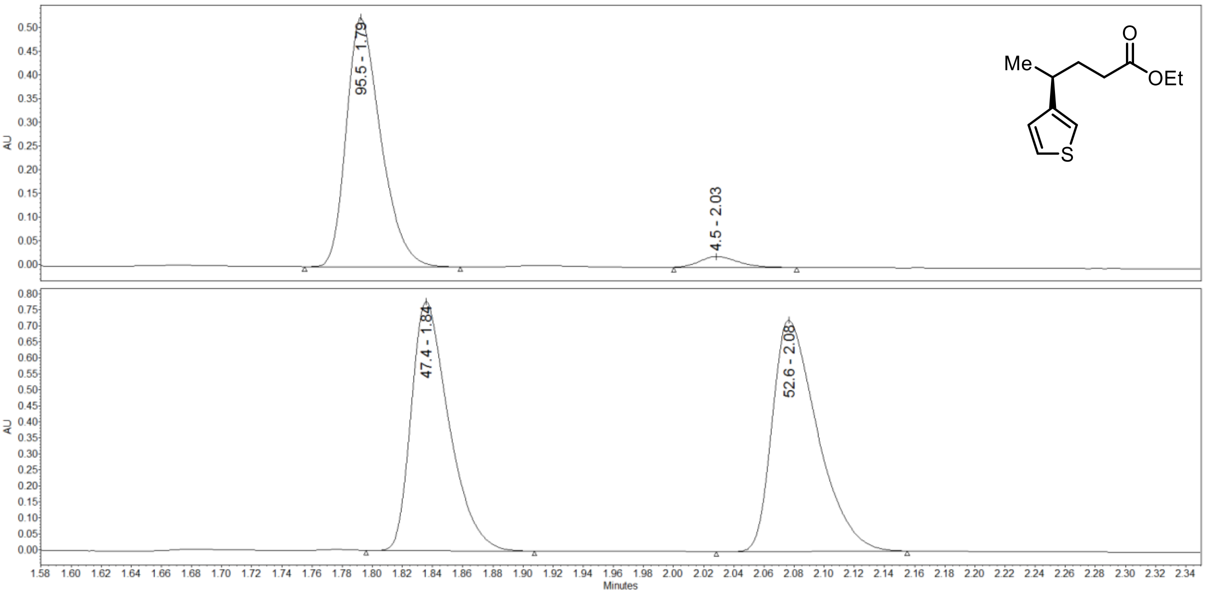

Figure 11.21 SFC trace for (*S*)-*red*-**3s** and (±)-*red*-**3s**.

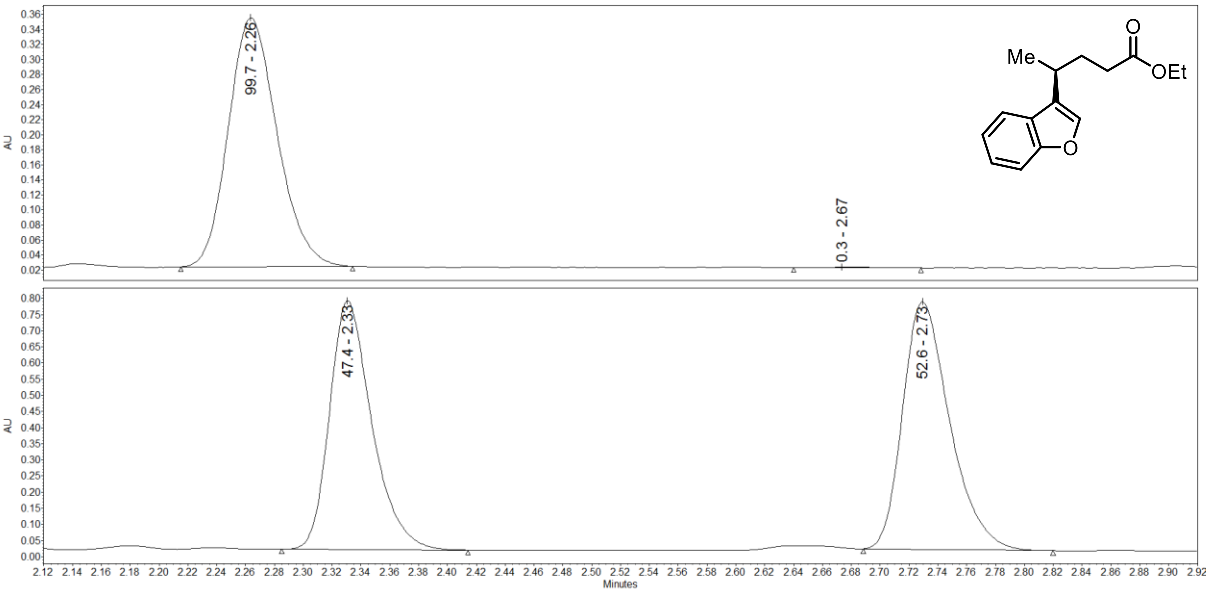

Figure 11.22 SFC trace for (*S*)-*red*-**3t** and (±)-*red*-**3t**.

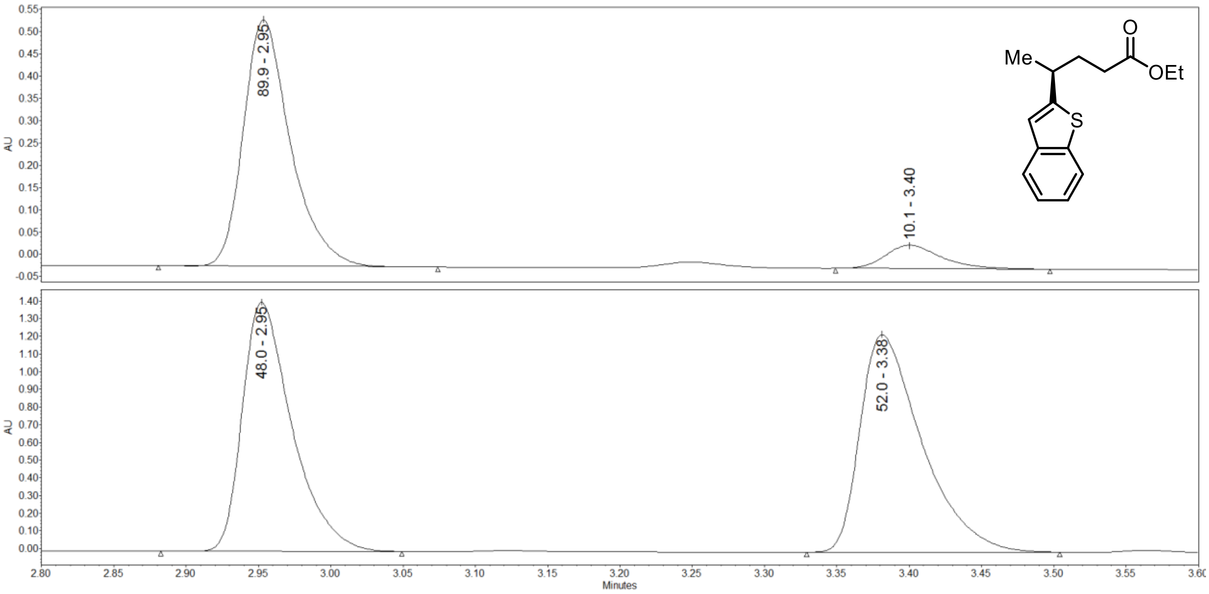

Figure 11.23 SFC trace for (*S*)-*red*-**3u** and (±)-*red*-**3u**.

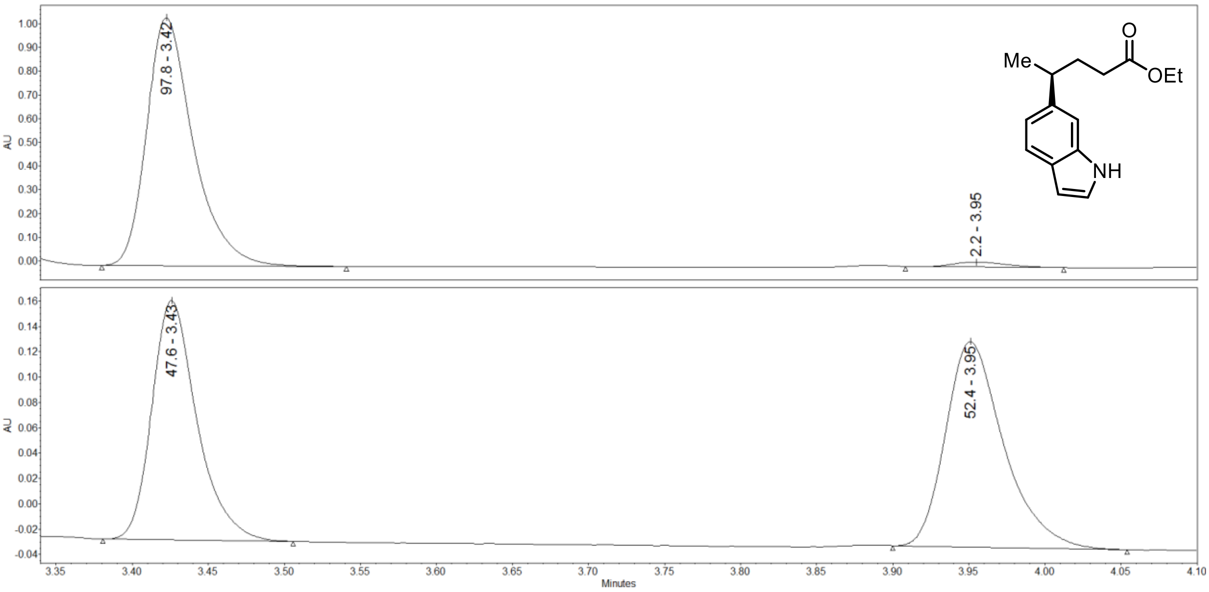

Figure 11.24 SFC trace for (*S*)-*red*-**3v** and (±)-*red*-**3v**.

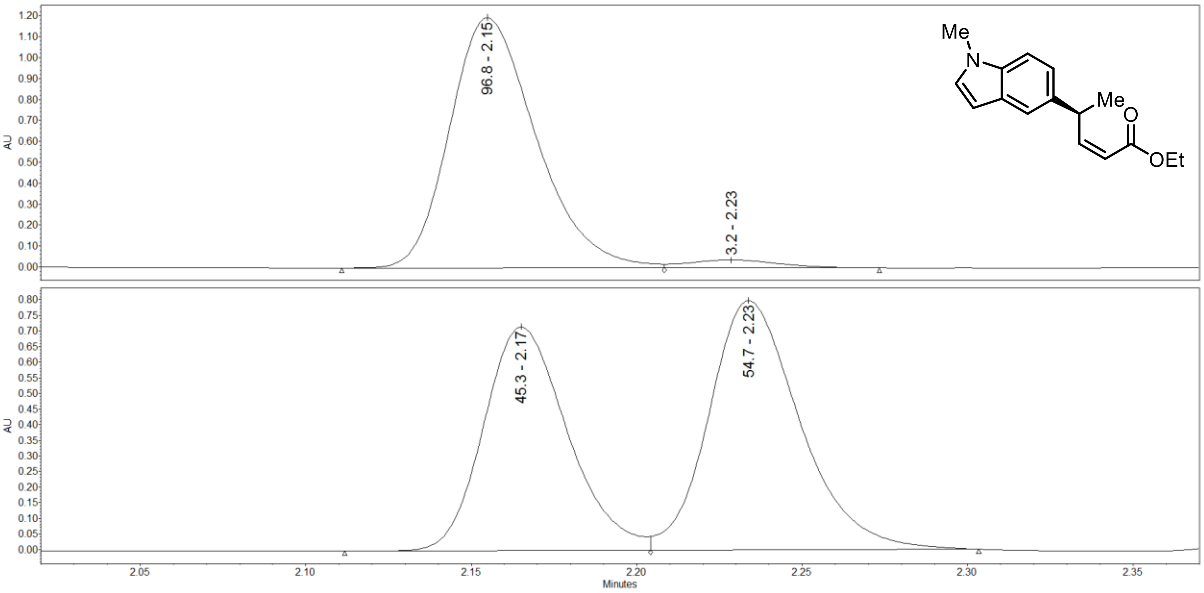

Figure 11.25 SFC trace for (*S*)-*Z*-**3w** and (±)-*Z*-**3w**.

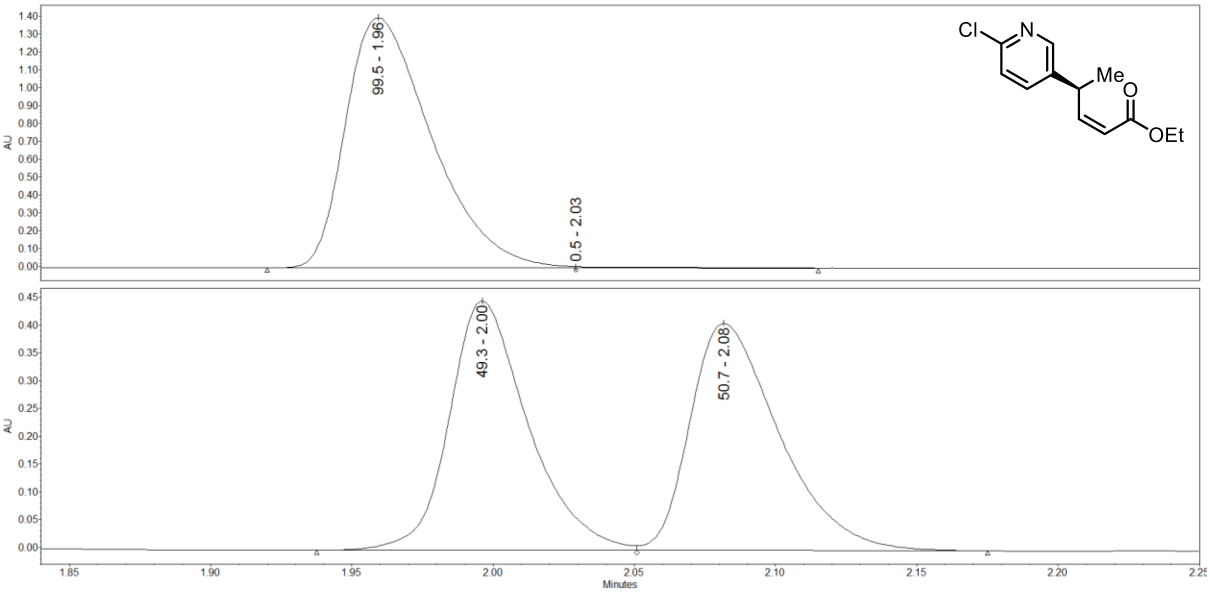

Figure 11.26 SFC trace for (*S*)-*Z*-**3x** and (±)-*Z*-**3x**.

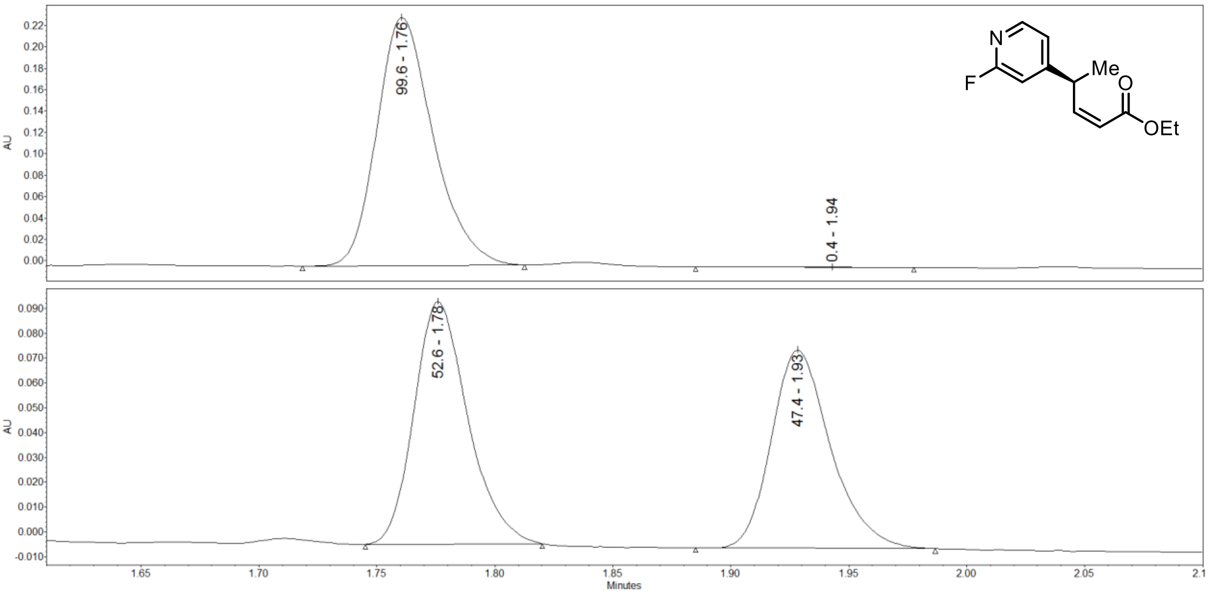

Figure 11.27 SFC trace for (*S*)-*Z*-**3y** and (±)-*Z*-**3y**.

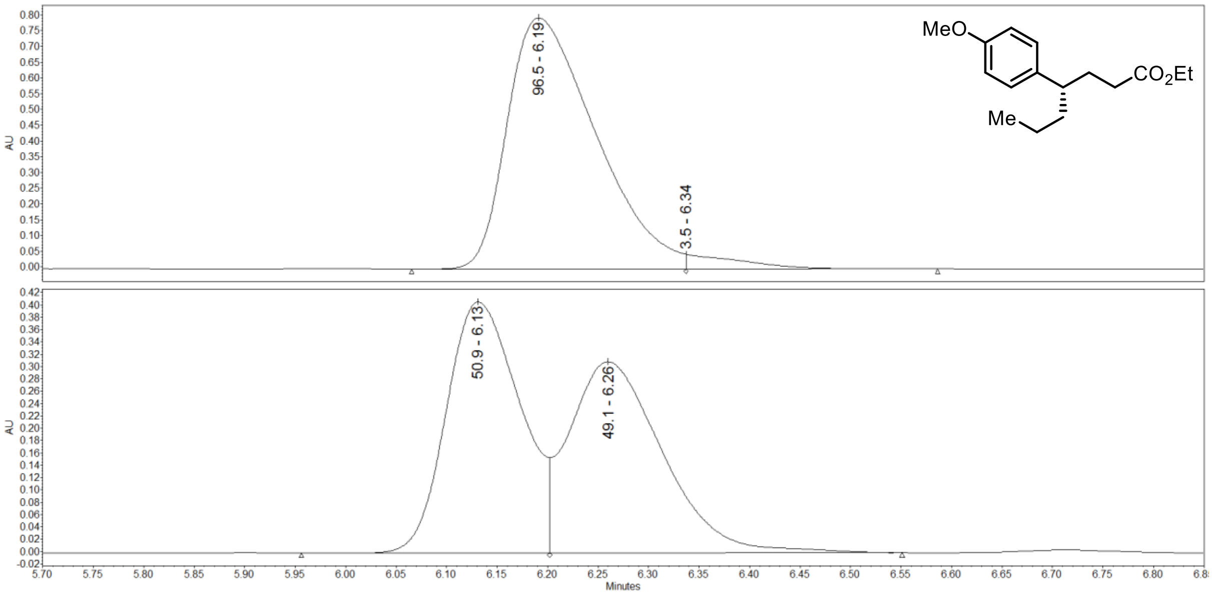

Figure S96. SFC trace for (*S*)-*red*-**5a** and (±)-*red*-**5a**.

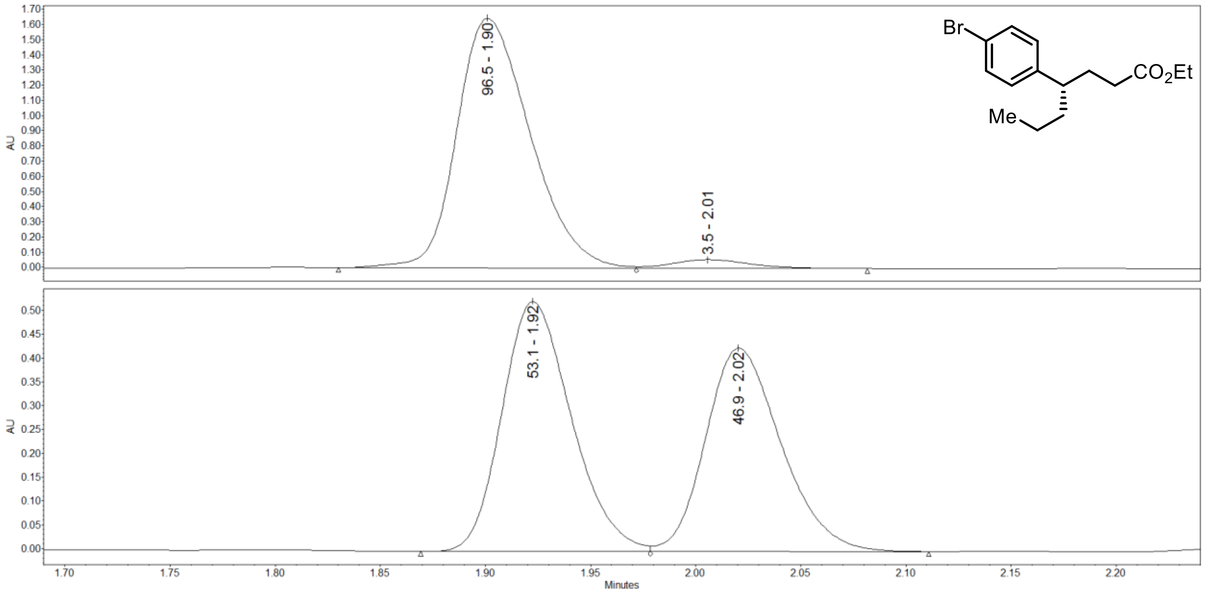

Figure S97. SFC trace for (*S*)-*red*-**5b** and (±)-*red*-**5b**.

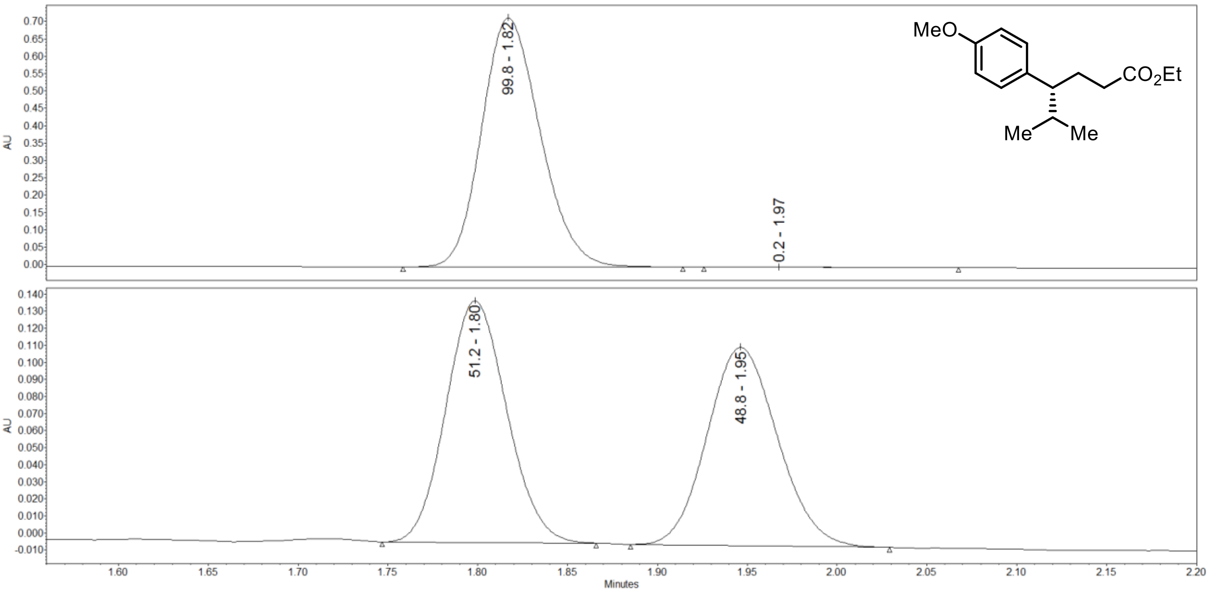

Figure 11.28 SFC trace for (*S*)-*red*-**5c** and (±)-*red*-**5c**.

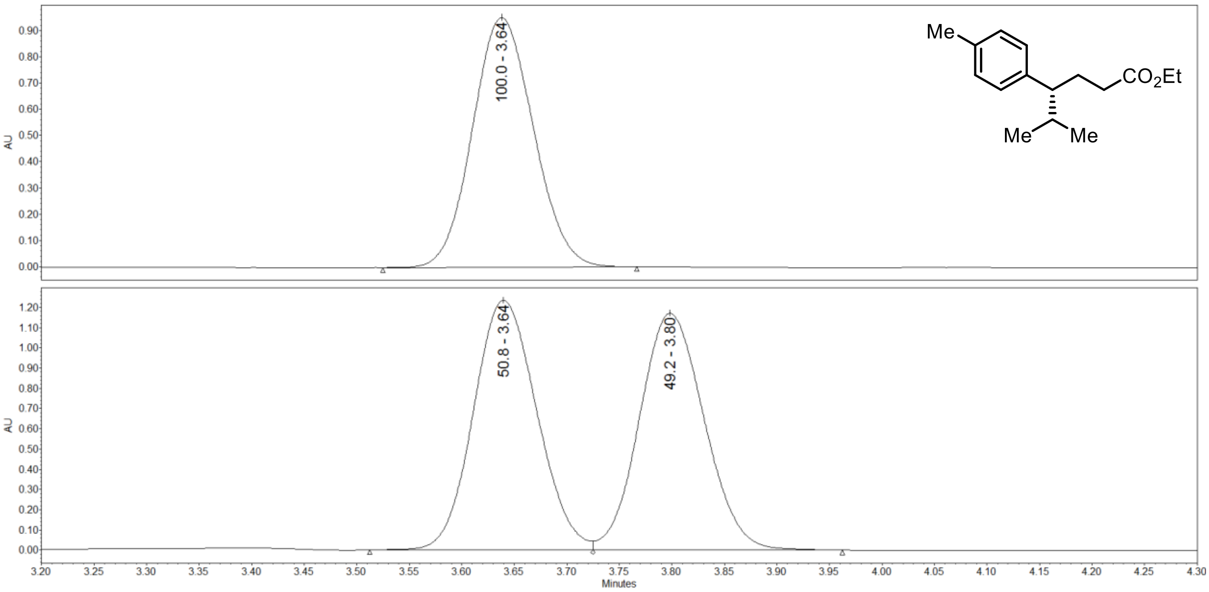

Figure 11.29 SFC trace for (*S*)-*red*-**5d** and (±)-*red*-**5d**.

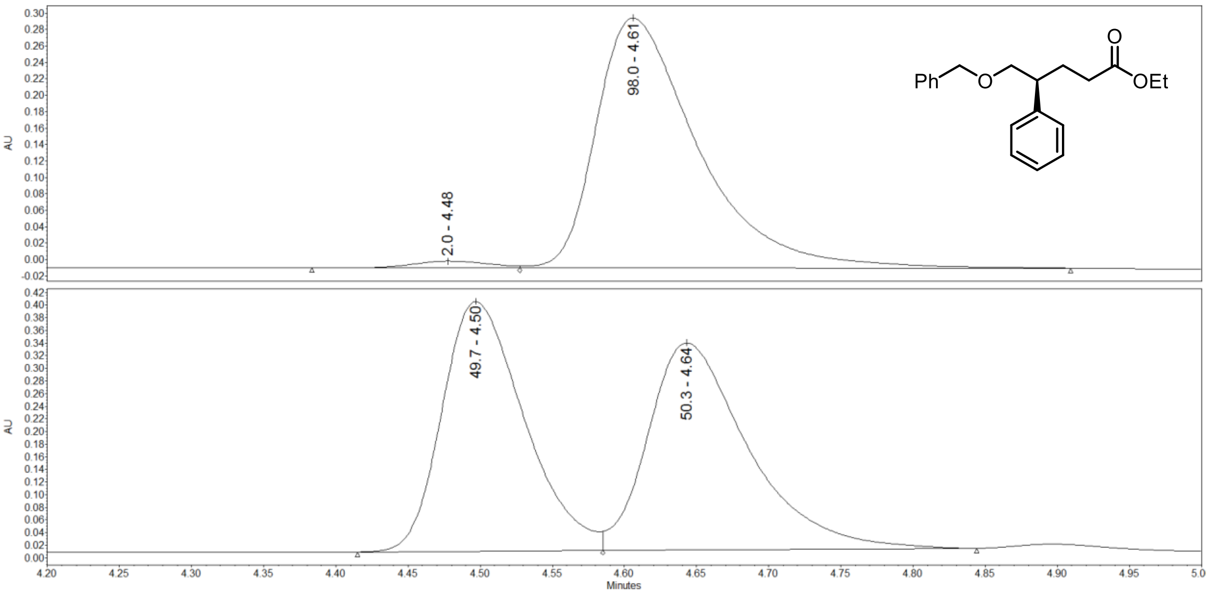

Figure 11.30 SFC trace for (*S*)-*red*-**5e** and (±)-*red*-**5e**.

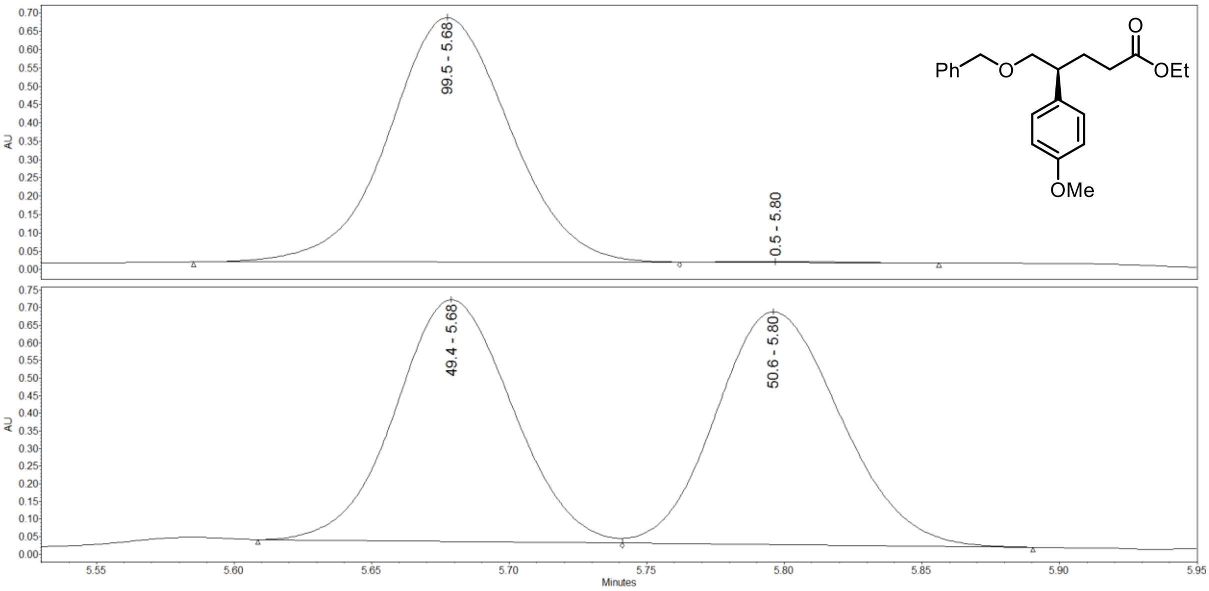

Figure 11.31 SFC trace for (*S*)-*red*-**5f** and (±)-*red*-**5f**.

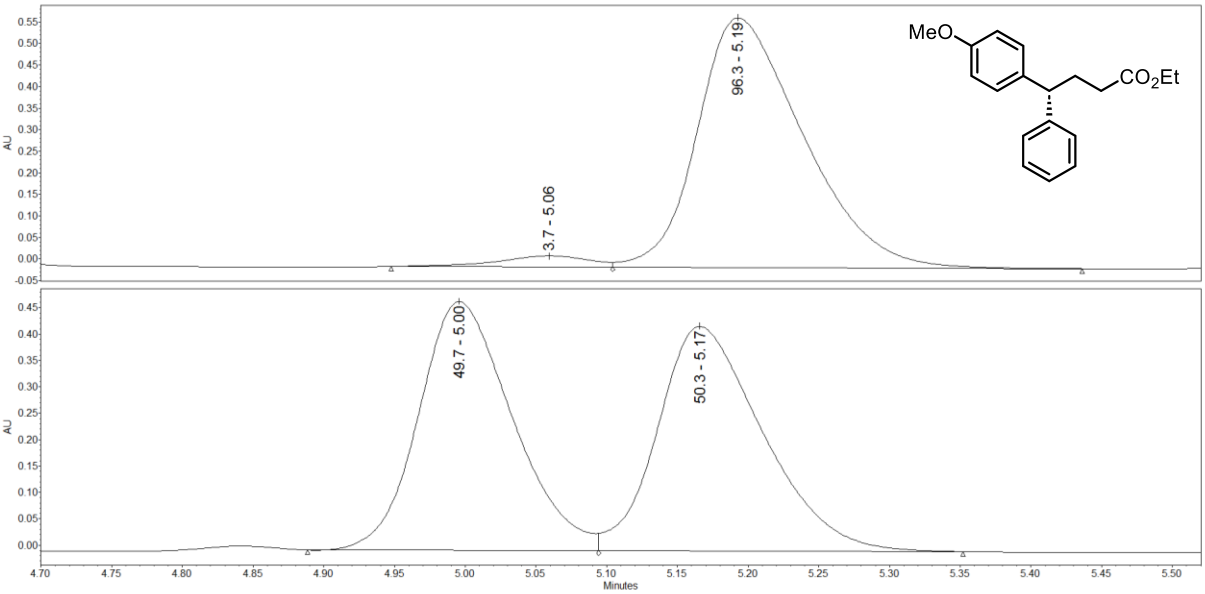

Figure 11.32 SFC trace for (*S*)-*red*-**5g** and (±)-*red*-**5g**.

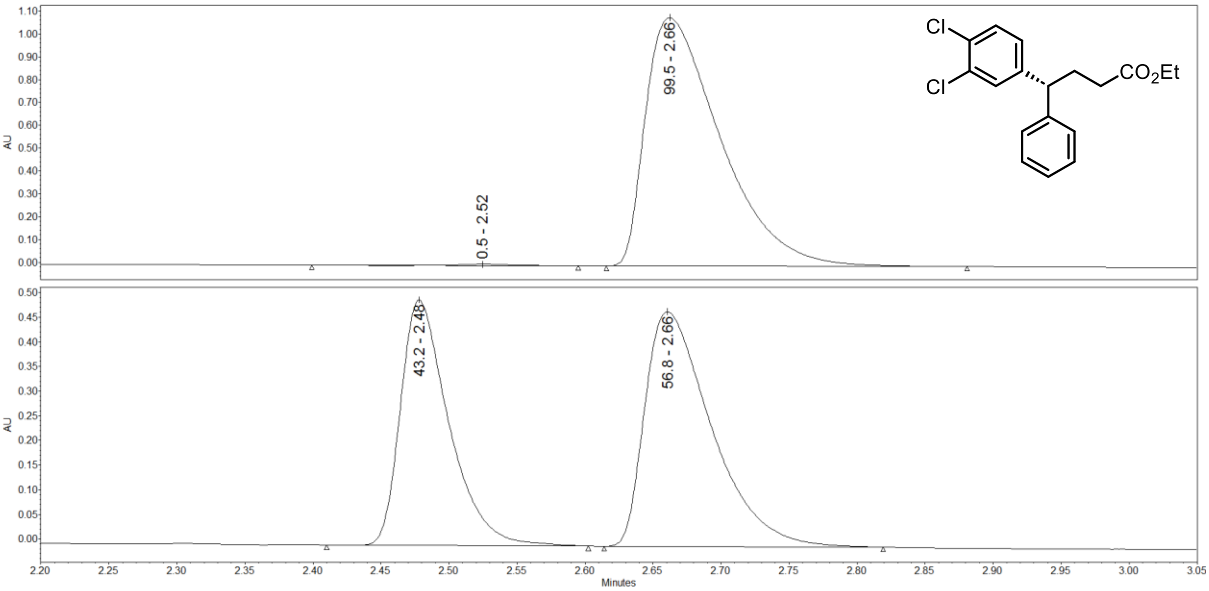

Figure 11.33 SFC trace for (*S*)-*red*-**5h** and (±)-*red*-**5h**.

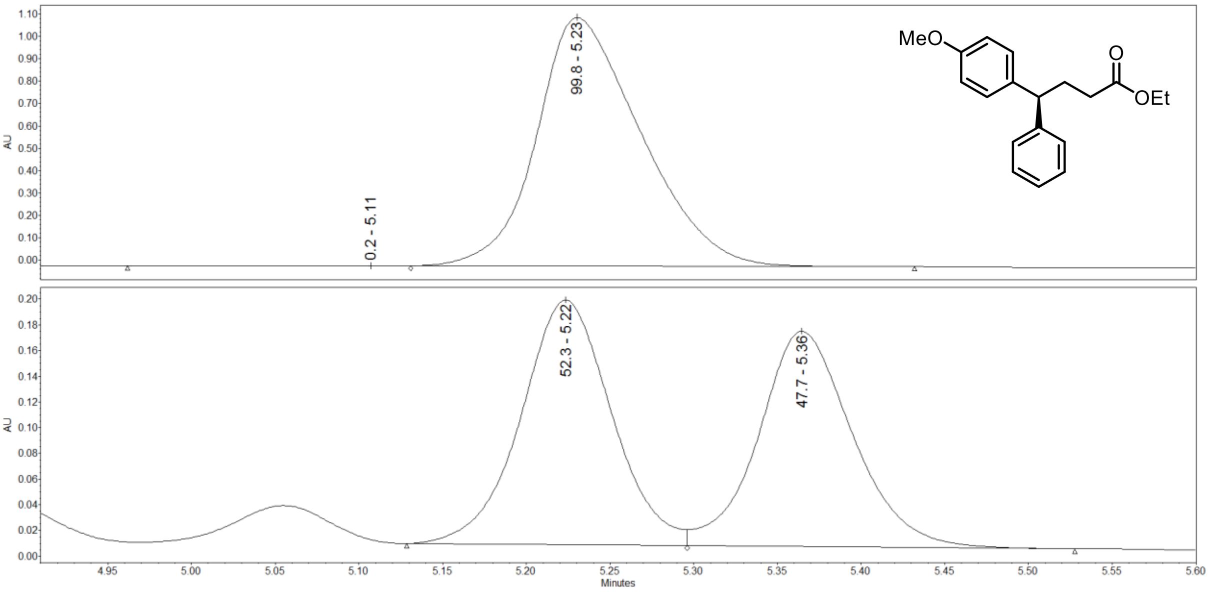

Figure 11.34 SFC trace for (*S*)-*red*-**5i** and (±)-*red*-**5i**.

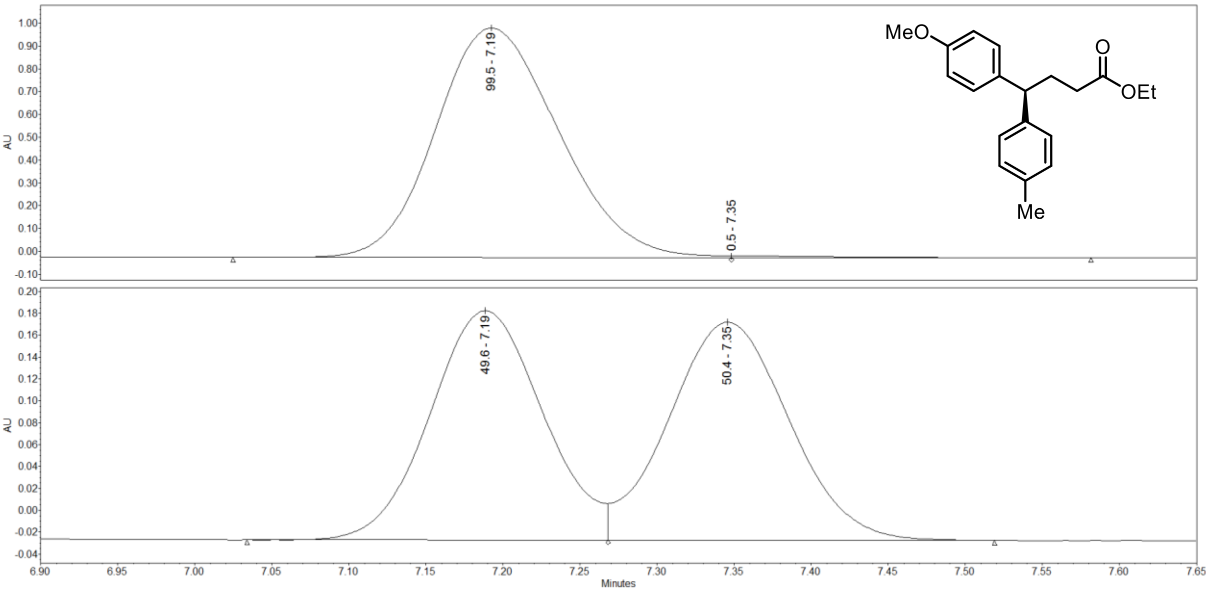

Figure 11.35 SFC trace for (*S*)-*red*-**5j** and (±)-*red*-**5j**.

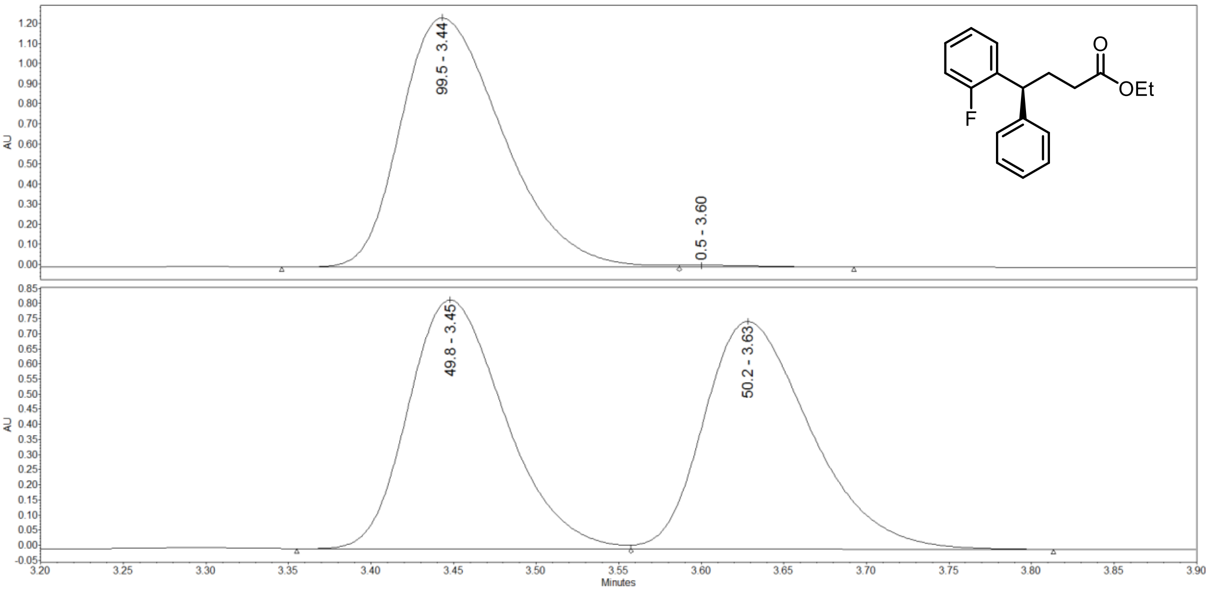

Figure 11.36 SFC trace for (*S*)-*red*-**5k** and (±)-*red*-**5k**.

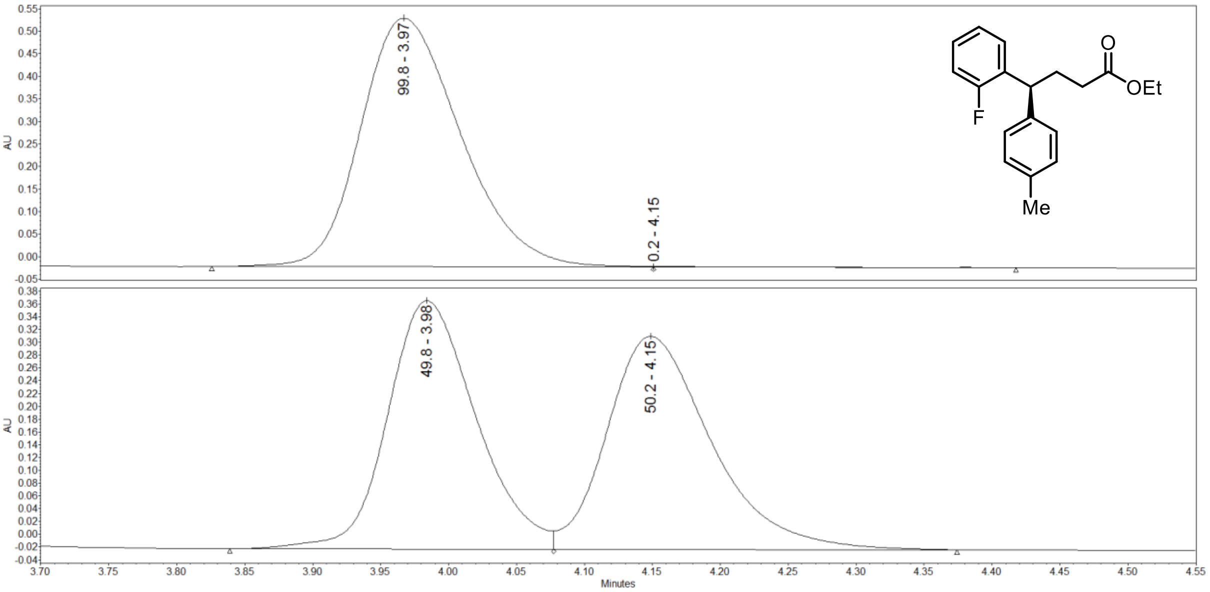

Figure 11.37 SFC trace for (*S*)-*red*-**5l** and (±)-*red*-**5l**.

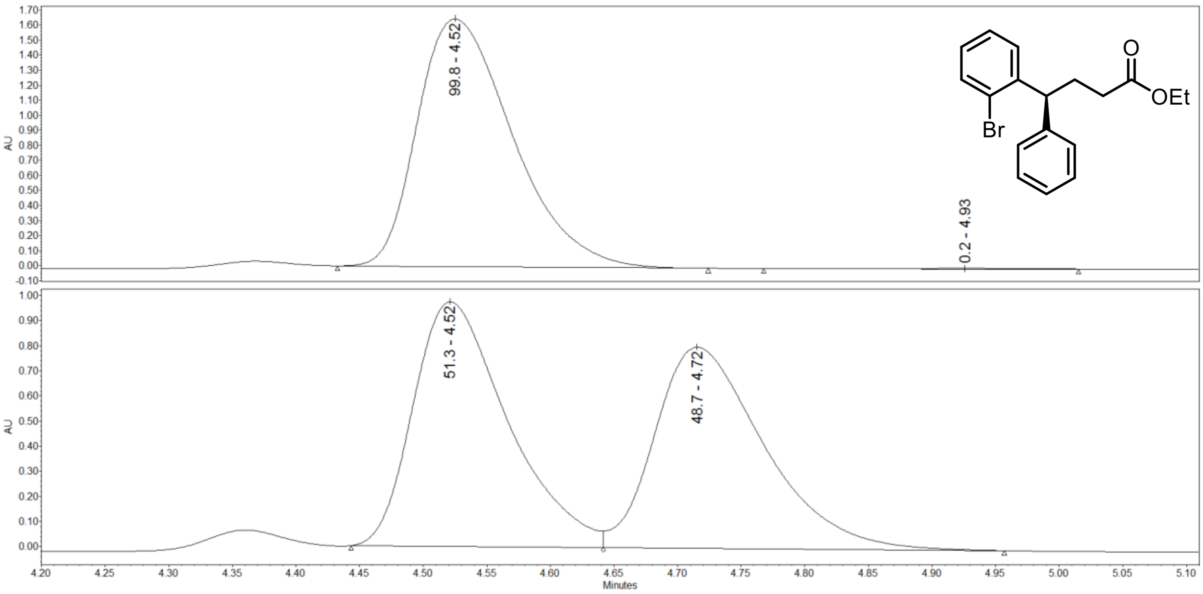

Figure 11.38 SFC trace for (*S*)-*red*-**5m** and (±)-*red*-**5m**.

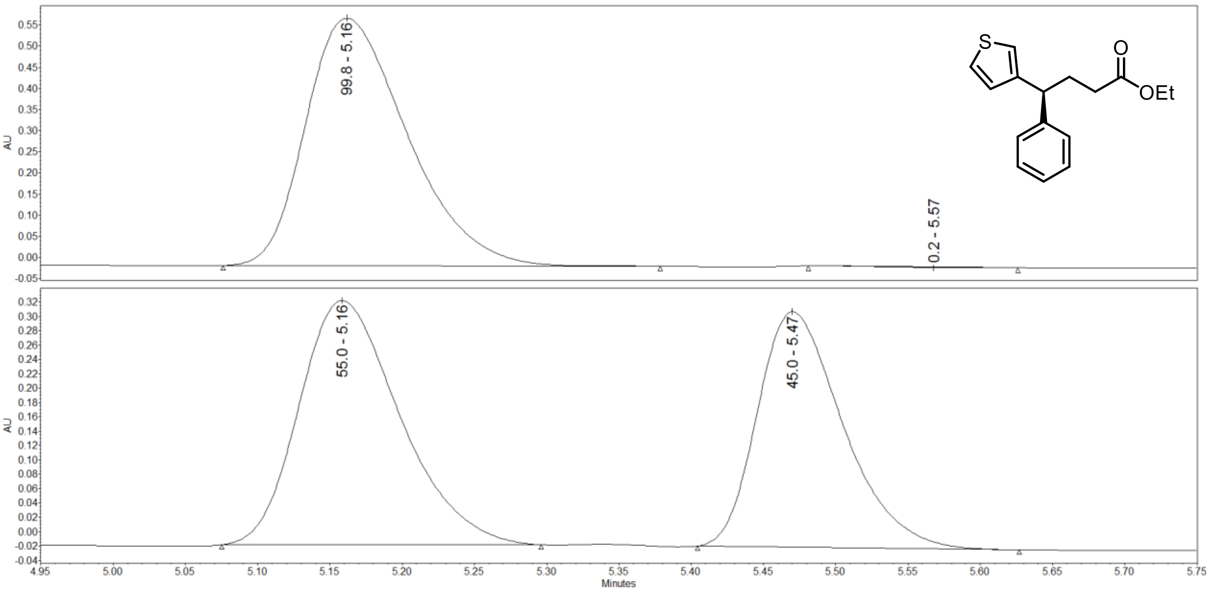

Figure 11.39 SFC trace for (*S*)-*red*-**5n** and (±)-*red*-**5n**.

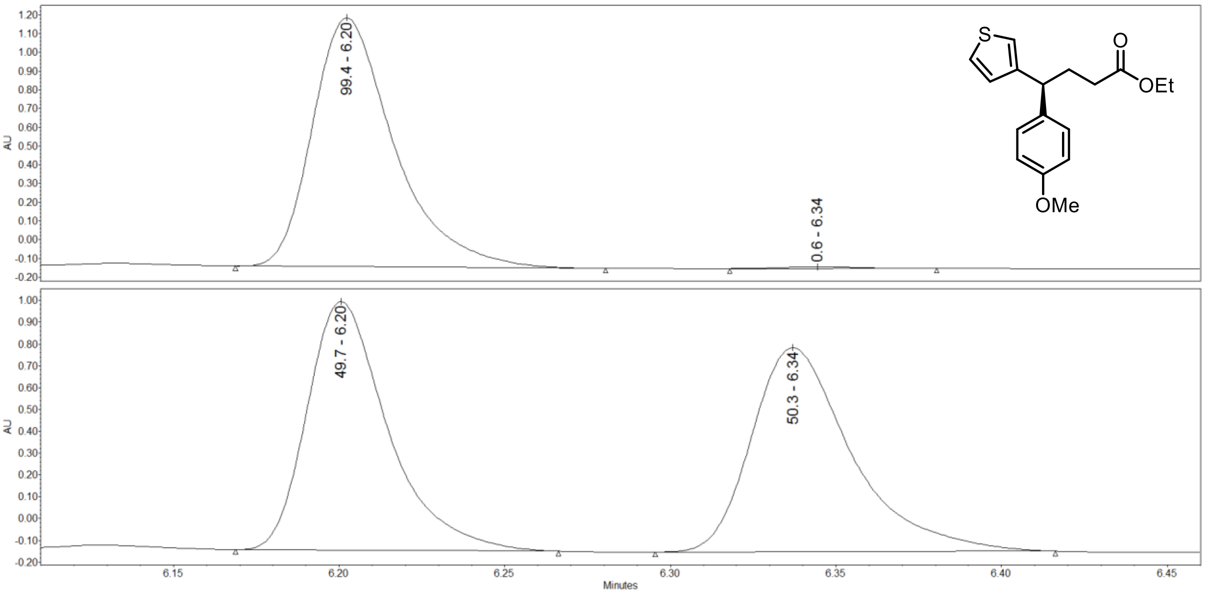

Figure 11.40 SFC trace for (*S*)-*red*-**5o** and (±)-*red*-**5o**.

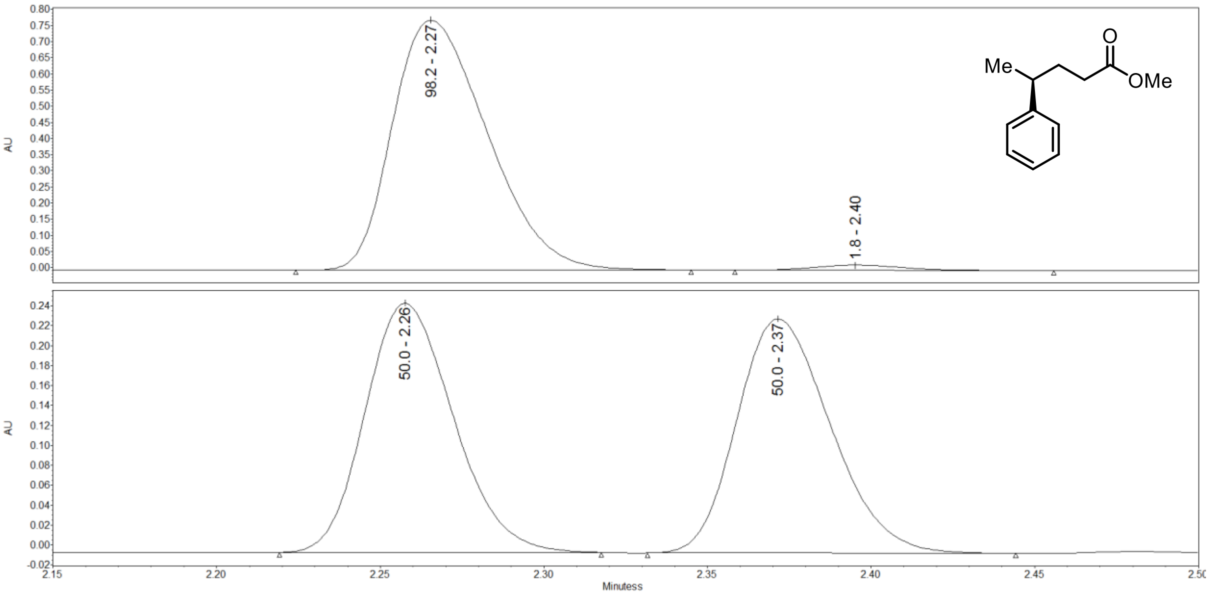

Figure 11.41 SFC trace for (*S*)-*red*-**5p** and (±)-*red*-**5p**.

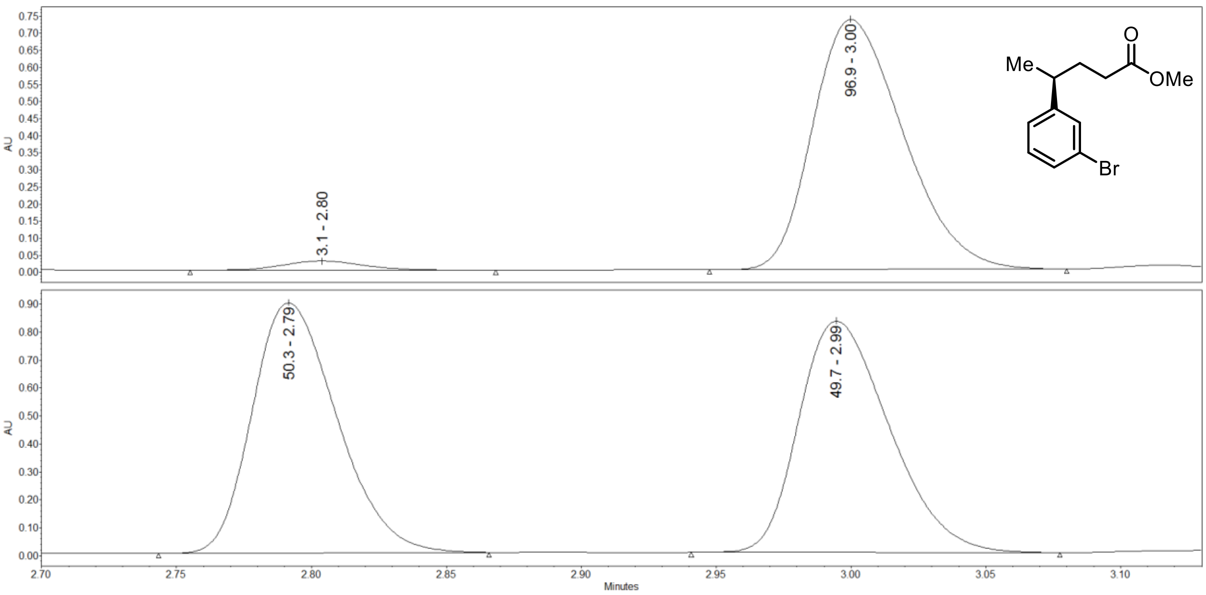

Figure 11.42 SFC trace for (*S*)-*red*-**5q** and (±)-*red*-**5q**.

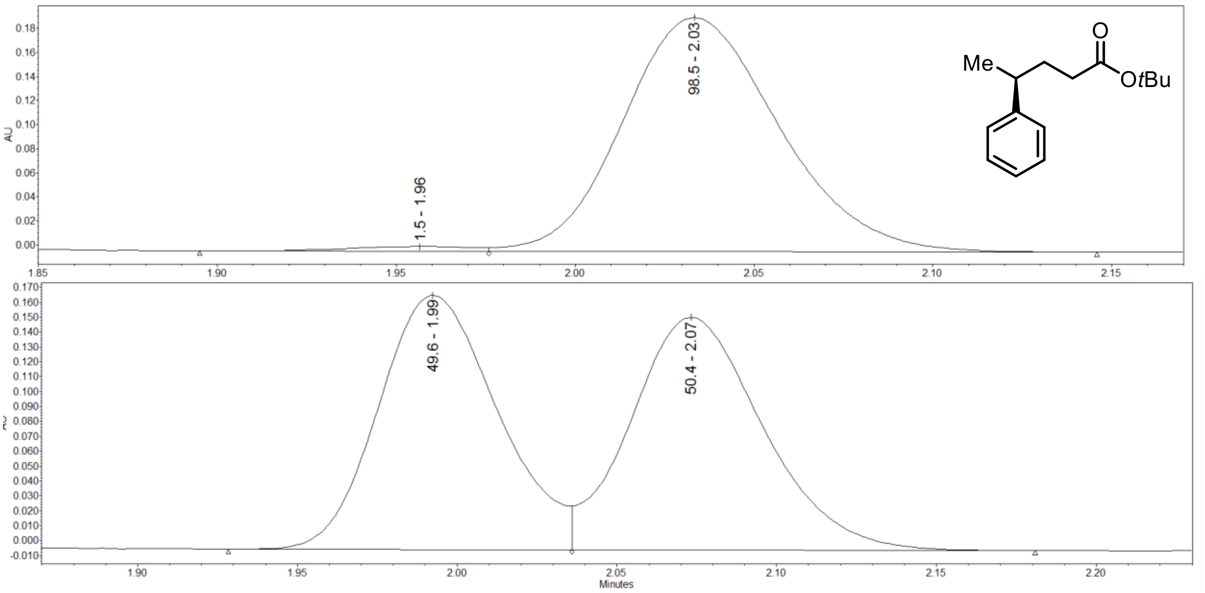

Figure 11.43 SFC trace for (*S*)-*red*-**5r** and (±)-*red*-**5r**.

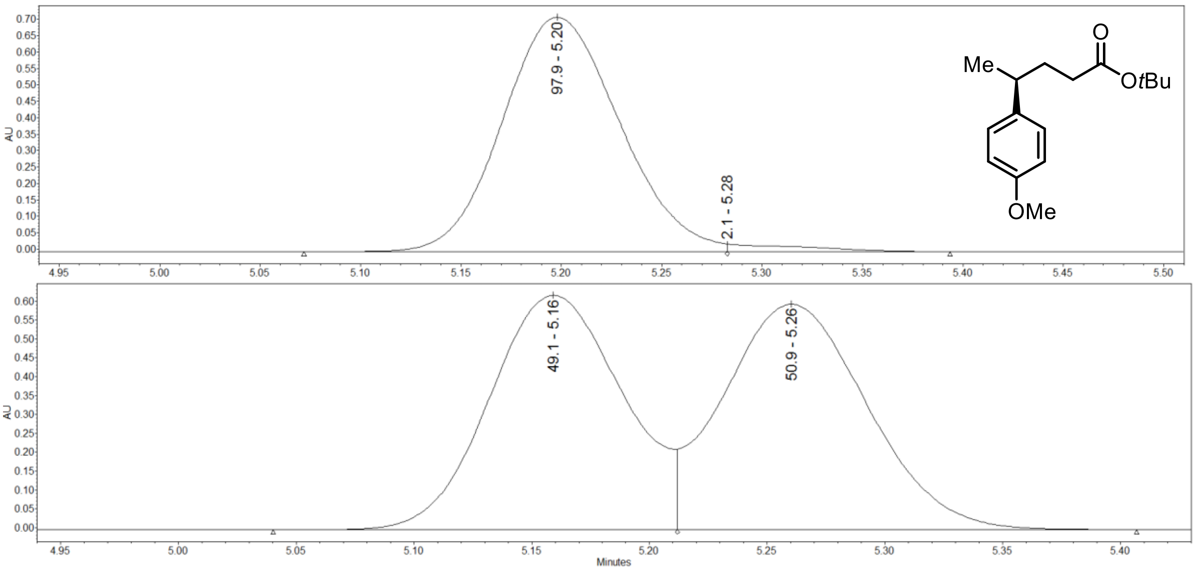

Figure 11.44 SFC trace for (*S*)-*red*-**5s** and (±)-*red*-**5s**.

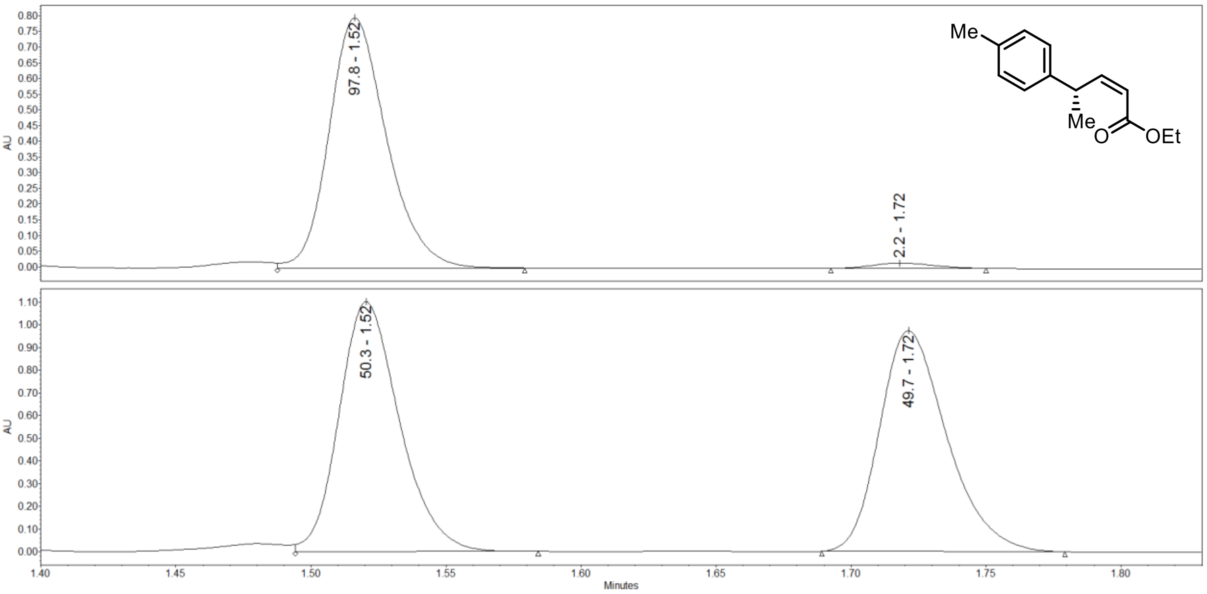

Figure 11.45 SFC trace for (*S*)-*Z*-**3d** and (±)-*Z*-**3d**.

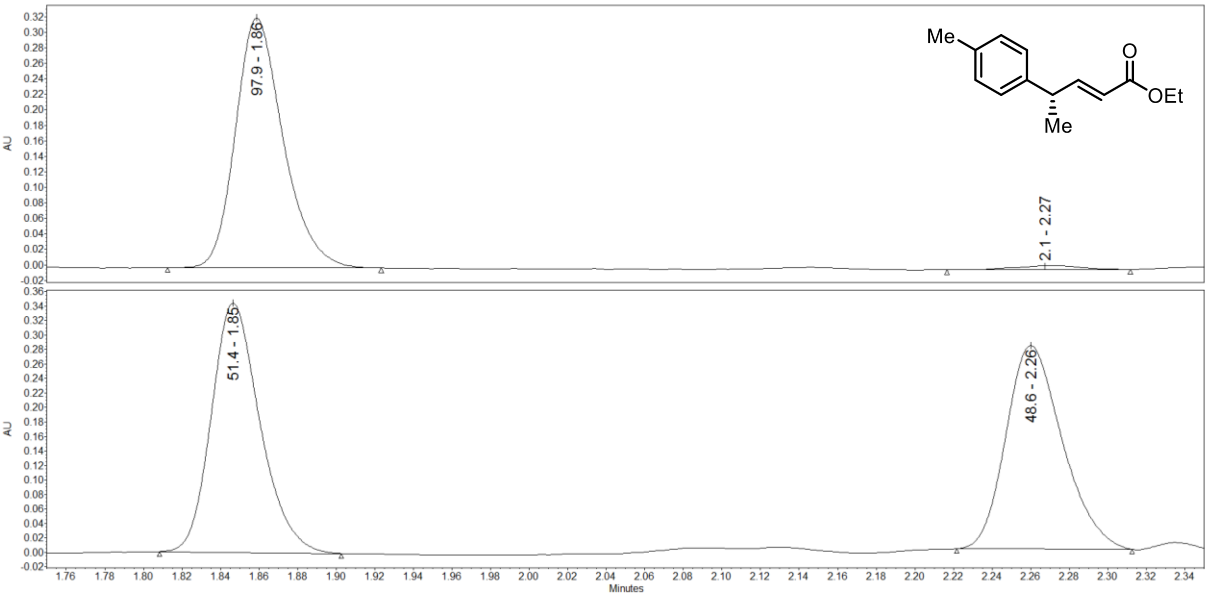

Figure 11.46 SFC trace for (*S*)-*E*-**3d** and (±)-*E*-**3d**.

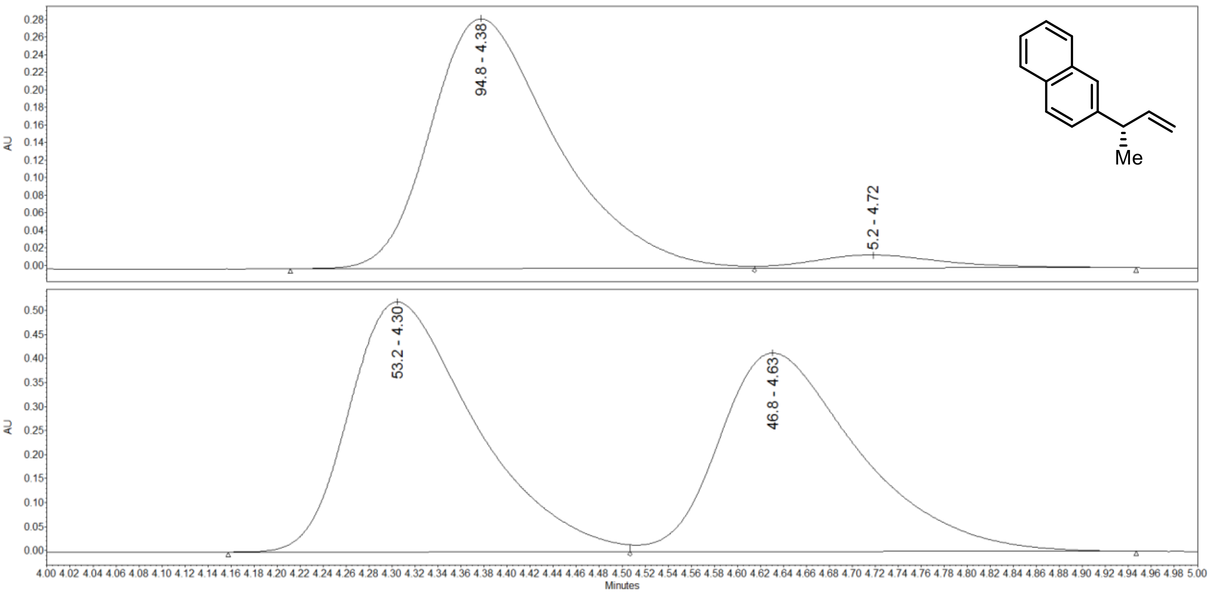

Figure 11.47 SFC trace for (*S*)-**6** and (±)-**6**.

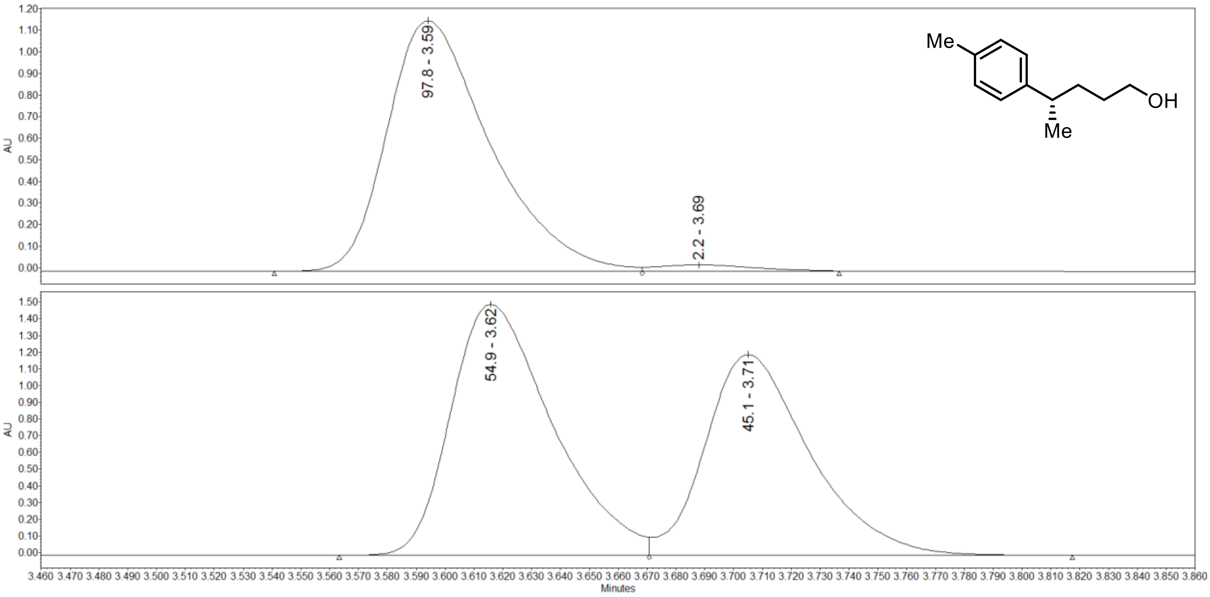

Figure 11.48 SFC trace for (*S*)-**9** and (±)-**9**.

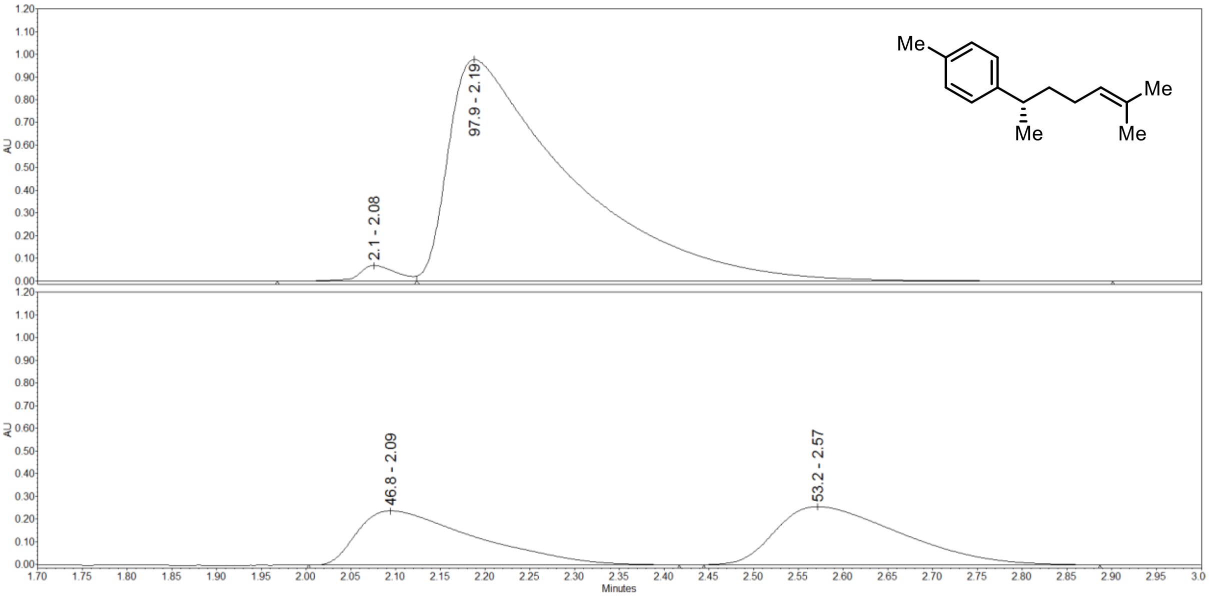

Figure 11.49 SFC trace for (*S*)-**10** and (±)-**10**.

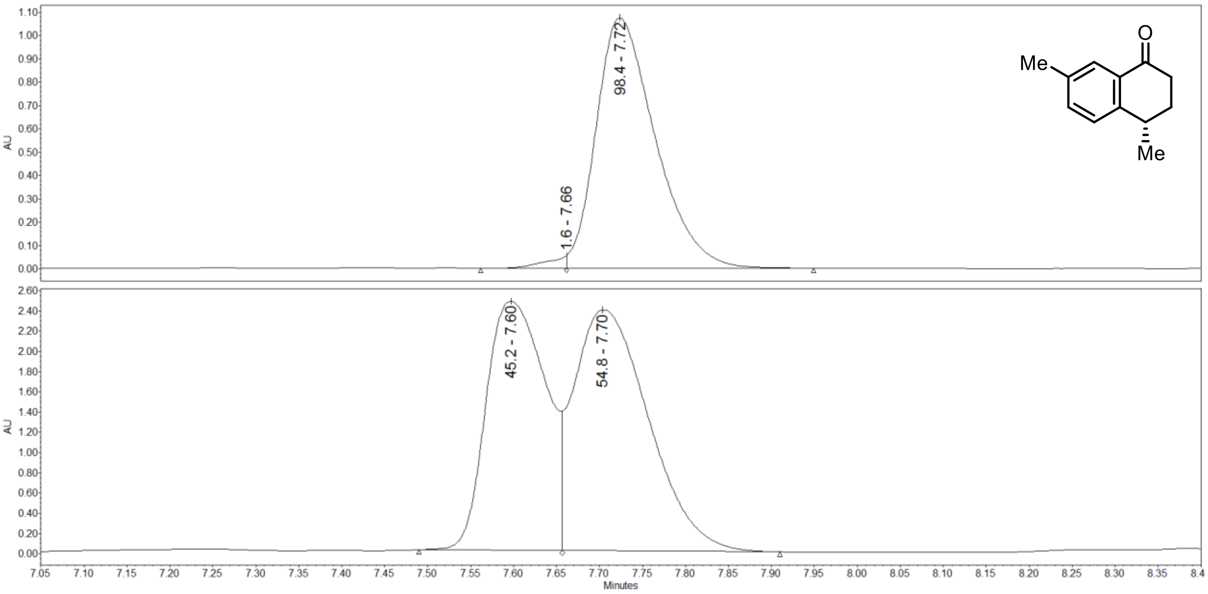

Figure 11.50 SFC trace for (*S*)-**11** and (±)-**11**.

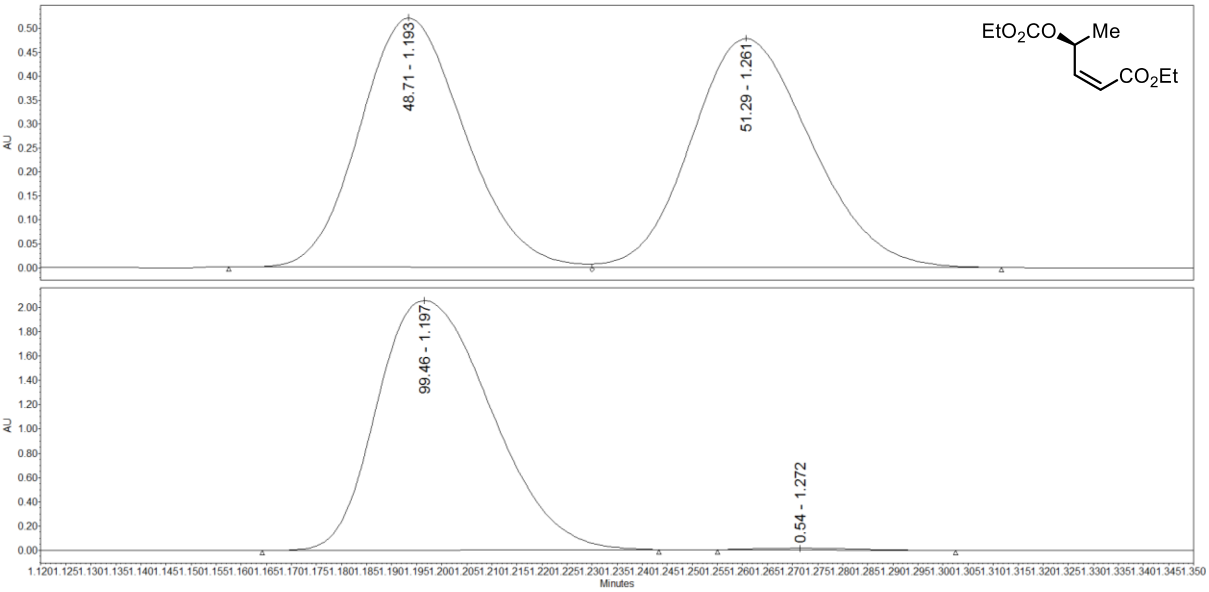

Figure 11.51 SFC trace for (*S*)-**1** (used in mechanistic studies) and (±)-**1**.

12. References

1 Yadav, J. S., Basak, A. K. & Srihari, P. An aldol approach to the synthesis of the anti-tubercular agent erogorgiaene. *Tetrahedron Lett.* **48**, 2841-2843 (2007).

2 Do, H. Q., Chandrashekar, E. R. & Fu, G. C. Nickel/bis(oxazoline)-catalyzed asymmetric Negishi arylations of racemic secondary benzylic electrophiles to generate enantioenriched 1,1-diarylalkanes. *J. Am. Chem. Soc.* **135**, 16288-16291 (2013).

3 Kc, S., Dhungana, R. K., Khanal, N. & Giri, R. Nickel-Catalyzed alpha-Carbonylalkylarylation of Vinylarenes: Expedient Access to gamma,gamma-Diarylcarbonyl and Aryltetralone Derivatives. *Angew. Chem. Int. Ed.* **59**, 8047-8051 (2020).

4 Alexakis, A., Hajjaji, S. E., Polet, D. & Rathgeb, X. Iridium-catalyzed asymmetric allylic substitution with aryl zinc reagents. *Org. Lett.* **9**, 3393-3395 (2007).

5 Takano, S., Yanase, M., Sugihara, T. & Ogasawara, K. Enantiodivergent route to the aromatic bisabolane sesquiterpenes by regio- and stereo-controlled epoxide opening. *J. Chem. Soc., Chem. Commun.*, 1538-1540 (1988).

6 Yang, S., Zhu, S. F., Guo, N., Song, S. & Zhou, Q. L. Carboxy-directed asymmetric hydrogenation of alpha-alkyl-alpha-aryl terminal olefins: highly enantioselective and chemoselective access to a chiral benzylmethyl center. *Org. Biomol. Chem.* **12**, 2049-2052 (2014).

7 Fernandes, R. A., Gangani, A. J. & Kunkalkar, R. A. Metal-free annulative hydrosulfonation of propiolate esters: synthesis of 4-sulfonates of coumarins and butenolides. *New J. Chem.* **44**, 3970-3984 (2020).

8 Kojima, N., Nishijima, S., Tsuge, K. & Tanaka, T. Asymmetric alkynylation of aldehydes with propiolates without high reagent loading and any additives. *Org. Biomol. Chem.* **9**, 4425-4428 (2011).

9 Ramón, R. S., Pottier, C., Gómez-Suárez, A. & Nolan, S. P. Gold(I)-Catalyzed Tandem Alkoxylation/Lactonization of γ-Hydroxy-α,β-Acetylenic Esters. *Adv. Synth. Catal.* **353**, 1575-1583 (2011).

10 Trost, B. M. & Quintard, A. Asymmetric catalytic synthesis of the proposed structure of trocheliophorolide B. *Org. Lett.* **14**, 4698-4700 (2012).

11 Tsui, G. C., Villeneuve, K., Carlson, E. & Tam, W. Ruthenium-Catalyzed [2+2] Cycloadditions between Norbornene and Propargylic Alcohols or Their Derivatives. *Organometallics* **33**, 3847-3856 (2014).

12 Downey, C. W., Mahoney, B. D. & Lipari, V. R. Trimethylsilyl Trifluoromethanesulfonate-Accelerated Addition of Catalytically Generated Zinc Acetylides to Aldehydes. *J. Org. Chem.* **74**, 2904-2906 (2009).

13 Pohlman, M., Kazmaier, U. & Lindner, T. Allylic alkylation versus Michael induced ring closure: chelated enolates as versatile nucleophiles. *J. Org. Chem.* **69**, 6909-6912 (2004).

14 Du, Z. *et al.* Enantioselective synthesis of (+)-nuciferal, (+)-(E)-nuciferol and (+)-α-curcumene by chiral hydrogenesterification reaction. *J. Chem. Res.* **2004**, 427-429 (2004).

15 Nguyen, T. N. T., Thiel, N. O. & Teichert, J. F. Copper(i)-catalysed asymmetric allylic reductions with hydrosilanes. *Chem. Commun.* **53**, 11686-11689 (2017).

16 Kiuchi, H., Takahashi, D., Funaki, K., Sato, T. & Oi, S. Rhodium-catalyzed asymmetric coupling reaction of allylic ethers with arylboronic acids. *Org. Lett.* **14**, 4502-4505 (2012).

17 Garcia Ruano, J. L., Schopping, C., Alvarado, C. & Aleman, J. Synthesis of unfunctionalized carbonated fragments containing two vicinal chiral centers: stereocontrolled benzylation of vinylsulfones mediated by a remote sulfinyl group. *Chem. Eur. J.* **16**, 8968-8971 (2010).

18 Aggarwal, V. K. *et al.* Application of the lithiation-borylation reaction to the rapid and enantioselective synthesis of the bisabolane family of sesquiterpenes. *Chem. Commun.* **48**, 9230-9232 (2012).

19 Wu, L. *et al.* Asymmetric synthesis of (R)-ar-curcumene, (R)-4,7-dimethyl-l-tetralone, and their enantiomers via cobalt-catalyzed asymmetric Kumada cross-coupling. *Tetrahedron: Asymmetry* **27**, 78-83 (2016).

20 Skotnitzki, J. *et al.* Stereoselective Csp(3)-Csp(2) Cross-Couplings of Chiral Secondary Alkylzinc Reagents with Alkenyl and Aryl Halides. *Angew. Chem. Int. Ed.* **59**, 320-324 (2020).

21 Chavan, S. P. & Khatod, H. S. Enantioselective synthesis of the essential oil and pheromonal component ar-himachalene by a chiral pool and chirality induction approach. *Tetrahedron: Asymmetry* **23**, 1410-1415 (2012).

22 Elford, T. G., Nave, S., Sonawane, R. P. & Aggarwal, V. K. Total synthesis of (+)-erogorgiaene using lithiation-borylation methodology, and stereoselective synthesis of each of its diastereoisomers. *J. Am. Chem. Soc.* **133**, 16798-16801 (2011).

23 Takayama, H. *et al.* Discovery of inhibitors of the Wnt and Hedgehog signaling pathways through the catalytic enantioselective synthesis of an iridoid-inspired compound collection. *Angew. Chem. Int. Ed.* **52**, 12404-12408 (2013).

24 Shimokawa, J., Harada, T., Yokoshima, S. & Fukuyama, T. Total synthesis of gelsemoxonine. *J. Am. Chem. Soc.* **133**, 17634-17637 (2011).

25 Wang, H. Y., Yang, K., Bennett, S. R., Guo, S. R. & Tang, W. Iridium-Catalyzed Dynamic Kinetic Isomerization: Expedient Synthesis of Carbohydrates from Achmatowicz Rearrangement Products. *Angew. Chem. Int. Ed.* **54**, 8756-8759 (2015).

26 Turrini, N. G. *et al.* Biocatalytic access to nonracemic γ-oxo esters via stereoselective reduction using ene-reductases. *Green Chem.* **19**, 511-518 (2017).

27 Brenna, E. *et al.* Biocatalytic synthesis of chiral cyclic γ-oxoesters by sequential C–H hydroxylation, alcohol oxidation and alkene reduction. *Green Chem.* **19**, 5122-5130 (2017).

28 Shukla, K. H. & DeShong, P. Studies on the mechanism of allylic coupling reactions: a hammett analysis of the coupling of aryl silicate derivatives. *J. Org. Chem.* **73**, 6283-6291 (2008).

29 Son, S. & Fu, G. C. Nickel-catalyzed asymmetric Negishi cross-couplings of secondary allylic chlorides with alkylzincs. *J. Am. Chem. Soc.* **130**, 2756-2757 (2008).

30 Rodríguez, S., Vidal, A., Monroig, J. J. & González, F. V. Diastereoselectivity in the epoxidation of γ-hydroxy α,β-unsaturated esters: temperature and solvent effect. *Tetrahedron Lett.* **45**, 5359-5361 (2004).

31 Prudel, C., Huwig, K. & Kazmaier, U. Stereoselective Allylic Alkylations of Amino Ketones and Their Application in the Synthesis of Highly Functionalized Piperidines. *Chem. Eur. J.* **26**, 3181-3188 (2020).
